# Supplementary material for: Electron density-based GPT for optimization and suggestion of host–guest binders
Source: Nat Comput Sci. 2024 Mar 8;4(3):200–9. doi: 10.1038/s43588-024-00602-x (PMC10965440; doi:10.1038/s43588-024-00602-x)
Supplement: Supplementary file 1 — Supplementary Sections 1–4, Tables 1–48 and Figs. 1–291. [file 43588_2024_602_MOESM1_ESM.pdf]

nature computational science

Article https://doi.org/10.1038/s43588-024-00602-x

Electron density-based GPT for optimization and suggestion of host–guest binders

In the format provided by the authors and unedited

Contents

Supplementary Section 1 Source code and machine learning algorithms 5

Supplementary Subsection 1.1 Software installation 5
Supplementary Subsection 1.2 Note about Tensorflow versions 6
Supplementary Subsection 1.3 Generating the training dataset 6
1.3.1 bin/dataset/generate_dataset.py 7
1.3.2 src/datasets/QM9.py generate() 7
1.3.3 src/datasets/QM9.py _compute_electron_density() 8
Supplementary Subsection 1.4 src/models/layers.py 10
1.4.1 Residual connections 10
1.4.2 Add and drop dimension layers 10
1.4.3 Attention layer 10
1.4.4 Transformer block 10
Supplementary Subsection 1.5 Using a Variational autoencoder (VAE) to model the QM9 dataset 10
1.5.1 src/models/VAE.py 11
1.5.2 src/models/VAEresnet.py 14
1.5.3 src/models/VAEattention.py 14
1.5.4 bin/train/train_vae.py 14
Supplementary Subsection 1.6 Using a Fully Convolutional Network (FCN) to convert electron densities to electrostatic potentials 18
1.6.1 src/models/ED2ESP.py 18
1.6.2 bin/train/train_ed2esp.py 19
Supplementary Subsection 1.7 Predicting numerical properties from electron densities 21
1.7.1 src/models/CNN3D_featureprediction.py 22
1.7.2 bin/train/train_property_prediction.py 22
Supplementary Subsection 1.8 Decorating electron densities with electrostatic potentials 22
Supplementary Subsection 1.9 Decreasing the size (volume) of the cavity . . 24
Supplementary Subsection 1.10 Using the GPT model to generate SMILES sequences 24
1.10.1 src/models/gpt.py 26
1.10.2 Generating new SMILES sequences: greedy vs probabilistic sampling 27
1.10.3 bin/train/train_gpt.py 27
Supplementary Subsection 1.11 Using the Transformer model to generate SMILES sequences from electron densities and/or electrostatic potentials 29
1.11.1 src/models/ED2smiles.py 29
1.11.2 src/models/ESP2smiles.py 30
1.11.3 src/models/ED_ESP2smiles.py 34
1.11.4 bin/train/train_e2s.py esp2s.py edesp2smiles.py 34
1.11.5 Testing the accuracy of the electron density to SMILES translation 35
1.11.6 Translating electron densities into SMILES using a trained model 37

Supplementary Subsection 1.12 Using the Transformer model to generate SELFIES sequences from electron densities and/or electrostatic potentials 38

Supplementary Section 2 Optimisation algorithms and optimisation results 38
Supplementary Subsection 2.1 src/utils/optimiser_utils.py 39
2.1.1 Generating the initial population of guest 39
2.1.2 Fitness functions 40
2.1.3 Loading hosts 44
2.1.4 Loading the machine learning models 44
Supplementary Subsection 2.2 Optimisation scripts 45
2.2.1 bin/optimisers/host_guest_overlapping.py 46
2.2.2 bin/optimisers/maximise_size.py 47
2.2.3 bin/optimisers/maximise_polar.py 48
2.2.4 bin/optimisers/maximise_hg_polar.py 50
2.2.5 bin/optimisers/maximise_hg_esp.py 51
2.2.6 bin/optimisers/cage_hg.py 55
2.2.7 bin/optimisers/cage_hg_esp.py 57
2.2.8 bin/optimisers/cage_hg_esp_lee.py 57
2.2.9 bin/optimisers/cage_hg_split_esp.py 60
Supplementary Subsection 2.3 Optimisation results 62
2.3.1 Results from bin/optimisers/host_guest_overlapping.py 62
2.3.2 Results from bin/optimisers/maximise_size.py 67
2.3.3 Results from bin/optimisers/maximise_polar.py 67
2.3.4 Results from bin/optimisers/maximise_hg_polar.py 67
2.3.5 Results from bin/optimisers/maximise_hg_esp.py 75
2.3.6 Results from bin/optimisers/cage_hg.py 95
2.3.7 Results from bin/optimisers/cage_hg_esp.py 95
2.3.8 Results from bin/optimisers/cage_hg_esp_lee.py 127
2.3.9 Results from bin/optimisers/cage_hg_split_esp.py 153
2.3.10 SELFIES results from bin/optimisers/maximise_hg_esp.py . . . 153
2.3.11 SELFIES results from bin/optimisers/cage_hg_esp_lee.py 193

Supplementary Section 3 Experimental validation 238
Supplementary Subsection 3.1 General experimental section 238
3.1.1 Solvents and commercial reagents 238
3.1.2 NMR 238
Supplementary Subsection 3.2 Synthesis 238
3.2.1 Synthesis of ligand 1 238
3.2.2 Synthesis of cage $[Pd_2\mathbf{1}_4](BArF)_4$ 238
Supplementary Subsection 3.3 Cucurbituril CB[6] guest binding tritations . . 239
3.3.1 Overview and curve-fitting function 239
3.3.2 Titration data for Cucurbituril CB[6] 240
Supplementary Subsection 3.4 Cage $[Pd_2\mathbf{1}_4](BArF)_4$ guest binding tritations 249
3.4.1 Overview and curve-fitting function 249
3.4.2 Titration data for Cage $[Pd_2\mathbf{1}_4](BArF)_4$ 249

Supplementary Section 4 Benchmark 255

Supplementary Subsection 4.1 Electron Density (ED) 267
4.1.1 ED 0.5 270
4.1.2 ED 1.0 272
4.1.3 ED 1.5 274
4.1.4 ED 2.0 276
4.1.5 ED 2.5 278
4.1.6 ED 5.0 280
4.1.7 ED 10.0 282
4.1.8 ED 25.0 283
4.1.9 ED 50.0 284
Supplementary Subsection 4.2 Electron densities decorated with Electrostatic Potentials (ED ESP) 286
4.2.1 ED ESP 0.5 289
4.2.2 ED ESP 1.0 291
4.2.3 ED ESP 1.5 293
4.2.4 ED ESP 2.0 294
4.2.5 ED ESP 2.5 295
4.2.6 ED ESP 5.0 297
4.2.7 ED ESP 10.0 299
4.2.8 ED ESP 25.0 301
4.2.9 ED ESP 50.0 303
Supplementary Subsection 4.3 Electron densities (ED) Probabilistic Samplig (proba10) 305
4.3.1 ED proba10 0.5 308
4.3.2 ED proba10 1.0 310
4.3.3 ED proba10 1.5 312
4.3.4 ED proba10 2.0 314
4.3.5 ED proba10 2.5 316
4.3.6 ED proba10 5.0 318
4.3.7 ED proba10 10.0 320
4.3.8 ED proba10 25.0 321
Supplementary Subsection 4.4 ED decorated with Electrostatic Potentials (ED ESP) Probabilistic Sampling (proba10) 322
4.4.1 ED ESP proba10 0.5 325
4.4.2 ED ESP proba10 1.0 327
4.4.3 ED ESP proba10 1.5 329
4.4.4 ED ESP proba10 2.0 330
4.4.5 ED ESP proba10 2.5 331
4.4.6 ED ESP proba10 5.0 333
4.4.7 ED ESP proba10 10.0 335
4.4.8 ED ESP proba10 25.0 337
4.4.9 ED ESP proba10 50.0 339
Supplementary Subsection 4.5 Benchmarking the novelty of the generated guests against the QM9 dataset 341
4.5.1 Benchmarking literature known CB[6] guests against the QM9 dataset 341
4.5.2 Bencharmking literature known $[Pd_2\mathbf{1}_4](BArF)_4$ Cage guests against the QM9 dataset 341

4.5.3 Bencharmking the molecules generated in this research through AI against the QM9 dataset 342

Supplementary Section 1 Source code and machine learning algorithms

Note: Our code heavily relies in the Tensorflow library to execute all the Machine Learning algorithms. This library is frequently updated, and newer versions are not always compatible with the older ones. Most of our development was done with Tensorflow 2.7 and 2.10, and these are the ones we would recommend. Please check Supplementary Section 1.2 for more information.

This supplementary section describes the source code implemented to obtain the results discussed in the main manuscript:

- Supplementary Section 1.1 describes how to install the software.
- Supplementary Section 1.3 describes how to generate the training dataset.
- Supplementary Sections 1.4 to 1.11 describe the different machine learning (ML) algorithms implemented, and the scripts used to test them.

Supplementary Subsection 1.1 Software installation

Before installing the software, it is recommended to have Python installed, including Pip. It is also recommended to have Git and Conda (or Miniconda) installed. To run the different machine learning algorithms, it is recommended to have a powerful GPU (this research used Nvidia RTX 3090 and A6000 GPUs), and to have installed the Cuda (`https://developer.nvidia.com/cuda-toolkit`) and CuDNN (`https://developer.nvidia.com/cudnn`) libraries.

To install the software, the following steps should be executed:

1. Using Git, clone the main repository:

```
$ git clone git@github.com:croningp/electrondensity2.git
```

2. The previous command will download all the source code, including a file named "environment.yml". We can invoke Conda with this file to install all the required libraries:

```
$ conda env create -f environment.yaml
```

3. This command will create a Conda environment named "electrondensity".
4. Modify the environmental variables in "env.sh" to suit your configuration:
   - *DATA_DIR* is the path where to store the datasets.
   - *LOG_DIR* is the path where the files generated by the ML algorithms (such as network weights or sampling outputs) will be saved.
   - *MODEL_DIR* is the path where to save the ML models.
   - *CPU_COUNT* specifies the number of CPUs used for the parallel processing of datasets. Setting it to "-1" means that all the available CPUs will be used.

5. In order to activate the Conda environment using the previous configuration, run:

```
$ source env.sh
```

6. The first time this command is executed, it will download and install Orbkit (`https://orbkit.github.io/`) locally within the project folder.
7. If all the steps were executed successfully and the Conda environment is enabled, then the software is ready to use.

The source code is divided into two folders: “bin” and “src”.

- “bin” contains the scripts to be directly executed by the users from the command prompt. These scripts can be used to generate the datasets, train the AI models, and test them. Users who are only interested in executing the code as provided, should only use this folder. An example of executing one of these scripts can be seen in the following command:

```
$ python bin/dataset/generate_dataset.py QM9
```

- ”src” contains the different algorithms used by the scripts found in “bin”. Code from “src” should not be executed directly. Users who want to, for example, implement different AI algorithms, should use this folder.

Supplementary Subsection 1.2 Note about Tensorflow versions

Most of the development and testing was done using Tensorflow version 2.7. Based on our experience and the extensive testing done, all the source code used in this research should work with this version of Tensorflow. A Conda environment named “environmentTF7.yml” is supplied with the Git repository which matches our Tensorflow 2.7 Conda environment.

In later stages of the development we updated Tensorflow to version 2.10, and in our experience all the source code provided worked with this version too. A Conda environment named “environmentTF10.yml” is supplied with the Git repository which matches our Tensorflow 2.10 Conda environment.

We have also tested our source code with the last version of Tensorflow available at the moment of writing this report. This version was 2.13. This version did not work with some of our code. For example, it returned (at least) one error related with the Learning Rate Scheduler. Some of the scripts which did not use the Learning Rate Scheduler seemed to work fine, but we have not tested it. We have not tested our code with Tensorflow version 2.11 or 2.12.

It is our suggestion to stick with either Tensorflow 2.7 or Tensorflow 2.10. Since the version we tested the most was 2.7, that is the one we would recommened.

Supplementary Subsection 1.3 Generating the training dataset

This section will explain the different processing steps taken to prepare the dataset, which in the case of this research was the publicly available QM9 dataset (`http://`

quantum-machine.org/datasets/). All the steps described in this supplementary section were automated in the following single command. This command downloaded the dataset, generated the electron densities and electrostatic potentials for all the molecules present in the QM9 dataset, and encapsulated them into a TFRecord file (https://www.tensorflow.org/api_docs/python/tf/data/TFRecordDataset), ready to be inputted into the different ML algorithms:

```
$ python bin/dataset/generate_dataset.py QM9
```

Note of caution: This command downloaded and unpacked the QM9 Dataset (∼530 Mb), created an individual folder for each molecule containing their electron densities and electrostatic potentials (∼540Gb), and finally created two "TFRecord" files (train and test) of 236Gb and 27Gb. Therefore, it is recommended to have around 1Tb available space before running it. Depending on the CPU used, this command can take several hours. Once the command has been successfully executed, all the data except the "TFRecord" files can be deleted. We recommend to keep all the files in case this process needs to be repeated.

The rest of this supplementary section will explain all the different steps performed by this command.

Note: Originally our code only focused on SMILES, but based on the suggestion from one of the reviewers, we extended it to work with SELFIES too. Since the QM9 dataset already came with the SMILES representations, for every molecule an extra processing step was performed to transform its SMILES into its corresponding SELFIES. This was done using the "selfies" Python package (https://pypi.org/project/selfies/). The generated SELFIES were also stored in the TFRecord. This means that during training, no extra processing was required.

1.3.1 bin/dataset/generate_dataset.py

The previous command started the data generation process by executing the `generate_dataset.py` script. When executing this script, the name of the dataset to use must be given as an extra argument. In our case, this argument was "QM9". This script first executed the `get_dataset` function, which fetched the dataset into a *Dataset* variable named "dataset" and also configured the size and resolution of the tensors, which by default are set to (64, 64, 64) points separated by 0.5 Angstroms. It then executed the *Dataset* "generate" function, which will be explained in the next supplementary section. In the case of the QM9 dataset this function can be found in `/src/datasets/qm9.py`.

1.3.2 src/datasets/QM9.py generate()

This function is where most of the functionality to generate the dataset was centralised. It started by creating two folders (if they did not already exist): one where to save the downloaded data, and another where to save the data once it had been processed. It continued by downloading the QM9 dataset. The URL address from where the data was downloaded can be found in the variable `self.url`. It then extracted the contents of the downloaded file, which created a "xyz" file for each QM9 molecule (over 136,000 files). These "xyz" files contained different numerical properties related to each molecule. The most important properties for this research were the (x, y, z) coordinates for each atom, and the SMILES representation of the molecule.

The `generate()` function continued by executing another function within the same file named `_compute_electron_density`. When this function is invoked, it can take as argument "esp=True" in which case it will generate the electron density and electrostatic potential for each molecule. If this argument is set to "False", then it will only generate electron densities. Details of this function will be explained in the next section (Supplementary Section 1.3.3).

Once the electron densities and electrostatic potentials were generated, it created a list containing all the SMILES strings, and initialised a tokeniser based on this. This tokeniser was implemented as a table that related each SMILES token (such as a "C" for carbon, or "=" for double bond) with an identification number. Finally, it randomly split the data between train and test (90/10) datasets, and saved them into TFRecords. These TFRecords contained entries for each molecule present in the QM9 dataset, and each entry contained the data related to the molecule (electron density, electrostatic potentials, SMILES, and other data contained within the "xyz" file).

Once finished, it generated two files: "train.tfrecords" and "valid.tfrecords". These files were ready to be directly inputted into the different ML algorithms.

1.3.3 src/datasets/QM9.py _compute_electron_density()

This function iterated through all the extracted "xyz" files. For each of them, it invoked the function `_parse_single_qm9_file`.

This "parse" function started by reading the "xyz" file and storing in a Python dictionary the information it contained: number of atoms, (x,y,z) coordinates of each atom, SMILES representation, and different properties such as dipole moment, zero point vibrational energy, gap difference between LUMO and HOMO, ...

Focusing on the coordinates, it first arranged the molecule so that the geometric centre was at the beginning of the coordinate system. Then it used the "xtb tool" (`https://github.com/grimme-lab/xtb`) to generate a Molden file, and a sparse array containing electrostatic information (if required).

Given the Molden file, the corresponding electron density was calculated using the function `rho_compute` from Orbkit. This electron density was calculated for the cube defined before in the configuration section (which defaults to 64,64,64 and 0.5 Angstrom of step size).

Given the sparse array containing the electrostatic potentials, first an empty cube of size 64,64,64 was created filled with zeros, and then this cube was updated with the data points from the sparse array. Later on when this data will be inputted into a ML model, these points will be dilated to occupy a bigger space, see Supplementary Section 1.6, but at this stage of the data processing, this cube was mostly empty.

The dictionary created before was updated with information regarding the electrostatic potentials and electron densities of the current molecule. At the end of `_parse_single_qm9_file`, this dictionary was saved to disk.

The process describing how the electron densities and sparse electrostatic potentials were obtained can be seen in Supplementary Figure 1. Supplementary Figure 2, shows two examples of electrostatic potentials calculated using the method just described. In this figure, the molecule in the left is water, while the one in the right is a random molecule from the QM9 dataset.

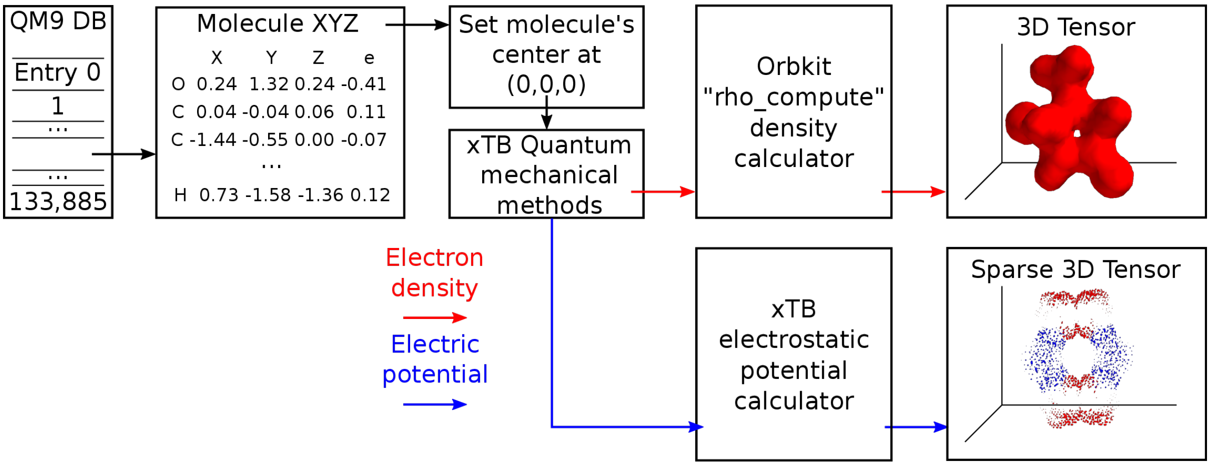

Supplementary Figure 1: From QM9 xyz descriptors to 3D electron densities and sparse electrostatic potentials. The data from the QM9 dataset was transformed into electron densities using "xTB" quantum mechanical methods, followed by the function "rho_computer" from Orbkit. To obtain the electrostatic potentials "xTB" electrostaic potential calculator was used to obtain sparse representations.

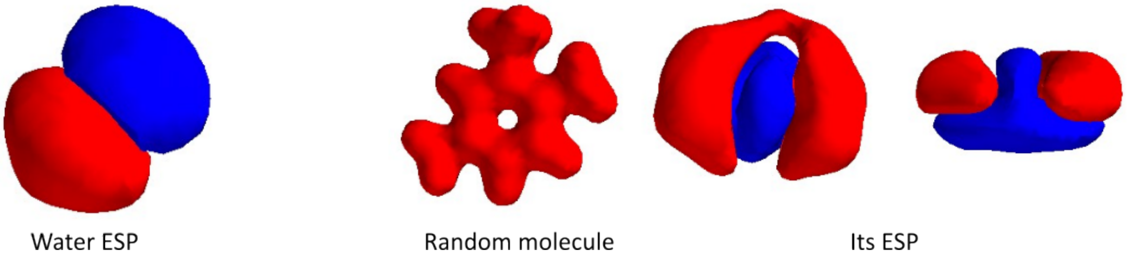

Supplementary Figure 2: Examples of electrostatic potentials calculated using xTB electrostatic potential calculator. Left: the electrostatic potential of a water molecule calculated with the explained method. Right: the electrostatic potential of a random QM9 molecule calculated with the explained method. In this figure the term "ESP" was used instead of "electrostatic potential".

Supplementary Subsection 1.4 src/models/layers.py

Since the different machine learning implementations shared some degree of functionality, it was decided to centralise the shared functionality in the file `src/models/layers.py`. In this file it can be found definitions for residual layers, attention layers, transformer blocks, and layers that either added or removed dimensionality to the tensors.

1.4.1 Residual connections

Our implementation was based on Keras' Resnet50 (`https://github.com/keras-team/keras-applications/blob/master/keras_applications/resnet50.py`). We also defined "identity" or "conv" blocks depending on if the input tensor was directly shortcutted or if it went through a convolution. Since our experiments used Tensors of different dimensions, the code was adapted to automatically work with tensors of four, three or two dimensions, using 3D, 2D or 1D convolutions. By default "relu" activation functions was used, although "leakyrelu" was also available.

1.4.2 Add and drop dimension layers

The "add dimension" layer used Tensorflow's `expand_dims` function, adding a new dimension at the end (-1). After the dimension was added, a convolution of the required dimensionality was performed.

The "drop dimension" layer performed a convolution of the required dimensionality with the number of filters set to 1. After this convolution, Tensorflow's `squeeze` function was used to remove the dimension with only 1 value.

1.4.3 Attention layer

Our attention layer implementation is adapted from SAGAN [1], but using 3D or 2D convolutions depending on the input tensor. In particular, we based our implementation in the following project:

`https://github.com/taki0112/Self-Attention-GAN-Tensorflow/`.

1.4.4 Transformer block

Our transformer block implementation was adapted from:

`https://keras.io/examples/nlp/text_classification_with_transformer/`

But using attention layers as defined above, convolution layers instead of dense layers, and batch normalisation instead of layer normalisation.

Supplementary Subsection 1.5 Using a Variational autoencoder (VAE) to model the QM9 dataset

A Variational Autoencoder (VAE) was used to model the chemical space defined by the electron densities as derived from the QM9. This VAE took as input the 3D tensors representing the electron densities, followed by several layers of 3D convolutions to compress the data into a 1D latent vector (Supplementary Figure 3), and then used mirrored layers of transposed convolutions to reconstruct the input data (Supplementary Figure 4). Therefore, the objective of the VAE was to learn a 1D latent space where the data

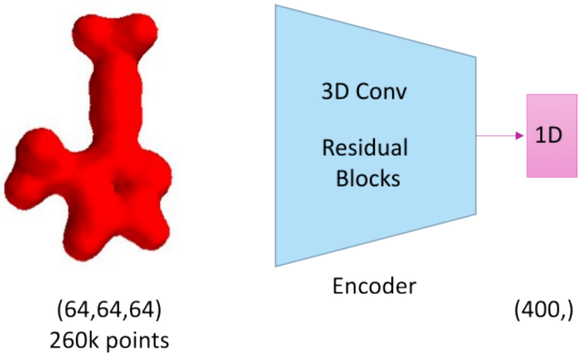

Supplementary Figure 3: Using a VAE to encode a 3D electron density with 260,000 points into a 1D vector with 400 points. The VAE's encoder took as input high dimensionality data and compressed it into a 1D latent vector with 400 positions (in this example).

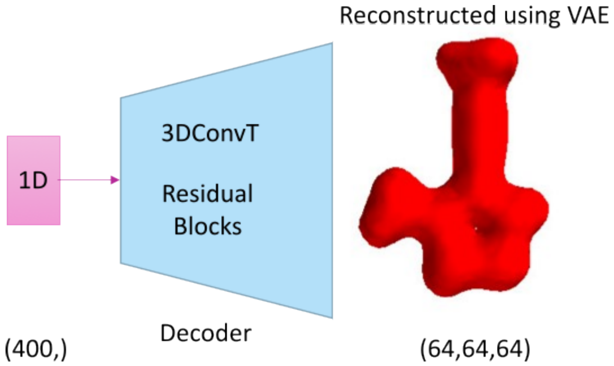

Supplementary Figure 4: Using a VAE to decode a 1D vector into a 3D electron density. The VAE's decoder took as input a 1D vector with 400 positions (in this example), and reconstructed it into a higher dimensionality tensor (a 3D tensor of 64,64,64 units) .

could be compressed and de-compressed losing the minimum amount of information. See Supplementary Figure 5 for an outline of this process.

The base VAE can be found in `src/models/VAE.py`, see Supplementary Section 1.5.1 for more information. An extended VAE using residual connections can be found in `src/models/VAEresnet.py`, see Supplementary Section 1.5.2 for more information. An extended VAE using attention mechanisms can be found in `src/models/VAEattention.py`, see Supplementary Section 1.5.3 for more information.

To train these models, the script `bin/train/train_vae.py` should be used, see 1.5.4 for more information.

1.5.1 src/models/VAE.py

This script contained the base VAE implementation. The extended implementations mentioned before built upon it. Our VAE implementation followed the one described in the book "Generative Deep Learning" by David Foster. Our code was heavily based on theirs: `https://github.com/davidADSP/GDL_code/blob/tensorflow_2/models/VAE.py`, but using 3D convolutions instead. As it is common with VAEs, our loss functions had two components: a reconstruction loss which compared input and output 3D volumes

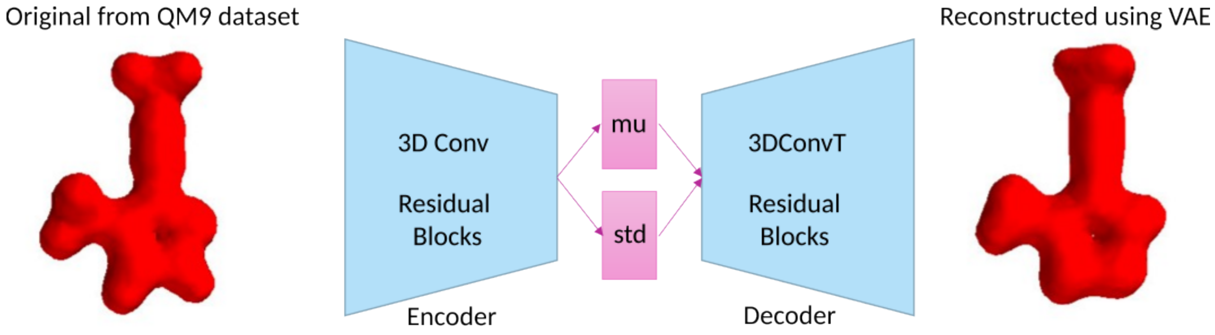

Supplementary Figure 5: Using a Variational Autoencoder to model the QM9 chemical space. The VAE model took as input the electron density from a QM9 molecule, and aimed to reconstruct it. In doing so, it learned to compress 3D electron densities into 1D latent vectors.

using mean squared error, and the Kullback-Leibler divergence used to measure how much the VAE's latent distribution diverted from the standard normal distribution.

The main changes related to how the data was pre-processed and post-processed. Focusing on the data pre-processing, the first step required to add an extra dimension, because all the electron density tensors were 3D, but to perform 3D convolutions the data must be 4D. The next step applied a "tanh" function to each data point in each tensor to guarantee that all the data points were between -1 and +1. Finally, a value of "1e-4" was added to each point, followed by applying a log operation, followed by dividing the result by "1e-4". After these steps, the data was inputted into the VAE. Once the data came out of the VAE, the reverse operations were executed minus the tanh operation: First the outputs (every point) was multiplied by "1e-4", then an exp operation was executed, and finally "1e-4" is subtracted.

The architecture of this VAE can be seen in Supplementary Figure 8, but using normal convolutions instead of residual connections.

Some other extra functionality that can be found in this file:

- The function `sample_model_validation`. This function was used with a callback to sample the model after every epoch. It took a batch from the training data, inputted it into the VAE, and saved to disk the reconstructed output.

- The function `interpolation_two_molecules`. This function received as input two molecules and a number of steps, and it performed a linear interpolation as it can be seen in the main manuscript, and in Supplementary Figures 6 and 7. First, it used the VAE's molecule encoder to obtain the 1D latent representations of the two input molecules. Then it did a N-step interpolation between the two latent representations, and for each step it used the VAE decoder to reconstruct its 3D volume and saved it to disk.

- The function `substract_add_molecules`. This function received as input two batches of data. It obtained the latent 1D representation for each molecule in the batches, and then added and subtracted the batches (producing two different outputs), finally it reconstructed the 3D representations and saved them to disk.

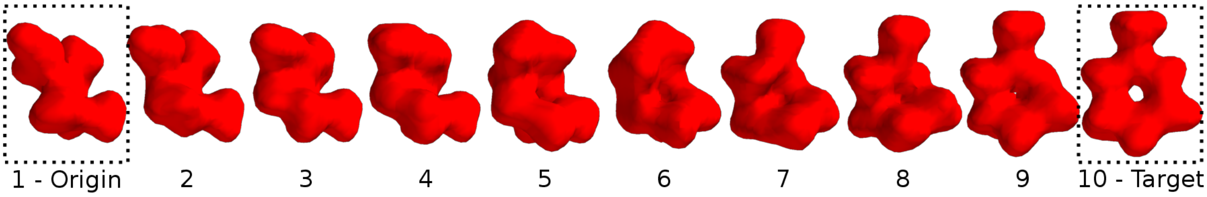

Supplementary Figure 6: Using the VAE latent space to interpolate between two molecules. The electron densities from two different molecules (origin and target) were encoded into their respective 1D latent vectors. A linear interpolation was executed from Origin to Target through the latent vectors. Each latent vector calculated as part of this interpolation, was then reconstructed into its related 3D electron density.

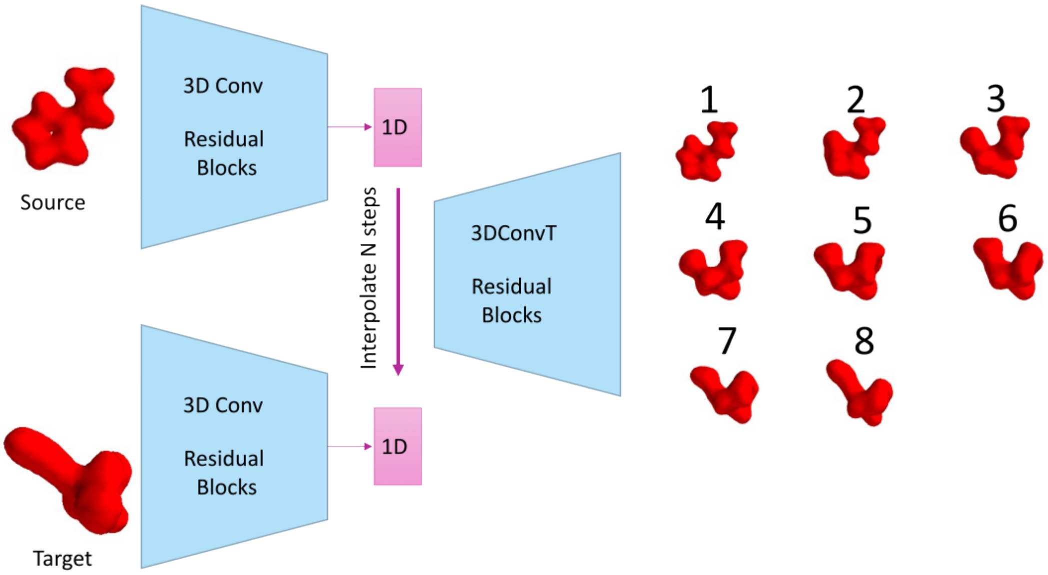

Supplementary Figure 7: Using the VAE latent space to interpolate between two molecules. This figure shows in more detail the calculations performed to create the previous figure. First, a source and a target molecule are chosen. Then, using the VAE encoder, a 1D latent vector representation is obtained for each of these mocules. Then, a linear interpolation is done between these two latent vectors, and each middle step is decoded into a 3D electron density using the VAE decoder.

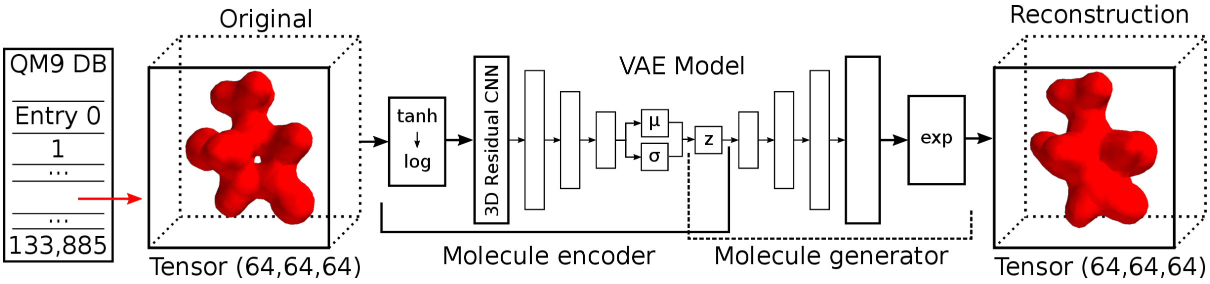

Supplementary Figure 8: VAE using residual connections used to model the QM9 dataset. Both encoder and decoder used residual connections. Before being inputted into the encoder, the 3D electron density was passed through a tanh and a log operation. At the other end, before being reconstructed, the generated 3D volume was passed through a exp operation..

1.5.2 src/models/VAEresnet.py

This VAE used residual connections. It subclassed the base VAE described in Supplementary Section 1.5.1, but replacing Tensorflow's stock convolutional layers with the residual layers described in Supplementary Section 1.4.1. While the base VAE upsampled the data in the decoder using transpose convolutions; here the decoder used residual convolutions, followed by an upsampling layer. This is the VAE that offered the best results, and the one used in the research described in the main manuscript. See Supplementary Figure 8 for an outline of its architecture.

To showcase its results, Supplementary Figure 9 shows five electron densities as generated by this VAE. The left column displays the electron densities inputted to the VAE, which were generated from the QM9 dataset as explained in Supplementary Section 1.3. The right column displays the previous molecules after going through the VAE.

1.5.3 src/models/VAEattention.py

This VAE used both residual connections (as the VAE described in Supplementary Section 1.5.2) and transformer blocks as detailed in Supplementary Section 1.4.4. To find the best VAE architecture, different strategies were tested, with different configurations of convolutional layers, residual connections and transformer blocks. For example, using as reference the VAE shown in Supplementary Figure 8, we tried adding attention mechanisms both after the encoder and before the generator (therefore mirrored blocks), or only at the end of the generator, or after every convolution, ... among many other configurations. But none of them improved the accuracy of the VAE with only residual connections, while the computational cost increased. Therefore, even though based on the literature it was expected that this implementation would produce the best results, it was instead decided to stick with the VAE with only residual connections.

1.5.4 bin/train/train_vae.py

While the previous VAE supplementary sections explained how the ML models were implemented, this supplementary section will describe the script used to train them. We will focus on describing the parameters that need to be set-up. These parameters will be

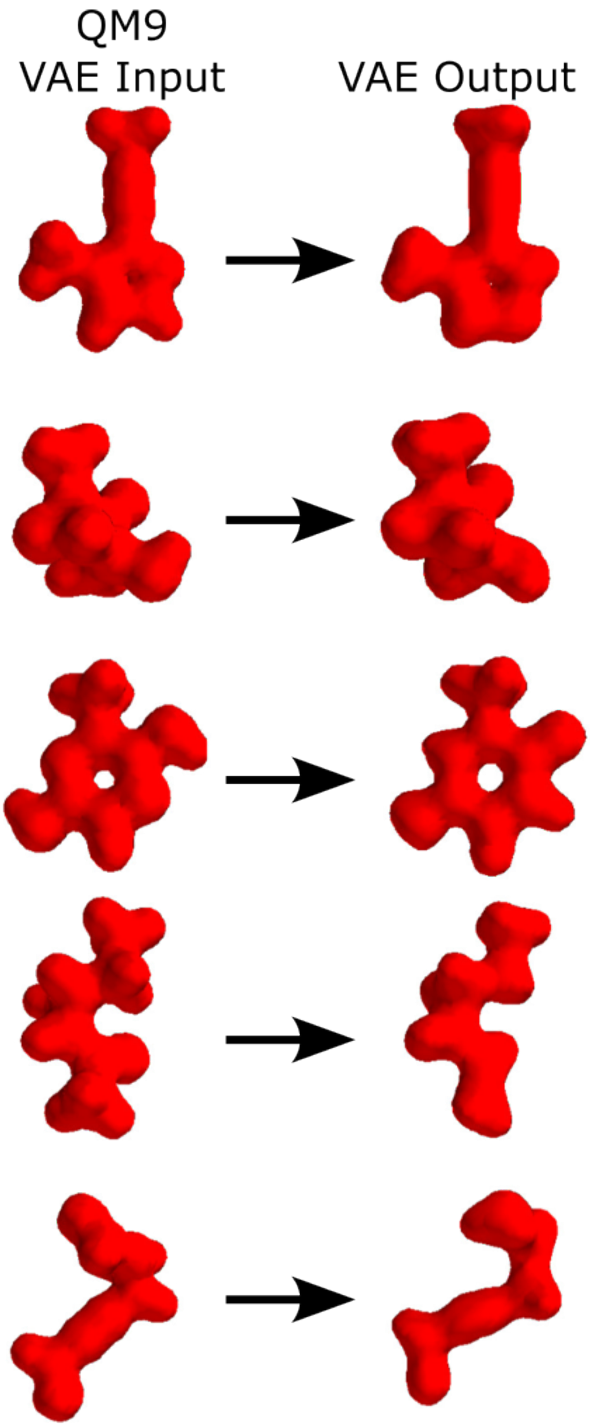

Supplementary Figure 9: Examples of reconstructed Electron Densities using the VAE. Left: Electron densities as obtained from the QM9 dataset. Right: The reconstructed electron densities as generated by the VAE. The QM9 molecules in the left column were inputted into the VAE, and it generated the molecules shown in the right column.

divided into three blocks: General configuration, VAE model architecture, and training configuration.

— General configuration:

- Line 19: Here the user can decide the GPUs to use. If only one GPU is present in the system, this value can be set to '0'. Otherwise, the user can specify the GPUs to use as a list.
- "RUN_FOLDER" (line 20). Folder where all the models and generated data will be saved. This folder must exist before the script is executed.
- "mode" (line 21). Mode can be set to either 'build' or 'load'. 'build' should be used to initialise and start the first training run, while 'load' can be used to continue a previous training run. If 'build' is chosen, a folder with the current date will be created inside RUN_FOLDER, where all the data will be saved. If 'load' is chosen, the user must specify the folder from where to fetch the data and continue the training in line 33. This folder must contain a file named "weights.h5". This file is auto-generated while training. If another file with weights is used to load and continue training, line 76 must be modified.
- "DATA_FOLDER" (line 35). This folder must contain the TFRecord files generated when the training data was created, see Supplementary Section 1.3. If the name of the TFRecord files is not left as generated by default ('train' and 'valid'), the new names must be changed in the "path2tf" and "path2va" variables (lines 40 and 41).
- Training and testing batch sizes. The batch sizes can be set in lines 43 and 44, where the class TFRecordLoader is used to load the training sets.

— VAE model architecture:

- Type of VAE. The type of VAE can be chosen in line 53. The names used can be "VAE", "VAEresnet" or "VAEattention". These three names relate to the VAE models described before, and they are the name of the Python classes that can be used to create the "vae" Python object. The following parameters (except the last one, learning rate) will define the attributes used to create the VAE class.
- "encoder_conv_filter". This parameter will be a list describing the convolutional filters used for each layer within the encoder. For example, if this parameter is set to "[16, 32, 64, 128]", the encoder will have four layers (the length of the list defines the number of layers), and the first layer will use convolutions with 16 filters, the second one with 32 filters, the third one with 64 filters, and the last one with 128 filters.
- "encoder_conv_kernel_size". This parameter will be a list like before, where each element in the list will define the kernel size of the convolutions within their respective layers. For example, if this parameter is set to "[3, 3, 3, 5]", the encoder will have four layers, where the first three layers will use kernel of size 3, while the last kernel will use kernels of size 5. The length of this list must be exactly the same as the previous list.
- "encoder_conv_strides". This parameter will be a list like before describing the strides taken while doing the convolutions. The length of this list must be exactly the same as the previous list.

- The following three parameters are the same as the three just explained, but their names start with “dec_”. These parameters define the same functionality as the previous ones, but in the decoder. The decoder will use transpose convolutions instead of normal convolutions. The length of their lists must be the same as before. Usually in VAEs the decoder mirrors the encoder. This is not mandatory in this implementation, but the shape of the output data must be equal to the shape of the input data.

- “z_dim”. The output of the last enconder’s layer will be flattened into a 1D array of size “z_dim”.

- “use_batch_norm”. This parameters can be set to True or False, and it is only enabled when using the base VAE. If True, a Batch Normalisation layer will be added after every convolution. If using “VAEresnet” or “VAEattention”, Batch Normalisation will be always used after the convolutions, following Keras’ ResNet50 implementation.

- “use_dropout”. This parameters can be set to True or False, and it is only enabled when using the base VAE. If True, dropout regularisation will be applied after the convolution layers (and after Batch Normalisation if present). Dropout normalisation is not used if using “VAEresnet” or “VAEattention”.

- “r_loss_factor”. This parameter relates to the VAE loss function, and represents a factor by which the reconstruction loss is multiplied by, before being added to the Kullback–Leibler loss.

- “LEARNING_RATE”. This parameter is not part of the VAE constructor, but it is part of the model architecture, since Tensorflow requires to know the learning rate before compiling the model. This parameter relates to the learning rate factor used when training the models.

— Training configuration:

- “EPOCHS” (line 79). Total number of epochs to iterate trough the training data.

- “INITIAL_EPOCH (line 80). If using “mode = `"load"`”, this parameters indicates the epoch from where to continue the training. In “mode = `"build"`” this parameter is ignored.

- “EPOCHS_PRINT” (line 81). The callback described in Supplementary Section 1.5.1 that samples the model will be executed every “EPOCHS_PRINT” epochs.

Once the parameters have been properly configured, the user started (or continued) training the model with:

```
$ python bin/train/train_vae.py
```

For this command to work properly the “electrondensity” Conda environment must be enabled (see Supplementary Section 1.1).

When executed in “build” mode, this script created a new folder inside `logs/vae` named with the current date, for example `logs/vae/2022-06-09`. Inside the folder named with the current date (for example `logs/vae/2022-06-09`), there will be a `params.pkl` file and two folders named `edms` and `weights`:

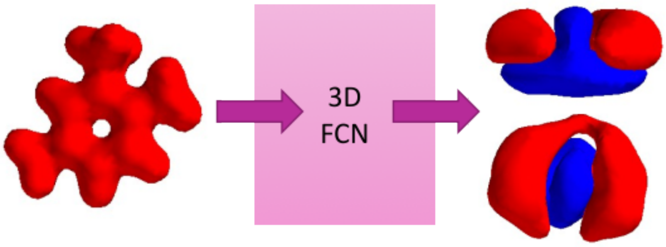

Supplementary Figure 10: Example of an electrostatic potential calculated from an electron density, using a trained Fully Convolutional Network (FCN). The objective of the FCN was to calculate electrostatic potentials from electron densities. This Supplementary Figures shows on the left an example of an input molecules obtained from the QM9 dataset, and on the right its electrostatic potentials as calculated by the FCN.

- `params.pkl` is a Python Pickle file containing the parameters used to create the model.
- `edms` contains a number of Pickle files created each time the callback function was used to sample the model. These Pickle files contained 3D tensors representing electron densities generated using the validation set.
- `weights` contains a list of "h5" files. Each of these files contained the weights of the model saved after a particular epoch. These are the files that later on can be loaded into the model to either keep training (when executed in load mode) or to test new data.

Supplementary Subsection 1.6 Using a Fully Convolutional Network (FCN) to convert electron densities to electrostatic potentials

A Fully Convolutional Network (FCN) was used to calculate the electrostatic potentials from a molecule given its electron densities. This FCN took as input 3D tensors representing the electron densities, used several layers of 3D convolutions, and then used mirrored layers of transposed convolutions to generate the related 3D tensor representing the electrostatic potential, see Supplementary Figure 10. Therefore, the objective of the FCN was to learn how to calculate 3D electrostatic potentials given 3D electron densities.

The FCN sub-classed the VAE class (see Supplementary Section 1.5.1), and used residual connections in a similar way to the VAE described in Supplementary Section 1.5.2. The FCN source code can be found in `src/models/ED2ESP.py`, see Supplementary Section 1.6.1 for more information. To train this model, the script found in `bin/train/train_ed2esp.py` should be used, see 1.6.2 for more information.

1.6.1 src/models/ED2ESP.py

This file contained the source code that implemented the FCN. It sub-classed the base VAE to get some of the boilerplate functionality, but the model itself was implemented

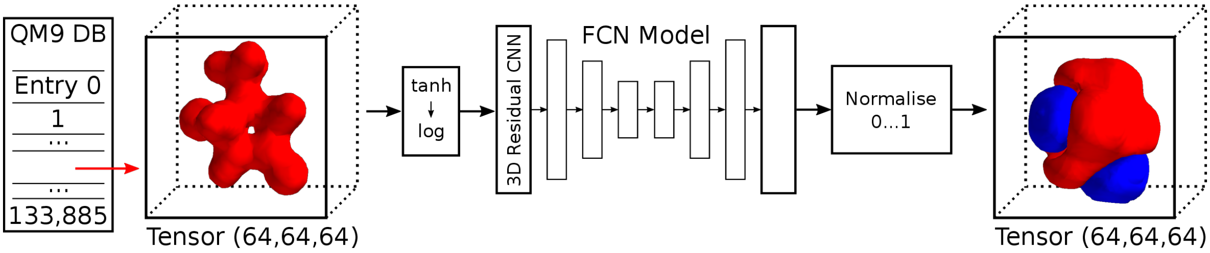

Supplementary Figure 11: Fully Convolutional Network (FCN) used to calculate electrostatic potentials from electron densities. The QM9 dataset was used to train the FCN. First, for all the QM9 molecules, their electrostatic potentials were calculated using xTB (as previously explained). Then the QM9 molecule (its electron density) was inputted into the FCN, and this outputted an electrostatic potential. This outputted electrostatic potential was compared with the one generated using xTB, and the FCN was trained to generate electrostatic potentials as similar as possible to the ones generated using xTB.

from scratch.

The FCN was implemented as a simpler VAE: it had two mirrored networks similar to the VAE's encoder and decoder, but instead of flattening the last layer of the encoder into a 1D latent vector, in the FCN the encoder was directly connected to the decoder, see Supplementary Figure 11. Similarly to the VAE implementation described in Supplementary Section 1.5.2, the FCN was implemented using residual connections, since this showed to improve the results. Upsampling in the decoder was also achieved using an "Upsampling" layer after performing a convolution. Another major difference was the loss function used. In our implementation the FCN also used a simplified version of the VAE's loss function only using the reconstruction loss, and ignoring the KL terms.

The other main difference relates to the way the training data was processed. The FCN training data contained for each molecule pairs of electron densities, which were inputted into the network, and electrostatic potentials, which were compared against the network outputs. The input 3D tensors containing electron densities were processed in the same way as before (see Supplementary Section 1.5.1). The output 3D electrostatic potentials were what our model was compared against, and the training was based on the difference between what the network outputted, and what the dataset contained (the reconstruction loss). As explained in the "Generating the training data" section (Supplementary Section 1.3.3), the tensors in the dataset representing the electrostatic potentials were very sparse: they did not contain continuous data, but single points. Thus, the pre-processing step in this file transformed the electrostatic potentials from single points to continuos 3D volumes. To achieve this, the single points were dilated. In our implementation, each point was dilated into a 3D neighbourhood of size 5. The data points were also scaled between 0 and 1. See Supplementary Figure 12 to visualise the processing pipeline, and Supplementary Figure 13 to see examples of generated electrostatic potentials using the described method.

1.6.2 bin/train/train_ed2esp.py

The script `bin/train/train_ed2esp.py` should be used to train the FCN. Before invoking it, its contents should be modified to match the required configuration. Since this file is very similar to `bin/train/train_vae.py` (Supplementary Section 1.5.4), in this

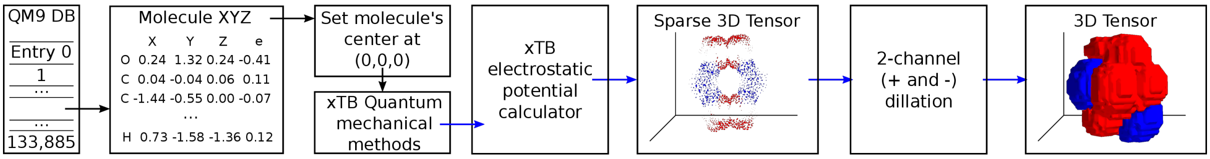

Supplementary Figure 12: Diagram showing how the electrostatic potentials were obtained from the QM9 dataset, and processed to be inputted into the FCN. Using the XYZ data from the QM9 dataset, first a sparse representation was obtained using xTB, and then this sparse representaion was converted into a volume using 2-channel dillations.

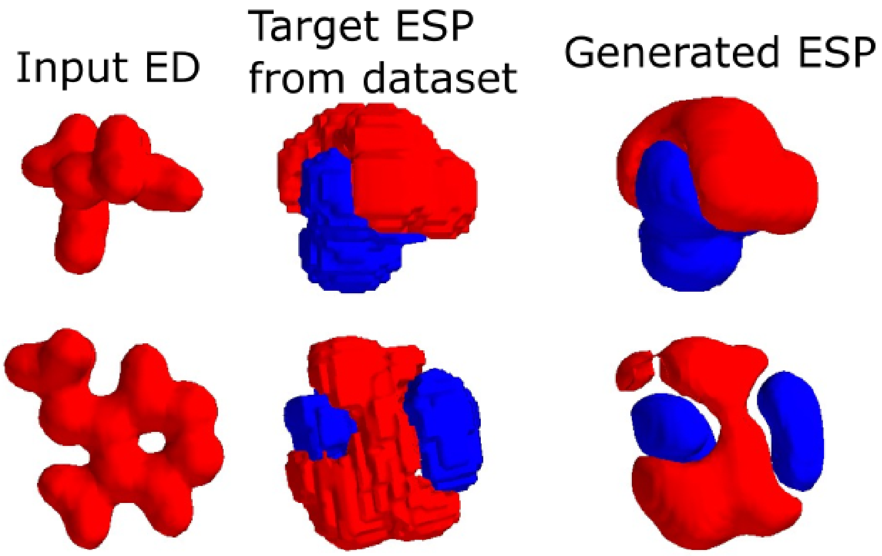

Supplementary Figure 13: Examples of electrostatic potentials generated with the described FCN. This figure shows, in the left column, electron densities as fetched from the QM9 dataset, in the middle column electrostatic potentials as calculated using quantum methods, and on the right column, electrostatic potentials as generated through the described neural network, which used as input the electron densities shown in the left column. In this figure, “ED” is a shorthand for electron density, and “ESP” is a shorthand for electrostatic potential.

supplementary section we will only focus on the differences.

- The general configuration parameters were exactly the same as before with the exception of how the `TFRecordLoader` object was created. While before when creating this object we only specified the batch size as a parameter, in this script we also needed to specify as a property that the data must contain electrostatic potentials (lines 44 and 45), because by default only electron densities were loaded. These changes have already been applied.
- The network architecture was the same as before. The main difference is that the network was created using the class `VAE_ed_esp`. When creating the network, the parameters `z_dim`, `use_batch_norm`, `use_dropout` and `r_loss_factor` were ignored. These parameters were needed in the constructor because the code subclassed from the base VAE, but the FCN did not use them. The rest of the parameters can be freely modified to define the network architecture.
- The training configuration parameters were the same as before.

The user should start or continue training the model with the following command:

```
$ python bin/train/train_ed2esp.py
```

For this command to work properly, the “electrondensity” Conda environment must be enabled (see Supplementary Section 1.1).

When executed in “build” mode, this script created a new folder inside `logs/vae_ed_esp` named with the current date, for example `logs/vae_ed_esp/2022-06-09`. Inside the folder named with the current data (for example `logs/vae_ed_esp/2022-06-09`), there will be a `params.pkl` file and two folders named `edms` and `weights`:

- `params.pkl` is a Python Pickle file containing the parameters used to create the model.
- `edms` contains a number of Pickle files created each time the callback function was used to sample the model. These Pickle files contained 3D tensors representing electrostatic potentials generated using the validation set.
- `weights` contains a list of “h5” files. Each of these files contained the weights of the model saved after a particular epoch. These are the files that later on can be loaded into the model to either keep training (when executed in load mode) or to test new data.

Supplementary Subsection 1.7 Predicting numerical properties from electron densities

This supplementary section discusses the model used to predict numerical properties from electron densities. Both the electron densities and the different numerical properties were obtained from the QM9 dataset. The model can be found in `src/models/CNN3D_featureprediction.py`, see Supplementary Section 1.7.1 for more information. To train this model, the script found in `bin/train/train_property_prediction.py` should be used, see 1.7.2 for more information.

Although the code to perform these predictions is included as part of the project repository, none of the results shown in the main manuscript were obtained using this network.

1.7.1 src/models/CNN3D_featureprediction.py

This script contains a ML model that predicted a numerical (float) value from input multidimensional data. In our case, the input multidimensional data were 3D tensors representing electron densities, while the numerical properties were any of the values contained in the QM9 dataset, such as dipole moment, or isotropic polarisability, among many others. The full list of numerical properties can be seen in the file `src/datasets/qm9.py`, within the function `read_qm9_file()` (lines 32 to 50). To perform the numerical prediction, the model took as input 3D tensors, processed them through a series of convolutional layers, and then flattened and inputted them into a single output neuron which outputted a float value. The loss function used was "mean squared error".

1.7.2 bin/train/train_property_prediction.py

To train this network, the user should use the script `bin/train/train_property_prediction.py`. Before invoking it, its contents should be modified to match the required configuration. Since this file is very similar to `bin/train/train_vae.py` (Supplementary Section 1.5.4), in this supplementary section we will only focus on the differences.

- The general configuration parameters were exactly the same as before with the exception of how the `TFRecordLoader` object was created. While before when creating this object we only specified the batch size as a parameter, in this script we also needed to specify as a property the numerical properties to load from the QM9 dataset (lines 43 and 44). The full list of numerical properties can be seen in the file `src/datasets/qm9.py`, within the function `read_qm9_file` (lines 32 to 50).
- The architecture configuration contained four parameters, although the first one ("cubeside") can be ignored. Both "filters" and "strides" must be lists of equal length describing the number of filters per layer, and the strides that the convolutions will take. "dense_size" is the number of neurons in the dense flattened layer after the last layer of convolutions.
- The training configuration parameters are the same as before.

The user should start or continue training a model with the following command:

```
$ python bin/train/train_property_prediction.py
```

For this command to work properly the "electrondensity" Conda environment must be enabled (see Supplementary Section 1.1).

Supplementary Subsection 1.8 Decorating electron densities with electrostatic potentials

In the previous supplementary sections, the inputs to all the ML models were electron densities as obtained from the QM9 dataset. Supplementary Section 1.3 detailed how the training data was obtained, while Supplementary Section 1.5.1 detailed how the data was preprocessed before being inputted into the network. In Supplementary Section 1.3.3 it was also detailed how to calculate the electrostatic potentials for every molecule using

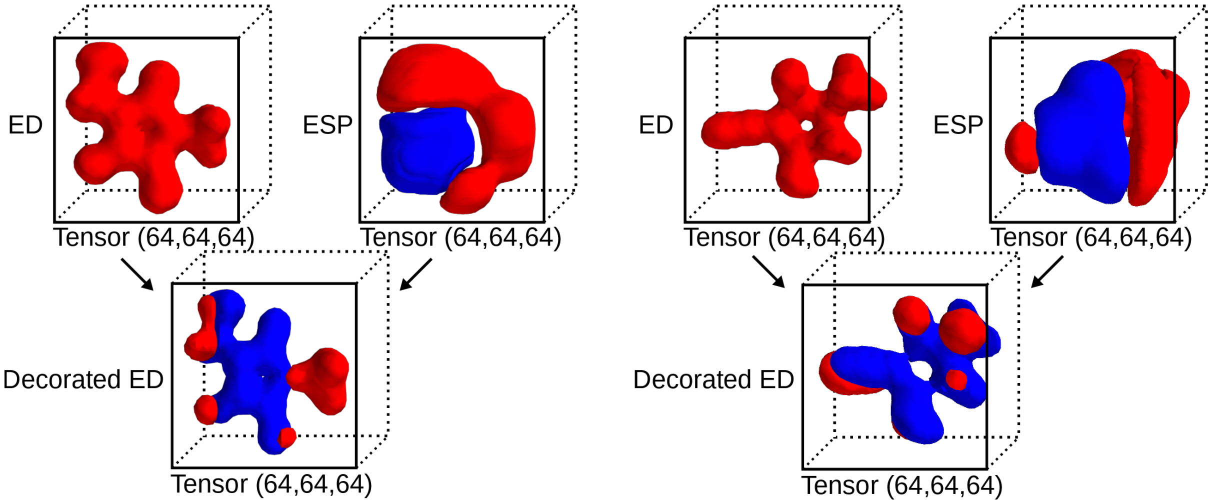

Supplementary Figure 14: Process showing the result of decorating electron densities with electrostatic potentials. The decorated electron densities are achived are achieved my multiplying the original electron densities with the original electrostatic potentials. In this figure, “ED” means Electron density, and “ESP” means Electrostatic potentials.

the XYZ information provided by the QM9 dataset. In this supplementary section it will be explained how electron densities and electrostatic potentials were combined into a new data structure which from now on will be referred as decorated electron densities. In our experience, decorated electron densities slightly improved the results over non-decorated electron densities.

To decorate the electron densities the following steps were performed:

1. Both electron densities and electrostatic potentials were obtained from the QM9 dataset using quantum methods as explained in Supplementary Section 1.3.3.
2. The electrostatic potentials were preprocessed in a similar way to how it was described in Supplementary Section 1.6.1 but using dilations with a 3D neighbourhood of 10 units.
3. The dilated electrostatic potentials were multiplied with the electron densities.
4. A “tanh” operation was applied.
5. A value of “1e-4” was added to each point, followed by applying a log operation, followed by dividing the result by “1e-4”.
6. A “tanh” operation was applied.
7. The data was re-scaled between 0 and 1.
8. The data was now inputted into the different models.

Supplementary Figure 14 shows two different decorated electron densities.

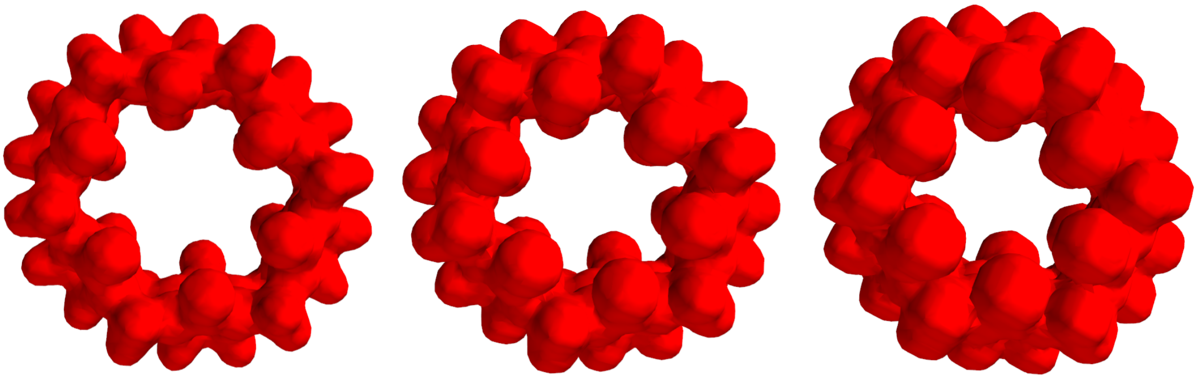

Supplementary Figure 15: Decreasing the size of the host's cavity. The cavity of the host was decreased by applying a 3D max pool operation with different kernel sizes (in this image, kernels of size 1, 2 and 3).

Supplementary Subsection 1.9 Decreasing the size (volume) of the cavity

It is reported in the literature that given a host, the best guests will occupy around a 55% of the host's cavity [2]. Therefore, given a host, an expert chemist would focus on studying guests that occupy a 55% of the host's volume. To achieve a similar functionality in our optimisation algorithms, we decreased the size of the cavity before testing the different guests: given a host's electron density calculated as described in Supplementary Section 1.3.3, a post-processing step was performed to decrease the size of the cavity.

To do this, we applied a "max_pool3d" (as provided by Tensorflow) over the 3D cube defining the electron density. When using this operation, if a kernel of size 1 was used then there were no changes, but if kernels of a bigger size were used, the electron density dilated, decreasing the size of the cavity, see Supplementary Figure 15. Unless otherwise specified, all the results shown were obtained using the stock electron density, without decreasing the size of the cavity.

Supplementary Subsection 1.10 Using the GPT model to generate SMILES sequences

The Generative Pre-trained Transformer (GPT) architecture is an autoregressive language model that uses stacked transformer blocks with attention mechanisms (see Supplementary Section 1.4.4) to generate novel data from a given seed [3]. It is commonly used to generate human-like text. For example, given an input like "I am writing", the GPT model would generate something like "the Supplementary Information". In this research, our GPT model received as input the start of a SMILES sequence, and generated as output the rest of the SMILES sequence to complete the molecule. For example, given the SMILES string "O" as input (just one oxygen atom), our GPT SMILES model would generate an output string such as "O=C=O" (CO_2) (among many other molecules whose SMILES sequence starts with an oxygen). See Supplementary Figure 16 for an outline of this process.

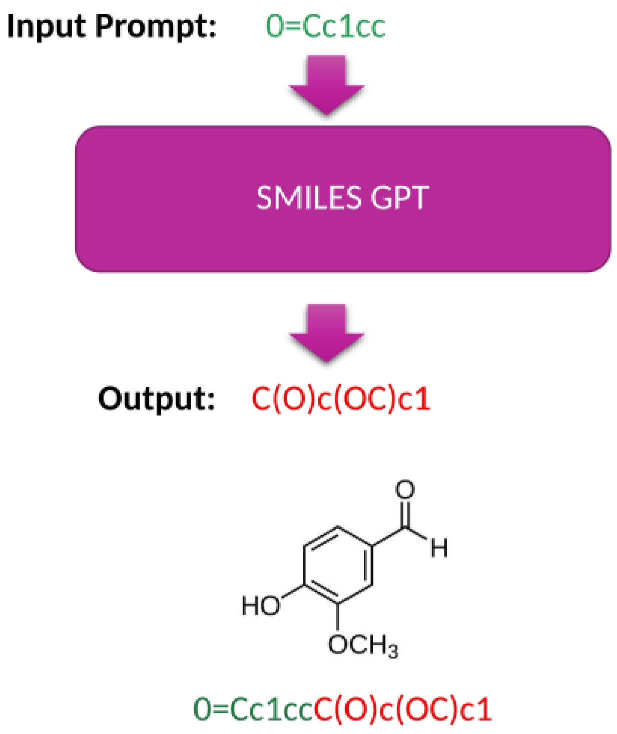

Supplementary Figure 16: The SMILES GPT model received as input a SMILES string representing the start of a molecule, and outputted a completed molecule in SMILES representation. The 2D drawing of the molecules displayed in this image was obtained using RDKit, and it was not generated by the SMILES GPT model. This model only worked with SMILES data.

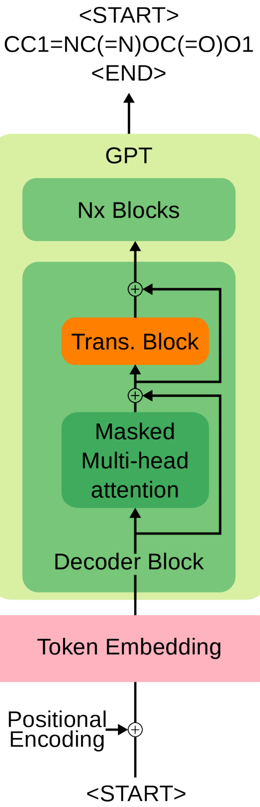

Supplementary Figure 17: SMILES GPT model architecture. This architecture is very similar to the decoder part of a standard Transformer model. The input to this model are sequences of SMILES tokens, plus the special token <START> to signal the start of a molecule. It then follows a number of Attention layers. The model outputs the special token <START>, followed by the SMILES tokens, and finishing with the special token <END>.

1.10.1 src/models/gpt.py

Our SMILES GPT architecture was heavily based on the following Keras example:
`https://keras.io/examples/generative/text_generation_with_miniature_gpt/`

The main architectural difference is that while this example used only one Transformer block, our implementation allowed for a variable number of stacked Transformer blocks (see Supplementary Figure 17). Another difference in our implementation, is that while this example used the stock Keras' training step with "SparseCategoricalCrossentropy" as loss function, we implemented our own training (and test) step following the following Keras example, which allowed for a finer control:
`https://keras.io/examples/audio/transformer_asr/`

The callback/function to generate new text was also slightly different, since in our case we generated SMILES sequences token by token, which was simpler than generating full English sentences. This function took as seed the "<START>" token or a SMILES sequence without the "<END>" token. This seed was then inputted into the model, which generated a new token. This new token was appended into the previous sequence, and inputted again into the model. The process was repeated until the model outputted an

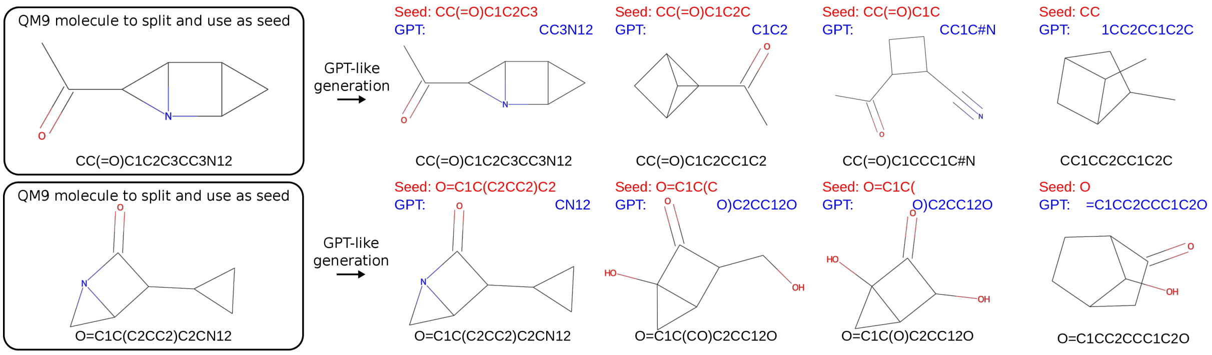

Supplementary Figure 18: Examples of SMILES sequences generated using the described architecture. Using the SMILES representation of molecules on the left as starting point, SMILES tokens were removed from the right of the representation, and the GPT model was asked to generate the rest of the molecule using that incomplete SMILES representation as seed. The 2D diagrams of the molecules were drawn from the SMILES sequences using RDKit.

"<END>" token. Supplementary Figures 18 and 19 shows examples of SMILES sequences generated using this process.

1.10.2 Generating new SMILES sequences: greedy vs probabilistic sampling

The function used to generate new tokens operated in two different modes: greedy or using probabilistic sampling. In greedy mode, when generating a new SMILES token, the model chose the one with the highest score (as outputted by the model). In probabilistic sampling, first the user needed to specify a window "n" (for example five). Then, once the model generated an output, the top "n" tokens were saved based on their scores, and then one of them was chosen randomly but weighted by their respective scores. Thus, tokens with highest scores were more likely to be chosen, but tokens with low scores could also be chosen. This type of selection is also known as "roulette algorithm".

1.10.3 bin/train/train_gpt.py

The script used to train the model is very similar to the training scripts discussed before (see Supplementary Sections 1.5.4 and 1.6.2). The general and training configuration sections are the same as before, with the only difference being that the user had to make sure that the SMILES data was loaded (see lines 33 and 34). The main differences in this file are in the section where the architecture was defined. In this section, a Python object using the GPT class was created, and the following four parameters need to be defined:

- "embed_dim": The dimension of the embedding layer.
- "num_heads": Number of heads used in the multi-head attention blocks.
- "feed_forward_dim": Number of neurons that the feed-forward dense layers contained.
- "num_trans_blocks": Number of stacked Transformer blocks.

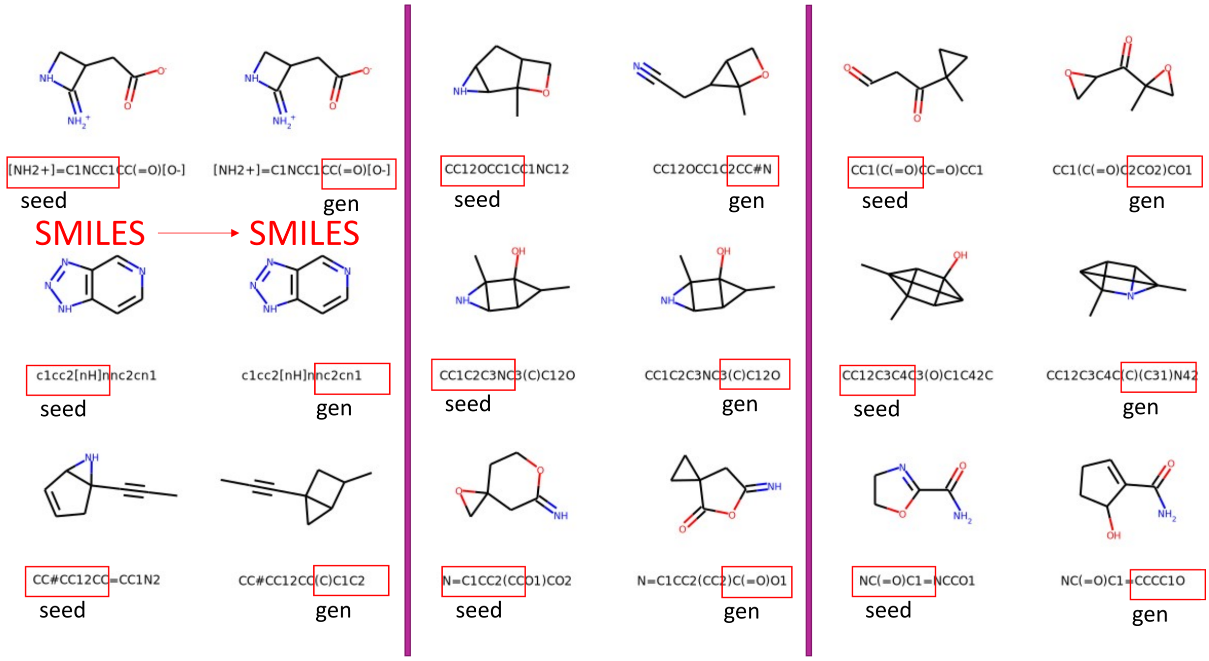

Supplementary Figure 19: Examples of SMILES sequences generated the described architecture. Columns one, three and five display SMILES sequences fetched from the QM9 dataset, and their related 2D diagram generated using RDKit. The 10 first tokens from these sequences were inputted into the described GPT model. Columns two, four and six display the tokens generated by the model, and the reconstructed molecule. The 2D diagrams of the molecules were drawn from the SMILES sequences using RDKit.

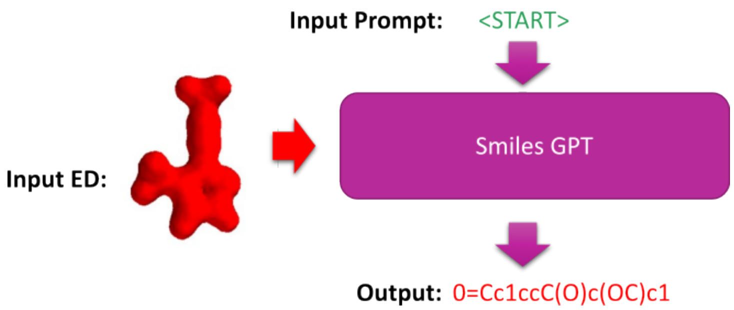

Supplementary Figure 20: The transformer model expanded the previously discussed GPT model by adding an encoder that took as input electron densities, while the decoder received as input an SMILES sequence and generated the related SMILES sequence. The input electron was processed through the Encoder The Decoder focused on generating SMILES tokens, but its attention layers received information from the encoder.

The user should start or continue training the model with the following command:

```
$ python bin/train/train_gpt.py
```

For this command to work properly, the "electrondensity" Conda environment must be enabled (see Supplementary Section 1.1).

Supplementary Subsection 1.11 Using the Transformer model to generate SMILES sequences from electron densities and/or electrostatic potentials

While the just described SMILES GPT model (Supplementary Section 1.10) is a purely generative model, from SMILES to SMILES, it is the objective now to expand this model to transform electron densities (or electrostatic potentials or a combination of both – from now on when electron densities are mentioned, it might refer to any of these three possibilities) into SMILES. The reasoning behind this is that this research focused on operating with molecules represented as 3D data such as electron densities, but to fully characterise a molecule its atoms and positions must be known – having only the 3D data is not enough. Therefore it is the objective now to create a machine learning model that will take as input 3D data such as electron densities, and will output the SMILES sequence that best describes it, see Supplementary Figure 20.

1.11.1 src/models/ED2smiles.py

Our electron densities to SMILES architecture is heavily based on the following Keras example:
`https://keras.io/examples/audio/transformer_asr/`

As in this example, we used the decoder to generate strings of characters (in our case SMILES sequences), while the encoder was used to process the multi-dimensional data (in our case 3D electron densities). Therefore, the encoder received as input 3D tensors

containing electron densities (see Supplementary Section 1.3.3), used stacked Transformer blocks to extract and calculate attention matrices, and sent these attention matrices to the decoder. The decoder had two inputs: the attention matrices from the encoder and the sequences of SMILES tokens from the dataset. The decoder used these inputs to calculate the best SMILES sequence related molecule inputted as electron density. See Supplementary Figure 21 to see the model architecture.

Our implementation of the Encoder, Decoder and Token Embedding were the same as in the shared example, with very minor variations. Our focus was placed on designing an encoder "Molecule Embedding" layer that would take as input a 3D tensor representing the electron density of a molecule, and would output 2D data that could operate in the decoder with the 2D data from the Token Embedding.

To achieve this transformation from 3D to 2D, first the 3D data was expanded to 4D so that 3D convolutions could be applied. To transform the 4D tensors into 2D, we tested two different strategies:

The first one started with 3D convolutions, setting the number of filters to 1, dropping the dimension with size 1 after the convolution had been done, and then repeating this process with 2D convolutions and 1D convolutions until the data was 2D. As an example, if the initial 4D was (64, 64, 64, 64), setting the number of filters to 1 would output (1, 64, 64, 64) and then dropping the first dimension would output (64, 64, 64). If this process is repeated again, we would first obtain (1, 64, 64), and then dropping the first dimension we would obtain 2D data: (64, 64).

The second strategy used again 3D convolutions, but their strides were of different sizes depending on the dimension. These convolutions were applied until two of the dimensions had a size of 1, and then dropping them, thus getting again 2D data. As an example, if the initial 4D data was (64, 64, 64, 64) and the strides of the 3D convolutions were (1, 2, 2), keeping the number of filters to 64, an initial convolution would output (64, 64, 32, 32). We can repeat these convolutions with these strides until it outputs (64, 64, 1, 1), and then dropping the two single unit dimensions, to obtain (64, 64). We expected the second strategy to produce better results, since it kept the data 4D for longer, but both strategies produced very similar results.

Supplementary Figures 22, 23 and 24 show in the left columns different electron densities that were inputted to the described model, and in the right columns the SMILES outputted by the model (with a drawing of the molecule using RDKit).

As happened with the SMILES GPT architecture described before, the generation of SMILES sequences could follow a greedy approach, or a probabilistic sampling one (see Supplementary Section 1.10.2). An interesting feature of selecting the next token using probabilistic sampling is that the model could be used to generate new molecules that fit a particular pocket defined by a target electron density. Supplementary Figure 25 shows an example of this application. On the left of this Figure there can be seen the target electron densities and their related molecules. On the right section there can be seen different molecules generated using probabilistic sampling that could fit this pocket.

1.11.2 src/models/ESP2smiles.py

This script describes the same model as just discussed (Supplementary Section 1.11.1), but taking as input electrostatic potentials instead of electron densities. The electrostatic potentials were obtained from the QM9 dataset as explained in Supplementary Section 1.3.3, and they were preprocessed by using dilations as discussed in Supplementary Sec-

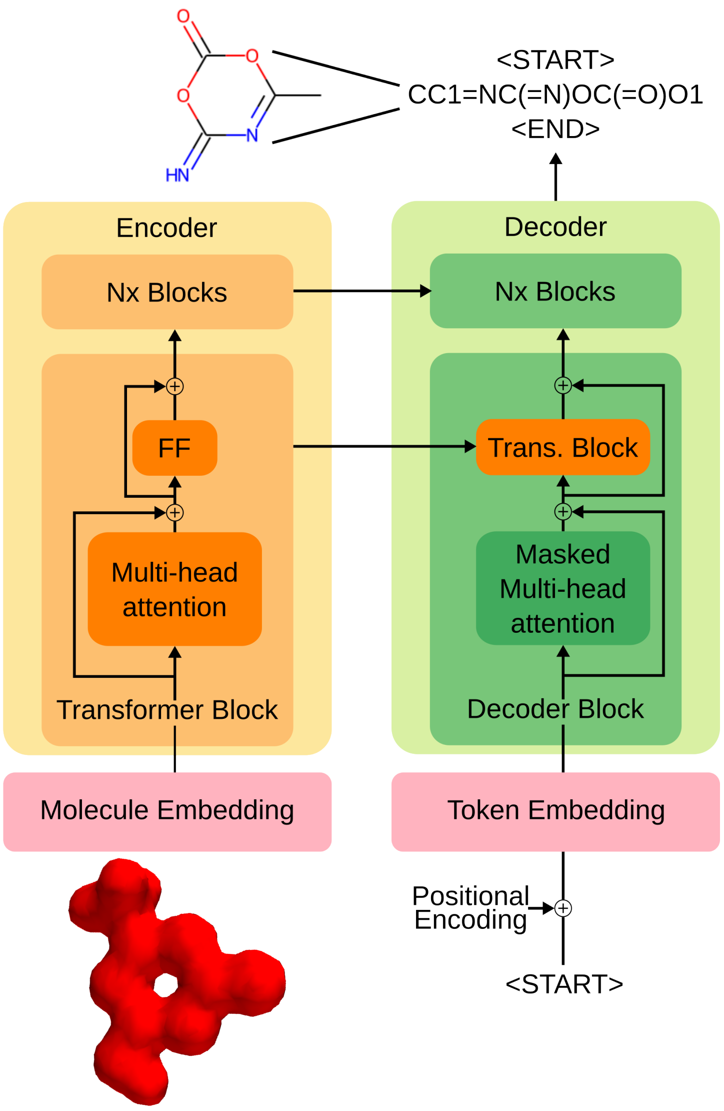

Supplementary Figure 21: Examples of SMILES sequences generated using the described architecture. The Encoder received the 3D electron density, and processed it through it attention layers. The decoder received as input the "<START>" token, and the attention matrix from the encoder. Using this information, the decoder generated a SMILES sequence representing the Electron Density inputted through the encoder.

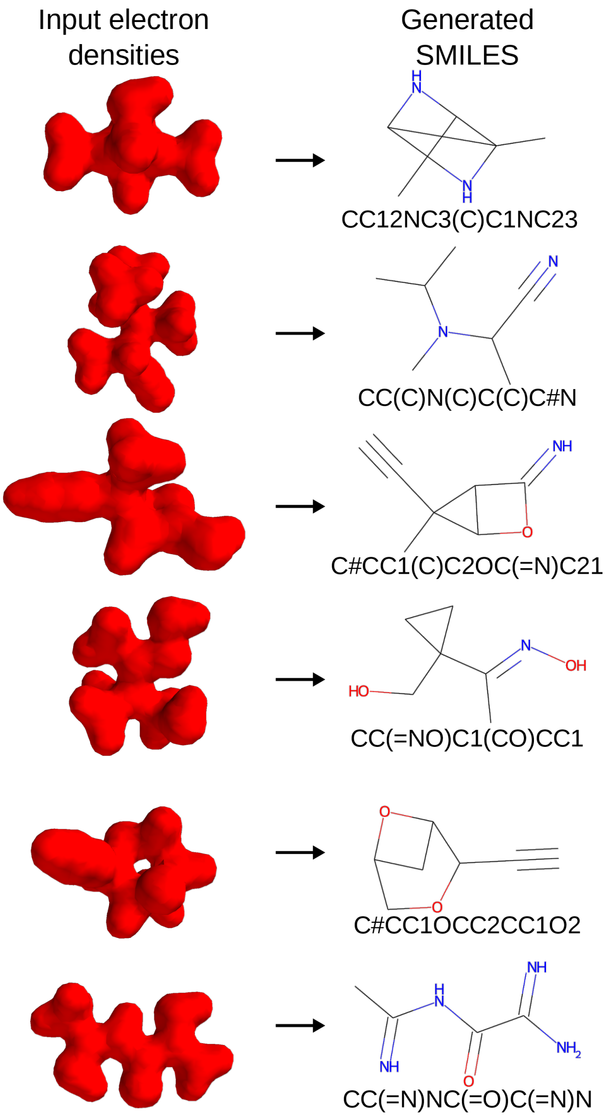

Supplementary Figure 22: Examples of translated electron densities into SMILES sequences. Left: Examples of 3D electron densities inputted to the Encoder. Right: The generated SMILES sequences outputted by the Decoder. The 2D diagrams of the molecules were drawn from the SMILES sequences using RDKit.

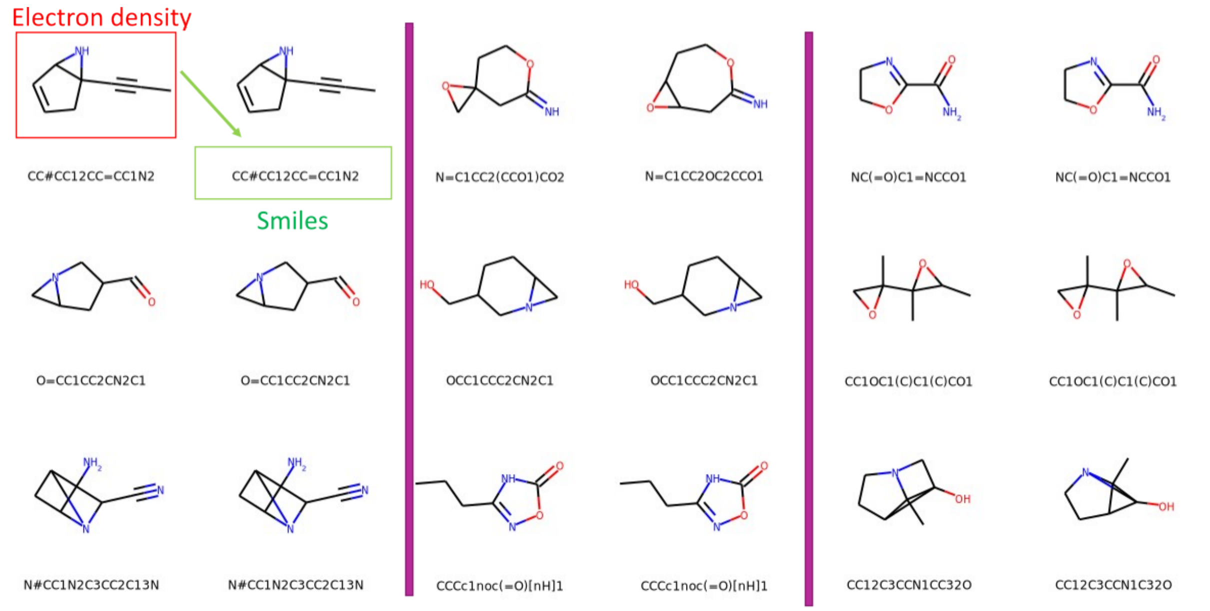

Supplementary Figure 23: Examples of translated electron densities into SMILES sequences. For each of the pairs of molecules shown, the electron density of the molecules on the left was used as input, and the model outputted the SMILES sequence on the right. The 2D diagrams of the molecules were drawn from the SMILES sequences using RDKit.

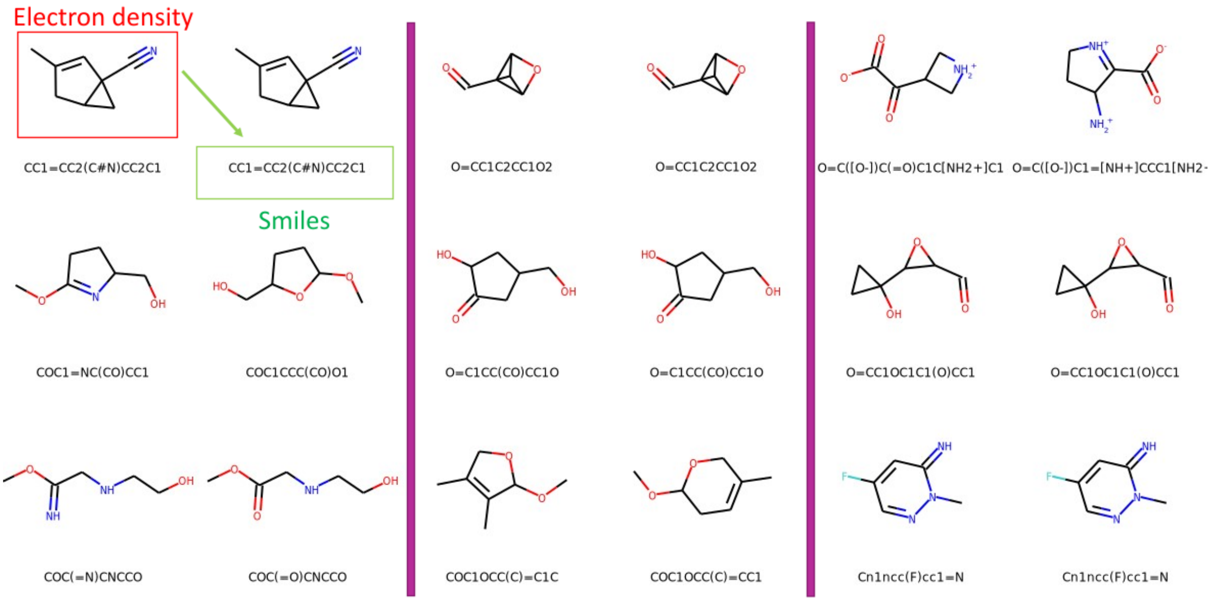

Supplementary Figure 24: Examples of translated electron densities into SMILES sequences. For each of the pairs of molecules shown, the electron density of the molecules on the left was used as input, and the model outputted the SMILES sequence on the right. The 2D diagrams of the molecules were drawn from the SMILES sequences using RDKit.

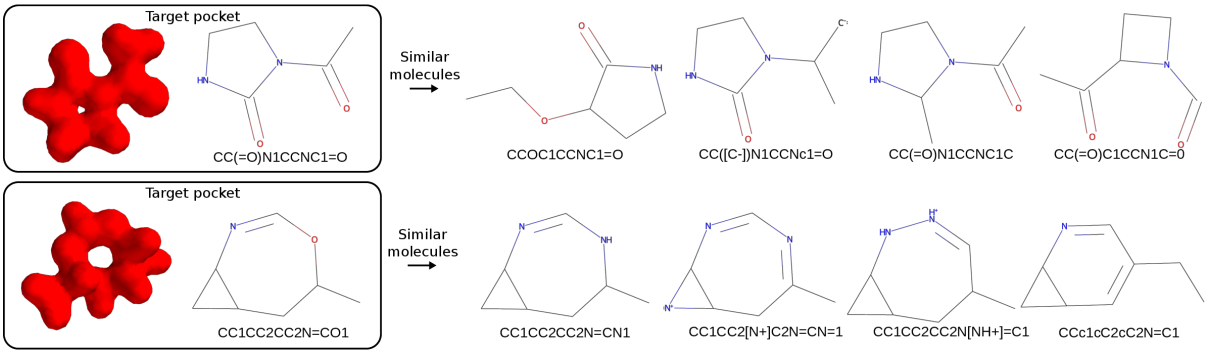

Supplementary Figure 25: Using the transformer model and probabilistic sampling to find molecules with similar electron density pockets. The electron density displayed in red was used as input. The decoder chose the next token using probabilistic sampling.

tion 1.6.1.

1.11.3 src/models/ED_ESP2smiles.py

This script describes the same model as just discussed (Supplementary Section 1.11.1), but taking as input electron densities decorated with electrostatic potentials. The electron densities and electrostatic potentials were obtained from the QM9 dataset as explained in Supplementary Section 1.3.3, and pre-processed and combined as explained in Supplementary Section 1.8. This is the method which offered the best results.

1.11.4 bin/train/train_e2s.py esp2s.py edesp2smiles.py

These three scripts are used to trained the three models just described:

- `bin/train/train_e2s.py` can be used to train the transformer model that takes as input electron densities (see Supplementary Section 1.11.1).
- `bin/train/train_esp2s.py` can be used to train the transformer model that takes as input electrostatic potentials (see Supplementary Section 1.11.2).
- `bin/train/train_edesp2smiles.py` can be used to train the transformer model that takes as input decorated electron densities (see Supplementary Section 1.11.3).

These three scripts are exactly the same, and they are very similar to the training scripts discussed before (see Supplementary Sections 1.5.4 and 1.6.2). The only differences between these three scripts are that the scripts that used electrostatic potentials as part of the training data needed to fetch this data from the dataset (see lines 40 and 41), and that the correct class needed to be chosen when creating the object that will do the training (see line 54).

The main difference between these three training scripts and the training scripts we discussed previously (such as the training script discussed in Supplementary Section 1.5.4) relates to the parameters used when creating the training object (lines 55 to 59):

- "num_hid" refers to the number of hidden dimensions used in the model. This is similar to the embedding dimension discussed during the GPT implementation (see

Supplementary Section 1.10.3). This parameter was used to define the number of filters the convolutions used inside the molecular embedding, and also the number of neurons in the last dense layer inside the transformer blocks used both in the encoder and decoder.

- "num_head" refers to the number of heads used when defining the multi-head attention.
- "num_feed_forward" refers to the number of neurons in the first dense layer inside the transformer blocks used both in the encoder and decoder.
- "num_layers_enc" and "num_layers_dec" refer to the number of stacked transformed blocks used both in the encoder and decoder.

The user should start or continue training the model with the following command:

```
$ python bin/train/train_e2s.py
```

Or any of the other two scripts discussed in this supplementary section. Remember that for this command to work properly, the "electrondensity" Conda environment must be enabled (see Supplementary Section 1.1).

When executed in "build" mode, this script created a new folder inside `logs/e2s` named with the current date, for example `logs/e2s/2022-06-09`. The name of the `e2s` subfolder depended on the script used: `train_e2s.py` used `e2s`, `train_esp2s.py` used `esp2s`, and `edesp2smiles.py` used `ed_esp2smiles`. Inside the folder named with the current data (for example `logs/esp2s/2022-06-09`), there will be a file `params.pkl` and two folders named `smiles` and `weights`:

- `params.pkl` is a Python Pickle file containing the parameters used to create the model.
- `smiles` contains a number of Pickle files that were created each time the callback function was used to sample the model. These Pickle files contained SMILES sequences generated using the validation set.
- `weights` contains a list of "h5" files. Each of these files contained the weights of the model saved after a particular epoch. These are the files that later on can be loaded into the model to either keep training (when executed in load mode) or to test new data.

1.11.5 Testing the accuracy of the electron density to SMILES translation

The source code included three different scripts (one for each type of input as described above) to benchmark the accuracy of the Transformer model once it had been trained. These benchmark scripts tested the accuracy of the translation using molecules from the validation dataset (built from the QM9 dataset, see Supplementary Section 1.3.2). To benchmark our models, the SMILES sequences generated using electron densities as input were compared against the SMILES sequences from the QM9 dataset. Further benchmarking to test the quality of the data generated using the Transformer model, such as the diversity of SMILES generated, is discussed in Supplementary Section 4.

The three scripts used to test the accuracy can be found in `src/utils`:

- `test_accuracy_ed2smiles.py` used as input electron densities via the model described in Supplementary Section 1.11.1 and trained with the script `bin/train/train_e2s.py` as explained in Supplementary Section 1.11.4.
- `test_accuracy_esp2smiles.py` used as input electrostatic potentials via the model described in Supplementary Section 1.11.2 and trained with the script `bin/train/train_esp2s.py` as explained in Supplementary Section 1.11.4.
- `test_accuracy_edesp2smiles.py` used as input decorated electron densities via the model described in Supplementary Section 1.11.3 and trained with the script explained in Supplementary Section 1.11.4 (`bin/train/edesp2smiles.py`).

These three scripts are identical with the exception of the data and model loaded. They can be executed with a command such as:

```
$ python src/utils/test_accuracy_e2s.py logs/ed2smiles/2021-08-31/weights/
```

The actual name of the script is `test_accuracy_ed2smiles.py` (or any of the others discussed in the list before). In this example it has been shortened to `test_accuracy_e2s.py` so that it fits in a line of text. The second part of the command (in this example `logs/ed2smiles/2021-08-31/weights/`) is the folder were the weights to test were saved.

Once executed, the script started by creating a "txt" file inside the weights folder, using as name the current date (including hour and minutes). It then loaded the QM9 validation dataset TFRecord using a TFRecordLoader as in the training scripts, and then it created a model as in the training script. Then, for every h5 file inside the weights folder, it loaded these weights into the model, fetched N batches from the validation dataset (in our tests N=5), and used the model to generate the SMILES sequences related to each molecule in each batch. The generated SMILES were cleaned (more on this later) and compared with the original QM9 SMILES from the QM9 dataset. This comparison was done in two ways:

1. Perfect molecule matching. If the target molecule is "O=C=O" only an output of exactly "O=C=O" will be considered as valid.
2. Token matching. If the target molecule is "O=C=O" and the model outputted "O=C=C" this would consider that 4 of the 5 tokens were guessed correctly, and assigned to this entry an 80% accuracy.

The accuracy returned for each of these two ways was the average through all the batches and through all the molecules. In our tests we used 5 batches of 64 molecules, thus the value returned was the total over 320 molecules.

To match the SMILES generated through the model, with the ones as fetched from the QM9 dataset, the generated ones had to be "cleaned". To clean them, the outputted SMILES sequences were processed in the following way:

1. Every null token was replaced with a stop token.
2. Every token after the first stop was replaced with a stop token.

1.11.6 Translating electron densities into SMILES using a trained model

This supplementary section focuses on using a trained model to translate electron densities (or electrostatic potentials, or decorated electron densities) into SMILES. To perform this translation it is mandatory to have a trained model (the h5 file) using one of the training scripts described in Supplementary Section 1.11.4. Depending on the input data (electron densities, electrostatic potentials, or decorated electron densities) one of the following scripts found in `src/utils` should be used:

- `ed_to_smiles.py` used as input electron densities via the model described in Supplementary Section 1.11.1 and trained with the script `bin/train/train_e2s.py` as explained in Supplementary Section 1.11.4.
- `esp_to_smiles.py` used as input electrostatic potentials via the model described in Supplementary Section 1.11.2 and trained with the script `bin/train/train_esp2s.py` as explained in Supplementary Section 1.11.4.
- `edesp_to_smiles.py` used as input decorated electron densities via the model described in Supplementary Section 1.11.3 and trained with the script explained in Supplementary Section 1.11.4 (`bin/train/edesp2smiles.py`).

These three scripts are identical with the exception of the model loaded. They can be executed with a command such as:

```
$ python src/utils/ed2smiles.py electron_densities.p
```

Where `electron_densities.p` should be the file with the electron densities (or any of the other inputs) that we want to translate to SMILES. For this command to work properly it is important to have enabled the "electrondensity" Conda environment (as explained in Supplementary Section 1.1). These scripts made heavy use of the RDkit software package, which was installed as part of this Conda environment.

These scripts started by loading the Transformer model with the trained weights. To properly load the model, first it was needed to indicate where the dataset was located in disk (line 85) and then indicate where the weights were (line 94). To properly load the model, the script needed to load a batch of data, and use this batch to build the model. This data was not used later on, but it was needed to build the model. Once the model was loaded with the weights, the script continued loading the tokenizer, which used a Python dictionary saved as a Pickle file, linking SMILES token to number ids. This file should be in the same folder as were the data was (like 85). Once the tokenizer is loaded, the next step loaded a Pandas file which contained a list of all the known commercial molecules. This data was used later on to visualise the molecules and quickly discern if the molecules were commercially available or not. The script continued loading the data we passed as argument when launching the script (in the example above this file was `electron_densities.p`). Once this data had been loaded, it was inputted into the model to obtain the related SMILES sequences. Finally, for each SMILES molecule, they were marked as commercially available or not, its synthetic accessibility score was calculated and displayed, and a 2D visual diagram of the molecule was drawn using RDKit.

After this script was executed, the user received a PNG file, with all the SMILES as outputted by the ML model, their related 2D diagrams, the synthetic accessibility score, and its commercial availability.

Supplementary Subsection 1.12 Using the Transformer model to generate SELFIES sequences from electron densities and/or electrostatic potentials

In exactly the same way as in the previous section (Supplementary Section 1.11), a series of scripts are also provided to transform electron densities and/or electrostatic potentials into SELFIES sequences. It is worth noting that the Transformer decoder receives tokenised sequences (where SMILES or SELFIES tokens are converted into id numbers), therefore from its perspective it doesn't matter if the inputs are SMILES or SELFIES because all its seeing are tokens such as '11' or '32'. This means that the changes to generate SELFIES instead of SMILES are minimal:

1. The Transformer model created to generate SELFIES can be seen in `src/models/ED_ESP2selfies.py`. This model is very similar to the one discussed in Supplementary Section 1.11.3. We are only focusing on generating SELFIES from decorated electron densities since this input is the one that generated better results.

2. This model can be trained with `bin/train/train_edesp2selfies.py`.

3. Once the model has been trained, we can finally use it to generate SELFIES sequences from decorated electron densities as inputs. To do so, we can use the script `src/utils/edesp_to_selfies.py`.

This process is very similar to the one described in Supplementary Section 1.11.6, but for SELFIES instead of SMILES.

Supplementary Section 2 Optimisation algorithms and optimisation results

Note: Originally our code only focused on SMILES, but based on the suggestion from one of the reviewers, we extended it to work with SELFIES too. All the following optimisation algorithms can be used with either of them.

The main results reported in the main manuscript focussed on finding guest molecules that best fitted inside a target host. To achieve this, the machine learning models described in Supplementary Section 1 were used inside an optimisation pipeline, where gradient descent was used to optimise latent vectors as generated by the VAE to output 3D guests that fitted inside the target host, and then the Transformer model was used to convert these 3D guests into SMILES sequences to fully characterise them. See Supplementary Figure 26 for an outline of this process. In this Supplementary section it will be explained how the different optimisation pipelines were implemented, and the results we obtained when executing them.

All the different optimisation experiments followed the same steps:

1. Generate an initial population of guests.

2. Optimise this population of guests against a target fitness function using gradient descent.

3. Obtain the SMILES representation from the optimised guests.

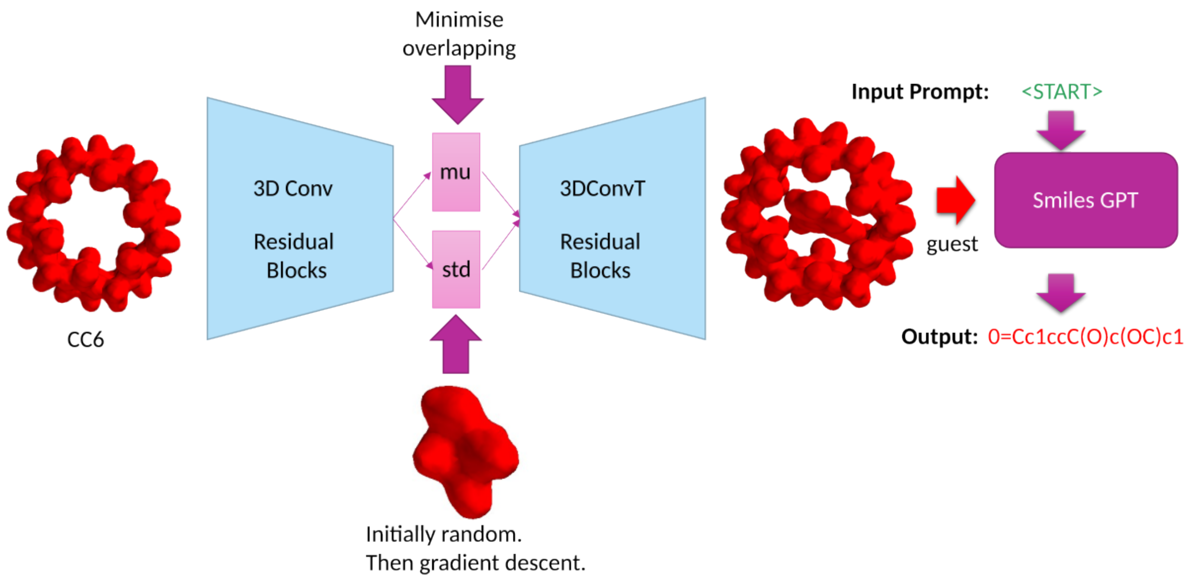

Supplementary Figure 26: Outline displaying the steps taken during the optimisation process: First a host was selected, then a random population of guests were generated, and gradient descent was used to fit them inside the host. Finally, the Transformer model was used to translate the outputted guest into its related SMILES sequence.

Supplementary Subsection 2.1 src/utils/optimiser_utils.py

The script `src/utils/optimisers_utils.py` contains the main building blocks that will be used later on to define the different optimisation pipelines. These building blocks relate to the following pipeline steps:

- Generate an initial population of guests (Supplementary Section 2.1.1).
- Optimise this population of guests against a target fitness function using gradient descent (Supplementary Section 2.1.2).
- Load a target host (Supplementary Section 2.1.3).
- Load the machine learning model (Supplementary Section 2.1.4).

2.1.1 Generating the initial population of guest

The function that generated the initial population of guests starts in line 21, "`initial_population`". This function had five parameters:

1. `batch_size` referred to the size of the population. We used populations as big as possible, which depending on the GPU were between 48 and 64.
2. `random` referred to if the initial population would be randomly generated or if existing data would be used instead. If random, the last two parameters can be ignored. All the reported results used random populations.
3. `z_dim` referred to the size of the latent vector. This size needs to match the size of the latent vector used when training the VAE. Our size was usually 400.

4. `datapath` referred to the path to find the data to be used to create the initial population. If the initial population was randomly generated this parameter was not used.

5. `vae` referred to a loaded VAE model that was used to generate the latent vectors from the initial population of molecules. If the initial population was randomly generated this parameter was not used.

Since all the reported results used a random initial population, we will only discuss this option. To generate the initial random population we used the `random_uniform` function from Keras Backend. The shape of the random uniform vector was defined by the batch size and the length of the latent vector, as just discussed in the list above. The other two parameters this function needed were "minval" and "maxval", which referred to the lower and upper boundary of the uniform distribution to draw samples from. We tested different boundaries, such as "-2,2" or "-5,5". Most of the reported results were obtained with "-2,2".

This function returned a tensor of size (`batch_size`, `z_dim`).

2.1.2 Fitness functions

The different optimisation experiments used a combination of the following three fitness functions with different objectives:

- To maximise the size of the molecule.
- To minimise the overlapping between host and guest electron densities.
- To maximise the interactions between host and guest electrostatic potentials.

A visualisation of these fitness functions can be seen on Supplementary Figure 27. All of them performed optimisation through gradient descent. This script contained the functionality required to perform for each of them one gradient descent step. The scripts describing the full optimisation experiments, with multiple gradient descent steps, can be found in Supplementary Section 2.2. We will now explain how a single optimisation step was performed for each of these fitness functions. Due to Tensorflow requirements, each of these functions was decorated with "@tf.function".

To perform one step towards maximising (or minimising) the size of a molecule, the function `grad_size` (line 196) was used. This function received two parameters:

1. `noise` referred to a tensor of size (`batch_size`, `z_dim`) which represented the VAE latent vectors. This latent vector must refer to electron densities.

2. `vae` referred to a loaded VAE model. The latent vector of this VAE must be of size `z_dim`. This VAE must had been trained on electron densities.

To perform one step towards maximising the size of the molecule, this function did the following:

1. Used the VAE decoder to, given an input latent vector (`noise`), reconstruct the 3D shapes of the molecules.

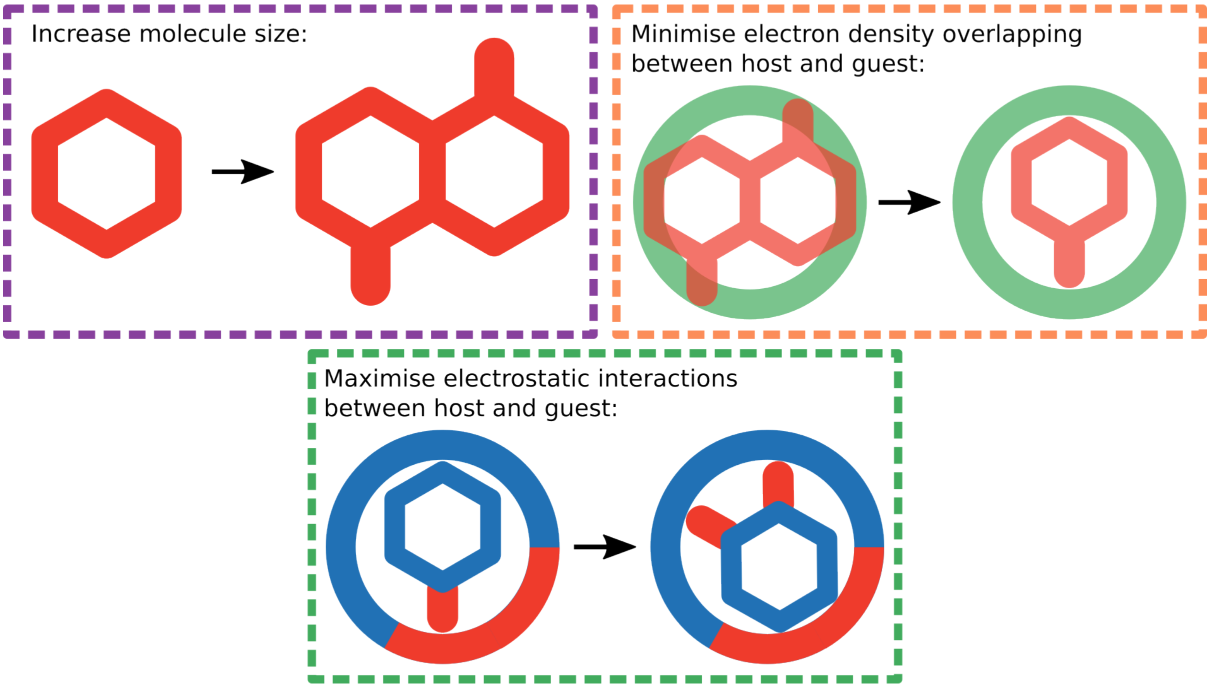

Supplementary Figure 27: The three fitness functions used in the different optimisation experiments. Top Left: This fitness function aimed to maximise the size of the molecule. Top Right: This fitness function aimed to minimise the overlapping between electron densities. Bottom: This fitness function aimed to maximise the electrostatic interactions between host and guest.

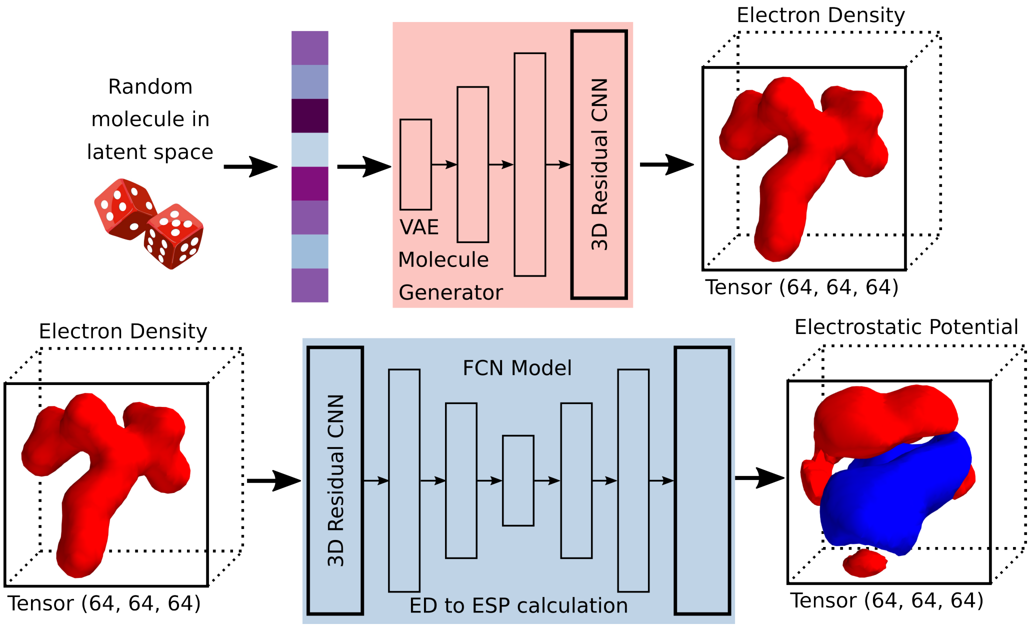

Supplementary Figure 28: Top: Generating a latent vector using a random distribution, and then using the VAE to reconstruct the 3D molecule from the latent vector. This was achieved using the decoder part of the VAE. Bottom: Using a Fully Convolutional Network to calculate the electrostatic potential from a molecule's electron density.

2. These 3D shapes just outputted by the VAE were post-processed in a identical way to how VAE outputs were post-processed (see Supplementary Section 1.5.1). We named this reconstructed 3D molecules as `output`. This step can be seen at the top of Supplementary Figure 28.

3. Tensorflow's `tf.reduce_sum` took as input `output` and was used to calculate a single value representing the whole 3D electron density by adding together the electron density at each location (within the 64,64,64 tensor). This value was used to define the `fitness` of each molecule.

4. Tensorflow's `tf.gradients` was used to calculate the changes needed to increase (or decrease) the fitness of the molecule. This function took as input two parameters: (1) `fitness` as just described in the previous point, (2) and `noise` as received as a parameter in the `grad_size` function. This function (`tf.gradients`) returned a tensor, which we named `gradients` which explained how to modify the latent vectors (`noise`) in order to maximise (or minimise if subtracted) their fitness values.

5. `grad_size` returned three tensors: fitness, gradients and output.

To perform one step towards minimising (or maximising) the overlapping between host and guest electron densities, the function `grad_ed_overlapping` (line 214) was used. This function received three parameters, being "noise" and "vae" the same as before. The new parameter was named "host" and it must be a 3D tensor representing the electron density of the target host.

To perform one step towards minimising (or maximising) the overlapping between host and guest electron densities, this function did exactly the five steps as the previous fitness function, with only two small differences:

- The variable which before was named as `output` in step two, was named as `guests` in this function.
- In step three `tf.reduce_sum` took as input the product between host and guest. Thus while before the code was `tf.reduce_sum(output)` now it is `tf.reduce_sum(guest*host)`.

With these two changes, the `gradients` vectors as returned by Tensorflow's `tf.gradients` indicated how to modify the latent vectors to minimise (or maximise if added) the overlapping between host and guest molecules.

To perform one step towards minimising (or maximising) the interactions between host and guest electrostatic potentials, the function `grad_esp_overlapping` (line 238) was used. This function received four parameters:

1. `noise` referred to a tensor of size (`batch_size`, `z_dim`) which represented the VAE latent vectors. This latent vector must refer to electron densities.
2. `vae` referred to a loaded VAE model. The latent vector of this VAE must be of size `z_dim`. This VAE must had been trained on electron densities.
3. `ed2esp` referred to a loaded machine learning model to calculate electrostatic potentials from electron densities. This was implemented using a FCN as detailed in Supplementary Section 1.6.
4. `hostesp` referred to a tensor describing the target host's electrostatic potential.

To perform one step towards maximising the electrostatic potential interactions, this function did the following:

1. Generated the 3D electron densities from the latent vectors (which were named as `noise` in the input parameters). This step is the same as steps one and two discussed when describing the gradient step to maximise the size of a molecule. The output of this step was named `guests`. This step can be seen at the top of Supplementary Figure 28.
2. Used the model `ed2esp` to calculate the electrostatic potentials from `guests`. This step can be seen at the bottom of Supplementary Figure 28.
3. The output from `ed2esp` (a tensor with values from 0 to 1) was post-processed to mimic the original data: first they were normalised between -1 and 1, and finally they were multiplied by "0.33" to be in the same range as the original data in the training set. We named the output from this step `guests_esps`.
4. Tensorflow's `tf.reduce_sum` took as input the product between `guests_esps` and the input parameter `hostesp`. We named the result of this calculation `fitness`.
5. Tensorflow's `tf.gradients` was used to calculate the gradients in the same was as before. This function took as input the just calculated `fitness` and the input parameter `noise`. We named the result of this step `gradients`.
6. This function returned the following four variables: fitness, gradients, guests and guests_esps.

2.1.3 Loading hosts

This script contained functions to load the different hosts used during our research. To obtain a host to load into our system, first the XYZ file of the target host must be obtained, and it should be processed in exactly the same way as we did with the QM9 molecules, see Supplementary Section 1.3.3. Thus, from the initial XYZ file, we must calculate its electron density and electrostatic potential in exactly the same way as before. The results must be saved in a Pickle file. A single Pickle file must be used for each host. In the results described in the main manuscript we used two different hosts: CB6 and Pd cage.

This script contained two functions to load the CB6 host:

- `load_host` loaded the electron density of the target host into a Tensorflow tensor. This function had two parameters: (1) `filepath` must be the path to the Pickle file that contains the host — its electron density. (2) `batch_size` must be a number, and it represented how many times the host was repeated in the output tensor. This function loaded the Pickle file into a variable, and then used Tensorflow's `tf.tile` to transform it into a Tensorflow tensor, repeated as many times as required by the `batch_size` parameter.

- `load_host_ed_esp` loaded the electron density and electrostatic potential of the target host into two different Tensorflow tensors, one for each type. This function had three parameters: (1) `filepathED` must be the path to the Pickle file that contains the host — its electron density. (2) `filepathESP` must be the path to the Pickle file that contains the electrostatic potential of the host. This must be in sparse format, es discussed in Supplementary Section 1.3.3. This function would, after loading the sparse tensor, dilate it using five by five windows, and then scale the data between 0 and 1. This is the same process as discussed in Supplementary Section 1.6.1. (3) `batch_size` must be a number, and it represented how many times the host was repeated in each of the output tensor. This function loaded the two Pickle files into two variables, dilated the electrostatic potential, and then used Tensorflow's `tf.tile` to transform them into Tensorflow tensors, repeated as many times as required by the `batch_size` parameter.

The script also contained two functions to load the Pd cage host. These two functions are identical to the previous ones. The only difference is that the Pd cage did not fit in a (64,64,64) tensor, but an (80,80,80) was required instead. Therefore these two functions take the Pickle files with tensors of size (80,80,80) and cuts them around the borders to fit a size (64,64,64). This removed some of the outer parts of the electron density, but since we only cared about the inside cavity, this was not a problem. To perform this operation (transforming the original cage with size 80,80,80 to a cage with size 64,64,64) we used Python's array slicing functionality. Thus this transformation was achieved with the following command:

$host = host[:, 8 : -8, 8 : -8, 8 : -8, :]$

Check Supplementary Figure 29 to see the Cage host before and after this transformation.

2.1.4 Loading the machine learning models

The optimisation experiments used two different machine learning models: (1) A VAE to convert latent vectors into 3D tensors representing a molecule (its electron density or its

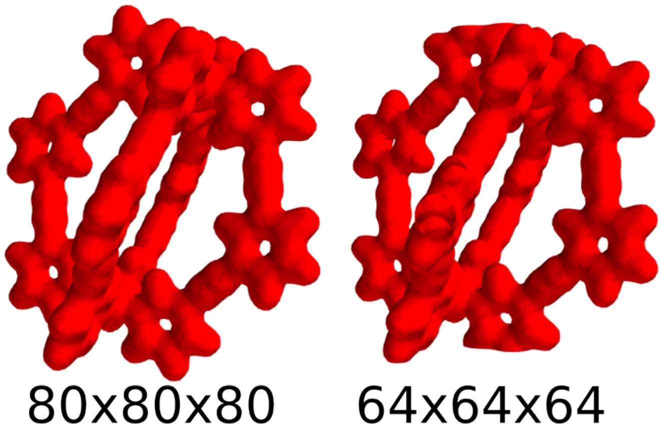

Supplementary Figure 29: Transforming the Pd Cage host to fit into a (64,64,64) Cube. Since the Pd Cage host was stored in a Numpy array, this transformation was done my simply slicing the array into the right size. Left: Fully sized Cage (size 80,80,80). Right: Transformed size with size (64,64,64).

electrostatic potential), see Supplementary Section 1.5, and (2) a FCN that transformed an electron density into its related electrostatic potential, see Supplementary Section 1.6. It has also been discussed the Transformer model that transforms electron densities into SMILES sequences. This model was not used during the optimisation experiments, but only at the end to convert the guest candidates into SMILES. We could have used it to obtain SMILES sequences during the optimisation processes, and then use this information as part of the optimisation, but it was decided not to do it and keep the optimisation processes using only structural 3D data.

This script contains two functions, one to load the VAE and one to load the FCN:

1. `load_vae_model` referred to the function used to load the VAE model. It had only one argument, "modelpath" which must be the path from where to load the model. Inside this path there must be a "params.pkl" file, with the parameters used when creating the model, and a folder named "weights" with a file named "weights.h5" inside, which contains the weights to load.

2. `load_ED_to_ESP` referred to the function used to load the FCN that would transform the electron densities into electrostatic potentials. As the previous function, it has one argument, "modelpath", which works in the same way as before.

Supplementary Subsection 2.2 Optimisation scripts

The scripts used to perform the different optimisation experiments can be found inside the `bin/optimisers` folder. Each of these scripts can be executed with a command such as:

```
$ python bin/optimisers/host_guest_overlapping.py
```

Where `host_guest_overlapping.py` can be replaced with any of the other scripts. Remember that for this command to work properly, the "electrondensity" Conda environment must be enabled (see Supplementary Section 1.1). The output of these commands differred depending on the script executed, but all of them saved into disk a number

of Pickle files containing the 3D electron densities (and/or electrostatic potentials) at different snapshots of the optimisation process. At the minimum these scripts saved the initial random population, and the final and optimised population. These Pickle files could then be inputted into the Transformer model (see Supplementary Section 1.11.6) to obtain the SMILES sequences related to each 3D electron density.

In this supplementary section we will know describe each of the optimisation scripts. The order at which they will be described here is the order at which the scripts were written and tested.

2.2.1 bin/optimisers/host_guest_overlapping.py

This script focused on running experiments that tried to find guests for the CB6 host. In this script the calculations were performed using only electron density data. Therefore, given a host's electron density, and a candidate guest's electron density, this script tried to minimise the overlapping between electron densities by manipulating the guest. How this manipulation was performed depended purely on the gradient descent steps, and it was transparent to the user. Checking the evolution of the molecules through the optimisation processes, the manipulations usually consisted on a mixture of rotations and decreasing the size of the guest, but the user had to input on this.

This script performed the following steps:

1. Line 27: The variable `BATCH_SIZE` was used to define the size of the population of guests.

2. Lines 28-29: The host's electron density was loaded into a variable (see Supplementary Section 2.1.3).

3. Line 30: The VAE model was loaded (see Supplementary Section 2.1.4).

4. Lines 32-40: An initial population of guests (in latent vector form) was randomly generated, see Supplementary Section 2.1.1. We named this population `noise_t`. From the latent vector `noise_t`. The 3D structures of these molecules (in latent form) were generated and saved into disk.

5. Line 42: A for loop started that iterated 10,000 times. This number of iterations was empirically chosen. This number of iterations seemed to make sure that the optimisation was finished. The following instructions are all inside the for loop.

6. Line 43 (inside the for loop): A gradient step was taken to minimise the overlapping between host and guests electron densities. This gradient step was performed using the function `grad_ed_overlapping` described in Supplementary Section 2.1.2. This function returned three values: fitness, gradients and outputs (the 3D shapes of the electron densities).

7. Line 44 (inside the for loop): The fitness value just obtained was displayed on screen.

8. Line 45 (inside the for loop): The `noise_t` latent vector was modified using the gradient information obtained before. In particular, this script performed the following manipulation: `noise_t-=0.05*gradient`. The"0.05" was empirically chosen, and it represented a sort of learning rate.

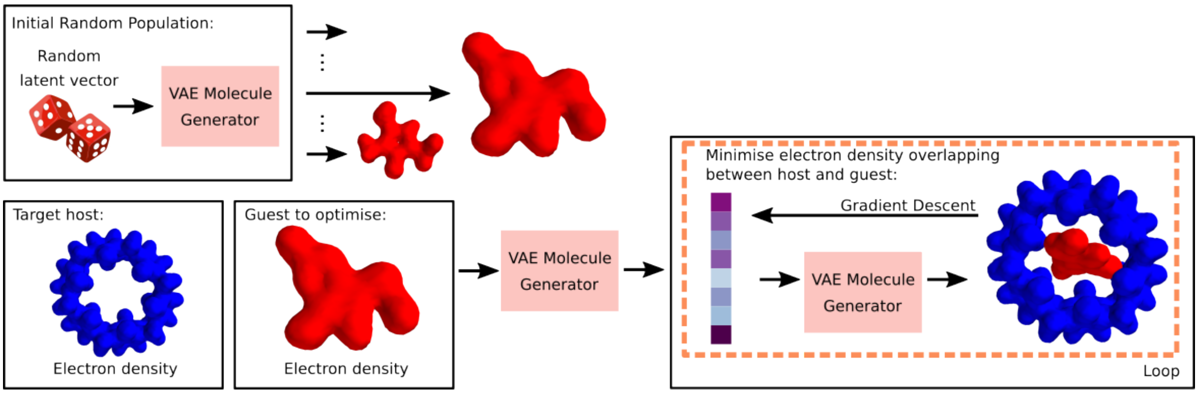

Supplementary Figure 30: Diagram outlining CB6 host-guest optimisation using only electron densities. First, an initial random population of guests was generated. Then, the overlapping between these guests and the target host was calculated. Finally, the guests were modified through gradient descent to minimise this overlapping.

9. Line 46 (inside the for loop): The `noise_t` latent vector was clipped between -4 and +4 using the "clip" function from Numpy. This clipping operation was performed to make sure that the latent vector did not divert towards impossible values. This operation finishes the gradient descent iteration.

10. Lines 48-53 (inside the for loop): The current guests were saved into disk. As the optimisation progressed, different snapshots of the guests population were saved into disk.

This process is outlined in Supplementary Figure 30. The electron densities obtained can be transformed into SMILES using a very similar process to the one depicted in Supplementary Figure 35, but using only the electron density data.

The results obtained using this script can be seen in Supplementary Section 2.3.1.

2.2.2 bin/optimisers/maximise_size.py

This script focused on running experiments that tried to maximise the size of a molecule by increasing the size of its electron density. In this script the calculations were performed using only electron density data.

This script performed the following steps:

1. Line 24: The variable `BATCH_SIZE` was used to define the size of the population of molecules.

2. Line 25: The VAE model was loaded (see Supplementary Section 2.1.4).

3. Lines 27-31: An initial population of molecules (in latent vector form) was randomly generated, see Supplementary Section 2.1.1. We named this population `noise_t`. From the latent vector `noise_t`. The 3D structures of these molecules (in latent form) were generated and saved into disk.

4. Line 33: A for loop started that iterated 10,000 times. This number of iterations was empirically chosen. This number of iterations seemed to make sure that the optimisation was finished. The following instructions are all inside the for loop.

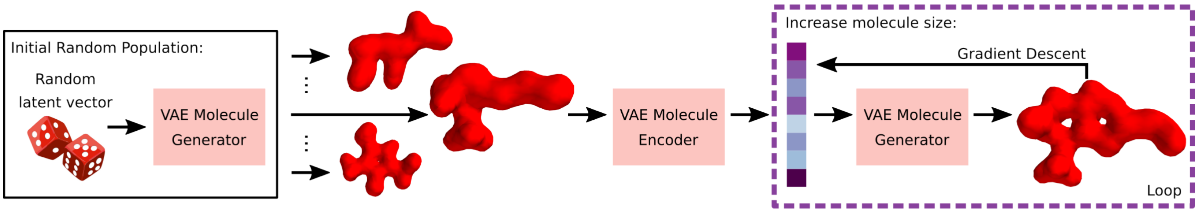

Supplementary Figure 31: Diagram outlining the optimisation process used to increase the size of the molecules using only electron density data. First, an initial random population of guests was generated. Then, the size (as in volume) of the guests was calculated. Finally, the guests were modified through gradient descent to maximise their sizes.

5. Line 34 (inside the for loop): A gradient step was taken to maximise the size of the molecule. This gradient step was performed using the function `grad_size` described in Supplementary Section 2.1.2. This function returned three values: fitness, gradients and outputs (the 3D shapes of the electron densities).

6. Line 35 (inside the for loop): The fitness value just obtained was displayed on screen.

7. Line 36 (inside the for loop): The `noise_t` latent vector was modified using the gradient information obtained before. In particular, this script performed the following manipulation: `noise_t-=0.001*gradient`. The"0.001" was empirically chosen, and it represented a sort of learning rate.

8. Lines 39-41 (inside the for loop): The current molecules were saved into disk. As the optimisation progressed, different snapshots of the molecules population were saved into disk.

This process is outlined in Supplementary Figure 31. The electron densities obtained can be transformed into SMILES using a very similar process to the one depicted in Supplementary Figure 35, but using only the electron density data.

The results obtained using this script can be seen in Supplementary Section 2.3.2.

2.2.3 bin/optimisers/maximise_polar.py

Note: This script was never used in the results shown in the main manuscript. It will be discussed here because it part of the source code, and we might used it in the future.

This script focused on running experiments that tried to maximise the polarity of a molecule. As discussed in Supplementary Section 1.3.3, the QM9 dataset, for each molecule, contained mainly three data entries: the atoms' XYZ positions, the molecule's SMILES representation, and 17 different single value properties, such as the dipole moment of the molecule, its internal energy at 0 K, ... One of these properties that is of interest here is the molecule's isotropic polarisability. Thus, the objective of this optimisation experiment was to perform gradient descent molecular optimisation based on this property. To achieve this, first we trained a neural network that took as input an electron density and outputted a single value related to the isotropic polarisability (see Supplementary Section 1.7 where this network was explained) Then, given a population

of electron densities, we could calculate using this neural network the isotropic polarisability for each of them, and then perform a gradient descent to either maximise or minimise it.

Before performing the optimisation loop, this script introduced two new functions:

1. Line 25: `load_PredictionModel`. This function has a single argument, `modelpath`, which must be the path to a model trained as discussed in Supplementary Section 1.7. This function returns the loaded model.

2. Line 44: `grad`. This is a fitness function very similar to the ones discussed in Supplementary Section 2.1.2. it has three parameters: noise and vae are the same as in the previous fitness functions; cnn3D must the a neural network loaded with the previous function (`load_PredictionModel`). To perform a gradient descent step, in a similar way to the previous fitness functions, first it used the VAE to generate the 3D electron densities from the latent vectors, then it used the "cnn3D" which took as input the just generated 3D electron densities, and outputted a single value for each electron density, related to their isotropic polarisability. We named this the fitness. Finally it used Tensorflow's `tf.gradients` to calculate the gradient, taking as input the fitnesses just calculated, and the latent vectors. This function returned three variables: the fitnesses, the gradients, and the 3D electron densities.

To perform optimisation based on the isotropic polarisability, this script performed the following steps:

1. Line 65: The variable `BATCH_SIZE` was used to define the size of the population of molecules.

2. Line 66: The VAE model was loaded (see Supplementary Section 2.1.4).

3. Line 67: The 3D-CNN model to calculate single value properties from electron densities was loaded (see Supplementary Section 1.7).

4. Lines 69-74: An initial population of molecules (in latent vector form) was randomly generated, see Supplementary Section 2.1.1. We named this population `noise_t`. From the latent vector `noise_t`. The 3D structures of these molecules (in latent form) were generated and saved into disk.

5. Line 75: A for loop started that iterated 10,000 times. This number of iterations was empirically chosen. This number of iterations seemed to make sure that the optimisation was finished. The following instructions are all inside the foor loop.

6. Line 76 (inside the for loop): A gradient step was taken to maximise the isotropic polarisability of the molecule. This gradient step was performed using the function `grad` described above. This function returned three values: fitness, gradients and outputs (the 3D shapes of the electron densities).

7. Line 77 (inside the for loop): The fitness value just obtained was displayed on screen.

8. Line 78 (inside the for loop): The `noise_t` latent vector was modified using the gradient information obtained before. In particular, this script performed the following manipulation: `noise_t-=0.01*gradient`. The"0.01" was empirically chosen, and it represented a sort of learning rate.

9. Line 79 (inside the for loop): The `noise_t` latent vector was clipped between -1 and +1 using the "clip" function from Numpy. This clipping operation was performed to make sure that the latent vector did not divert towards impossible values. This operation finishes the gradient descent iteration.

10. Lines 81-83 (inside the for loop): The current molecules were saved into disk. As the optimisation progressed, different snapshots of the molecules population were saved into disk.

The electron densities obtained can be transformed into SMILES using a very similar process to the one depicted in Supplementary Figure 35, but using only the electron density data.

The results obtained using this script can be seen in Supplementary Section 2.3.3.

2.2.4 bin/optimisers/maximise_hg_polar.py

This script focused on running experiments that tried to find guests for the CB6 host. In this script the calculations were performed using only electron density data. Given a host's electron density, and a candidate guest's electron density, this script tried to minimise the overlapping between electron densities by manipulating the guest, and it also tried to maximise the isotropic polarisability of the guest. Thus, this script combined the previously discussed scrips `host_guest_overlapping.py` (Supplementary Section 2.2.1) and `maximise_polar.py` (Section 2.2.3).

To achieve this, this script introduced a new fitness function, which combined the fitness function to minimise overlapping introduced in Supplementary Section 2.1.2 and the fitness function used to maximised isotropic polarisability discussed in the previous section (Supplementary Section 2.2.3). This fitness function was named `grad` (line 42). It had four input parameters, the first three (noise, vae and cnn3d) were exactly the same as the fitness function described in the previous section (Supplementary Section 2.2.3). The fourth one was the host, which is the same as in the fitness function `grad_ed_overlapping` described in Supplementary Section 2.1.2.

This fitness function performed the following steps:

1. Lines 56-57: Generated the 3D electron densities from the latent vectors (which were named as "noise" in the input parameters). This step is the same as steps one and two in the list describing the gradient step to maximise the size of the molecules. The output of this step was named "guests".

2. Line 59: Used Tensorflow's `tf.reduce_sum` to calculate the overlapping between hosts and guests (`tf.reduce_sum(guest*host)`). The result of this operation was named `fitness_overlapping`. This fitness value was multiplied by a negative number. We used "-10", which was calculated empirically.

3. Line 60: Used the input parameter "cnn3D" and the just generated "guests" to calculate the isotropic polarisability for each of the guests. The result of this operation was named `fitness_polarity`.

4. Line 61: Both fitness values were combined. `fitness=fitness_overlapping+fitness_polarity`.

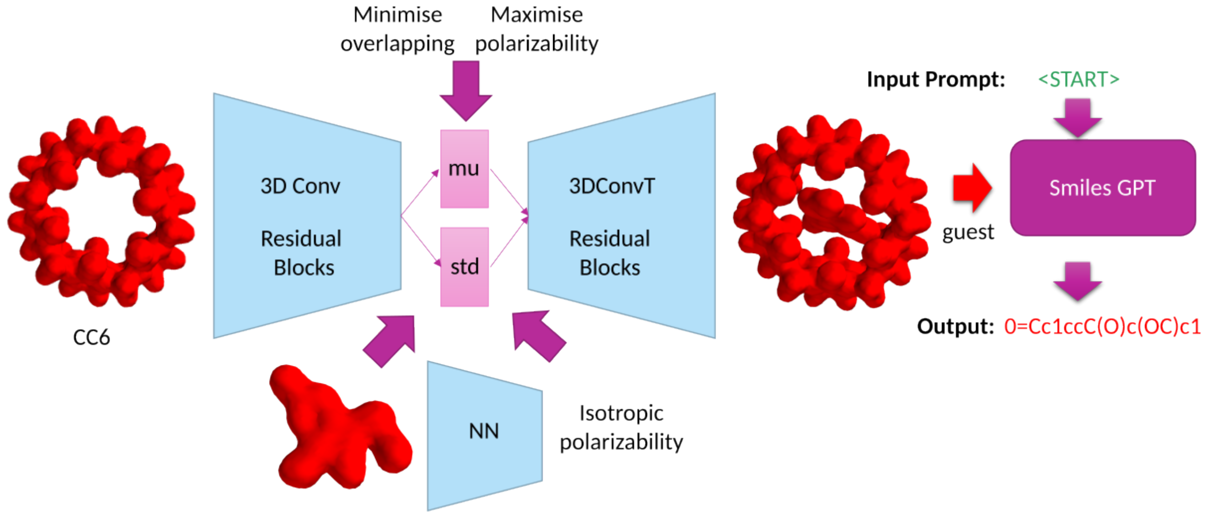

Supplementary Figure 32: Outline displaying the steps taken during the optimisation process: First a host was selected, then a random population of guests were generated, and gradient descent was used to fit them inside the host while maximising its isotropic polarisability. Finally, the Transformer model was used to translate the outputted guest into its related SMILES sequence.

5. Line 62: This "fitness" value was used with Tensorflow's `tf.gradients` function to calculate the gradients against the input latent vectors (noise). `gradients=tf.gradients(fitness,noise)`.

6. Line 63: The fitness function returned the following variables: fitness, gradients, guests, fitness_overlapping and fitness_polarity.

To perform optimisation based on minimising host/guest overlapping while maximising the guest polarity, this script followed very similar steps to the optimisation script discussed before in Supplementary Section 2.2.3. The only difference is that this script used the fitness function introduced in this Supplementary section, instead of the one used before. Everything else remained the same. Supplementary Figure 32 outlines the process this script did to find guests based on the described fitness function.

The electron densities obtained can be transformed into SMILES using a very similar process to the one depicted in Supplementary Figure 35, but using only the electron density data.

The results obtained using this script can be seen in Supplementary Section 2.3.4.

2.2.5 bin/optimisers/maximise_hg_esp.py

This script focused on running experiments that tried to find guests for the CB6 host. In this script the calculations were performed using both electron densities and electrostatic potentials. Given a host and a candidate guest, this script tried to minimise the overlapping between electron densities, and it tried to maximise the electrostatic interactions. See Supplementary Figure 33 for an outline of this process.

This script did not introduce any new fitness functions, and the optimisation source code is almost the same to the previous scripts, although this script introduced some new code which mostly focused on improving the output files generated so that it was easier

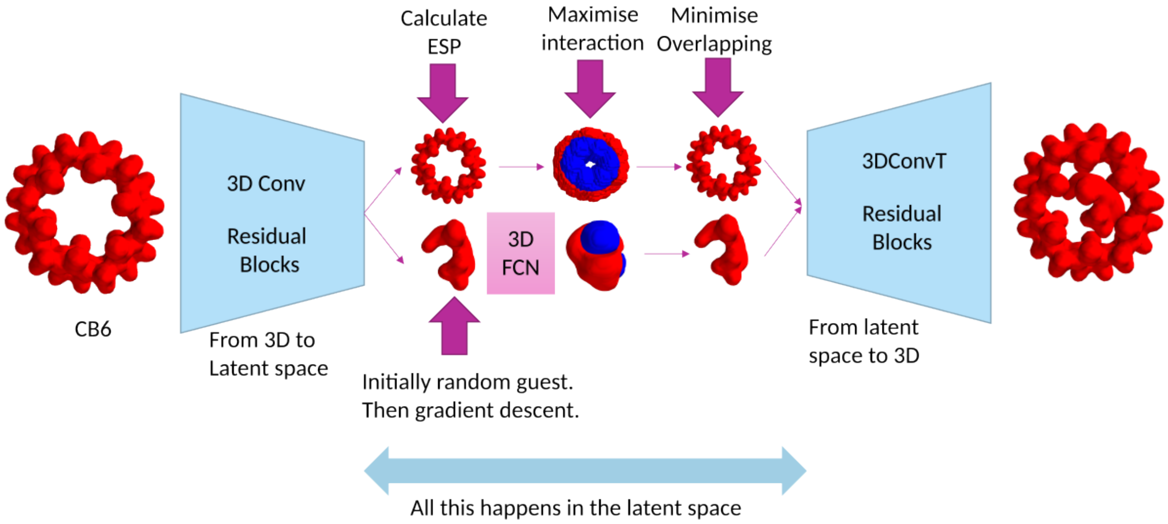

Supplementary Figure 33: Outline displaying the steps taken during the optimisation process: First a host was selected, then a random population of guests were generated, and gradient descent was used to fit them inside the host while maximising the electrostatic interactions between host and guess.

to analyse them later on. We will first discuss the new source code added, and then we will focus on the optimisation procedures.

1. Line 30. A variable named `ed_factor` is declared here. This script used two different fitness functions: one to minimise the overlapping between host and guests, and one to maximise the electrostatic interactions. This variable `ed_factor` explained how these two fitness functions were combined. A simple calculation such as `fitness=r*f1+(r-1)*f2` was followed, where "r" would be replaced by `ed_factor`. The fitness related to minimising electron densities overlapping was multiplied by `ed_factor`, while the part related to maximising electrostatic interactions was multiplied by `1-ed_factor`.

2. Lines 32-41: A folder is created where to save the files the optimiser created. This folder was named using the current date.

3. Lines 47-48: The CB6 host was loaded using the function `load_host_ed_esp`, explained in Supplementary Section 2.1.3.

4. Lines 49-50: The AI models were loaded. These models were the VAE model and the NN that converts electron densities to electrostatic potentials. This functions were explained in Supplementary Section 2.1.3.

5. Lines 52-63: An initial population of molecules (in latent vector form) was randomly generated, see Supplementary Section 2.1.1. We named this population `noise_t`. From the latent vector `noise_t`, the 3D structured were generated and saved into disk. The 3D structures created here were the electron densities and the electrostatic potentials.

This would finish the set-up, and the optimisation loop started. While before the optimisation ran in a loop for 10,000 iterations with a fixed learning rate, now the loop used

a variable learning rate that decreased as more iterations were executed. To implement this, we defined a variable named `factor`, which iterated through the following values: 1, 5, 10, 20 and 50. For each of these values, `10,000/factor` optimisation steps were taken, using as learning rate `lr=0.05/factor`. Therefore, initially 10,000 iterations were taken using a learning rate of 0.5. Then, 2000 iterations were taken using a learning rate of 0,01; followed by 1000 iterations with a learning rate of 0.005, 500 optimisation steps with a learning rate of 0.0025, and finishing with 200 optimisation steps with a learning rate of 0.001.

Another important consideration of this script is that it combined two different fitness functions (electron density overlapping and electrostatic interactions) into a single value. How the two fitness functions were combined was explained at the start of this supplementary section when the `ed_factor` variable was discussed.

The optimisation loop was implemented with two for loops:

1. Line 66: The outer loop started. In this loop, `factor` iterated being assigned values in the list `[1,5,10,20,50]`.
2. Lines 67-67 (inside outer loop): The learning rate was calculated from factor (`lr= 0.05/factor`). The calculate learning rate was also converted into a string to print it later on.
3. Line 70: (inside outer loop): The inner loop started. This inner loop iterated `10000/factor` iterations.
4. Lines 72-73 (inside inner loop): The gradient of the current latent vector was calculated using the `grad_esp_overlapping`, which calculated the electrostatic interactions, see 2.1.2. The fitness value returned by this function was printed.
5. Lines 74-75 (inside inner loop): The latent vectors were modified using the gradient information just calculated. The latent vectors were modified using this command: `noise_t-=lr*gradients*(1-ed_factor)`. After this, the latent vectors were clipped between -5 and 5, in a similar way to how was discussed previously.
6. Lines 77-81 (inside inner loop): The current guests were saved into the folder created before.
7. Lines 84-85 (inside inner loop): The gradient of the current latent vector was calculated using the `grad_ed_overlapping`, which calculated the overlapping between host and guest electron densities, see 2.1.2. The fitness value returned by this function was printed.
8. Lines 86-87 (inside inner loop): The latent vectors were modified using the gradient information just calculated. The latent vectors were modified using this command: `noise_t-=lr*gradients*ed_factor`. After this, the latent vectors were clipped between -5 and 5, in a similar way to how was discussed previously.
9. Lines 89-91 (inside inner loop): The current guests were saved into the folder created before.

This process is outlined in Supplementary Figure 34. The electron densities obtained can be transformed into SMILES using a the process depicted in Supplementary Figure 35.

The results obtained using this script can be seen in Supplementary Section 2.3.5.

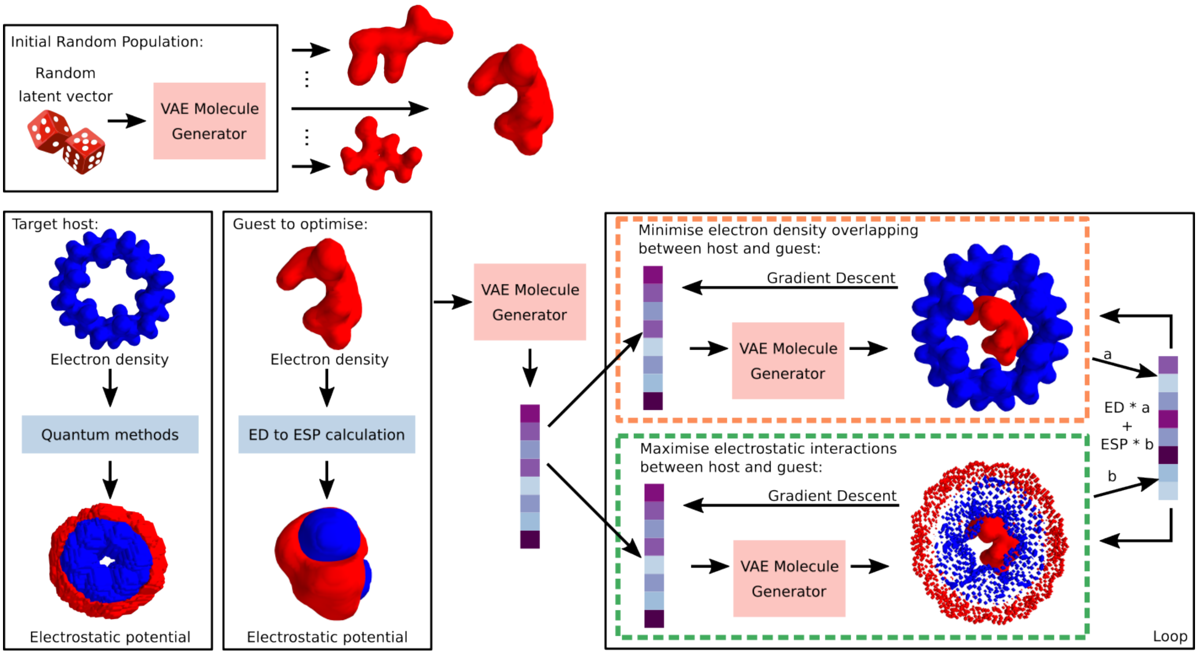

Supplementary Figure 34: Diagram outlining the optimisation process used to find guests for the CB6 host, using both electron densities and electrostatic potentials. First, an initial random population of guests was generated. Then, the overlapping between these guests and the target host was calculated. After this, the electrostatic interactions between the guests and the host were calculated. Finally, the guests were modified through gradient descent to minimise the overlapping of electron densities, while maximising the electrostatic interactions.

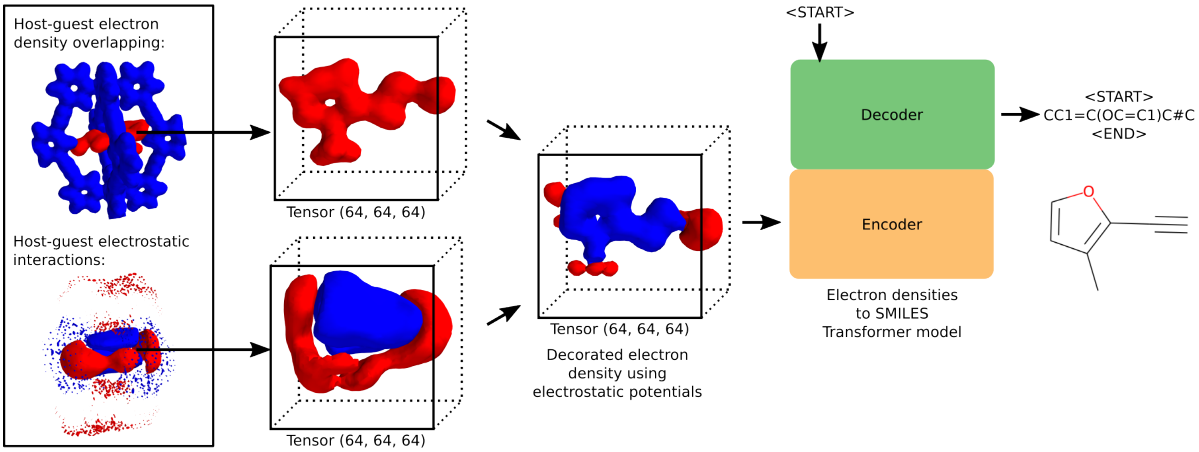

Supplementary Figure 35: Diagram outlining how the Transformer model was used to generated SMILES sequences from 3D electron densities decorated with electrostatic potential information. The encoder received as input the decorated electron densities, while the decoder received as input the “<START>” token. With this information, the Transformer generated the SMILES sequence that best matched the decorated electron density.

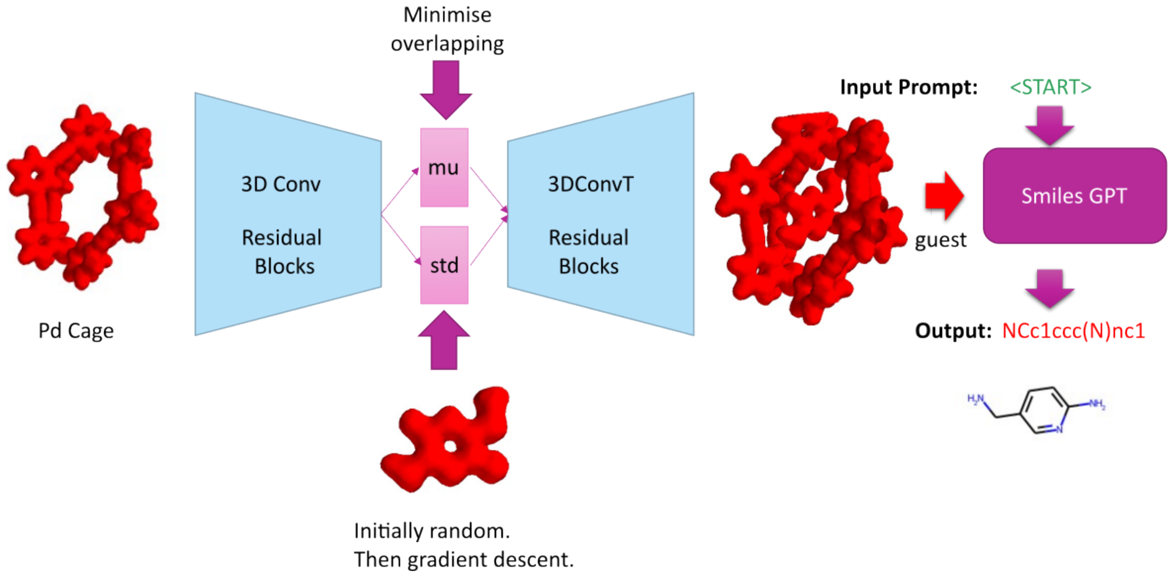

Supplementary Figure 36: Optimisation process aiming to find guests that fitted inside the Pd Cage, using only electron density data. The outputted optimised guests were translated into SMILES sequences using the described Trasnformer model.

2.2.6 bin/optimisers/cage_hg.py

This script focused on running experiments that tried to find guests for the Pd Cage host. In this script the calculations were performed using only electron densities. Given a host and a candidate guest, this script tried to minimise the overlapping between host and guest electron densities, see Supplementary Figure 36.

This script did not introduce any new fitness functions, and the optimisation source code is almost the same to the previous scripts, although this script introduced some new code which mostly focused on improving the output files generated so that it was easier to analyse them later on. We will first discuss the new source code added, and then we will focus on the optimisation process.

The steps performed before the optimisation started were:

1. Line 25: The variable `BATCH_SIZE` was used to define the size of the population of guests.
2. Lines 26-27: The host's electron density was loaded into a variable (see Supplementary Section 2.1.3).
3. Line 28: The VAE model was loaded (see Supplementary Section 2.1.4).
4. Lines 33-37: An initial population of guests (in latent vector form) was randomly generated, see Supplementary Section 2.1.1. We named this population `noise_t`. From the latent vector `noise_t`, the 3D structures were generated and saved into disk.

The optimisation process was divided into two steps: (1) gradient descent was used to increase the size of the molecules as much as possible, (2) gradient descent was used to minimise the overlapping between host and guest. Before, when using the CB6, it

was not required to increase the size of the molecules, because the CB6 host is "small" compared to the average molecule present in the QM9 dataset, thus almost every random molecule generated overlapped with the CB6 host. On the other hand, the Pd Cage is "bigger" than the average QM9 molecule, and therefore a randomly generated molecule would probably fully fit inside, with minimal overlapping. This is why, when working with the Pd Cage, we increased first the size of the molecules, to try and force some degree of overlapping.

The optimisation loop which increased the size of the molecules was executed first, and it did the following steps:

1. Line 40: A for loop started that iterated 5,000 times. This number of iterations was empirically chosen. This number of iterations seemed to make sure that the optimisation was finished. The following instructions are all inside the for loop.

2. Line 41 (inside the for loop): A gradient step was taken to maximise the size of the molecule. This gradient step was performed using the function `grad_size` described in Supplementary Section 2.1.2. This function returned three values: fitness, gradients and outputs (the 3D shapes of the electron densities).

3. Line 42 (inside the for loop): The fitness value just obtained was displayed on screen.

4. Line 43 (inside the for loop): The `noise_t` latent vector was modified using the gradient information obtained before. In particular, this script performed the following manipulation: `noise_t-=0.01*gradient`. The "0.01" was empirically chosen, and it represented a sort of learning rate.

5. Line 44 (inside the for loop): The `noise_t` latent vector was clipped between -5 and +5 using the "clip" function from Numpy. This clipping operation was performed to make sure that the latent vector did not divert towards impossible values. This operation finishes the gradient descent iteration.

6. Lines 46-48 (inside the for loop): The current molecules were saved into disk. As the optimisation progressed, different snapshots of the molecules population were saved into disk.

At this point, `noise_t` contained a list of molecules (their latent vectors) with maximised sizes. The next part of the optimisation process tried to minimise the overlapping of these molecules with the Pd Cage host. The optimisation loop was implemented in the same way as in the previous supplementary section, were the learning rate decreased every (in this case) 8,000 iterations, exactly as we did before. The actual gradient optimisation process was the same as in Supplementary Section 2.2.1: the function `grad_ed_overlapping` was used to calculate the gradients related to the host/guest overlapping, and the latent vectors in `noise_t` were modified using these gradients.

This process is outlined in Supplementary Figure 37. The electron densities obtained can be transformed into SMILES using a very similar process to the one depicted in Supplementary Figure 35, but using only the electron density data.

The results obtained using this script can be seen in Supplementary Section 2.3.6.

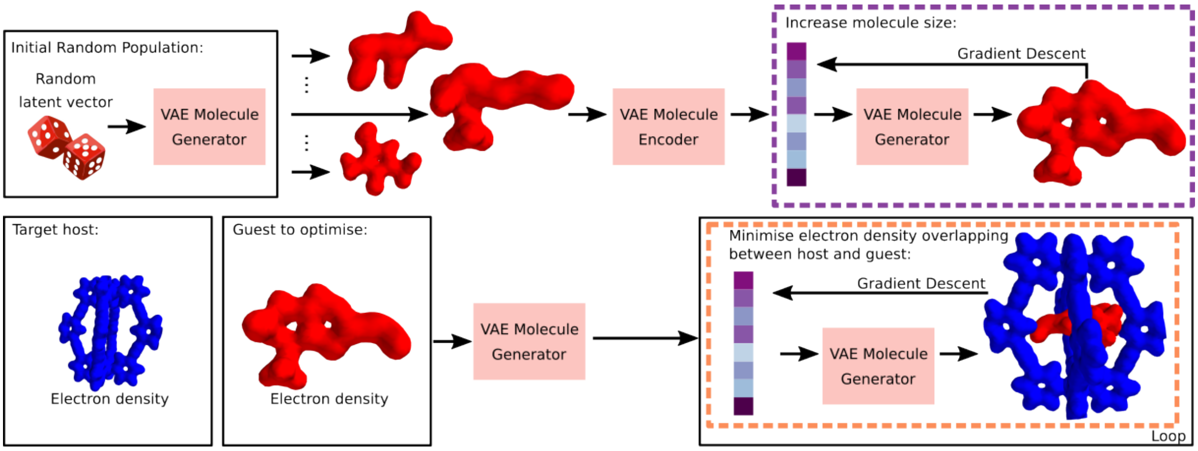

Supplementary Figure 37: Diagram outlining the optimisation process used to find guests for the Pd Cage host by minimising the host/guest electron densities overlapping. First, an initial random population of guests was generated. Then, the overlapping between these guests and the target host was calculated. Finally, the guests were modified through gradient descent to minimise this overlapping.

2.2.7 bin/optimisers/cage_hg_esp.py

This script focused on running experiments that tried to find guests for the Pd Cage host. In this script the calculations were performed using both electron densities and electrostatic potentials. Given a host and a candidate guest, this script tried to minimise the overlapping between electron densities, and it tried to maximise the electrostatic interactions. See Supplementary Figure 38 for an outline of this process.

This script is very similar to the script discussed in Supplementary Section 2.2.5. Here we will only highlight the differences:

- The host loaded was the Pd Cage instead of CB6.
- Before performing the electron densities and electrostatic potentials optimisations, a gradient descent loop was used to increase the size of the molecules from the initial population. This loop was very similar to the one described in the previous supplementary section, were the size of the molecules was also increased. In this case, the loop iterated 1,000 times, instead of 5,000.
- The learning rate was calculated with `lr=0.01/factor` instead of `lr=0.05/factor`.
- No clipping was performed to `noise_t` after it had been updated with the new gradients.

The full optimisation process is outlined in Supplementary Figure 39. The electron densities obtained can be transformed into SMILES using the process depicted in Supplementary Figure 35.

The results obtained using this script can be seen in Supplementary Section 2.3.7.

2.2.8 bin/optimisers/cage_hg_esp_lee.py

This script focused on running experiments that tried to find guests for the Pd Cage host. In this script the calculations were performed using both electron densities and

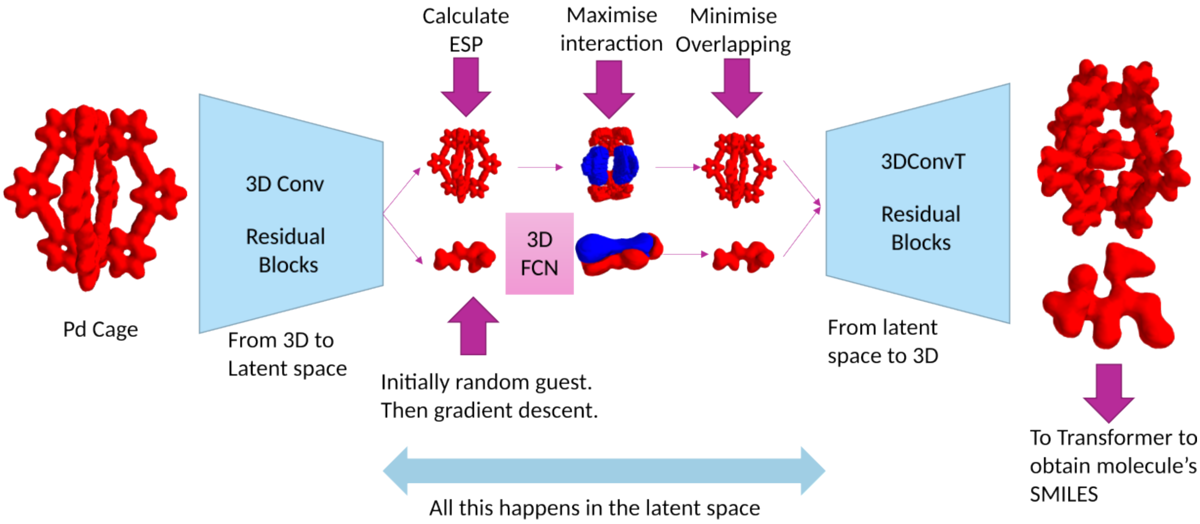

Supplementary Figure 38: Outline displaying the steps taken during the optimisation process: First a host was selected, then a random population of guests were generated, and gradient descent was used to fit them inside the host while maximising the electrostatic interactions between host and guess.

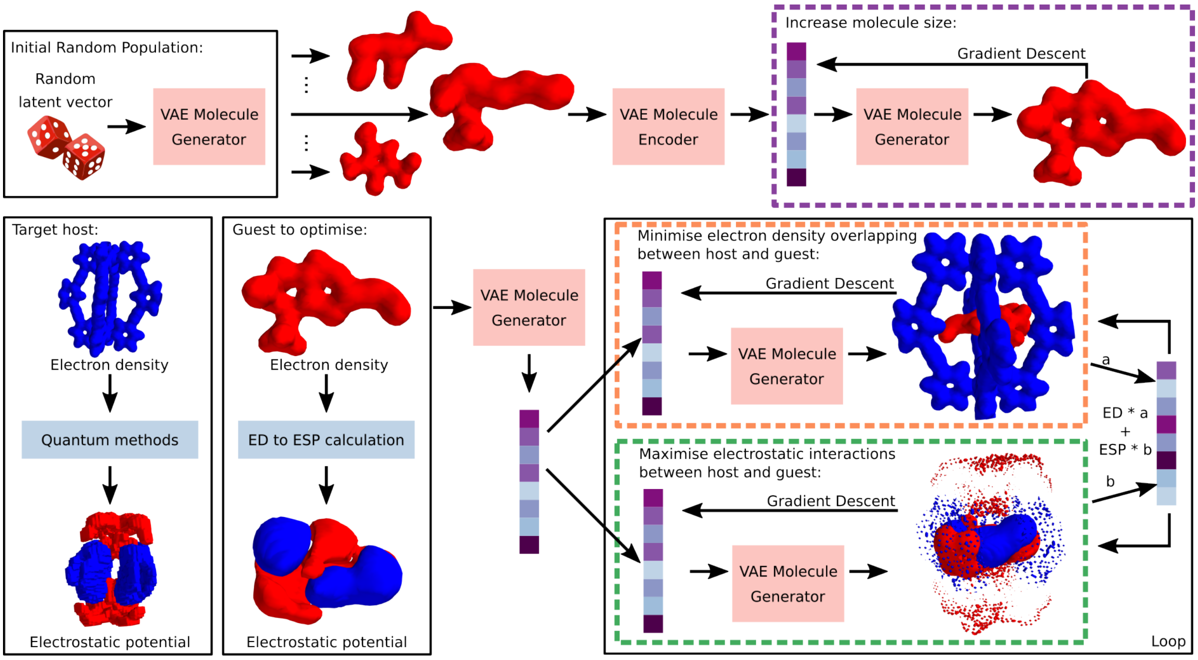

Supplementary Figure 39: Diagram outlining the optimisation process used to find guests for the PD Cage host, using both electron densities and electrostatic potentials. First, an initial random population of guests was generated. Then, the overlapping between these guests and the target host was calculated. After this, the electrostatic interactions between the guests and the host were calculated. Finally, the guests were modified through gradient descent to minimise the overlapping of electron densities, while maximising the electrostatic interactions.

electrostatic potentials. Given a host and a candidate guest, this script tried to minimise the overlapping between electron densities, and it tried to maximise the electrostatic interactions. Thus, the guests were manipulated to achieve this.

This script is very similar to the scripts discussed in Supplementary Section 2.2.5 and Supplementary Section 2.2.7. The main difference is that before, inside the optimisation loop, the latent vector was modified twice: once based on the gradients derived from the electron densities overlapping, and once derived from the electrostatic interactions. In this script, a new fitness function was created, named `combined_ed_esp`, which calculated the gradients for both fitness functions (electron density overlapping and electrostatic interactions), then combined both fitness values, and then modified the latent vector. Therefore, while before the latent vectors were modified twice, in this script they were only modified once.

This new function, as discussed, was named `combined_ed_esp` and had the following input parameters:

1. `latent_vector` referred to a tensor of size (`batch_size`, `z_dim`) which represented the VAE latent vectors. This latent vector must refer to electron densities. Before, this input parameter was named `noise`.

2. `vae` referred to a loaded VAE model. The latent vector of this VAE must be of size `z_dim`. This VAE must had been trained on electron densities.

3. `ed2esp` referred to a loaded machine learning model to calculate electrostatic potentials from electron densities. This was implemented using a FCN as detailed in Supplementary Section 1.6.

4. `hosted` referred to a tensor describing the target host's electron density.

5. `hostesp` referred to a tensor describing the target host's electrostatic potential.

6. `ed_factor` referred to float value which indicated how both fitness functions were combined. It had values between 0 and 1.

This new fitness function executed the following steps:

1. Line 40: The gradient of the input latent vector against was calculated using `grad_esp_overlapping`, which calculated the electrostatic interactions, see 2.1.2. It returned four values: fitness, esp_gradients, 3D electron densities, and 3D electrostatic potentials.

2. Line 41: The fitness calculated was printed.

3. line 44: The gradient of the input latent vector was calculated using `grad_ed_overlapping`, which calculated the overlapping between electron densities, see 2.1.2. It returned three values: fitness, ed_gradients, and 3D electron densities.

4. Line 45: The fitness calculated was printed.

5. Line 48: The gradients were combined using the following formula: `gradients=ed_gradients*ed_factor+esp_gradients*(1-ed_factor)`.

6. Line 50: The following values were returned: fitness, gradients, 3D electron densities, 3D electrostatic potentials.

The optimisation process was very similar to the one described in Supplementary Section 2.2.5, with a decreasing learning rate, but now using only one fitness function: `combined_ed_esp` as just described.

The main difference compared with the script described in Supplementary Section 2.2.5, is that while before the script ran once with a user set `ed_factor`, now the optimisation process was repeated a number of times, screening different values for `ed_factor` between 0 and 1.

The full optimisation process is outlined in Supplementary Figure 39. The electron densities obtained can be transformed into SMILES using the process depicted in Supplementary Figure 35.

The results obtained using this script can be seen in Supplementary Section 2.3.8.

2.2.9 bin/optimisers/cage_hg_split_esp.py

This script focused on running experiments that tried to find guests for the Pd Cage host. In this script the calculations were performed using both electron densities and electrostatic potentials. Given a host and a candidate guest, this script tried to minimise the overlapping between electron densities, and it tried to maximise the electrostatic interactions. Thus, the guests were manipulated to achieve this.

This script is very similar to the scripts discussed in Supplementary Section 2.2.5 and Supplementary Section 2.2.7. The main difference is that in the previous optimisation experiments which aimed to maximise the electrostatic interactions between host and guest, we used the fitness function `grad_esp_overlapping`, explained in Supplementary Section 2.1.2, which multiplied the electrostatic potentials from the host with the ones from the guest, and then summed the resulting 3D tensor together to into a single value. On the other hand, in this script the host's electrostatic potentials were split into their positive or negative parts, and the host/guest interactions were calculated individually with each of them. Then, they were combined together using a weight factor to decided if more weight was given to the positive or negative parts. The main reasoning behind this is that in the case of the Pd Cage, the palladium atoms are critical to find good guests. Therefore, instead of considering the whole host's electrostatic potentials in equal terms, we wanted the optimisation processes to focus more on the palladium atoms, but also to still consider everything else.

To achieve this, two new functions were introduced, being the first one `split_host_esp`. This received as input a tensor representing the electrostatic potentials from the host, and returned two different tensors: one that only contained the positive values, and another that only contained the negative ones, see Supplementary Figure 40.

The second new function is a new fitness function named `combined_ed_esp`. This function had eight input parameters. Five of them: latent_vector, vae, ed2esp, hosted and ed_factor, were exactly the same as the fitness function introduced in the previous section (Supplementary Section 2.2.8). We will now discuss the three new input parameters:

- `hostesp_pos` referred to a tensor describing the positive values within the target host's electrostatic potential.
- `hostesp_neg` referred to a tensor describing the negative values within the target host's electrostatic potential.

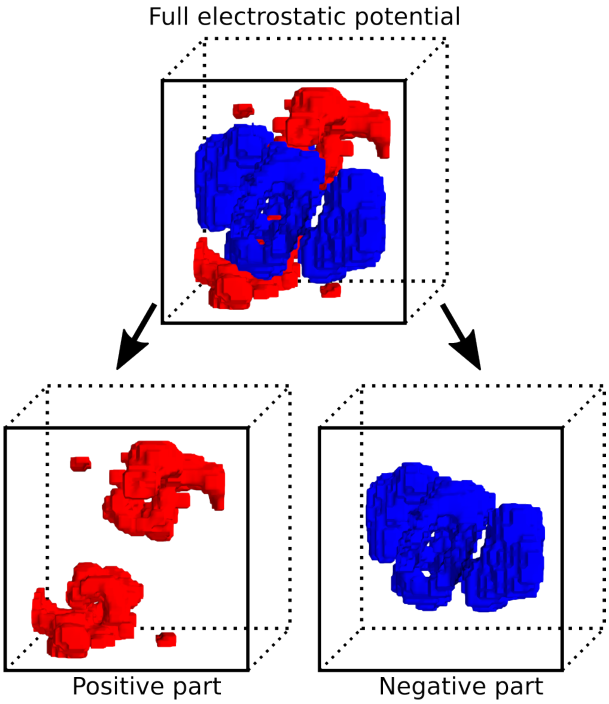

Supplementary Figure 40: Splitting the electrostatic potential from the Pd Cage into its positive and negative parts. This was achieved by creating two new tensors: one that only stored the positive parts of the original electrostatic potential, and one that only stored the negative parts.

- `esp_pos_factor` referred to float value which indicated how the electrostatic potential calculations between the positive and negative parts of the host, and the guests, were combined. It had values between 0 and 1.

This new fitness function executed the following steps:

1. Line 46: Calculated the fitness and gradients between the input latent vector and `hostesp_pos` using `grad_esp_overlapping`. We named the fitness `fp` and the gradients `esp_grads_p`.

2. Line 47: Calculated the fitness and gradients between the input latent vector and `hostesp_neg` using `grad_esp_overlapping`. We named the fitness `fn` and the gradients `esp_grads_n`..

3. Line 48: The gradients were combined using the following formula: `esp_grads=esp_grads_p*esp_pos_factor+esp_grads_n*(1-esp_post_factor)`.

4. Line 50: The fitnesses were combined using the following formula: `combined_fitness=fp+fn`.

5. Line 54: The gradient of the input latent vector against the the overlapping between electron densities was calculated using `grad_ed_overlapping`, see 2.1.2. It returned three values: fitness, ed_gradients, and 3D electron densities.

6. Line 58: The gradients were combined using the following formula: `gradients=ed_gradients*ed_factor+esp_gradients*(1-ed_factor)`.

7. Line 60: The following values were returned: fitness, gradients, 3D electron densities, 3D electrostatic potentials.

The actual optimisation process was very similar to the ones described previously, in particular to the one described in the previous section (Supplementary Section 2.2.8). The main difference with respect to the previous script, is that while that one screened a range of values for `ed_factor`, in this script `ed_factor` was fixed, and instead a range of values for `esp_pos_factor` was screened.

The full optimisation process is very similar to the one outlined in Supplementary Figure 39. In a similar way to how in this figure it is shown how gradients calculated from the electron density overlapping were combined with gradients calculated from the electrostatic potential interactions, in this script gradients calculated from electrostatic potential interactions of the positive part of the host with the guests were also combined with gradients calculated from electrostatic potential interactions of the negative part of the host with the guests The electron densities obtained can be transformed into SMILES using the process depicted in Supplementary Figure 35.

This method did not improve the results obtained previously, and thus results from this method are not present in the main manuscript.

The results obtained using this script can be seen in Supplementary Section 2.3.9.

Supplementary Subsection 2.3 Optimisation results

The following sections show the results obtained after executing the scripts described in Section 2.2. These scripts generated a population of guests that minimised or maximised the different fitness functions. These guests were outputted as 3D electron densities (or electrostatic potentials). Using the Transformer model described in Section 1.11, we translated these 3D structures into SMILES sequences, and then using RDkit we calculated the 2D structure to draw the molecule as can be seen in the different figures. Thus, every figure contains a number of molecules, where each molecule is represented by its SMILES sequence and its 2D structure. In some of the later results, it can be seen that besides the SMILES sequence there is a "Y" or "N" letter, indicating if the molecule is commercially available or not, and besides this letter a number, which indicates the synthetic accessibility score as calculated using RDKit.

Before showing the results, it is important to remember that the Transformer model generated the SMILES sequences token by token, and that the next token could be chosen in a "greedy" way (directly picking the one with maximum probability) or using "probabilistic sampling". These two options were discussed in Section 1.10.2. Unless the opposite is said, all the results shown followed the "greedy" approach.

Finally, as discussed in Section 1.11, the Transformer model took as input electron densities, electrostatic potentials, or decorated electron densities (see Section 1.8). Unless the opposite is said, all the results were calculated using electron densities. If another type of input was used, this will be specified in the figure captions.

2.3.1 Results from bin/optimisers/host_guest_overlapping.py

These results were obtained using the `host_guest_overlapping.py` script described in Section 2.2.1. The results can be seen in Supplementary Figures 41 to 44.

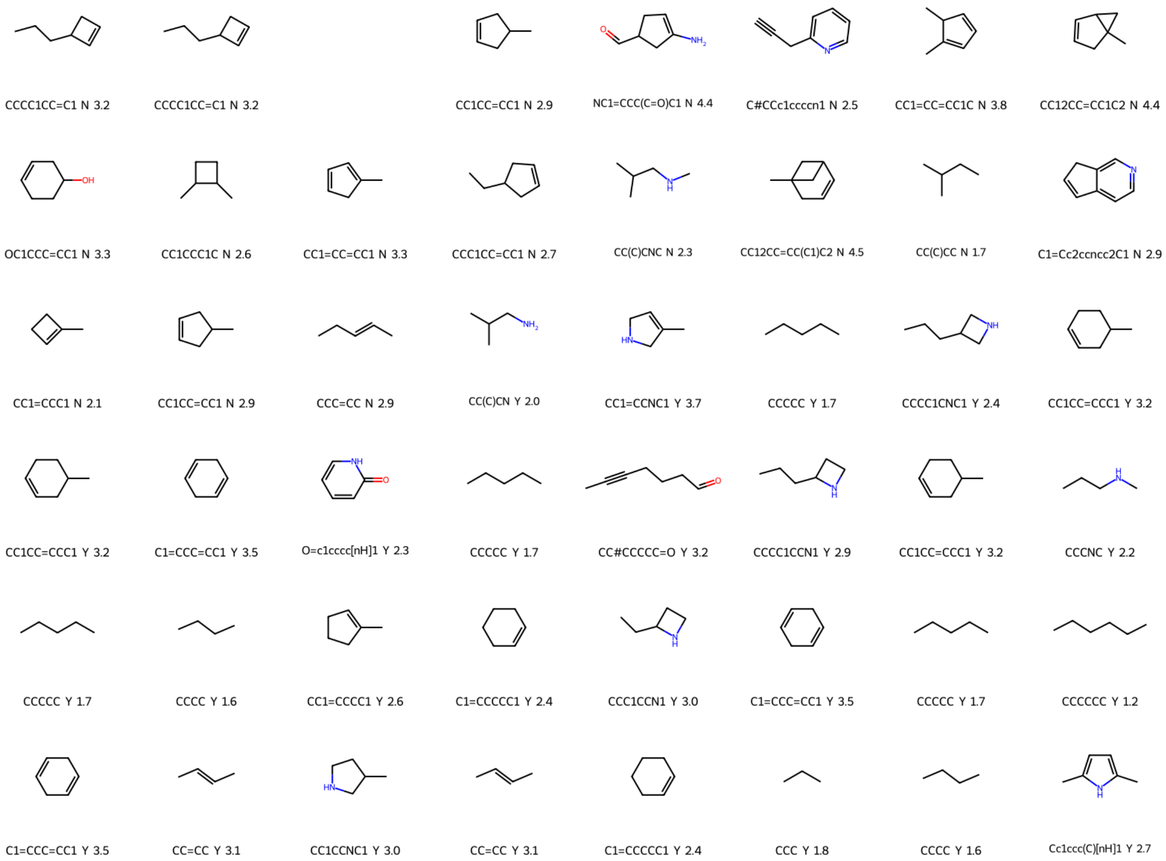

Supplementary Figure 41: Molecules obtained when trying to minimise the overlapping between CB6 host and guest electron densities. The optimisation routine only worked using electron densities. The final ones were converted into SMILES using the previously described Transformer model. The 2D images of molecules displayed in this image where obtained using RDkit.

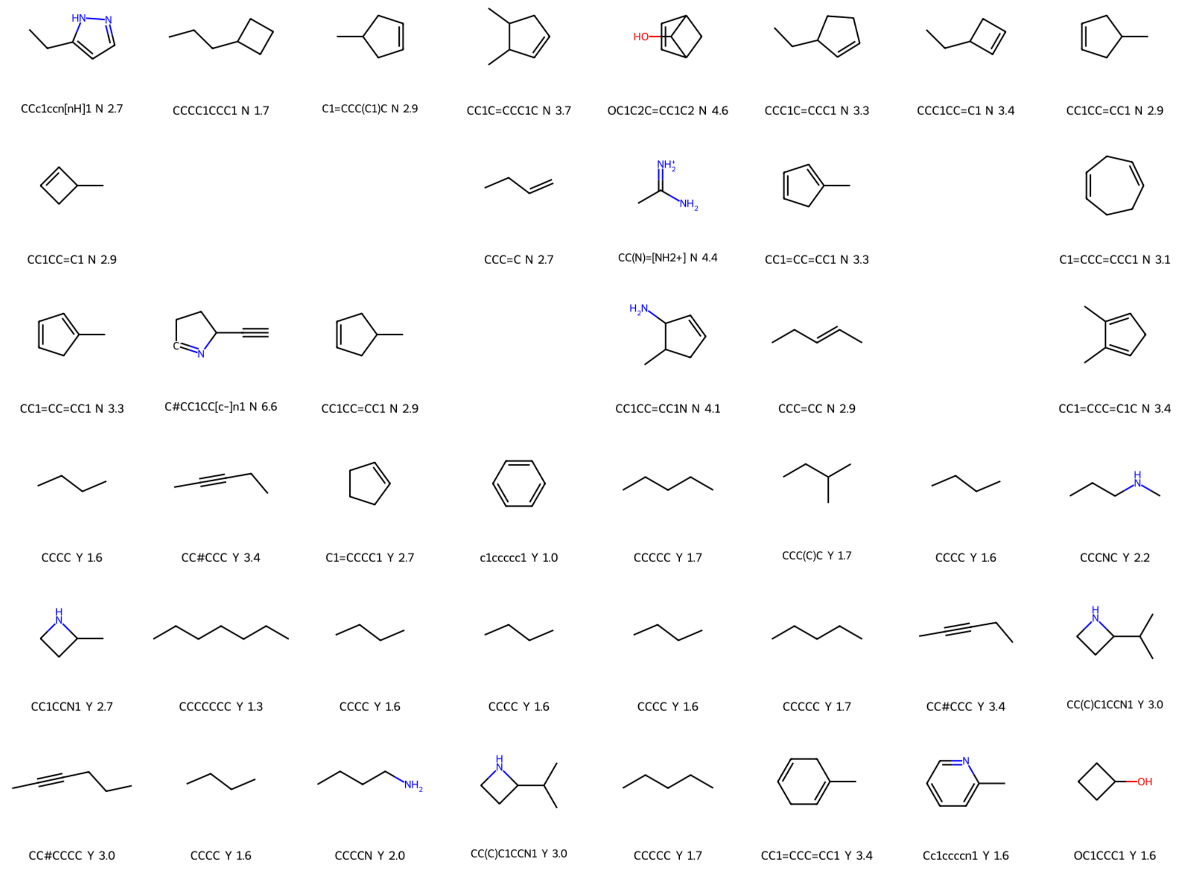

Supplementary Figure 42: Molecules obtained when trying to minimise the overlapping between CB6 host and guest electron densities. These molecules were obtained using the probabilistic sampling approach. The optimisation routine only worked using electron densities. The final ones were converted into SMILES using the previously described Transformer model. The 2D images of molecules displayed in this image where obtained using RDkit.

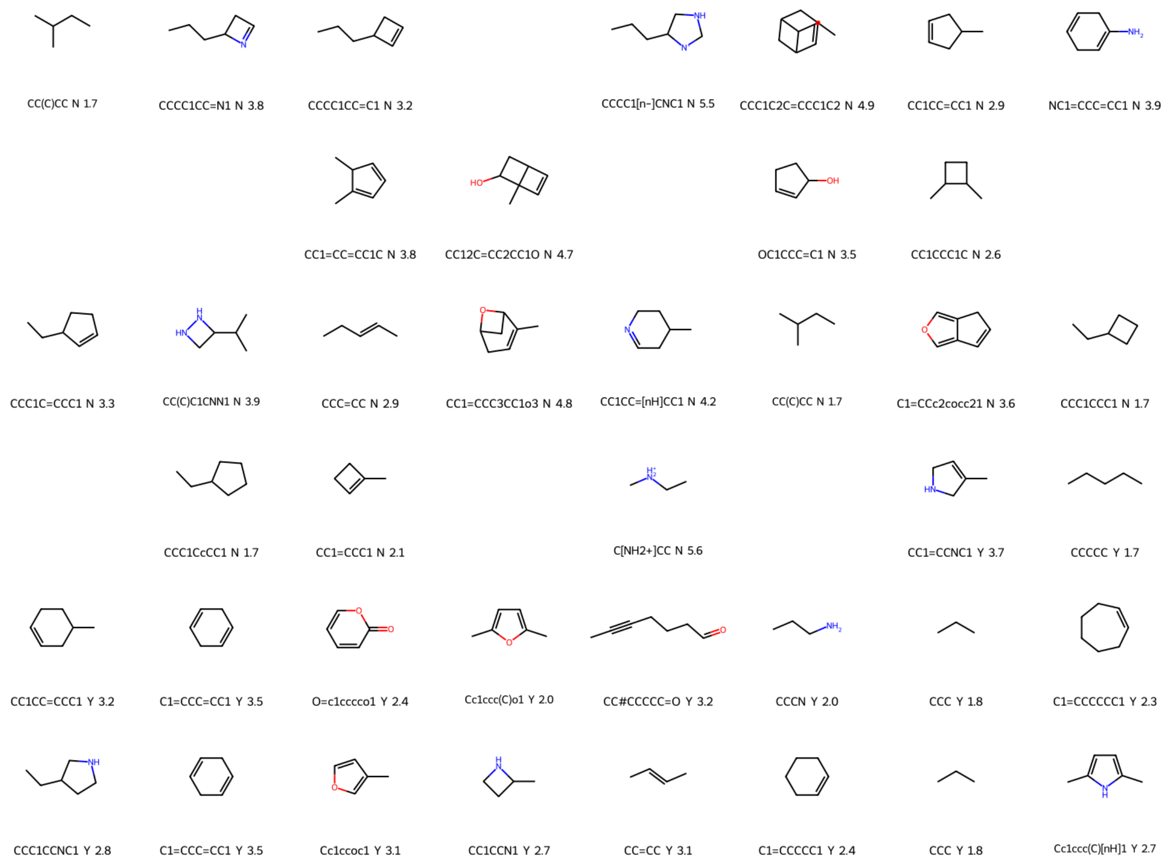

Supplementary Figure 43: Molecules obtained when trying to minimise the overlapping between CB6 host and guest electron densities. These molecules were obtained using the probabilistic sampling approach. The optimisation routine only worked using electron densities. The final ones were converted into SMILES using the previously described Transformer model. The 2D images of molecules displayed in this image where obtained using RDkit.

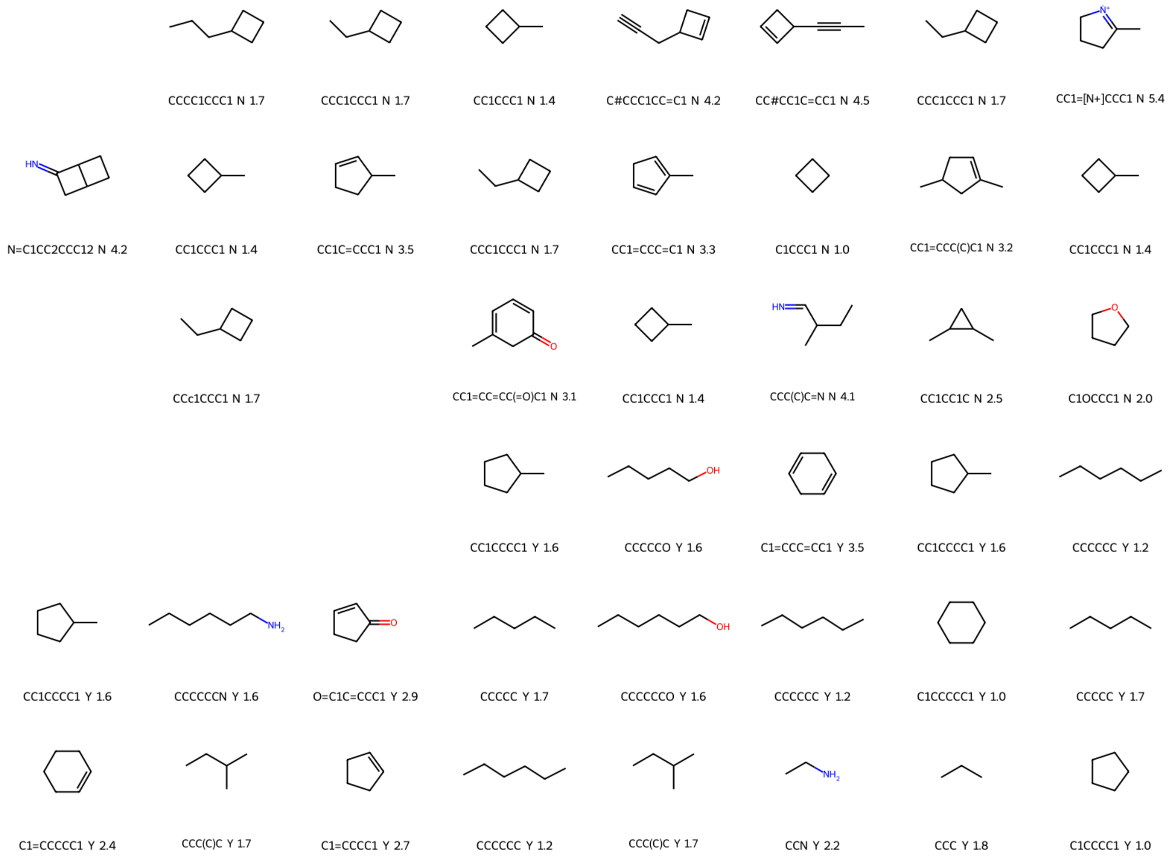

Supplementary Figure 44: Molecules obtained when trying to minimise the overlapping between CB6 host and guest electron densities. These molecules were obtained using the probabilistic sampling approach. The optimisation routine only worked using electron densities. The final ones were converted into SMILES using the previously described Transformer model. The 2D images of molecules displayed in this image where obtained using RDkit.

2.3.2 Results from bin/optimisers/maximise_size.py

These results were obtained using the `maximise_size.py` script described in Section 2.2.2. The results can be seen in Supplementary Figure 45.

2.3.3 Results from bin/optimisers/maximise_polar.py

These results were obtained using the `maximise_polar.py` script described in Section 2.2.3. The results can be seen in Supplementary Figures 46 and 47.

2.3.4 Results from bin/optimisers/maximise_hg_polar.py

These results were obtained using the `maximise_hg_polar.py` script described in Section 2.2.4. The results can be seen in Supplementary Figures 48 to 51.

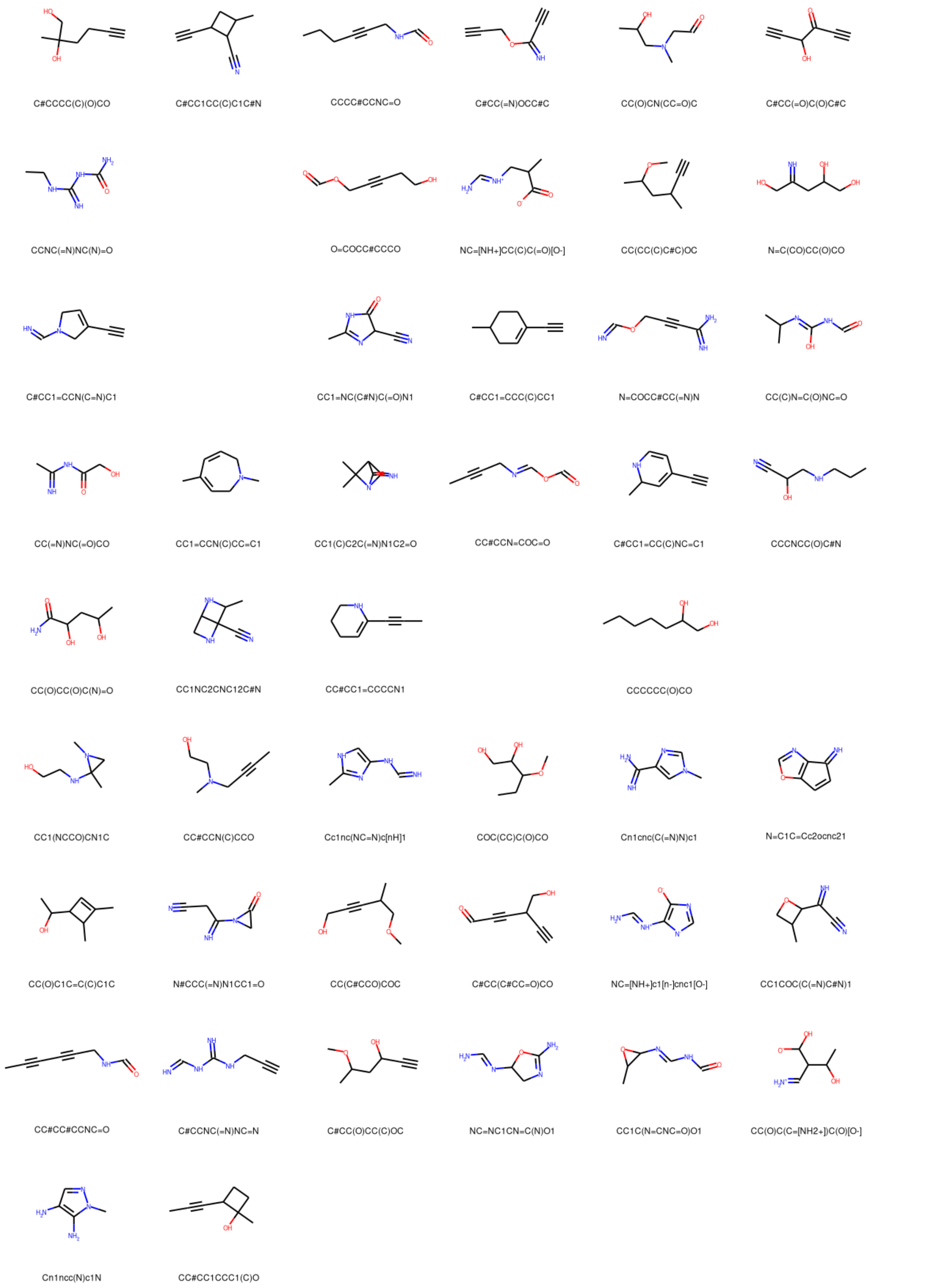

Supplementary Figure 45: Molecules obtained when trying to maximise the size of their electron densities. The optimisation routine only worked using electron densities. The final ones were converted into SMILES using the previously described Transformer model. The 2D images of molecules displayed in this image where obtained using RDkit.

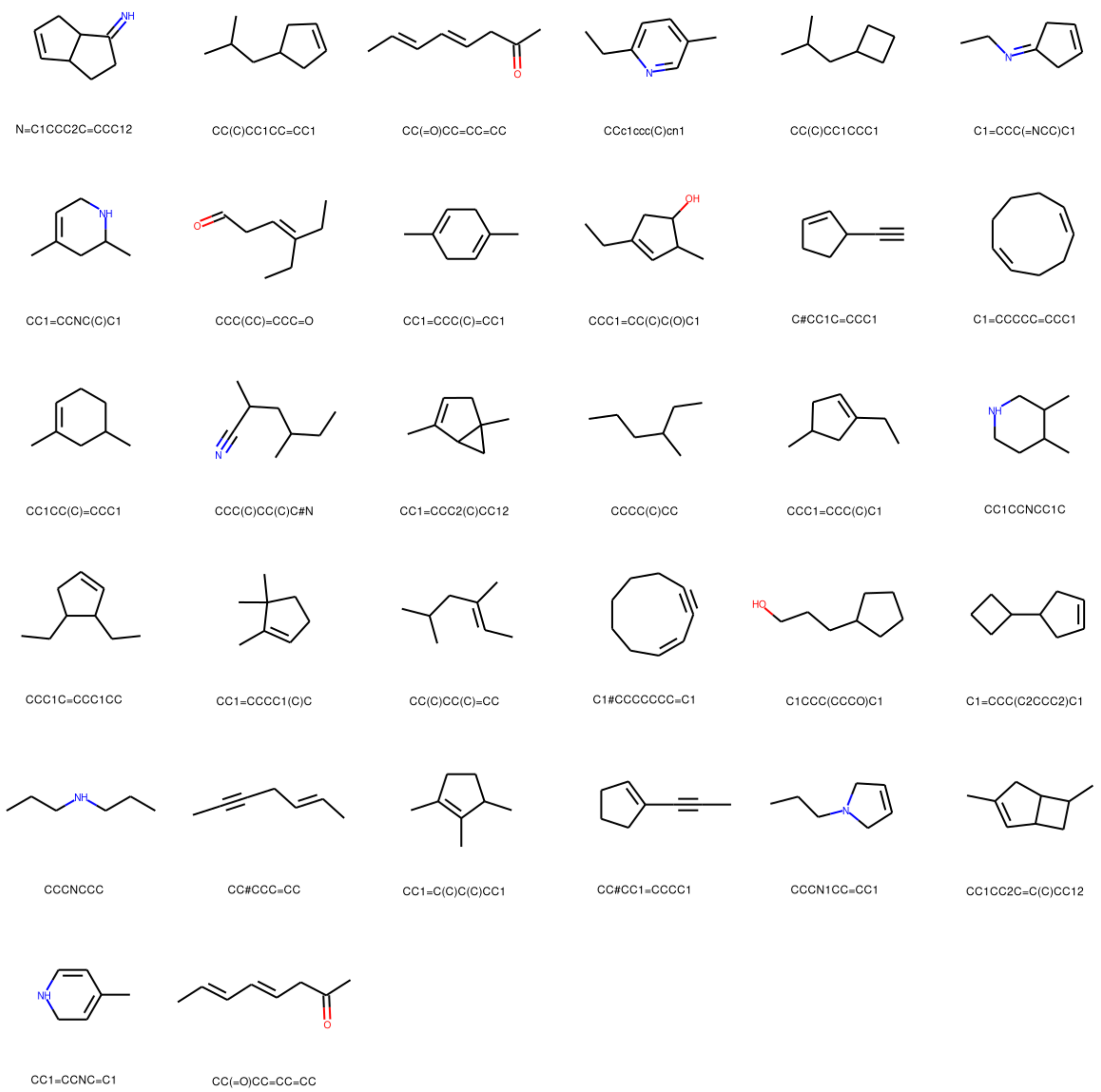

Supplementary Figure 46: Molecules obtained when trying to maximise their isotropic polarisability. The optimisation routine only worked using electron densities. The final ones were converted into SMILES using the previously described Transformer model. The 2D images of molecules displayed in this image where obtained using RDkit.

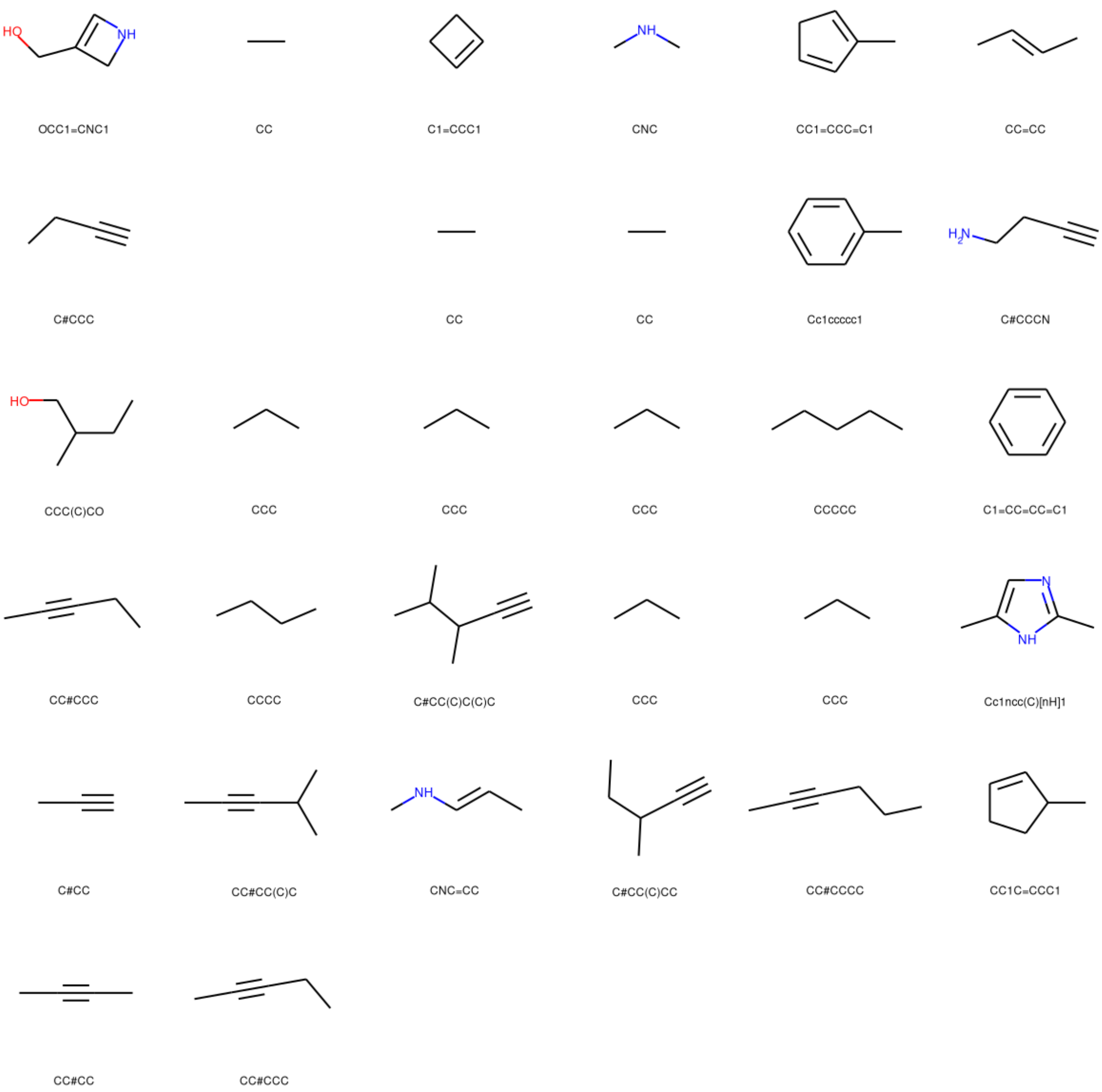

Supplementary Figure 47: Molecules obtained when trying to minimise their isotropic polarisability. The optimisation routine only worked using electron densities. The final ones were converted into SMILES using the previously described Transformer model. The 2D images of molecules displayed in this image where obtained using RDkit.

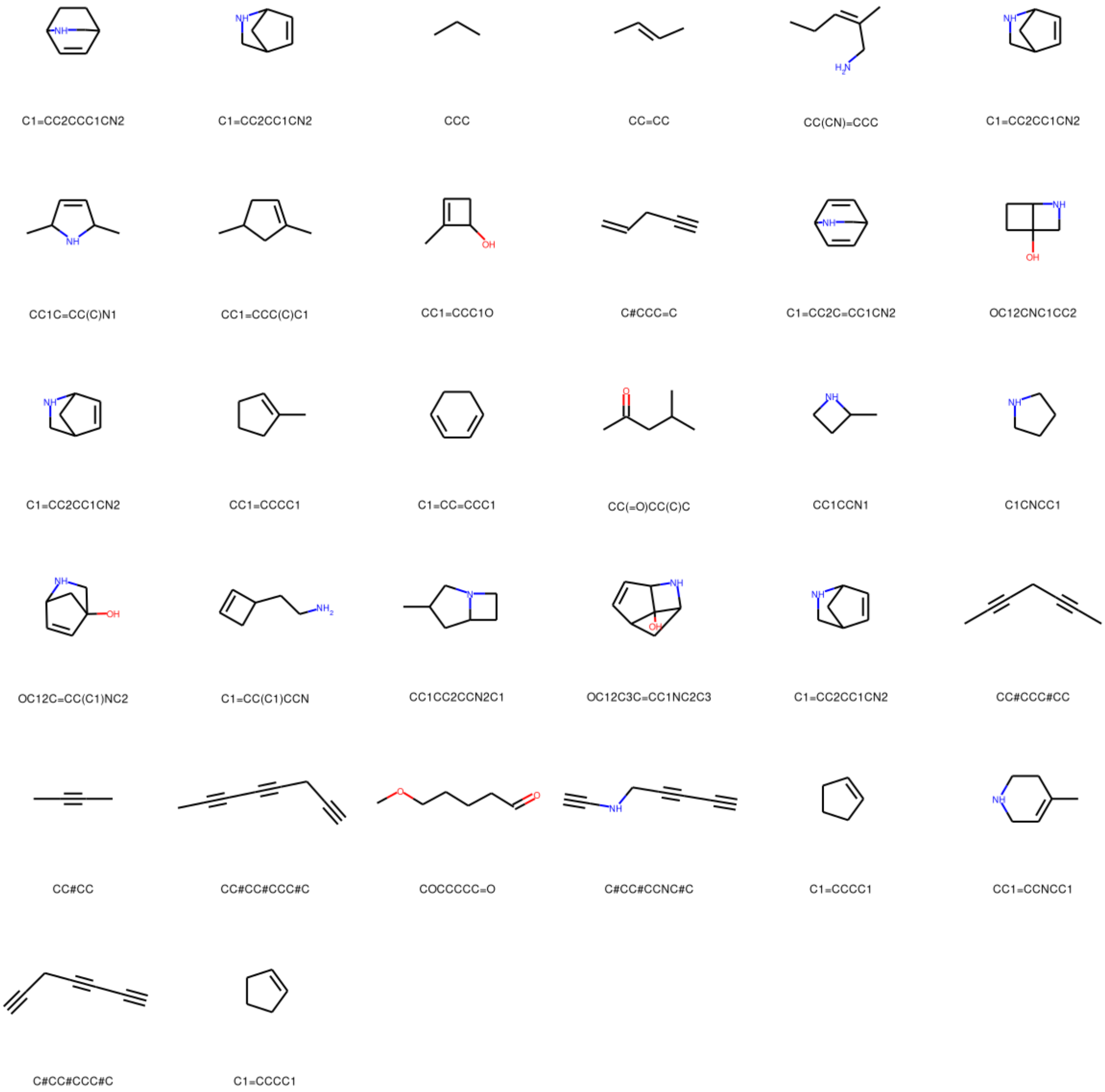

Supplementary Figure 48: Molecules obtained when trying to minimise the overlapping between host (CB6) and guest electron densities, while maximising the isotropic polarisability from the guests. The optimisation routine only worked using electron densities. The final ones were converted into SMILES using the previously described Transformer model. The 2D images of molecules displayed in this image where obtained using RDkit.

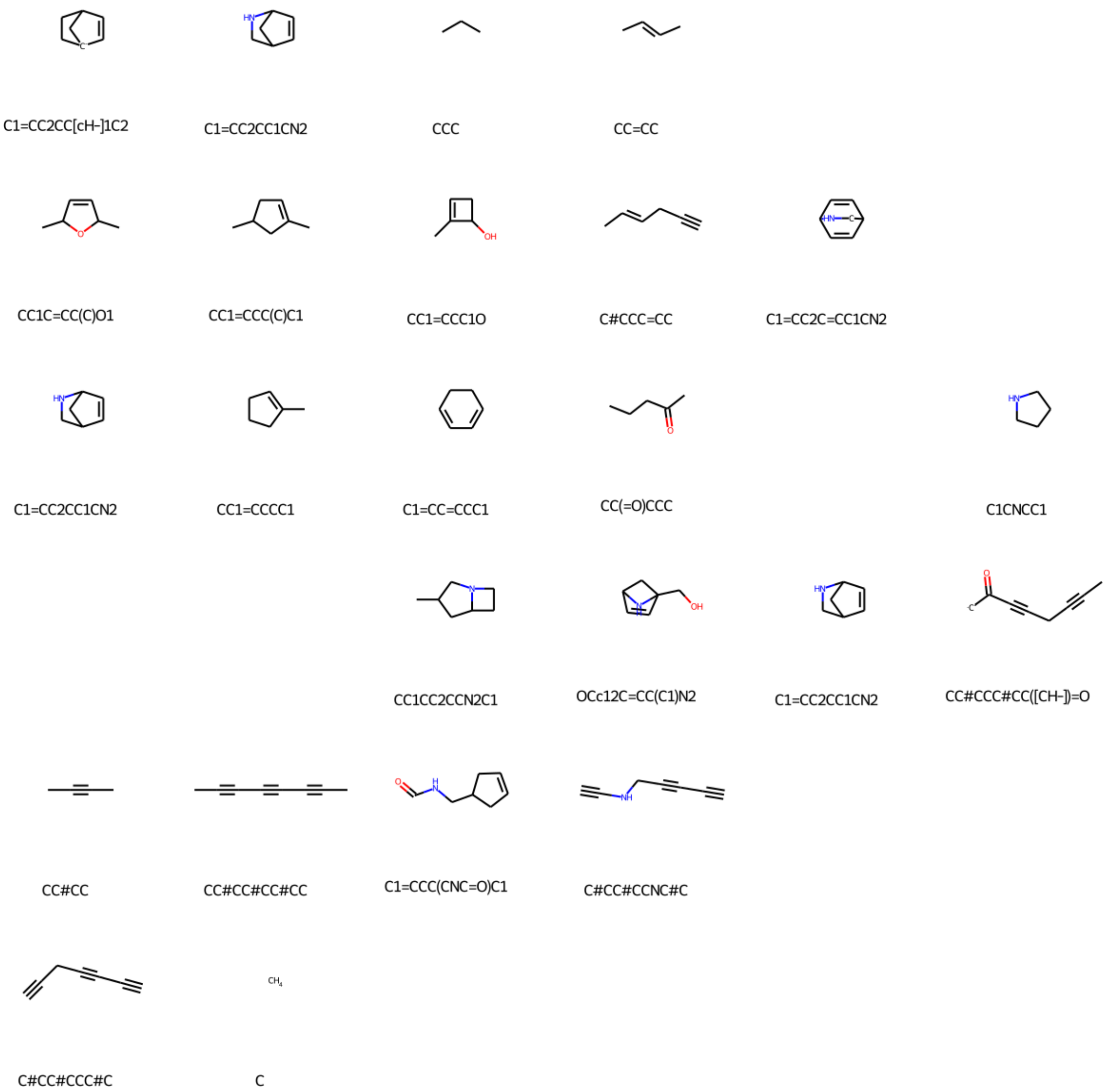

Supplementary Figure 49: Molecules obtained when trying to minimise the overlapping between host (CB6) and guest electron densities, while maximising the isotropic polarisability from the guests. The optimisation routine only worked using electron densities. The final ones were converted into SMILES using the previously described Transformer model. The 2D images of molecules displayed in this image where obtained using RDkit.

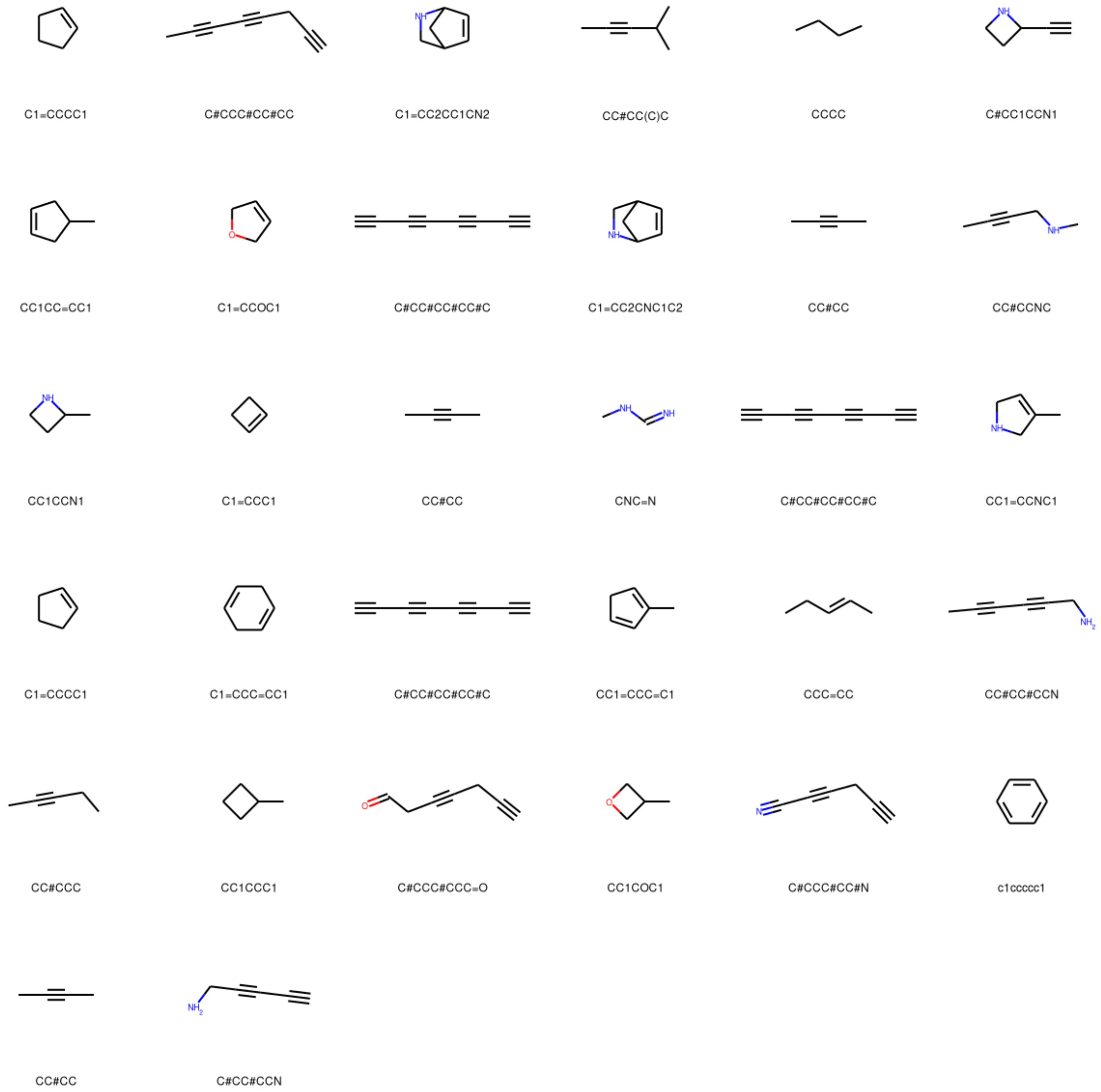

Supplementary Figure 50: Molecules obtained when trying to minimise the overlapping between host (CB6) and guest electron densities, while maximising the isotropic polarisability from the guests. The optimisation routine only worked using electron densities. The final ones were converted into SMILES using the previously described Transformer model. The 2D images of molecules displayed in this image where obtained using RDkit.

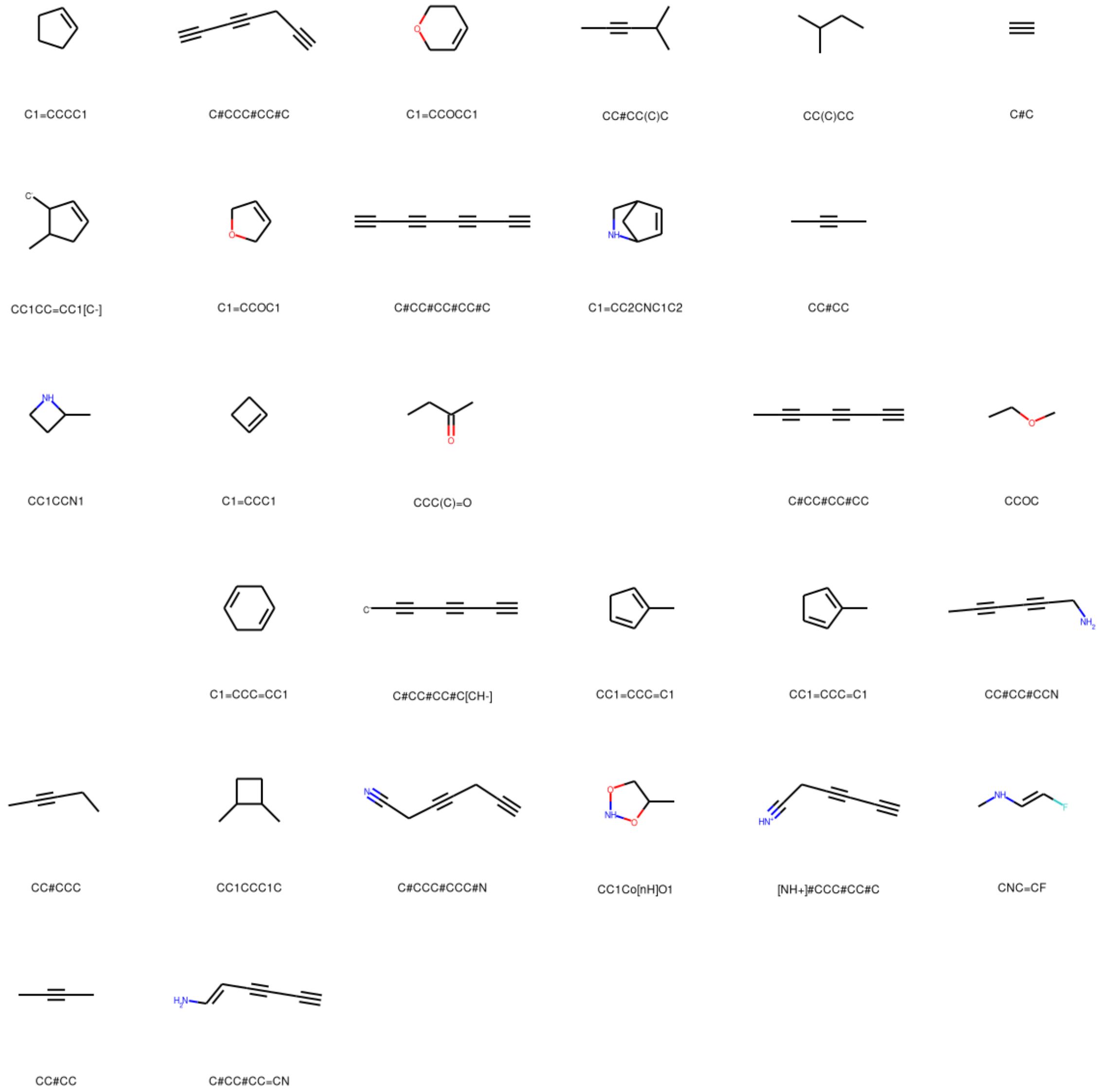

Supplementary Figure 51: Molecules obtained when trying to minimise the overlapping between host (CB6) and guest electron densities, while maximising the isotropic polarisability from the guests. The optimisation routine only worked using electron densities. The final ones were converted into SMILES using the previously described Transformer model. The 2D images of molecules displayed in this image where obtained using RDkit.

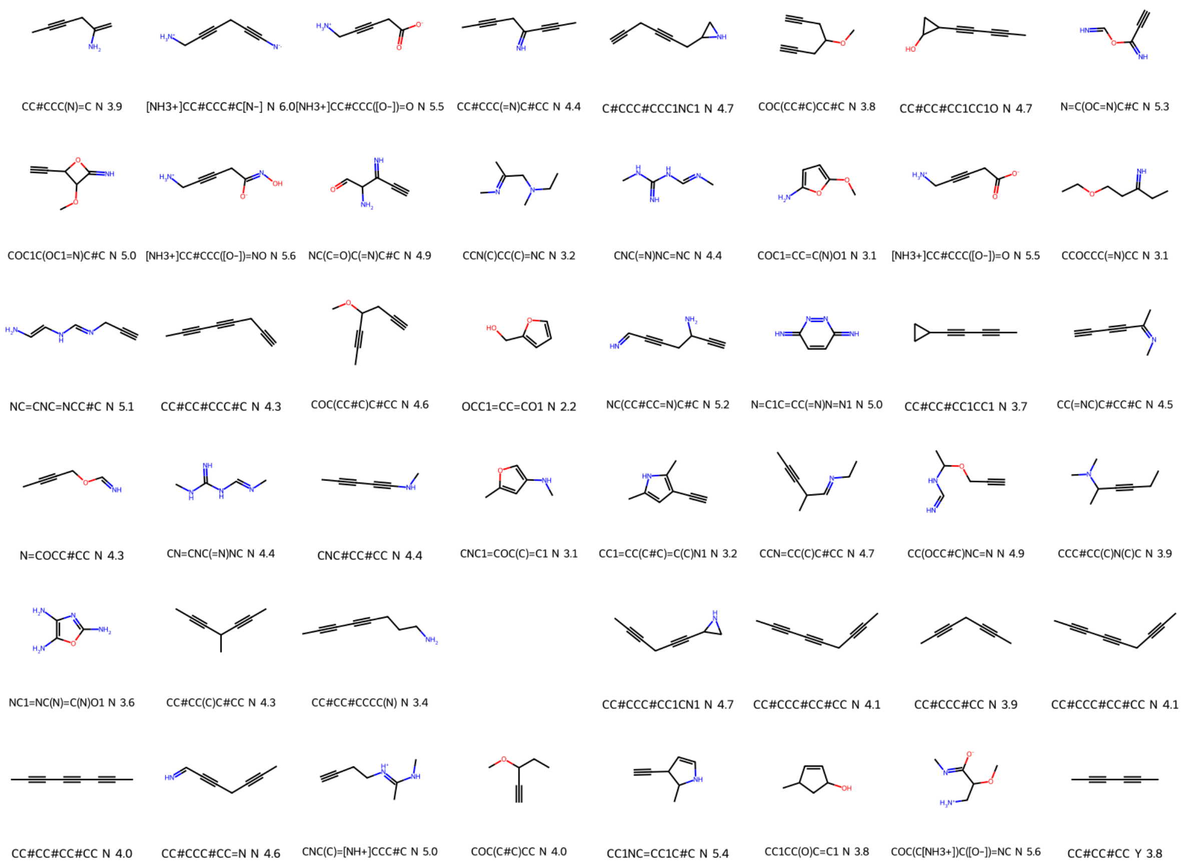

Supplementary Figure 52: Molecules obtained when trying to minimise the overlapping between host (CB6) and guest electron densities, while maximising their electrostatic interactions. $ed\_factor = 0$. The optimisation routine only worked using electron densities. The final ones were converted into SMILES using the previously described Transformer model. The 2D images of molecules displayed in this image where obtained using RDkit.

2.3.5 Results from bin/optimisers/maximise_hg_esp.py

These results were obtained using the `maximise_hg_esp.py` script described in Section 2.2.5. A total of 20 experiments/runs were done using this script using different values for `ed_factor`. This generated 960 different guests. These were converted into SMILES sequences using the two methods based on electron density data described in Section 1.11, plus the method using decorated electron densities described in Section 1.8. Therefore, each 3D tensor representing a guest could output three slightly different molecules. Thus, the total number of molecules generated was 2880. Since this is a very big number, we will only show some of there here. Unless mentioned, the molecules shown were obtained using the method using decorated electron densities. The results can be seen in Supplementary Figures 52 to 71.

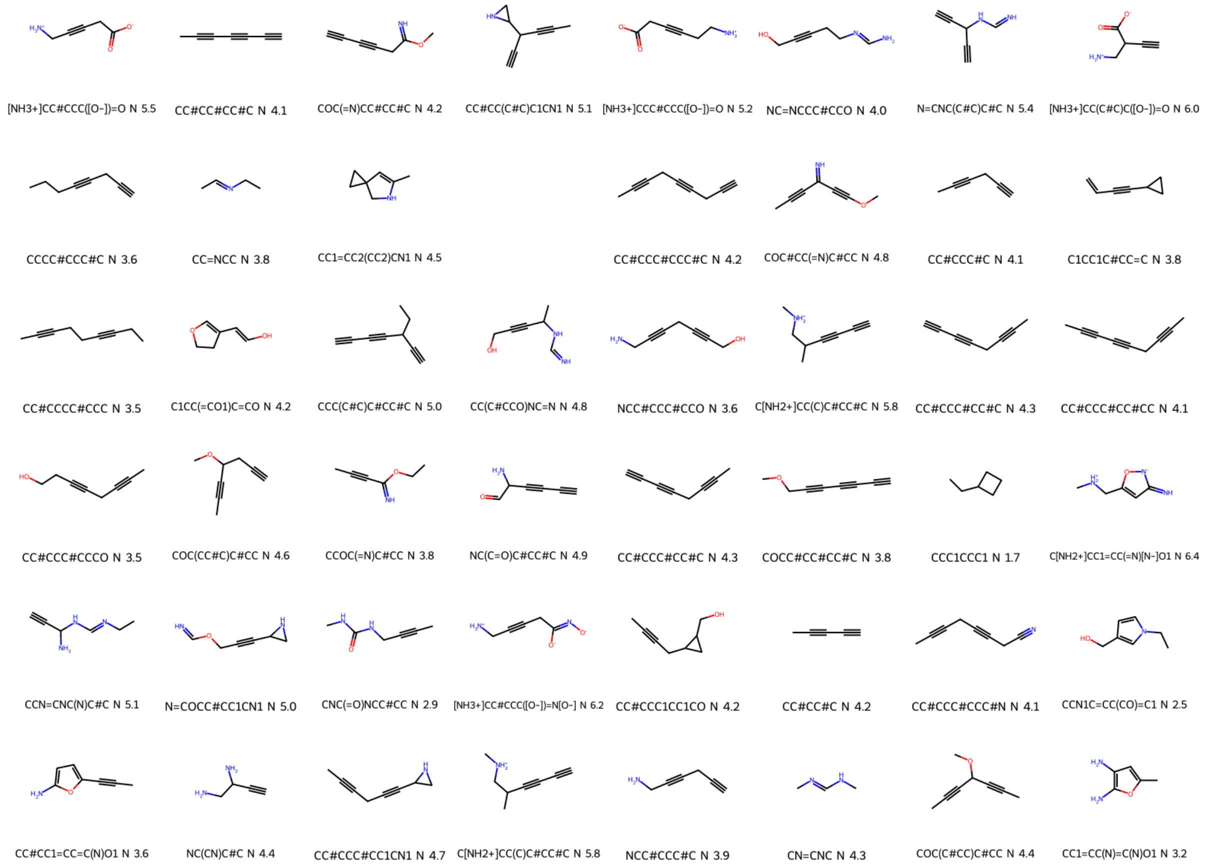

Supplementary Figure 53: Molecules obtained when trying to minimise the overlapping between host (CB6) and guest electron densities, while maximising their electrostatic interactions. *ed_factor* = 0.05. The optimisation routine only worked using electron densities. The final ones were converted into SMILES using the previously described Transformer model. The 2D images of molecules displayed in this image where obtained using RDkit.

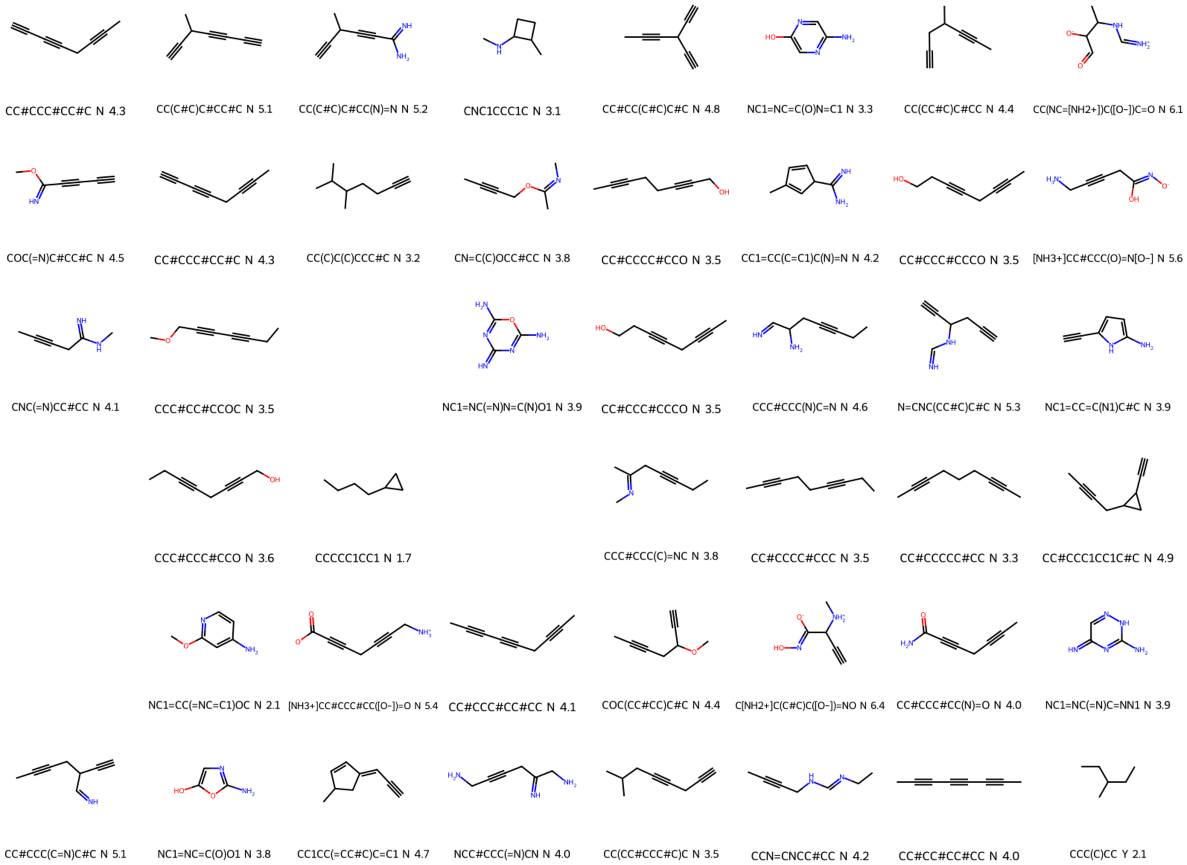

Supplementary Figure 54: Molecules obtained when trying to minimise the overlapping between host (CB6) and guest electron densities, while maximising their electrostatic interactions. $ed\_factor = 0.1$

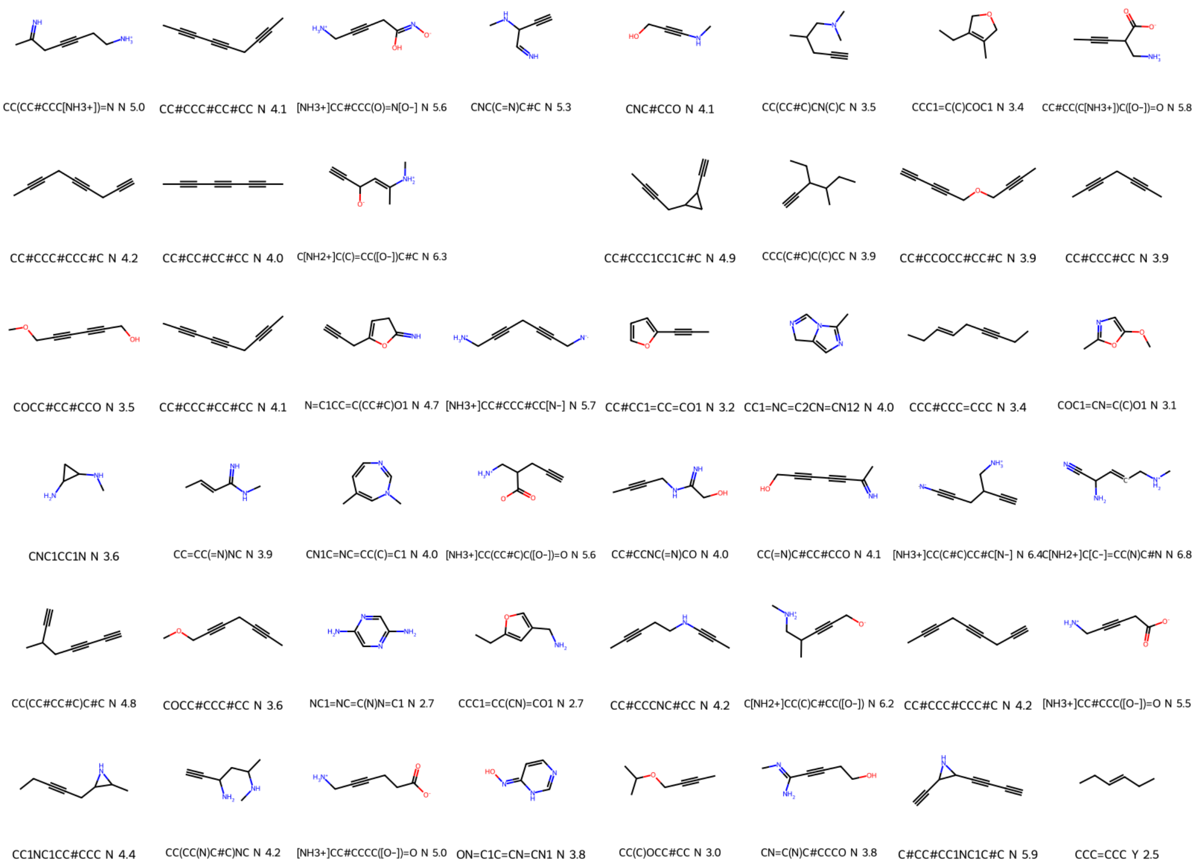

Supplementary Figure 55: Molecules obtained when trying to minimise the overlapping between host (CB6) and guest electron densities, while maximising their electrostatic interactions. $ed\_factor = 0.2$

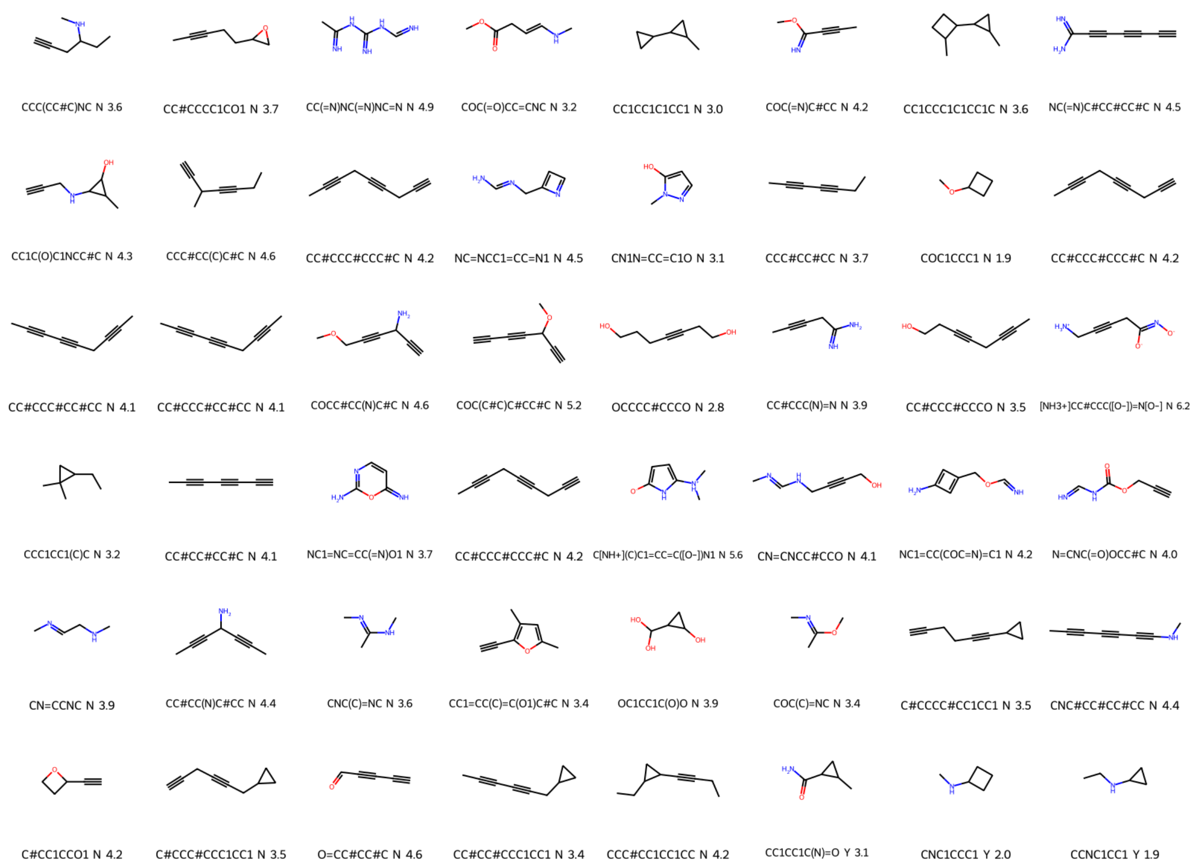

Supplementary Figure 56: Molecules obtained when trying to minimise the overlapping between host (CB6) and guest electron densities, while maximising their electrostatic interactions. $ed\_factor = 0.3$

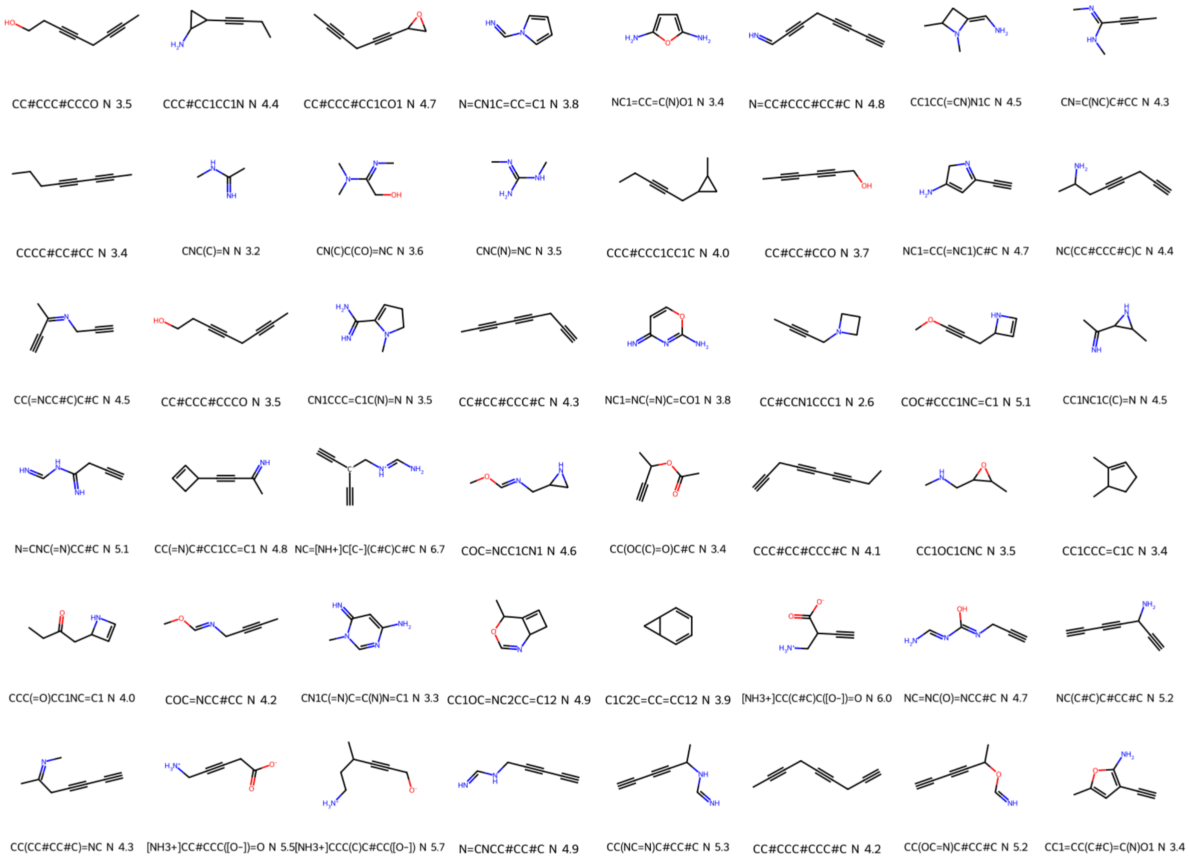

Supplementary Figure 57: Molecules obtained when trying to minimise the overlapping between host (CB6) and guest electron densities, while maximising their electrostatic interactions. $ed\_factor = 0.4$

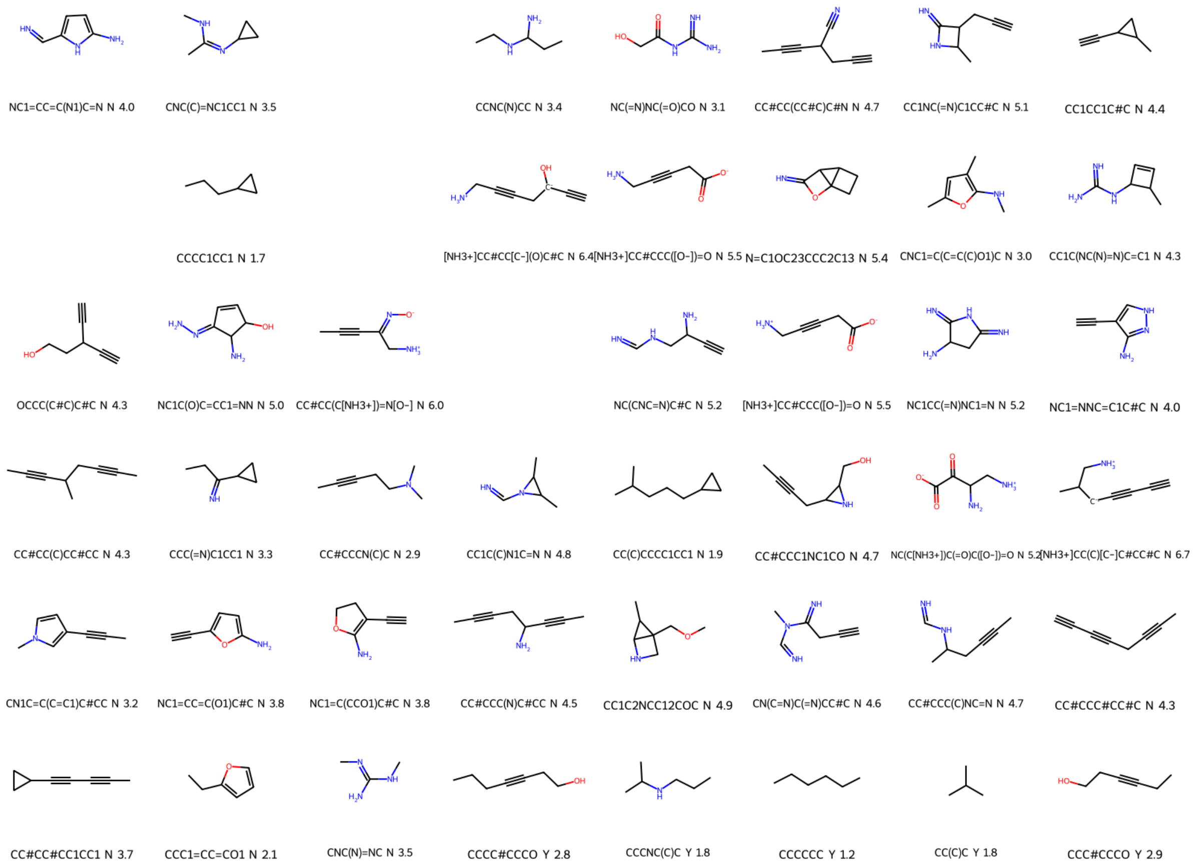

Supplementary Figure 58: Molecules obtained when trying to minimise the overlapping between host (CB6) and guest electron densities, while maximising their electrostatic interactions. $ed\_factor = 0.5$

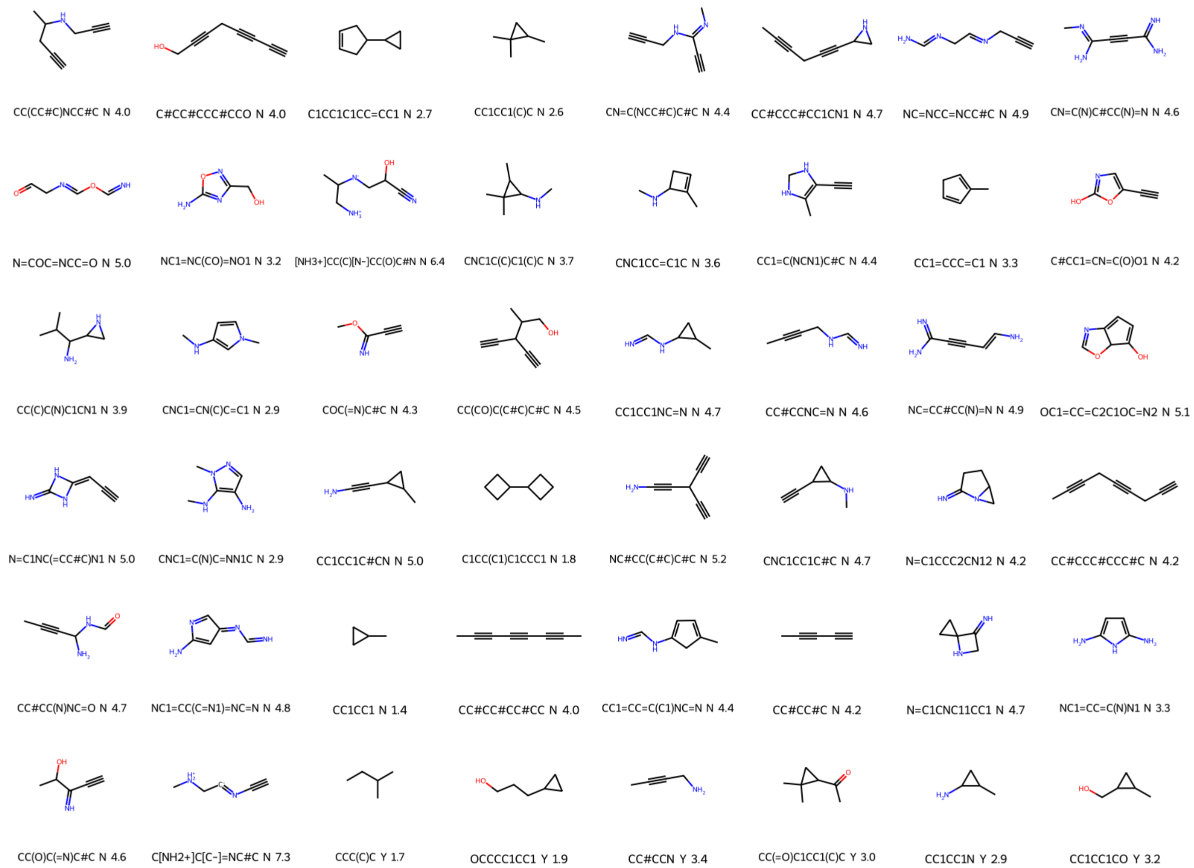

Supplementary Figure 59: Molecules obtained when trying to minimise the overlapping between host (CB6) and guest electron densities, while maximising their electrostatic interactions. $ed\_factor = 0.6$

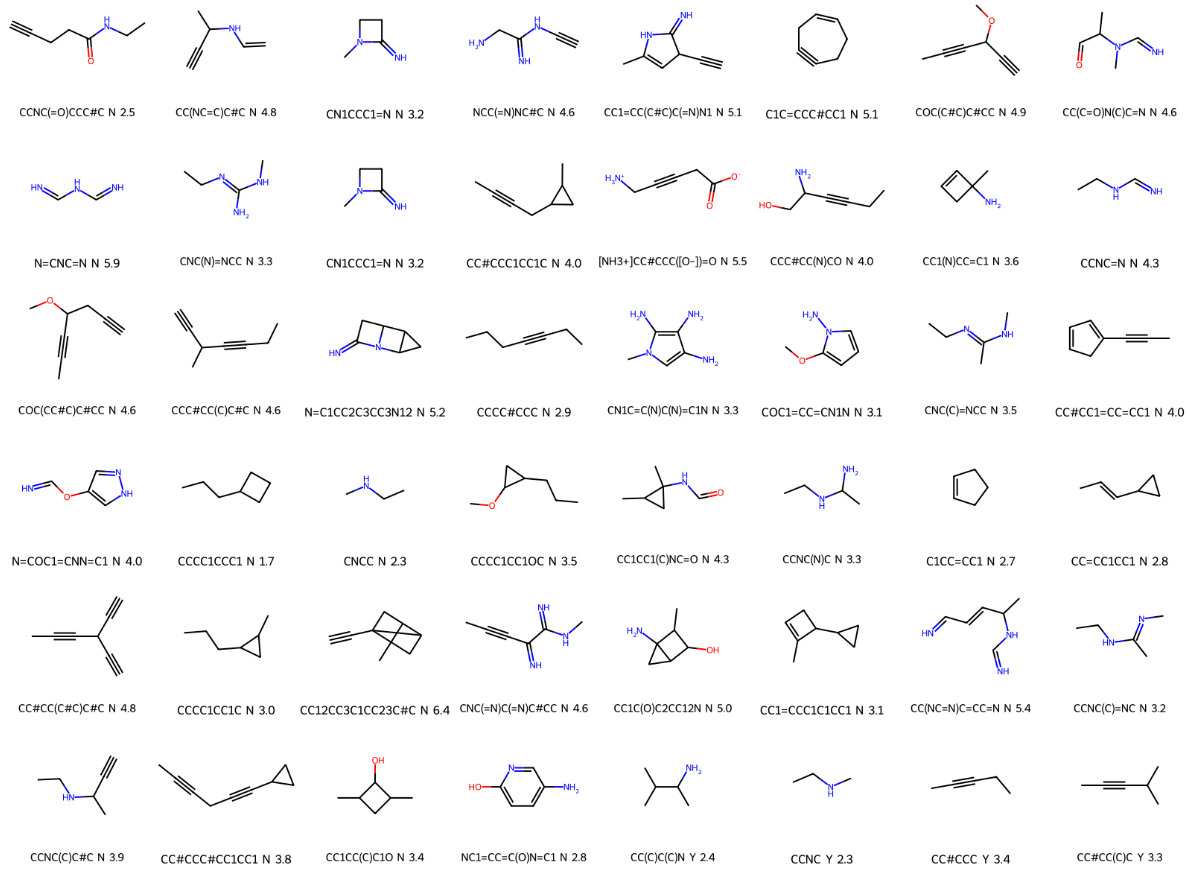

Supplementary Figure 60: Molecules obtained when trying to minimise the overlapping between host (CB6) and guest electron densities, while maximising their electrostatic interactions. $ed\_factor = 0.7$

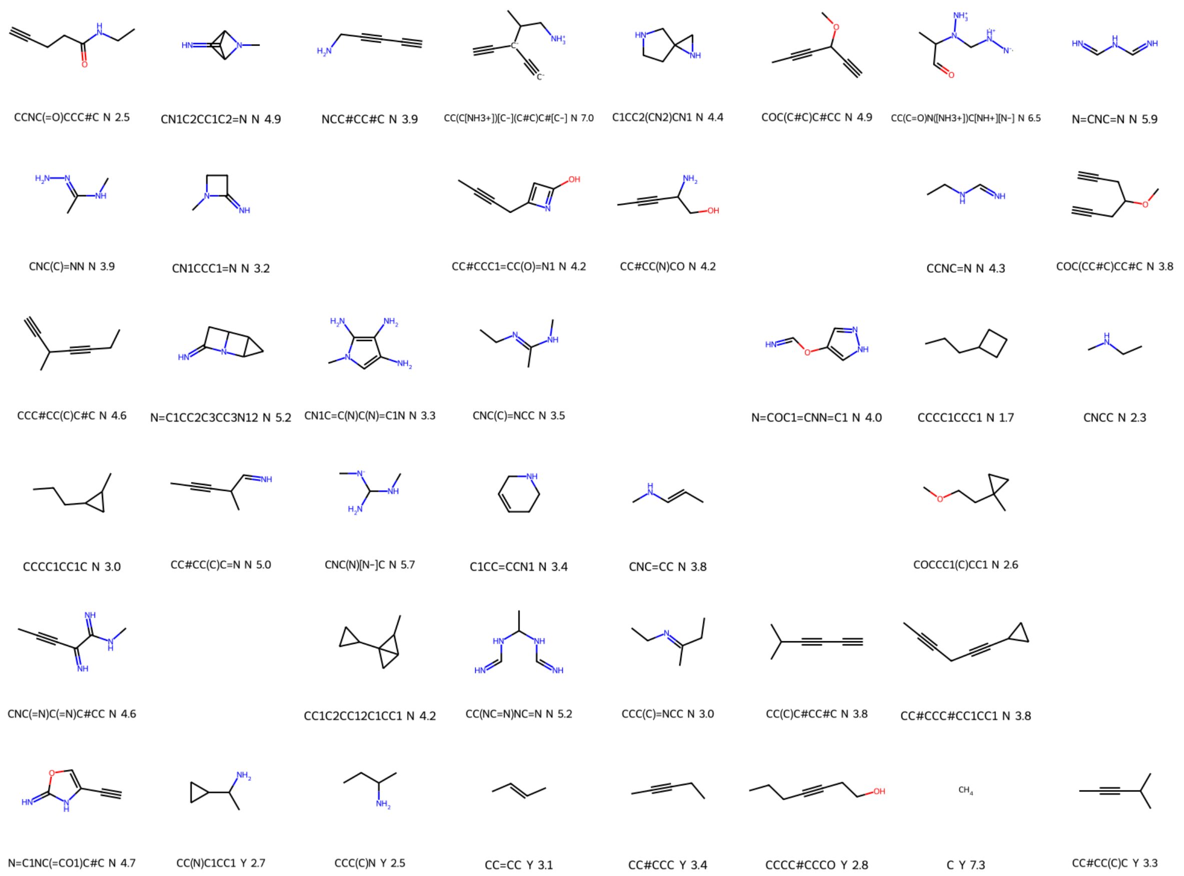

Supplementary Figure 61: Molecules obtained when trying to minimise the overlapping between host (CB6) and guest electron densities, while maximising their electrostatic interactions. These molecules were generated using the probabilistic sampling method. $ed\_factor = 0.7$

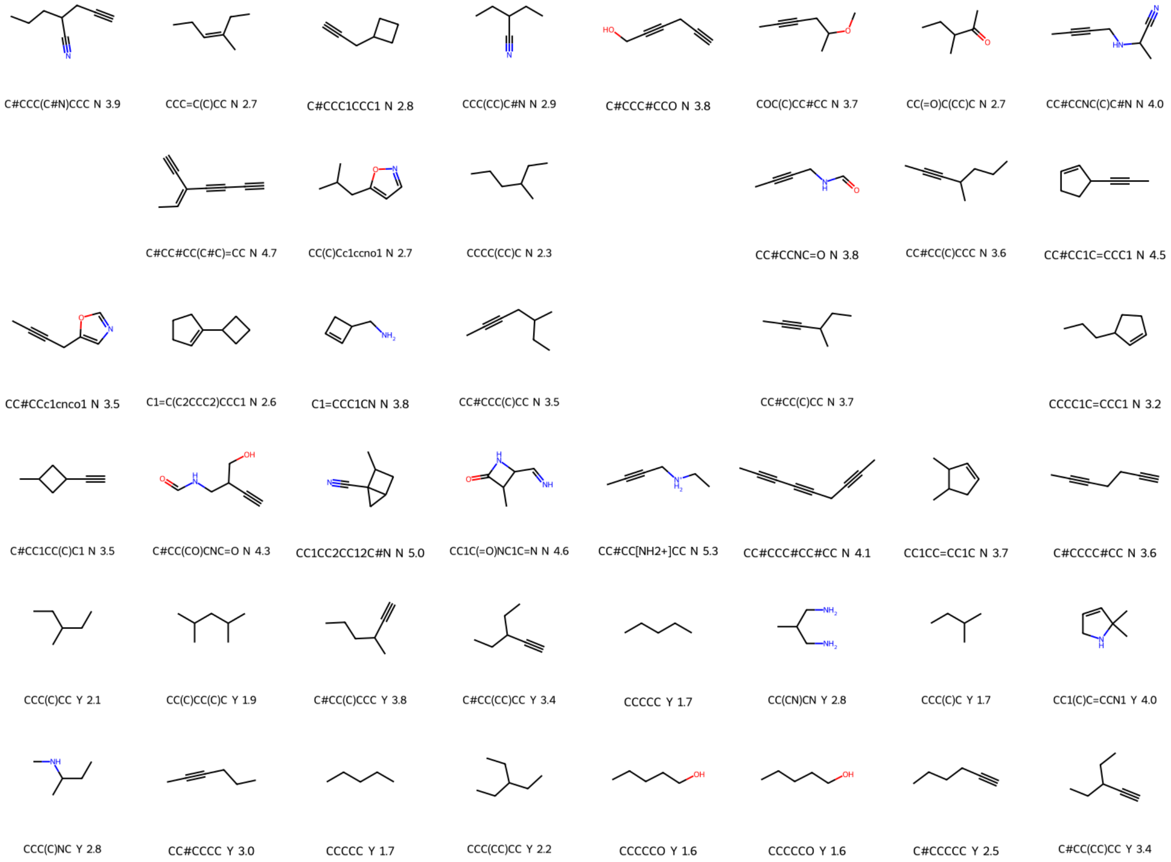

Supplementary Figure 62: Molecules obtained when trying to minimise the overlapping between host (CB6) and guest electron densities, while maximising their electrostatic interactions. These molecules were generated using the probabilistic sampling method. The SMILES representation of these molecules was calculated using only their electron densities. $ed\_factor = 0.7$

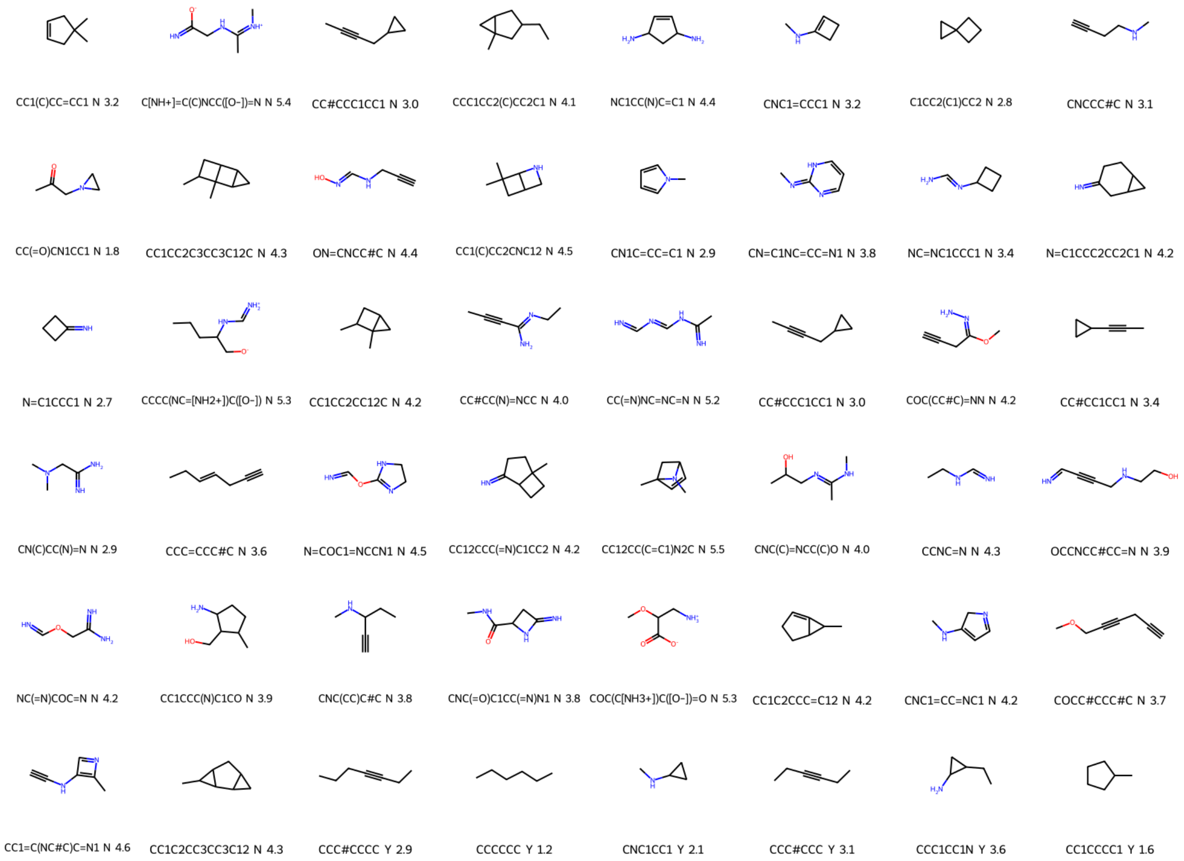

Supplementary Figure 63: Molecules obtained when trying to minimise the overlapping between host (CB6) and guest electron densities, while maximising their electrostatic interactions. $ed\_factor = 0.8$

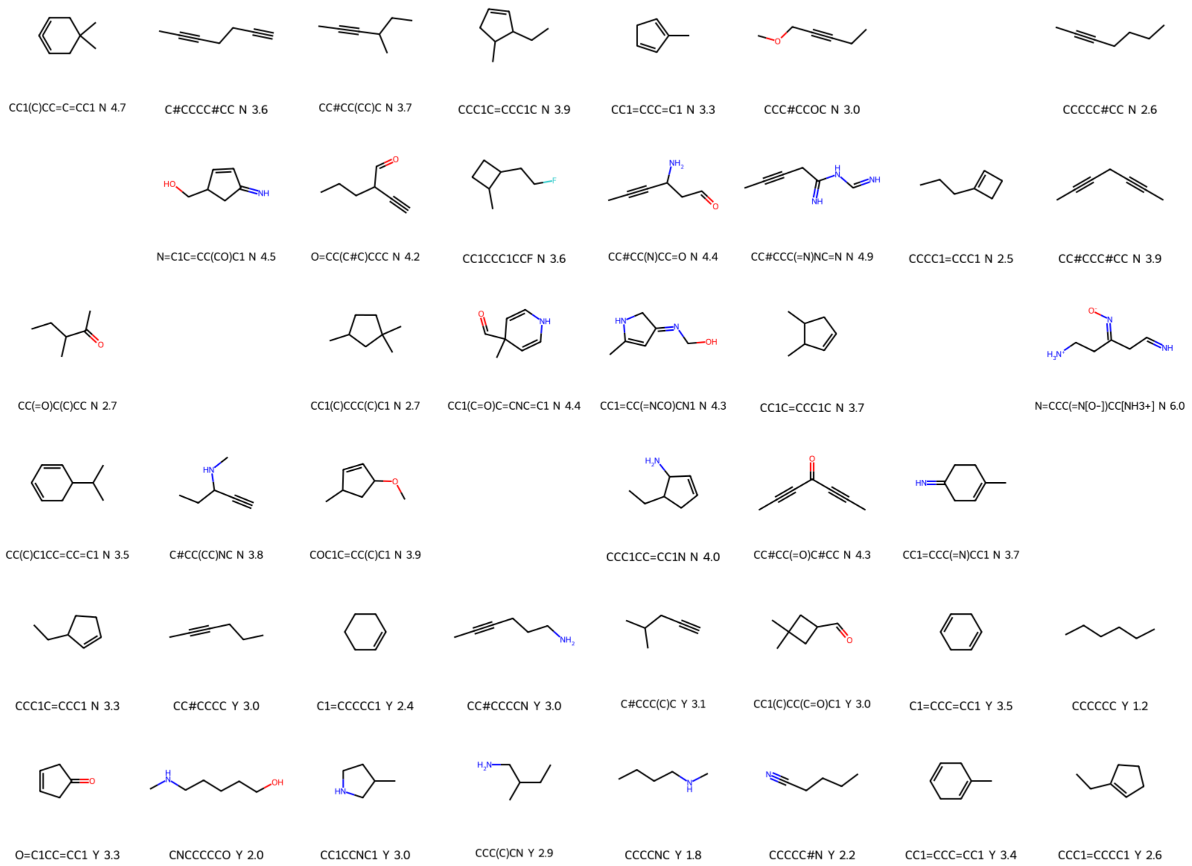

Supplementary Figure 64: Molecules obtained when trying to minimise the overlapping between host (CB6) and guest electron densities, while maximising their electrostatic interactions. These molecules were generated using the probabilistic sampling method. The SMILES representation of these molecules was calculated using only their electron densities. $ed\_factor = 0.8$

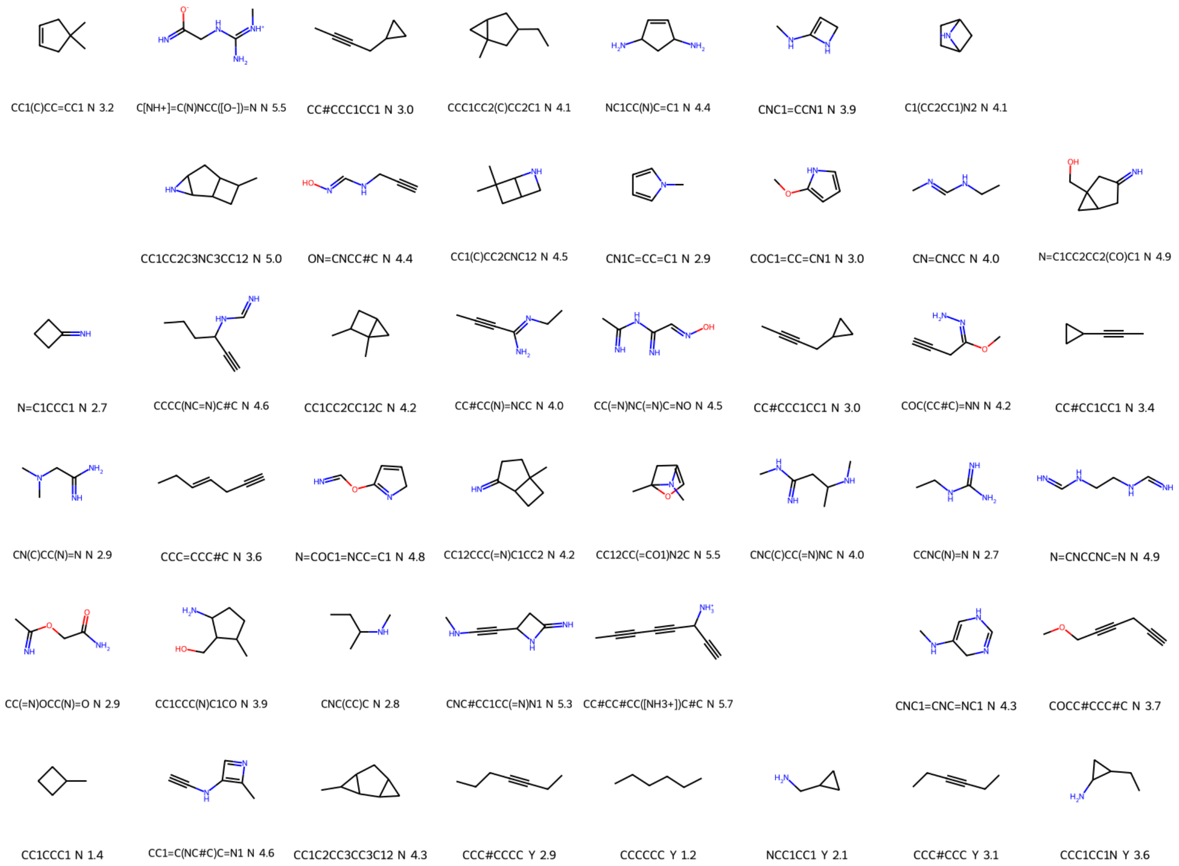

Supplementary Figure 65: Molecules obtained when trying to minimise the overlapping between host (CB6) and guest electron densities, while maximising their electrostatic interactions. These molecules were generated using the probabilistic sampling method. The SMILES representation of these molecules was calculated using only their electron densities. $ed\_factor = 0.8$

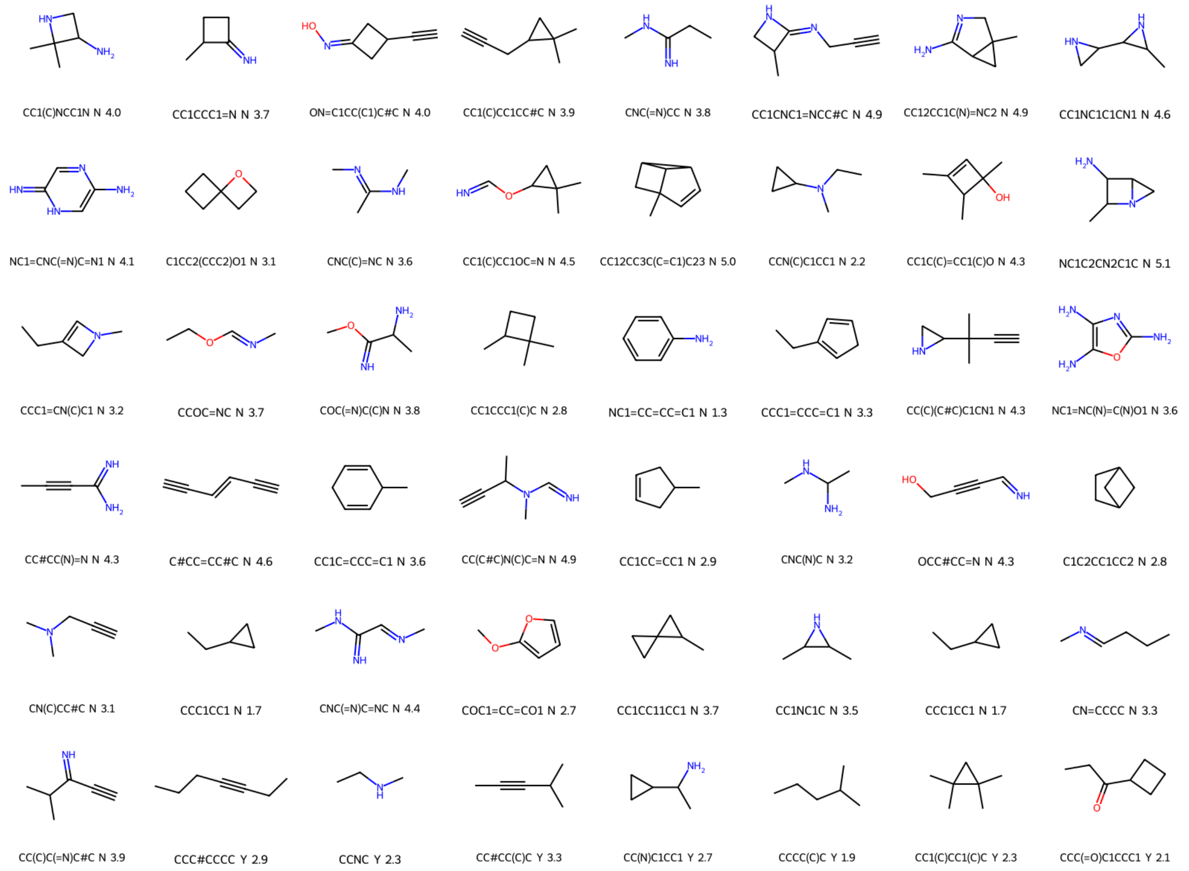

Supplementary Figure 66: Molecules obtained when trying to minimise the overlapping between host (CB6) and guest electron densities, while maximising their electrostatic interactions. $ed\_factor = 0.9$

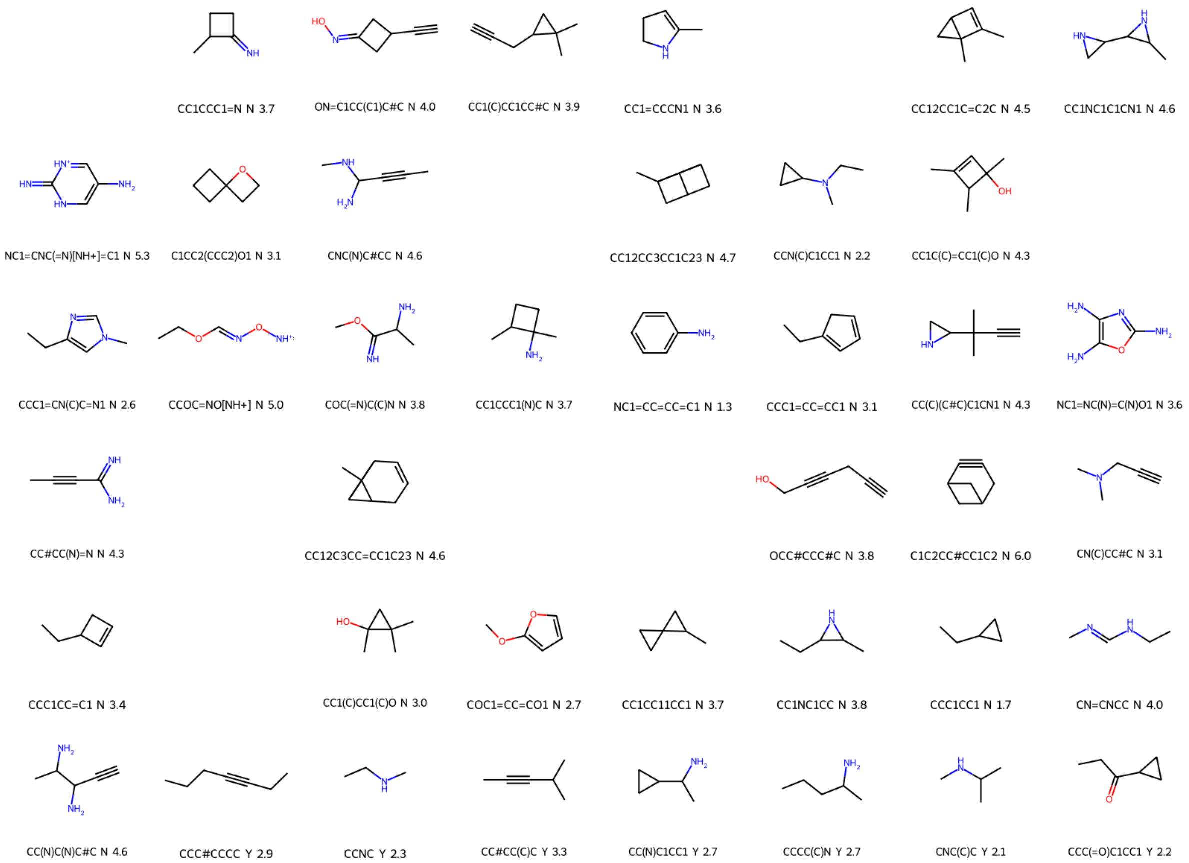

Supplementary Figure 67: Molecules obtained when trying to minimise the overlapping between host (CB6) and guest electron densities, while maximising their electrostatic interactions. $ed\_factor = 0.95$

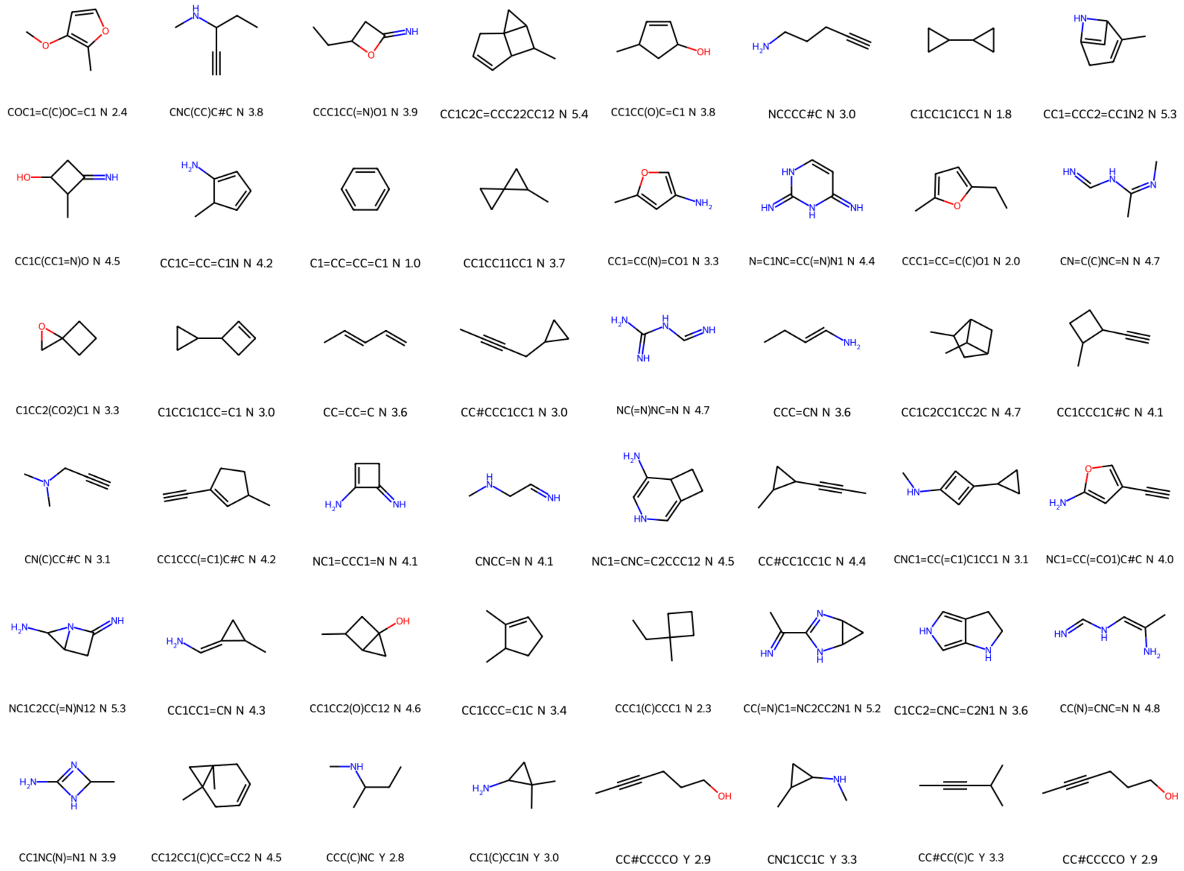

Supplementary Figure 68: Molecules obtained when trying to minimise the overlapping between host (CB6) and guest electron densities, while maximising their electrostatic interactions. $ed\_factor = 0.95$

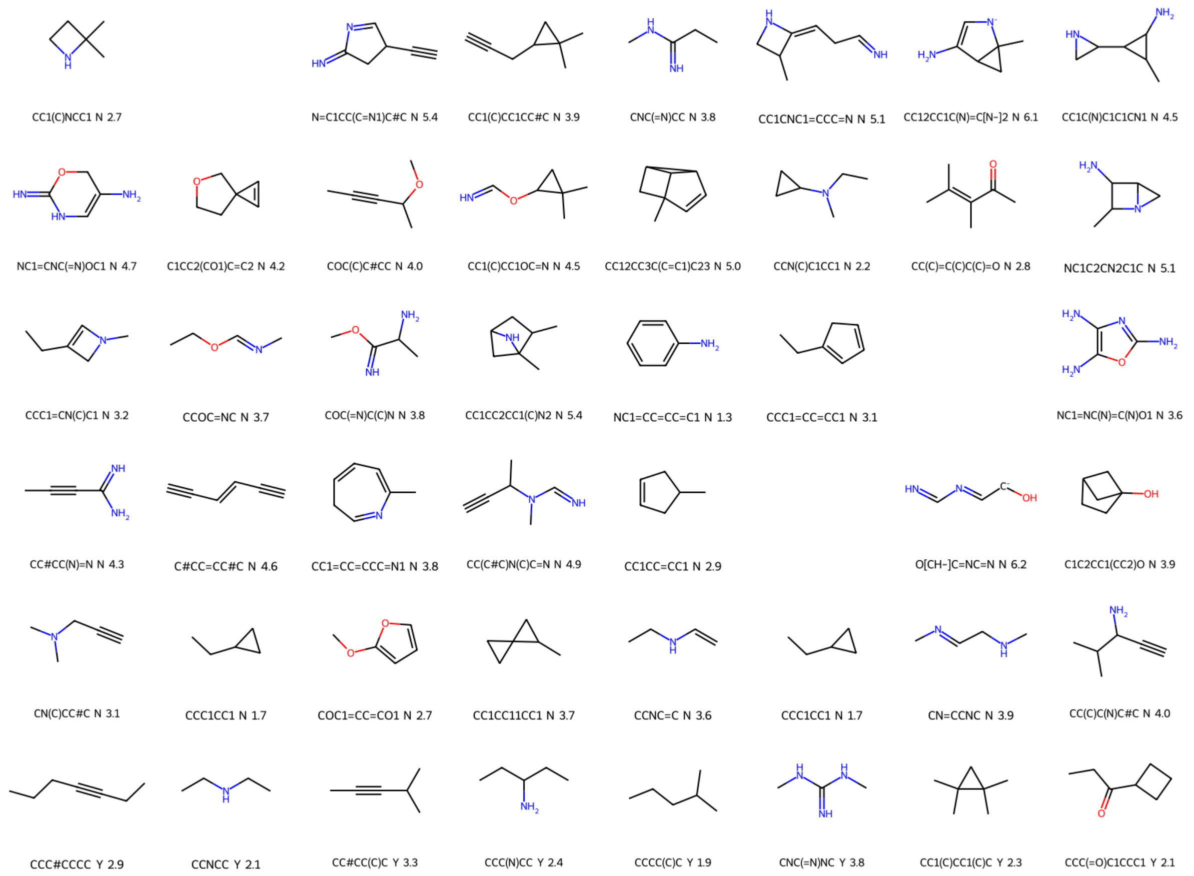

Supplementary Figure 69: Molecules obtained when trying to minimise the overlapping between host (CB6) and guest electron densities, while maximising their electrostatic interactions. $ed\_factor = 0.95$

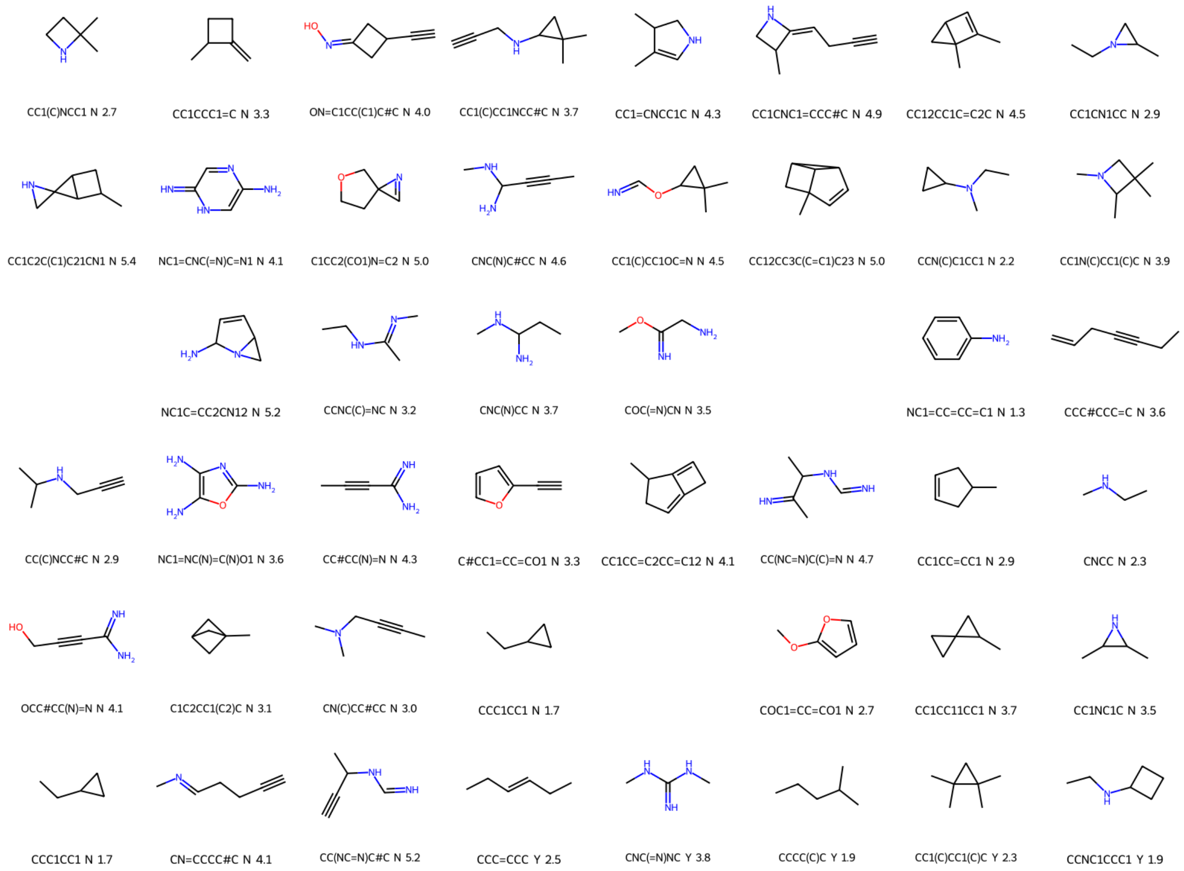

Supplementary Figure 70: Molecules obtained when trying to minimise the overlapping between host (CB6) and guest electron densities, while maximising their electrostatic interactions. $ed\_factor = 0.95$

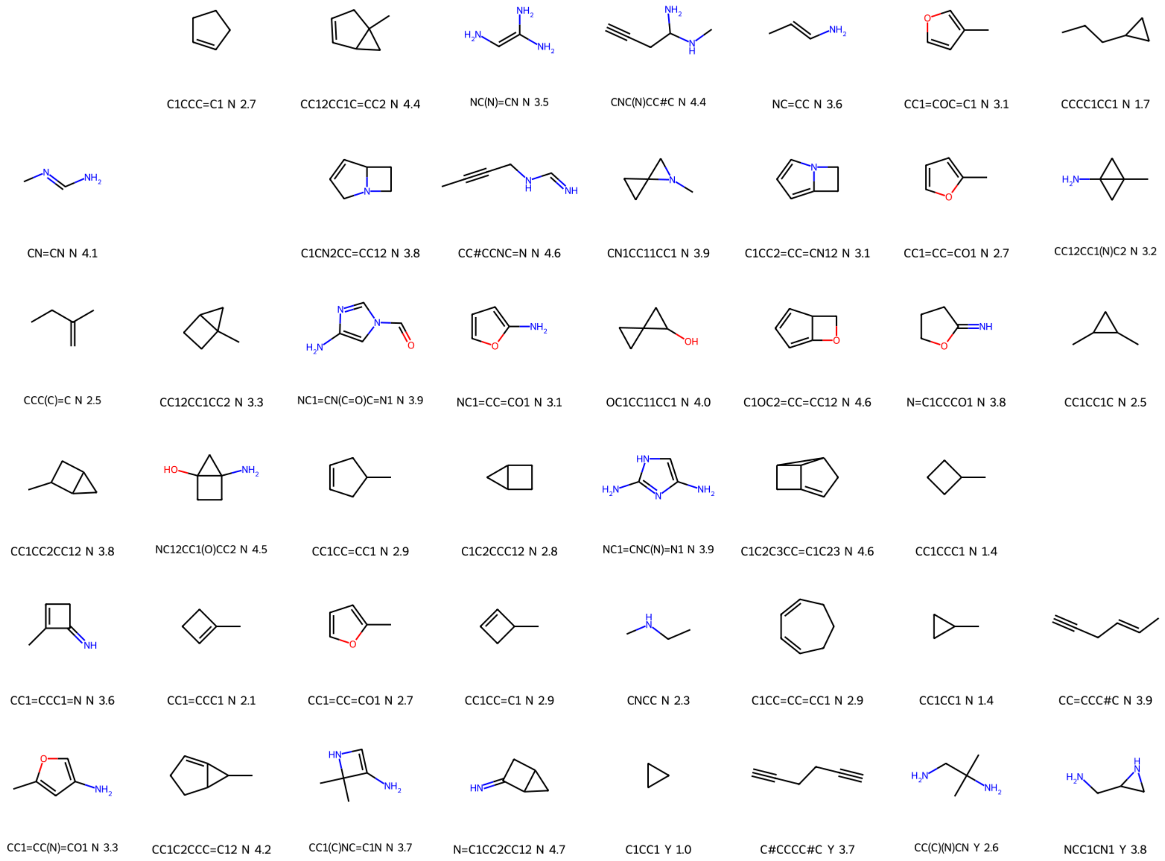

Supplementary Figure 71: Molecules obtained when trying to minimise the overlapping between host (CB6) and guest electron densities, while maximising their electrostatic interactions. $ed\_factor = 1.0$

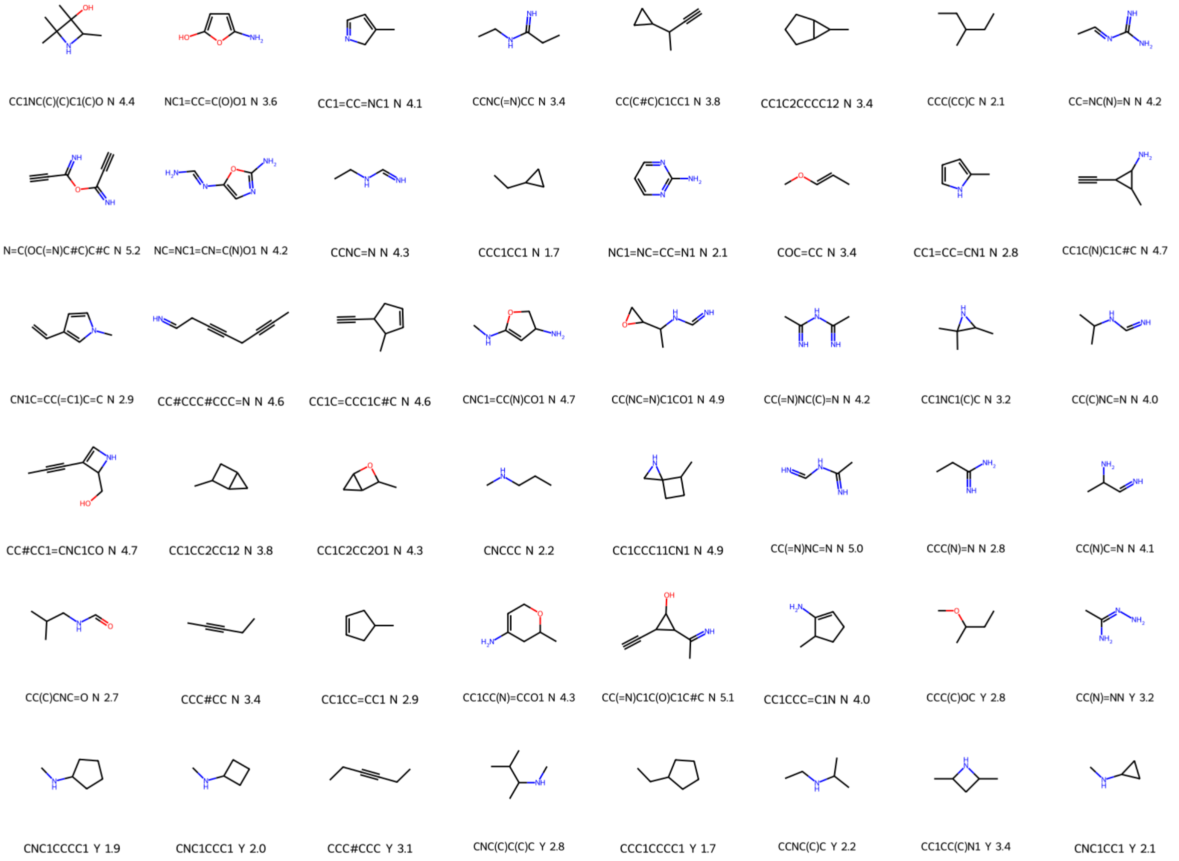

Supplementary Figure 72: Molecules obtained when trying to minimise the overlapping between host (CB6) and guest electron densities, while maximising their electrostatic interactions. The size of the cavity was reduced using the method described in Supplementary Section 1.9. $ed\_factor = 0.6$

Similar optimisation rounds were done decreasing the size of the cavity as described in Supplementary Section 1.9. A total of 27 optimisation rounds were executed, generating 1296 guests. As before, we will only highlight here some of the results obtained. All of the following guests were obtained for the CB6 host decreasing its cavity by using a kernel of size 4 (using the method described in Supplementary Section 1.9). See Supplementary Figures 72 to 76.

2.3.6 Results from bin/optimisers/cage_hg.py

These results were obtained using the `cage_hg.py` script described in Supplementary Section 2.2.6. The results can be seen in Supplementary Figure 77.

2.3.7 Results from bin/optimisers/cage_hg_esp.py

These results were obtained using the `cage_hg_esp.py` script described in Supplementary Section 2.2.7. A total of 38 experiments/runs were done using this script using different values for `ed_factor`. This generated 1520 different guests. These were converted into SMILES sequences using the two methods based on electron density data described in

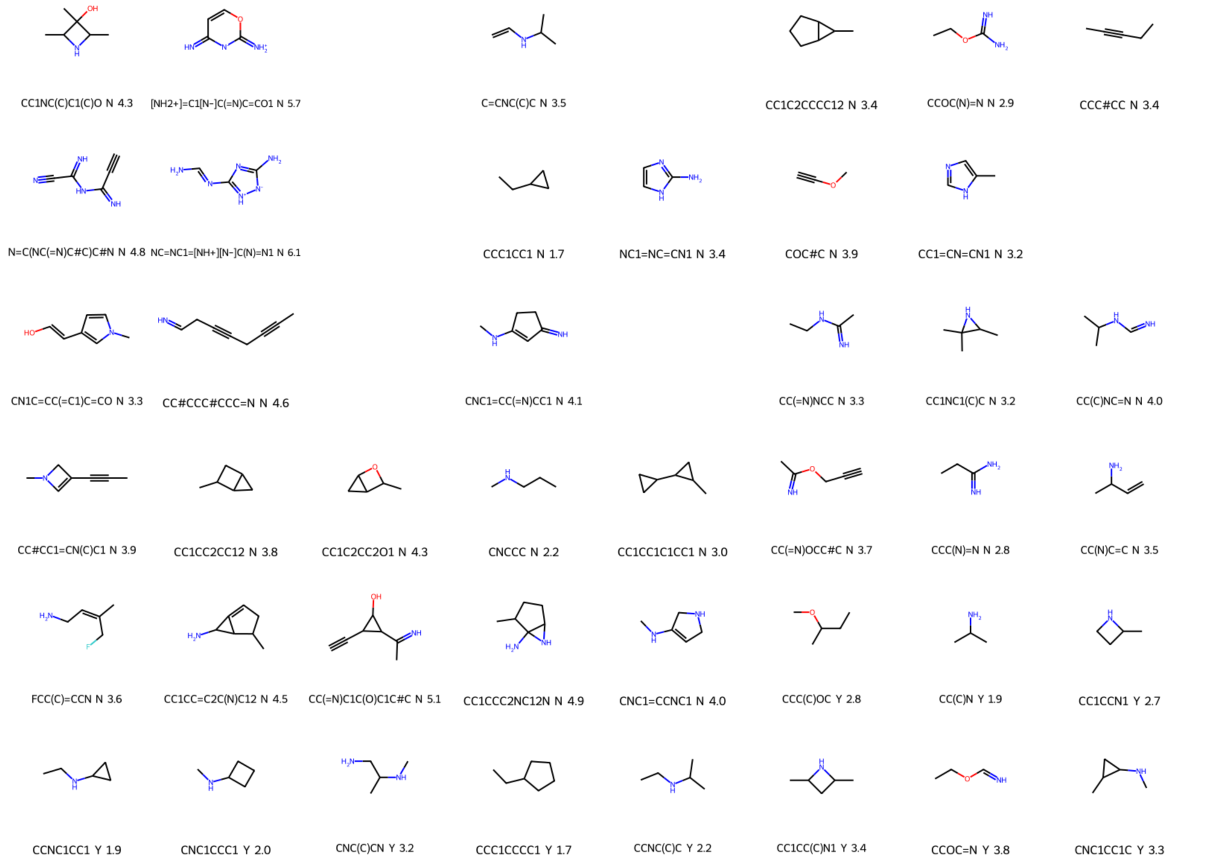

Supplementary Figure 73: Molecules obtained when trying to minimise the overlapping between host (CB6) and guest electron densities, while maximising their electrostatic interactions. These molecules were obtained using the probabilistic sampling method. The size of the cavity was reduced using the method described in Supplementary Section 1.9. $ed\_factor = 0.6$

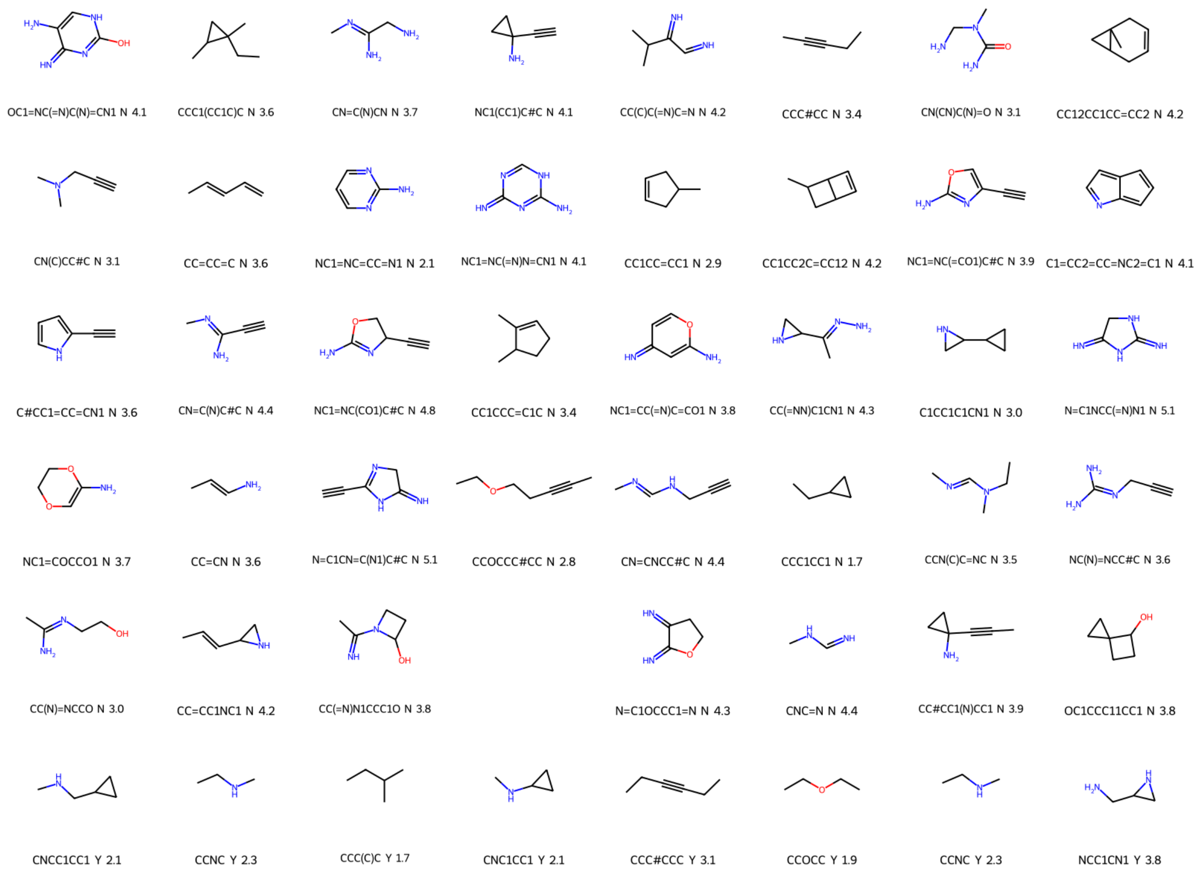

Supplementary Figure 74: Molecules obtained when trying to minimise the overlapping between host (CB6) and guest electron densities, while maximising their electrostatic interactions. The size of the cavity was reduced using the method described in Supplementary Section 1.9. $ed\_factor = 0.7$

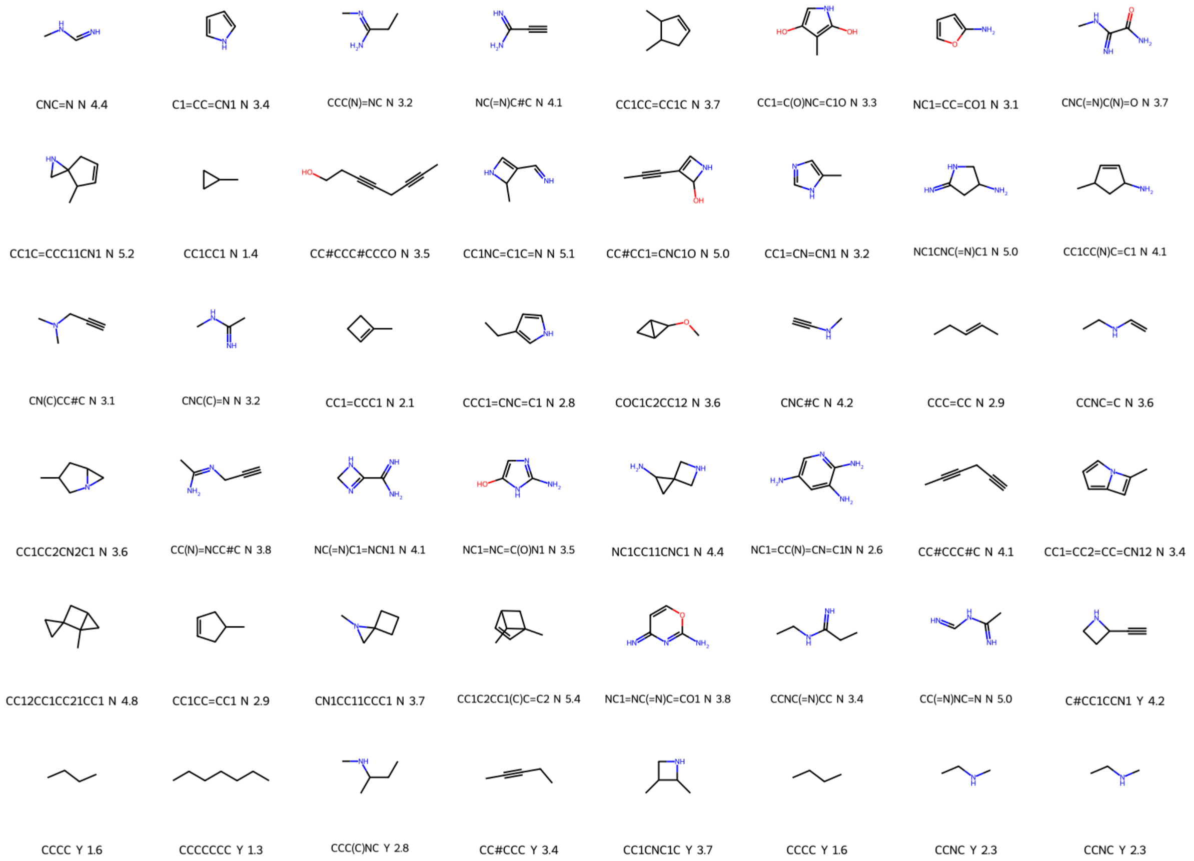

Supplementary Figure 75: Molecules obtained when trying to minimise the overlapping between host (CB6) and guest electron densities, while maximising their electrostatic interactions. The size of the cavity was reduced using the method described in Supplementary Section 1.9. $ed\_factor = 0.8$

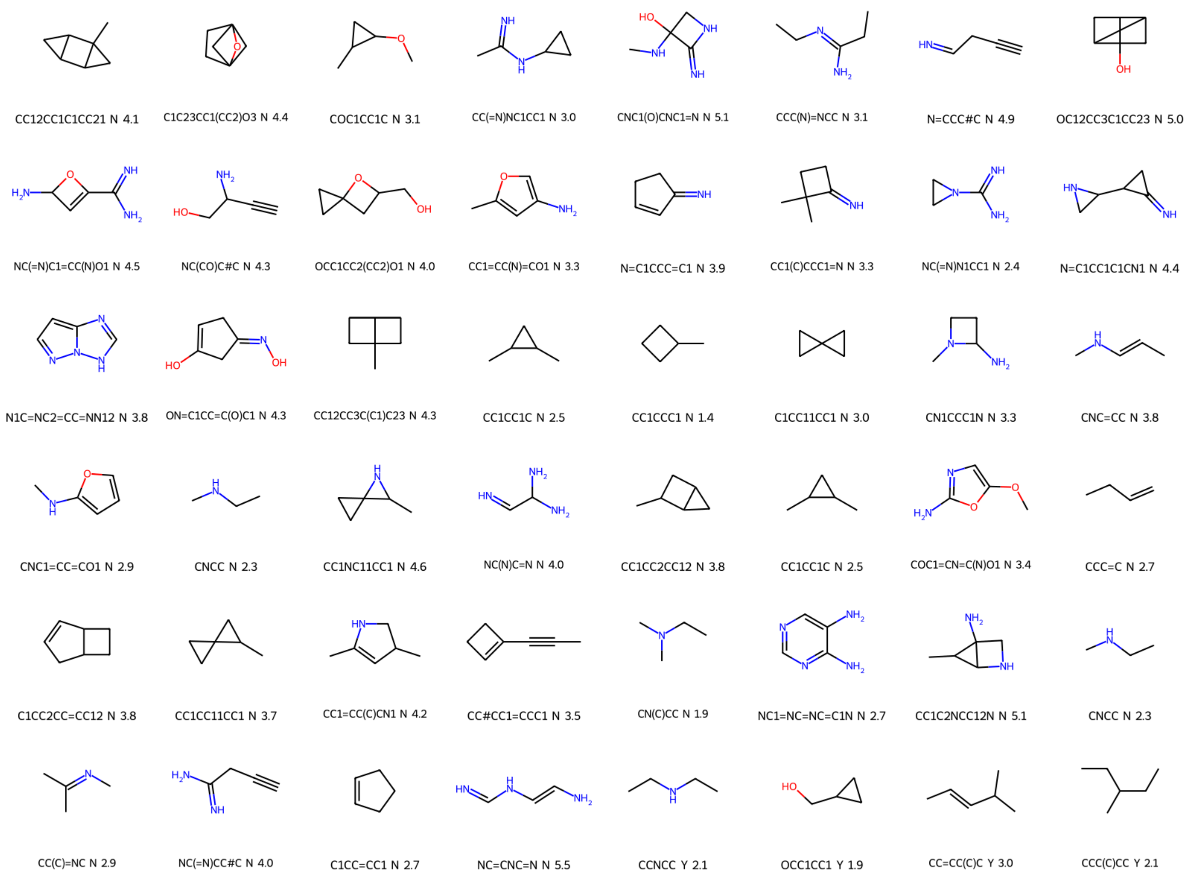

Supplementary Figure 76: Molecules obtained when trying to minimise the overlapping between host (CB6) and guest electron densities, while maximising their electrostatic interactions. The size of the cavity was reduced using the method described in Supplementary Section 1.9. $ed\_factor = 0.9$

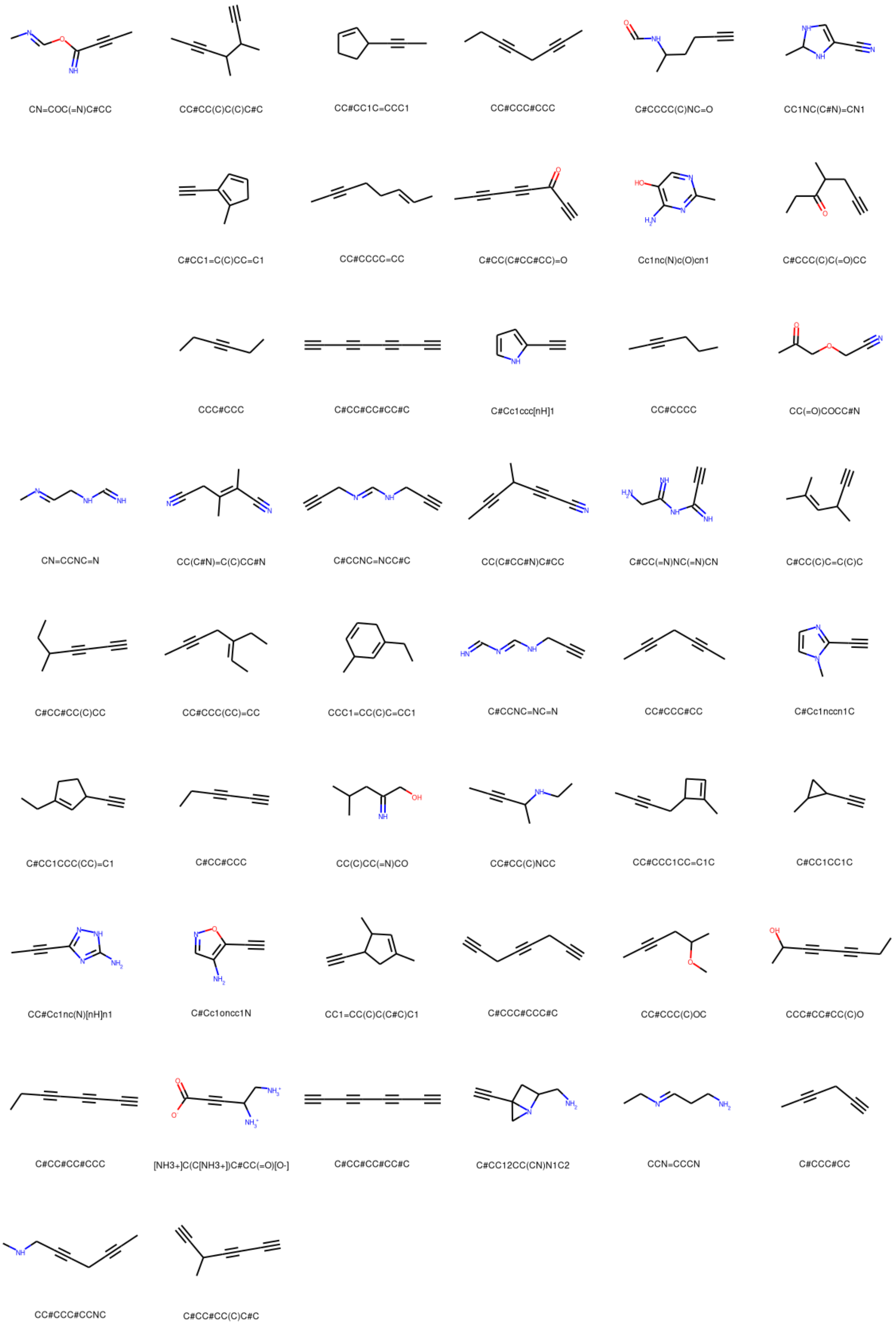

Supplementary Figure 77: Molecules obtained when trying to minimise the overlapping between host (Pd Cage) and guest electron densities.

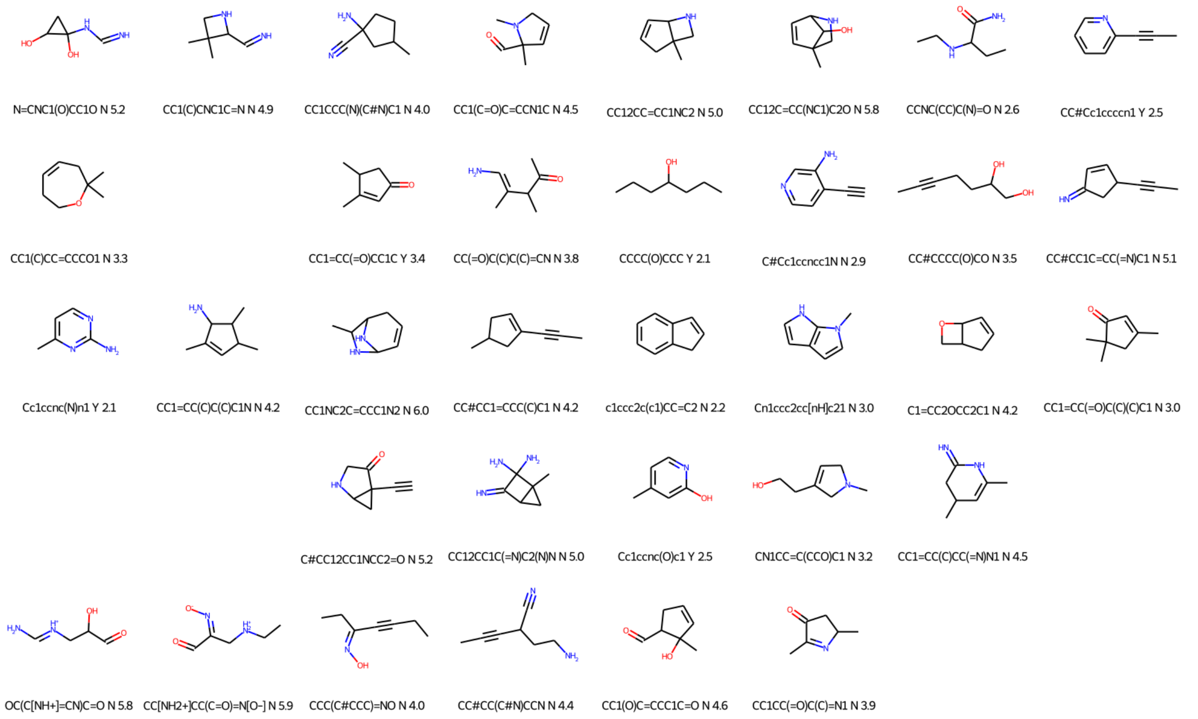

Supplementary Figure 78: Molecules obtained when trying to minimise the overlapping between host (Pd Cage) and guest electron densities, while maximising their electrostatic interactions. $ed\_factor = 0$

Supplementary Section 1.11, plus the method using decorated electron densities described in Supplementary Section 1.8. Therefore, each 3D tensor representing a guest could output three slightly different molecules. Thus, the total number of molecules generated was 4560. Since this is a very big number, we will only show some of there here. The results can be seen in Supplementary Figures 78 to 103.

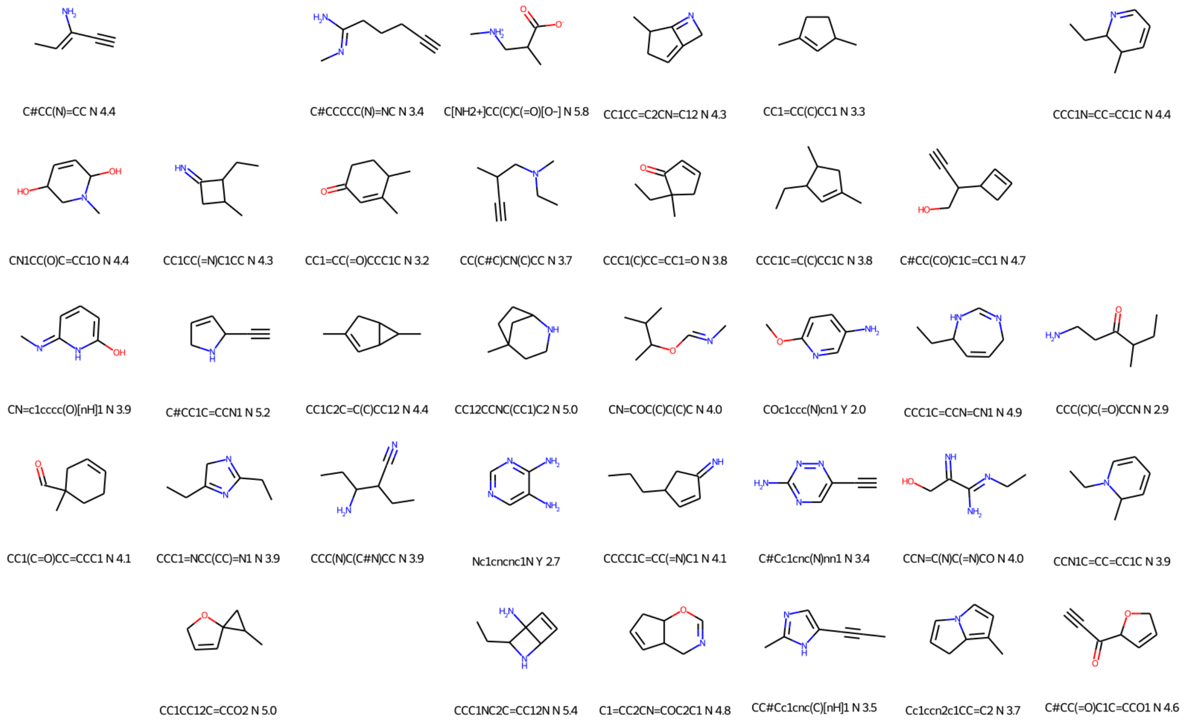

Supplementary Figure 79: Molecules obtained when trying to minimise the overlapping between host (Pd Cage) and guest electron densities, while maximising their electrostatic interactions. $ed\_factor = 0.001$

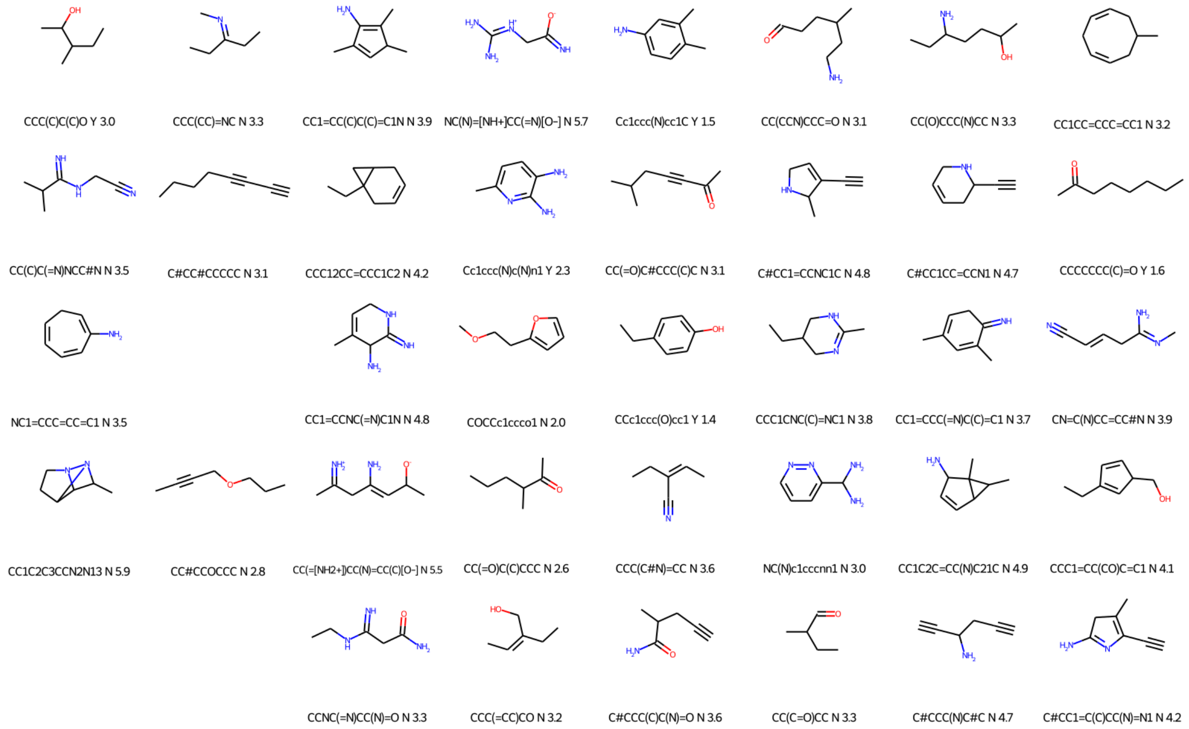

Supplementary Figure 80: Molecules obtained when trying to minimise the overlapping between host (Pd Cage) and guest electron densities, while maximising their electrostatic interactions. $ed\_factor = 0.05$

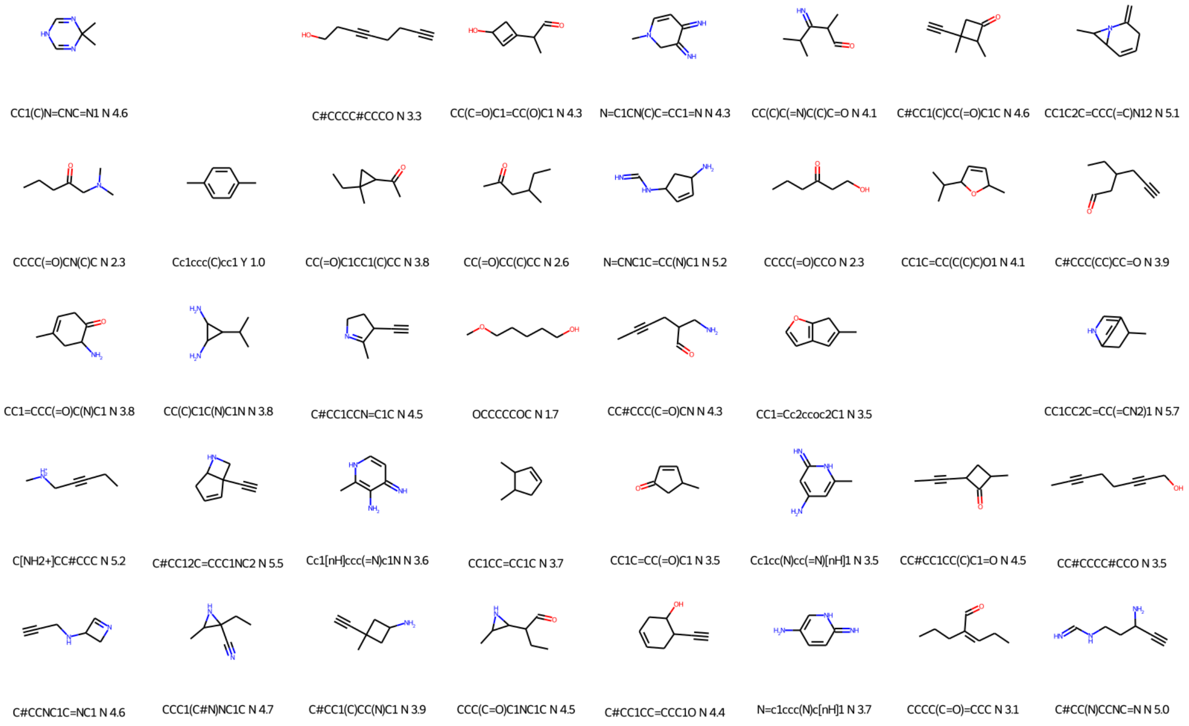

Supplementary Figure 81: Molecules obtained when trying to minimise the overlapping between host (Pd Cage) and guest electron densities, while maximising their electrostatic interactions. $ed\_factor = 0.1$

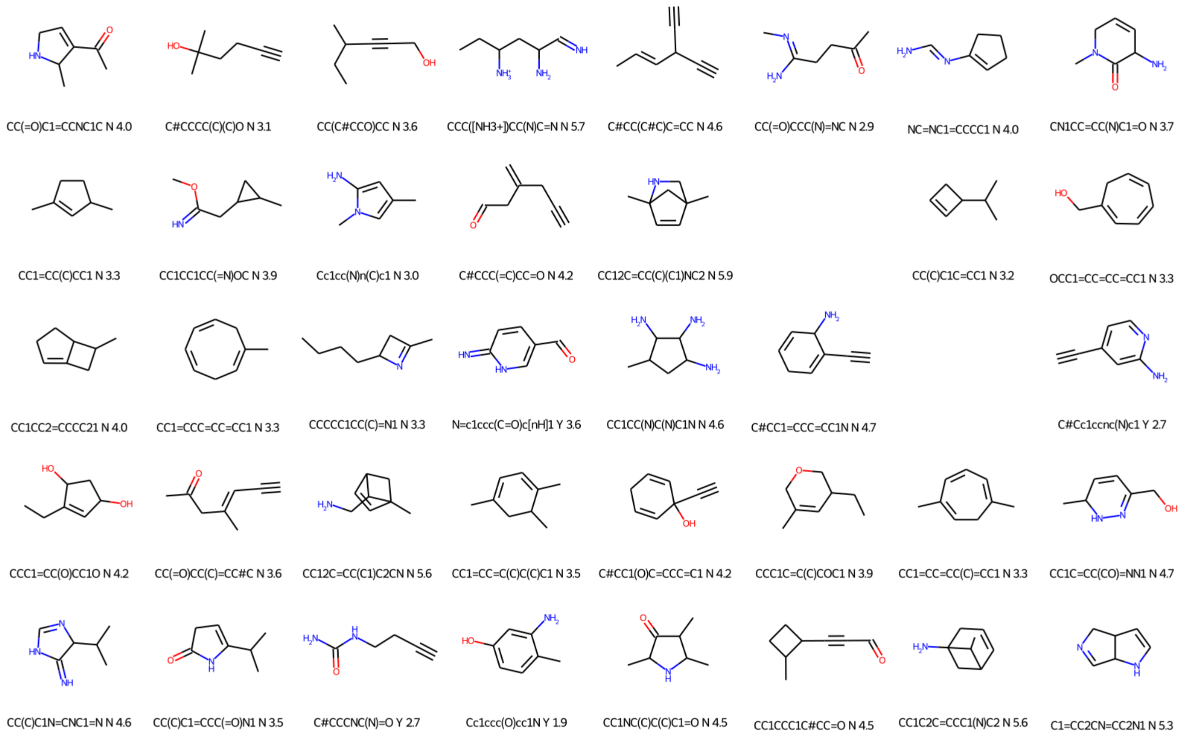

Supplementary Figure 82: Molecules obtained when trying to minimise the overlapping between host (Pd Cage) and guest electron densities, while maximising their electrostatic interactions. $ed\_factor = 0.2$

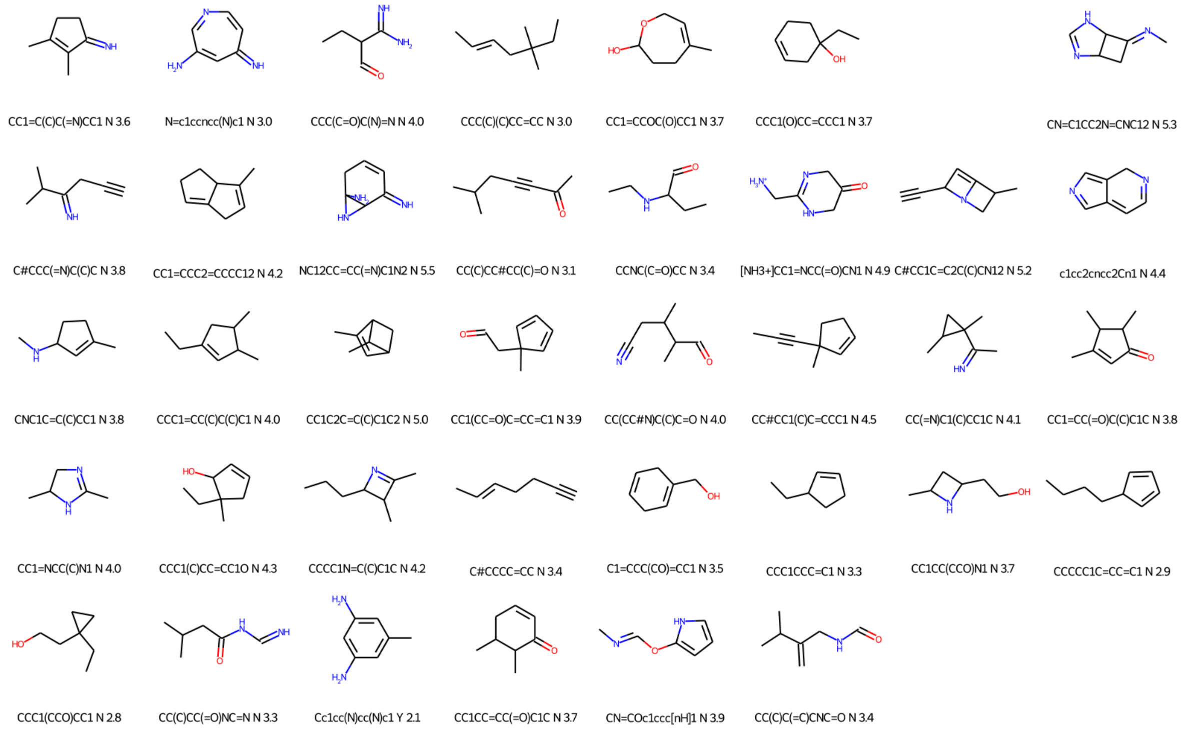

Supplementary Figure 83: Molecules obtained when trying to minimise the overlapping between host (Pd Cage) and guest electron densities, while maximising their electrostatic interactions. $ed\_factor = 0.3$

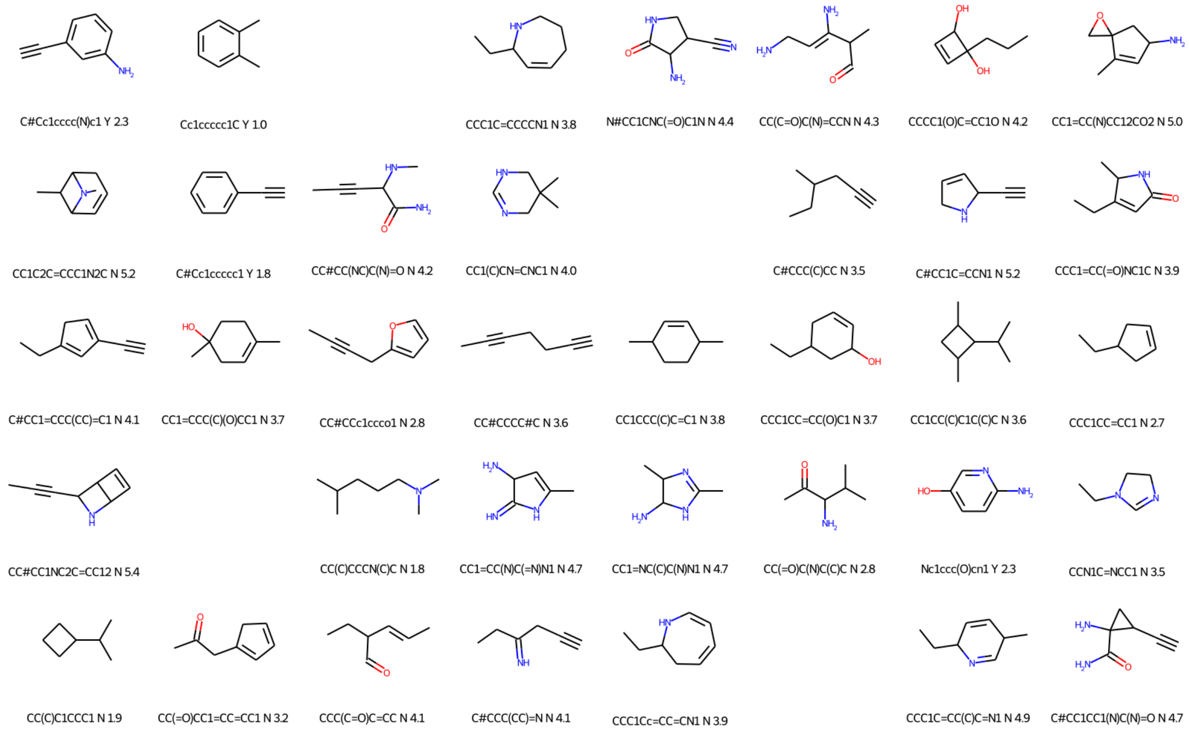

Supplementary Figure 84: Molecules obtained when trying to minimise the overlapping between host (Pd Cage) and guest electron densities, while maximising their electrostatic interactions. $ed\_factor = 0.5$

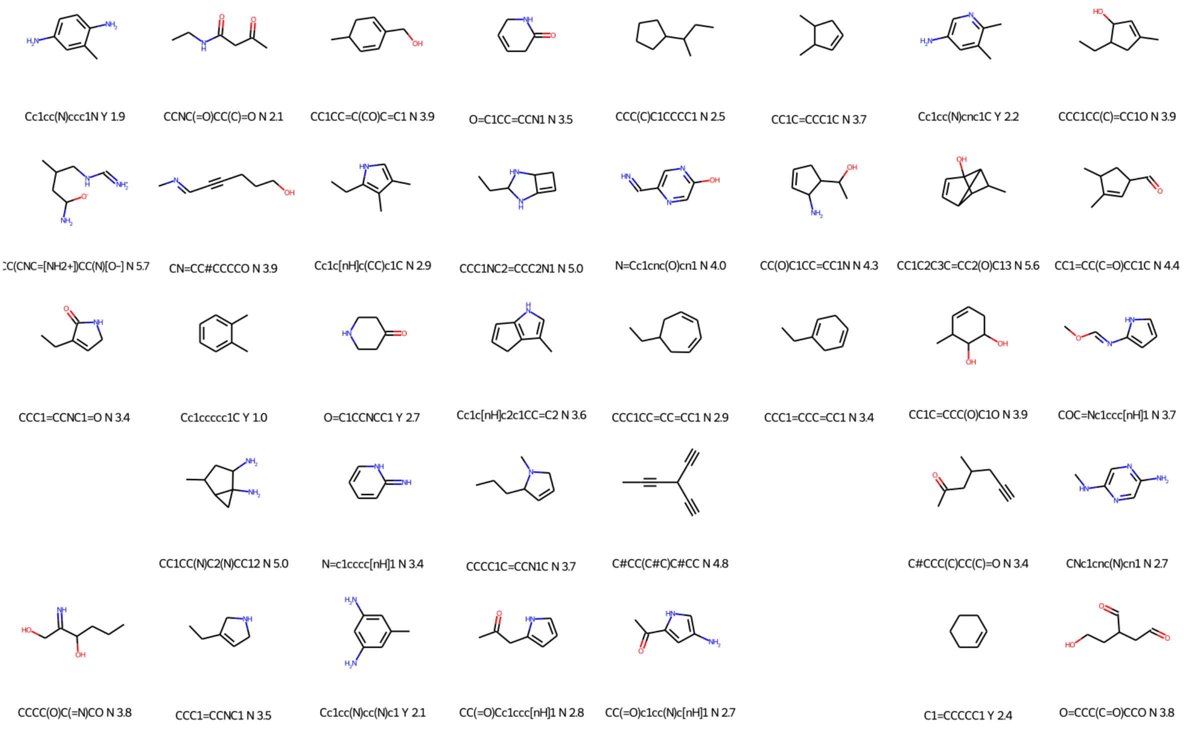

Supplementary Figure 85: Molecules obtained when trying to minimise the overlapping between host (Pd Cage) and guest electron densities, while maximising their electrostatic interactions. $ed\_factor = 0.7$

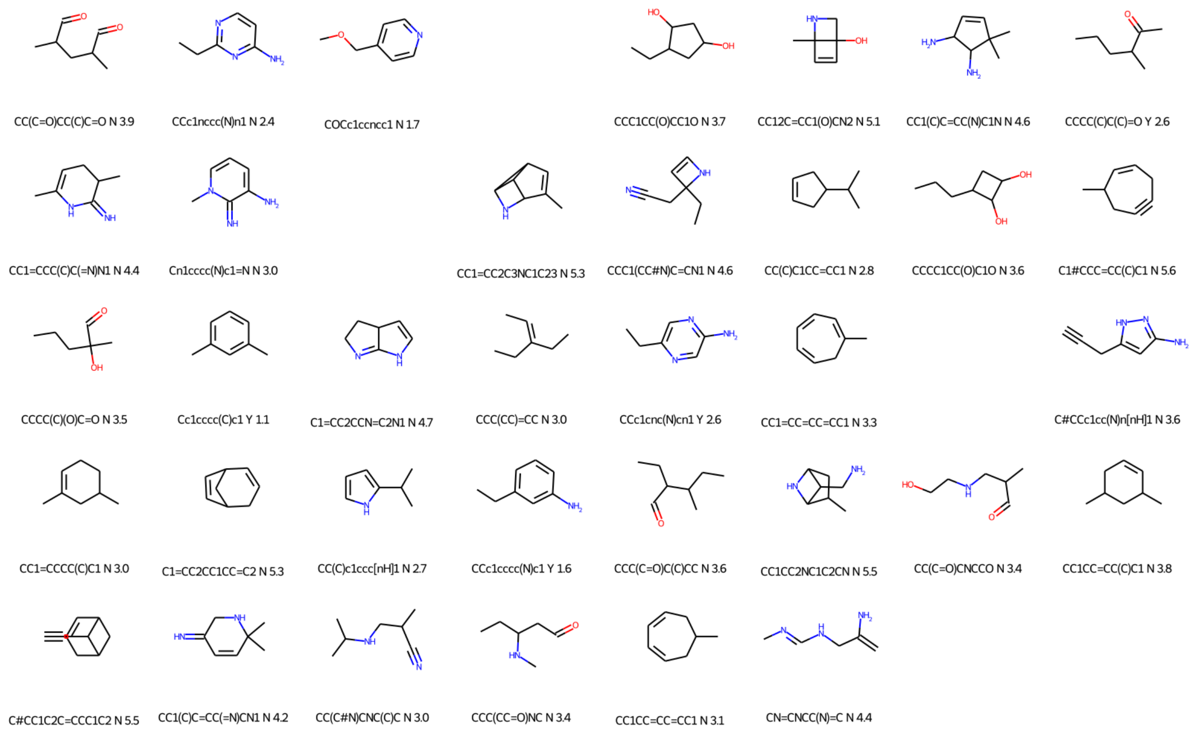

Supplementary Figure 86: Molecules obtained when trying to minimise the overlapping between host (Pd Cage) and guest electron densities, while maximising their electrostatic interactions. $ed\_factor = 0.8$

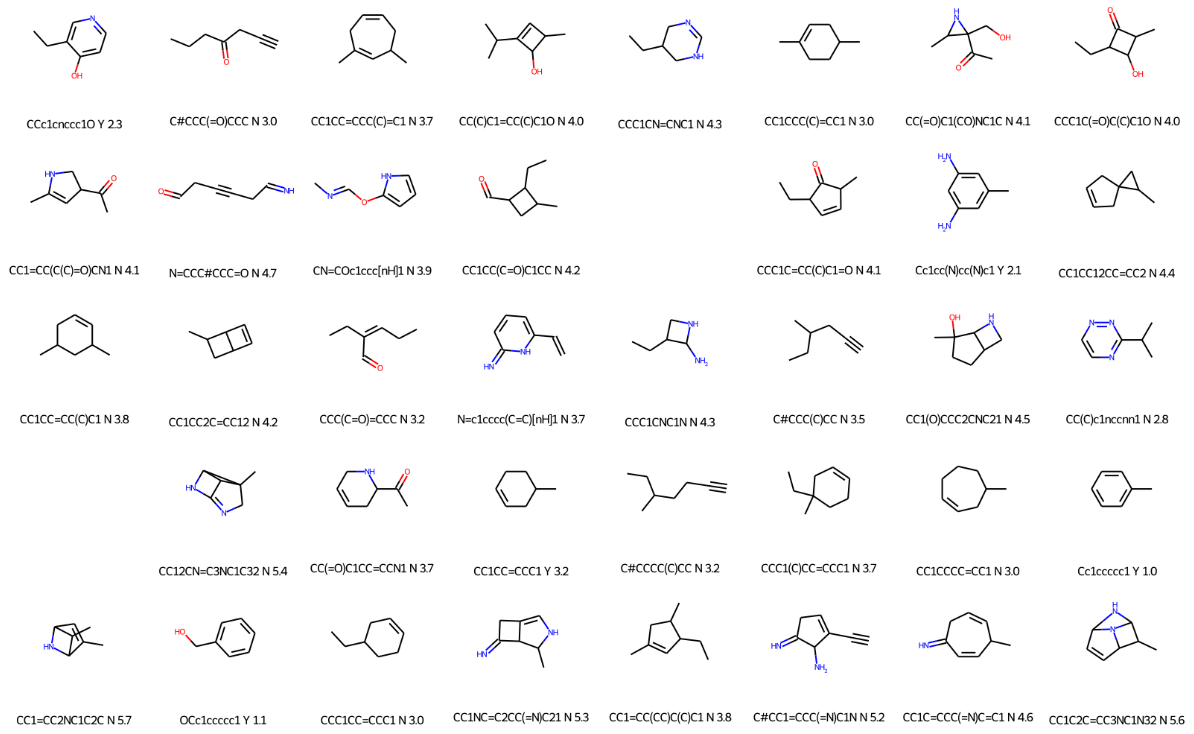

Supplementary Figure 87: Molecules obtained when trying to minimise the overlapping between host (Pd Cage) and guest electron densities, while maximising their electrostatic interactions. $ed\_factor = 0.9$

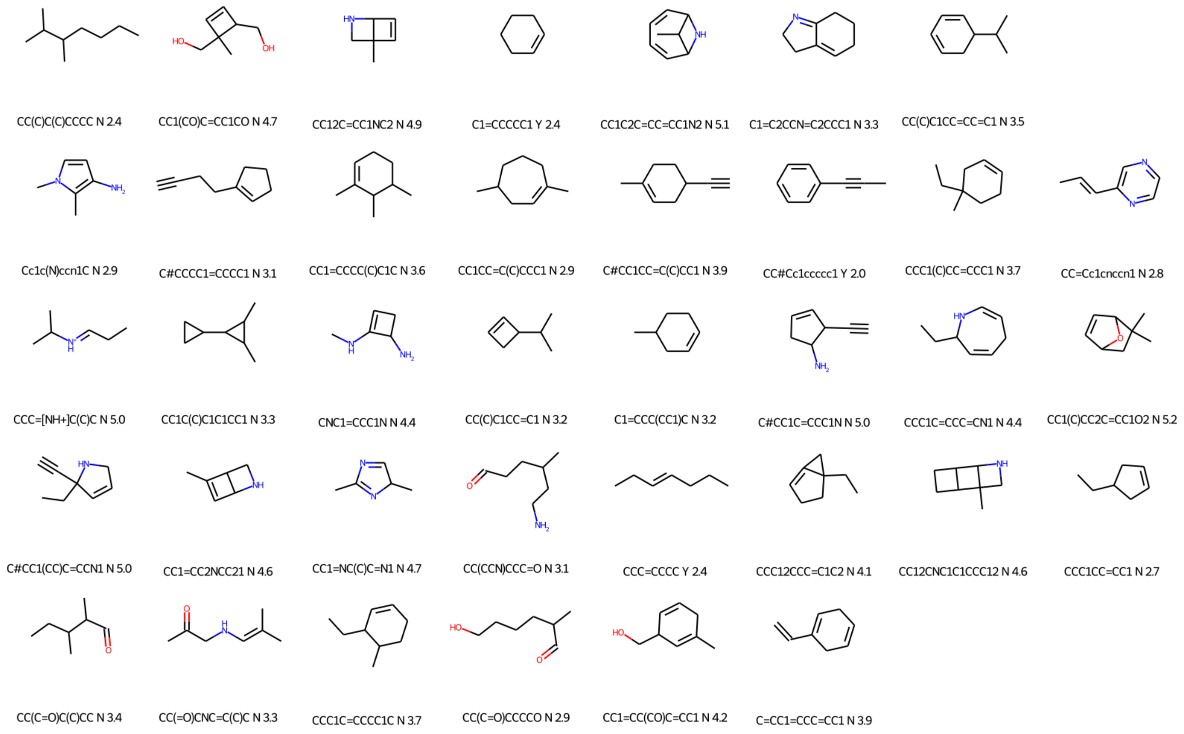

Supplementary Figure 88: Molecules obtained when trying to minimise the overlapping between host (Pd Cage) and guest electron densities, while maximising their electrostatic interactions. $ed\_factor = 0.95$

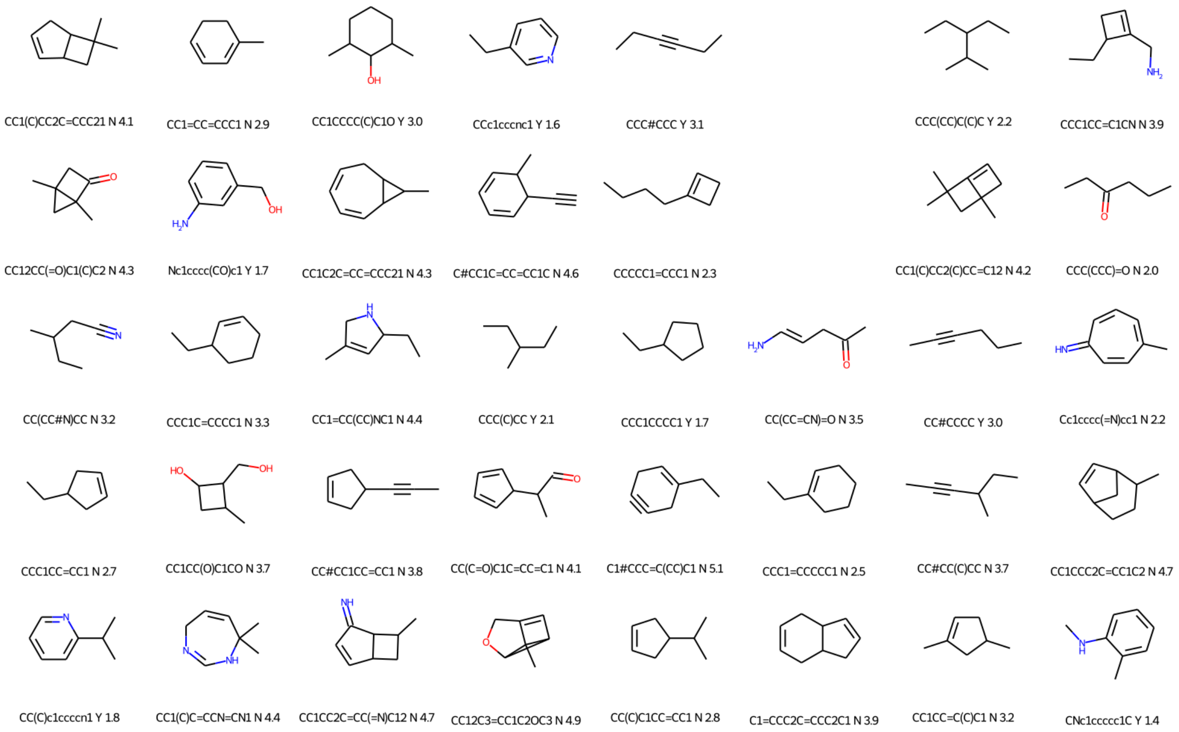

Supplementary Figure 89: Molecules obtained when trying to minimise the overlapping between host (Pd Cage) and guest electron densities, while maximising their electrostatic interactions. $ed\_factor = 0.999$

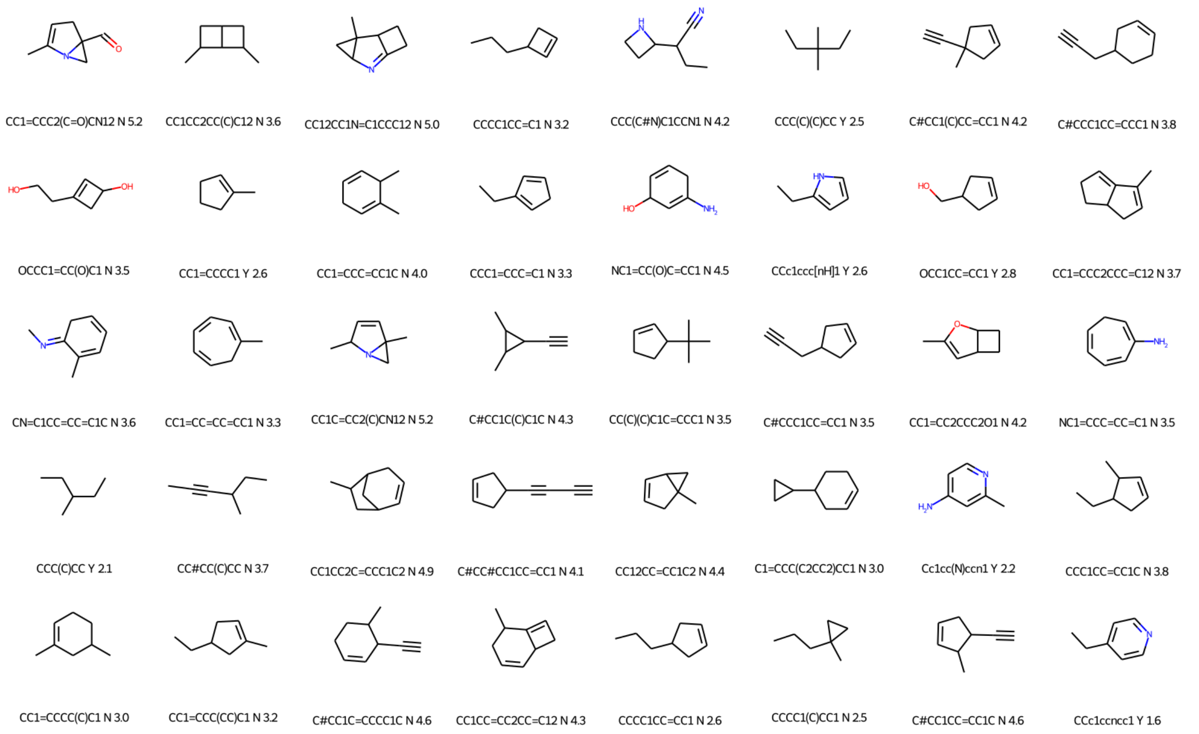

Supplementary Figure 90: Molecules obtained when trying to minimise the overlapping between host (Pd Cage) and guest electron densities, while maximising their electrostatic interactions. $ed\_factor = 1.0$

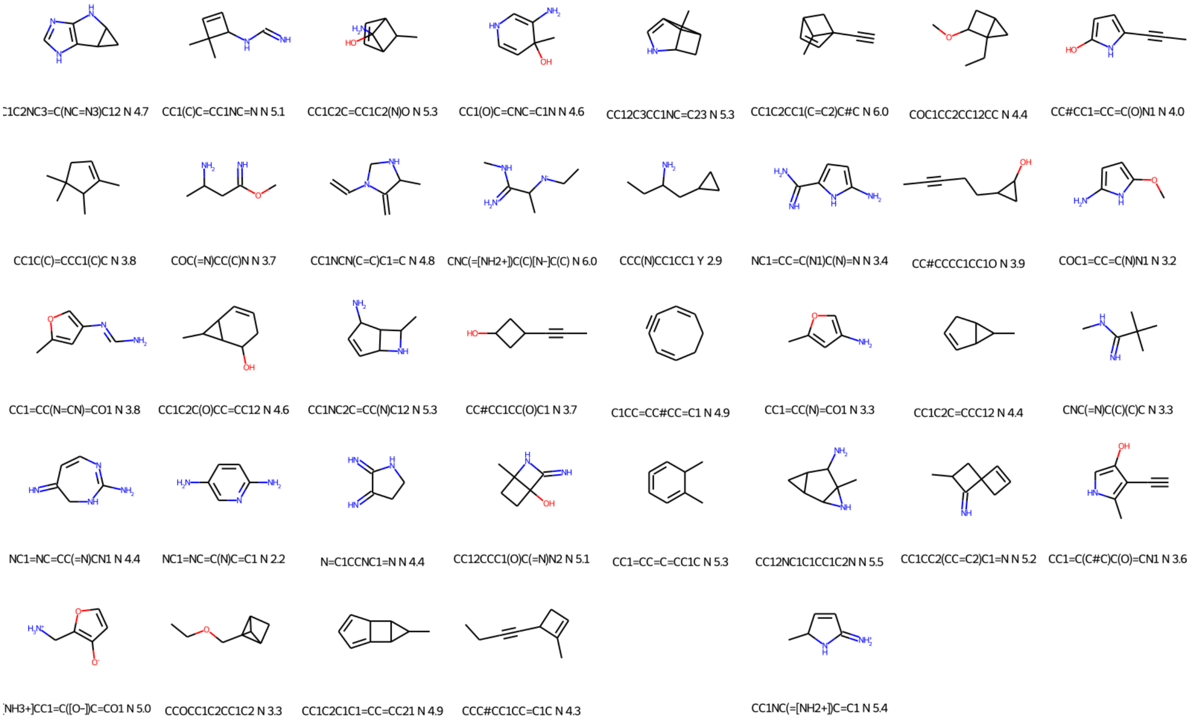

Supplementary Figure 91: Molecules obtained when trying to minimise the overlapping between host (Pd Cage) and guest electron densities, while maximising their electrostatic interactions. The translation from guests to SMILES sequences was done using decorated electron densities. $ed\_factor = 0$

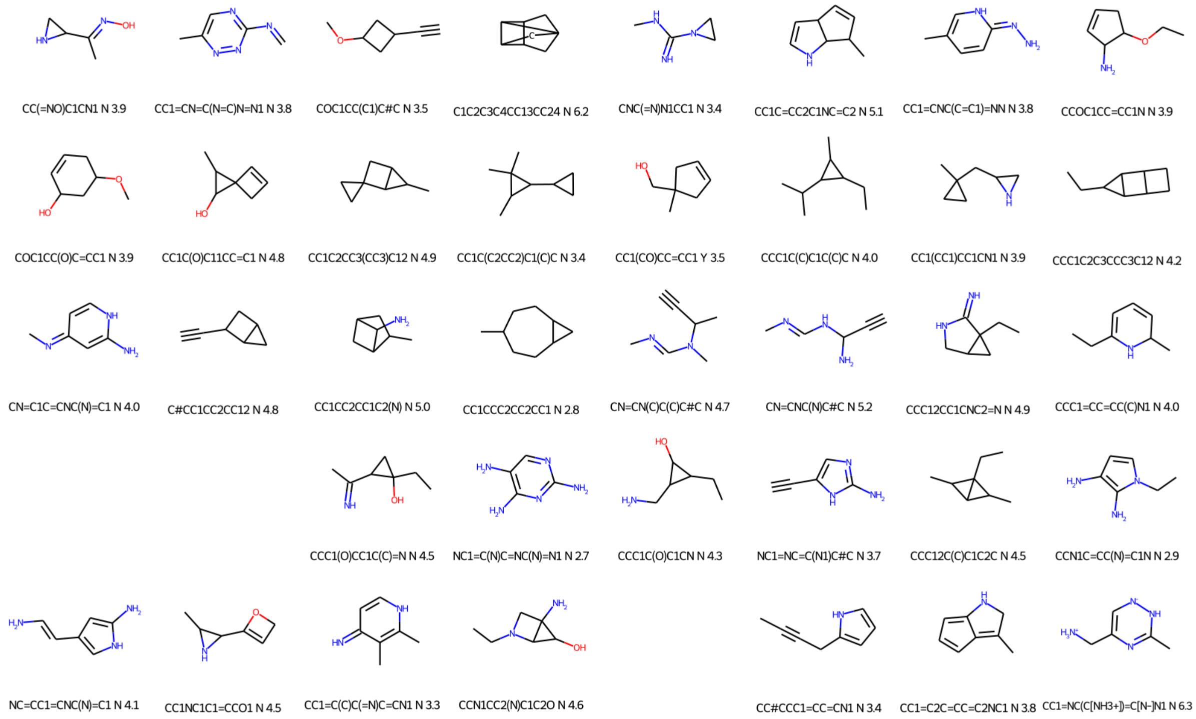

Supplementary Figure 92: Molecules obtained when trying to minimise the overlapping between host (Pd Cage) and guest electron densities, while maximising their electrostatic interactions. The translation from guests to SMILES sequences was done using decorated electron densities. $ed\_factor = 0.001$

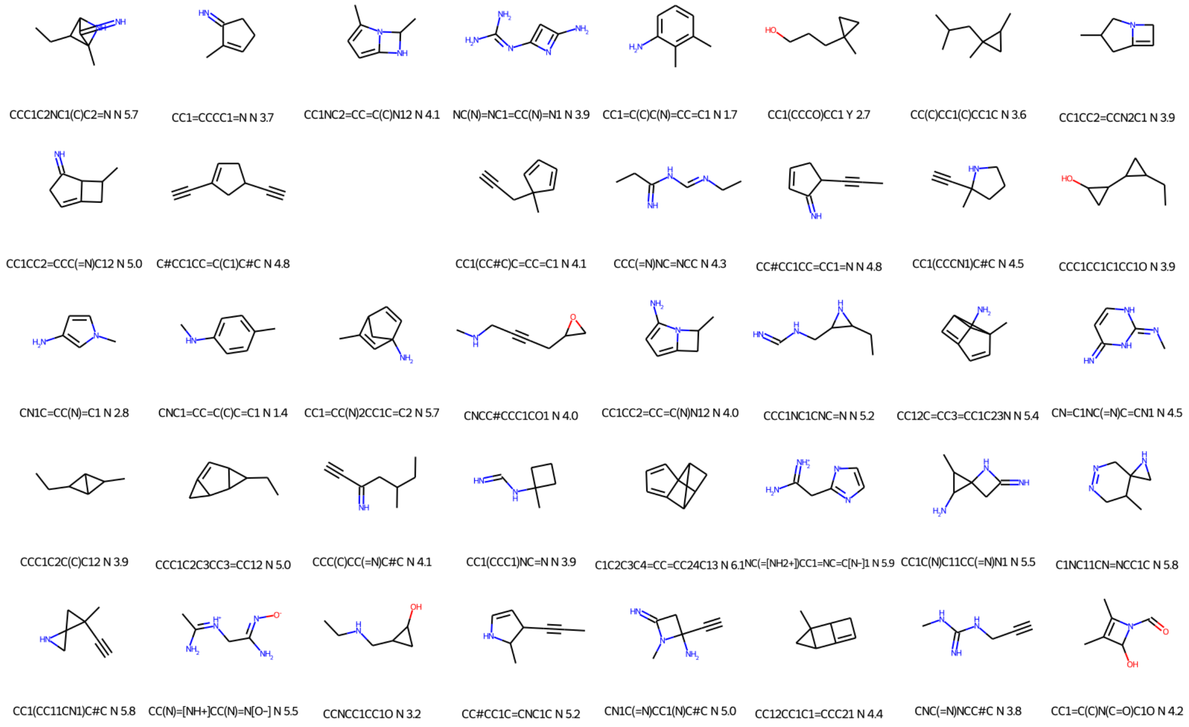

Supplementary Figure 93: Molecules obtained when trying to minimise the overlapping between host (Pd Cage) and guest electron densities, while maximising their electrostatic interactions. The translation from guests to SMILES sequences was done using decorated electron densities. $ed\_factor = 0.05$

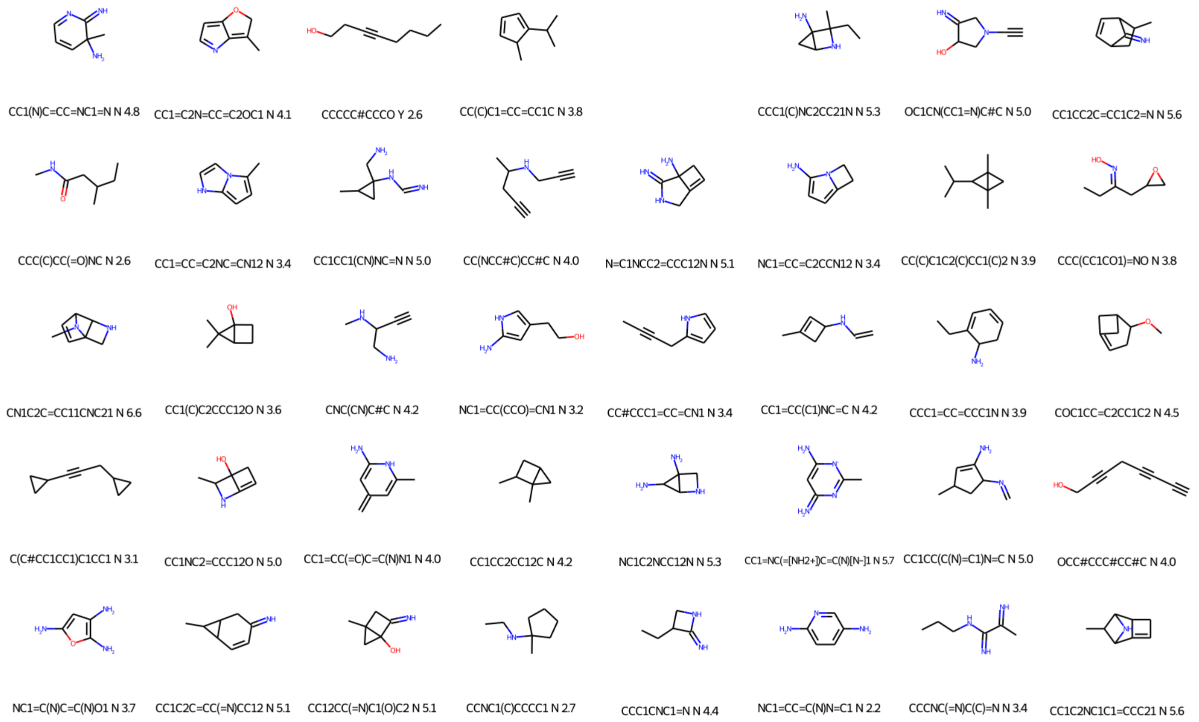

Supplementary Figure 94: Molecules obtained when trying to minimise the overlapping between host (Pd Cage) and guest electron densities, while maximising their electrostatic interactions. The translation from guests to SMILES sequences was done using decorated electron densities. $ed\_factor = 0.1$

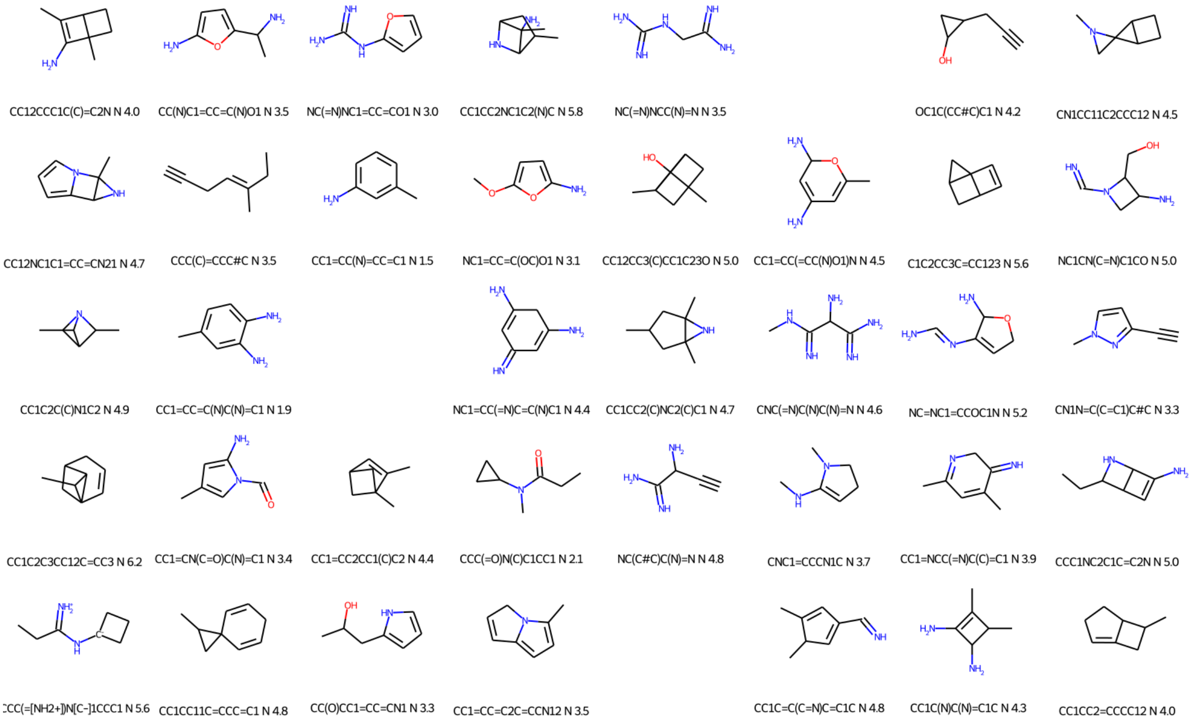

Supplementary Figure 95: Molecules obtained when trying to minimise the overlapping between host (Pd Cage) and guest electron densities, while maximising their electrostatic interactions. The translation from guests to SMILES sequences was done using decorated electron densities. $ed\_factor = 0.2$

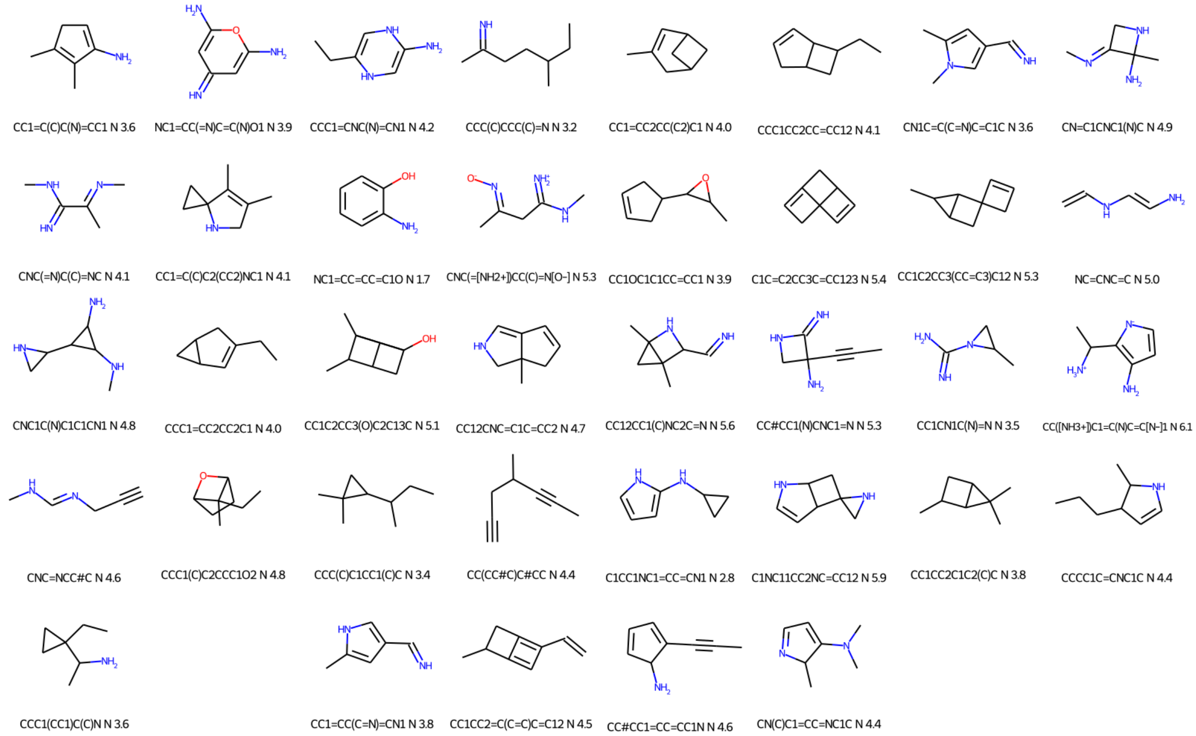

Supplementary Figure 96: Molecules obtained when trying to minimise the overlapping between host (Pd Cage) and guest electron densities, while maximising their electrostatic interactions. The translation from guests to SMILES sequences was done using decorated electron densities. $ed\_factor = 0.3$

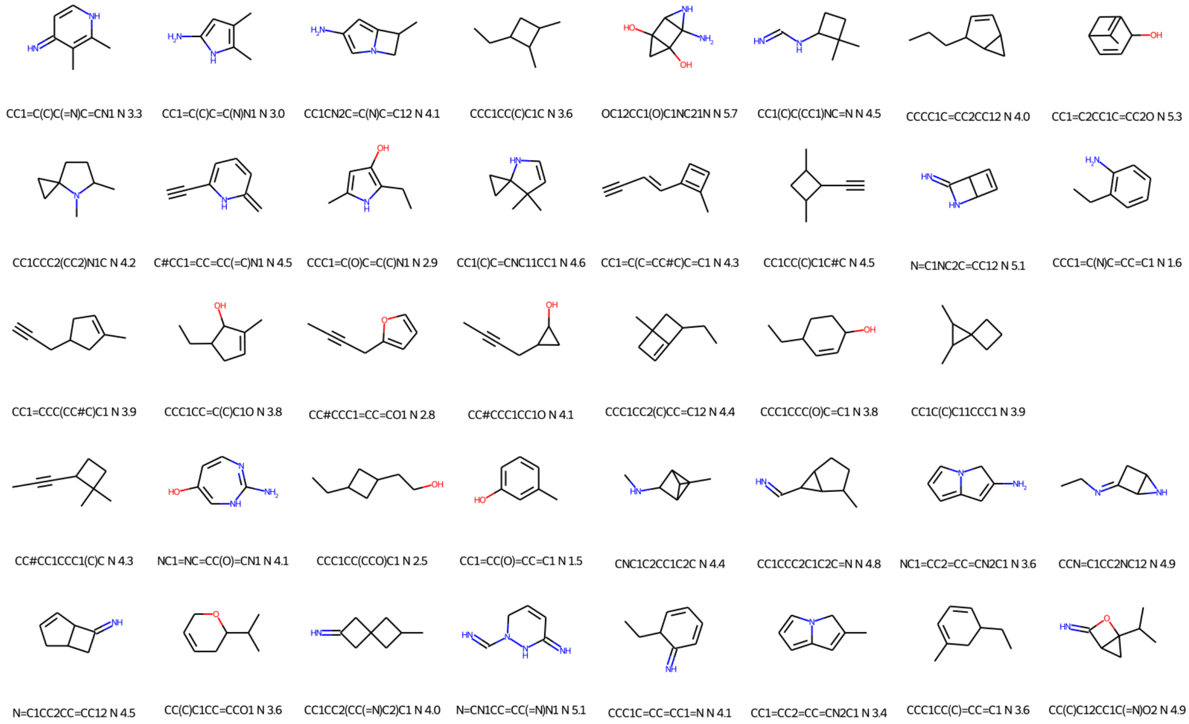

Supplementary Figure 97: Molecules obtained when trying to minimise the overlapping between host (Pd Cage) and guest electron densities, while maximising their electrostatic interactions. The translation from guests to SMILES sequences was done using decorated electron densities. $ed\_factor = 0.5$

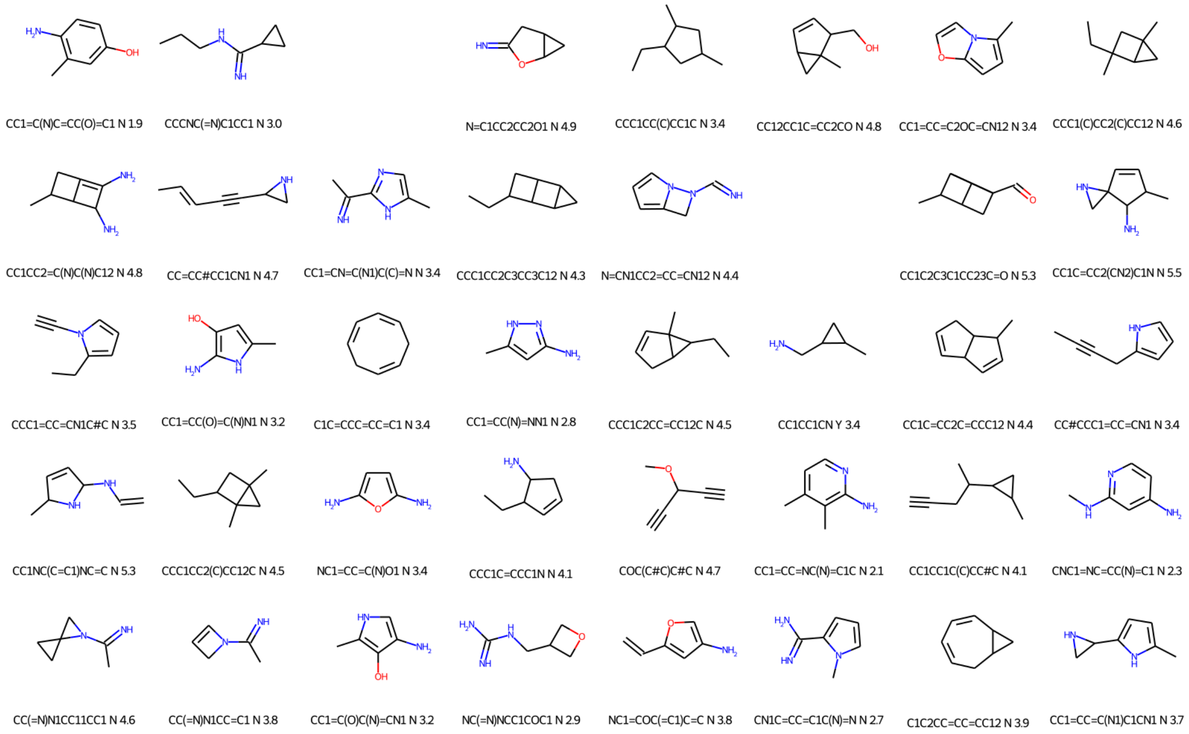

Supplementary Figure 98: Molecules obtained when trying to minimise the overlapping between host (Pd Cage) and guest electron densities, while maximising their electrostatic interactions. The translation from guests to SMILES sequences was done using decorated electron densities. $ed\_factor = 0.7$

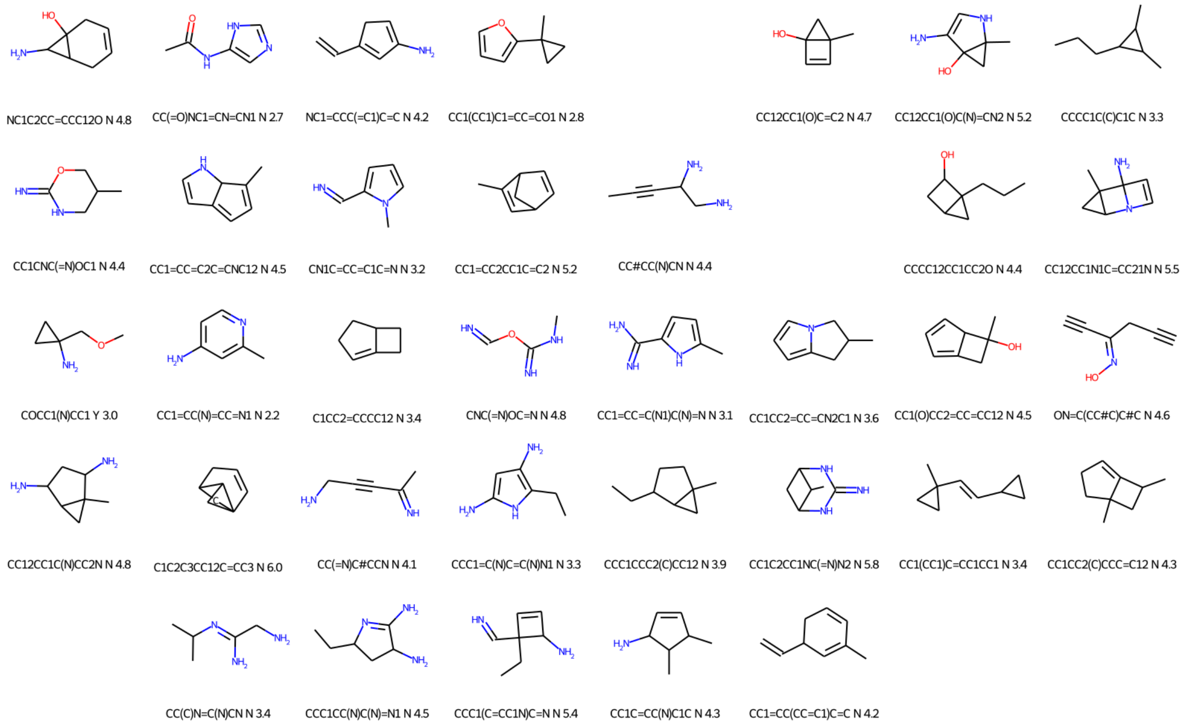

Supplementary Figure 99: Molecules obtained when trying to minimise the overlapping between host (Pd Cage) and guest electron densities, while maximising their electrostatic interactions. The translation from guests to SMILES sequences was done using decorated electron densities. $ed\_factor = 0.8$

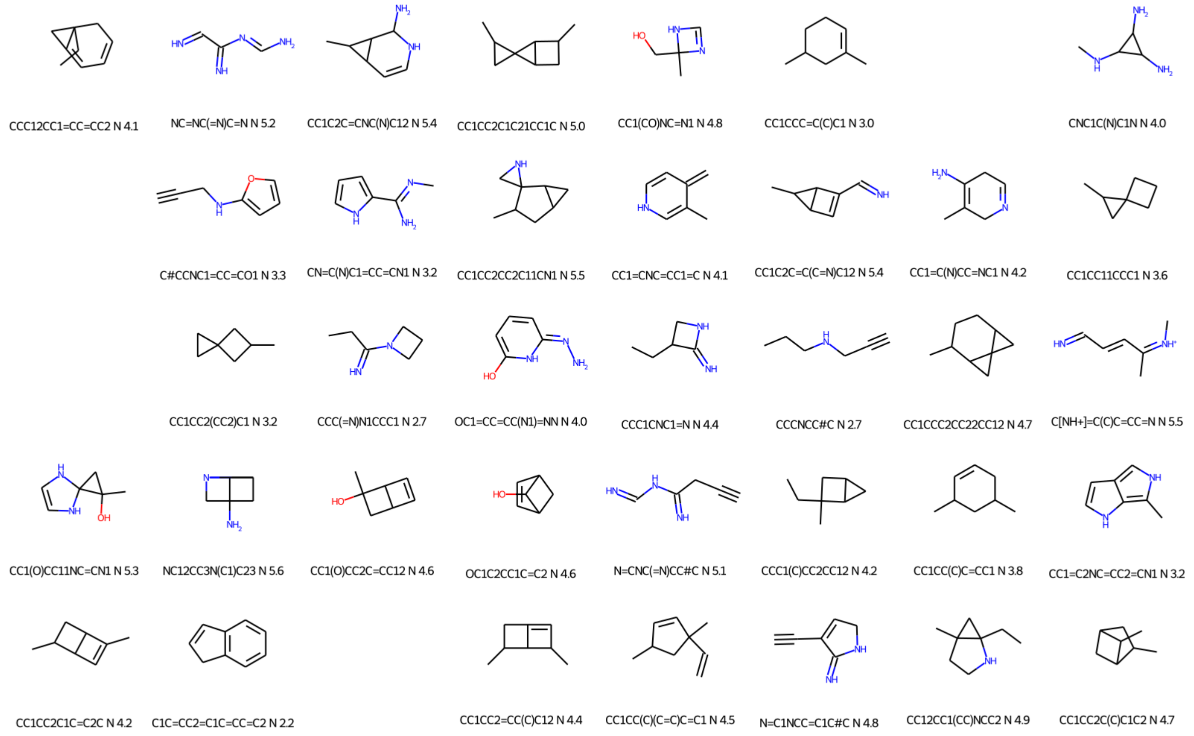

Supplementary Figure 100: Molecules obtained when trying to minimise the overlapping between host (Pd Cage) and guest electron densities, while maximising their electrostatic interactions. The translation from guests to SMILES sequences was done using decorated electron densities. $ed\_factor = 0.9$

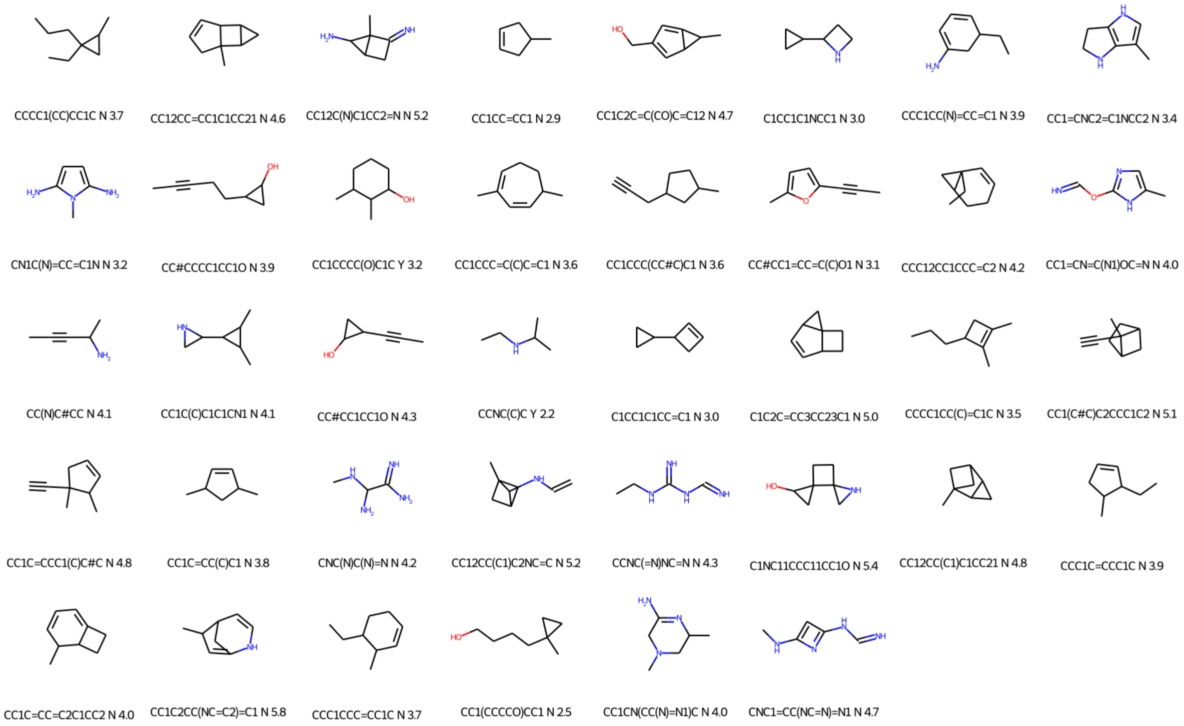

Supplementary Figure 101: Molecules obtained when trying to minimise the overlapping between host (Pd Cage) and guest electron densities, while maximising their electrostatic interactions. The translation from guests to SMILES sequences was done using decorated electron densities. $ed\_factor = 0.95$

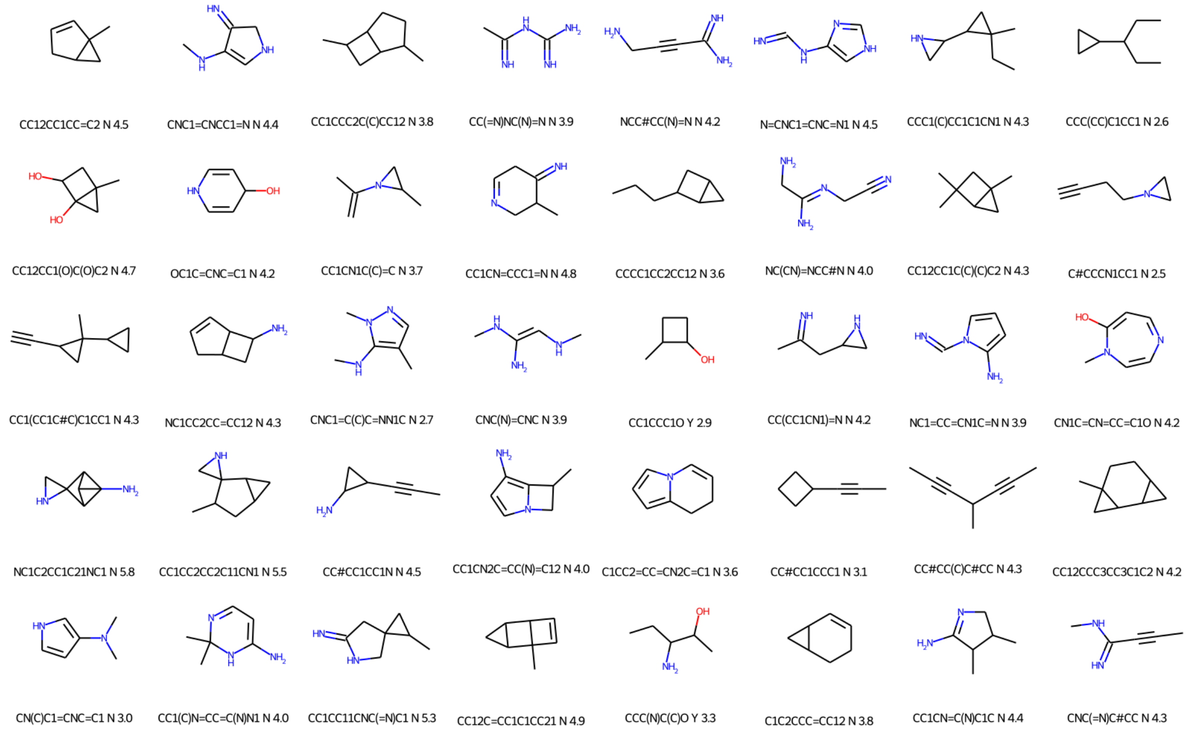

Supplementary Figure 102: Molecules obtained when trying to minimise the overlapping between host (Pd Cage) and guest electron densities, while maximising their electrostatic interactions. The translation from guests to SMILES sequences was done using decorated electron densities. $ed\_factor = 0.999$

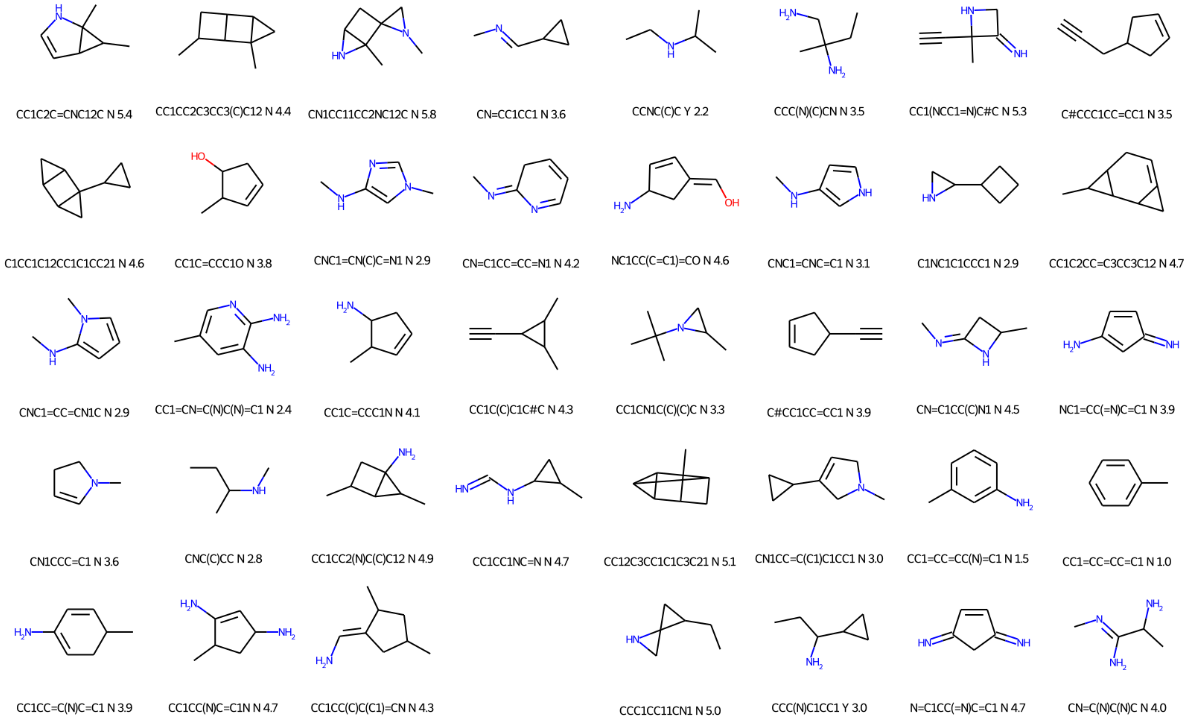

Supplementary Figure 103: Molecules obtained when trying to minimise the overlapping between host (Pd Cage) and guest electron densities, while maximising their electrostatic interactions. The translation from guests to SMILES sequences was done using decorated electron densities. $ed\_factor = 1.0$

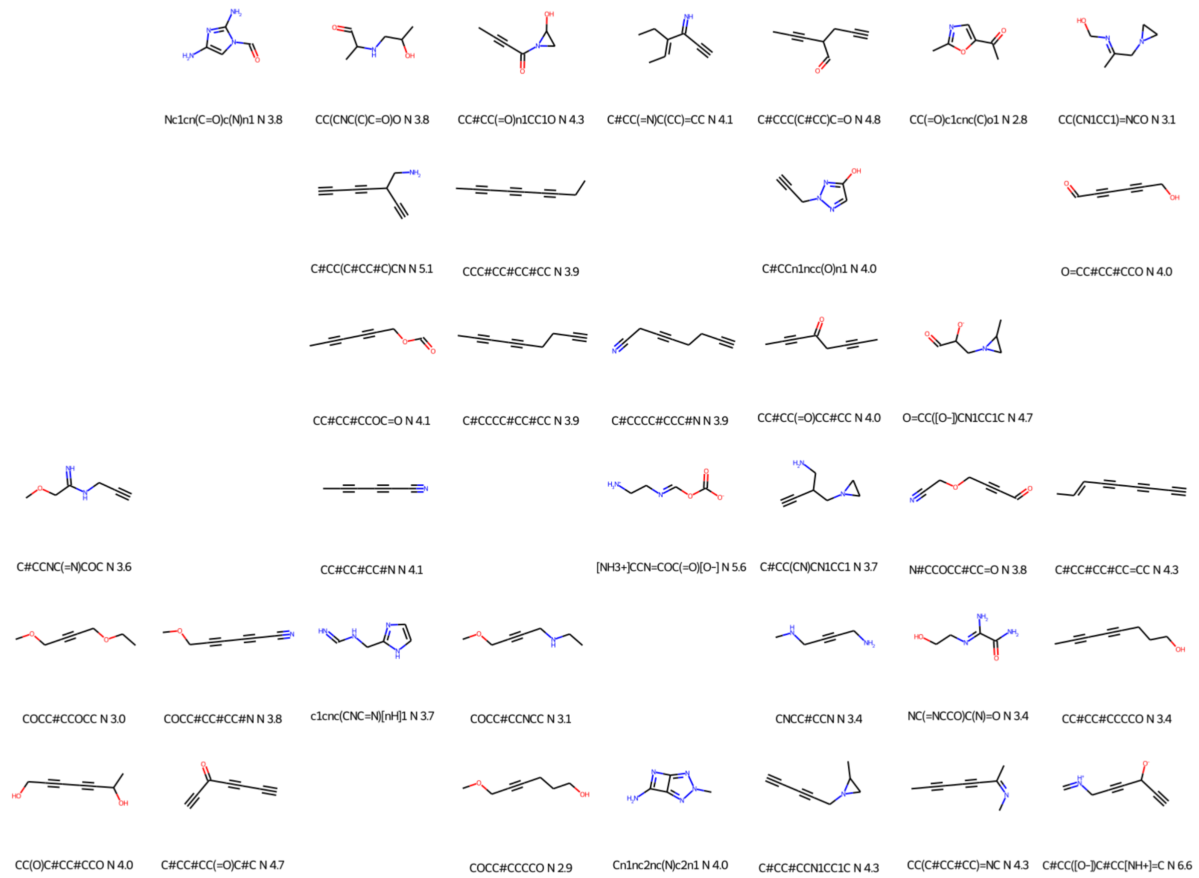

Supplementary Figure 104: Molecules obtained when trying to minimise the overlapping between host (Pd Cage) and guest electron densities, while maximising their electrostatic interactions. $ed\_factor = 0$

2.3.8 Results from bin/optimisers/cage_hg_esp_lee.py

These results were obtained using the `cage_hg_esp_lee.py` script described in Supplementary Section 2.2.8. A total of 32 experiments/runs were done using this script using different values for `ed_factor`. This generated 1384 different guests. These were converted into SMILES sequences using the two methods based on electron density data described in Supplementary Section 1.11, plus the method using decorated electron densities described in Supplementary Section 1.8. Therefore, each 3D tensor representing a guest could output three slightly different molecules. Thus, the total number of molecules generated was 3528. Since this is a very big number, we will only show some of there here. The results can be seen in Supplementary Figures 104 to 129.

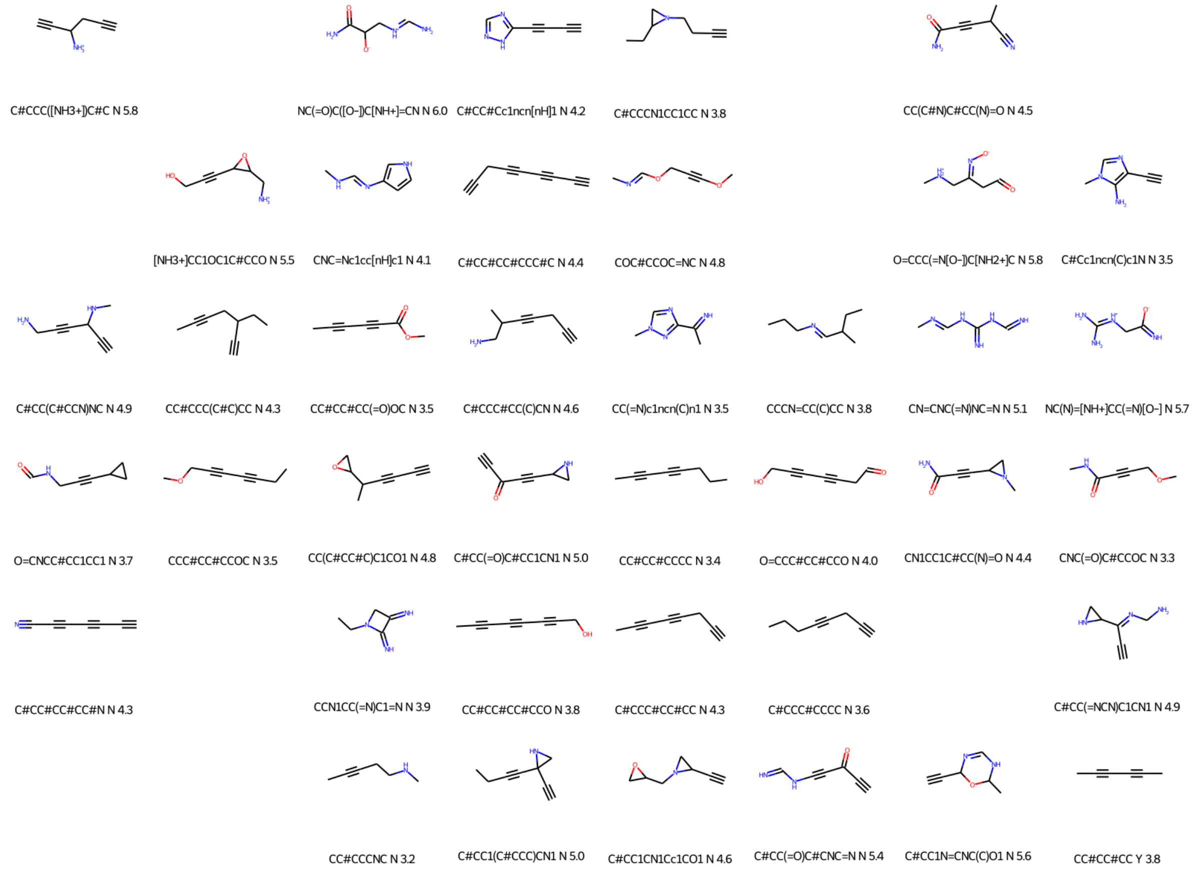

Supplementary Figure 105: Molecules obtained when trying to minimise the overlapping between host (Pd Cage) and guest electron densities, while maximising their electrostatic interactions. $ed\_factor = 0.05$

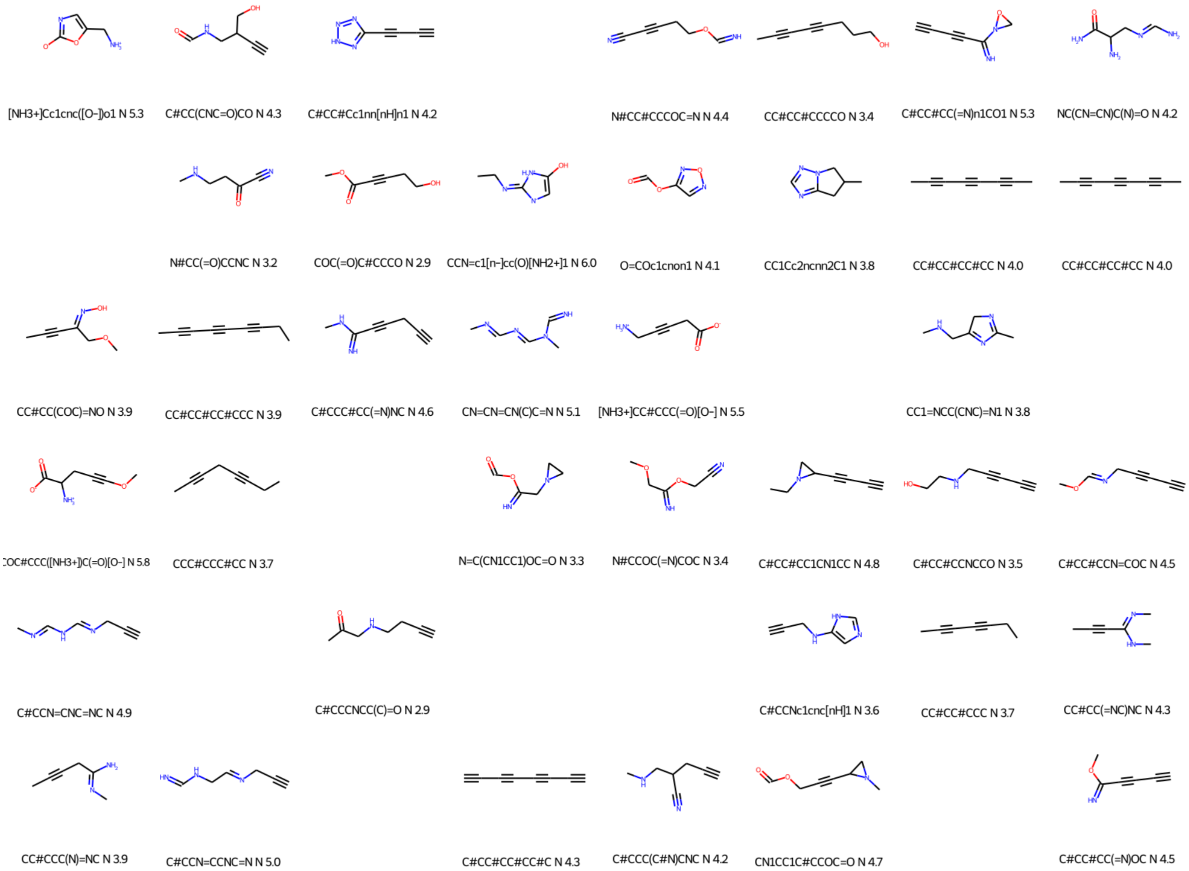

Supplementary Figure 106: Molecules obtained when trying to minimise the overlapping between host (Pd Cage) and guest electron densities, while maximising their electrostatic interactions. $ed\_factor = 0.1$

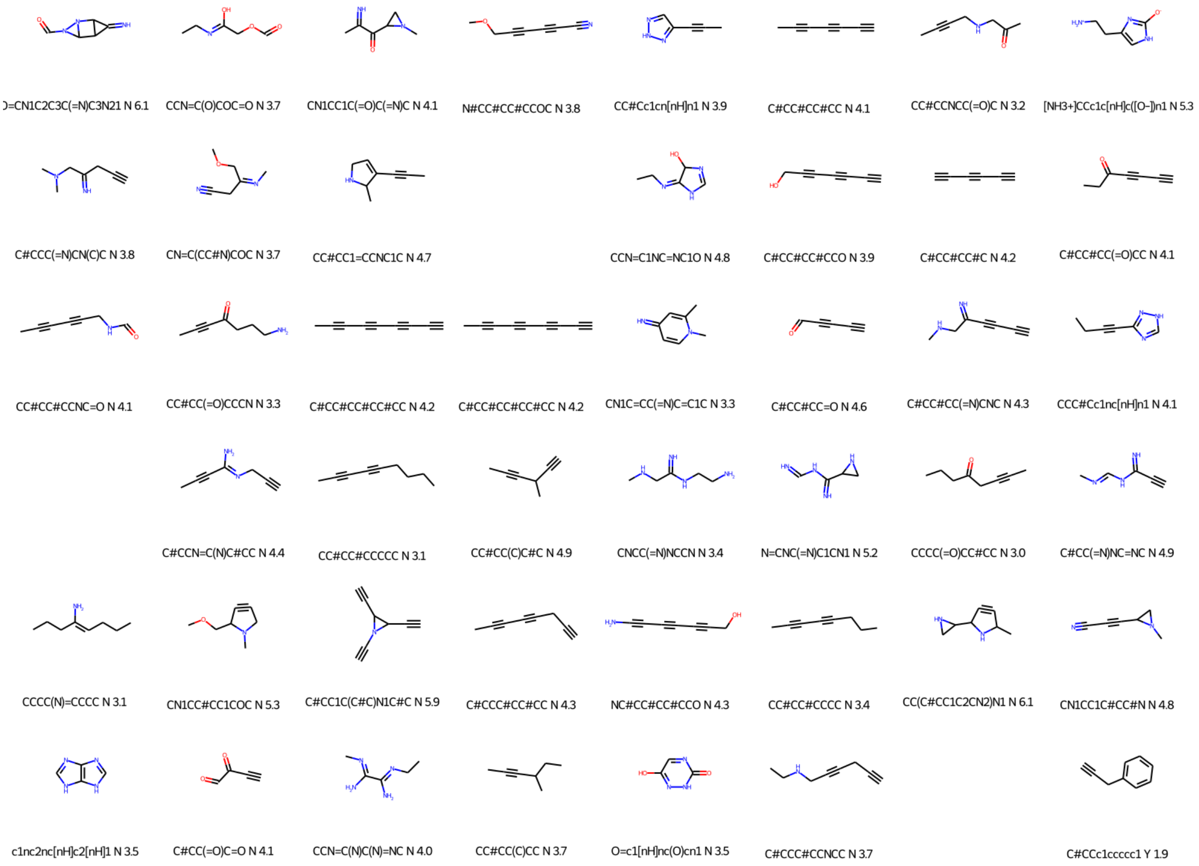

Supplementary Figure 107: Molecules obtained when trying to minimise the overlapping between host (Pd Cage) and guest electron densities, while maximising their electrostatic interactions. $ed\_factor = 0.2$

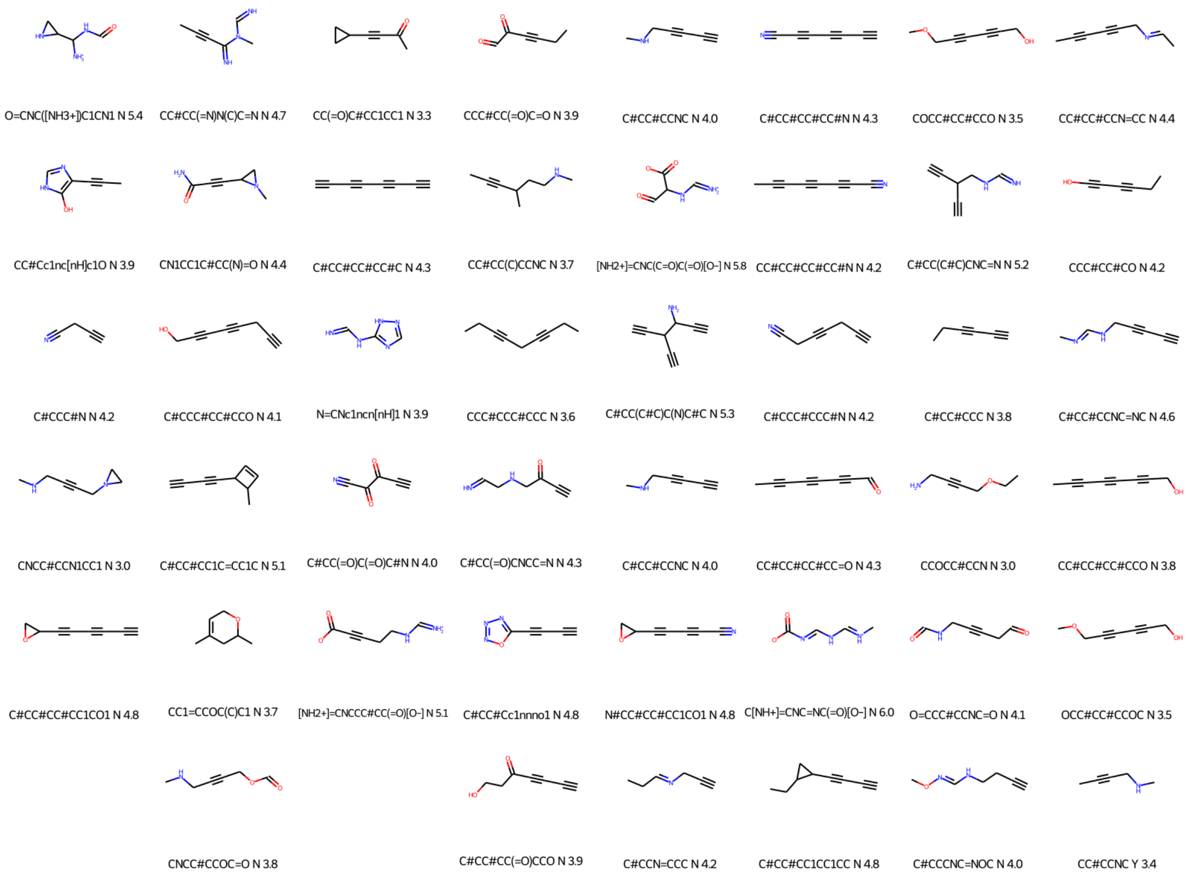

Supplementary Figure 108: Molecules obtained when trying to minimise the overlapping between host (Pd Cage) and guest electron densities, while maximising their electrostatic interactions. $ed\_factor = 0.3$

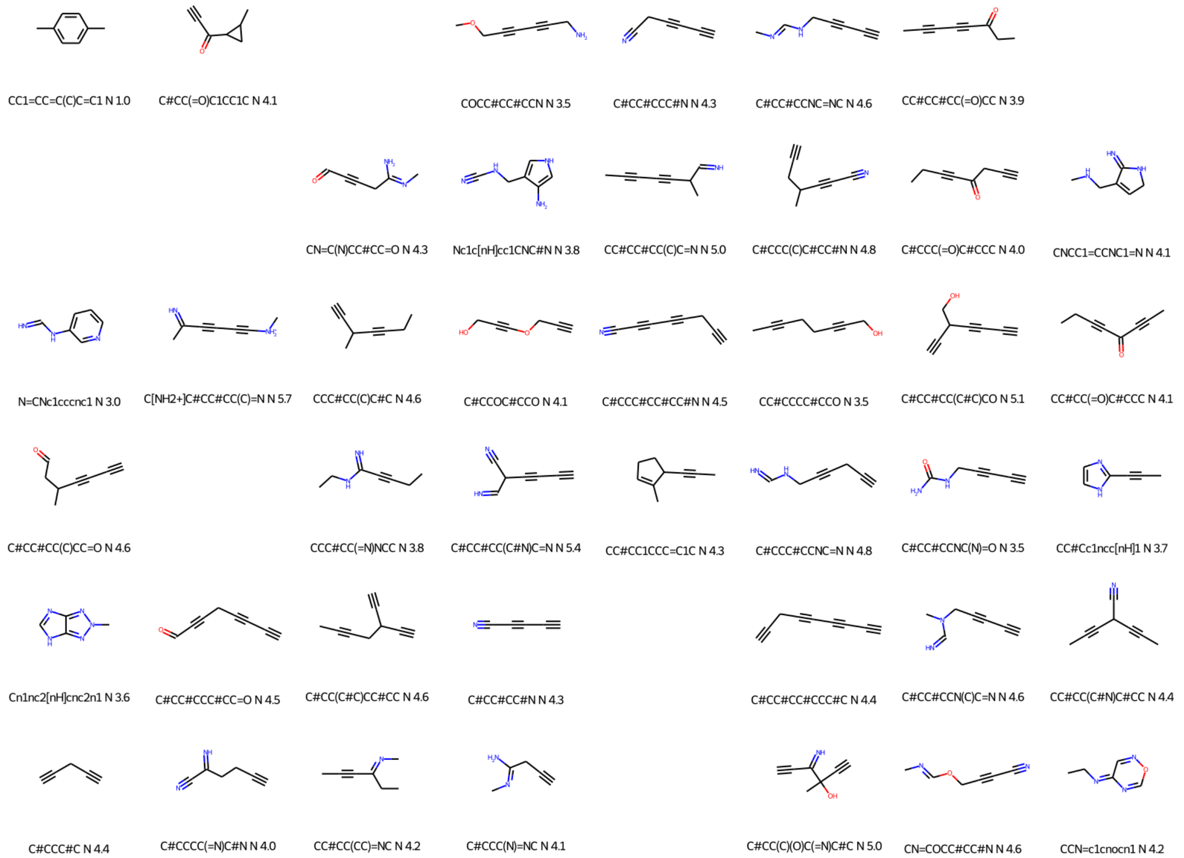

Supplementary Figure 109: Molecules obtained when trying to minimise the overlapping between host (Pd Cage) and guest electron densities, while maximising their electrostatic interactions. $ed\_factor = 0.4$

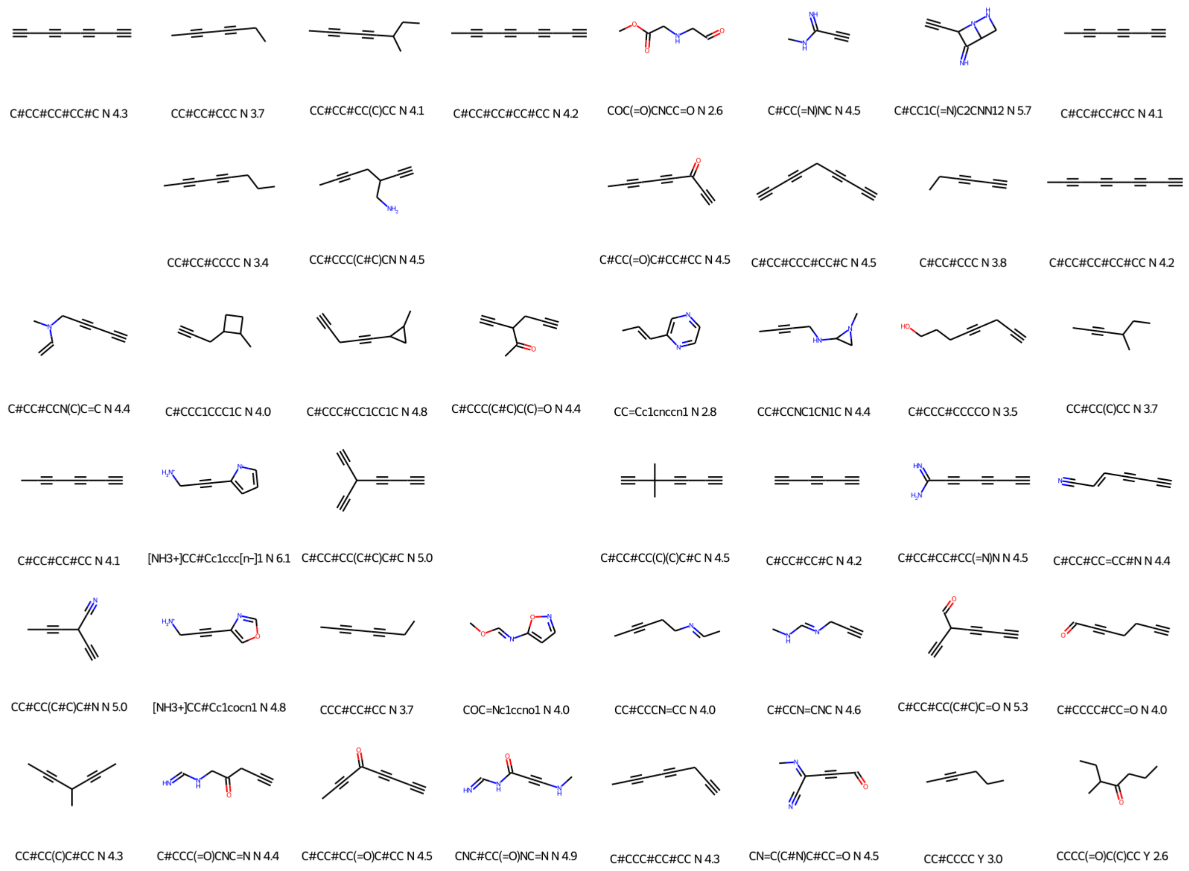

Supplementary Figure 110: Molecules obtained when trying to minimise the overlapping between host (Pd Cage) and guest electron densities, while maximising their electrostatic interactions. $ed\_factor = 0.5$

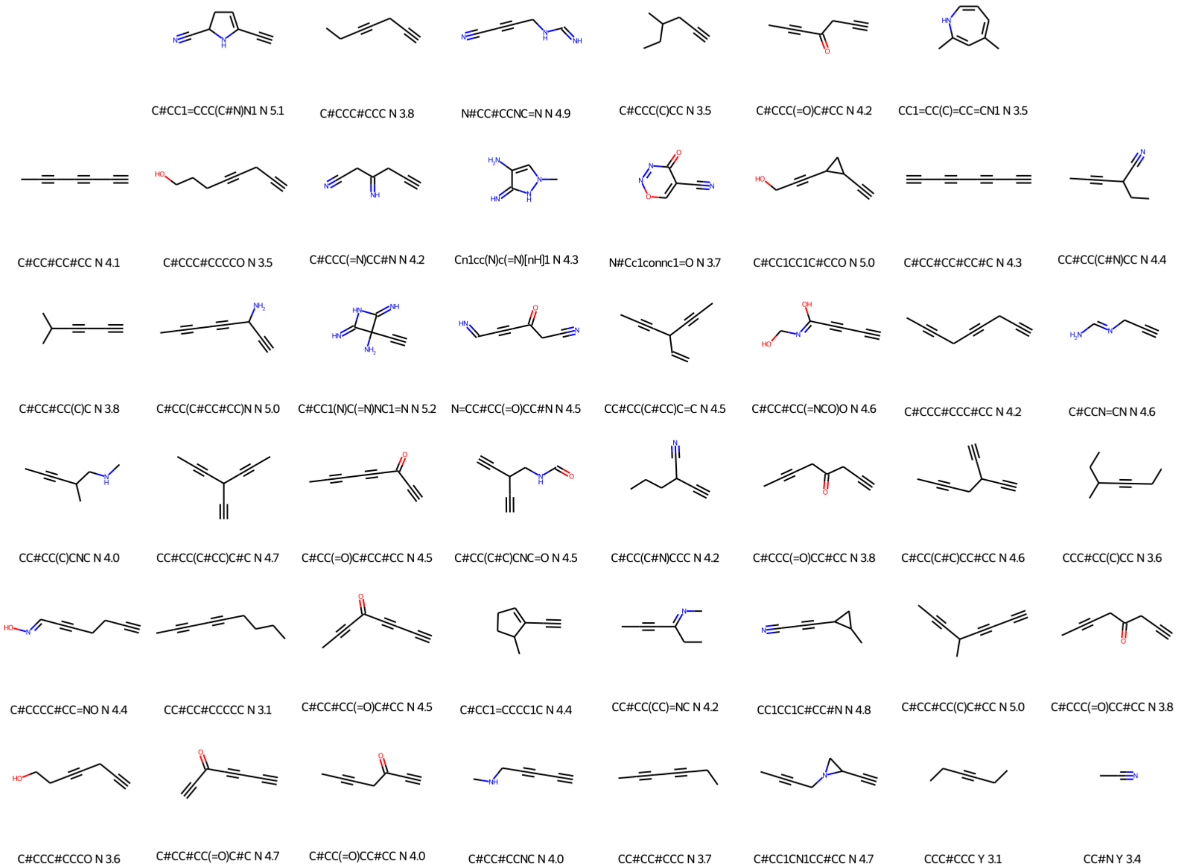

Supplementary Figure 111: Molecules obtained when trying to minimise the overlapping between host (Pd Cage) and guest electron densities, while maximising their electrostatic interactions. $ed\_factor = 0.6$

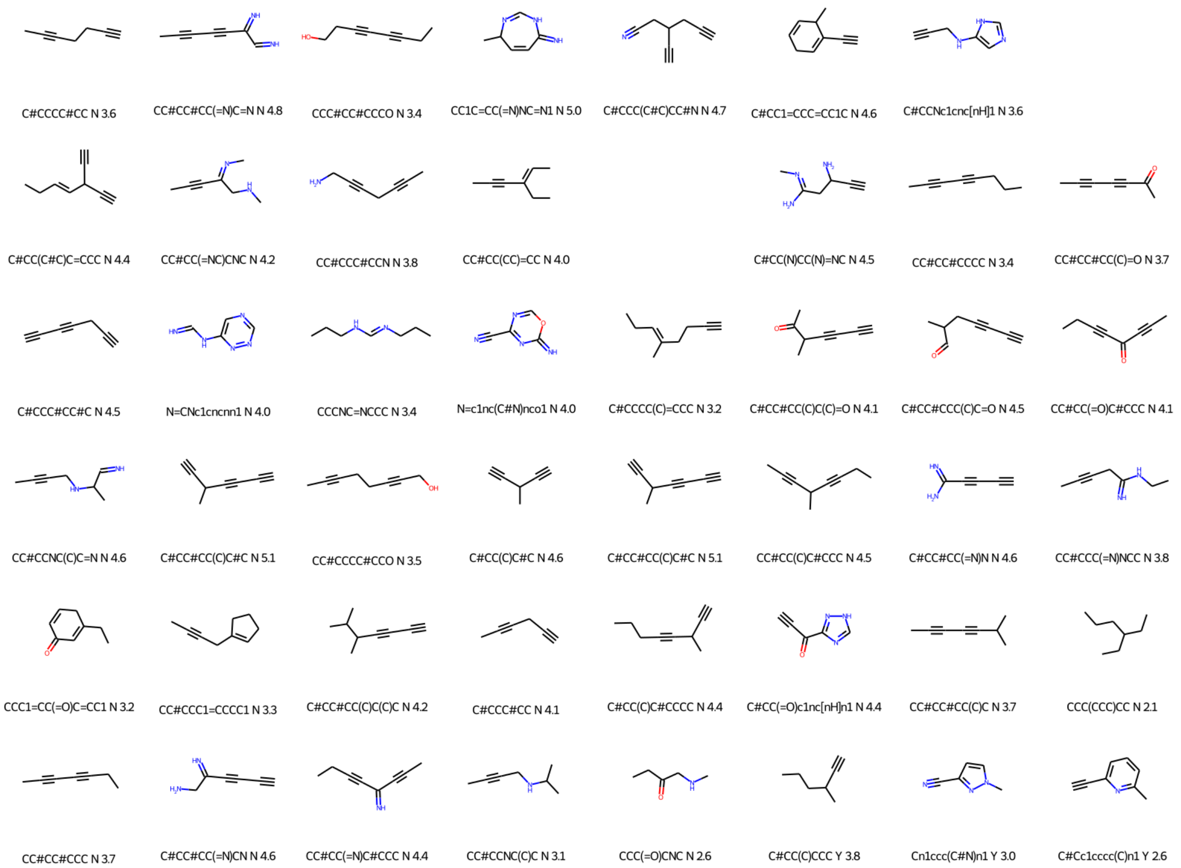

Supplementary Figure 112: Molecules obtained when trying to minimise the overlapping between host (Pd Cage) and guest electron densities, while maximising their electrostatic interactions. $ed\_factor = 0.7$

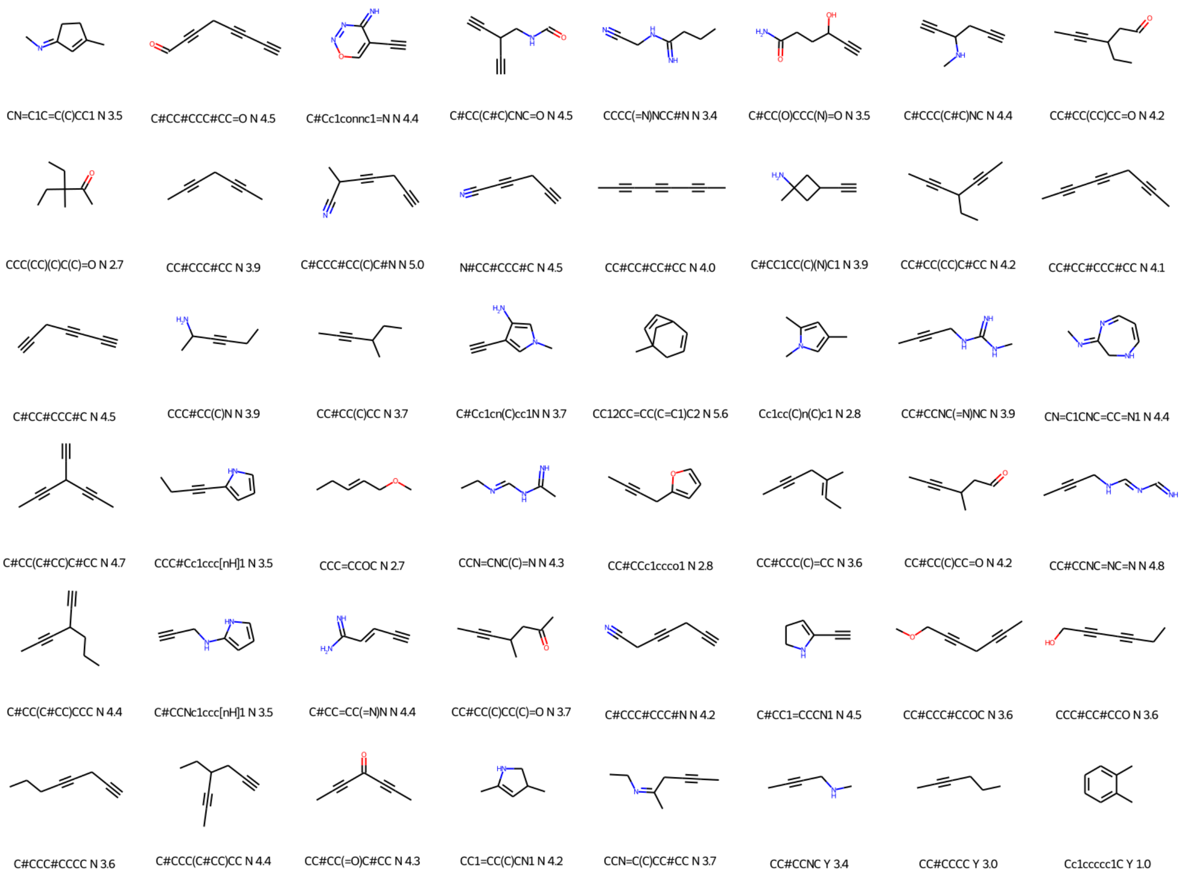

Supplementary Figure 113: Molecules obtained when trying to minimise the overlapping between host (Pd Cage) and guest electron densities, while maximising their electrostatic interactions. $ed\_factor = 0.8$

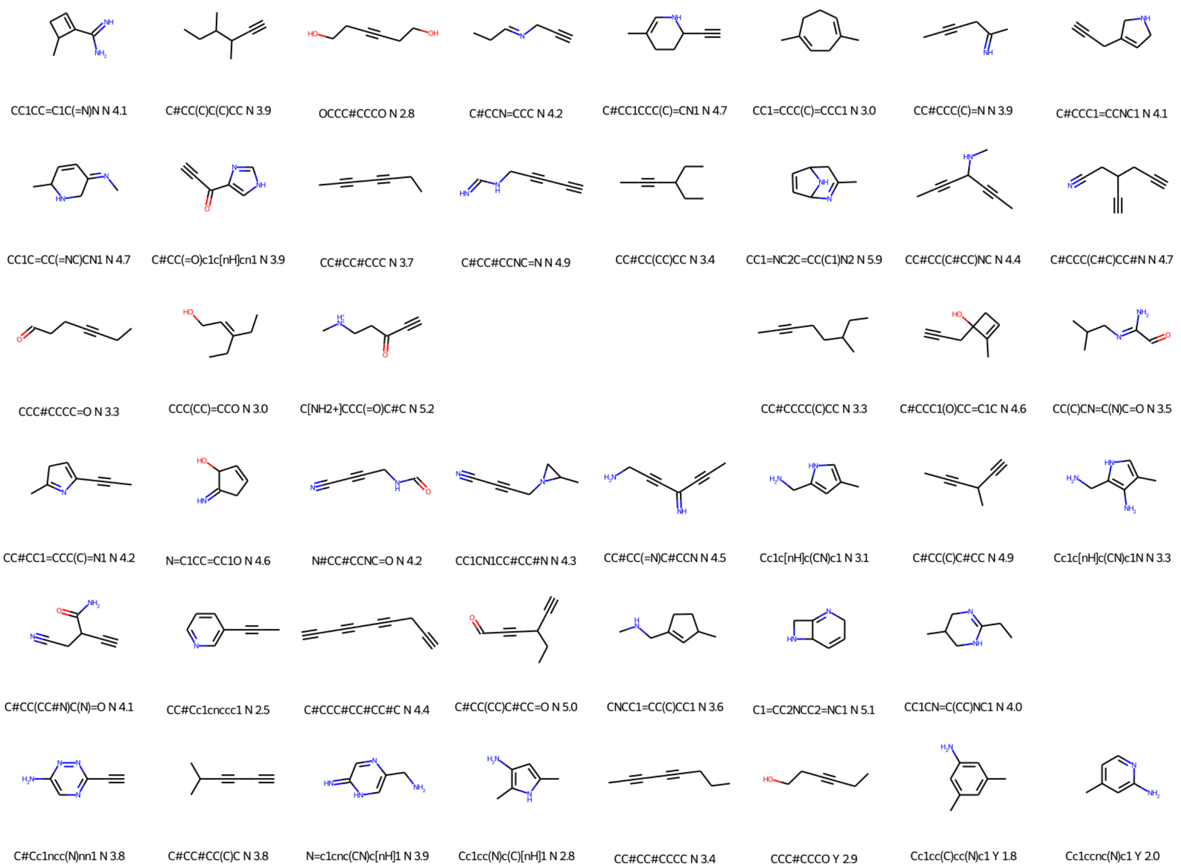

Supplementary Figure 114: Molecules obtained when trying to minimise the overlapping between host (Pd Cage) and guest electron densities, while maximising their electrostatic interactions. $ed\_factor = 0.9$

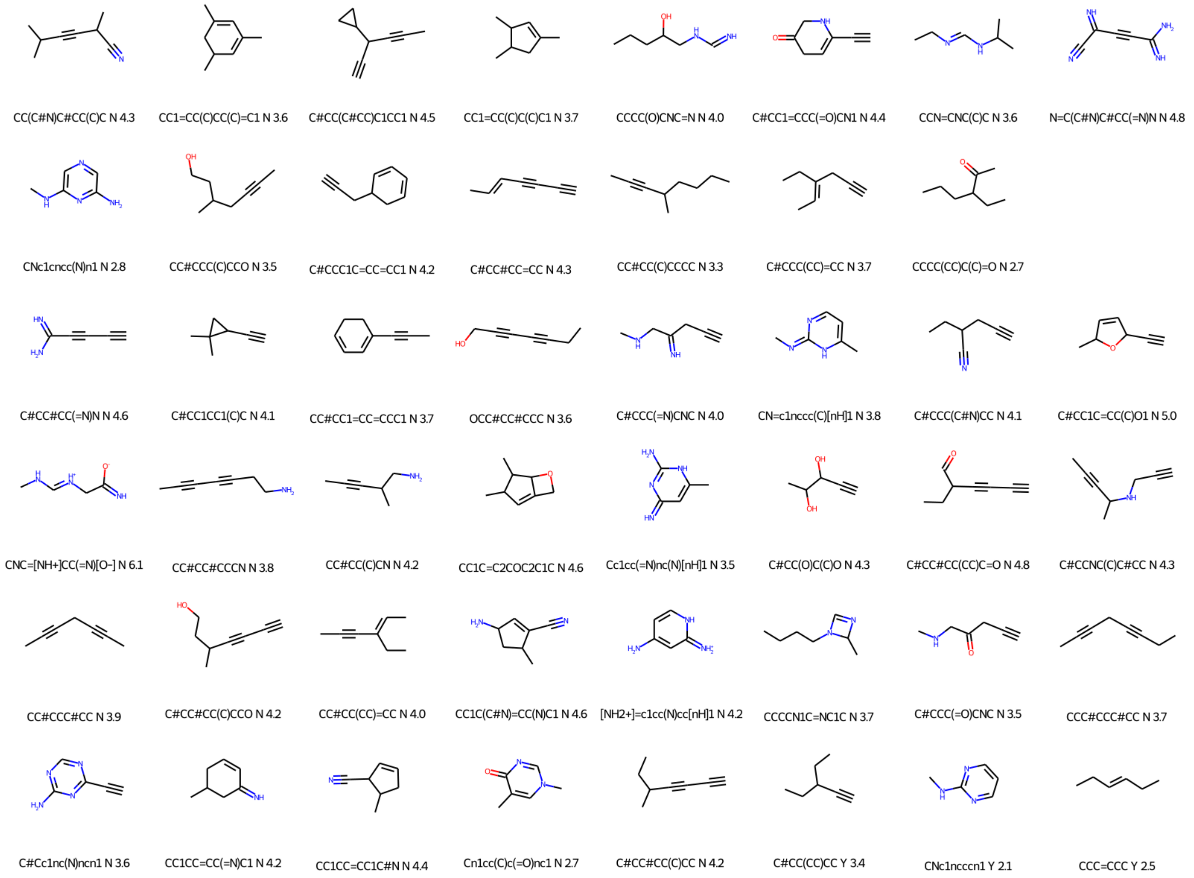

Supplementary Figure 115: Molecules obtained when trying to minimise the overlapping between host (Pd Cage) and guest electron densities, while maximising their electrostatic interactions. $ed\_factor = 0.95$

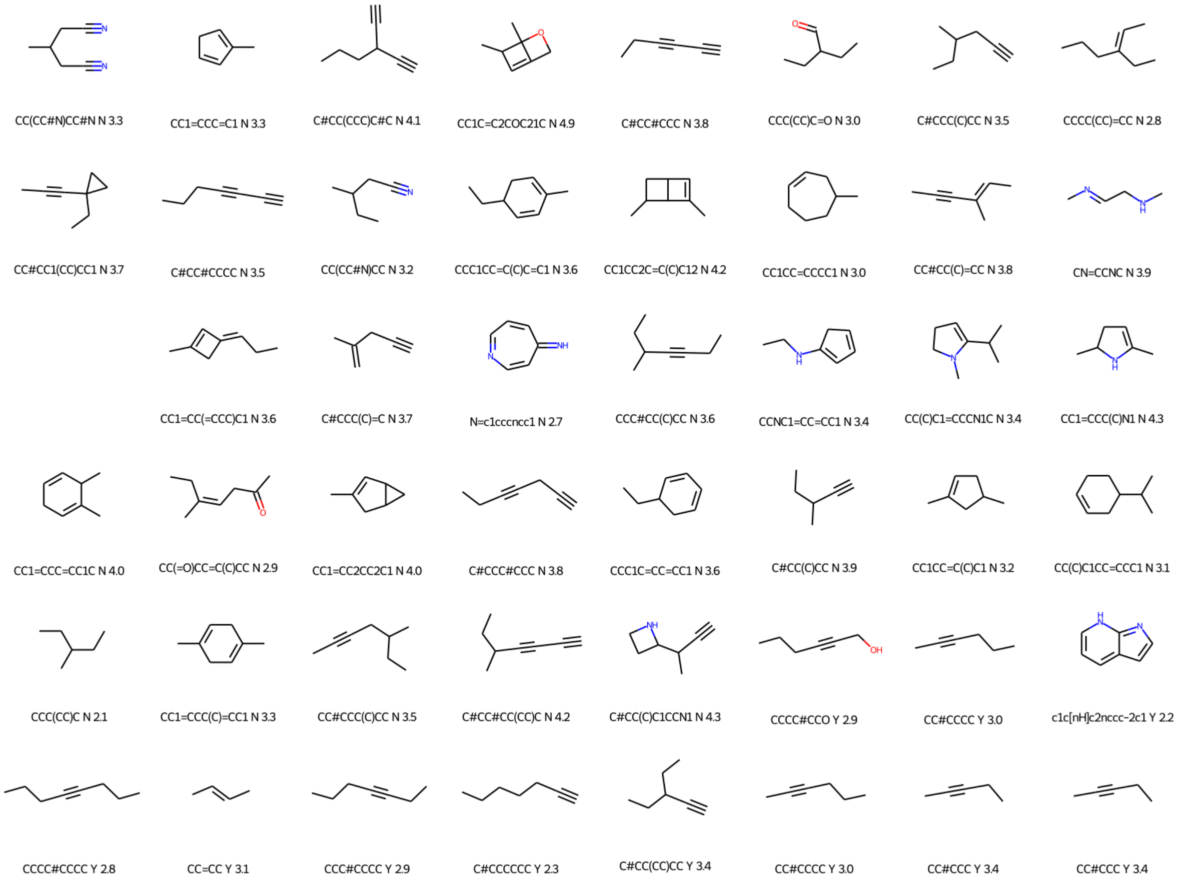

Supplementary Figure 116: Molecules obtained when trying to minimise the overlapping between host (Pd Cage) and guest electron densities, while maximising their electrostatic interactions. $ed\_factor = 1.0$

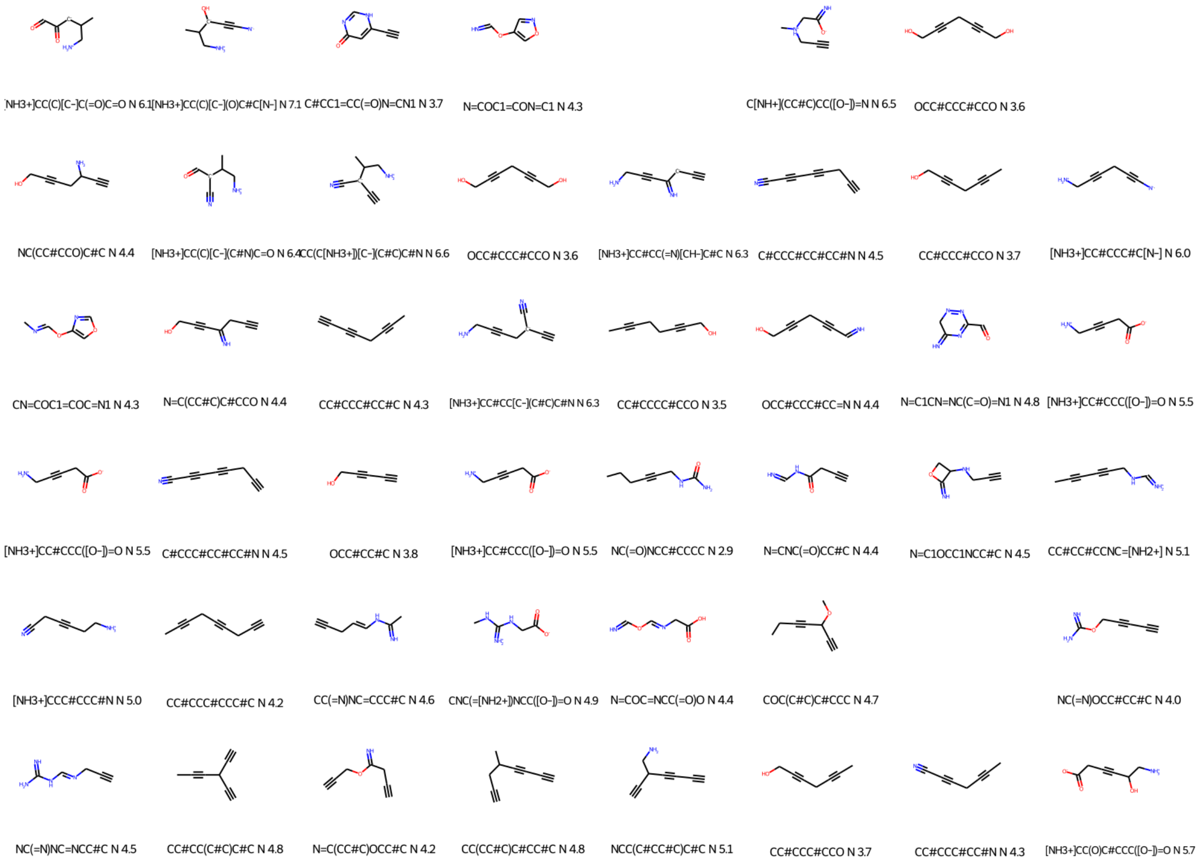

Supplementary Figure 117: Molecules obtained when trying to minimise the overlapping between host (Pd Cage) and guest electron densities, while maximising their electrostatic interactions. The translation from guests to SMILES sequences was done using decorated electron densities. $ed\_factor = 0$

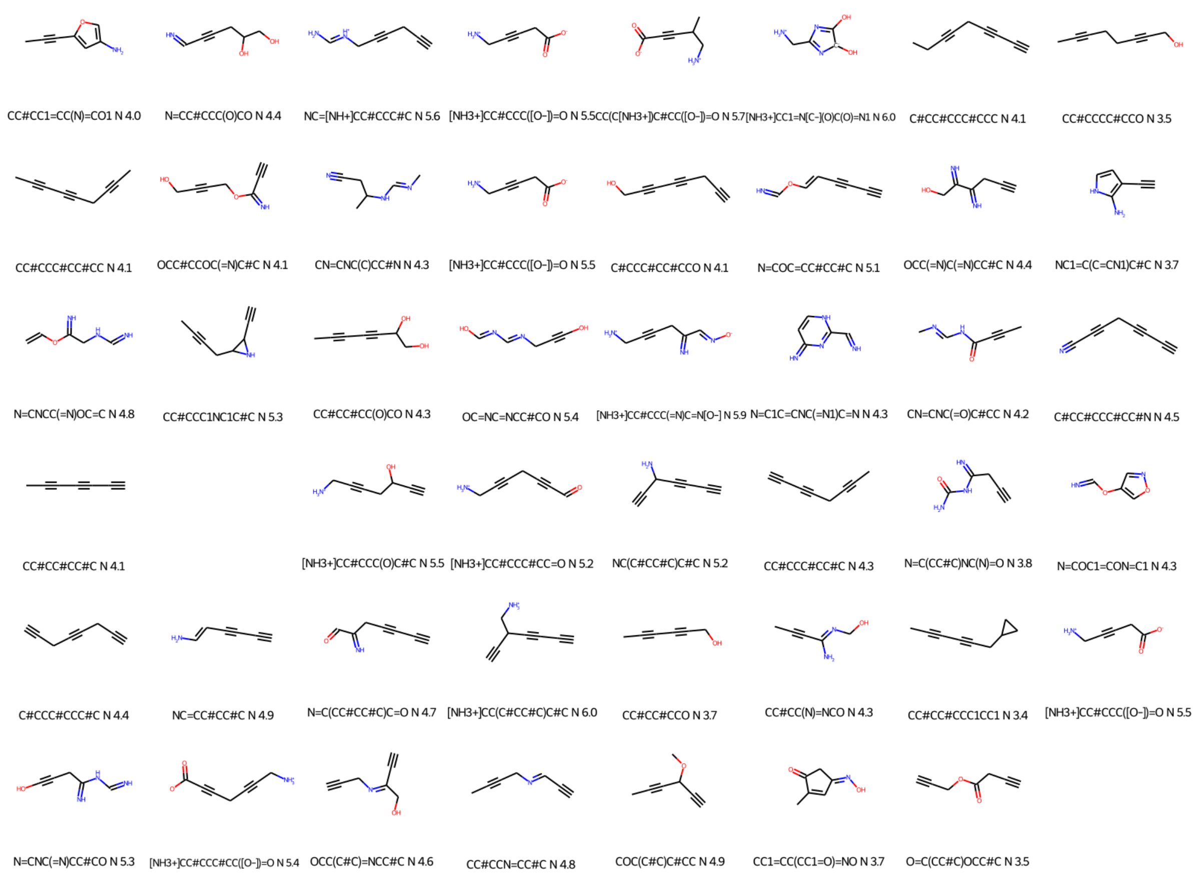

Supplementary Figure 118: Molecules obtained when trying to minimise the overlapping between host (Pd Cage) and guest electron densities, while maximising their electrostatic interactions. The translation from guests to SMILES sequences was done using decorated electron densities. $ed\_factor = 0.05$

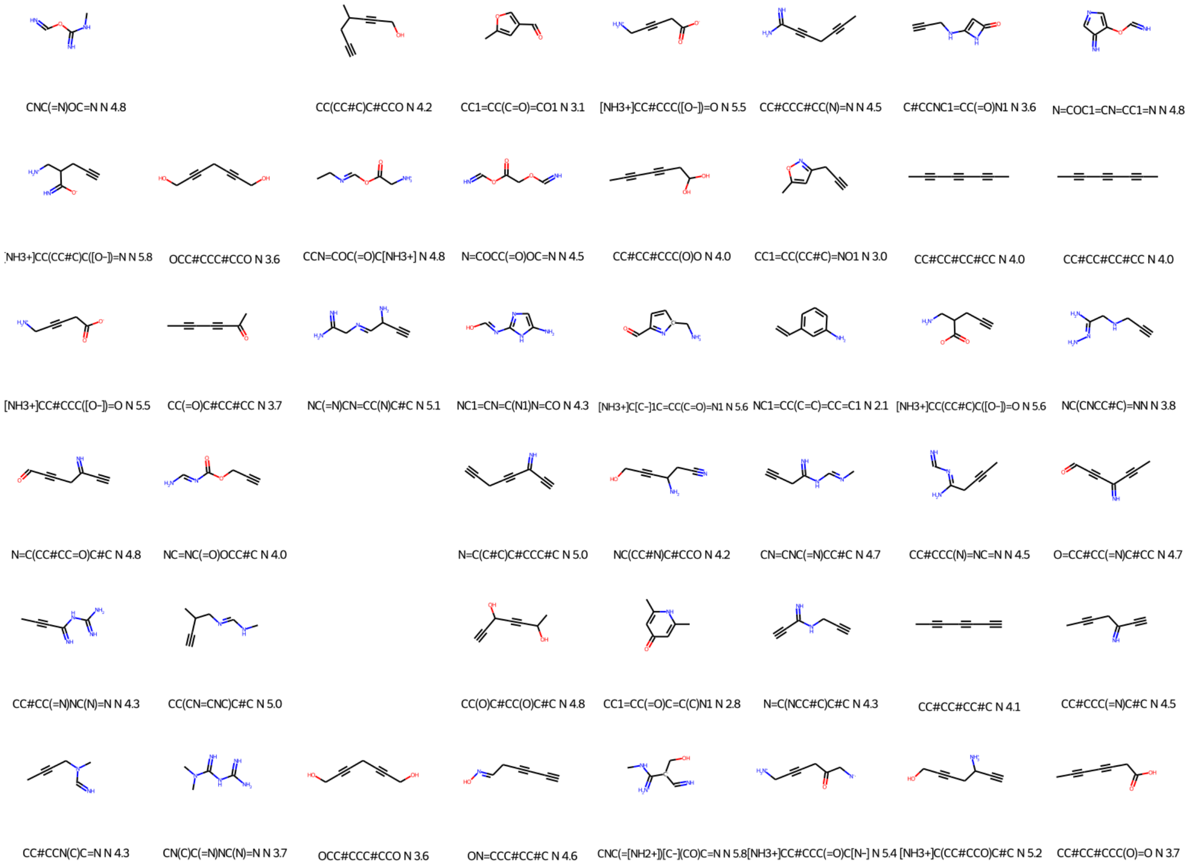

Supplementary Figure 119: Molecules obtained when trying to minimise the overlapping between host (Pd Cage) and guest electron densities, while maximising their electrostatic interactions. The translation from guests to SMILES sequences was done using decorated electron densities. $ed\_factor = 0.1$

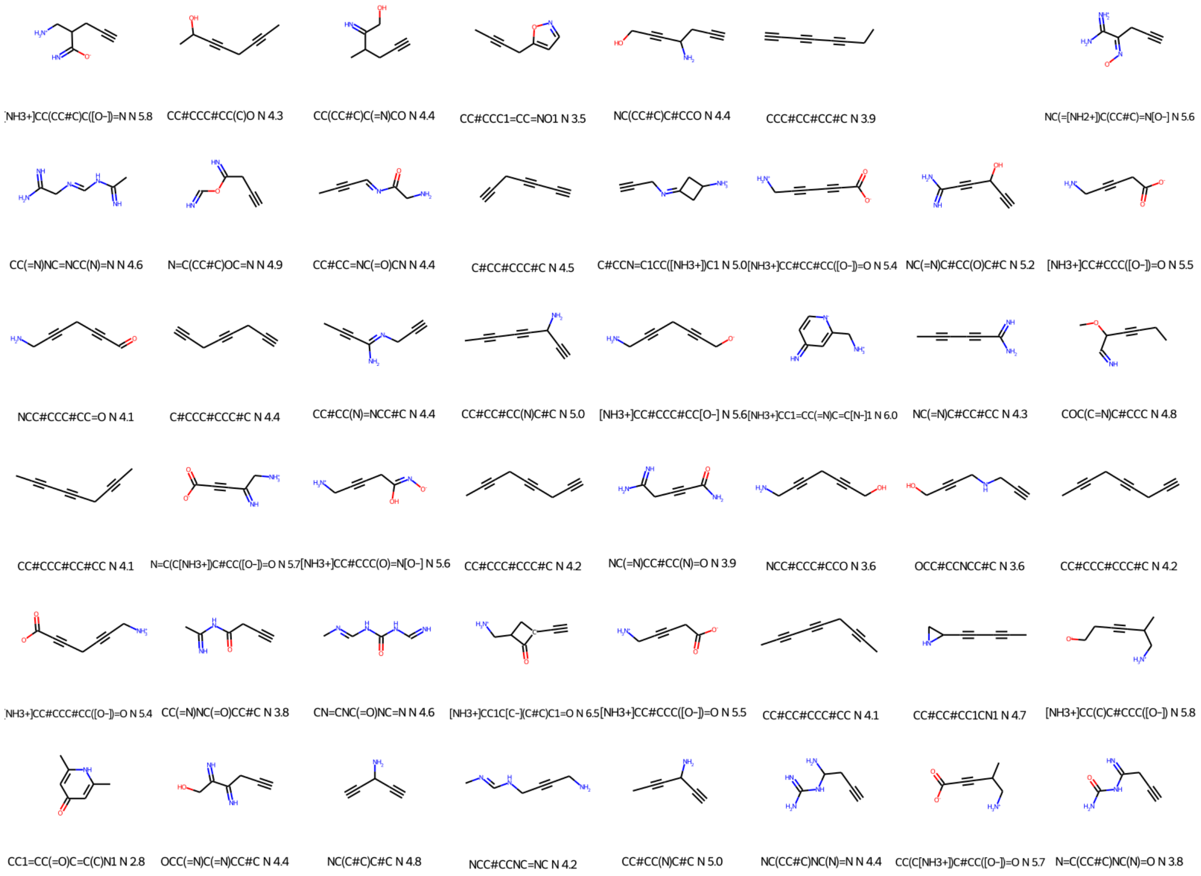

Supplementary Figure 120: Molecules obtained when trying to minimise the overlapping between host (Pd Cage) and guest electron densities, while maximising their electrostatic interactions. The translation from guests to SMILES sequences was done using decorated electron densities. $ed\_factor = 0.2$

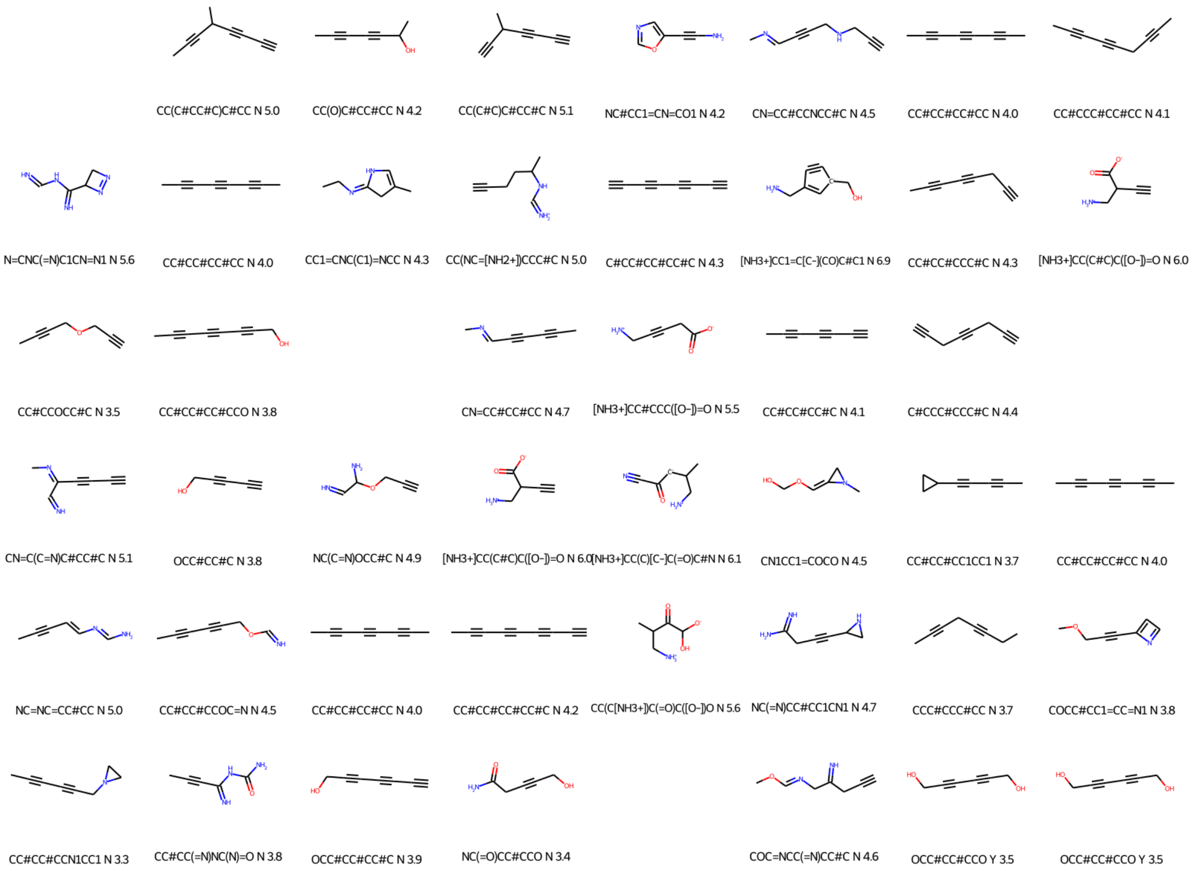

Supplementary Figure 121: Molecules obtained when trying to minimise the overlapping between host (Pd Cage) and guest electron densities, while maximising their electrostatic interactions. The translation from guests to SMILES sequences was done using decorated electron densities. $ed\_factor = 0.3$

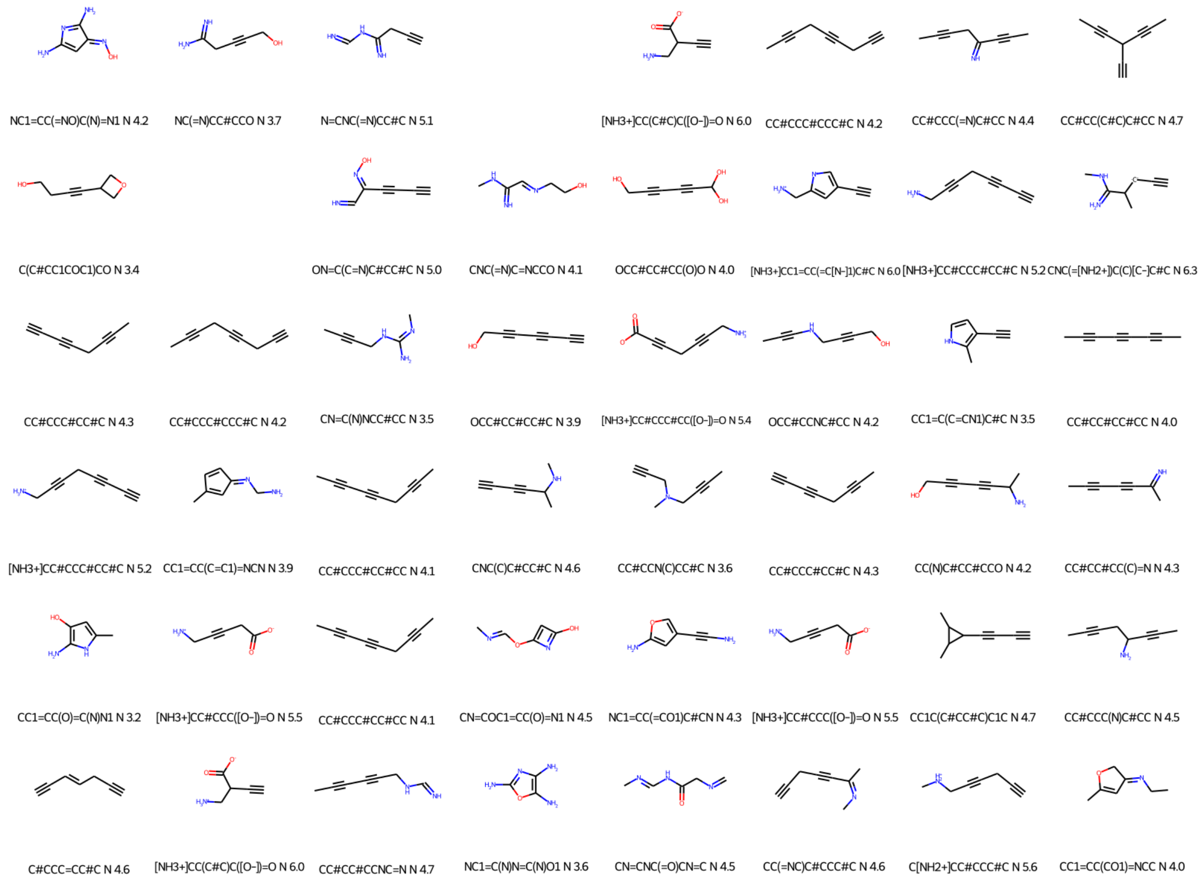

Supplementary Figure 122: Molecules obtained when trying to minimise the overlapping between host (Pd Cage) and guest electron densities, while maximising their electrostatic interactions. The translation from guests to SMILES sequences was done using decorated electron densities. $ed\_factor = 0.4$

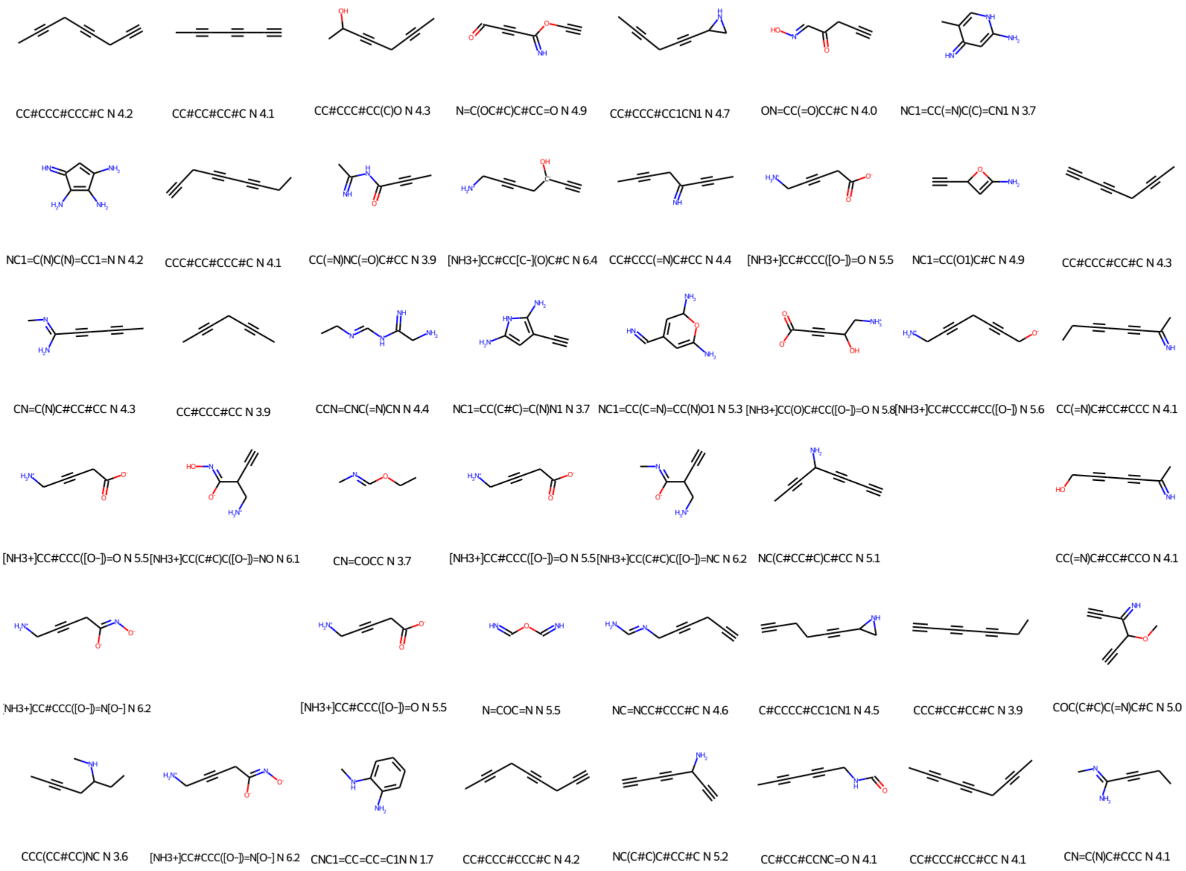

Supplementary Figure 123: Molecules obtained when trying to minimise the overlapping between host (Pd Cage) and guest electron densities, while maximising their electrostatic interactions. The translation from guests to SMILES sequences was done using decorated electron densities. $ed\_factor = 0.5$

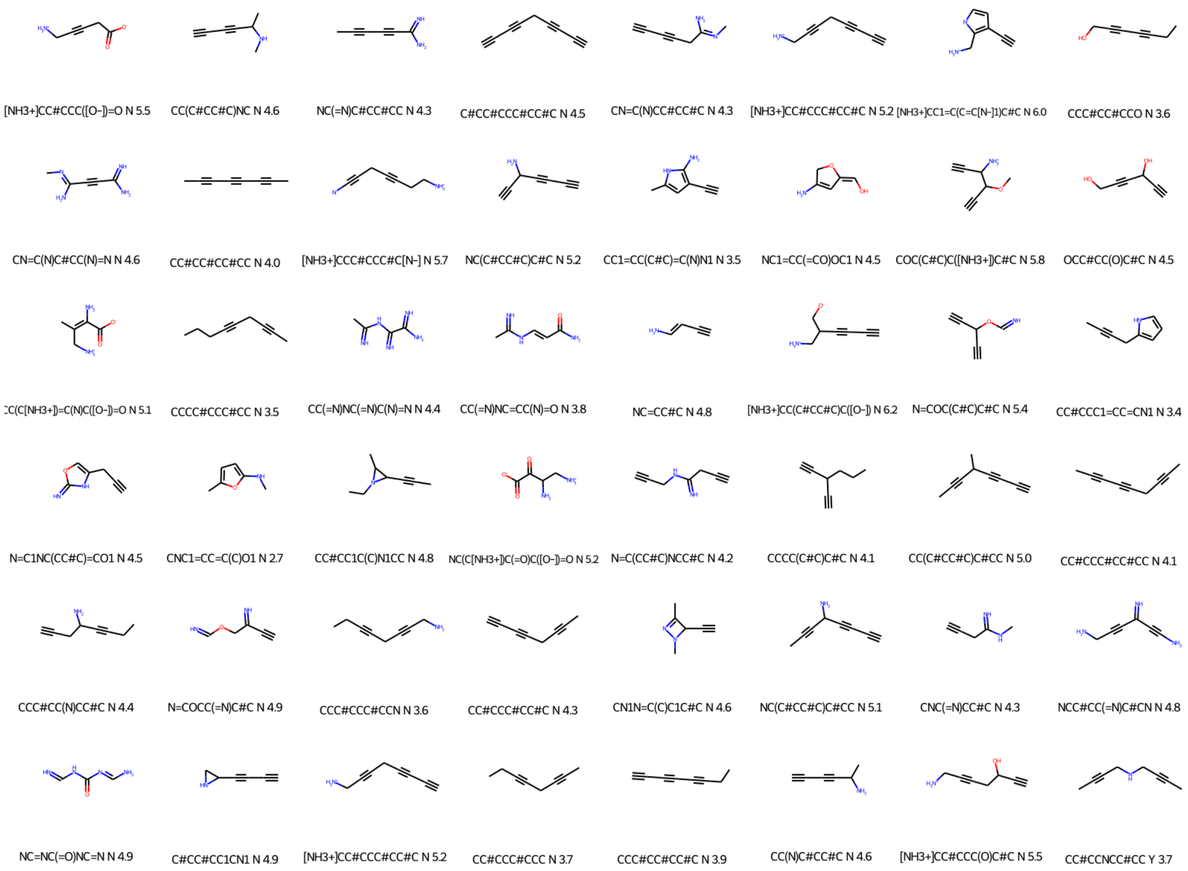

Supplementary Figure 124: Molecules obtained when trying to minimise the overlapping between host (Pd Cage) and guest electron densities, while maximising their electrostatic interactions. The translation from guests to SMILES sequences was done using decorated electron densities. $ed\_factor = 0.6$

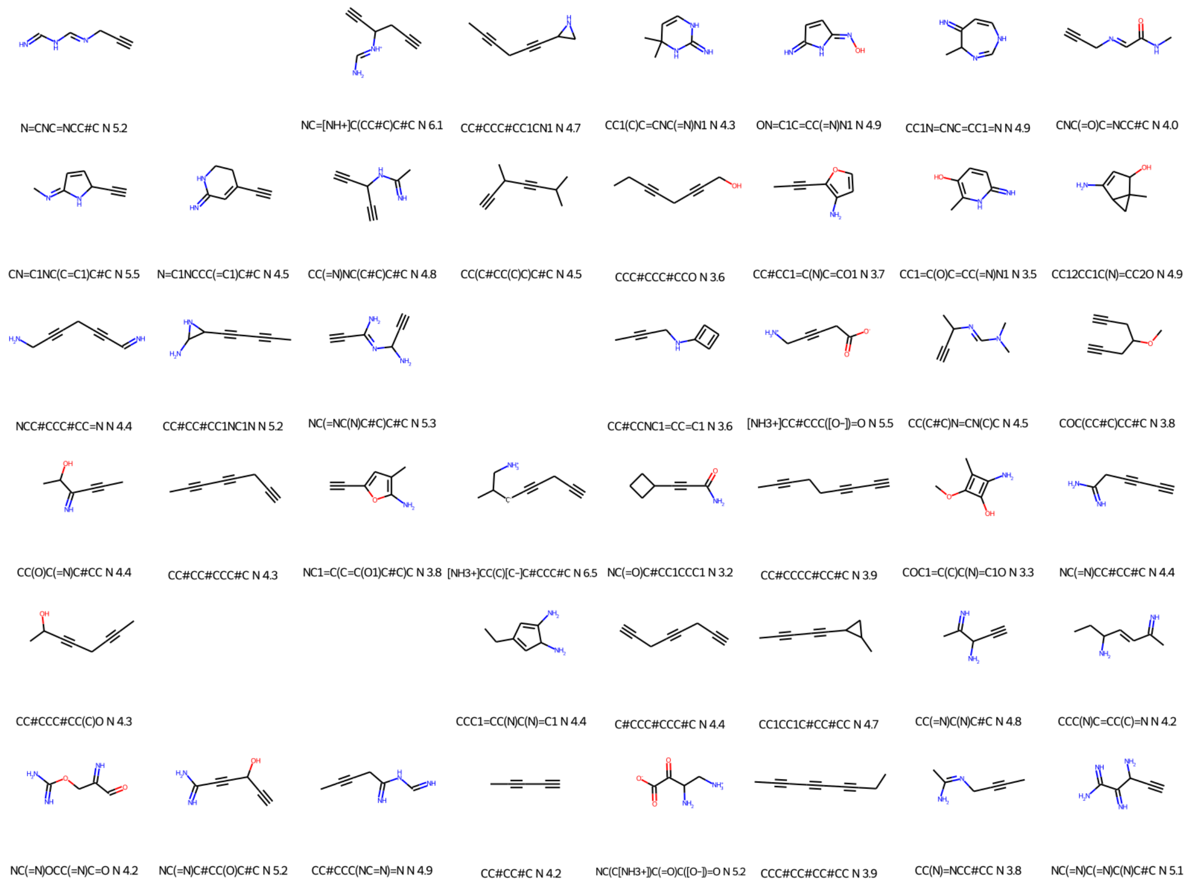

Supplementary Figure 125: Molecules obtained when trying to minimise the overlapping between host (Pd Cage) and guest electron densities, while maximising their electrostatic interactions. The translation from guests to SMILES sequences was done using decorated electron densities. $ed\_factor = 0.7$

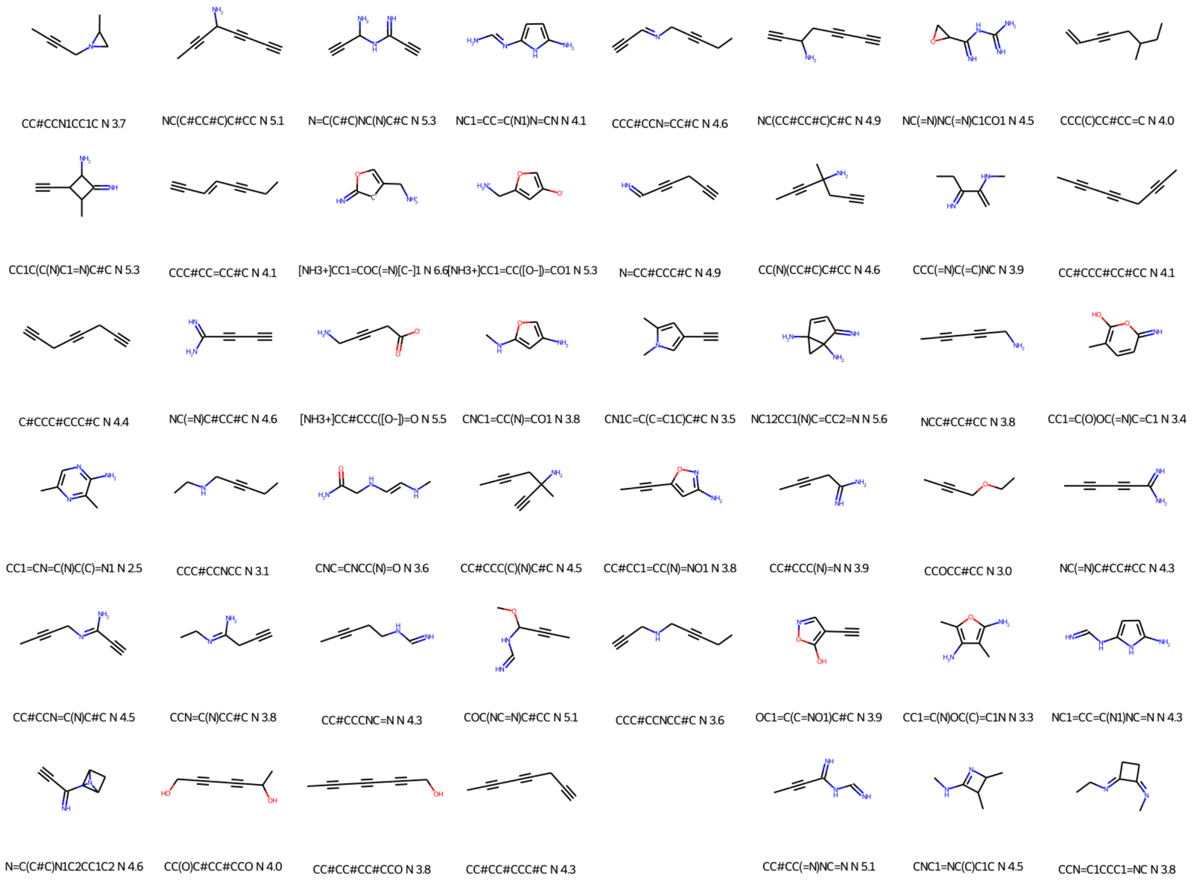

Supplementary Figure 126: Molecules obtained when trying to minimise the overlapping between host (Pd Cage) and guest electron densities, while maximising their electrostatic interactions. The translation from guests to SMILES sequences was done using decorated electron densities. $ed\_factor = 0.8$

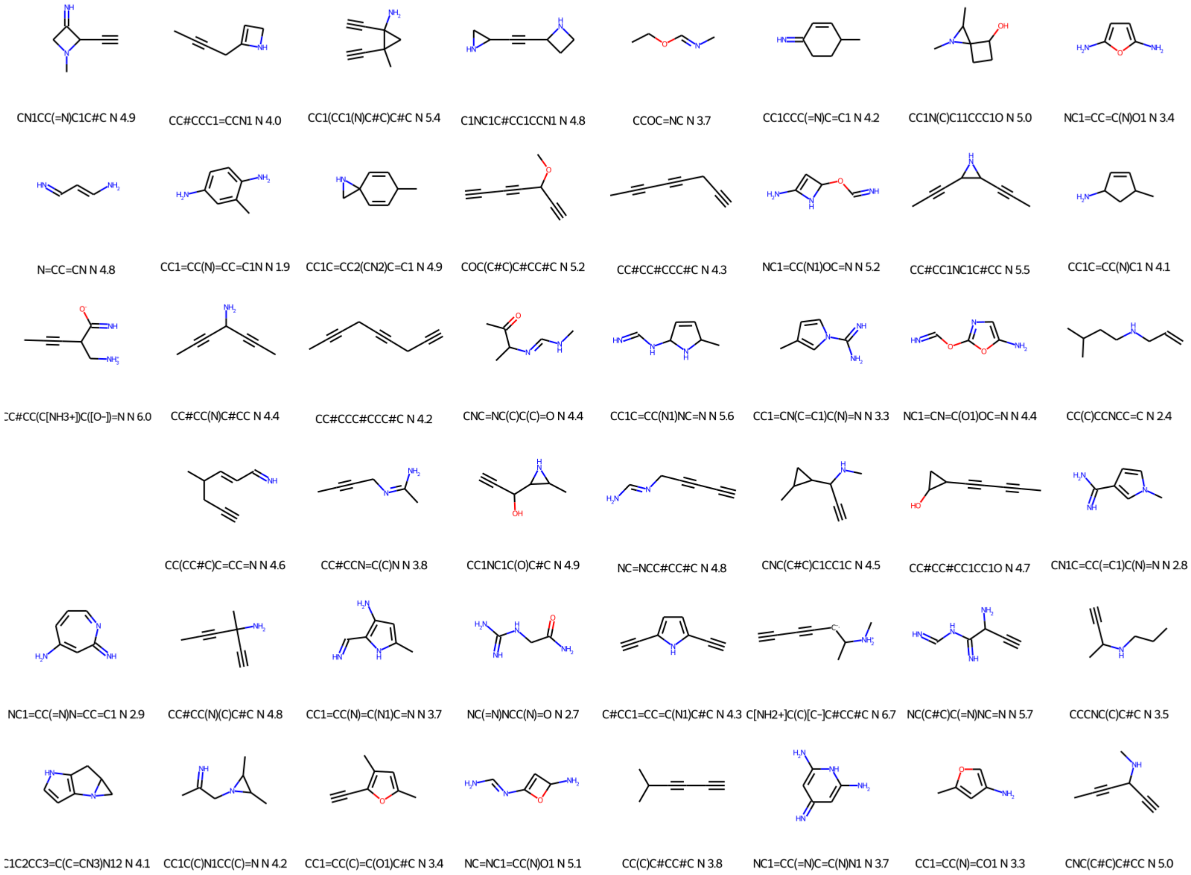

Supplementary Figure 127: Molecules obtained when trying to minimise the overlapping between host (Pd Cage) and guest electron densities, while maximising their electrostatic interactions. The translation from guests to SMILES sequences was done using decorated electron densities. $ed\_factor = 0.9$

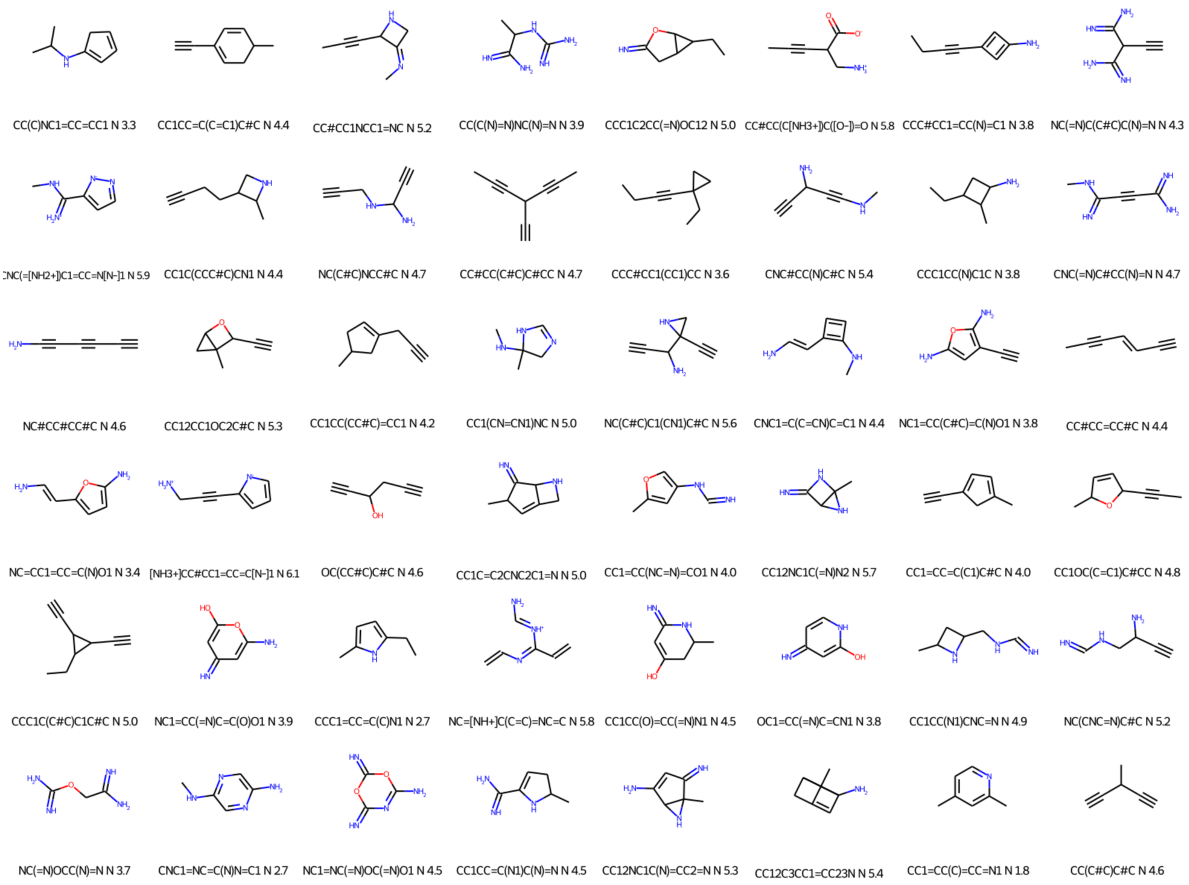

Supplementary Figure 128: Molecules obtained when trying to minimise the overlapping between host (Pd Cage) and guest electron densities, while maximising their electrostatic interactions. The translation from guests to SMILES sequences was done using decorated electron densities. $ed\_factor = 0.95$

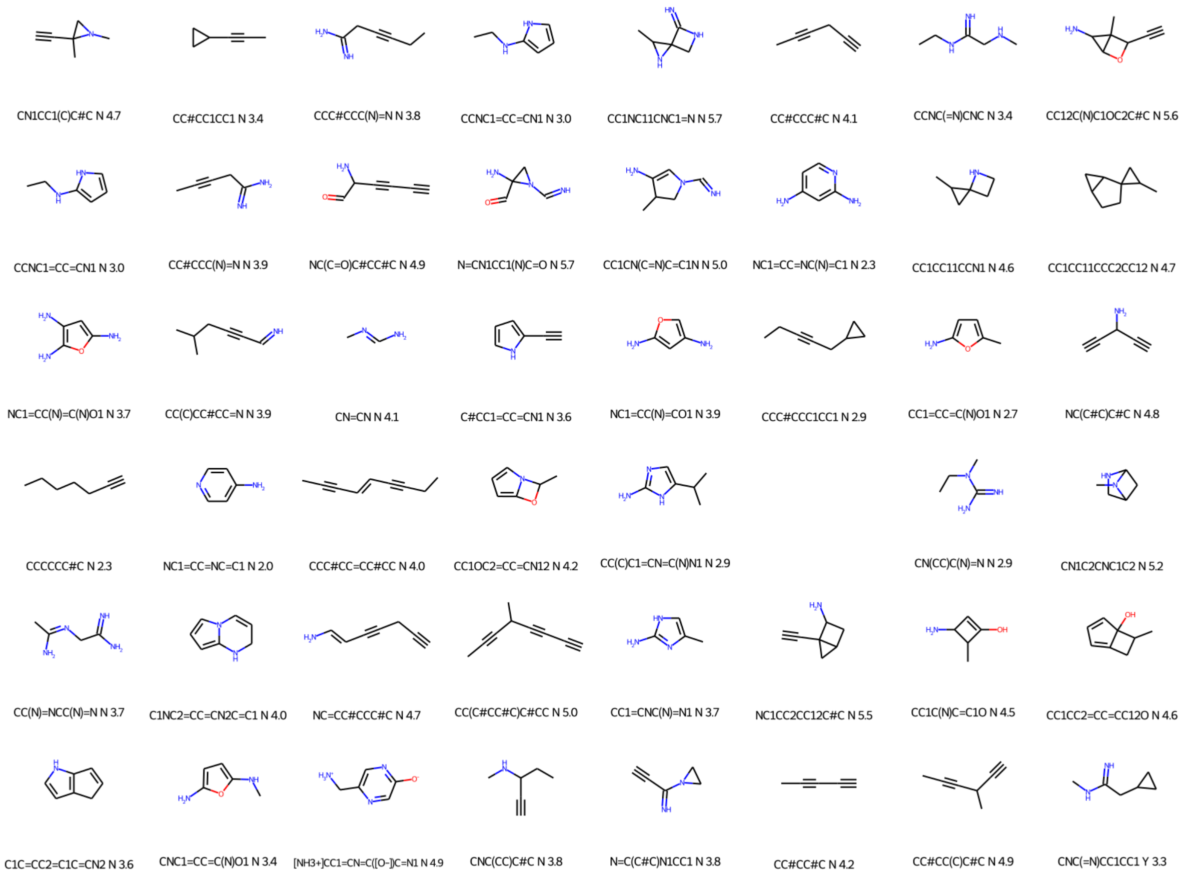

Supplementary Figure 129: Molecules obtained when trying to minimise the overlapping between host (Pd Cage) and guest electron densities, while maximising their electrostatic interactions. The translation from guests to SMILES sequences was done using decorated electron densities. $ed\_factor = 1.0$

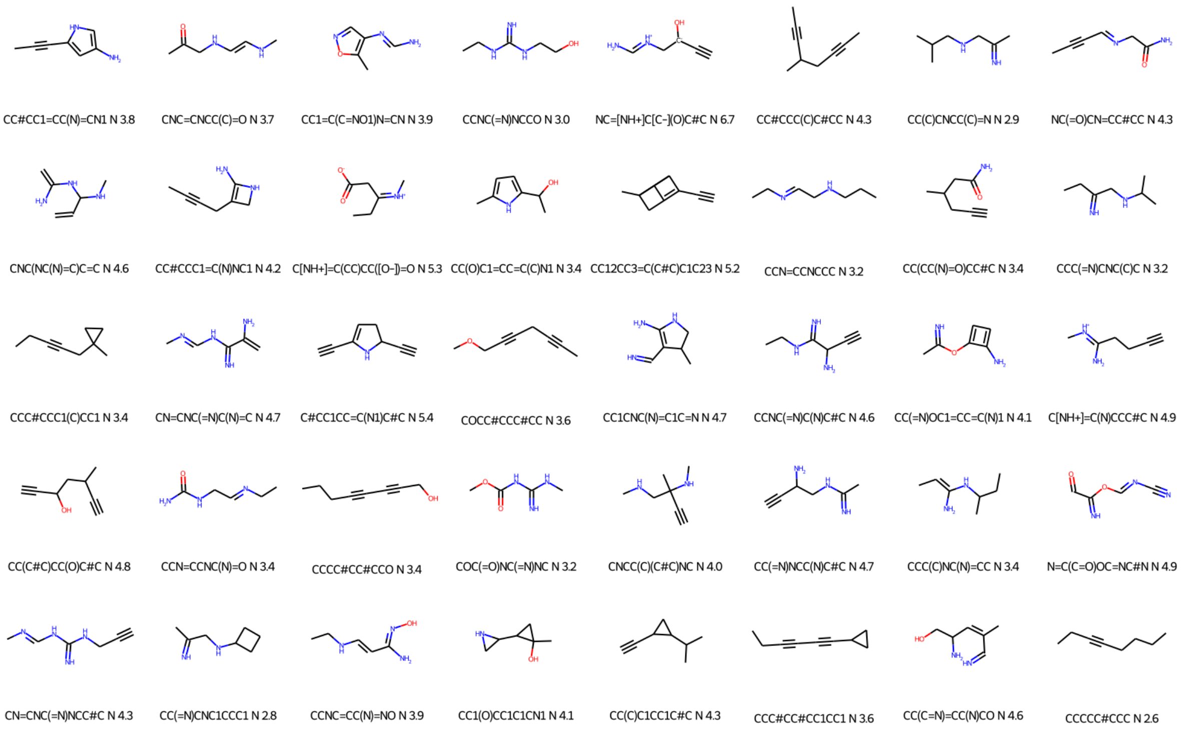

Supplementary Figure 130: Molecules obtained when trying to minimise the overlapping between host (Pd Cage) and guest electron densities, while maximising their electrostatic interactions. The translation from guests to SMILES sequences was done using decorated electron densities. $ed\_factor = 0.0$, $pf\_factor = 0.0$

2.3.9 Results from bin/optimisers/cage_hg_split_esp.py

These results were obtained using the `cage_hg_split_esp.py` script described in Supplementary Section 2.2.9. A total of 125 experiments/runs were done using this script using different values for `ed_factor` and `pf_factor`. This generated 5,000 different guests. These were converted into SMILES sequences using the two methods based on electron density data described in Supplementary Section 1.11, plus the method using decorated electron densities described in Supplementary Section 1.8. Therefore, each 3D tensor representing a guest could output three slightly different molecules. Thus, the total number of molecules generated was 12,768. Since this is a very big number, we will only show some of there here. The results can be seen in Supplementary Figures 130 to 169.

2.3.10 SELFIES results from bin/optimisers/maximise_hg_esp.py

These results were obtained using the `maximise_hg_esp.py` script described in Supplementary Section 2.2.5. To obtain these results, the cavity of CB6 was reduced using the method described in Supplementary Section 1.9. In particular, two rounds were performed, in the first one a kernel of size 3 was used, while in the second one a kernel of size 4 was used. A total of 12 experiments for each cavity size (therefore, 12 twice 2 equals 24 experiments) were done using this script using different values for `ed_factor`. This generated 1152 different guests. These were converted into SELFIES sequences with the method using decorated electron densities described in Supplementary Section 1.8.

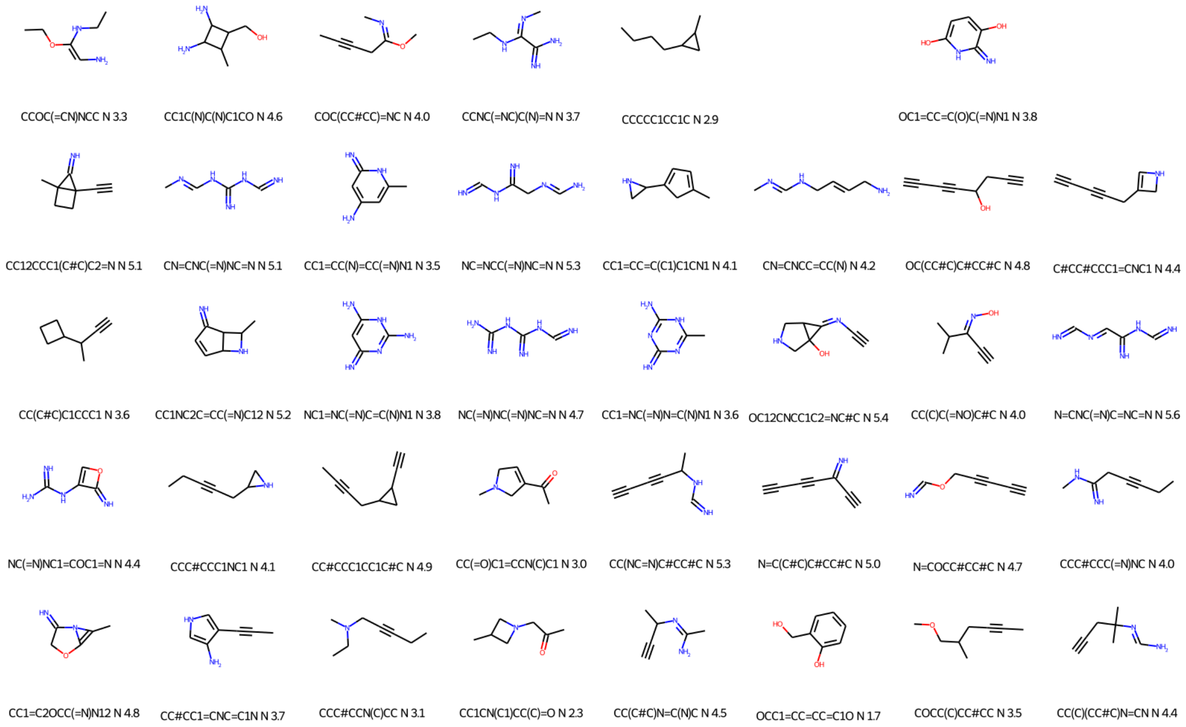

Supplementary Figure 131: Molecules obtained when trying to minimise the overlapping between host (Pd Cage) and guest electron densities, while maximising their electrostatic interactions. The translation from guests to SMILES sequences was done using decorated electron densities. $ed\_factor = 0.0$, $pf\_factor = 0.2$

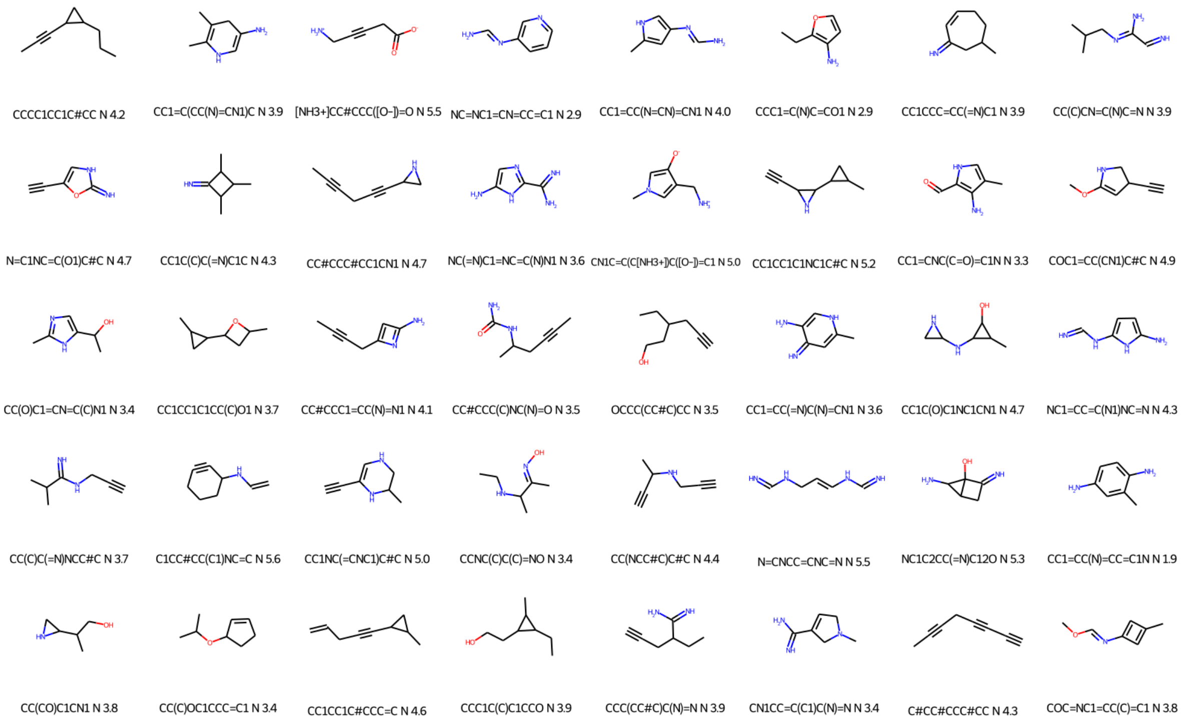

Supplementary Figure 132: Molecules obtained when trying to minimise the overlapping between host (Pd Cage) and guest electron densities, while maximising their electrostatic interactions. The translation from guests to SMILES sequences was done using decorated electron densities. $ed\_factor = 0.0$, $pf\_factor = 0.4$

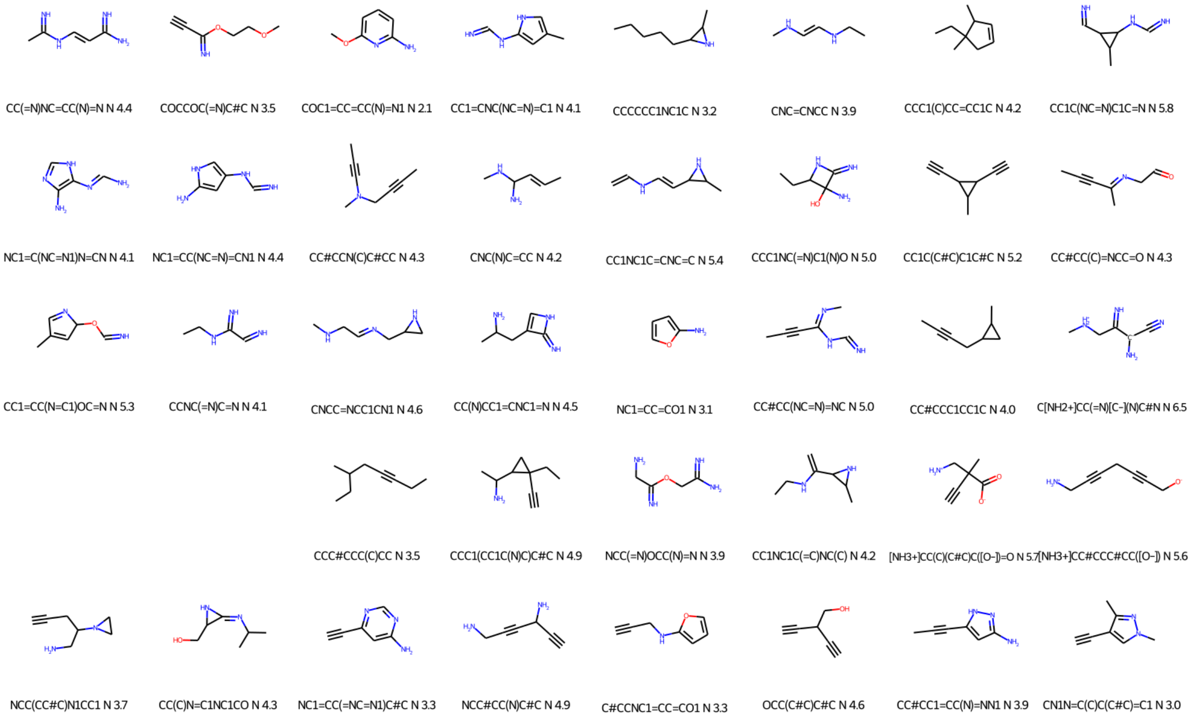

Supplementary Figure 133: Molecules obtained when trying to minimise the overlapping between host (Pd Cage) and guest electron densities, while maximising their electrostatic interactions. The translation from guests to SMILES sequences was done using decorated electron densities. $ed\_factor = 0.0$, $pf\_factor = 0.6$

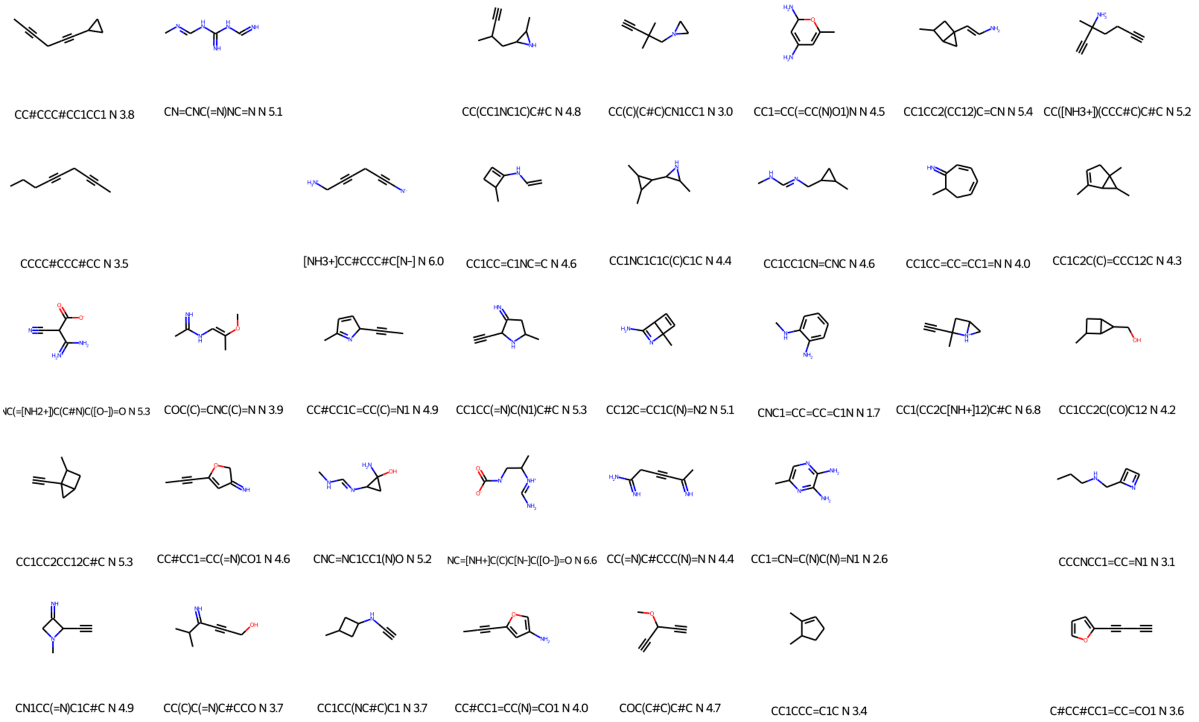

Supplementary Figure 134: Molecules obtained when trying to minimise the overlapping between host (Pd Cage) and guest electron densities, while maximising their electrostatic interactions. The translation from guests to SMILES sequences was done using decorated electron densities. $ed\_factor = 0.0$, $pf\_factor = 0.8$

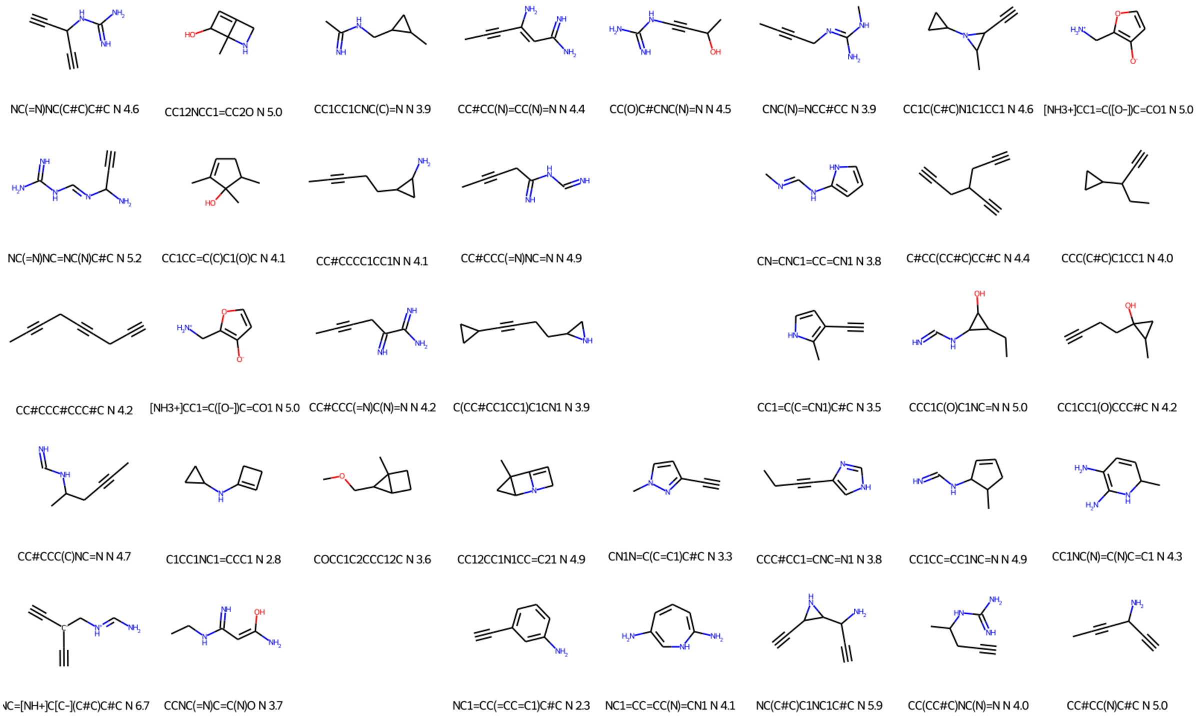

Supplementary Figure 135: Molecules obtained when trying to minimise the overlapping between host (Pd Cage) and guest electron densities, while maximising their electrostatic interactions. The translation from guests to SMILES sequences was done using decorated electron densities. $ed\_factor = 0.0$, $pf\_factor = 1.0$

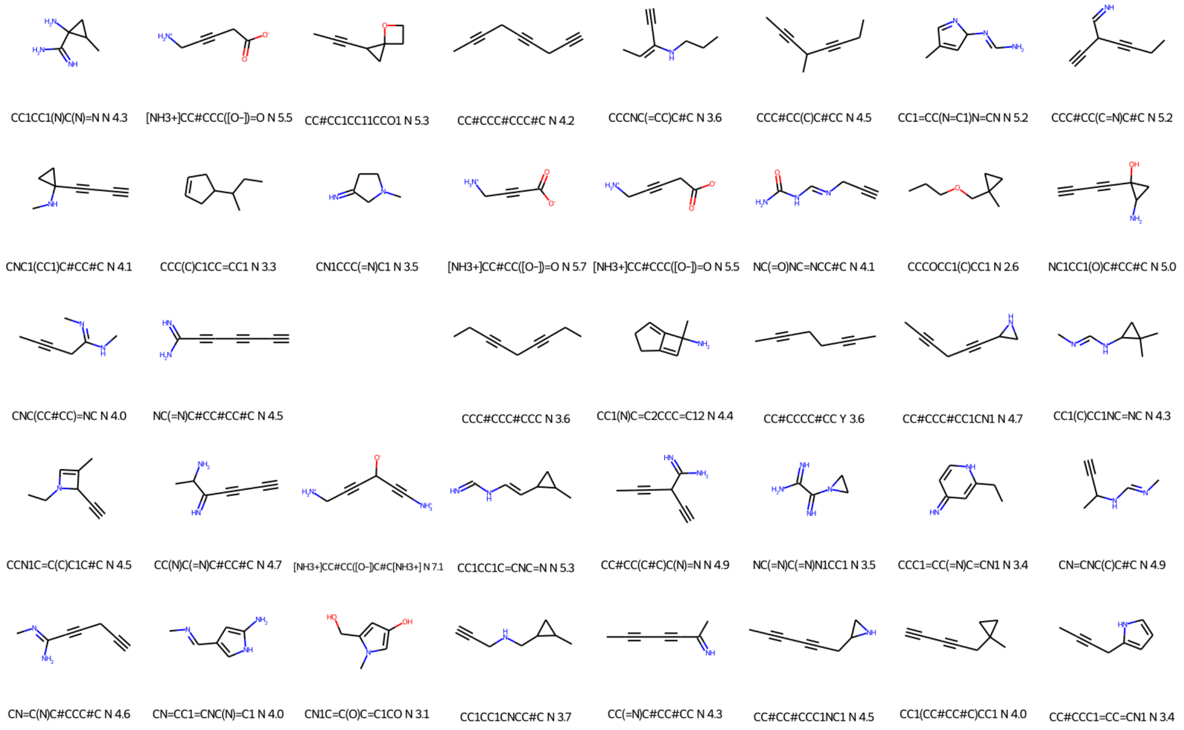

Supplementary Figure 136: Molecules obtained when trying to minimise the overlapping between host (Pd Cage) and guest electron densities, while maximising their electrostatic interactions. The translation from guests to SMILES sequences was done using decorated electron densities. $ed\_factor = 0.0$, $pf\_factor = 2.0$

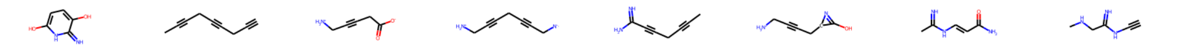

OC1=CC=C(O)C(=N)N1 N 3.8 CC#CCC#CCC#C N 4.2 [NH3+]CC#CCC([O-])=O N 5.5 [NH3+]CC#CCC#CC[N-] N 5.7 CC#CCC#CC(N)=N N 4.5 [NH3+]CC#CC[C-]1C(O)=N1 N 6.2 CC(=N)NC=CC(N)=O N 3.8 CNCC(=N)NC#C N 4.3

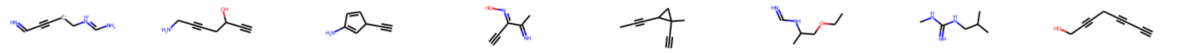

NC=[NH+]C[C-]C#CC=N N 6.9 [NH3+]CC#CCC(O)C#C N 5.5 NC1=CC(C=C1)C#C N 4.9 CC(=N)C(=NO)C#C N 4.3 CC1(CC1C#CC)C#C N 5.3 CCOCC(C)NC=N N 4.0 CNC(=N)NCC(C)C N 3.1 OCC#CCC#CC#C N 4.0

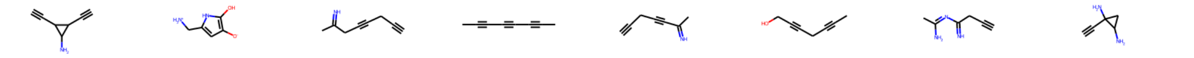

NC1C(C#C)C1C#C N 5.4 [NH3+]CC1=CC([O-])=C(O)N1 N 5.3 CC(=N)CC#CCC#C N 4.3 CC#CC#CC#CC N 4.0 CC(=N)C#CCC#C N 4.6 CC#CCC#CCO N 3.7 CC(N)=NC(=N)CC#C N 4.2 NC1CC1(N)C#C N 5.0

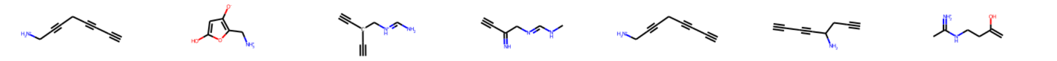

[NH3+]CC#CCC#CC#C N 5.2 [NH3+]CC1=C([O-])C=C(O)O1 N 5.3 NC=[NH+]C[C-](C#C)C#C N 6.7 CNC=NCC(=N)C#C N 5.0 [NH3+]CC#CCC#CC#C N 5.2 NC(CC#C)C#CC#C N 4.9 CC(=[NH2+])NCCC(O)=C N 4.1

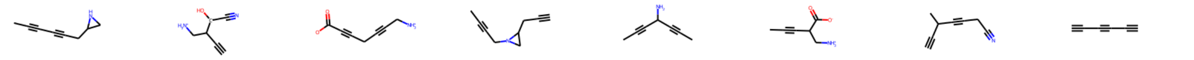

CC#CC#CCC1CN1 N 4.5 [NH3+]CC(C#C)[C-](O)C#N N 6.7 [NH3+]CC#CCC#CC([O-])=O N 5.4 CC#CCN1CC1CC#C N 4.7 CC#CC(N)C#CC N 4.4 CC#CC(C[NH3+])C([O-])=O N 5.8 CC(C#C)C#CCC#N N 5.0 C#CC#CC#C N 4.2

Supplementary Figure 137: Molecules obtained when trying to minimise the overlapping between host (Pd Cage) and guest electron densities, while maximising their electrostatic interactions. The translation from guests to SMILES sequences was done using decorated electron densities. $ed\_factor = 0.0$, $pf\_factor = 10.0$

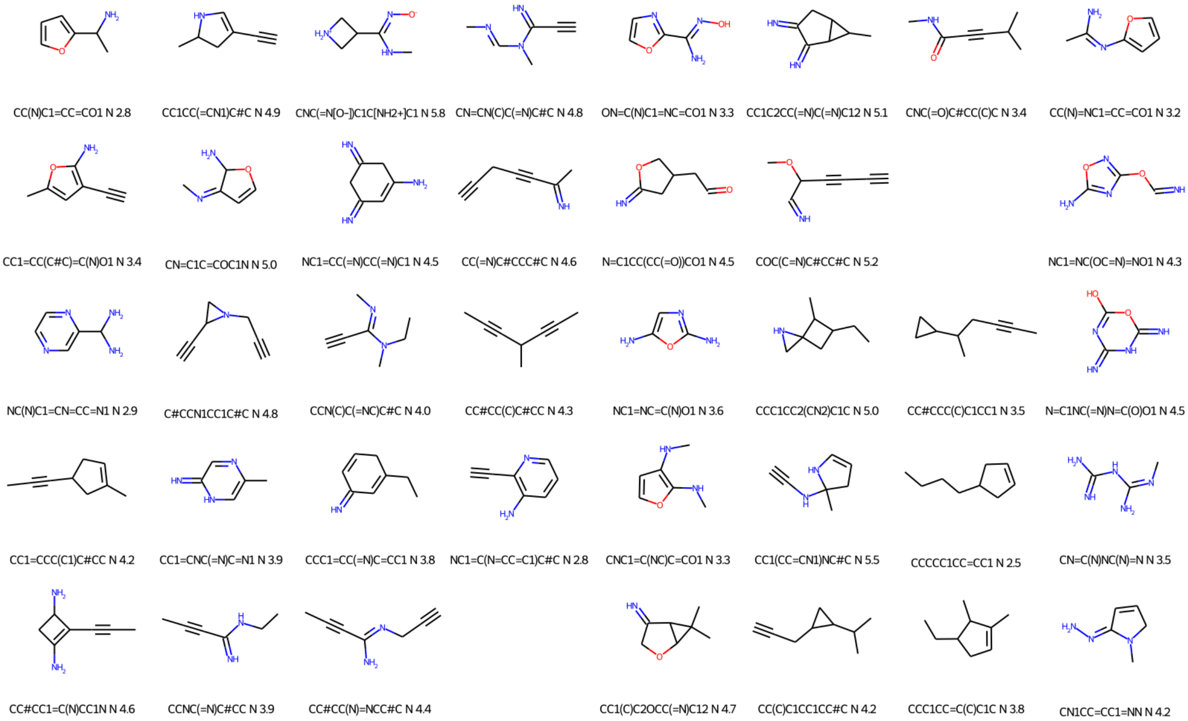

Supplementary Figure 138: Molecules obtained when trying to minimise the overlapping between host (Pd Cage) and guest electron densities, while maximising their electrostatic interactions. The translation from guests to SMILES sequences was done using decorated electron densities. $ed\_factor = 0.1$, $pf\_factor = 0.0$

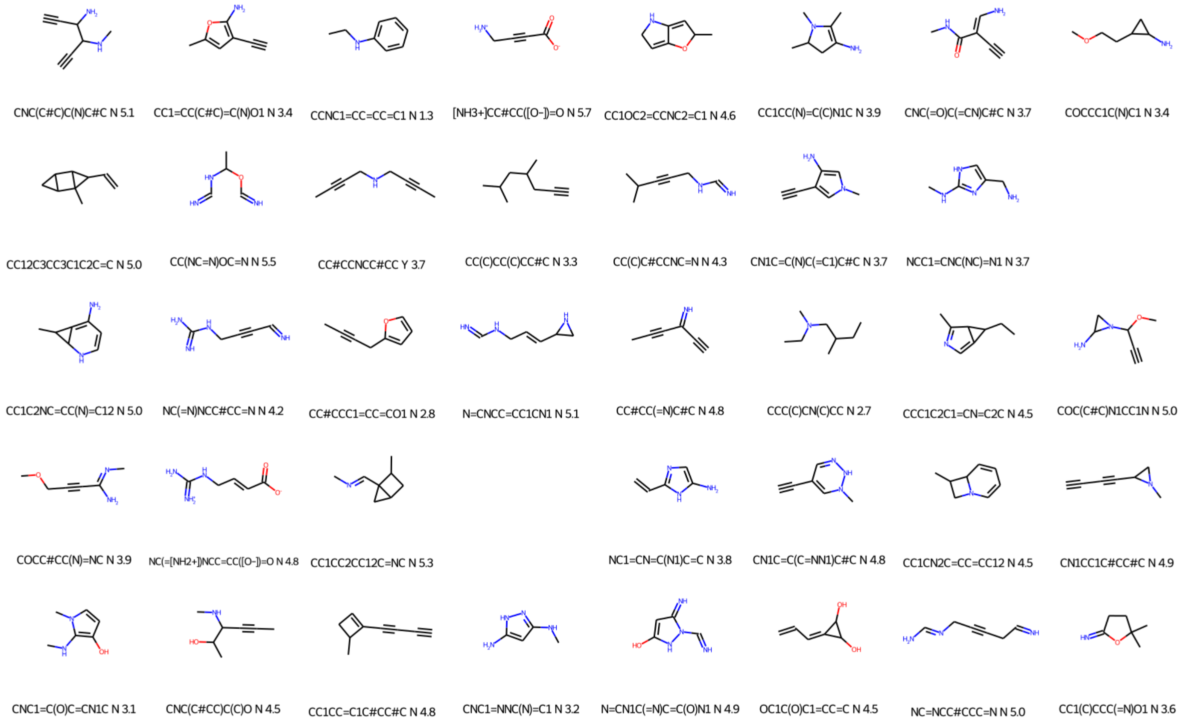

Supplementary Figure 139: Molecules obtained when trying to minimise the overlapping between host (Pd Cage) and guest electron densities, while maximising their electrostatic interactions. The translation from guests to SMILES sequences was done using decorated electron densities. $ed\_factor = 0.1$, $pf\_factor = 0.2$

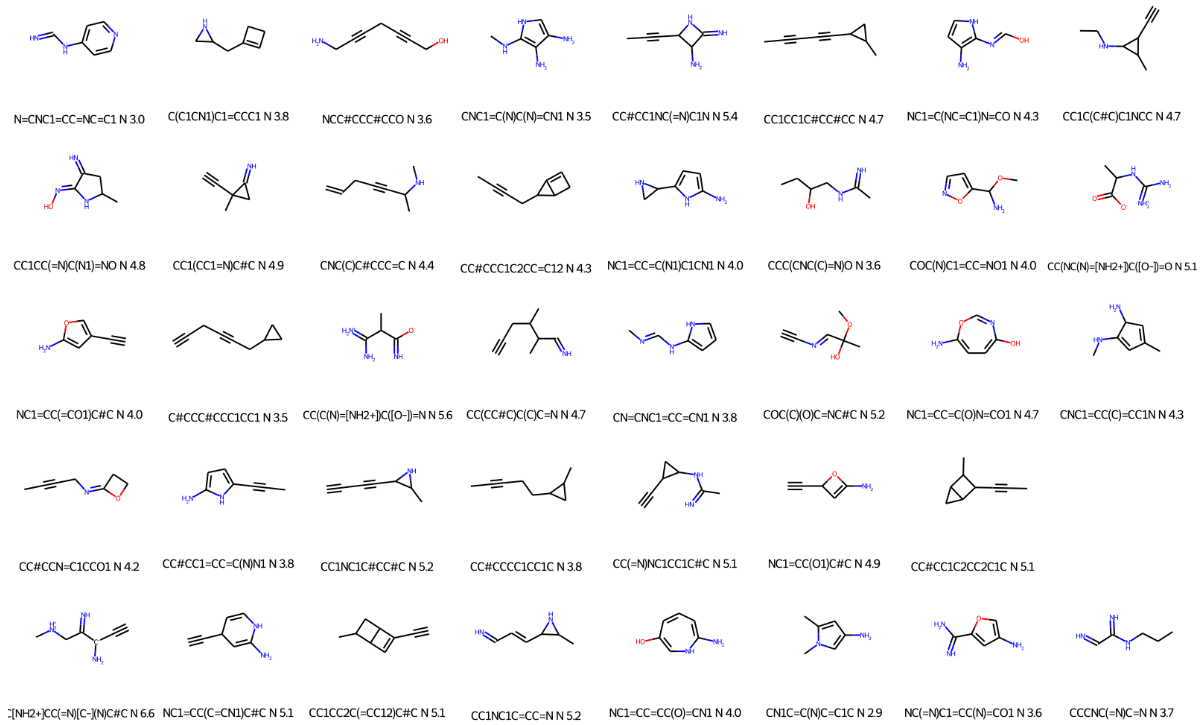

Supplementary Figure 140: Molecules obtained when trying to minimise the overlapping between host (Pd Cage) and guest electron densities, while maximising their electrostatic interactions. The translation from guests to SMILES sequences was done using decorated electron densities. $ed\_factor = 0.1$, $pf\_factor = 0.4$

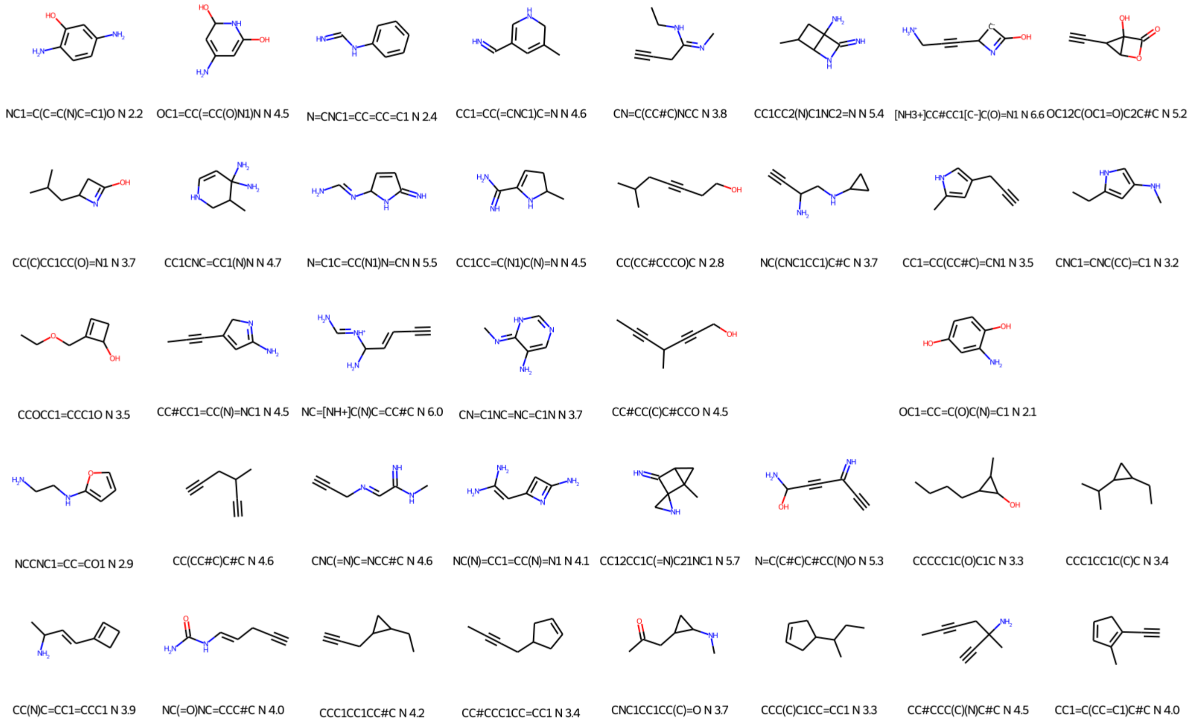

Supplementary Figure 141: Molecules obtained when trying to minimise the overlapping between host (Pd Cage) and guest electron densities, while maximising their electrostatic interactions. The translation from guests to SMILES sequences was done using decorated electron densities. $ed\_factor = 0.1$, $pf\_factor = 0.6$

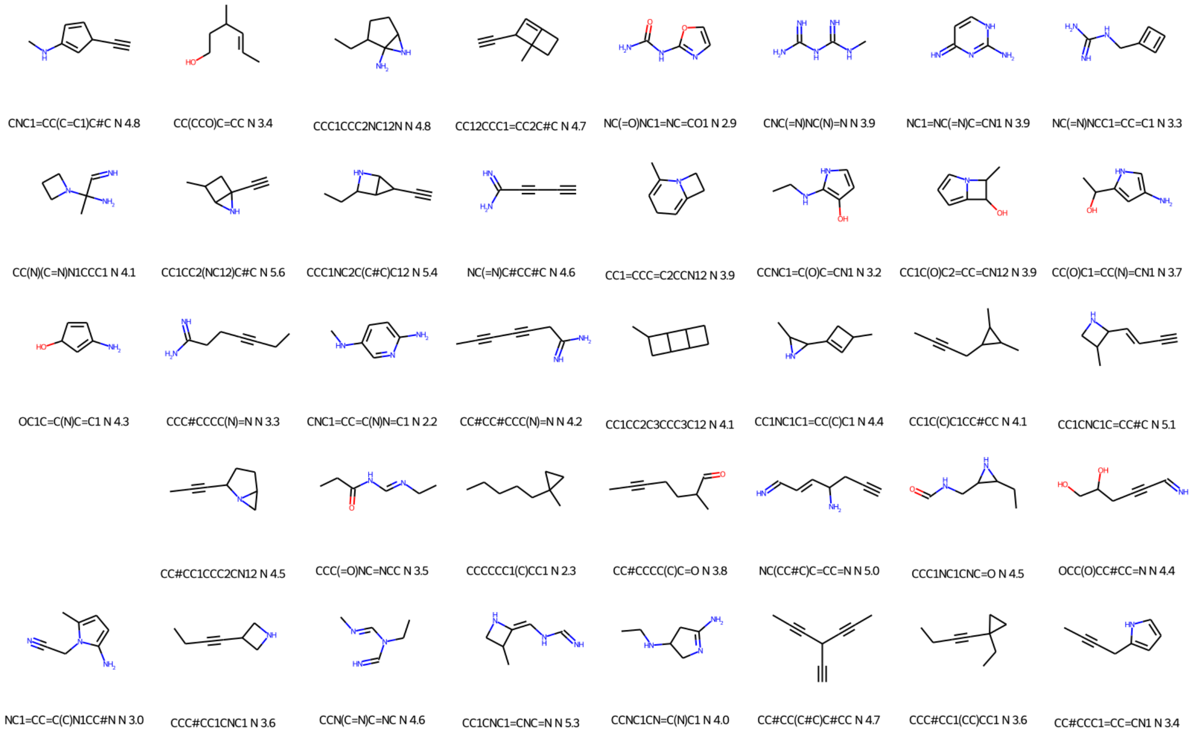

Supplementary Figure 142: Molecules obtained when trying to minimise the overlapping between host (Pd Cage) and guest electron densities, while maximising their electrostatic interactions. The translation from guests to SMILES sequences was done using decorated electron densities. $ed\_factor = 0.1$, $pf\_factor = 0.8$

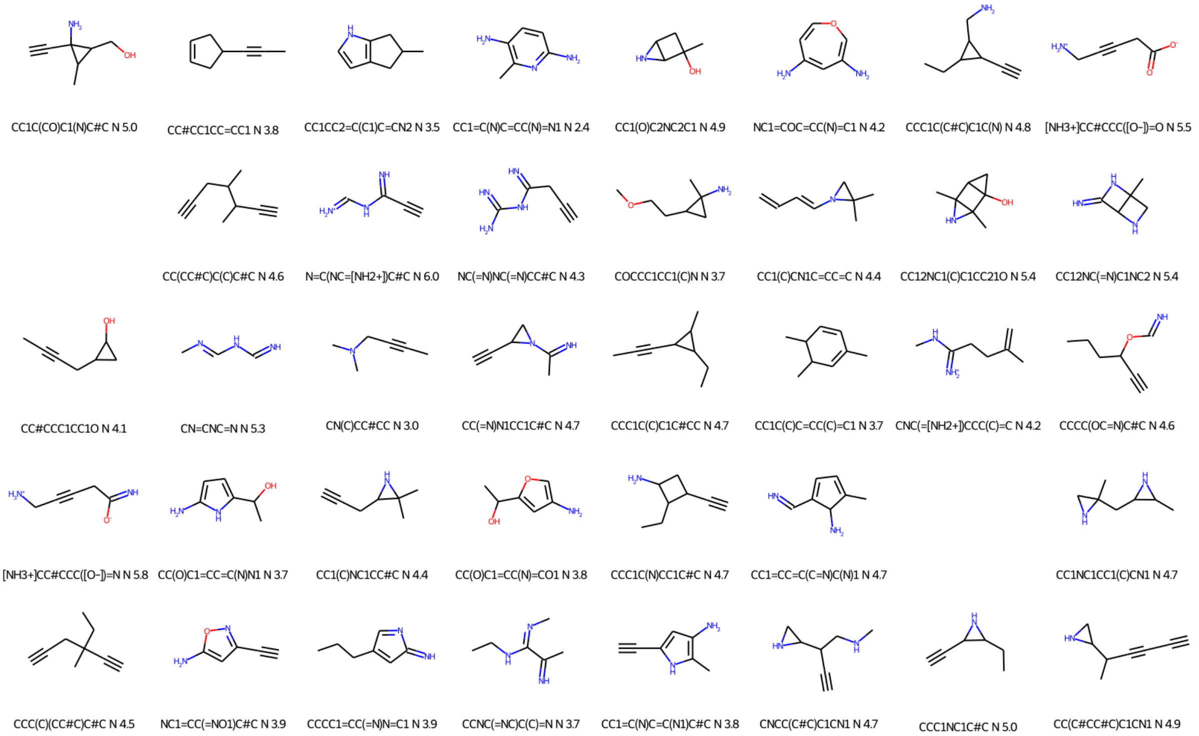

Supplementary Figure 143: Molecules obtained when trying to minimise the overlapping between host (Pd Cage) and guest electron densities, while maximising their electrostatic interactions. The translation from guests to SMILES sequences was done using decorated electron densities. $ed\_factor = 0.1$, $pf\_factor = 1.0$

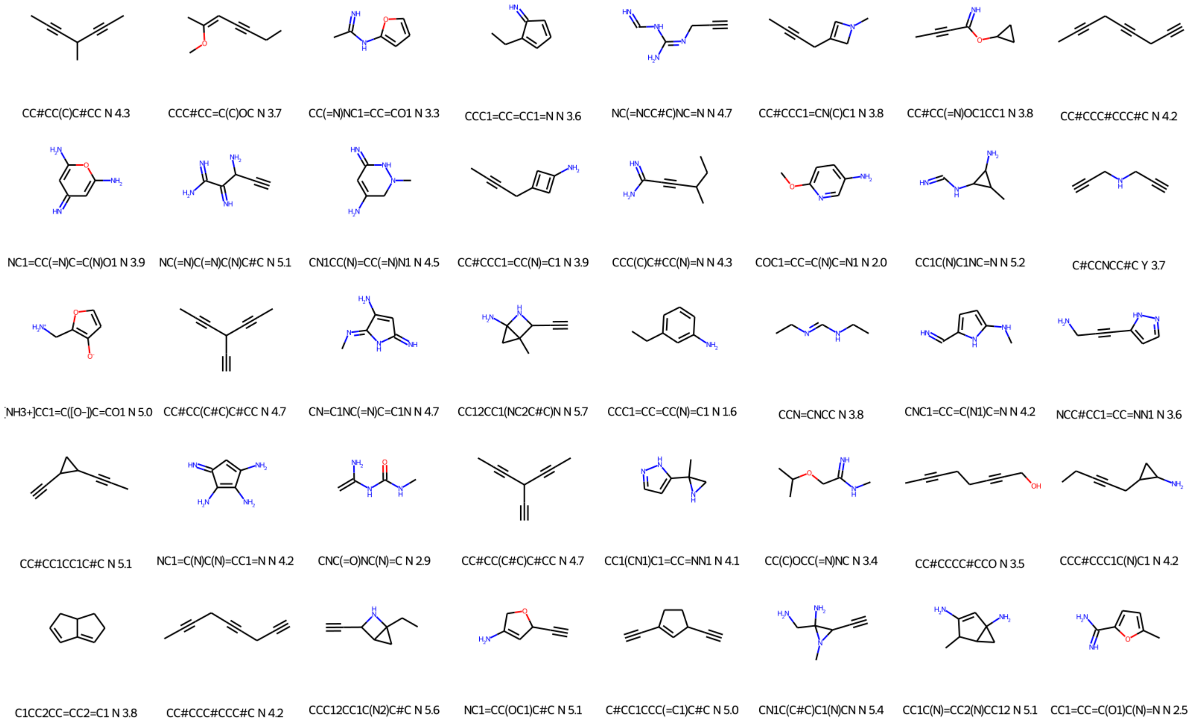

Supplementary Figure 144: Molecules obtained when trying to minimise the overlapping between host (Pd Cage) and guest electron densities, while maximising their electrostatic interactions. The translation from guests to SMILES sequences was done using decorated electron densities. $ed\_factor = 0.1$, $pf\_factor = 2.0$

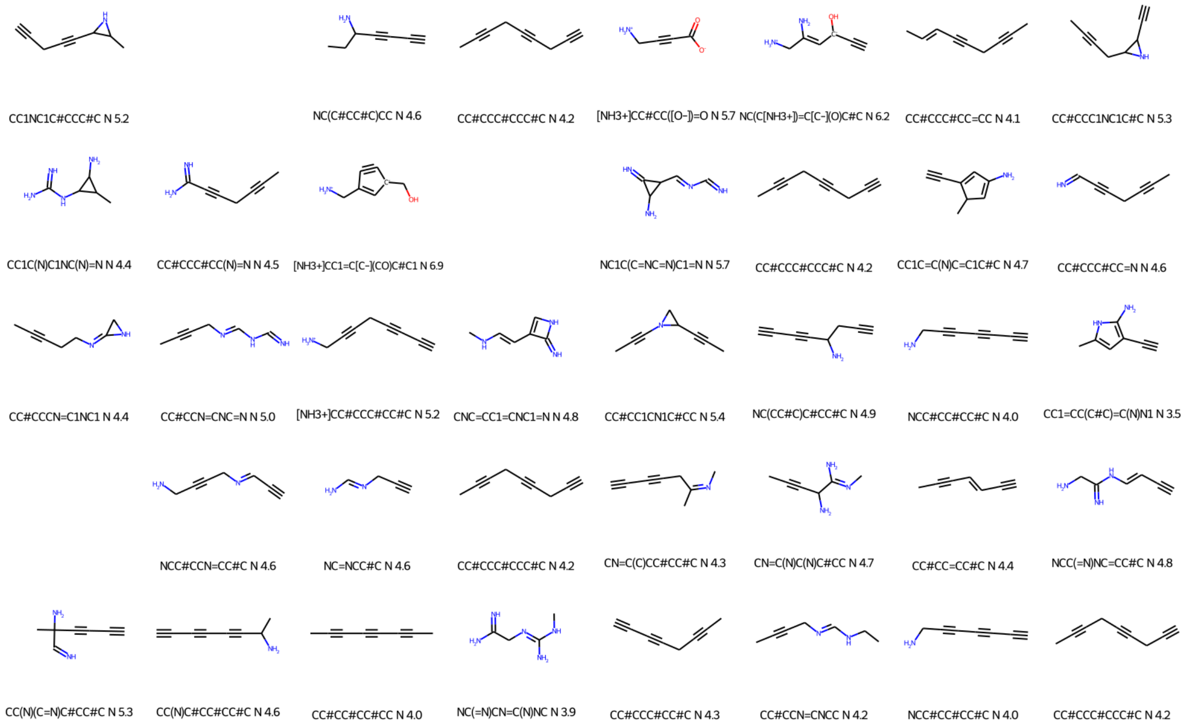

Supplementary Figure 145: Molecules obtained when trying to minimise the overlapping between host (Pd Cage) and guest electron densities, while maximising their electrostatic interactions. The translation from guests to SMILES sequences was done using decorated electron densities. $ed\_factor = 0.1$, $pf\_factor = 10.0$

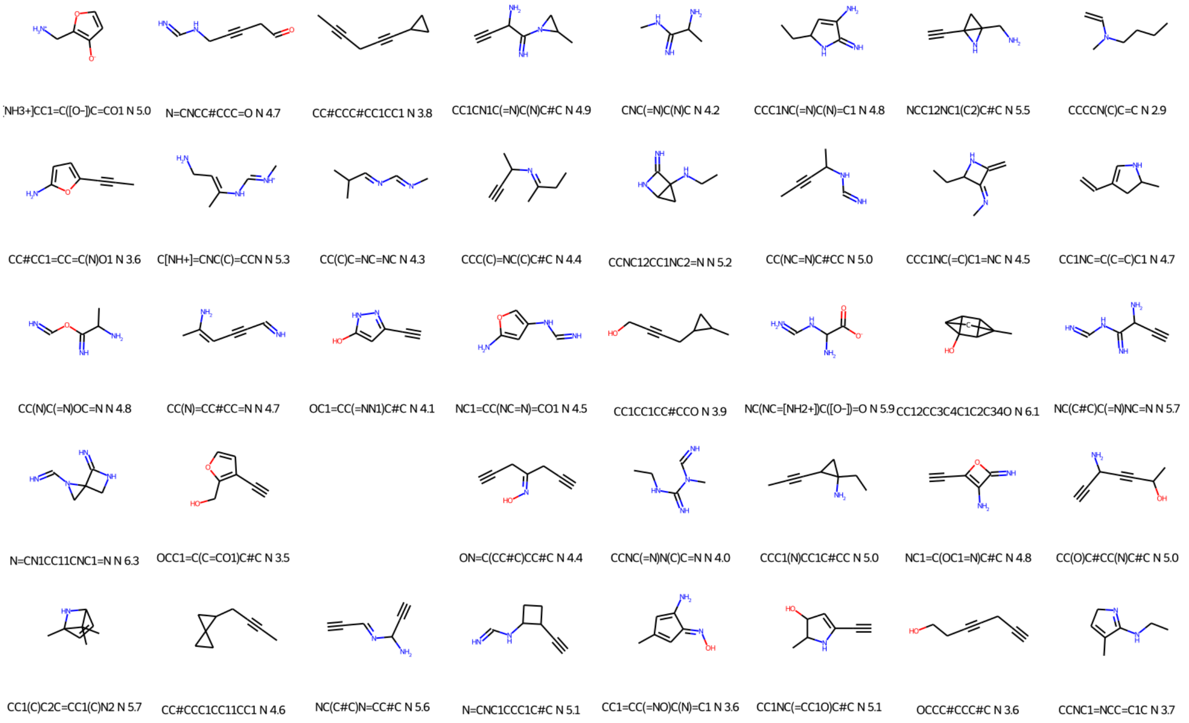

Supplementary Figure 146: Molecules obtained when trying to minimise the overlapping between host (Pd Cage) and guest electron densities, while maximising their electrostatic interactions. The translation from guests to SMILES sequences was done using decorated electron densities. $ed\_factor = 0.3$, $pf\_factor = 0.0$

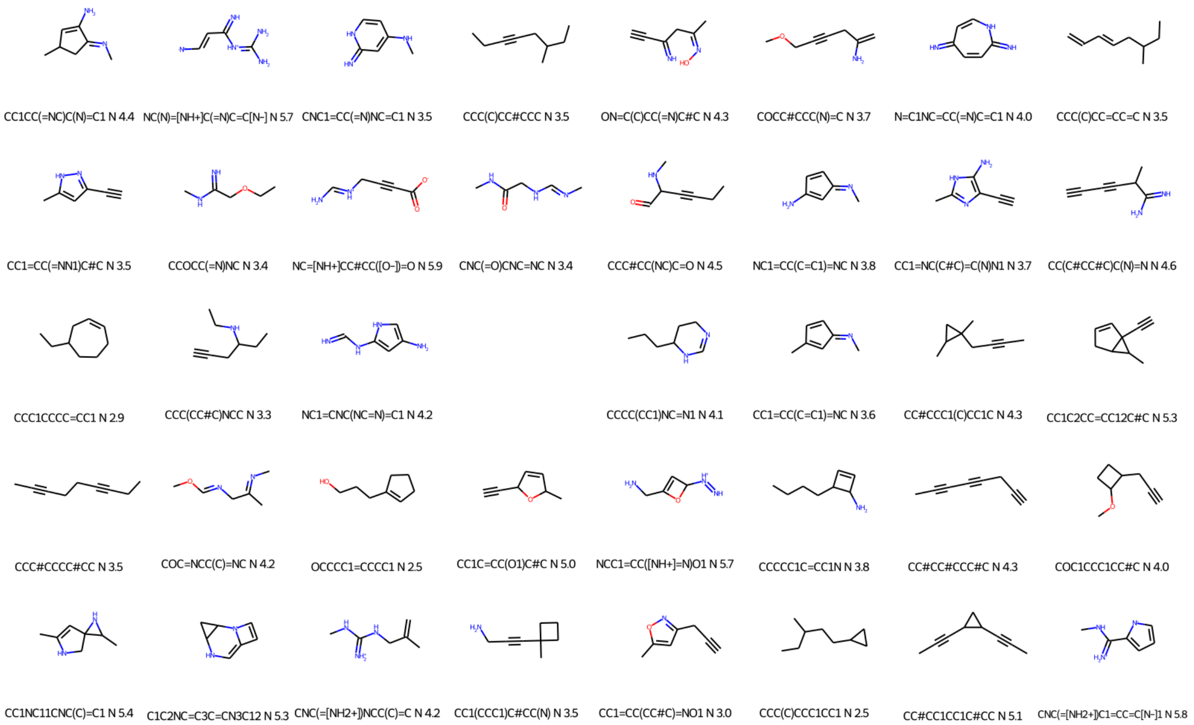

Supplementary Figure 147: Molecules obtained when trying to minimise the overlapping between host (Pd Cage) and guest electron densities, while maximising their electrostatic interactions. The translation from guests to SMILES sequences was done using decorated electron densities. $ed\_factor = 0.3$, $pf\_factor = 0.2$

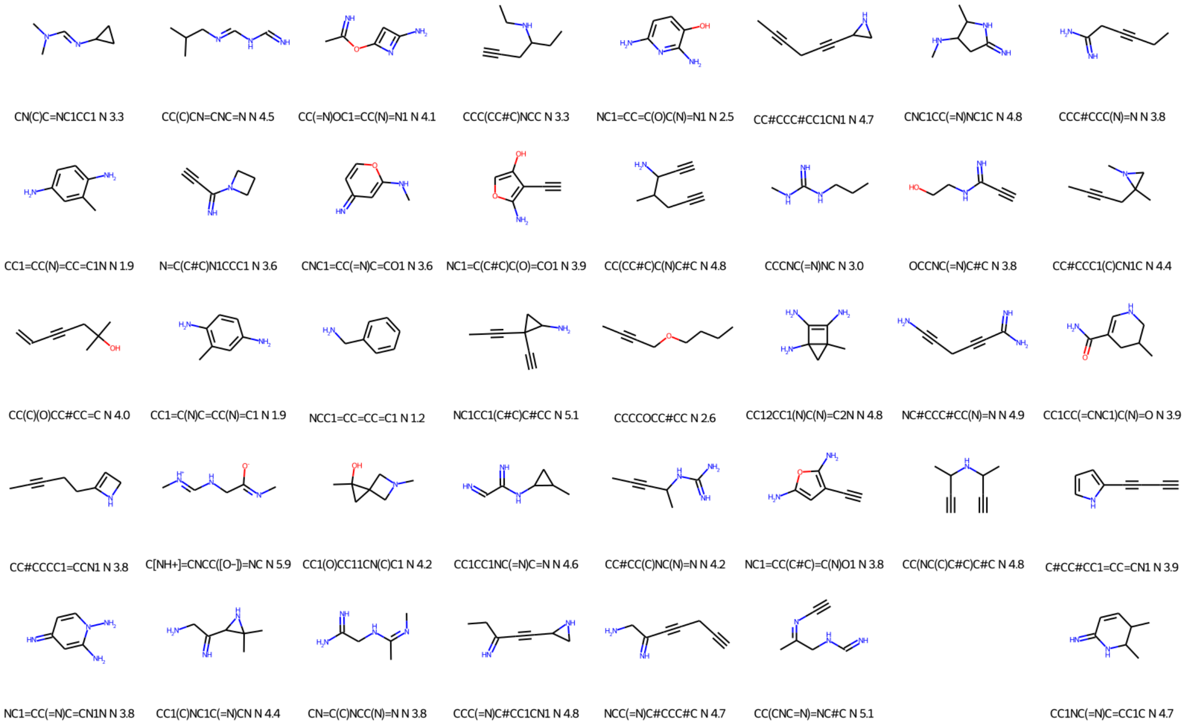

Supplementary Figure 148: Molecules obtained when trying to minimise the overlapping between host (Pd Cage) and guest electron densities, while maximising their electrostatic interactions. The translation from guests to SMILES sequences was done using decorated electron densities. $ed\_factor = 0.3$, $pf\_factor = 0.4$

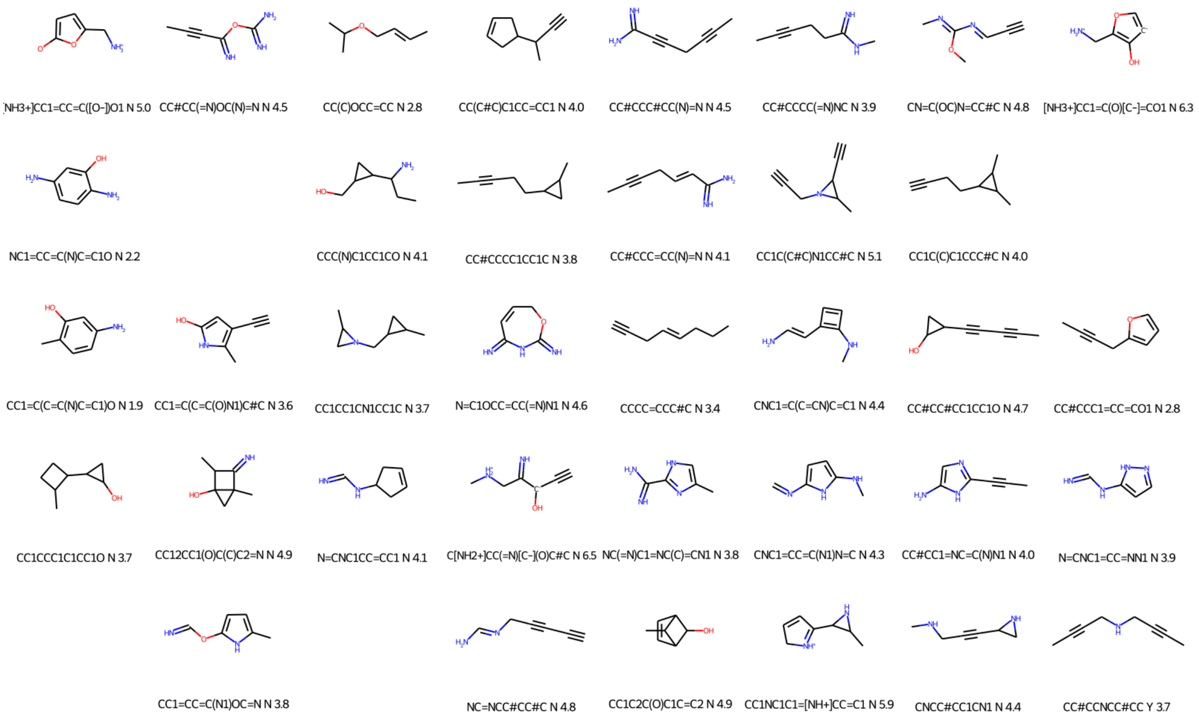

Supplementary Figure 149: Molecules obtained when trying to minimise the overlapping between host (Pd Cage) and guest electron densities, while maximising their electrostatic interactions. The translation from guests to SMILES sequences was done using decorated electron densities. $ed\_factor = 0.3$, $pf\_factor = 0.6$

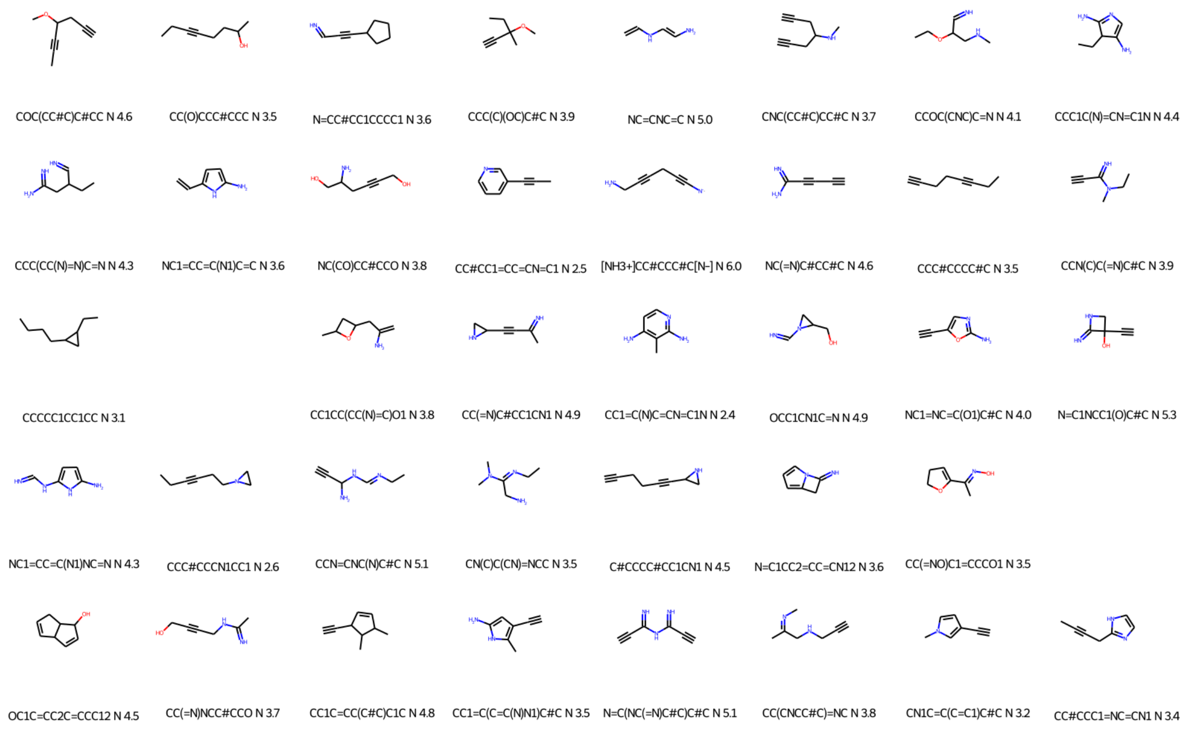

Supplementary Figure 150: Molecules obtained when trying to minimise the overlapping between host (Pd Cage) and guest electron densities, while maximising their electrostatic interactions. The translation from guests to SMILES sequences was done using decorated electron densities. $ed\_factor = 0.3$, $pf\_factor = 0.8$

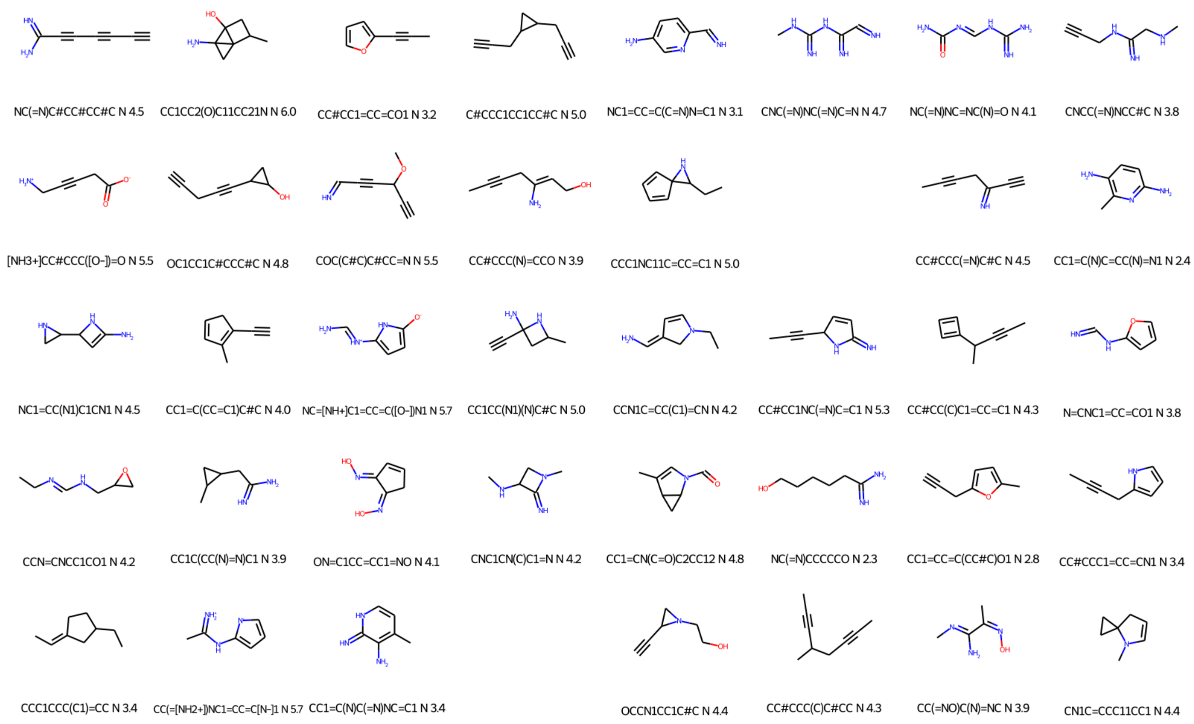

Supplementary Figure 151: Molecules obtained when trying to minimise the overlapping between host (Pd Cage) and guest electron densities, while maximising their electrostatic interactions. The translation from guests to SMILES sequences was done using decorated electron densities. $ed\_factor = 0.3$, $pf\_factor = 1.0$

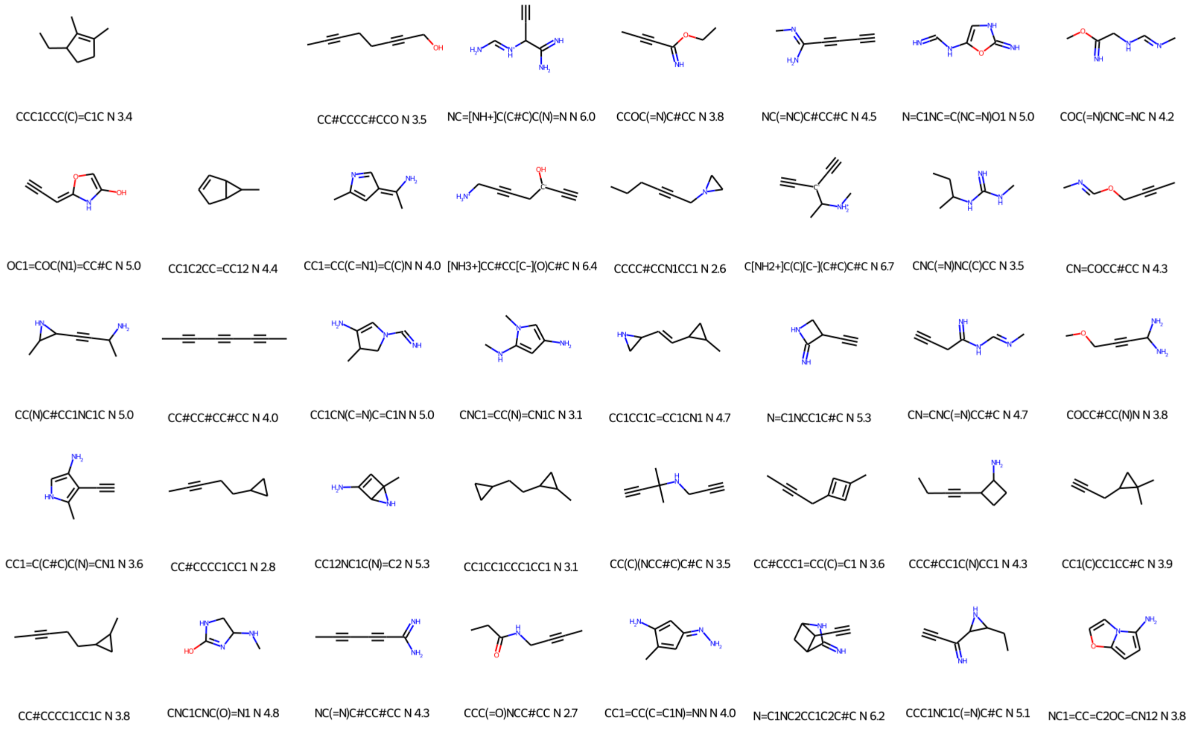

Supplementary Figure 152: Molecules obtained when trying to minimise the overlapping between host (Pd Cage) and guest electron densities, while maximising their electrostatic interactions. The translation from guests to SMILES sequences was done using decorated electron densities. $ed\_factor = 0.3$, $pf\_factor = 2.0$

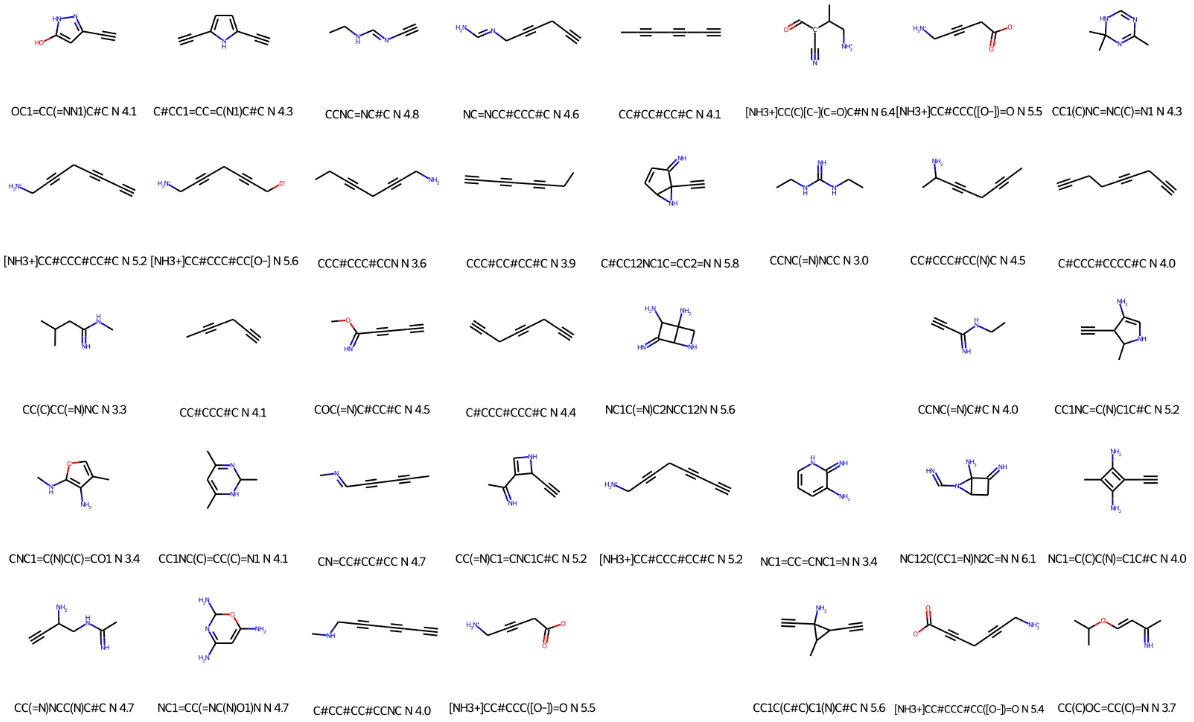

Supplementary Figure 153: Molecules obtained when trying to minimise the overlapping between host (Pd Cage) and guest electron densities, while maximising their electrostatic interactions. The translation from guests to SMILES sequences was done using decorated electron densities. $ed\_factor = 0.3$, $pf\_factor = 10.0$

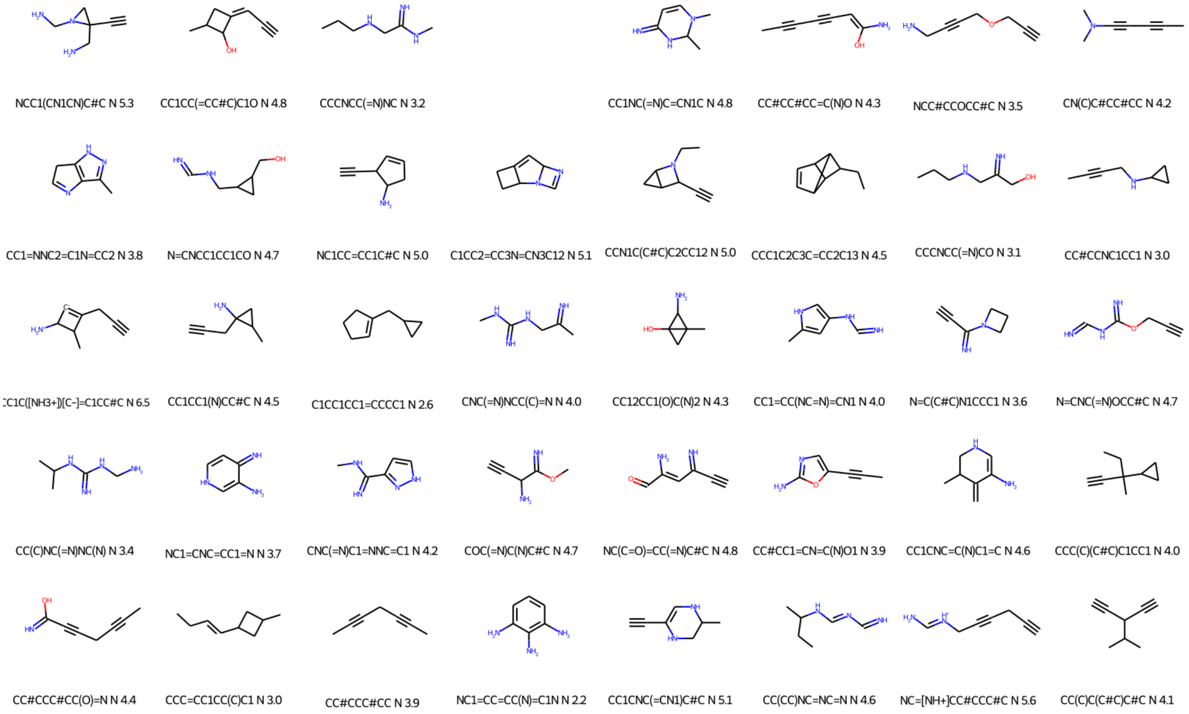

Supplementary Figure 154: Molecules obtained when trying to minimise the overlapping between host (Pd Cage) and guest electron densities, while maximising their electrostatic interactions. The translation from guests to SMILES sequences was done using decorated electron densities. $ed\_factor = 0.6$, $pf\_factor = 0.0$

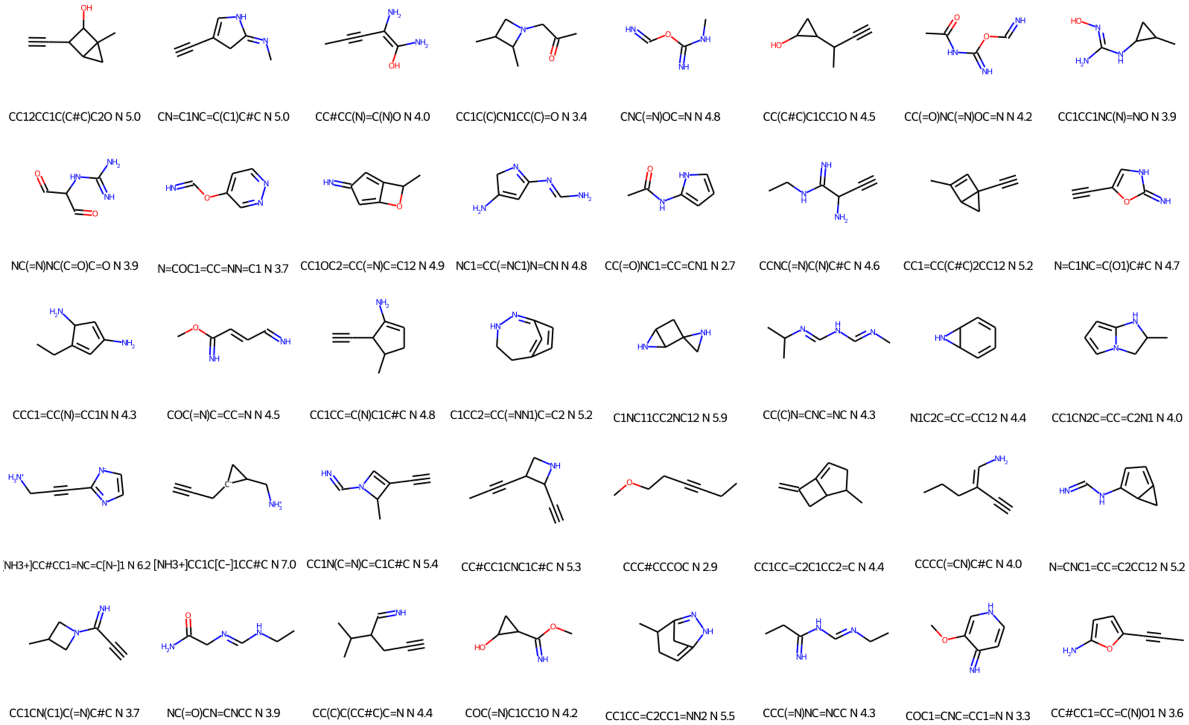

Supplementary Figure 155: Molecules obtained when trying to minimise the overlapping between host (Pd Cage) and guest electron densities, while maximising their electrostatic interactions. The translation from guests to SMILES sequences was done using decorated electron densities. $ed\_factor = 0.6$, $pf\_factor = 0.2$

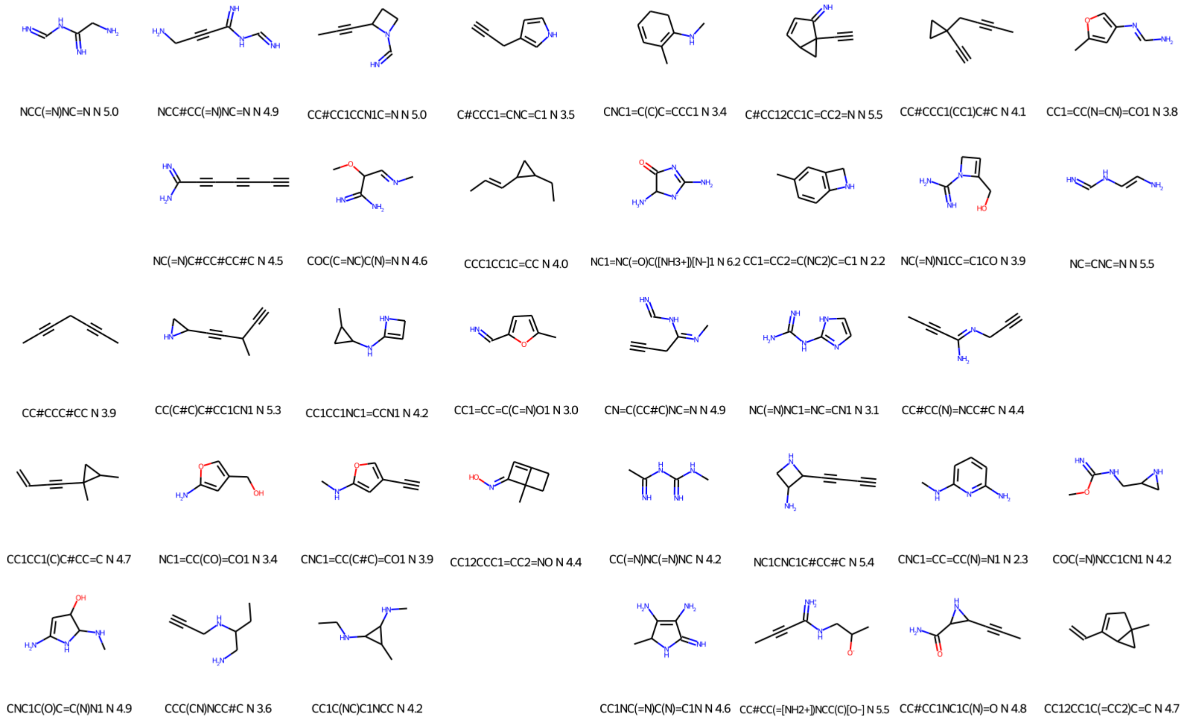

Supplementary Figure 156: Molecules obtained when trying to minimise the overlapping between host (Pd Cage) and guest electron densities, while maximising their electrostatic interactions. The translation from guests to SMILES sequences was done using decorated electron densities. $ed\_factor = 0.6$, $pf\_factor = 0.4$

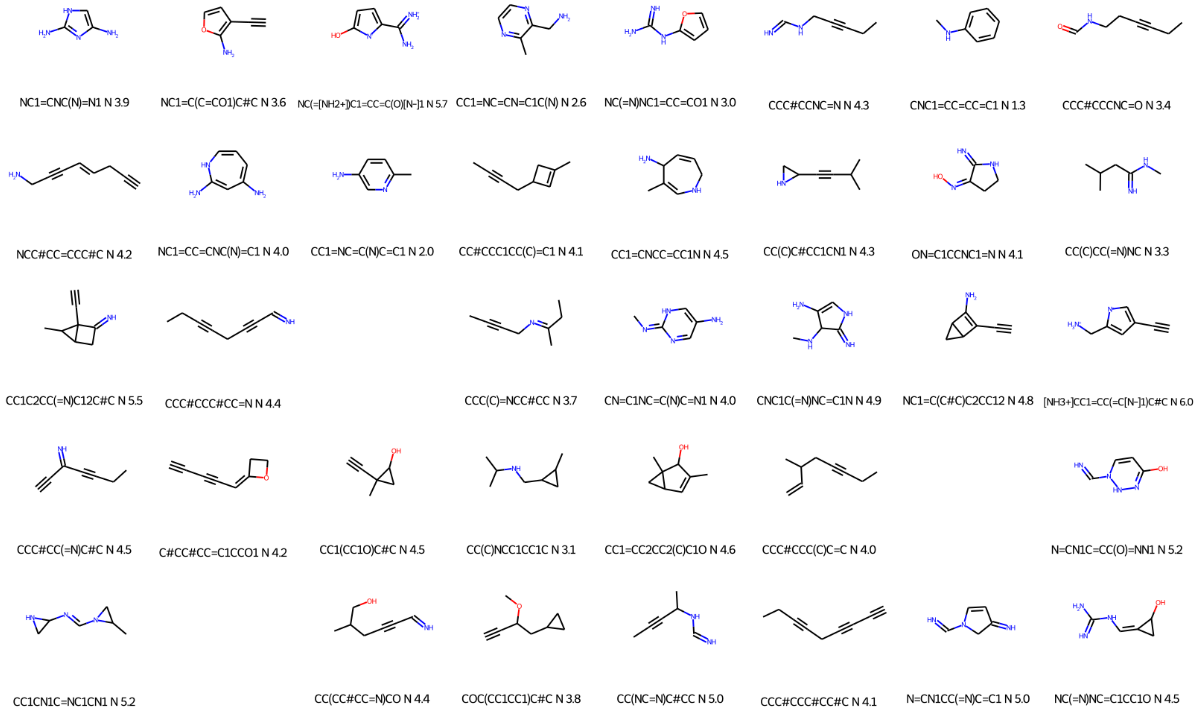

Supplementary Figure 157: Molecules obtained when trying to minimise the overlapping between host (Pd Cage) and guest electron densities, while maximising their electrostatic interactions. The translation from guests to SMILES sequences was done using decorated electron densities. $ed\_factor = 0.6$, $pf\_factor = 0.6$

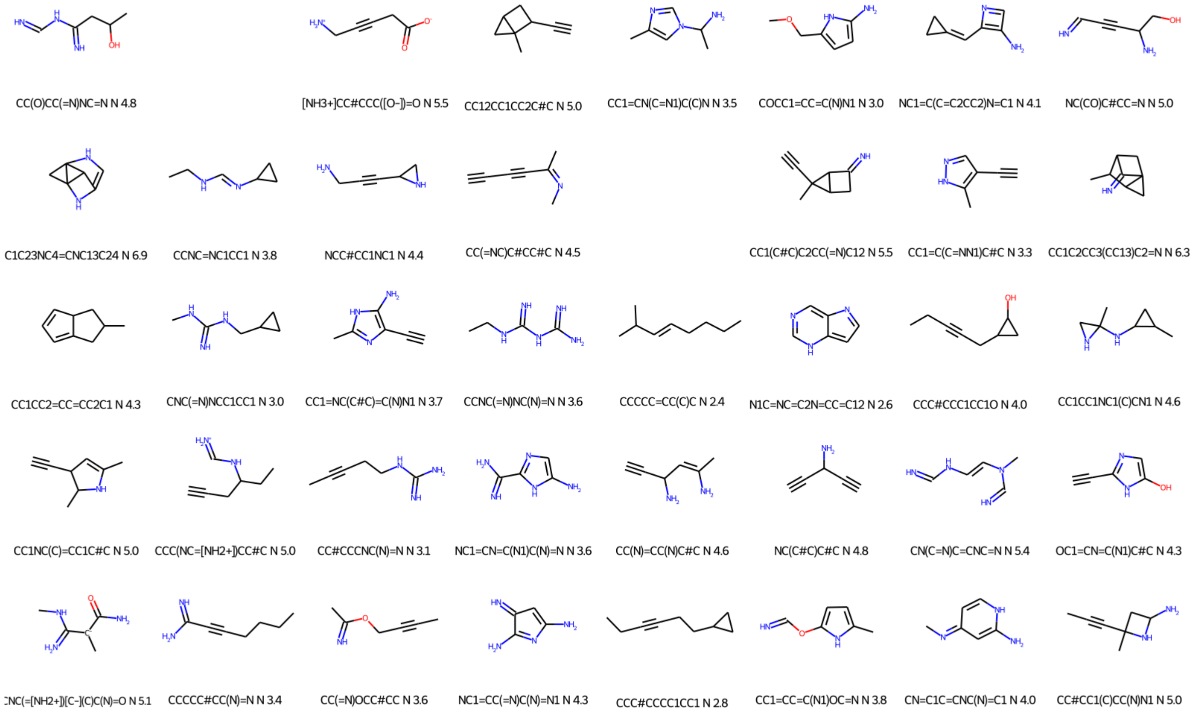

Supplementary Figure 158: Molecules obtained when trying to minimise the overlapping between host (Pd Cage) and guest electron densities, while maximising their electrostatic interactions. The translation from guests to SMILES sequences was done using decorated electron densities. $ed\_factor = 0.6$, $pf\_factor = 0.8$

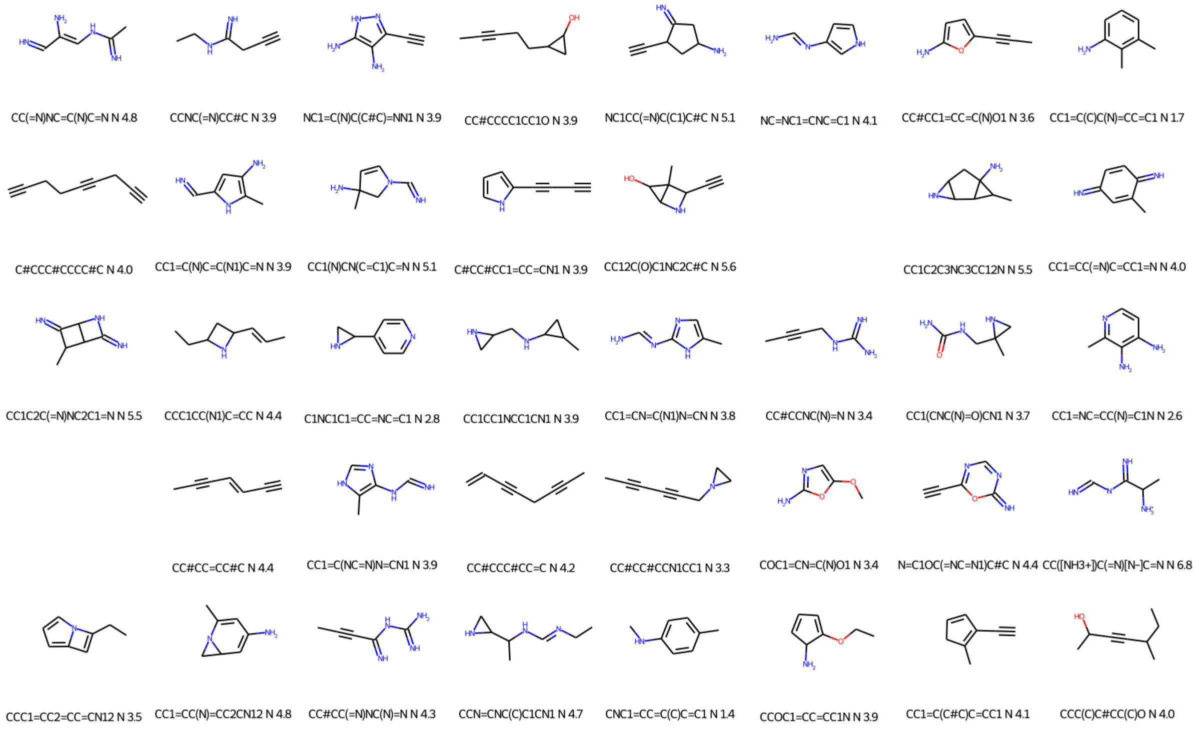

Supplementary Figure 159: Molecules obtained when trying to minimise the overlapping between host (Pd Cage) and guest electron densities, while maximising their electrostatic interactions. The translation from guests to SMILES sequences was done using decorated electron densities. $ed\_factor = 0.6$, $pf\_factor = 1.0$

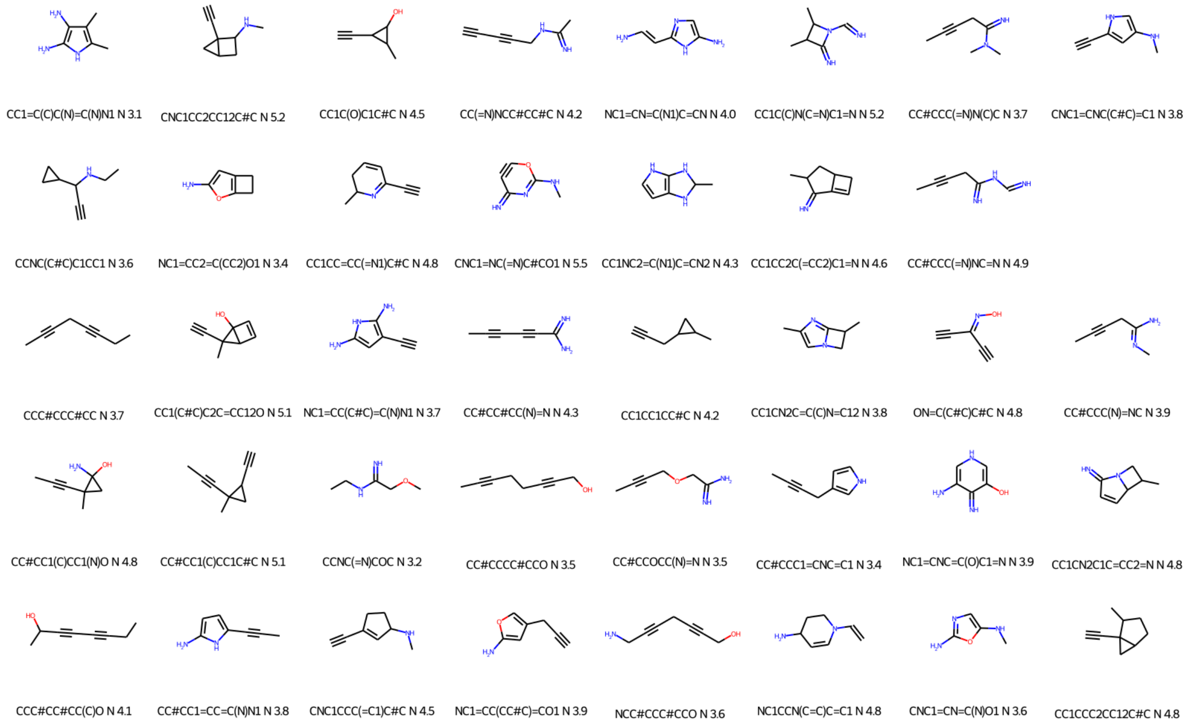

Supplementary Figure 160: Molecules obtained when trying to minimise the overlapping between host (Pd Cage) and guest electron densities, while maximising their electrostatic interactions. The translation from guests to SMILES sequences was done using decorated electron densities. $ed\_factor = 0.6$, $pf\_factor = 2.0$

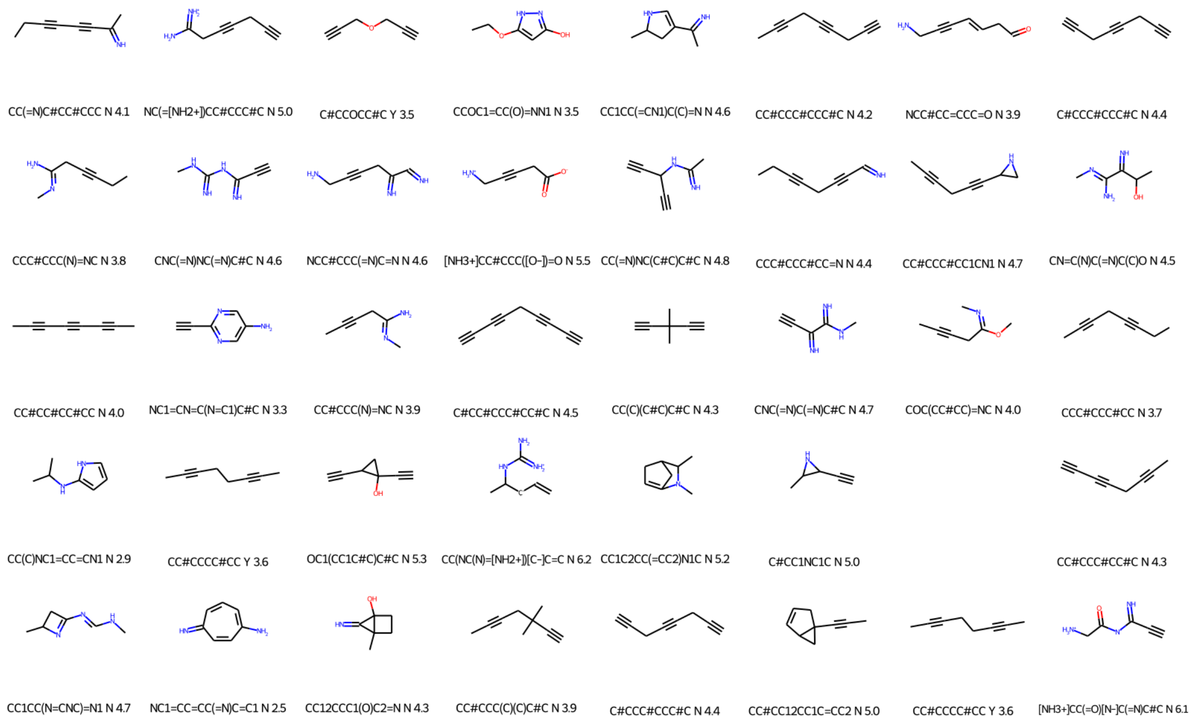

Supplementary Figure 161: Molecules obtained when trying to minimise the overlapping between host (Pd Cage) and guest electron densities, while maximising their electrostatic interactions. The translation from guests to SMILES sequences was done using decorated electron densities. $ed\_factor = 0.6$, $pf\_factor = 10.0$

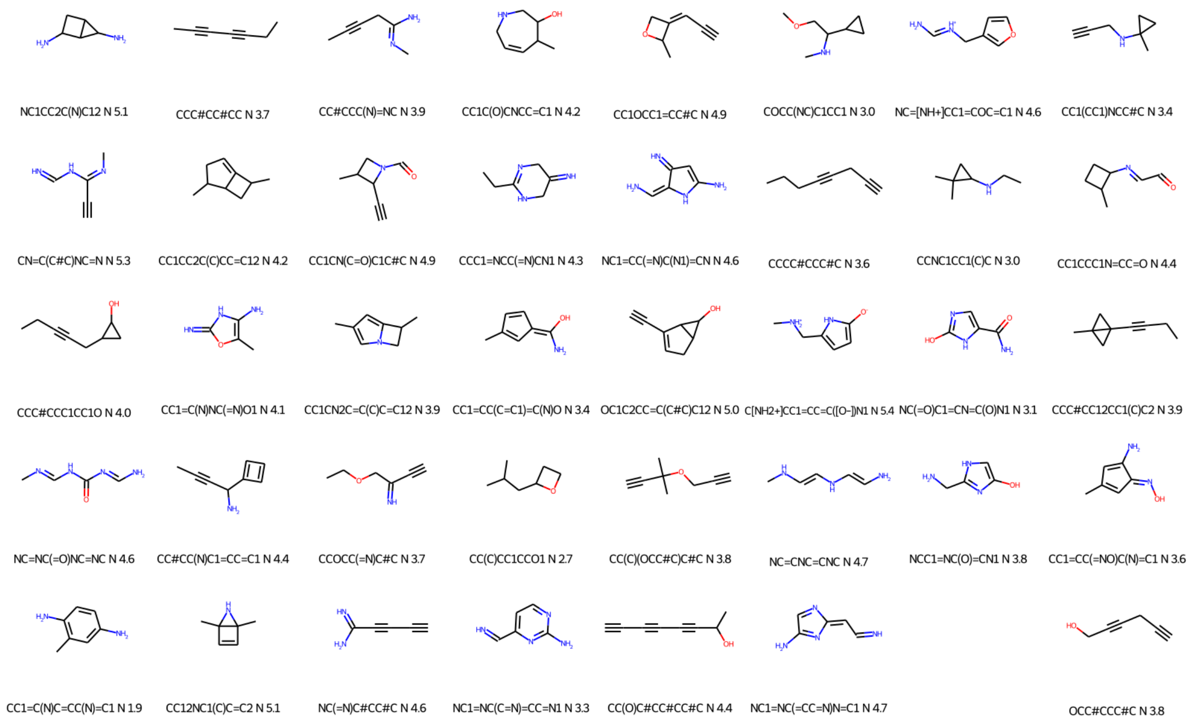

Supplementary Figure 162: Molecules obtained when trying to minimise the overlapping between host (Pd Cage) and guest electron densities, while maximising their electrostatic interactions. The translation from guests to SMILES sequences was done using decorated electron densities. $ed\_factor = 0.9$, $pf\_factor = 0.0$

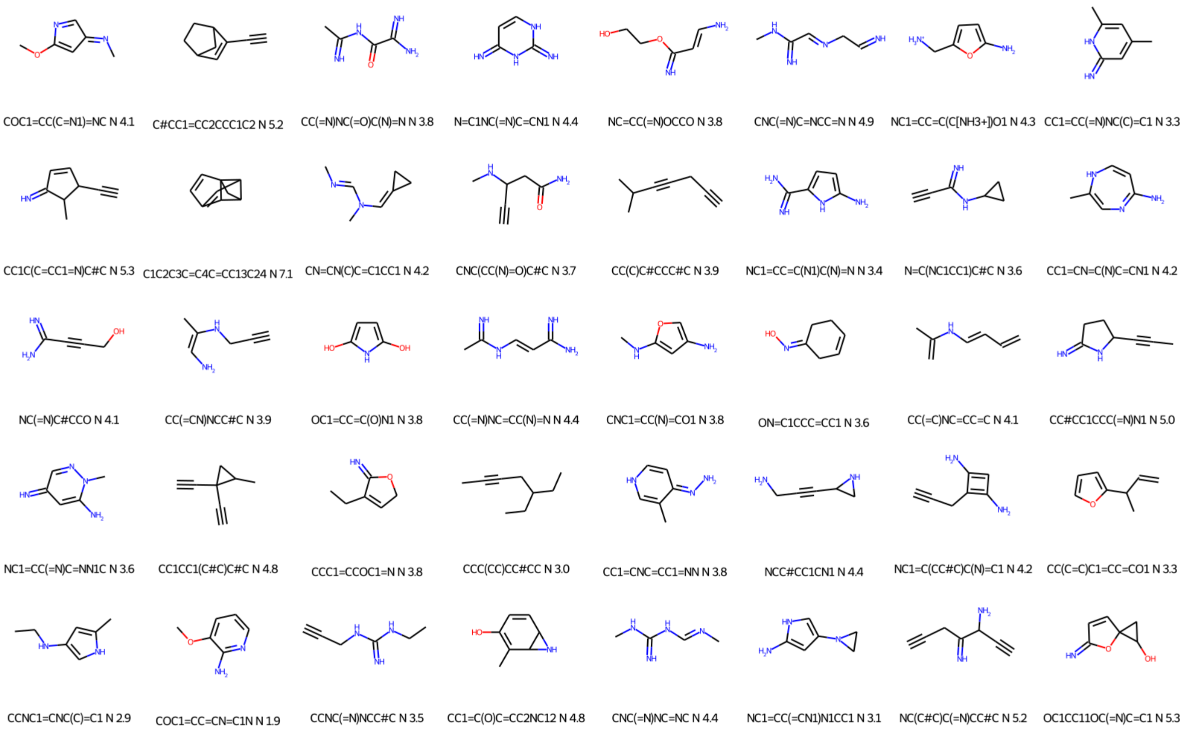

Supplementary Figure 163: Molecules obtained when trying to minimise the overlapping between host (Pd Cage) and guest electron densities, while maximising their electrostatic interactions. The translation from guests to SMILES sequences was done using decorated electron densities. $ed\_factor = 0.9$, $pf\_factor = 0.2$

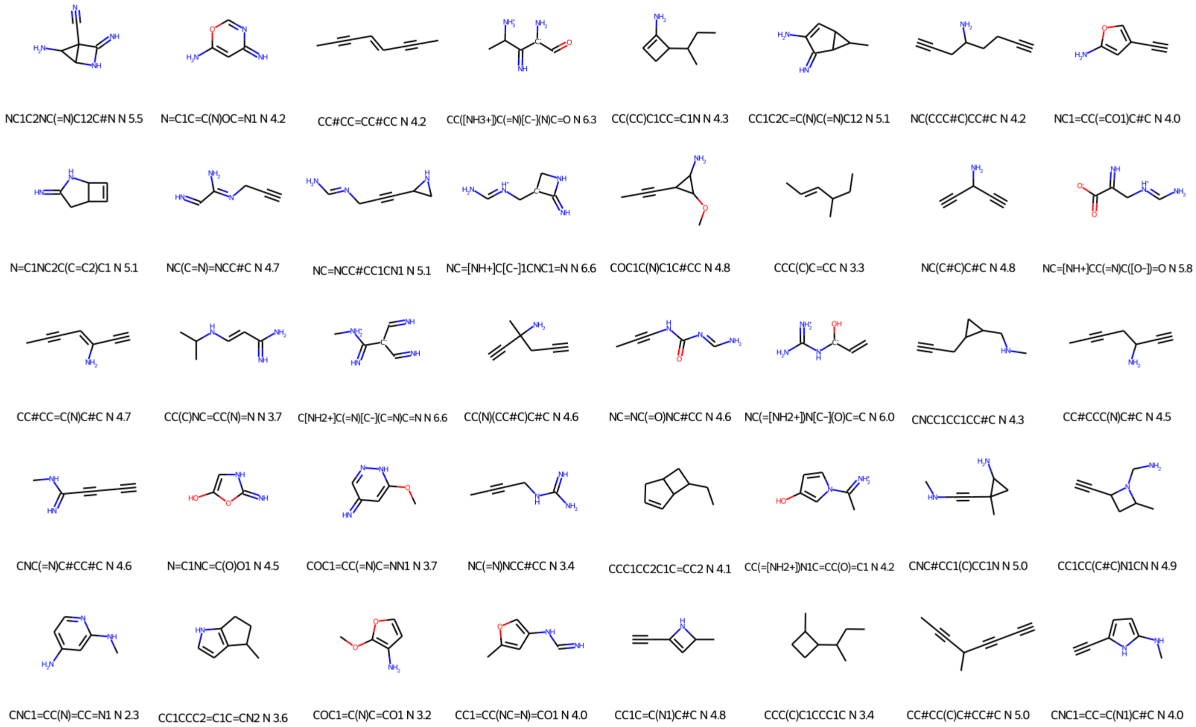

Supplementary Figure 164: Molecules obtained when trying to minimise the overlapping between host (Pd Cage) and guest electron densities, while maximising their electrostatic interactions. The translation from guests to SMILES sequences was done using decorated electron densities. $ed\_factor = 0.9$, $pf\_factor = 0.4$

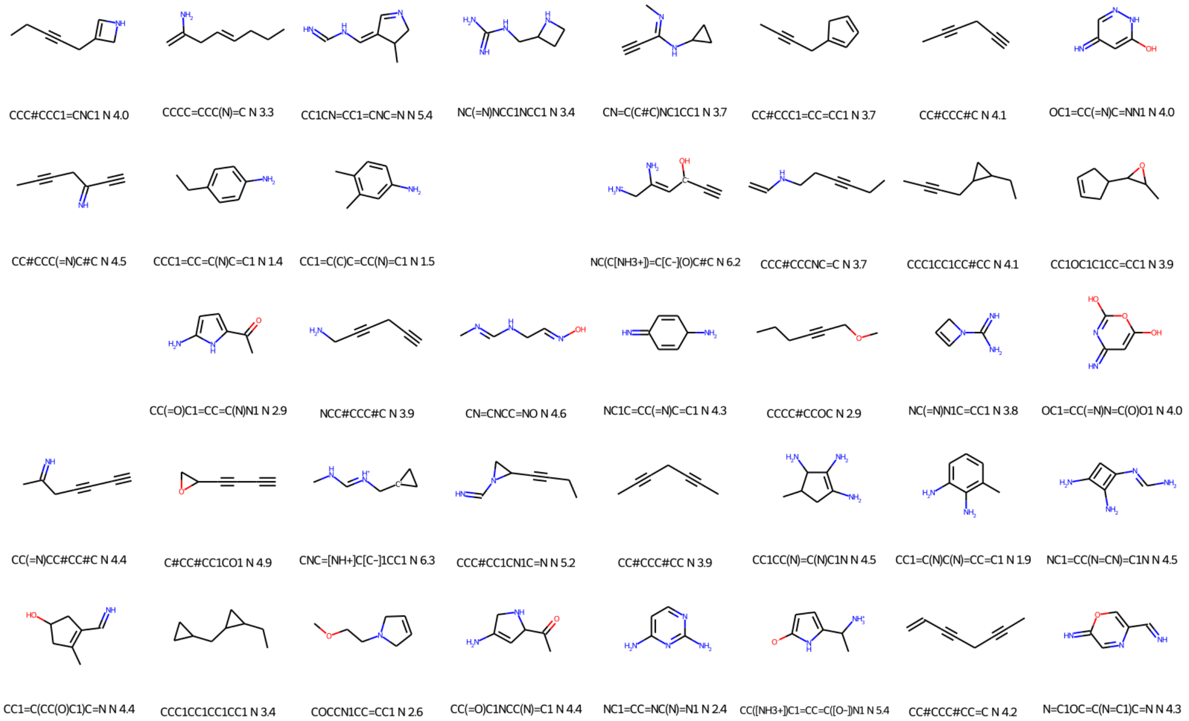

Supplementary Figure 165: Molecules obtained when trying to minimise the overlapping between host (Pd Cage) and guest electron densities, while maximising their electrostatic interactions. The translation from guests to SMILES sequences was done using decorated electron densities. $ed\_factor = 0.9$, $pf\_factor = 0.6$

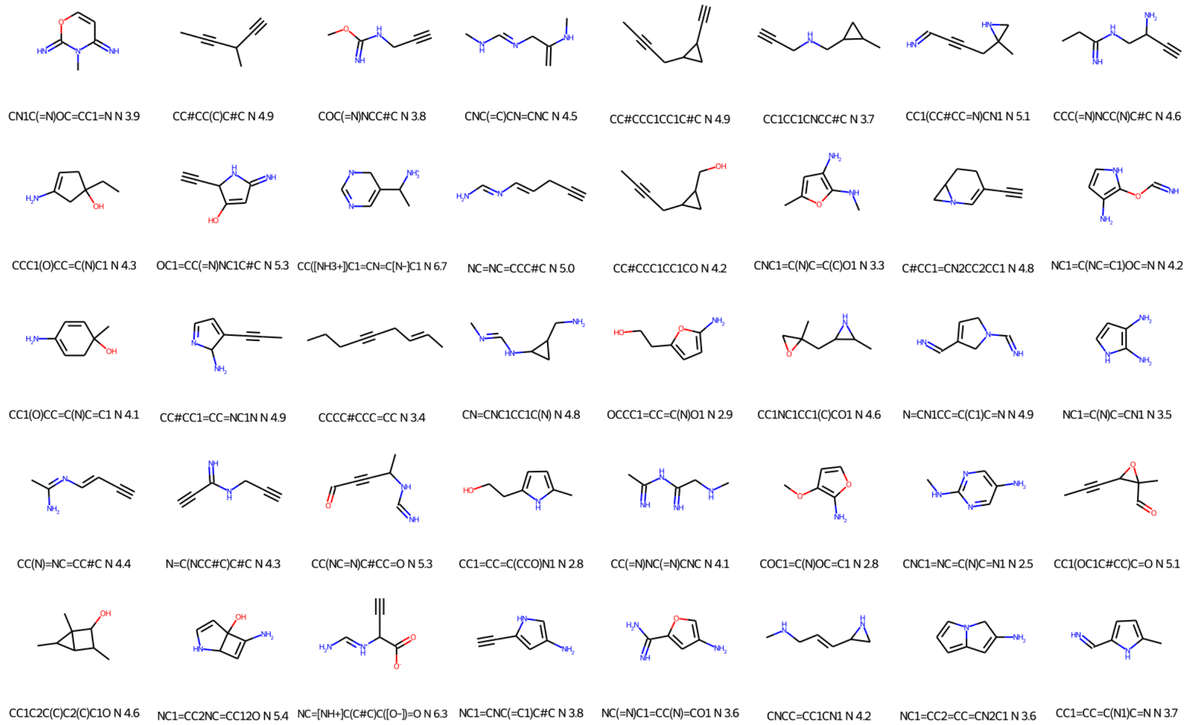

Supplementary Figure 166: Molecules obtained when trying to minimise the overlapping between host (Pd Cage) and guest electron densities, while maximising their electrostatic interactions. The translation from guests to SMILES sequences was done using decorated electron densities. $ed\_factor = 0.9$, $pf\_factor = 0.8$

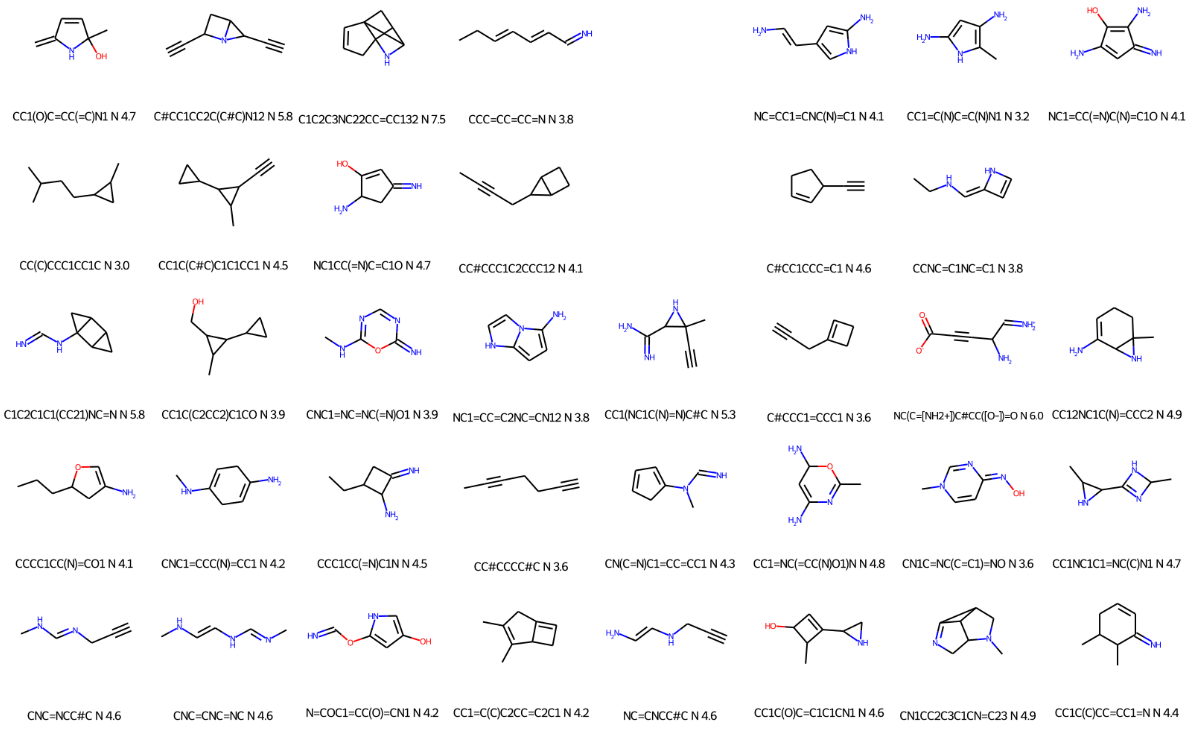

Supplementary Figure 167: Molecules obtained when trying to minimise the overlapping between host (Pd Cage) and guest electron densities, while maximising their electrostatic interactions. The translation from guests to SMILES sequences was done using decorated electron densities. $ed\_factor = 0.9$, $pf\_factor = 1.0$

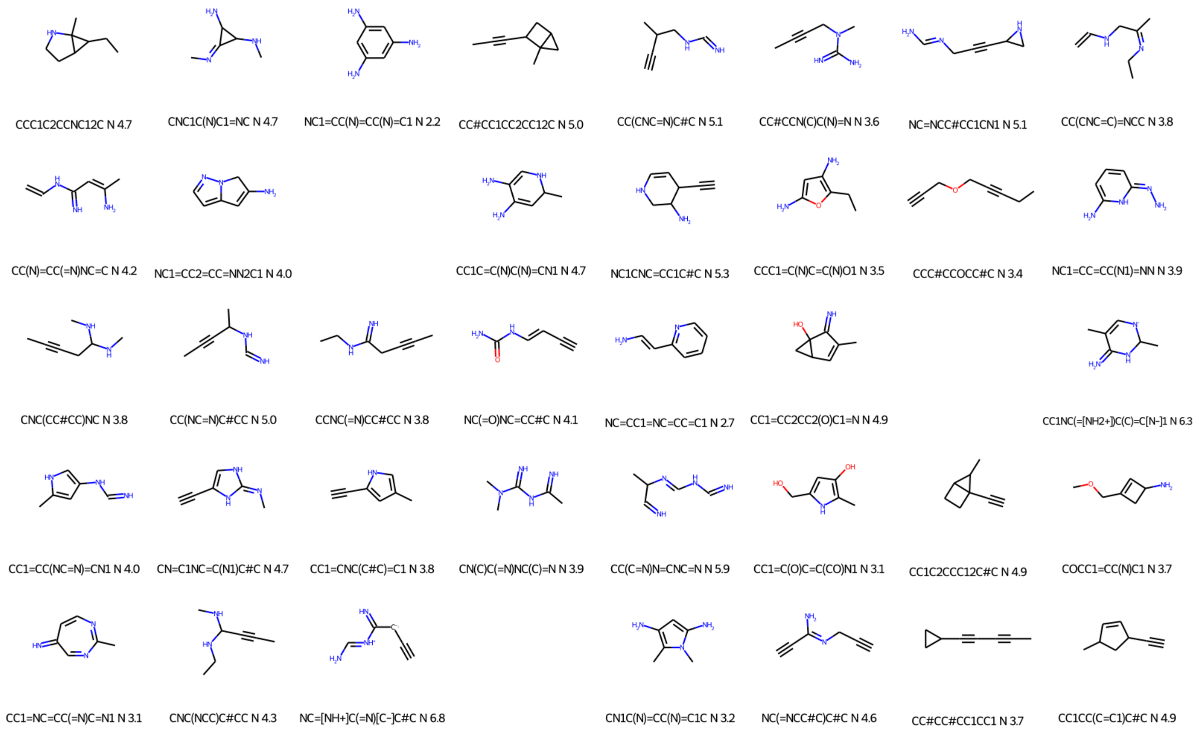

Supplementary Figure 168: Molecules obtained when trying to minimise the overlapping between host (Pd Cage) and guest electron densities, while maximising their electrostatic interactions. The translation from guests to SMILES sequences was done using decorated electron densities. $ed\_factor = 0.9$, $pf\_factor = 2.0$

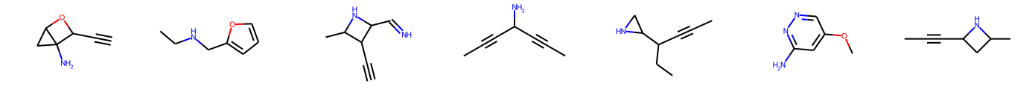

NC12CC1OC2C#C N 5.7 | CCNCC1=CC=CO1 N 1.9 | CC1NC(C=N)C1C#C N 5.8 | CC#CC(N)C#CC N 4.4 | CCC(C#CC)C1CN1 N 4.6 | COC1=CC(N)=NN=C1 N 2.7 | CC1CC(N1)C#CC N 4.6

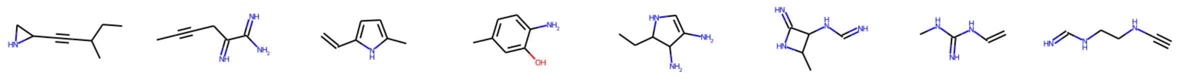

CCC(C)C#CC1CN1 N 4.5 | CC#CCC(=N)C(N)=N N 4.2 | CC1=CC=C(N1)C=C N 3.2 | CC1=CC=C(N)C(O)=C1 N 1.9 | CCC1C(N)C(N)=CN1 N 4.8 | CC1NC(=N)C1NC=N N 5.6 | CNC(=N)NC=C N 4.2 | N=CNCCNC#C N 5.0

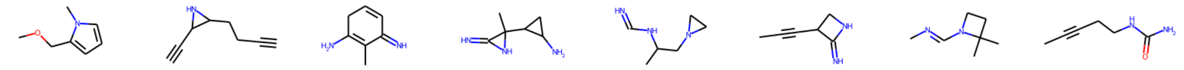

COCC1=CC=CN1C N 2.4 | C#CCCC1NC1C#C N 5.2 | CC1=C(N)CC=CC1=N N 3.9 | CC1(NC1=N)C1CC1N N 4.8 | CC(CN1CC1)NC=N N 3.8 | CC#CC1CNC1=N N 5.2 | CN=CN1CCC1(C)C N 4.2 | NC(=O)NCCC#CC N 2.8

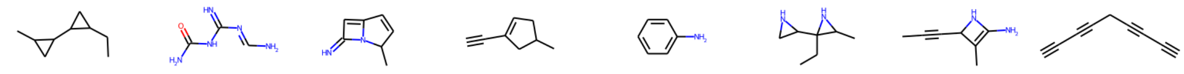

CCC1CC1C1CC1C N 3.9 | NC(=O)NC(=N)N=CN N 4.1 | CC1C=CC2=CC(=N)N12 N 4.8 | CC1CC=C(C#C)C1 N 4.2 | NC1=CC=CC=C1 N 1.3 | CC1NC1(CC)C1CN1 N 4.8 | CC#CC1NC(N)=C1C N 4.7 | C#CC#CCC#CC#C N 4.5

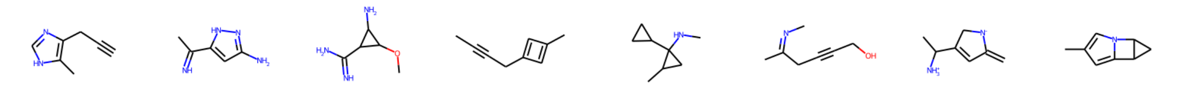

CC1=C(CC#C)N=CN1 N 3.4 | CC(=N)C1=CC(N)=NN1 N 3.4 | COC1C(N)C1C(N)=N N 4.6 | CC#CCC1=CC(C)=C1 N 3.6 | CNC1(CC1C)C1CC1 N 4.1 | CC(CC#CCO)=NC N 3.8 | CC([NH3+])C1=CC(=C)[N-]C1 N 6.5 | CC1=CN2C3CC3C2=C1 N 4.3

Supplementary Figure 169: Molecules obtained when trying to minimise the overlapping between host (Pd Cage) and guest electron densities, while maximising their electrostatic interactions. The translation from guests to SMILES sequences was done using decorated electron densities. $ed\_factor = 0.9$, $pf\_factor = 10.0$

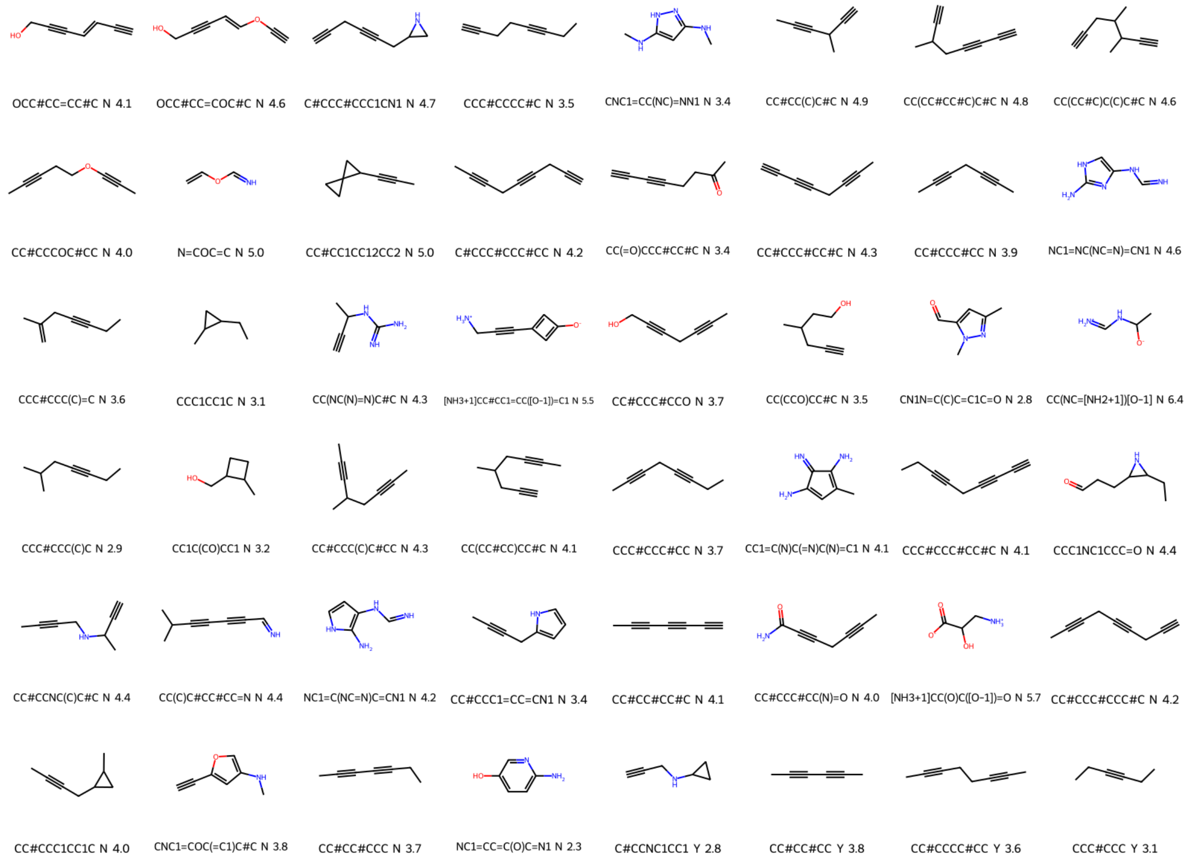

Supplementary Figure 170: Molecules obtained when trying to minimise the overlapping between host (CB6) and guest electron densities, while maximising their electrostatic interactions. The volume of the cavity was reduced with a kernel of size 3. $ed\_factor = 0$

Please note the following Supplementary Figures display the obtained guests and their related SMILES representation. To the best of our knwoledge "rdkit" (the library we used to generate the figures) does not work with SELFIES. Therefore, once the SELFIES were obtained, they were transformed into SMILES, and these SMILES were passed to "rdkit" to generate the figures. The results can be seen in Supplementary Figures 170 to 195.

2.3.11 SELFIES results from bin/optimisers/cage_hg_esp_lee.py

These results were obtained using the `cage_hg_esp_lee.py` script described in Supplementary Section 2.2.8. A total of 19 experiments/runs were done using this script using different values for `ed_factor`. This generated 760 different guests. These were converted into SELFIES sequences with the method using decorated electron densities described in Supplementary Section 1.8. Please note the following Supplementary Figures display the obtained guests and their related SMILES representation. To the best of our knwoledge "rdkit" (the library we used to generate the figures) does not work with SELFIES. Therefore, once the SELFIES were obtained, they were transformed into SMILES, and these SMILES were passed to "rdkit" to generate the figures. The results can be seen in Supplementary Figures 196 to 214.

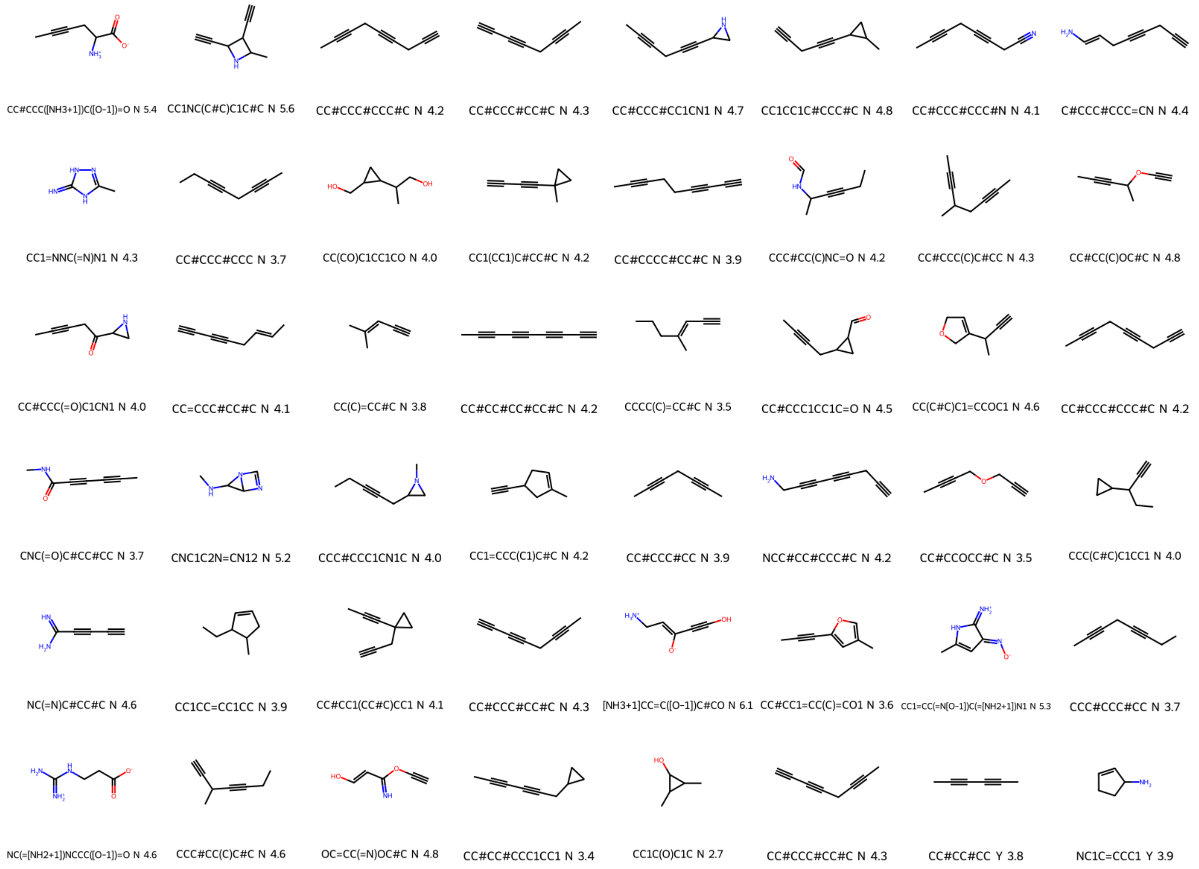

Supplementary Figure 171: Molecules obtained when trying to minimise the overlapping between host (CB6) and guest electron densities, while maximising their electrostatic interactions. The volume of the cavity was reduced with a kernel of size 3. $ed\_factor = 0.05$

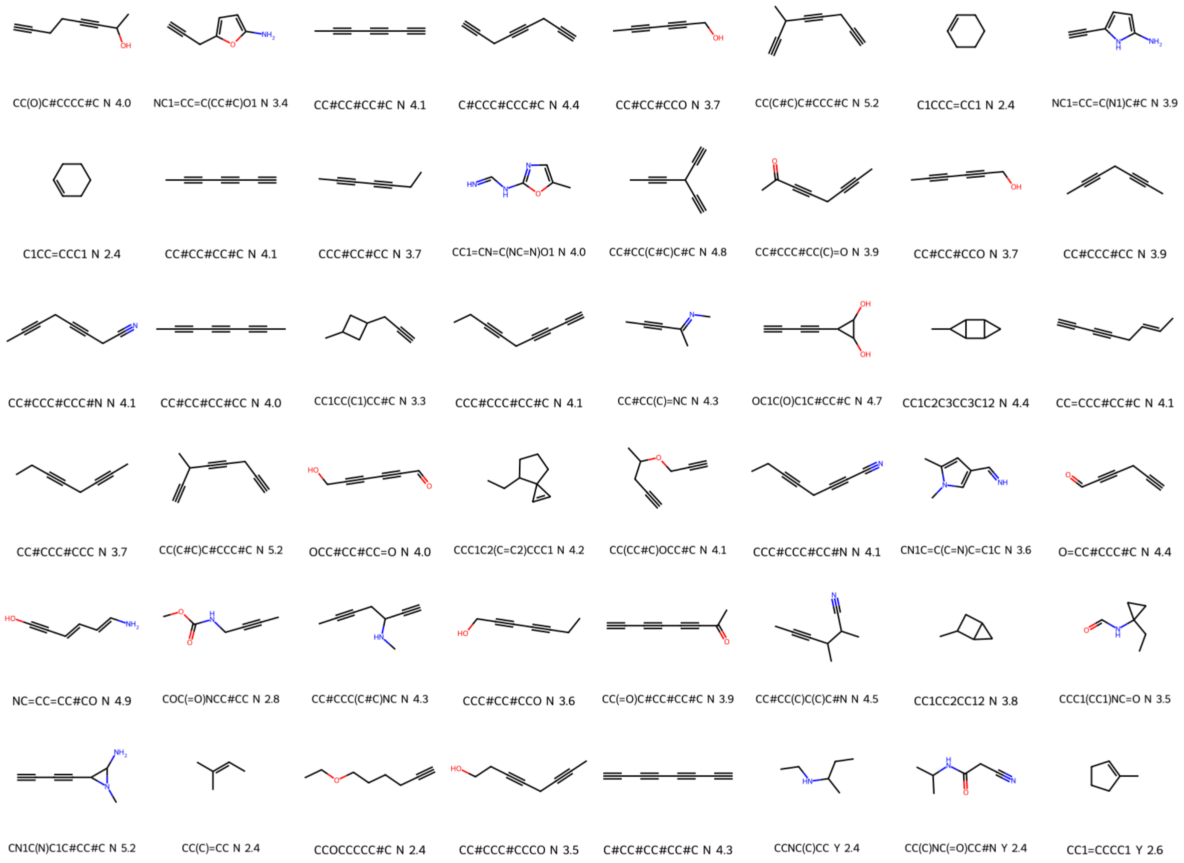

Supplementary Figure 172: Molecules obtained when trying to minimise the overlapping between host (CB6) and guest electron densities, while maximising their electrostatic interactions. The volume of the cavity was reduced with a kernel of size 3. $ed\_factor = 0.1$

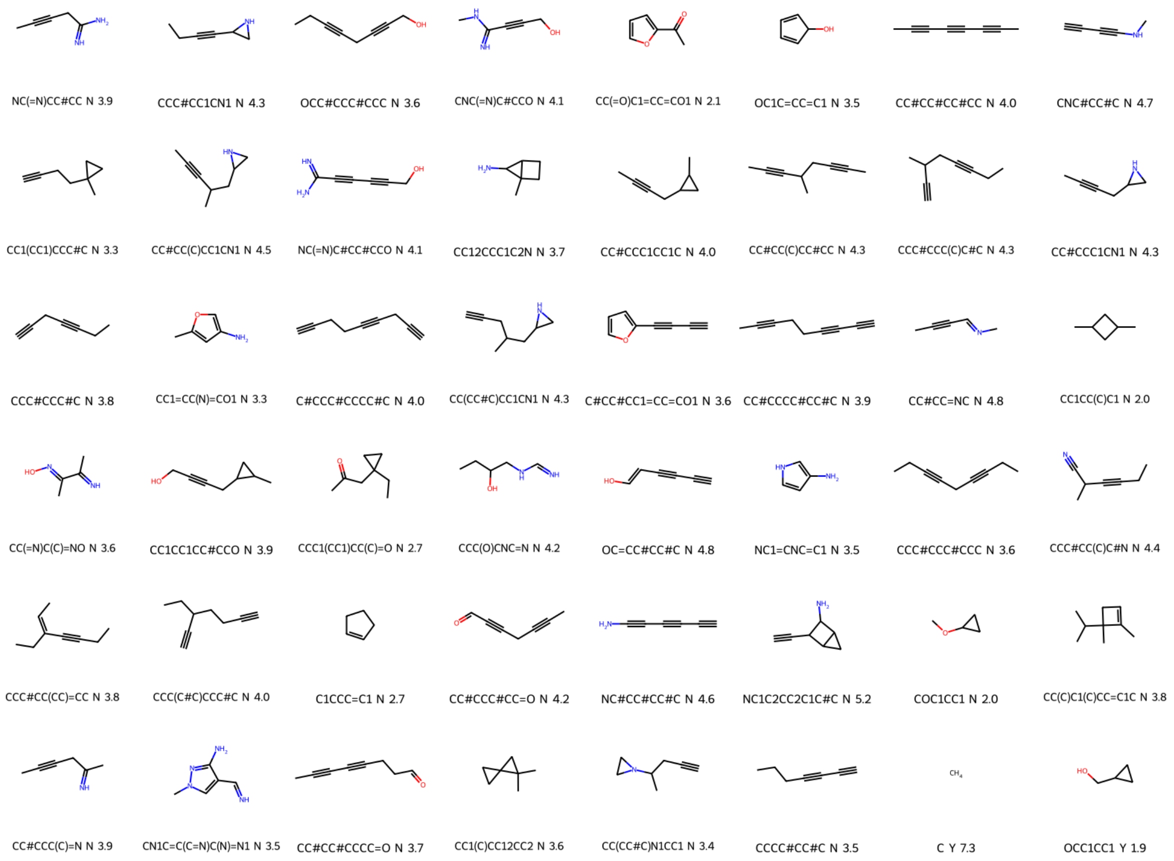

Supplementary Figure 173: Molecules obtained when trying to minimise the overlapping between host (CB6) and guest electron densities, while maximising their electrostatic interactions. The volume of the cavity was reduced with a kernel of size 3. $ed\_factor = 0.2$

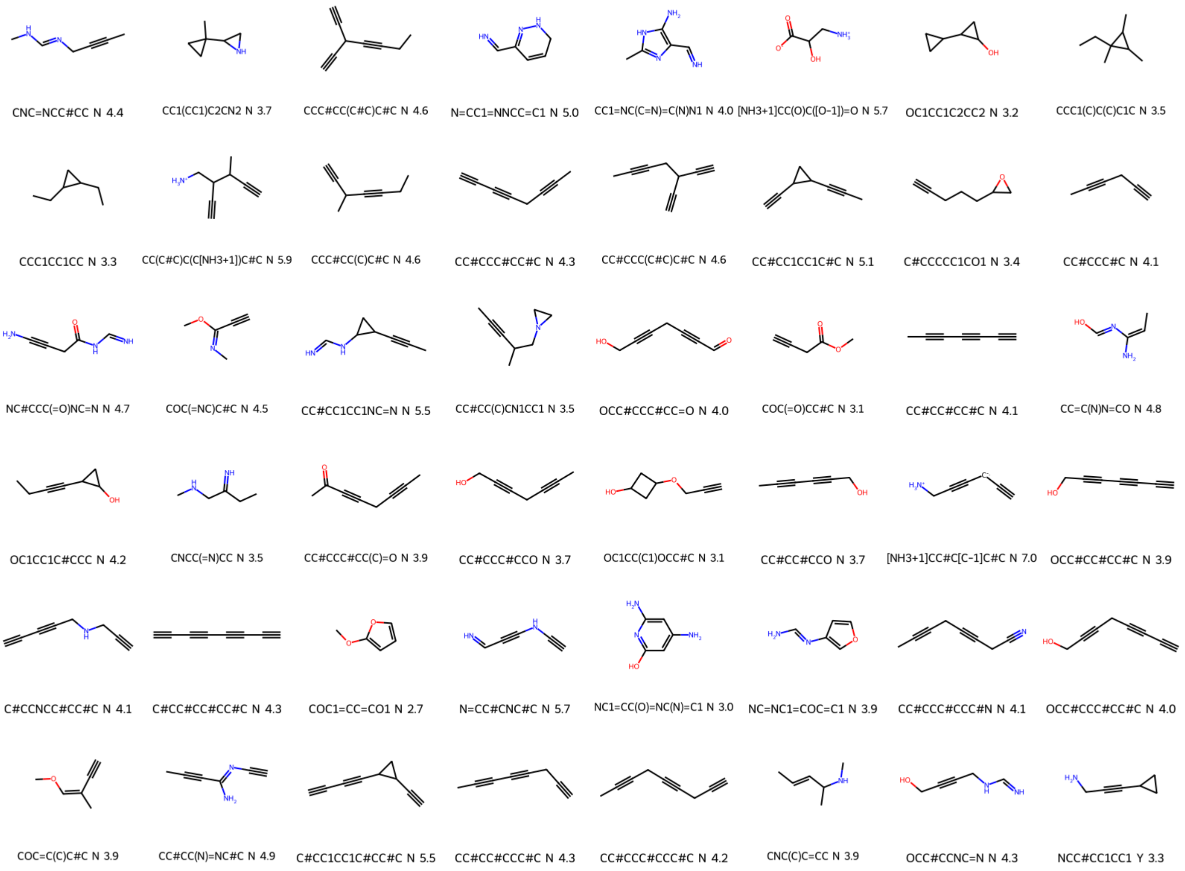

Supplementary Figure 174: Molecules obtained when trying to minimise the overlapping between host (CB6) and guest electron densities, while maximising their electrostatic interactions. The volume of the cavity was reduced with a kernel of size 3. $ed\_factor = 0.3$

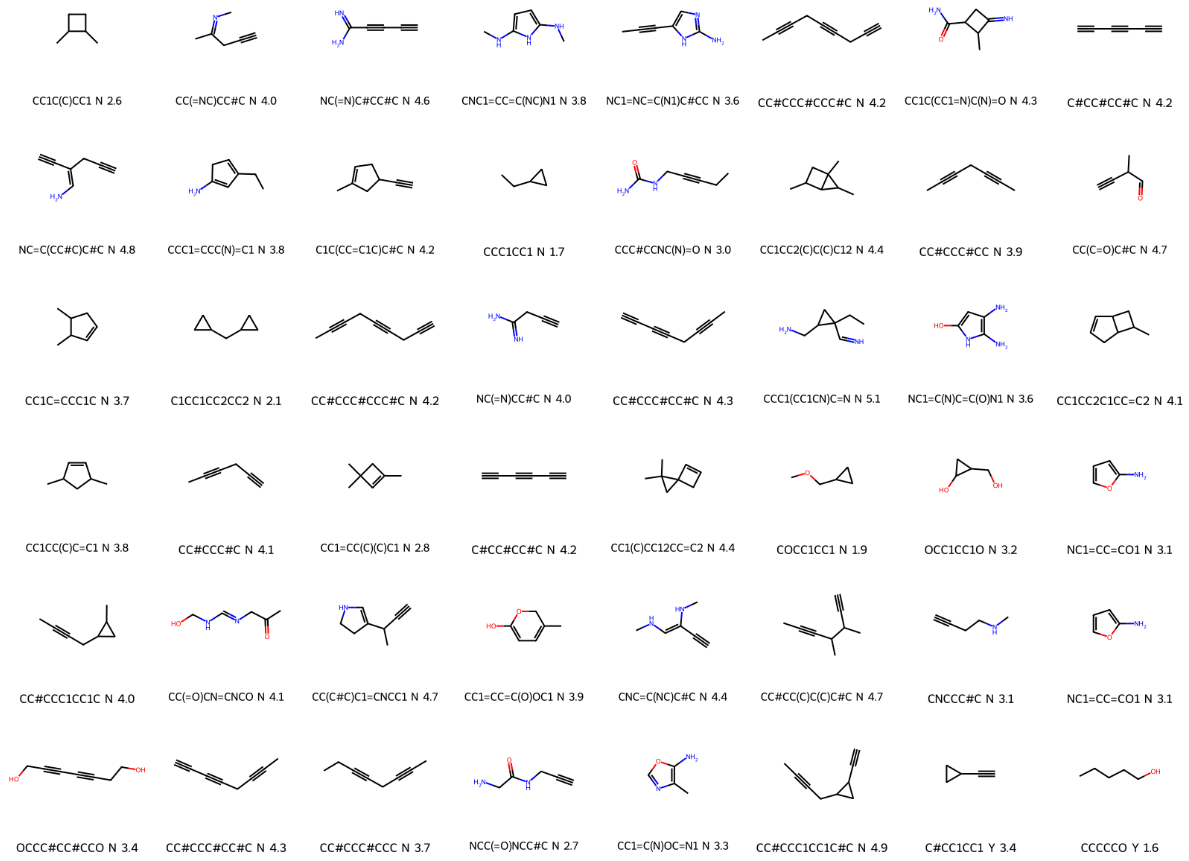

Supplementary Figure 175: Molecules obtained when trying to minimise the overlapping between host (CB6) and guest electron densities, while maximising their electrostatic interactions. The volume of the cavity was reduced with a kernel of size 3. $ed\_factor = 0.4$

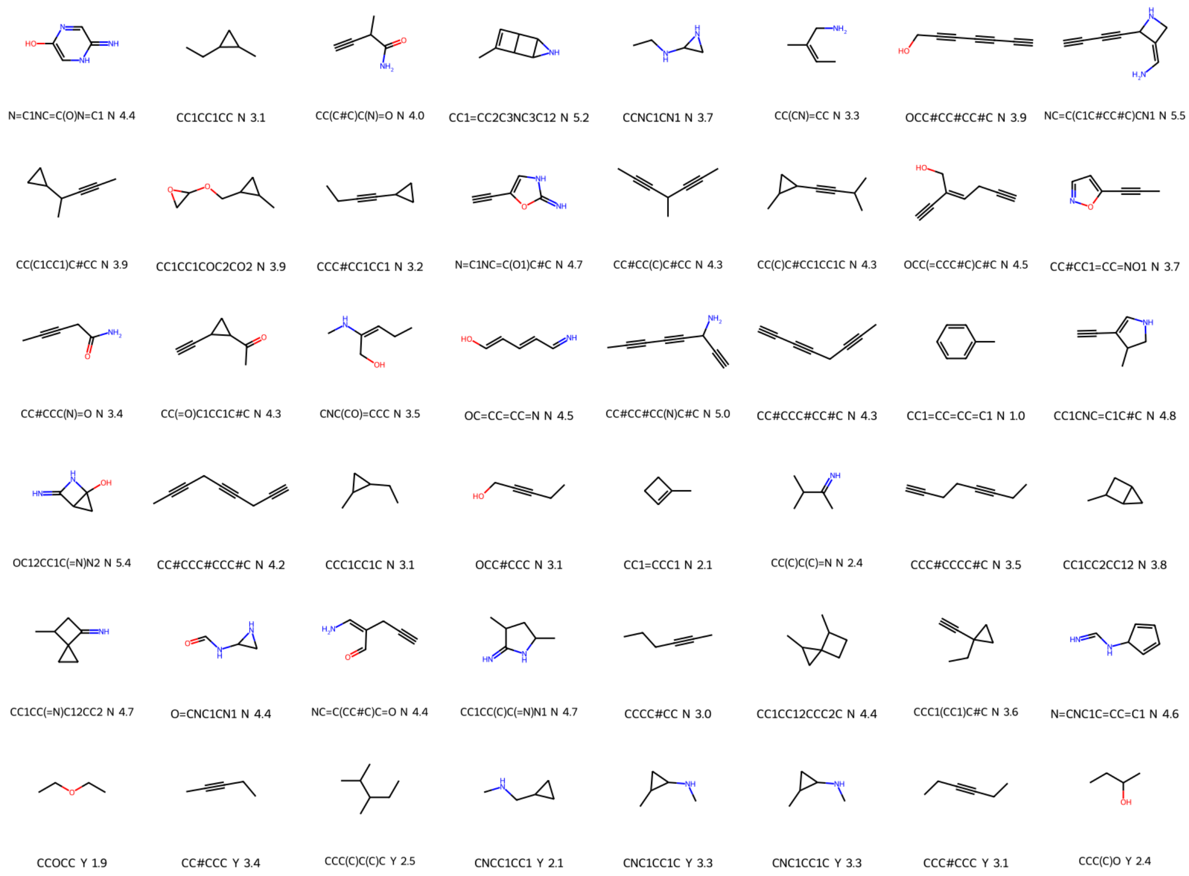

Supplementary Figure 176: Molecules obtained when trying to minimise the overlapping between host (CB6) and guest electron densities, while maximising their electrostatic interactions. The volume of the cavity was reduced with a kernel of size 3. $ed\_factor = 0.5$

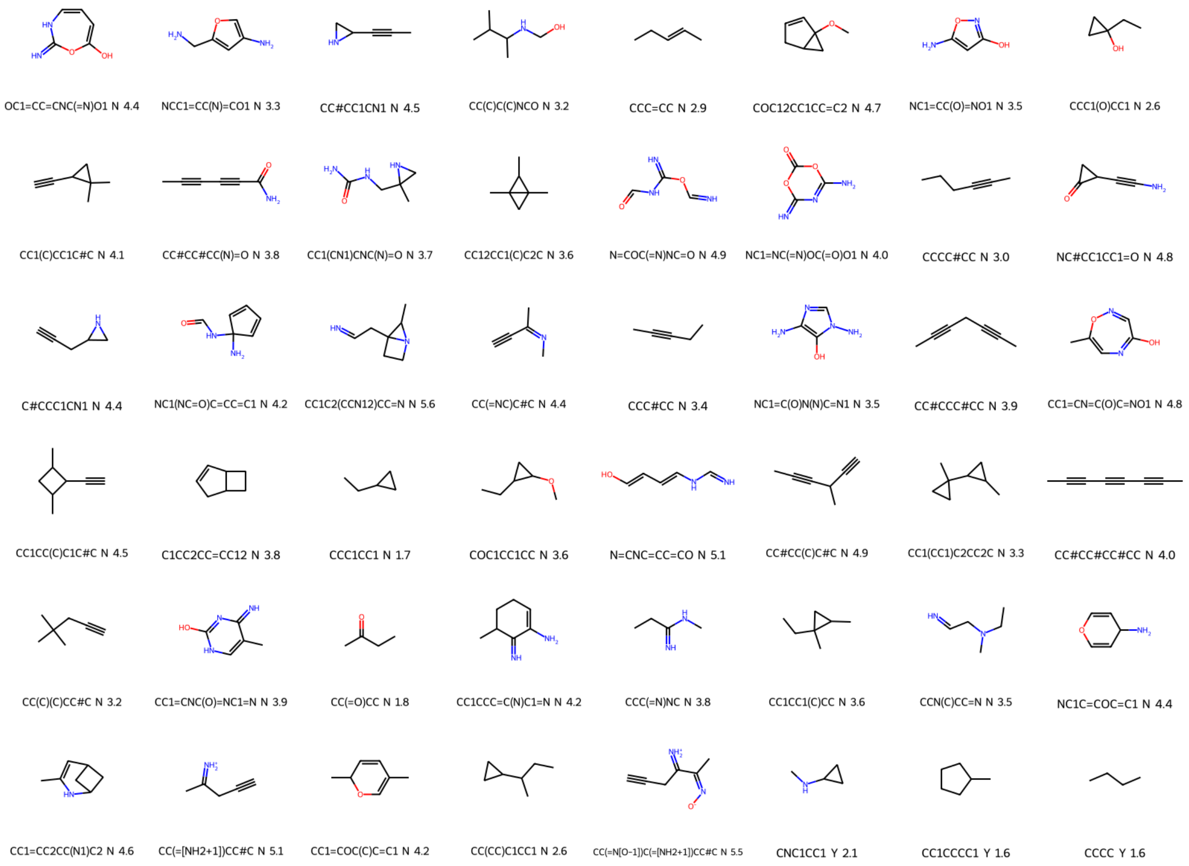

Supplementary Figure 177: Molecules obtained when trying to minimise the overlapping between host (CB6) and guest electron densities, while maximising their electrostatic interactions. The volume of the cavity was reduced with a kernel of size 3. $ed\_factor = 0.6$

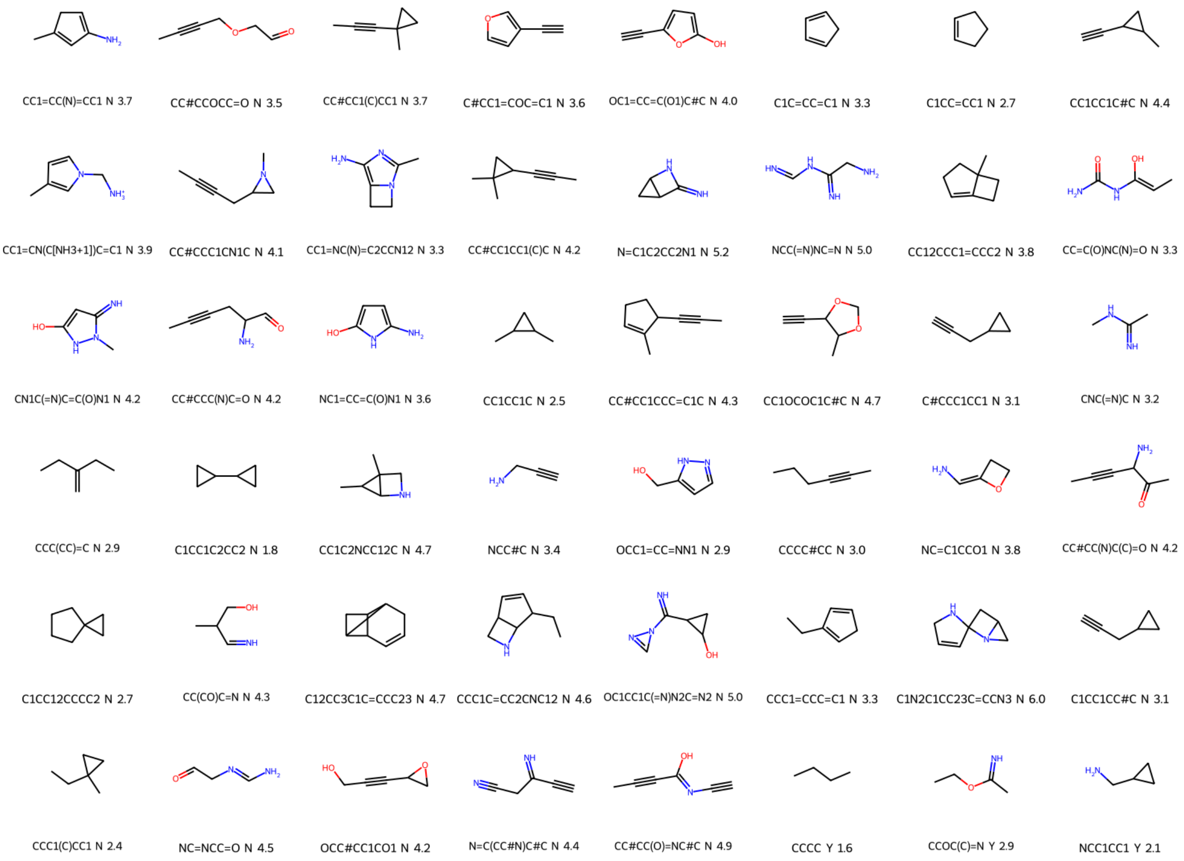

Supplementary Figure 178: Molecules obtained when trying to minimise the overlapping between host (CB6) and guest electron densities, while maximising their electrostatic interactions. The volume of the cavity was reduced with a kernel of size 3. $ed\_factor = 0.7$

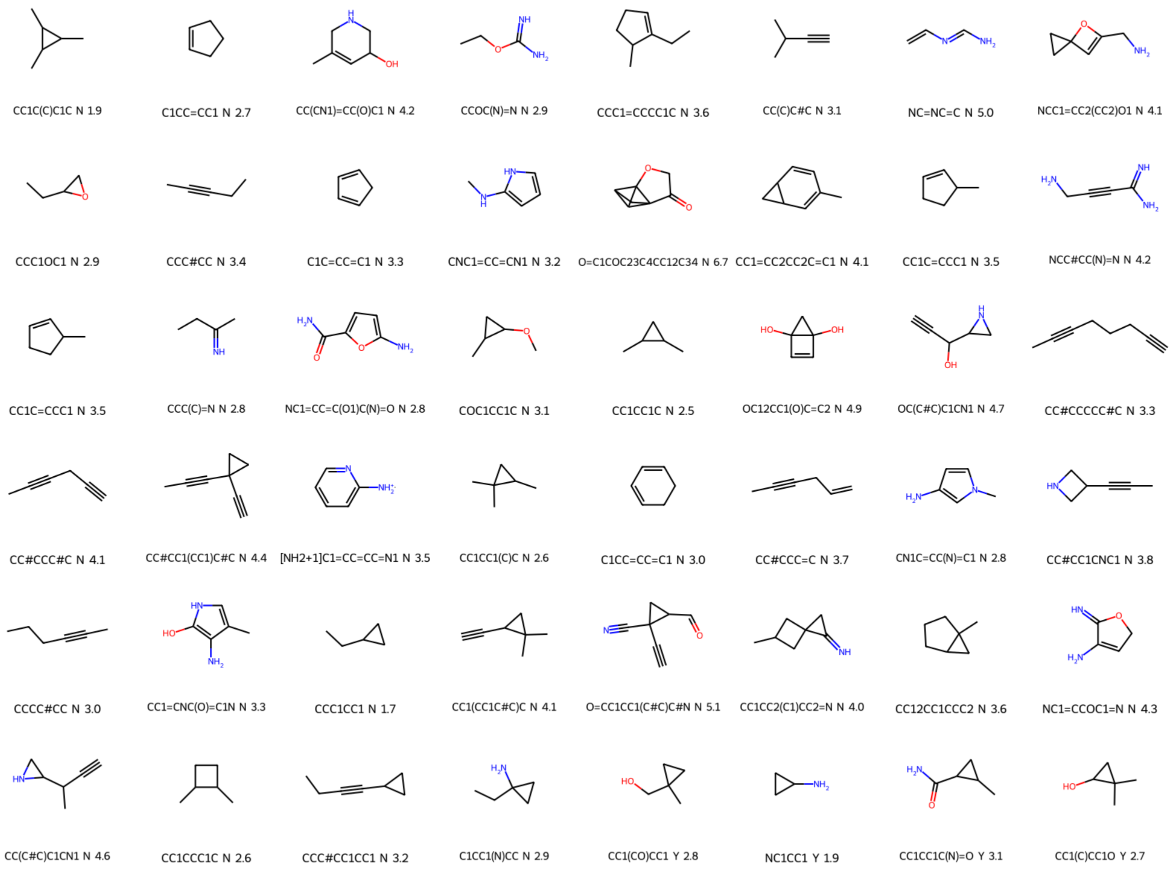

Supplementary Figure 179: Molecules obtained when trying to minimise the overlapping between host (CB6) and guest electron densities, while maximising their electrostatic interactions. The volume of the cavity was reduced with a kernel of size 3. $ed\_factor = 0.8$

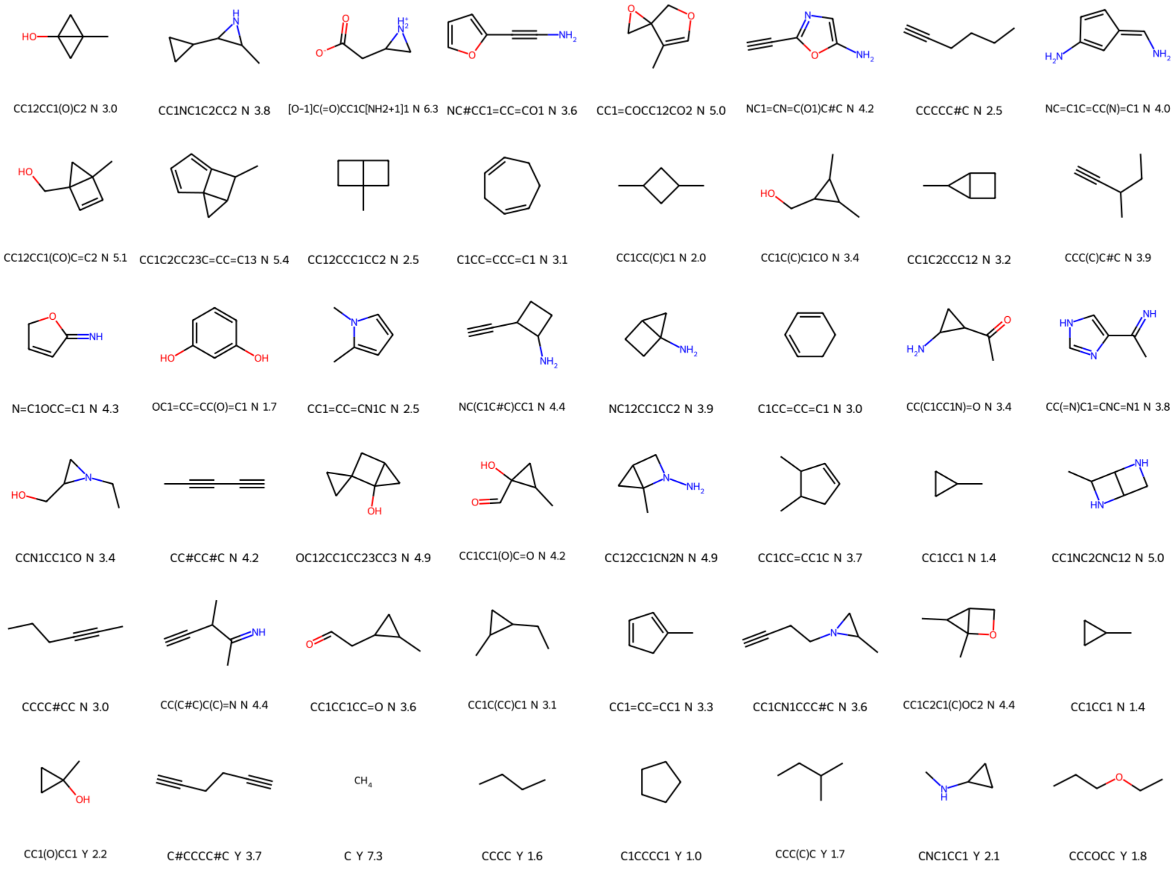

Supplementary Figure 180: Molecules obtained when trying to minimise the overlapping between host (CB6) and guest electron densities, while maximising their electrostatic interactions. The volume of the cavity was reduced with a kernel of size 3. $ed\_factor = 0.9$

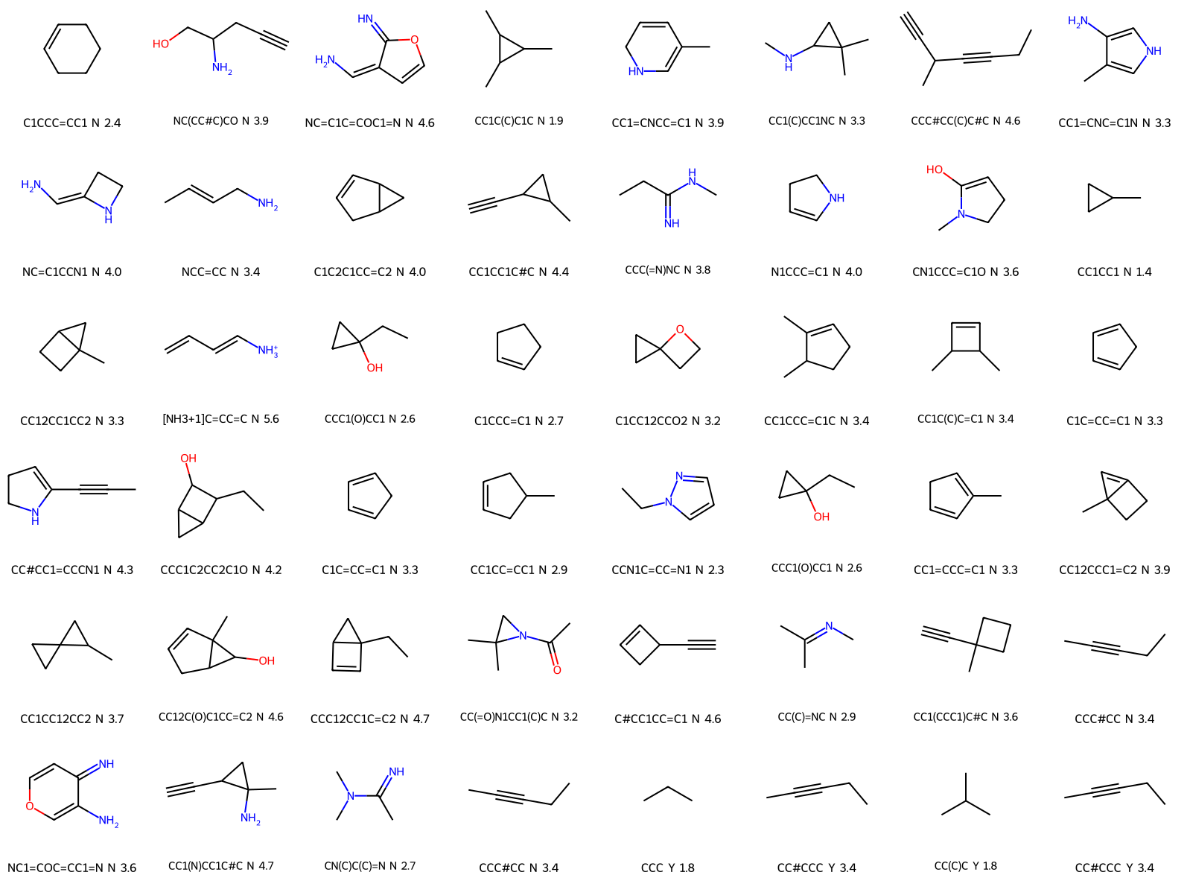

Supplementary Figure 181: Molecules obtained when trying to minimise the overlapping between host (CB6) and guest electron densities, while maximising their electrostatic interactions. The volume of the cavity was reduced with a kernel of size 3. $ed\_factor = 0.95$

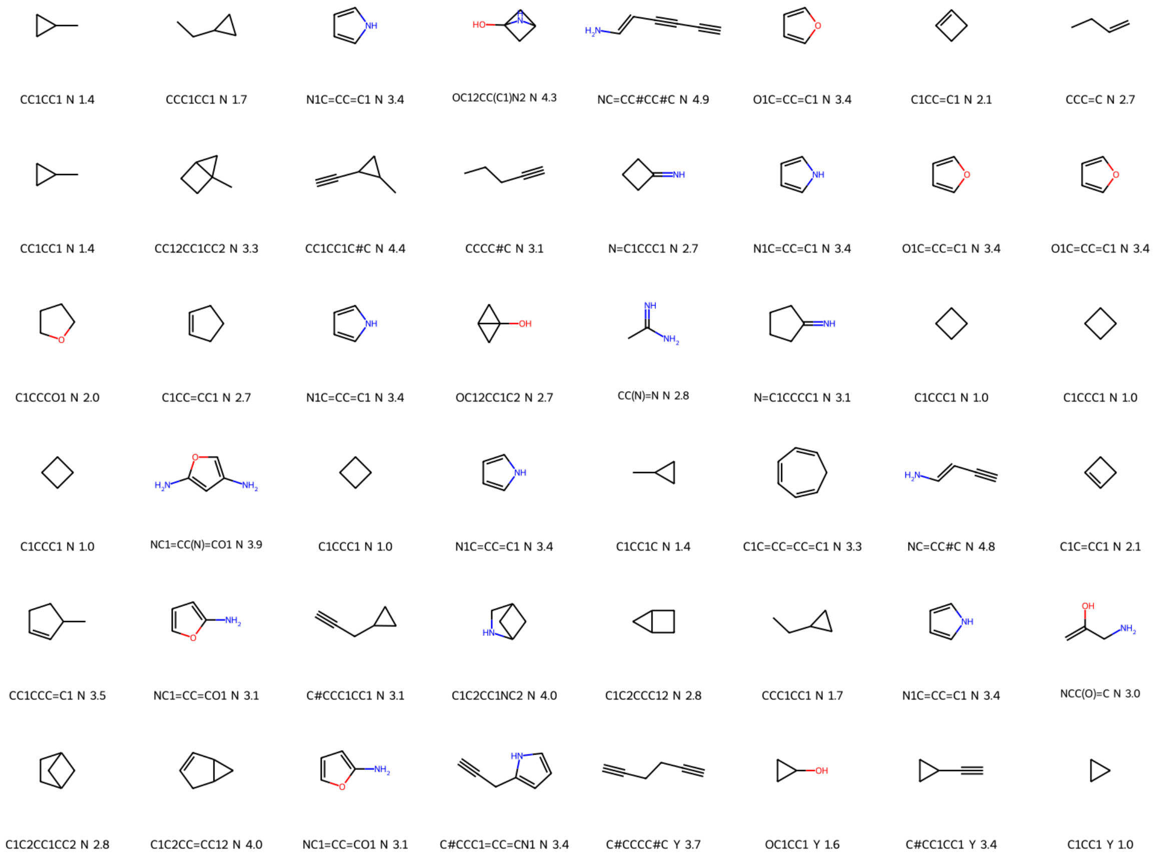

Supplementary Figure 182: Molecules obtained when trying to minimise the overlapping between host (CB6) and guest electron densities, while maximising their electrostatic interactions. The volume of the cavity was reduced with a kernel of size 3. $ed\_factor = 1$

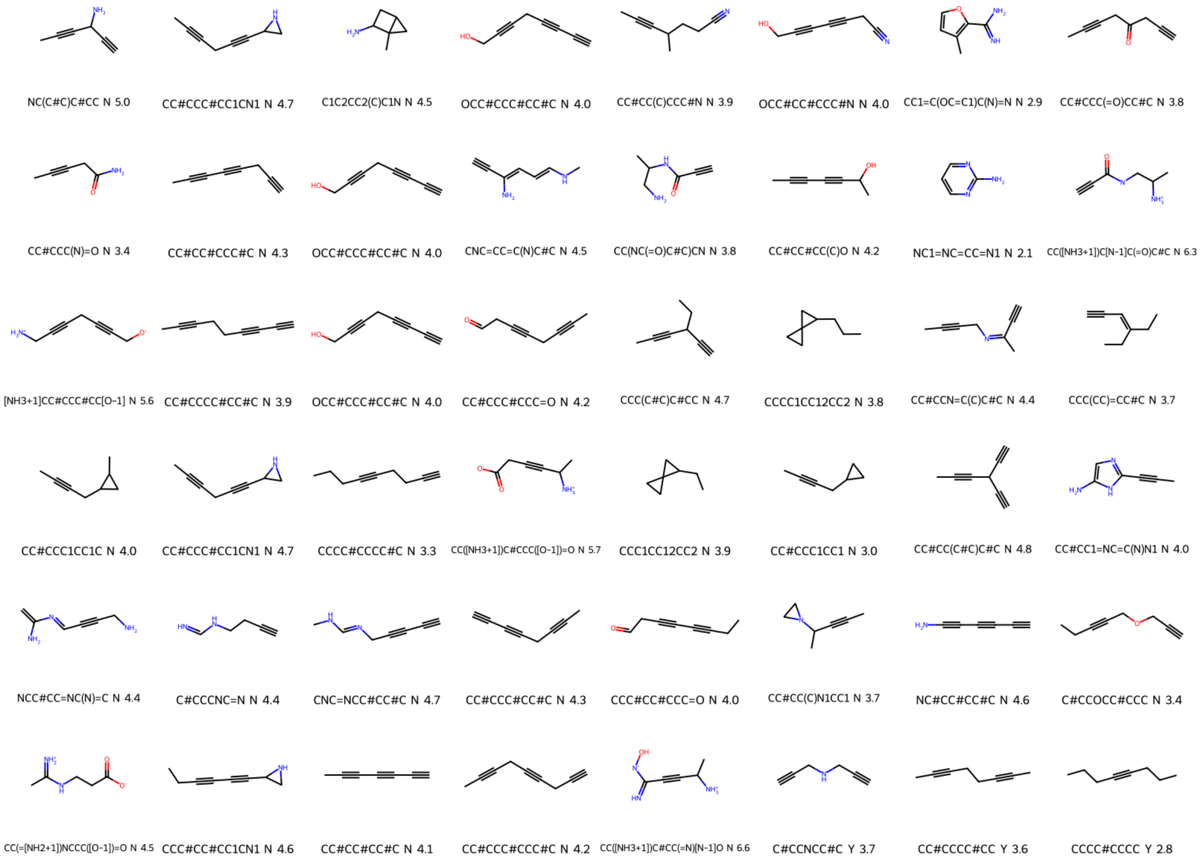

Supplementary Figure 183: Molecules obtained when trying to minimise the overlapping between host (CB6) and guest electron densities, while maximising their electrostatic interactions. The volume of the cavity was reduced with a kernel of size 4. $ed\_factor = 0$

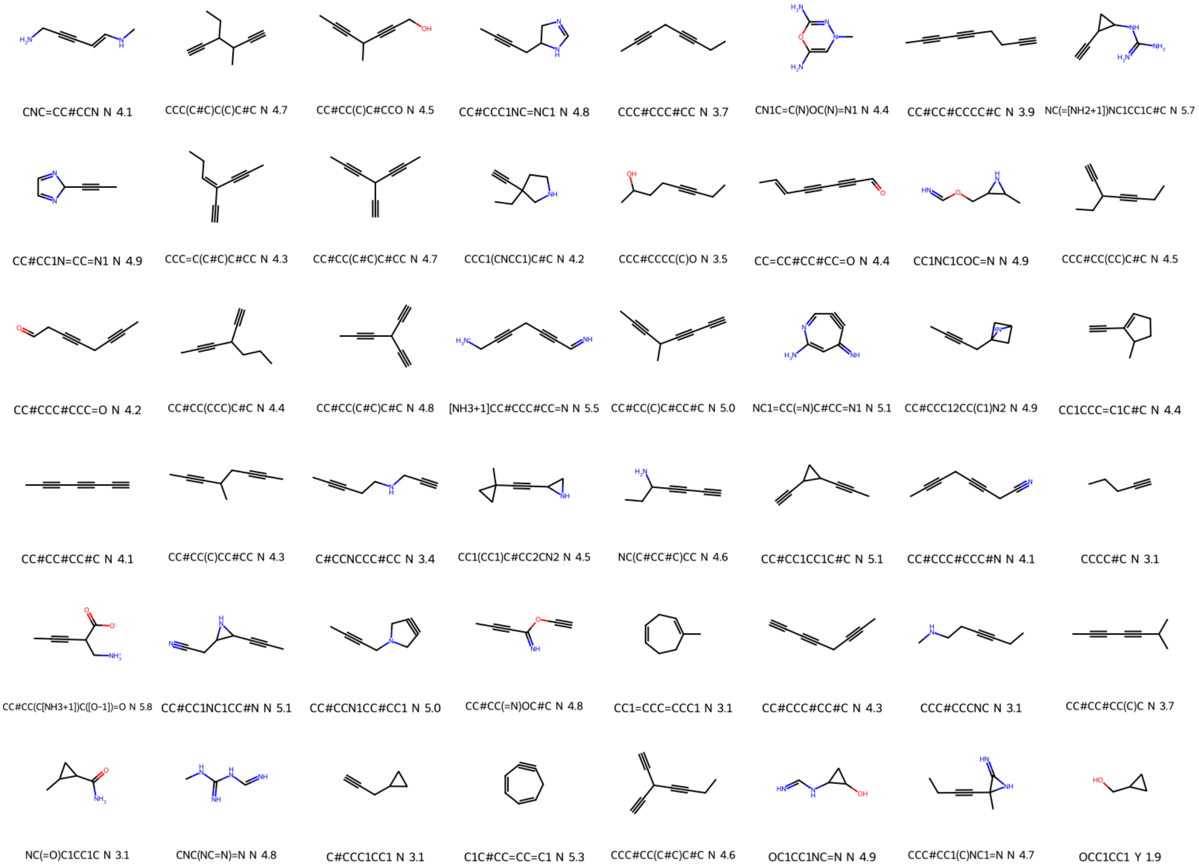

Supplementary Figure 184: Molecules obtained when trying to minimise the overlapping between host (CB6) and guest electron densities, while maximising their electrostatic interactions. The volume of the cavity was reduced with a kernel of size 4. $ed\_factor = 0.05$

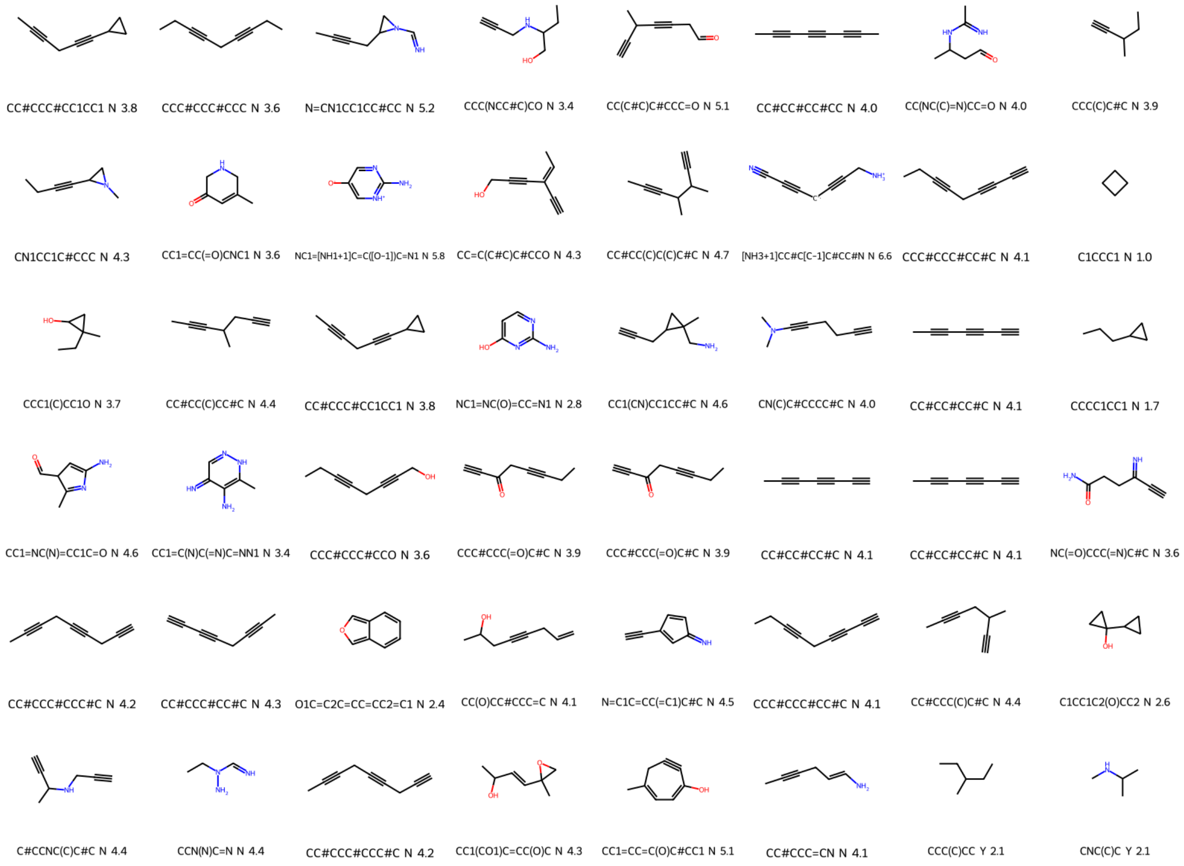

Supplementary Figure 185: Molecules obtained when trying to minimise the overlapping between host (CB6) and guest electron densities, while maximising their electrostatic interactions. The volume of the cavity was reduced with a kernel of size 4. $ed\_factor = 0.1$

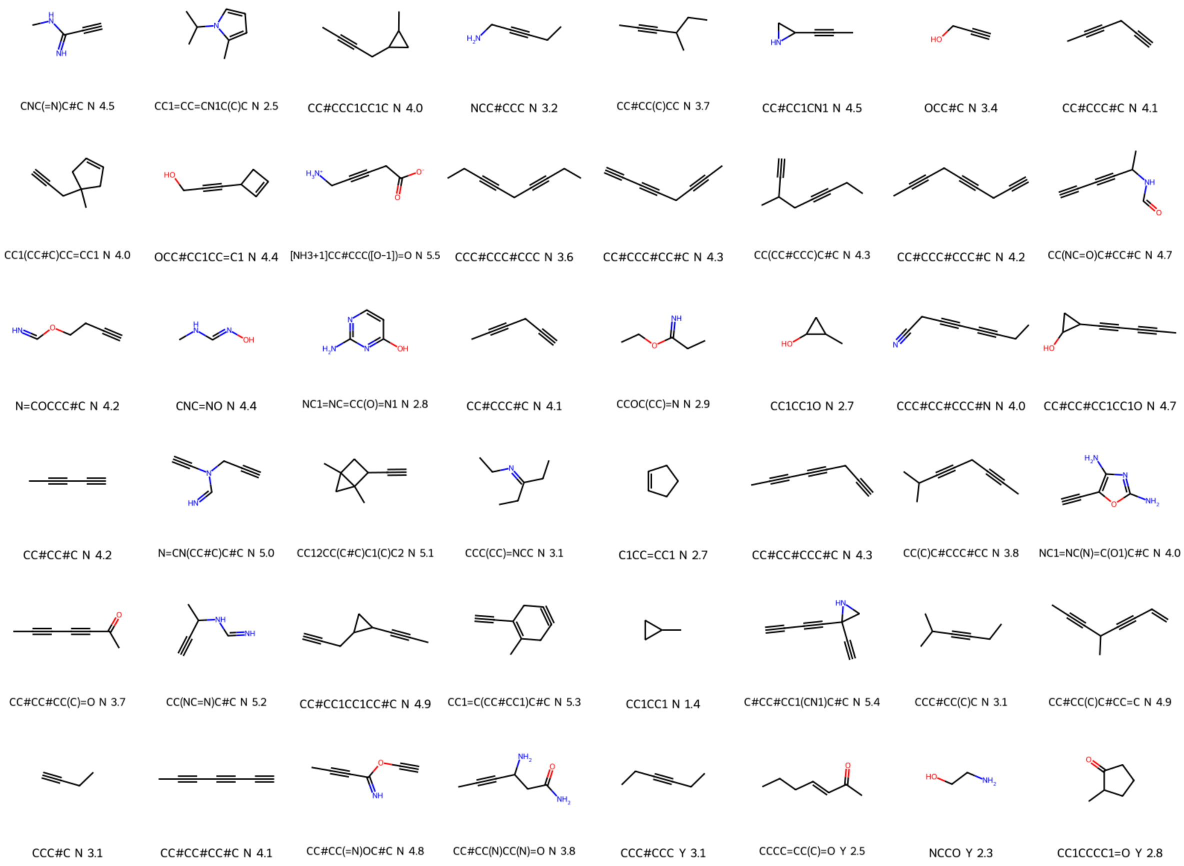

Supplementary Figure 186: Molecules obtained when trying to minimise the overlapping between host (CB6) and guest electron densities, while maximising their electrostatic interactions. The volume of the cavity was reduced with a kernel of size 4. $ed\_factor = 0.2$

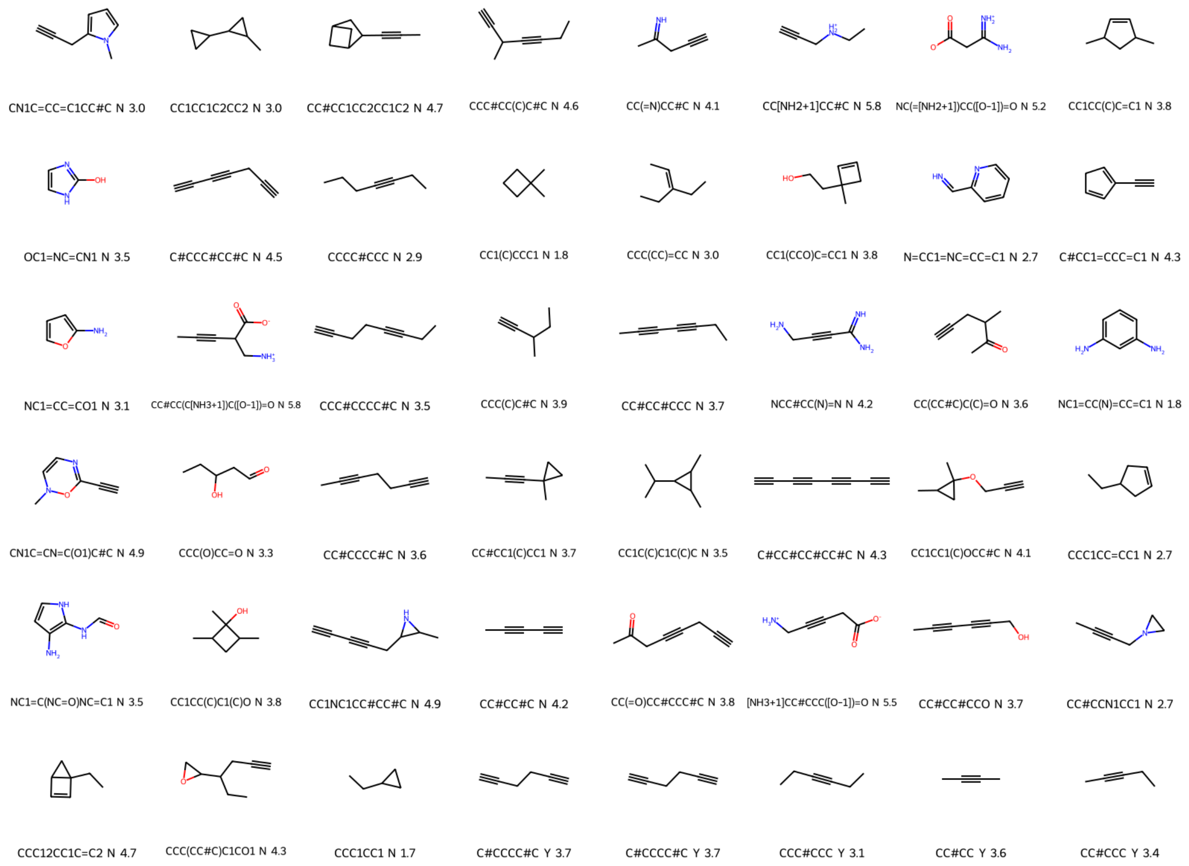

Supplementary Figure 187: Molecules obtained when trying to minimise the overlapping between host (CB6) and guest electron densities, while maximising their electrostatic interactions. The volume of the cavity was reduced with a kernel of size 4. $ed\_factor = 0.3$

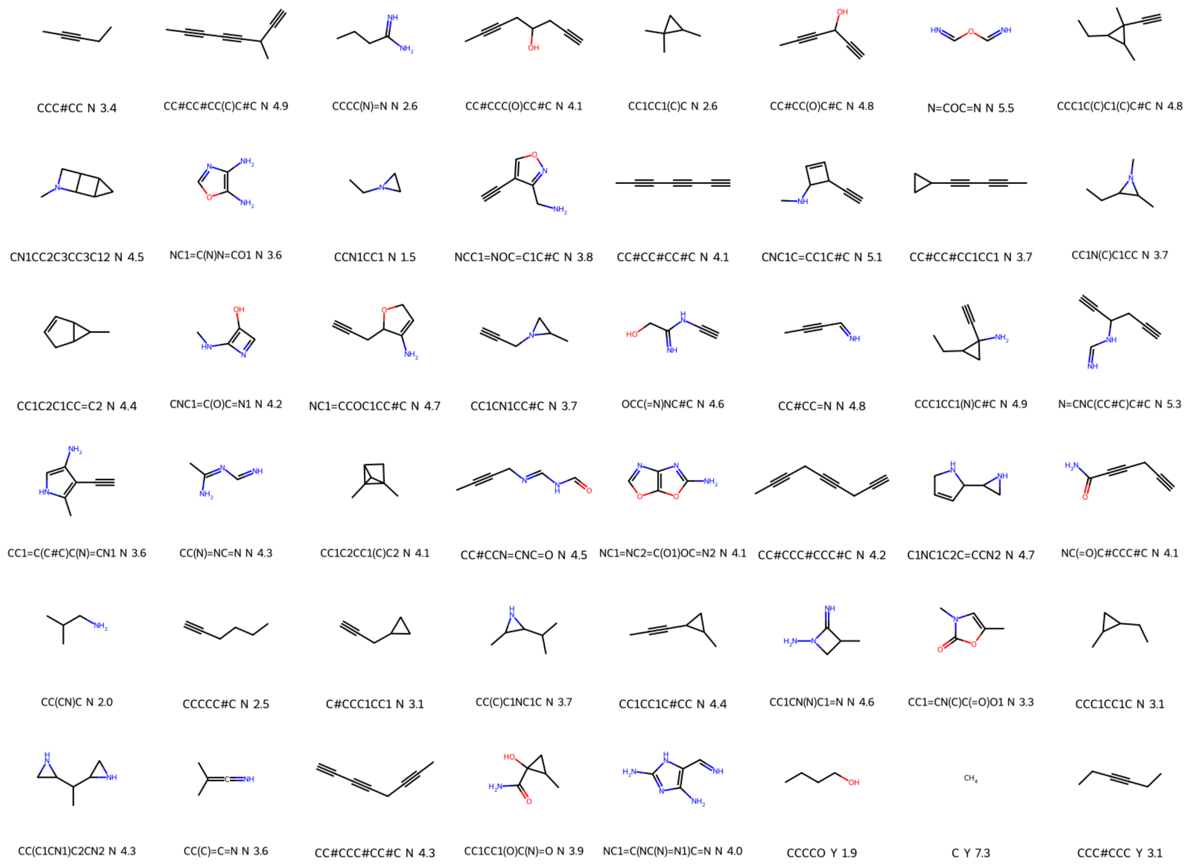

Supplementary Figure 188: Molecules obtained when trying to minimise the overlapping between host (CB6) and guest electron densities, while maximising their electrostatic interactions. The volume of the cavity was reduced with a kernel of size 4. $ed\_factor = 0.4$

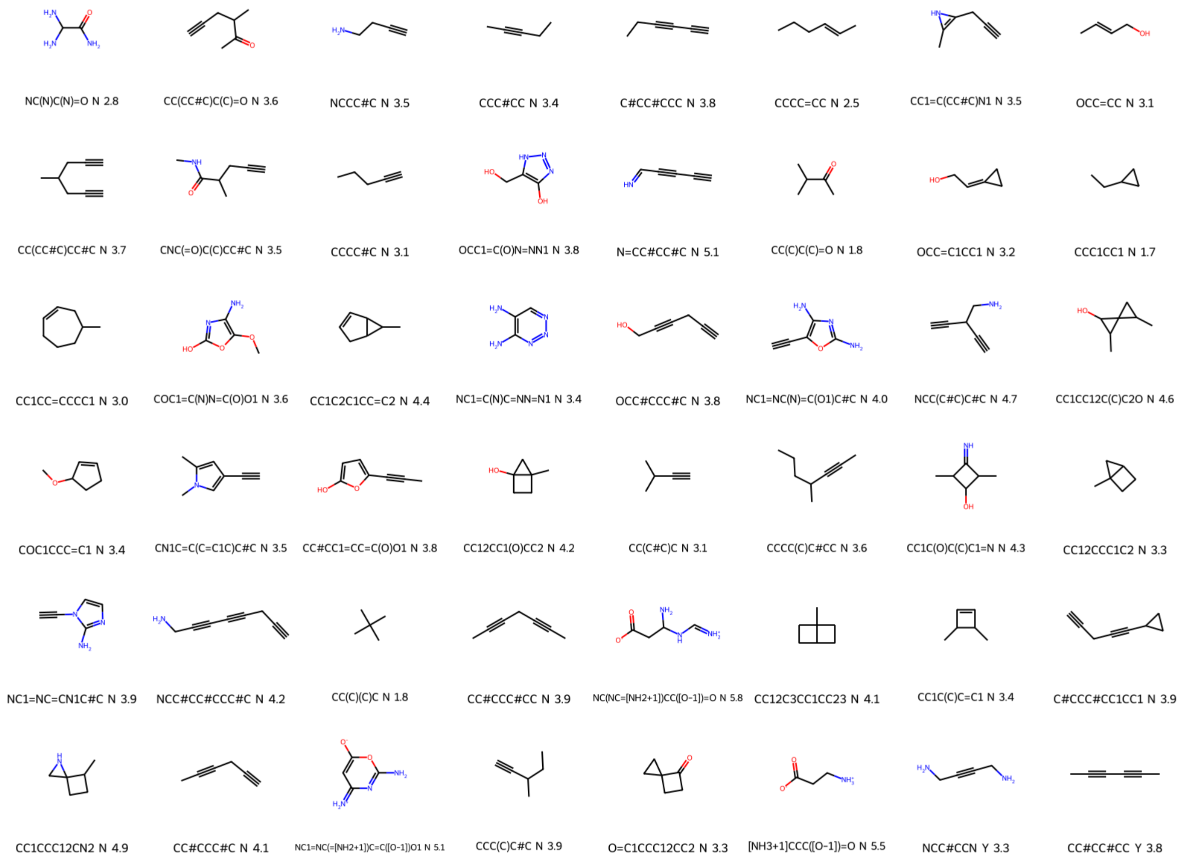

Supplementary Figure 189: Molecules obtained when trying to minimise the overlapping between host (CB6) and guest electron densities, while maximising their electrostatic interactions. The volume of the cavity was reduced with a kernel of size 4. $ed\_factor = 0.5$

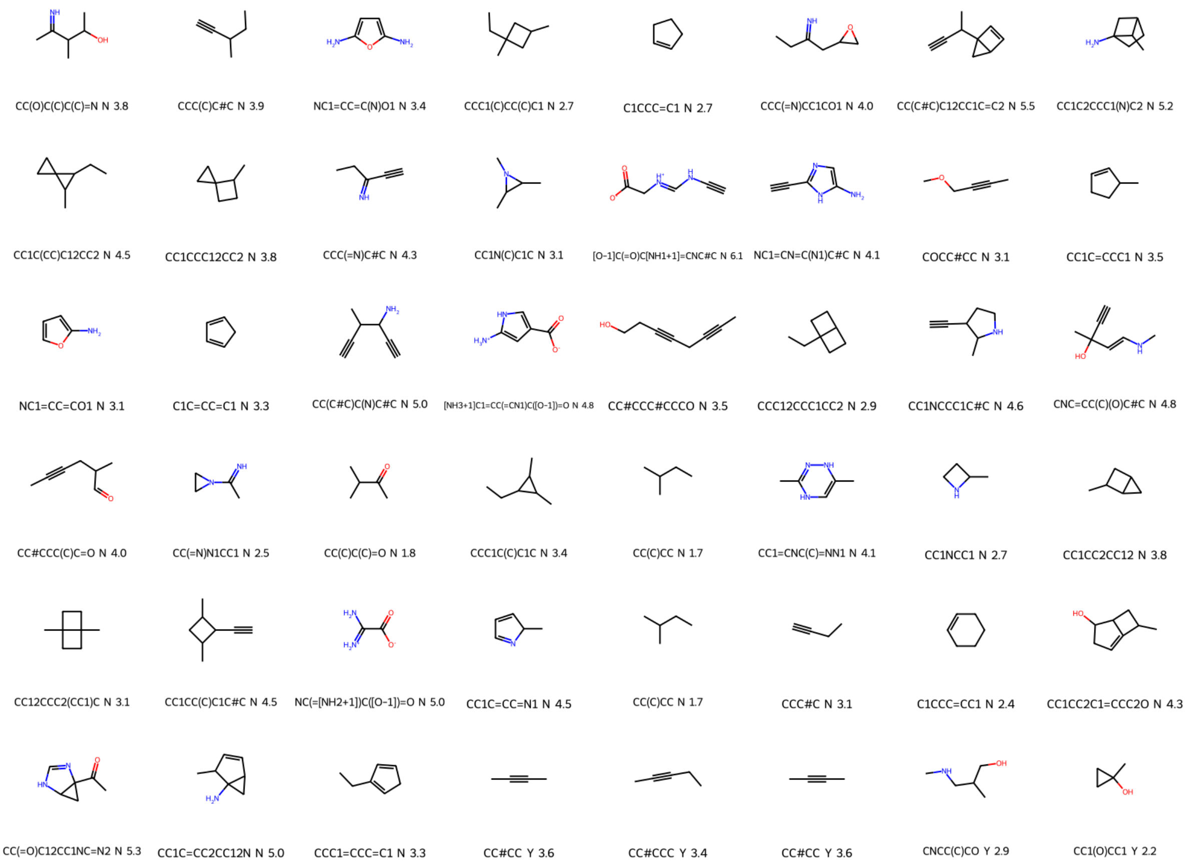

Supplementary Figure 190: Molecules obtained when trying to minimise the overlapping between host (CB6) and guest electron densities, while maximising their electrostatic interactions. The volume of the cavity was reduced with a kernel of size 4. $ed\_factor = 0.6$

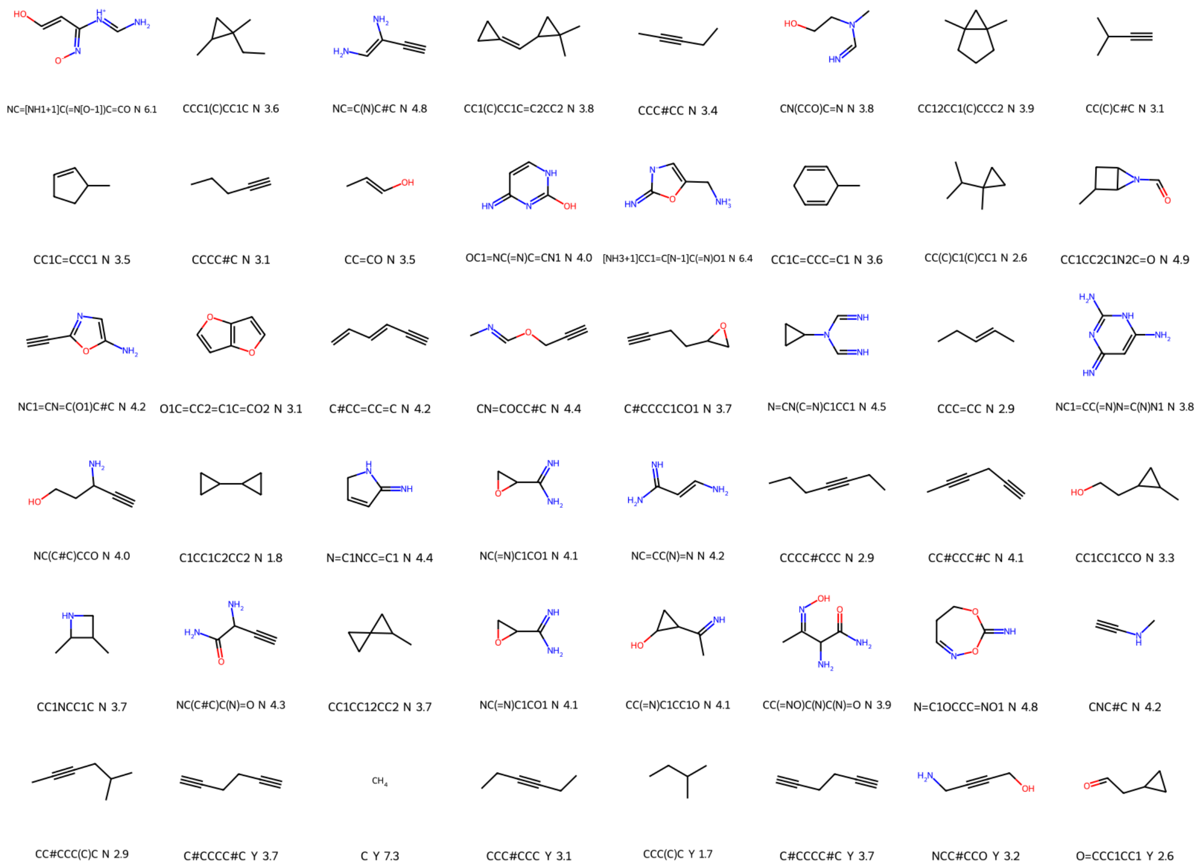

Supplementary Figure 191: Molecules obtained when trying to minimise the overlapping between host (CB6) and guest electron densities, while maximising their electrostatic interactions. The volume of the cavity was reduced with a kernel of size 4. $ed\_factor = 0.7$

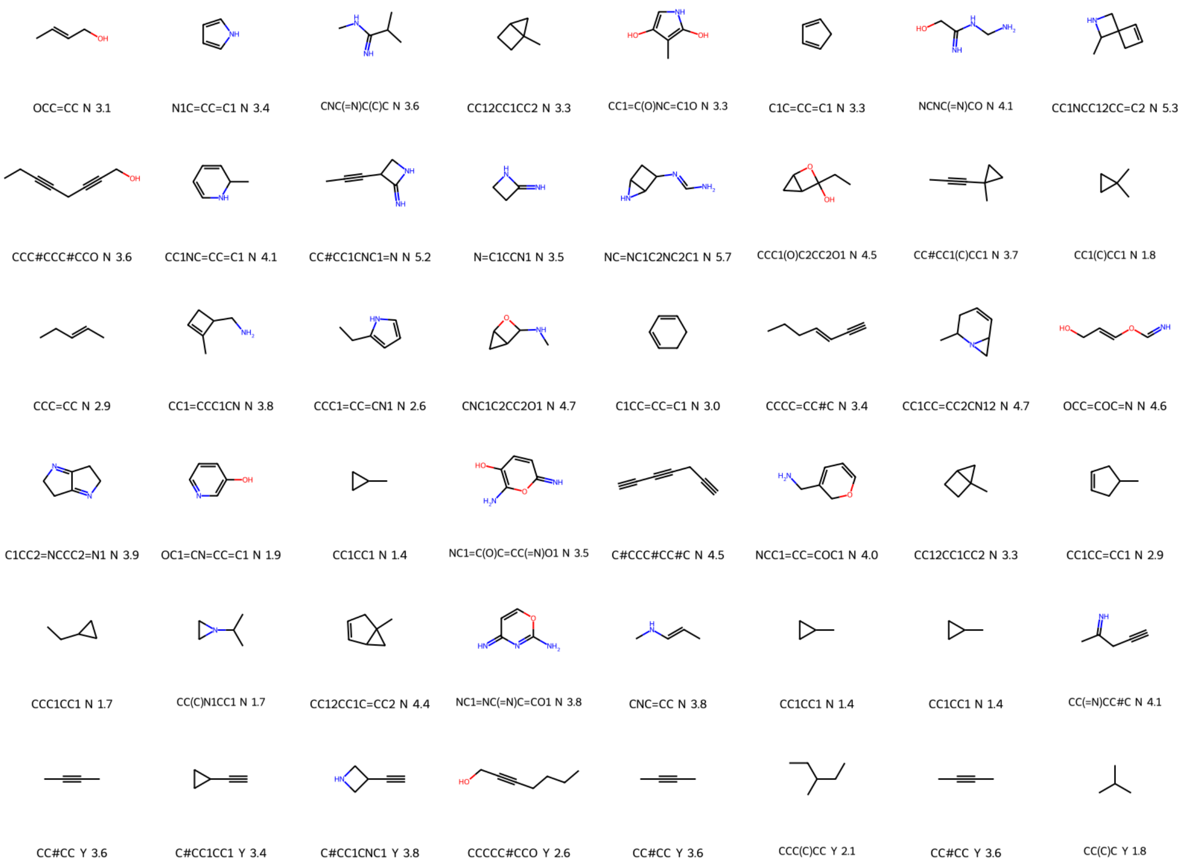

Supplementary Figure 192: Molecules obtained when trying to minimise the overlapping between host (CB6) and guest electron densities, while maximising their electrostatic interactions. The volume of the cavity was reduced with a kernel of size 4. $ed\_factor = 0.8$

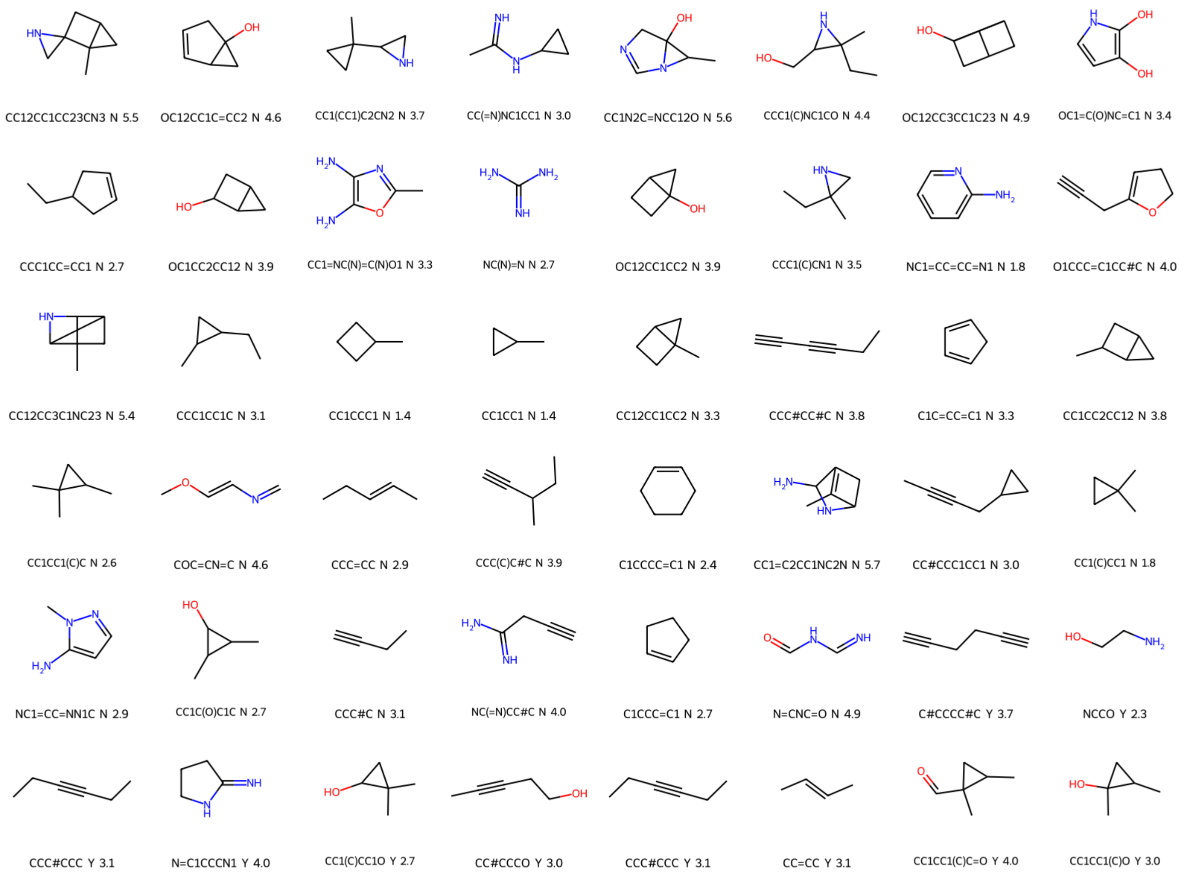

Supplementary Figure 193: Molecules obtained when trying to minimise the overlapping between host (CB6) and guest electron densities, while maximising their electrostatic interactions. The volume of the cavity was reduced with a kernel of size 4. $ed\_factor = 0.9$

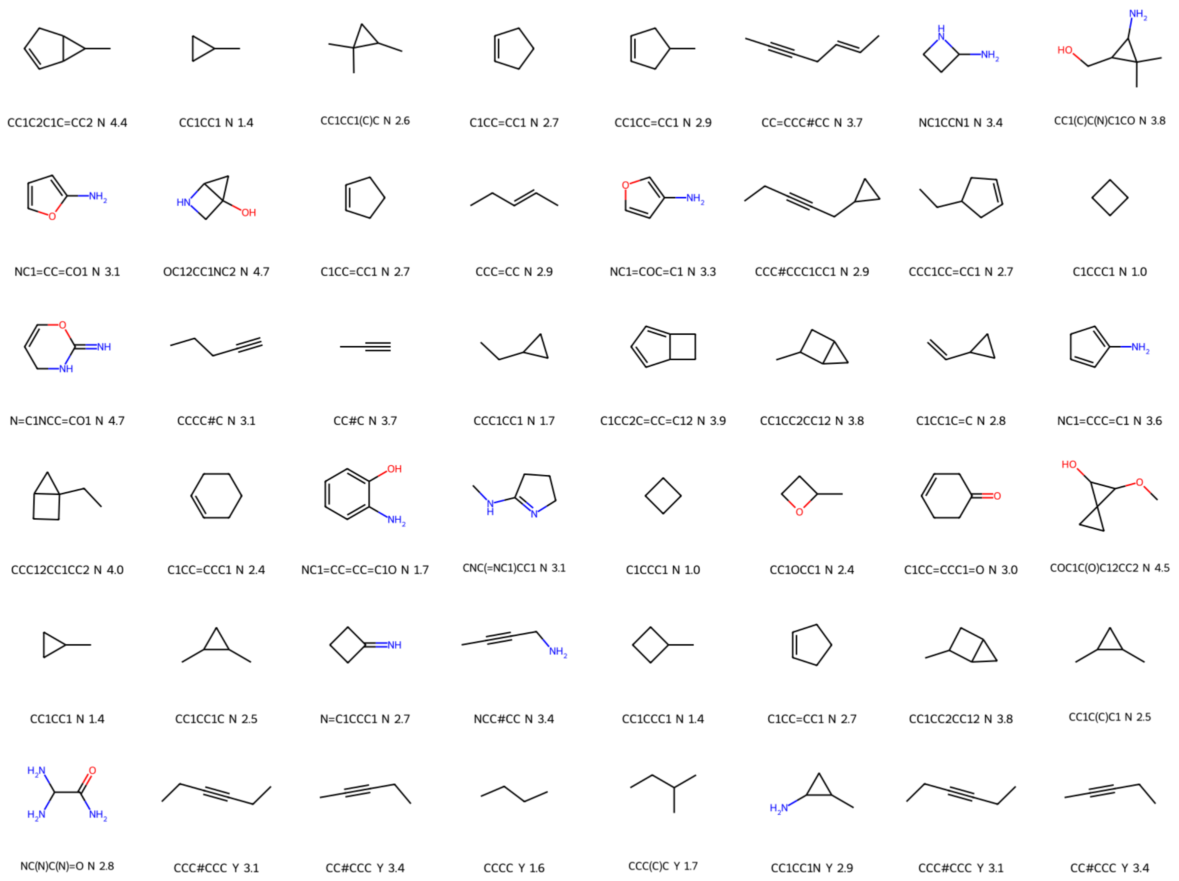

Supplementary Figure 194: Molecules obtained when trying to minimise the overlapping between host (CB6) and guest electron densities, while maximising their electrostatic interactions. The volume of the cavity was reduced with a kernel of size 4. $ed\_factor = 0.95$

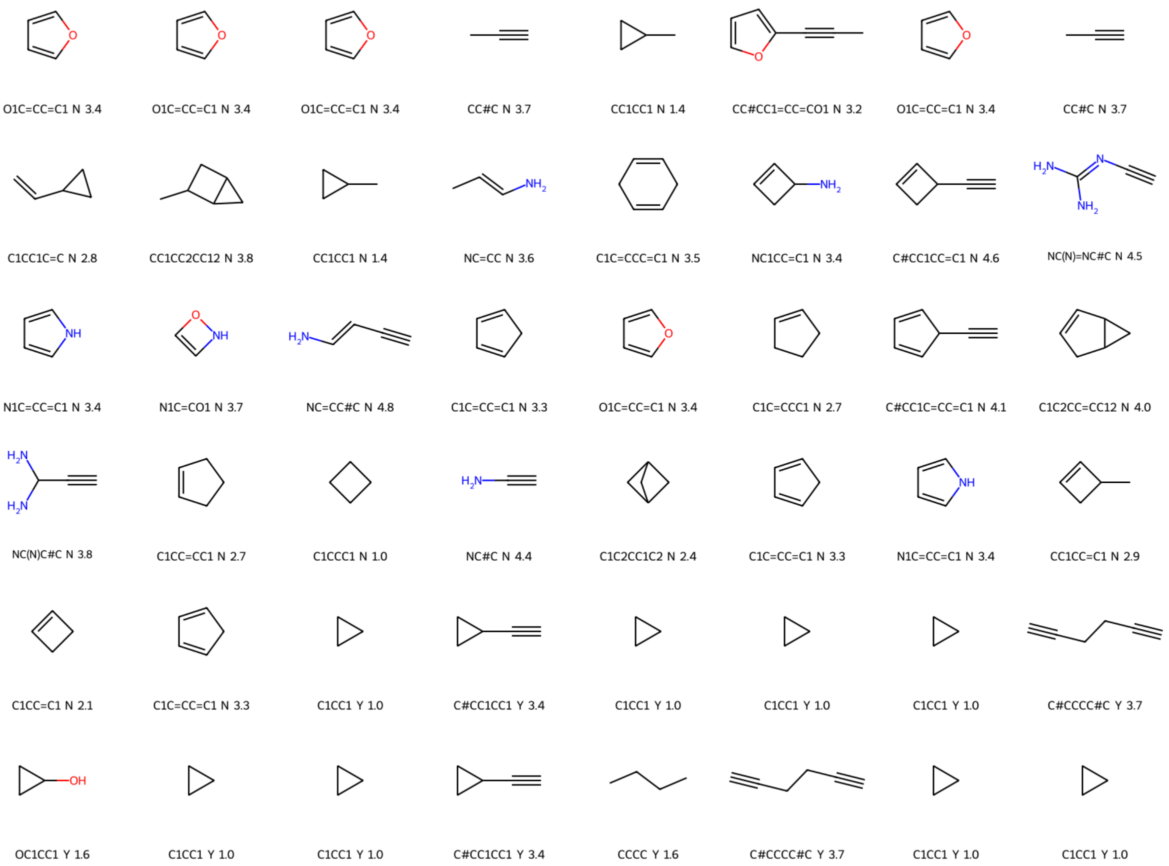

Supplementary Figure 195: Molecules obtained when trying to minimise the overlapping between host (CB6) and guest electron densities, while maximising their electrostatic interactions. The volume of the cavity was reduced with a kernel of size 4. $ed\_factor = 1$

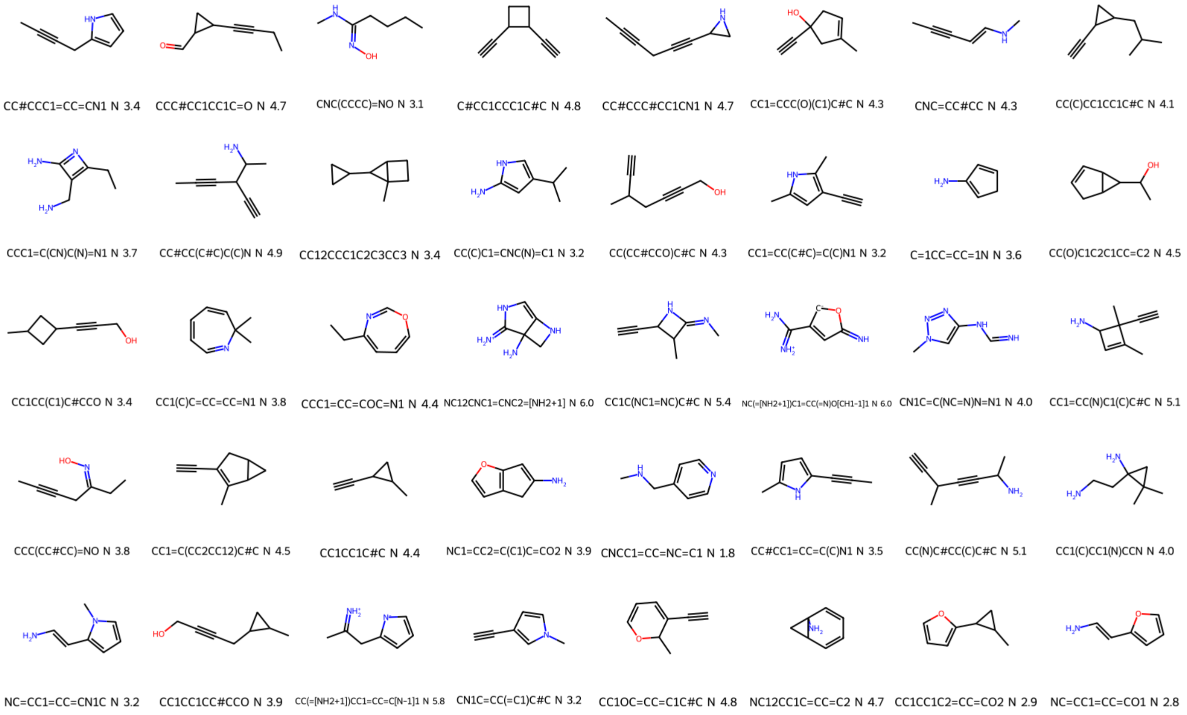

Supplementary Figure 196: Molecules obtained when trying to minimise the overlapping between host (cage) and guest electron densities, while maximising their electrostatic interactions. $ed\_factor = 0$

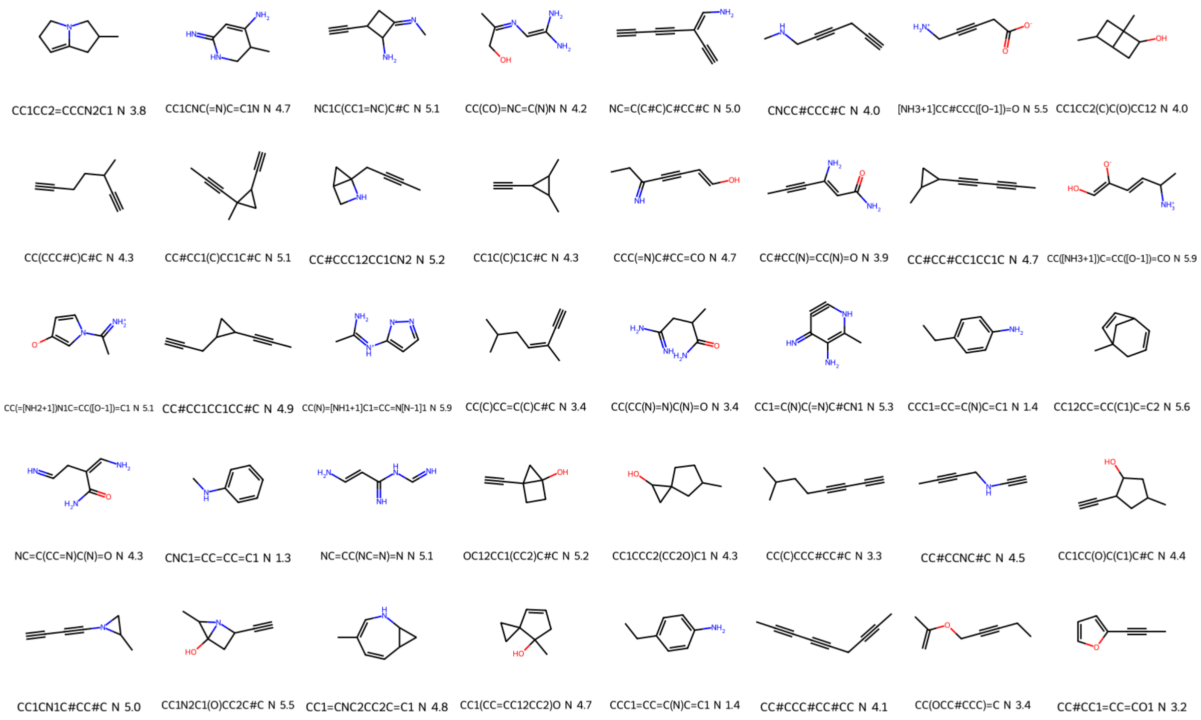

Supplementary Figure 197: Molecules obtained when trying to minimise the overlapping between host (cage) and guest electron densities, while maximising their electrostatic interactions. $ed\_factor = 0.0001$

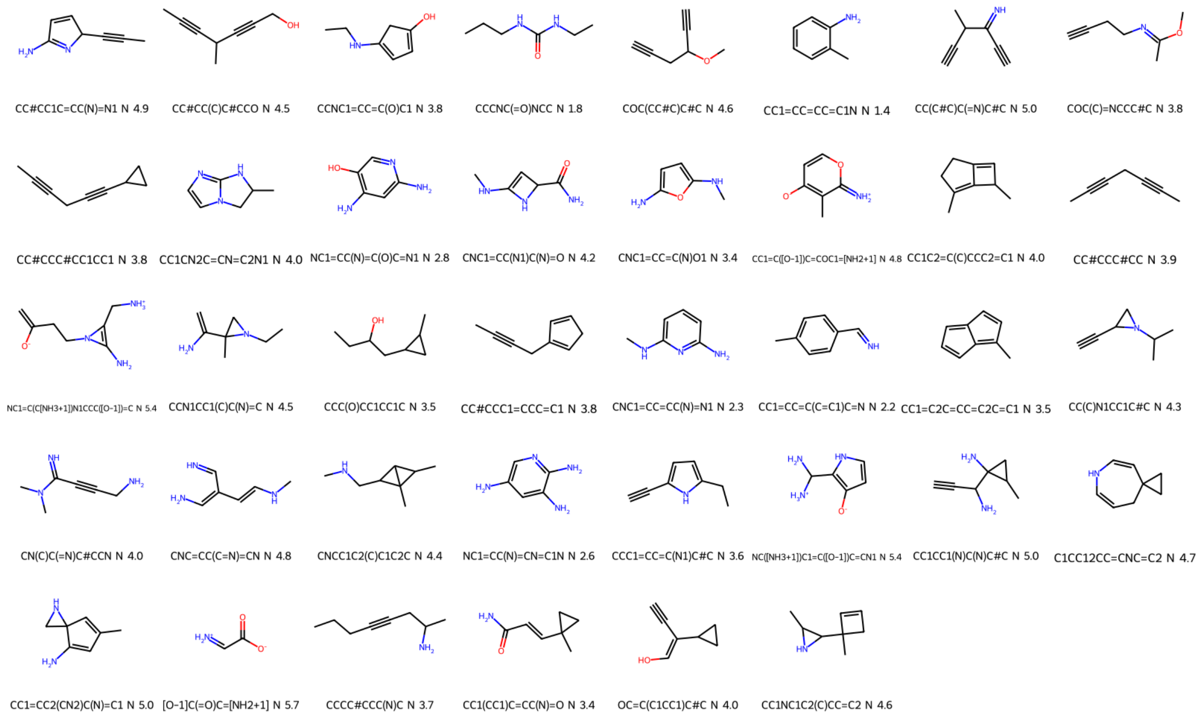

Supplementary Figure 198: Molecules obtained when trying to minimise the overlapping between host (cage) and guest electron densities, while maximising their electrostatic interactions. $ed\_factor = 0.001$

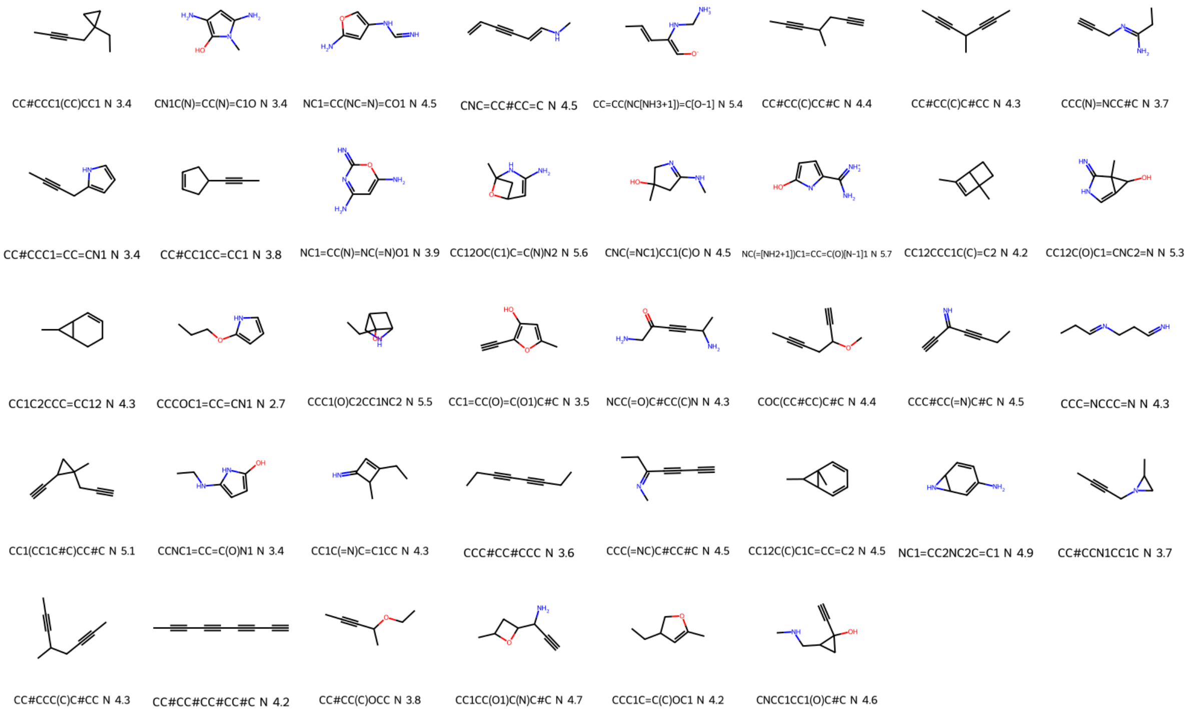

Supplementary Figure 199: Molecules obtained when trying to minimise the overlapping between host (cage) and guest electron densities, while maximising their electrostatic interactions. $ed\_factor = 0.01$

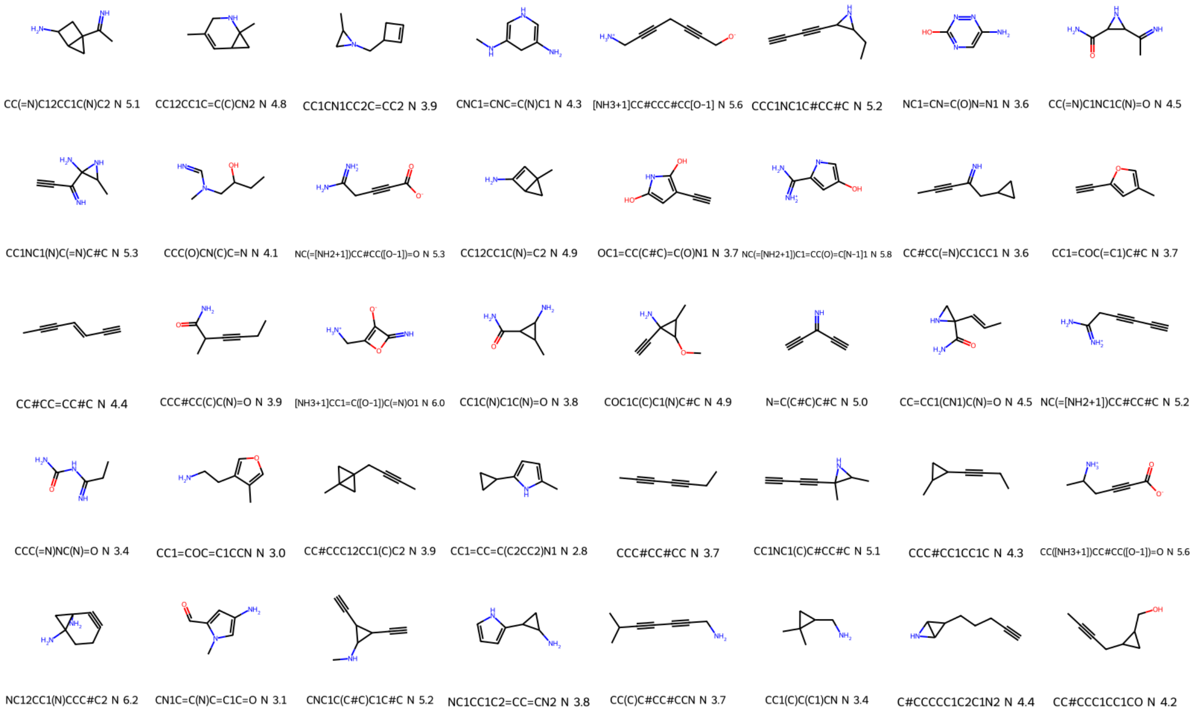

Supplementary Figure 200: Molecules obtained when trying to minimise the overlapping between host (cage) and guest electron densities, while maximising their electrostatic interactions. $ed\_factor = 0.05$

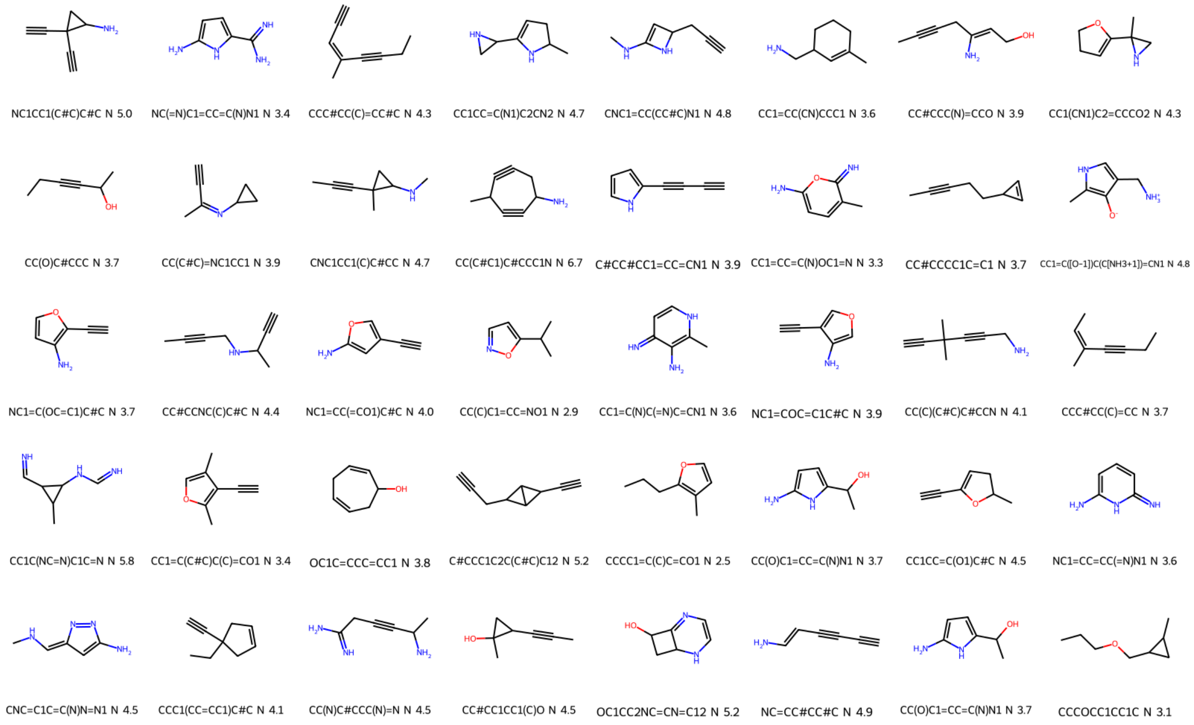

Supplementary Figure 201: Molecules obtained when trying to minimise the overlapping between host (cage) and guest electron densities, while maximising their electrostatic interactions. $ed\_factor = 0.1$

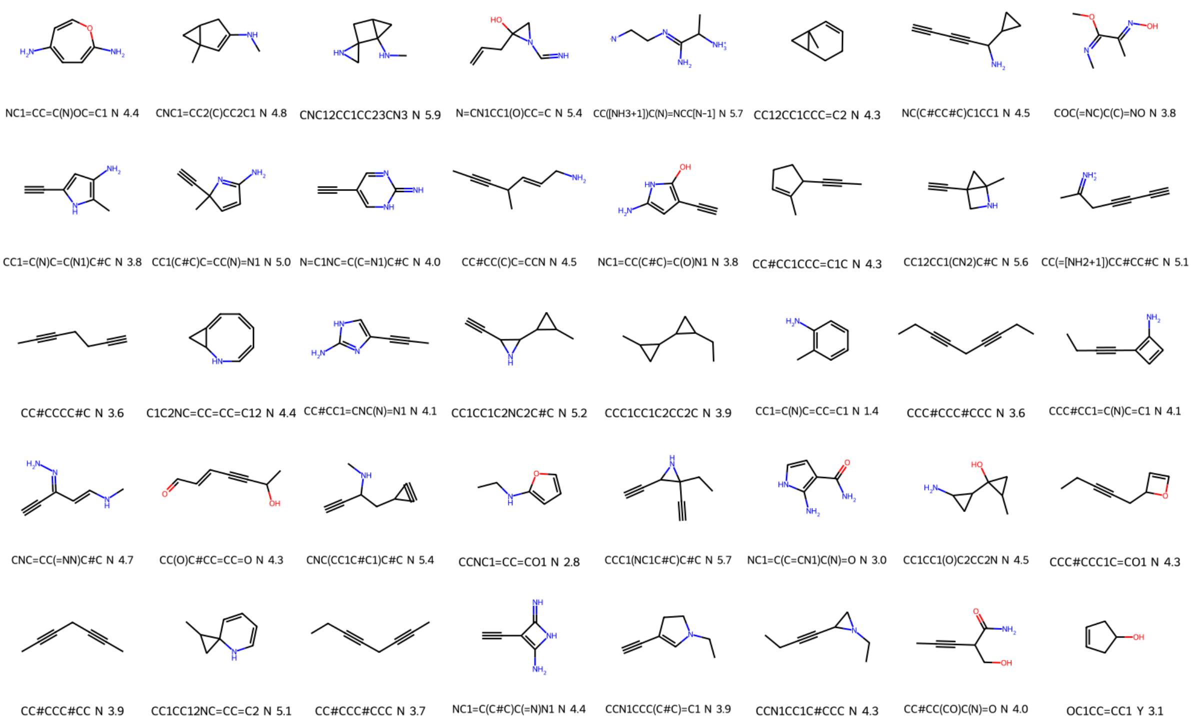

Supplementary Figure 202: Molecules obtained when trying to minimise the overlapping between host (cage) and guest electron densities, while maximising their electrostatic interactions. $ed\_factor = 0.2$

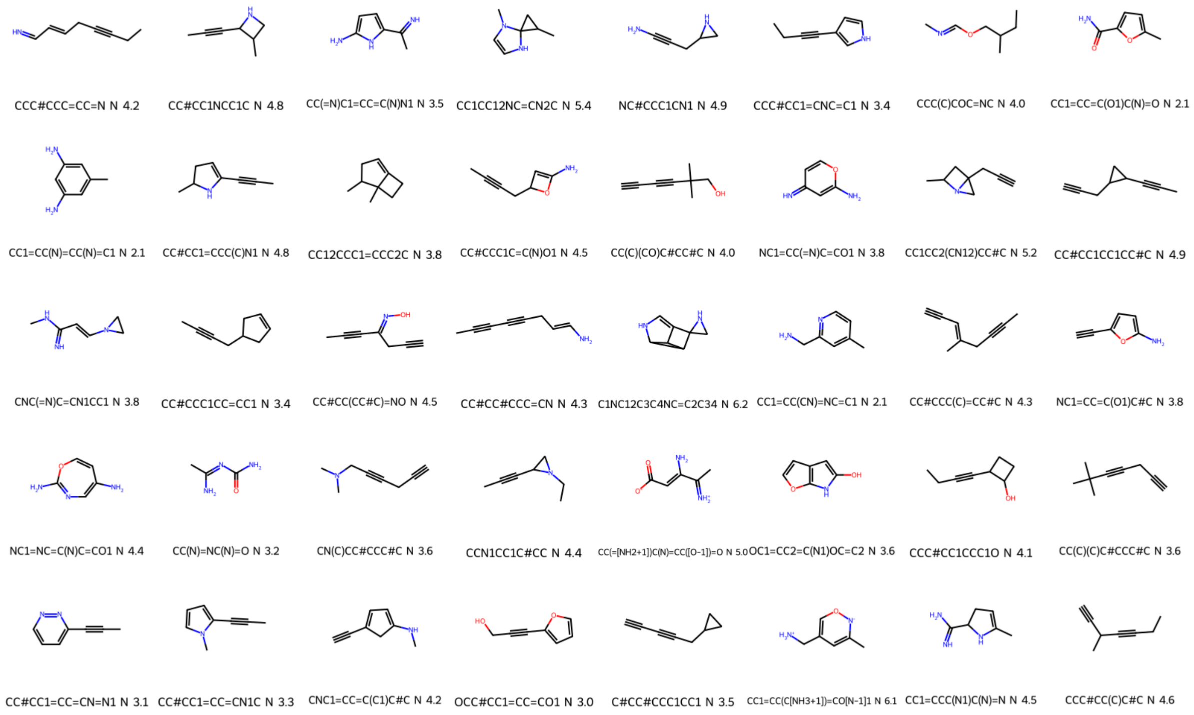

Supplementary Figure 203: Molecules obtained when trying to minimise the overlapping between host (cage) and guest electron densities, while maximising their electrostatic interactions. $ed\_factor = 0.3$

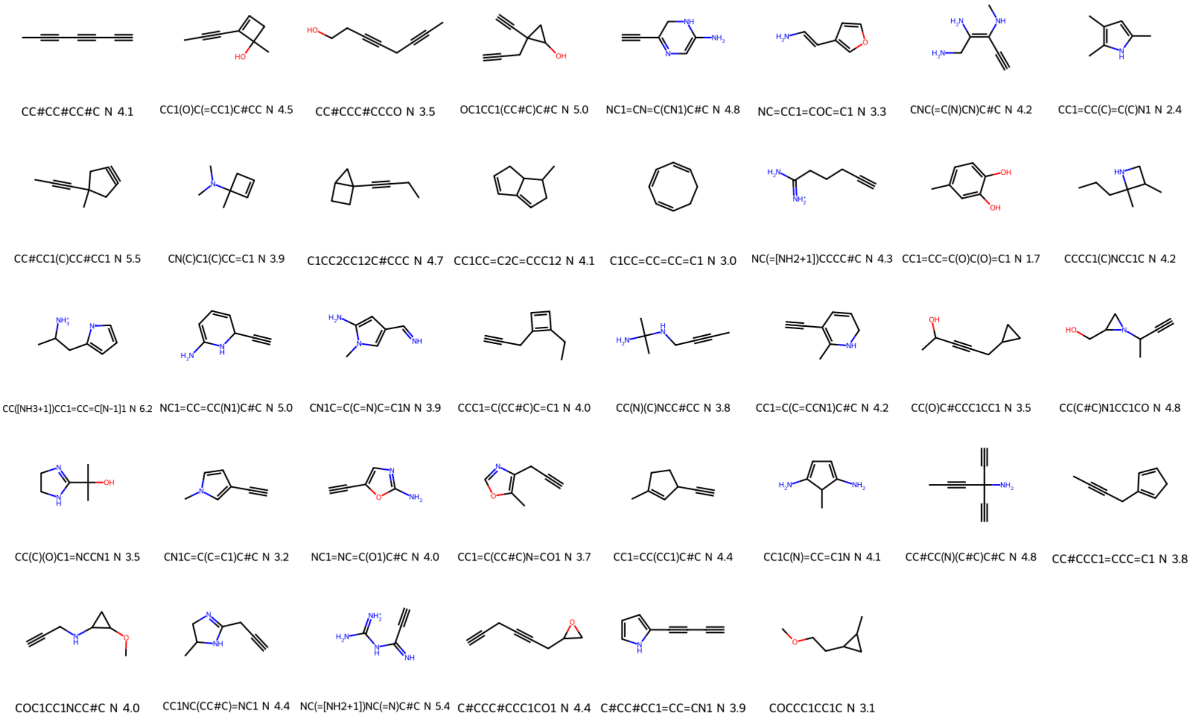

Supplementary Figure 204: Molecules obtained when trying to minimise the overlapping between host (cage) and guest electron densities, while maximising their electrostatic interactions. $ed\_factor = 0.4$

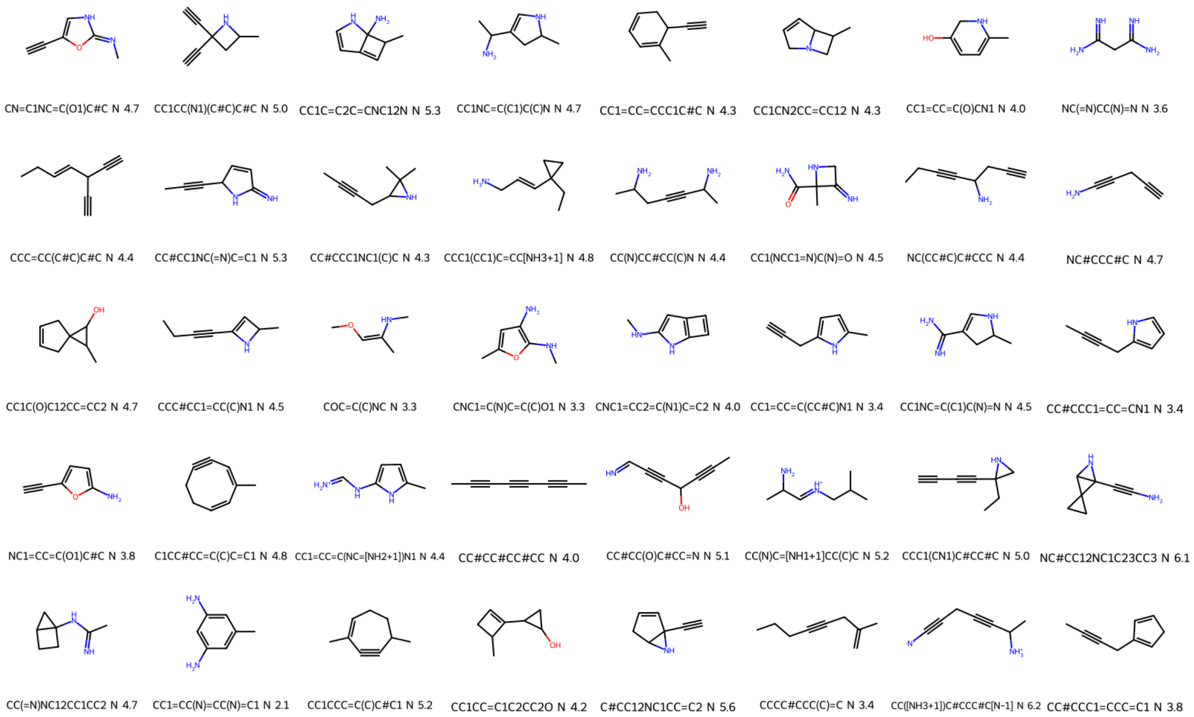

Supplementary Figure 205: Molecules obtained when trying to minimise the overlapping between host (cage) and guest electron densities, while maximising their electrostatic interactions. $ed\_factor = 0.5$

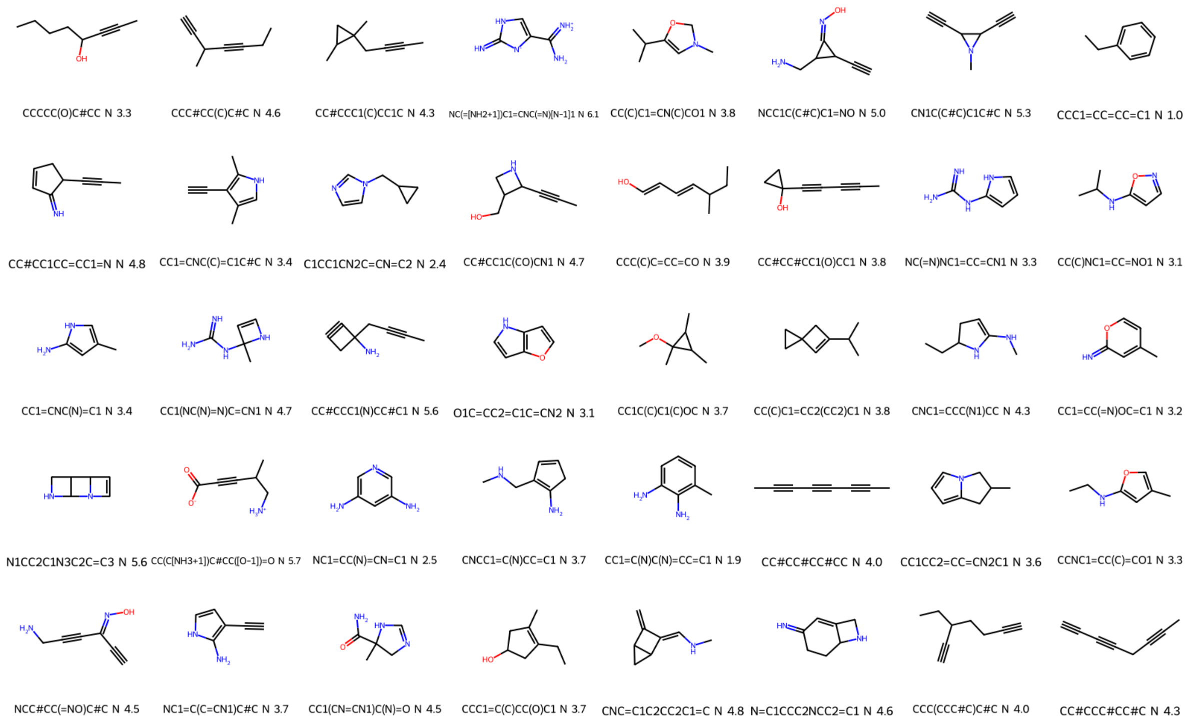

Supplementary Figure 206: Molecules obtained when trying to minimise the overlapping between host (cage) and guest electron densities, while maximising their electrostatic interactions. $ed\_factor = 0.6$

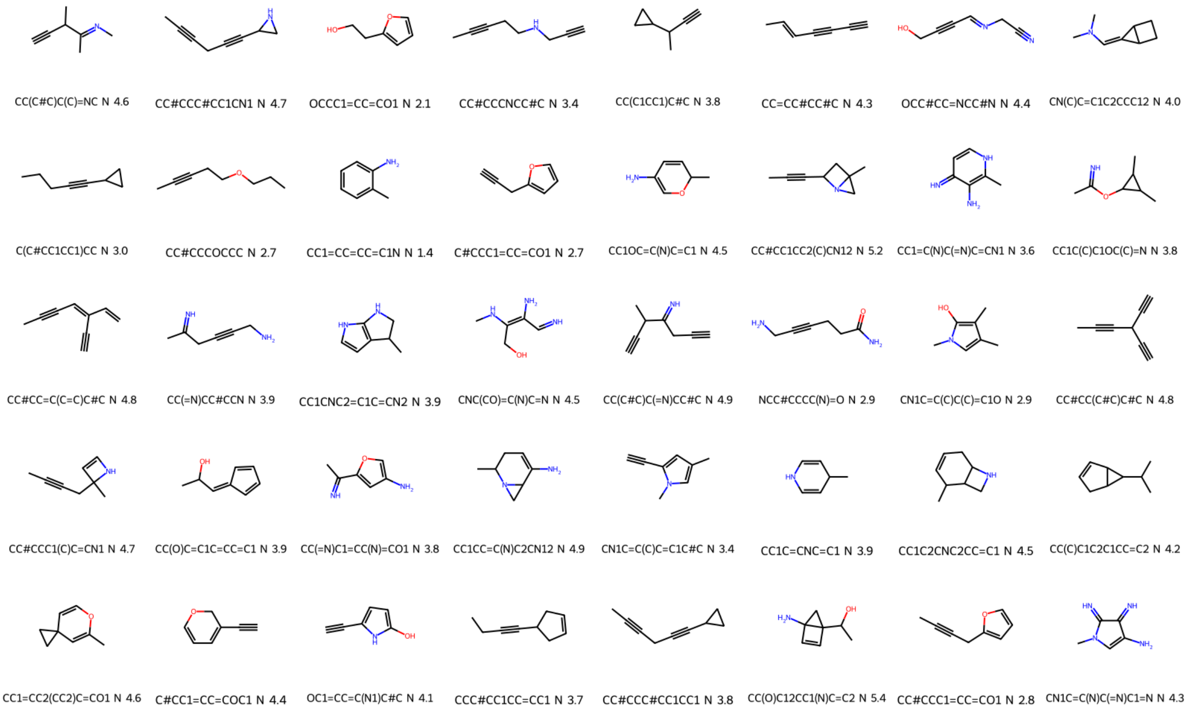

Supplementary Figure 207: Molecules obtained when trying to minimise the overlapping between host (cage) and guest electron densities, while maximising their electrostatic interactions. $ed\_factor = 0.7$

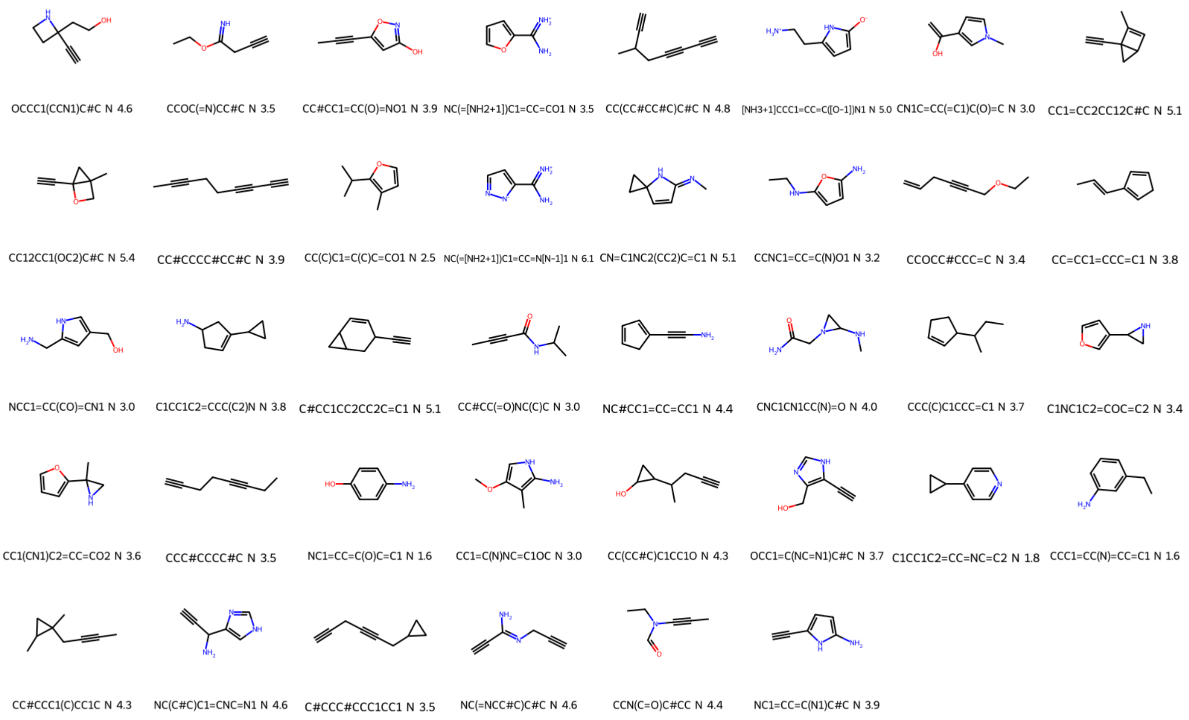

Supplementary Figure 208: Molecules obtained when trying to minimise the overlapping between host (cage) and guest electron densities, while maximising their electrostatic interactions. $ed\_factor = 0.8$

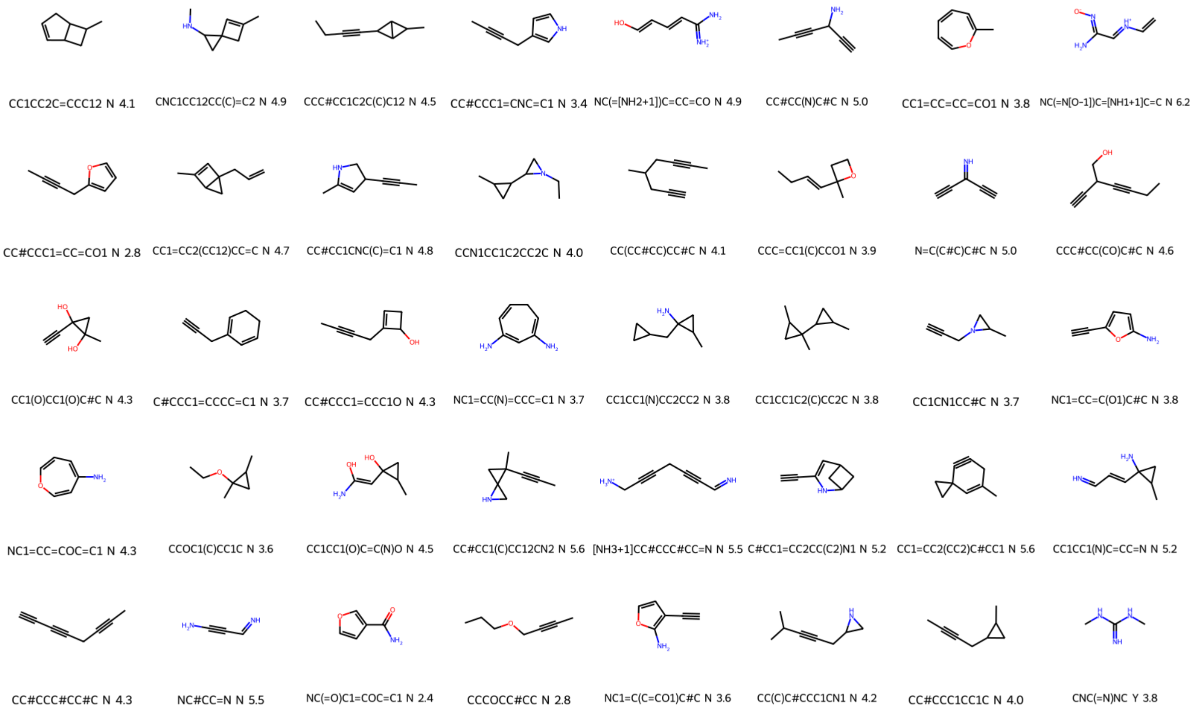

Supplementary Figure 209: Molecules obtained when trying to minimise the overlapping between host (cage) and guest electron densities, while maximising their electrostatic interactions. $ed\_factor = 0.9$

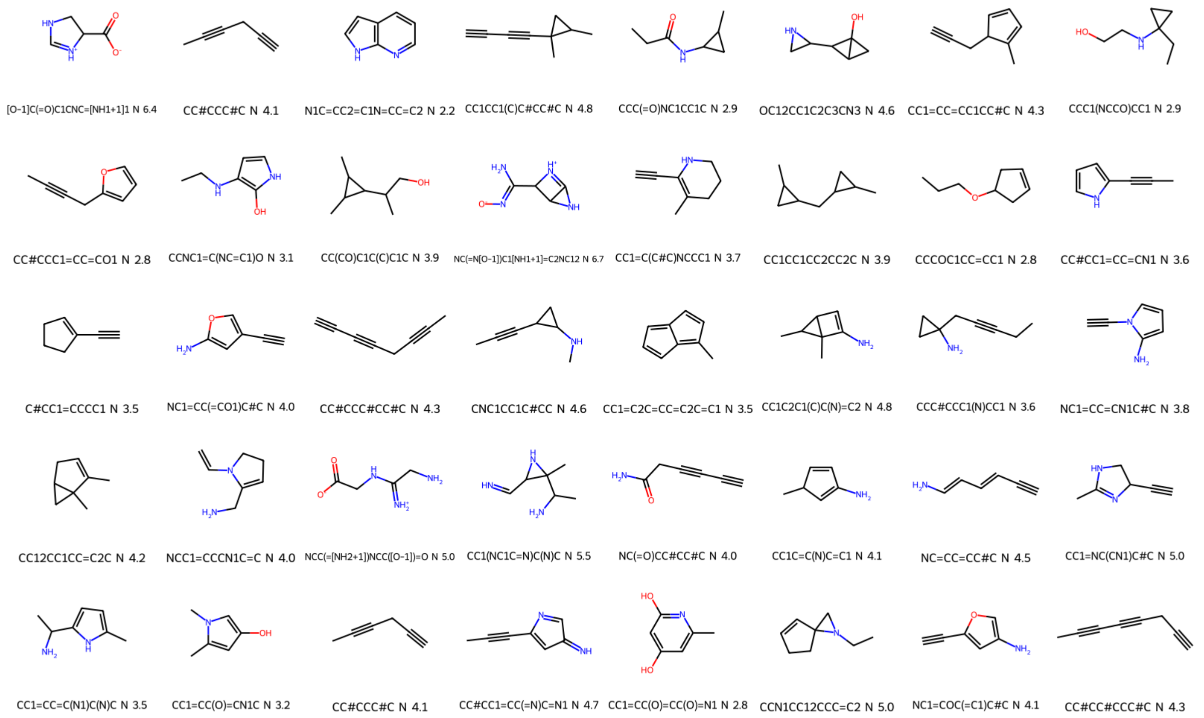

Supplementary Figure 210: Molecules obtained when trying to minimise the overlapping between host (cage) and guest electron densities, while maximising their electrostatic interactions. $ed\_factor = 0.95$

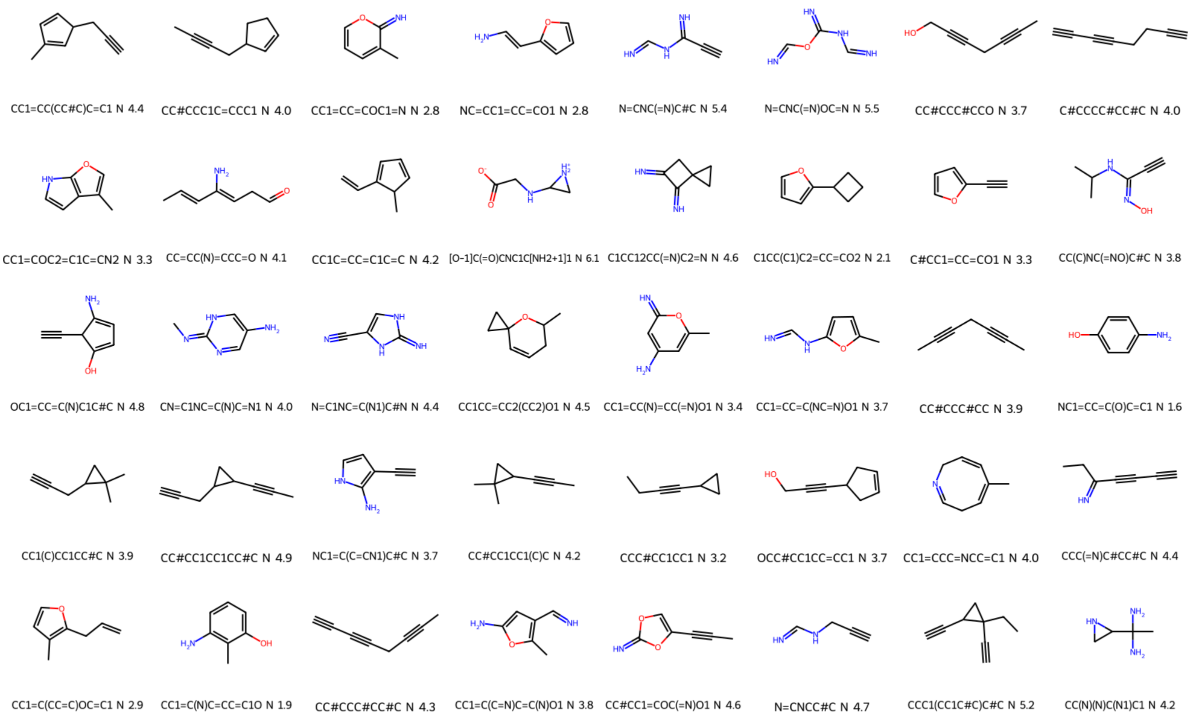

Supplementary Figure 211: Molecules obtained when trying to minimise the overlapping between host (cage) and guest electron densities, while maximising their electrostatic interactions. $ed\_factor = 0.99$

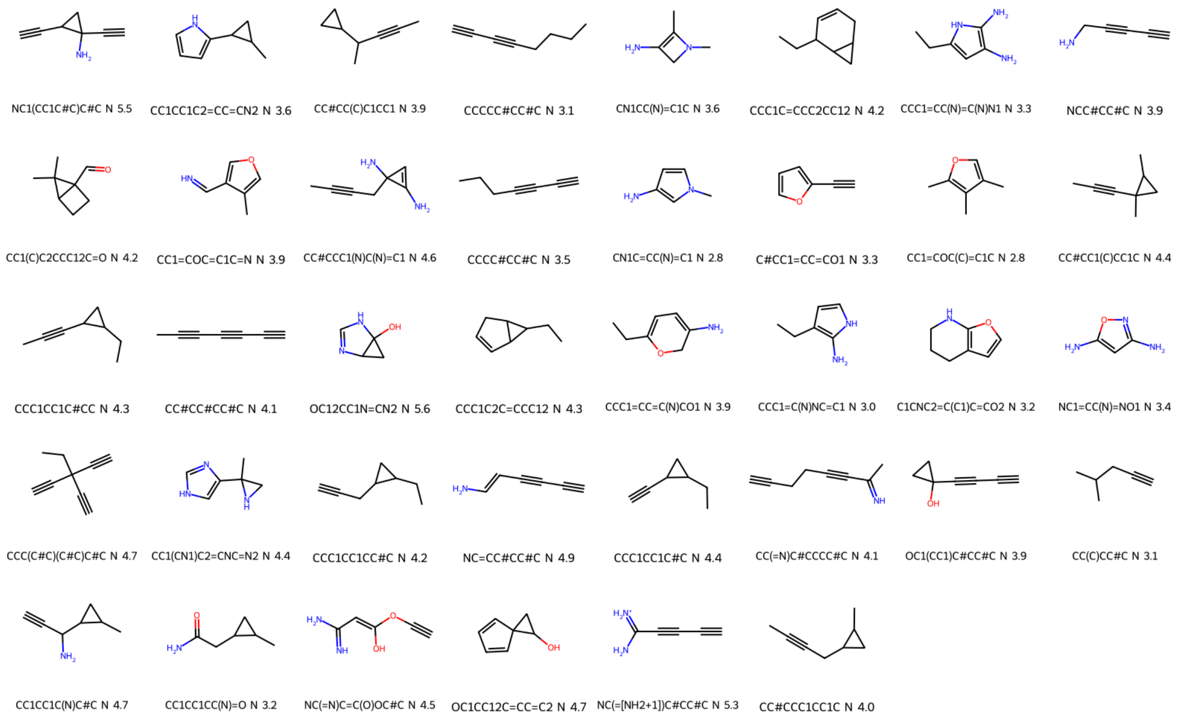

Supplementary Figure 212: Molecules obtained when trying to minimise the overlapping between host (cage) and guest electron densities, while maximising their electrostatic interactions. $ed\_factor = 0.999$

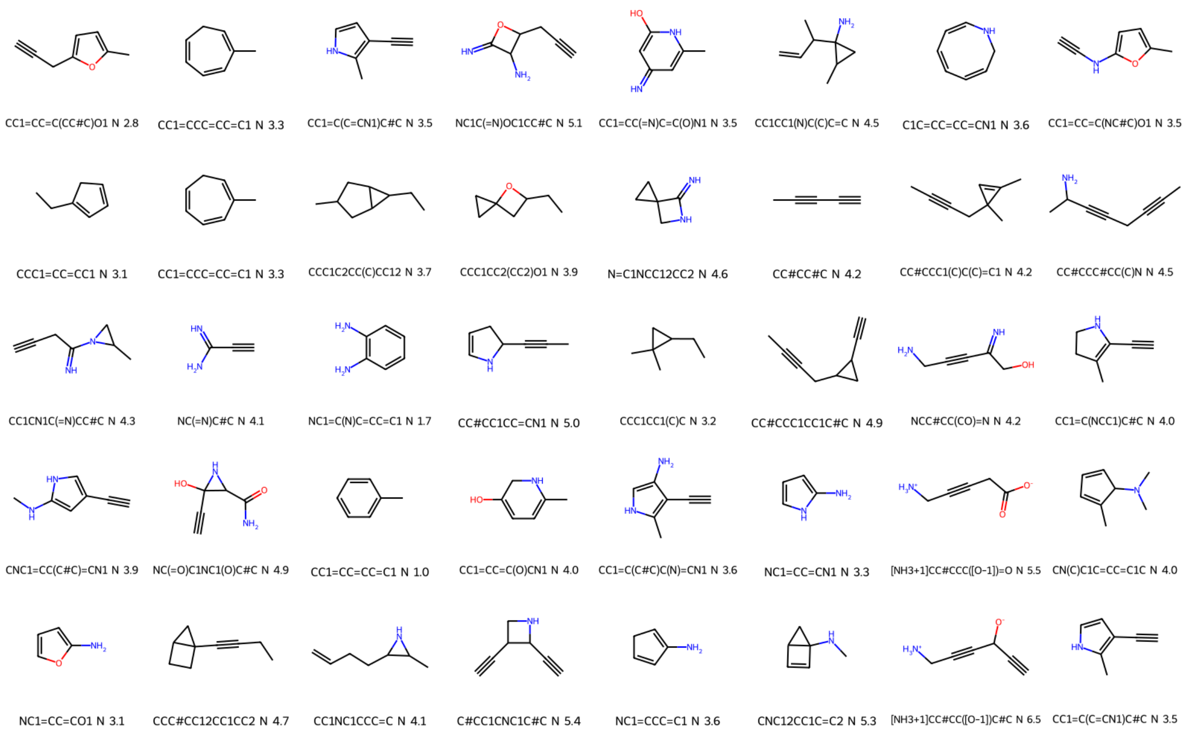

Supplementary Figure 213: Molecules obtained when trying to minimise the overlapping between host (cage) and guest electron densities, while maximising their electrostatic interactions. $ed\_factor = 0.9999$

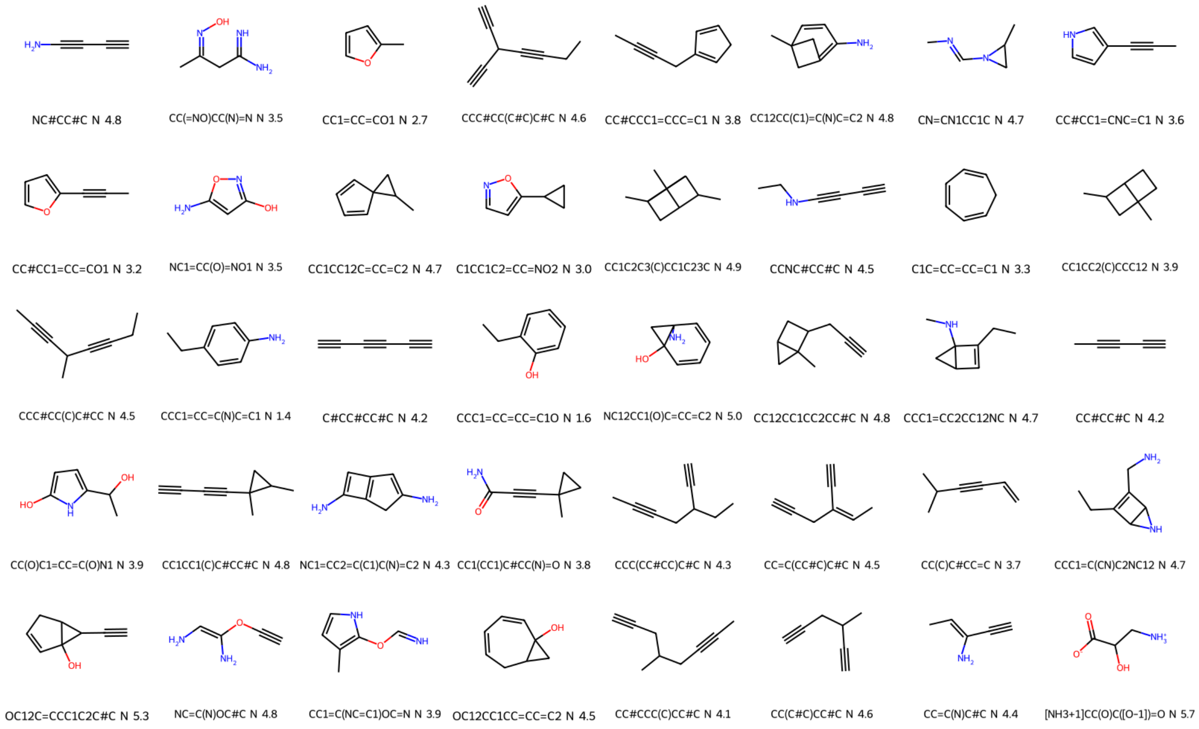

Supplementary Figure 214: Molecules obtained when trying to minimise the overlapping between host (cage) and guest electron densities, while maximising their electrostatic interactions. $ed\_factor = 1$

Supplementary Section 3 Experimental validation

Supplementary Subsection 3.1 General experimental section

3.1.1 Solvents and commercial reagents

Solvents and commercial reagents were used as received. All reactions requiring anhydrous conditions were carried-out in oven-dried glassware. All reactions (not performed in an NMR tubes) were agitated using magnetic stirrer bars. Room temperature is taken as 293 K. Flash column chromatography were carried out using silica gel cartages on a Biotage Selekt automated flash system. Cucurbit[6]uril, 2-aminobutane, N-ethylmethylamine and isopropylamine were sourced from Sigma-Aldrich. N-methylpropylamine, 3-aminopentane, N-methyl-2-butylamine, 4-aminoheptane, (prop-2-yn-1-yl)(propan-2-yl)amine, 3,4-dimethylcyclopent-2-en-1-one, 2,5-dimethyl-1H-pyrrole-3-carbonitrile and m-tolunitrile were sourced from Fluorochem. 1-Methyl-prop-2-ynylamine was sourced from Apollo Scientific. 2-Ethynyl-3-methylpyrazine was sourced from "abcr".

3.1.2 NMR

NMR spectra were recorded on a Bruker Avance III HD 600 spectrometer operating at 600.1 MHz for ^{1}H, respectively. NMR spectra were digitally processed (phase and baseline corrections, integration, peak analysis) using MestReNova 14.0.0. Chemical shifts are reported in parts per million (ppm) using residual protonated solvent signals as reference (for ^{1}H NMR spectra D_2O = 4.79 ppm, DMSO-d_6 = 2.50 ppm).

Supplementary Subsection 3.2 Synthesis

3.2.1 Synthesis of ligand 1

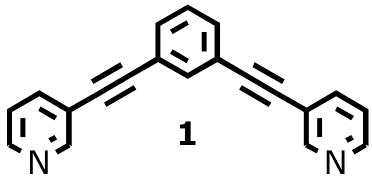

Supplementary Figure 215: Ligand 1. Chemical structure of ligand 1.

Ligand **1** (Supplementary Figure 215) was synthesized as described in the literature. NMR and mass data were consistent with those previously reported.[4]

3.2.2 Synthesis of cage $[Pd_2\mathbf{1}_4](BArF)_4$

Cage $[Pd_2\mathbf{1}_4](BArF)_4$ (Supplementary Figure 216) was synthesized as described in the literature. NMR and mass data were consistent with those previously reported.[5]

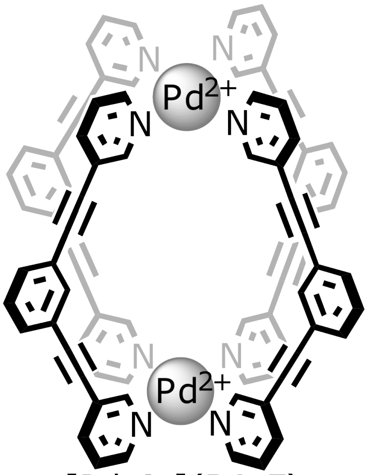

Supplementary Figure 216: Cage $[Pd_2\mathbf{1}_4](BArF)_4$. Chemical structure of cage $[Pd_2\mathbf{1}_4](BArF)_4$.

Supplementary Subsection 3.3 Cucurbituril CB[6] guest binding tritations

3.3.1 Overview and curve-fitting function

The association constant Ka between **CB[6]** and various amines was determined through ^{1}H NMR titration in D_2O/formic acid-d_2 1:1, v/v. For each titration, a solution of **CB[6]** with a guest amine was titrated into a solution of the amine, thus maintaining the concentration of the amine constant throughout the titration.

In all **CB[6]**-amine systems, a single set of signals was observed in the ^{1}H NMR spectra of the host-guest system, indicating that the system is in fast exchange on the NMR timescale. For each **CB[6]**-amine systems, the peak position of a characteristic ^{1}H NMR signal of the amine was plotted against the concentration of **CB[6]**. A global non-linear curve fitting function was then used to fit the data in Origin 2020 to a 1:1 binding model developed by *Nitschke et al.*:[6]

$$\delta = \delta_0 + \Delta\delta \frac{\left((K_a([H]_0 + [G]_0) + 1) - \sqrt{(K_a([H]_0 + [G]_0) + 1)^2 - 4K_a^2[H]_0[G]_0}\right)}{2K_a[H]_0}$$

With:

δ the measured chemical shift of the host

δ_0 the chemical shift of empty host

$\Delta\delta$ the maximal change in chemical shift of the host

K_a the binding constant

$[H]_0$ the total concentration of host

$[G]_0$ the total concentration of guest

Origin 2020 function:

```
y=y0+DY*((1+Ka*(P+x))-sqrt(((1+Ka*(P+x))^2)-4*Ka*Ka*P*x))/(2*Ka*P)
```

3.3.2 Titration data for Cucurbituril CB[6]

Interaction with N-methylpropylamine $\mathbf{G^{10}}$ NMR titrations were performed by additions of aliquots of **CB[6]** (0.02 M) in a solution of the amine $\mathbf{G^{10}}$ (0.001 M) in D_2O/formic acid-d_2 1:1, v/v, to a 0.001 M solution of amine $\mathbf{G^{10}}$ in D_2O/formic acid-d_2 1:1, v/v. The binding constant K_a (M^{-1}) of amine $\mathbf{G^{10}}$ to **CB[6]** in D_2O/formic acid-d_2 1:1, v/v at 298 K was determined to be $K_a = 5.47 \pm 0.49 \times 10^3$ M^{-1}, based on the data of the NH$\underline{CH}_3$ peak of $\mathbf{G^{10}}$. See Supplementary Figure 217.

Interaction with N-ethylmethylamine $\mathbf{G^{11}}$ NMR titrations were performed by additions of aliquots of **CB[6]** (0.01 M) in a solution of the amine $\mathbf{G^{11}}$ (0.001 M) in D_2O/formic acid-d_2 1:1, v/v, to a 0.001 M solution of amine $\mathbf{G^{11}}$ in D_2O/formic acid-d_2 1:1, v/v. The binding constant K_a (M^{-1}) of amine $\mathbf{G^{11}}$ to **CB[6]** in D_2O/formic acid-d_2 1:1, v/v at 298 K was determined to be $K_a = 4.17 \pm 0.07 \times 10^2$ M^{-1}, based on the data of the CH_3 peak of $\mathbf{G^{11}}$. See Supplementary Figure 218.

Interaction N-methyl-2-butylamine $\mathbf{G^{12}}$ NMR titrations were performed by additions of aliquots of **CB[6]** (0.001 M) in a solution of the amine $\mathbf{G^{12}}$ (0.001 M) in D_2O/formic acid-d_2 1:1, v/v, to a 0.001 M solution of amine $\mathbf{G^{12}}$ in D_2O/formic acid-d_2 1:1, v/v. The binding constant K_a (M^{-1}) of amine $\mathbf{G^{12}}$ to **CB[6]** in D_2O/formic acid-d_2 1:1, v/v at 298 K was determined to be $K_a = 1.42 \times 10^2 \pm 0.03$ M^{-1}, based on the data of the $CH_2$$\underline{\text{CH3}}$ peak of $\mathbf{G^{12}}$. See Supplementary Figure 219.

Interaction 2-aminobutane $\mathbf{G^{13}}$ NMR titrations were performed by additions of aliquots of **CB[6]** (0.02 M) in a solution of the amine $\mathbf{G^{13}}$ (0.001 M) in D_2O/formic acid-d_2 1:1, v/v, to a 0.001 M solution of amine $\mathbf{G^{13}}$ in D_2O/formic acid-d_2 1:1, v/v. The binding constant K_a (M^{-1}) of amine $\mathbf{G^{13}}$ to **CB[6]** in D_2O/formic acid-d_2 1:1, v/v at 298 K was determined to be $K_a = 1.34 \times 10^2 \pm 0.02$ M^{-1}, based on the data of the $CH_2$$\underline{\text{CH3}}$ peak of $\mathbf{G^{13}}$. See Supplementary Figure 220.

Interaction with 3-aminopentane $\mathbf{G^{14}}$ NMR titrations were performed by additions of aliquots of **CB[6]** (0.03 M) in a solution of the amine $\mathbf{G^{14}}$ (0.001 M) in D_2O/formic acid-d_2 1:1, v/v, to a 0.001 M solution of amine $\mathbf{G^{14}}$ in D_2O/formic acid-d_2 1:1, v/v. The binding constant K_a (M^{-1}) of amine $\mathbf{G^{14}}$ to **CB[6]** in D_2O/formic acid-d_2 1:1, v/v at 298 K was determined to be $K_a = 13.5 \pm 0.5$ M^{-1}, based on the data of the CH_3 peak of $\mathbf{G^{14}}$. See Supplementary Figure 221.

Interaction isopropylamine $\mathbf{G^{15}}$ NMR titrations were performed by additions of aliquots of **CB[6]** (0.02 M) in a solution of the amine $\mathbf{G^{15}}$ (0.002 M) in D_2O/formic acid-d_2 1:1, v/v, to a 0.002 M solution of amine $\mathbf{G^{15}}$ in D_2O/formic acid-d_2 1:1, v/v. The binding constant K_a (M^{-1}) of amine $\mathbf{G^{15}}$ to **CB[6]** in D_2O/formic acid-d_2 1:1, v/v at 298 K was determined to be $K_a = 92.0 \pm 0.9$ M^{-1}, based on the data of the CH_3 peaks of $\mathbf{G^{15}}$. See Supplementary Figure 222.

Interaction with (prop-2-yn-1-yl)(propan-2-yl)amine $\mathbf{G^{16}}$ NMR titrations were performed by additions of aliquots of **CB[6]** (0.02 M) in a solution of the amine $\mathbf{G^{16}}$ (0.002 M) in D_2O/formic acid-d_2 1:1, v/v, to a 0.002 M solution of amine $\mathbf{G^{16}}$ in D_2O/formic acid-d_2 1:1, v/v. The binding constant K_a (M^{-1}) of amine $\mathbf{G^{16}}$ to **CB[6]** in D_2O/formic acid-d_2 1:1, v/v at 298 K was determined to be $K_a = 2.88 \pm 0.21 \times 10^2$ M^{-1}, based on the data for the CH_3 peaks of $\mathbf{G^{16}}$. See Supplementary Figure 223.

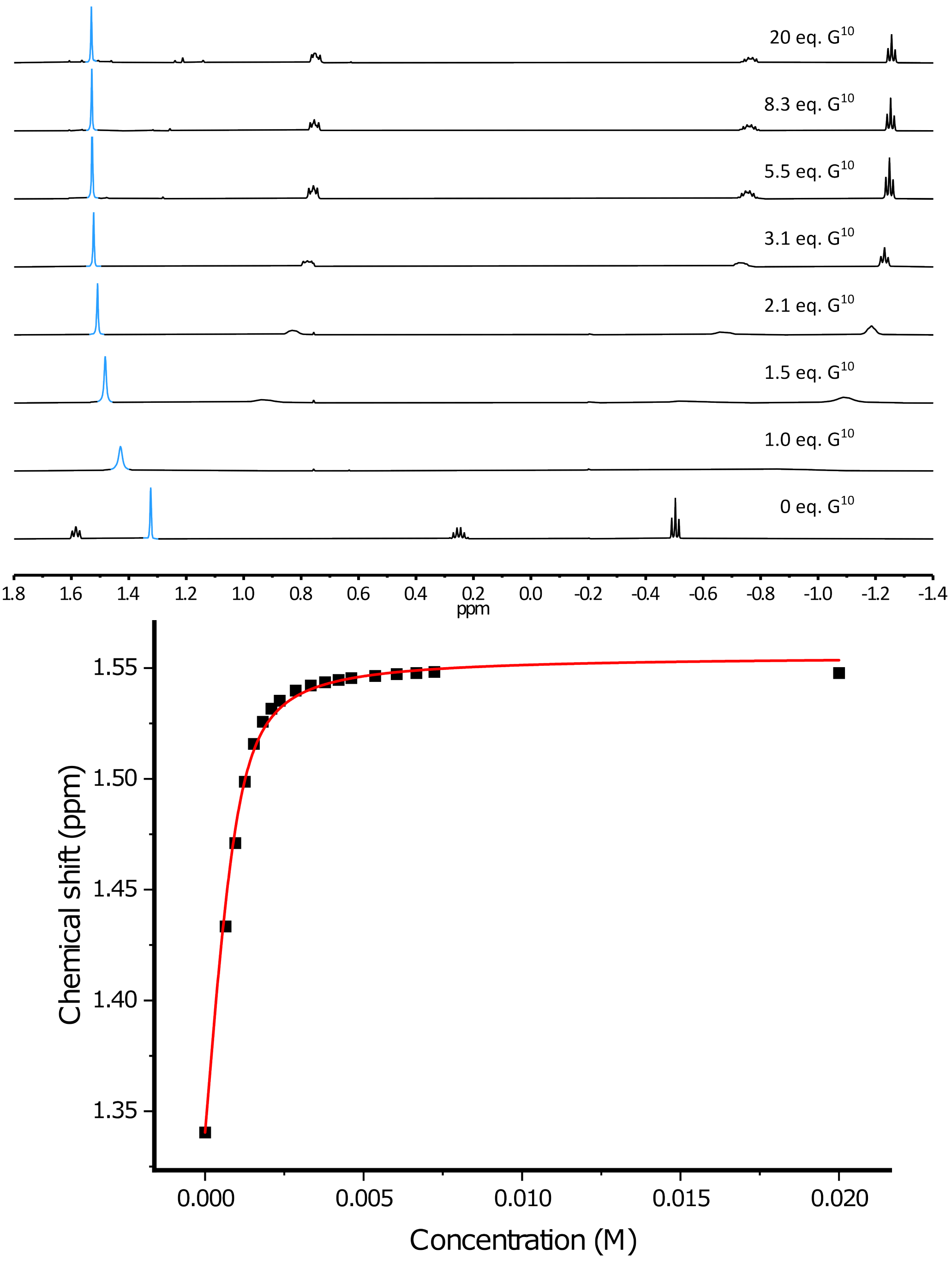

Supplementary Figure 217: Titration of amine $\mathbf{G^{10}}$ with **CB[6]** in D_2O/formic acid-d_2 1:1, v/v at 298 K. (upper) Stack plot, partial ^{1}H NMR (600 MHz, D_2O/formic acid-d_2 1:1, v/v., 298 K). (lower) Fitted curve for the NH$\underline{CH_3}$ proton at $\delta = 1.340$ ppm (highlighted in blue on the spectra).

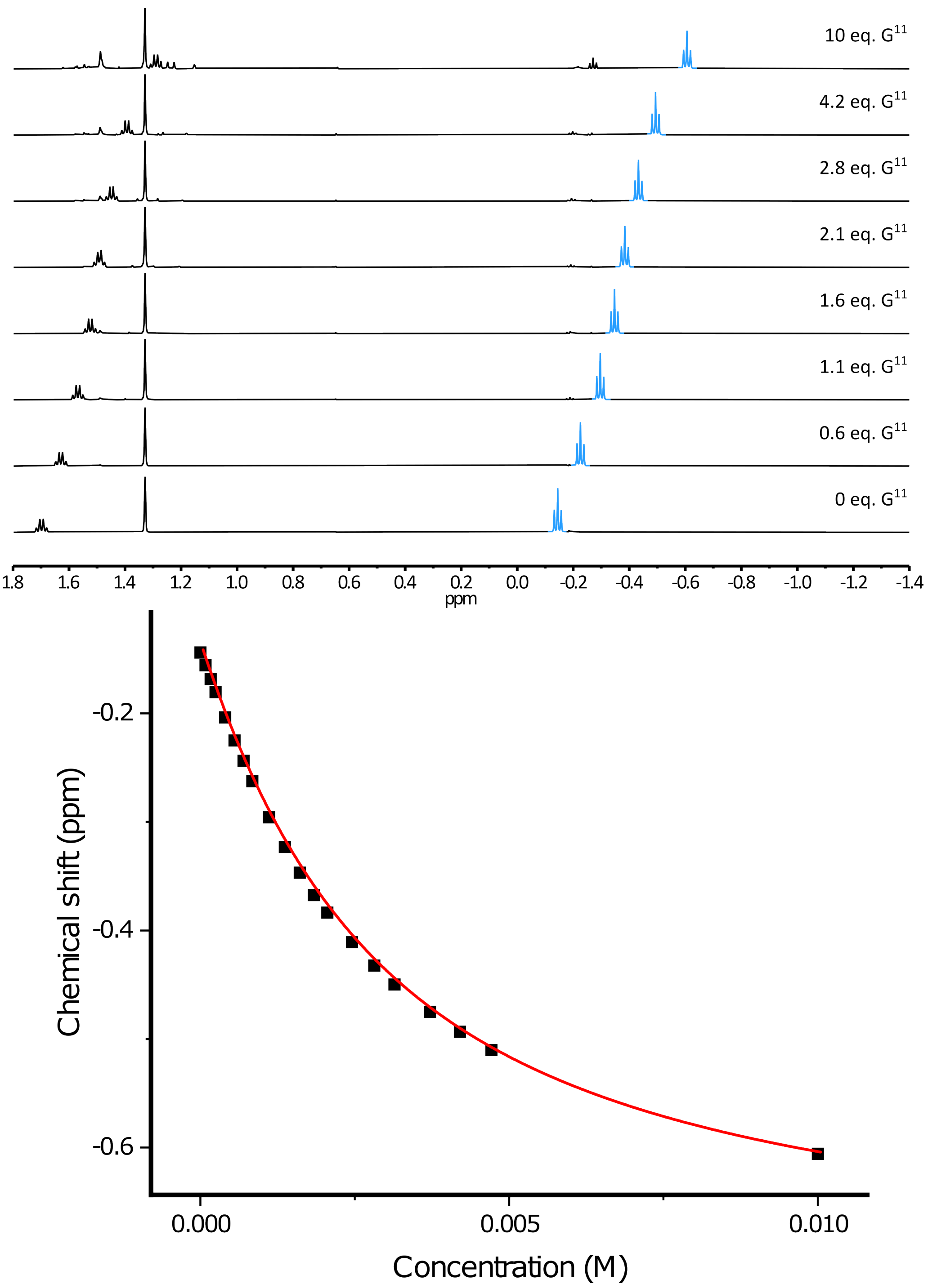

Supplementary Figure 218: Titration of amine $\mathbf{G^{11}}$ with **CB[6]** in D_2O/formic acid-d_2 1:1, v/v at 298 K. (upper) Titration data, partial ^1H NMR (600 MHz, D_2O/formic acid-d_2 1:1, v/v., 298 K). (lower) Fitted curve for the CH_3 protons at δ = -0.144 ppm (highlighted in blue on the spectra).

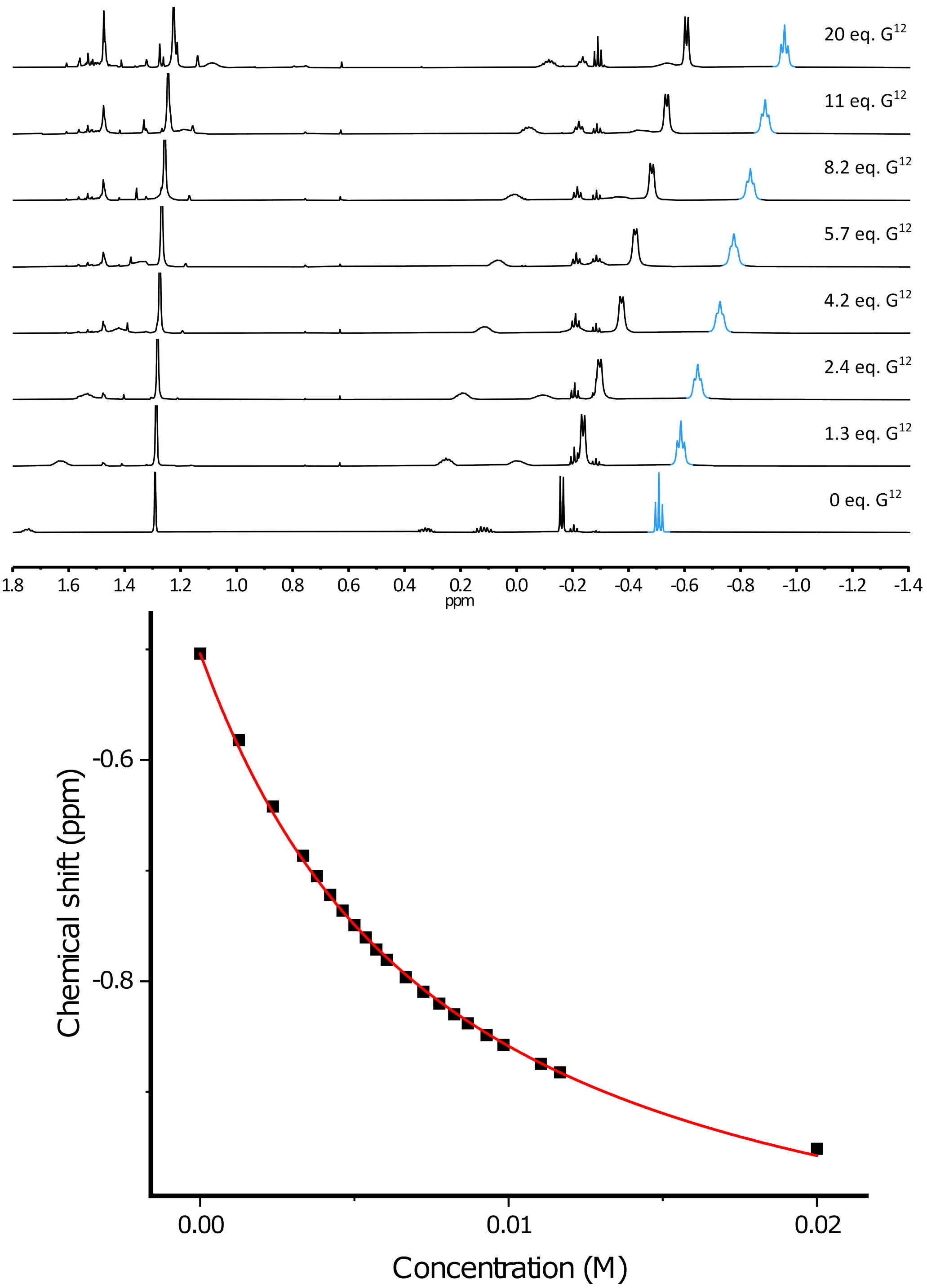

Supplementary Figure 219: Titration of amine **G**^{12} with **CB[6]** in D_2O/formic acid-d_2 1:1, v/v at 298 K. (upper) Titration data, partial ^1H NMR (600 MHz, D_2O/formic acid-d_2 1:1, v/v., 298 K). (lower) Fitted curve for the $CH_2\underline{CH3}$ protons at δ = -0.50 ppm (highlighted in blue on the spectra).

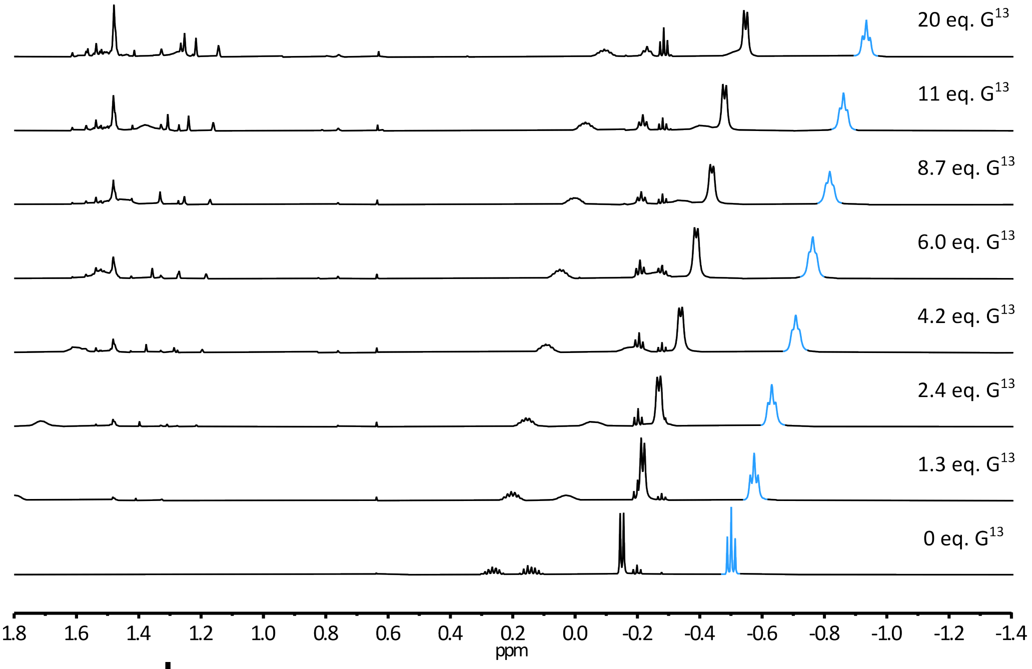

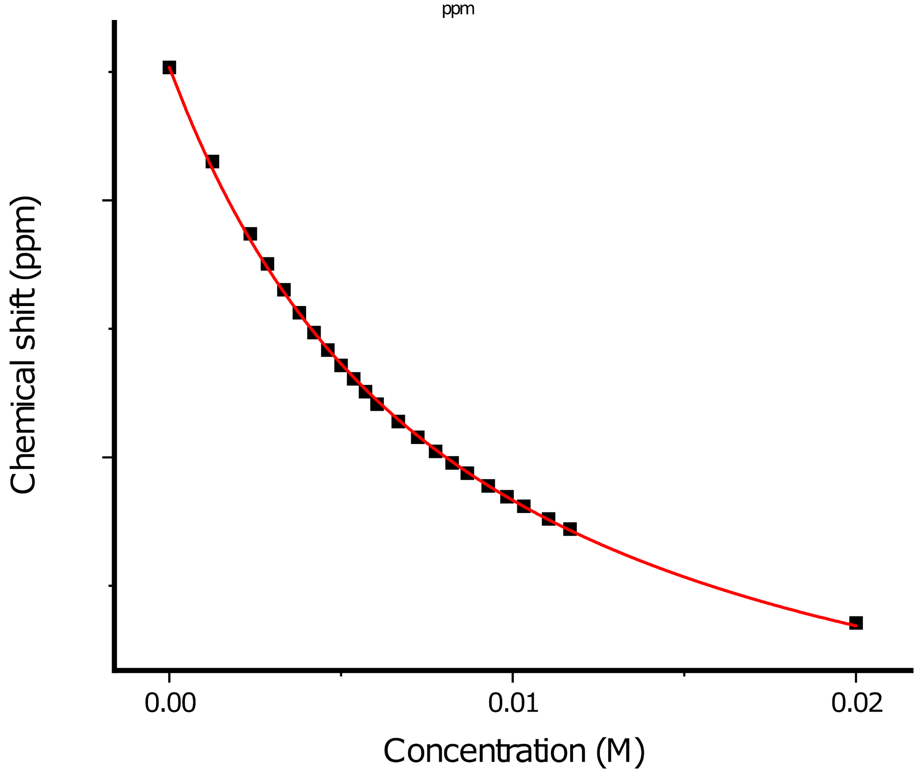

Supplementary Figure 220: Titration of amine $\mathbf{G^{13}}$ with **CB[6]** in D_2O/formic acid-d_2 1:1, v/v at 298 K. (upper) Titration data, partial ^1H NMR (600 MHz, D_2O/formic acid-d_2 1:1, v/v., 298 K). (lower) Fitted curve for the CH_2<u>CH3</u> protons at δ = -0.50 ppm (highlighted in blue on the spectra).

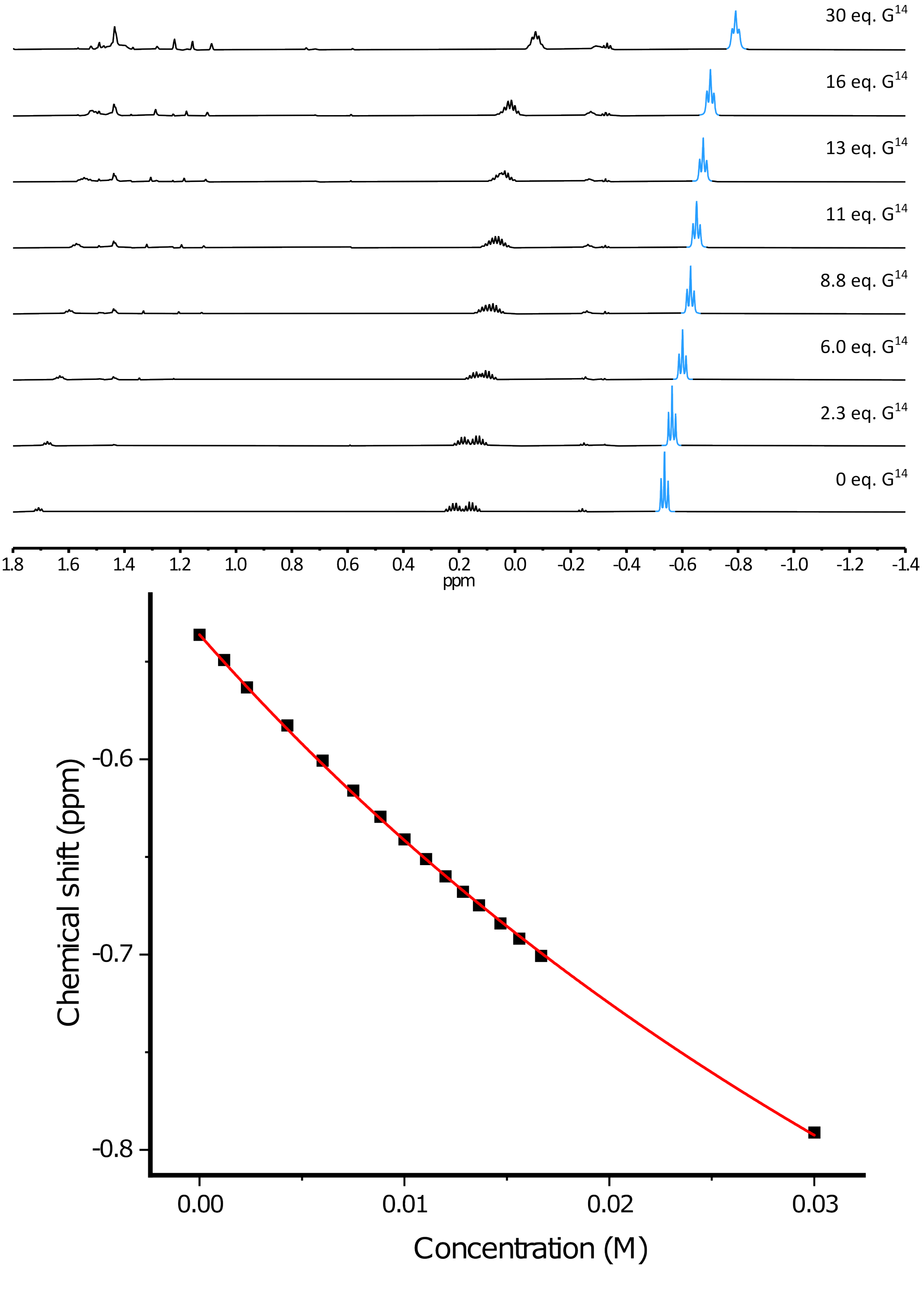

Supplementary Figure 221: Titration of amine $\mathbf{G^{14}}$ with **CB[6]** in D_2O/formic acid-d_2 1:1, v/v at 298 K. (upper) Titration data, partial ^{1}H NMR (600 MHz, D_2O/formic acid-d_2 1:1, v/v., 298 K). (lower) Fitted curve for the CH_3 protons at δ = -0.54 ppm (highlighted in blue on the spectra).

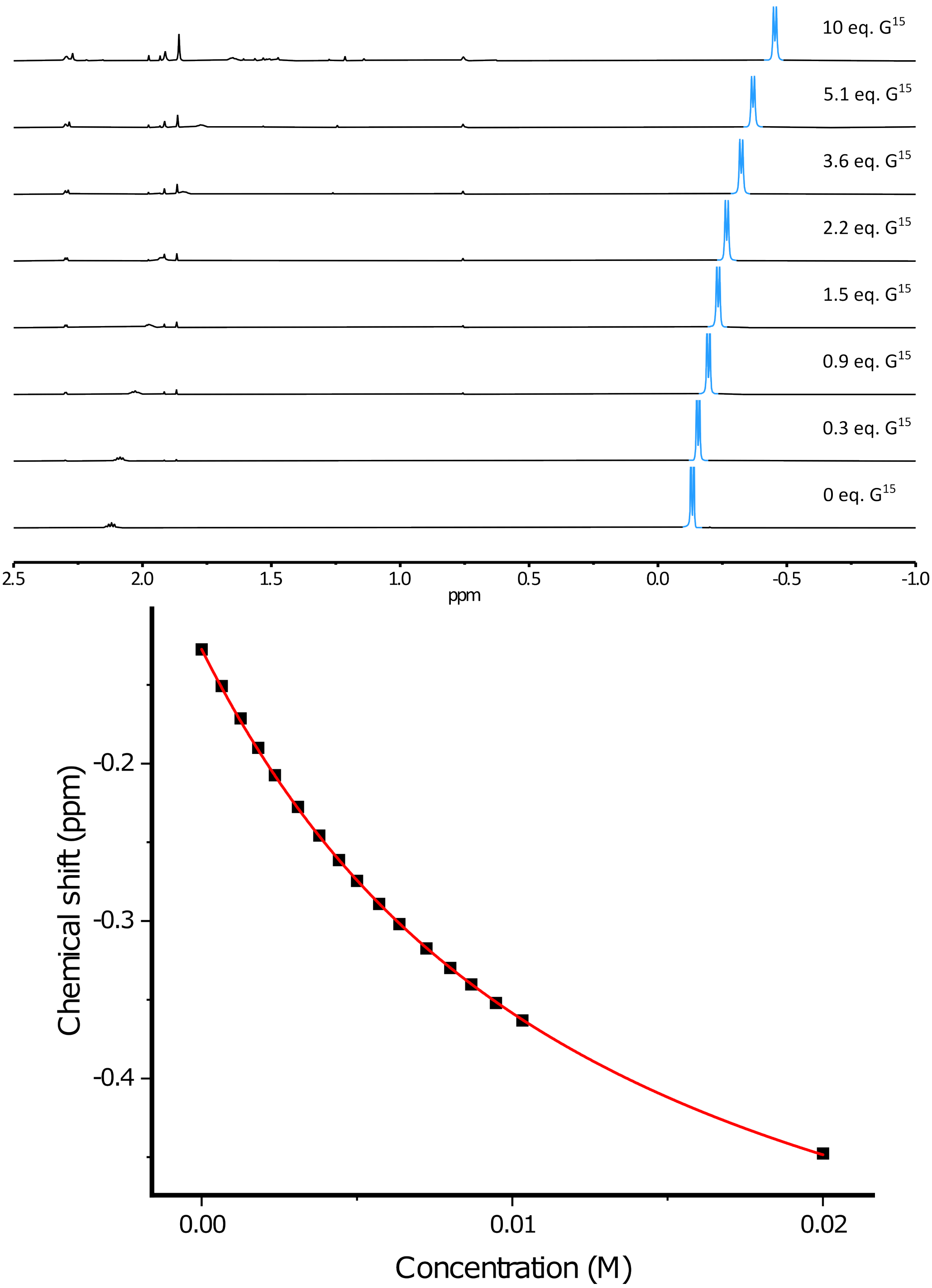

Supplementary Figure 222: Titration of amine $\mathbf{G^{15}}$ with **CB[6]** in D_2O/formic acid-d_2 1:1, v/v at 298 K. (upper) Titration data, partial ^{1}H NMR (600 MHz, D_2O/formic acid-d_2 1:1, v/v., 298 K). (lower) Fitted curve for the CH_3 protons at δ = -0.13 ppm (highlighted in blue on the spectra).

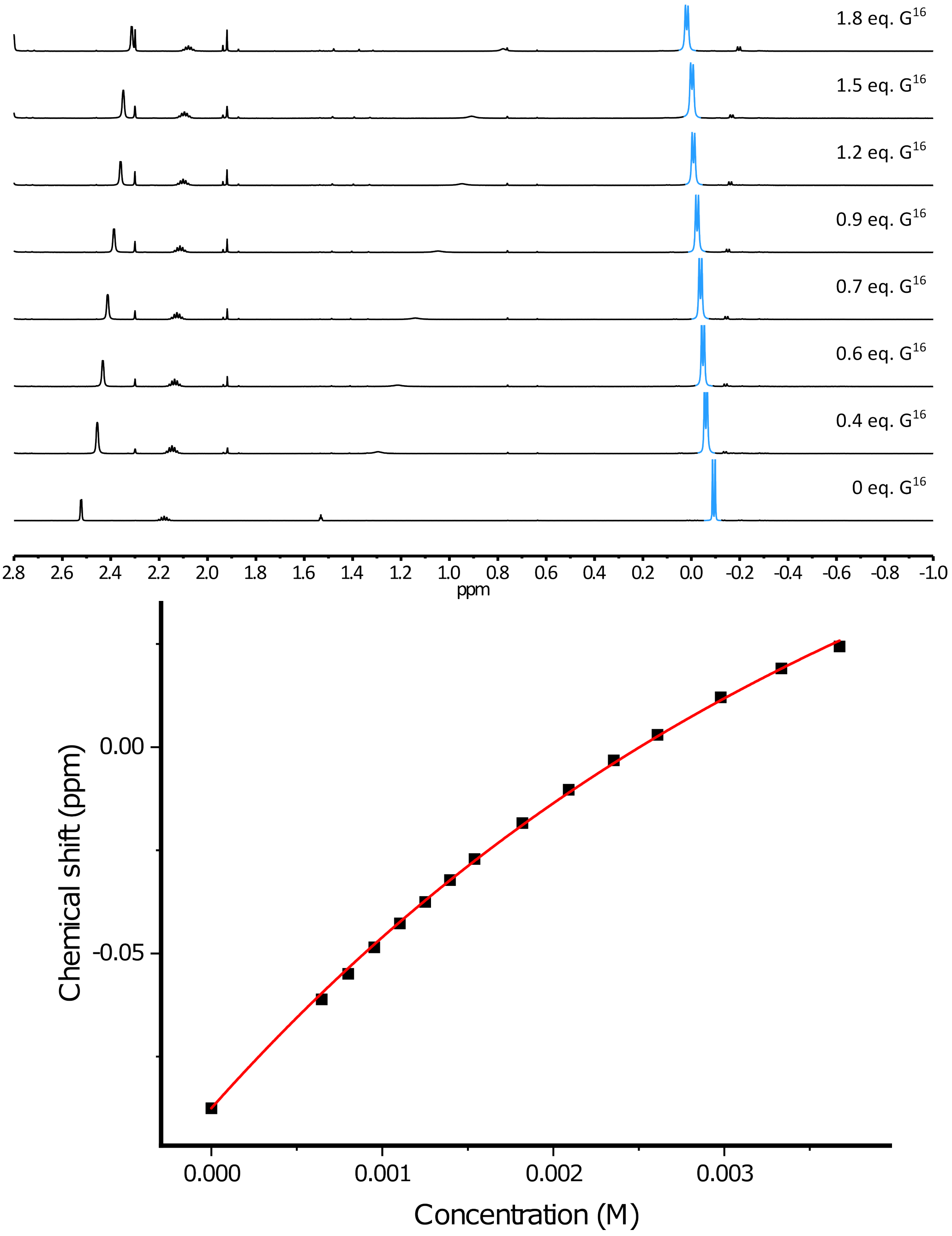

Supplementary Figure 223: Titration of amine **G16** with **CB[6]** in D_2O/formic acid-d_2 1:1, v/v at 298 K. (upper) Titration data, partial ^1H NMR (600 MHz, D_2O/formic acid-d_2 1:1, v/v., 298 K). (lower) Fitted curve for CH_3 protons at δ = -0.09 ppm (highlighted in blue on the spectra).

Supplementary Subsection 3.4 Cage $[Pd_2\mathbf{1}_4](BArF)_4$ guest binding tritations

3.4.1 Overview and curve-fitting function

The association constant K_a between $[Pd_2\mathbf{1}_4](BArF)_4$ and various guest molecules was determined through ^{1}H NMR titration in CD_2Cl_2. For each titration, a solution of $[Pd_2\mathbf{1}_4](BArF)_4$ with the studied guest was titrated into a solution of $[Pd_2\mathbf{1}_4](BArF)_4$, thus maintaining the concentration of the cage constant throughout the titration.

In all cage-guest systems, a single set of signals was observed in the ^{1}H NMR spectra of the host-guest system, indicating that the system is in fast exchange on the NMR timescale. For each cage-guest systems, the peak position of the proton Ha of the pyridines rings of the cage in the ^{1}H NMR spectrum was plotted against the concentration of the guest. A global non-linear curve fitting function was then used to fit the data in Origin 2020 to the 1:1 binding model presented in Section 3.3 [6]. See Supplementary Figure 224.

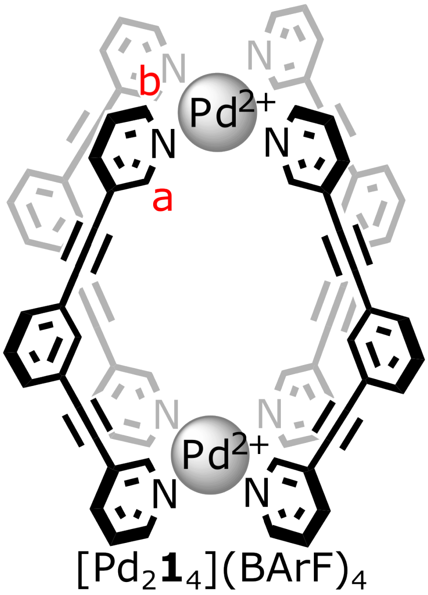

Supplementary Figure 224: Cage $[Pd_2\mathbf{1}_4](BArF)_4$. Chemical structure of cage $[Pd_2\mathbf{1}_4](BArF)_4$.

3.4.2 Titration data for Cage $[Pd_2\mathbf{1}_4](BArF)_4$

Interaction with 2-ethynyl-3-methylpyrazine $\mathbf{G^{21}}$ NMR titrations were performed by additions of aliquots of $\mathbf{G^{21}}$ (44.5 mM) in a solution of the $[Pd_2\mathbf{1}_4](BArF)_4$ (89 μM) in CD_2Cl_2, to a 89 μM solution of $[Pd_2\mathbf{1}_4](BArF)_4$ in CD_2Cl_2. The binding constant K_a (M^{-1}) of $\mathbf{G^{21}}$ to $[Pd_2\mathbf{1}_4](BArF)_4$ in CD_2Cl_2 at 298 K was determined to be $K_a = 1.01 \pm 0.03 \times 10^2$ M^{-1}, based on the data of the proton H_a of $[Pd_2\mathbf{1}_4](BArF)_4$. See Supplementary Figure 225.

Interaction with 2,5-dimethyl-1H-pyrrole-3-carbonitrile $\mathbf{G^{22}}$ NMR titrations were performed by additions of aliquots of $\mathbf{G^{22}}$ (31.1 mM) in a solution of the $[Pd_21_4]$-

$(BArF)_4$ (89 μM) in CD_2Cl_2, to a 89 μM solution of $[Pd_2\mathbf{1}_4](BArF)_4$ in CD_2Cl_2. The binding constant K_a (M^{-1}) of $\mathbf{G^{22}}$ to $[Pd_2\mathbf{1}_4](BArF)_4$ in CD_2Cl_2 at 298 K was determined to be $K_a = 5.29 \pm 0.30 \times 10^2$ M^{-1}, based on the data of the proton H_a of $[Pd_2\mathbf{1}_4](BArF)_4$. See Supplementary Figure 226.

Interaction with 3,4-dimethylcyclopent-2-en-1-one $\mathbf{G^{23}}$ NMR titrations were performed by additions of aliquots of $\mathbf{G^{23}}$ (11.1 mM) in a solution of the $[Pd_2\mathbf{1}_4](BArF)_4$ (89 μM) in CD_2Cl_2, to a 89 μM solution of $[Pd_2\mathbf{1}_4](BArF)_4$ in CD_2Cl_2. The binding constant K_a (M^{-1}) of $\mathbf{G^{23}}$ to $[Pd_2\mathbf{1}_4](BArF)_4$ in CD_2Cl_2 at 298 K was determined to be $K_a = 2.05 \pm 0.07 \times 10^2$ M^{-1}, based on the data of the proton H_a of $[Pd_2\mathbf{1}_4](BArF)_4$. Above the concentration of 11.1 mM of $\mathbf{G^{23}}$, $[Pd_2\mathbf{1}_4](BArF)_4$ started to decompose. See Supplementary Figure 227.

Interaction with m-tolunitrile $\mathbf{G^{24}}$ NMR titrations were performed by additions of aliquots of $\mathbf{G^{24}}$ (66.7 mM) in a solution of the $[Pd_2\mathbf{1}_4](BArF)_4$ (89 μM) in CD_2Cl_2, to a 89 μM solution of $[Pd_2\mathbf{1}_4](BArF)_4$ in CD_2Cl_2. The binding constant K_a (M^{-1}) of $\mathbf{G^{24}}$ to $[Pd_2\mathbf{1}_4](BArF)_4$ in CD_2Cl_2 at 298 K was determined to be $K_a = 4.45 \pm 0.12 \times 10^1$ M^{-1}, based on the data of the proton H_a of $[Pd_2\mathbf{1}_4](BArF)_4$. See Supplementary Figure 228.

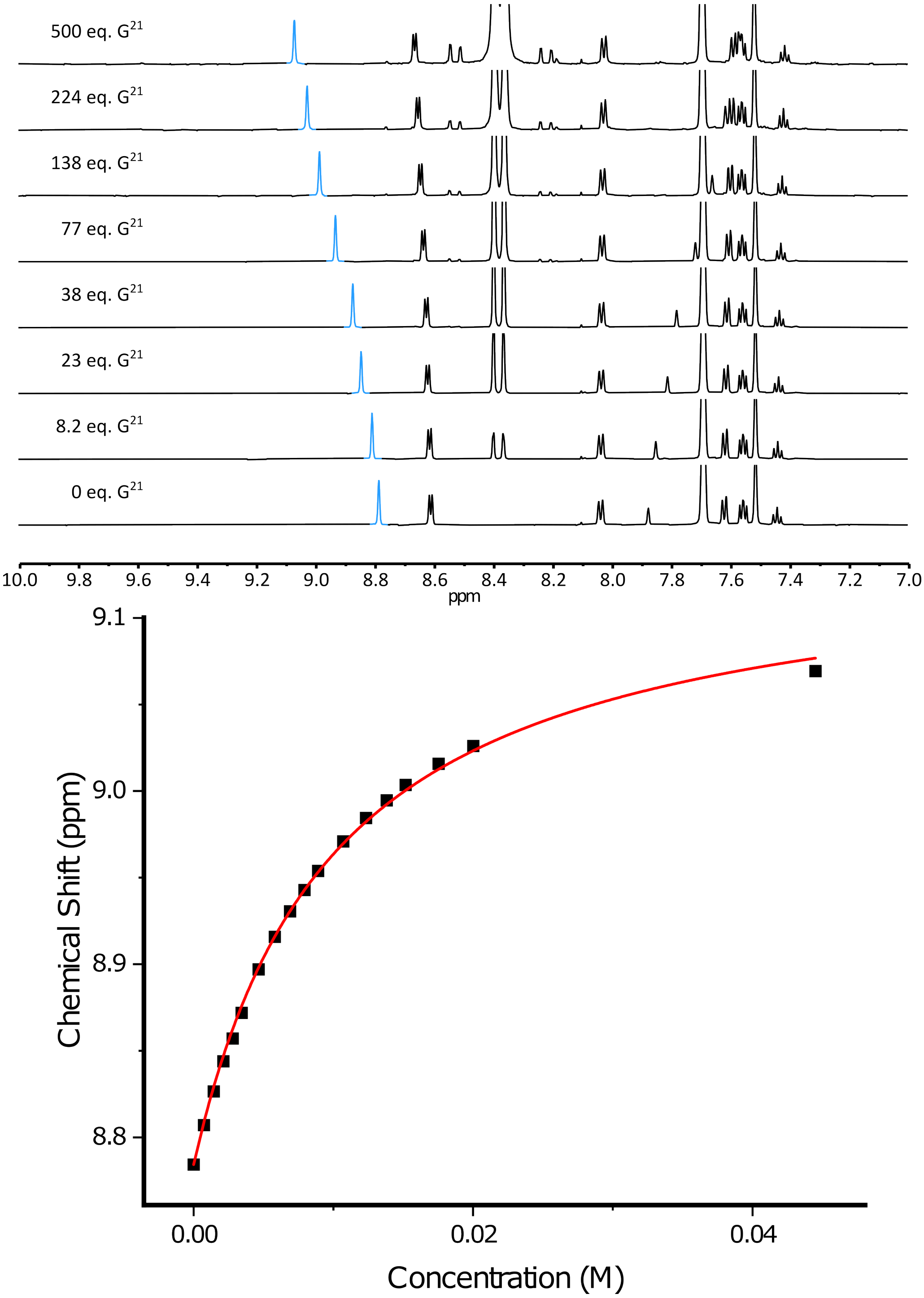

Supplementary Figure 225: Titration of $[Pd_2\mathbf{1}_4](BArF)_4$ with $\mathbf{G^{23}}$ inCD_2Cl_2 at 298 K. (upper) Titration data, partial ^{1}H NMR (600 MHz, CD_2Cl_2, 298 K). (lower) Fitted curve for proton H_a at $\delta = 8.78$ ppm (highlighted in blue on the spectra).

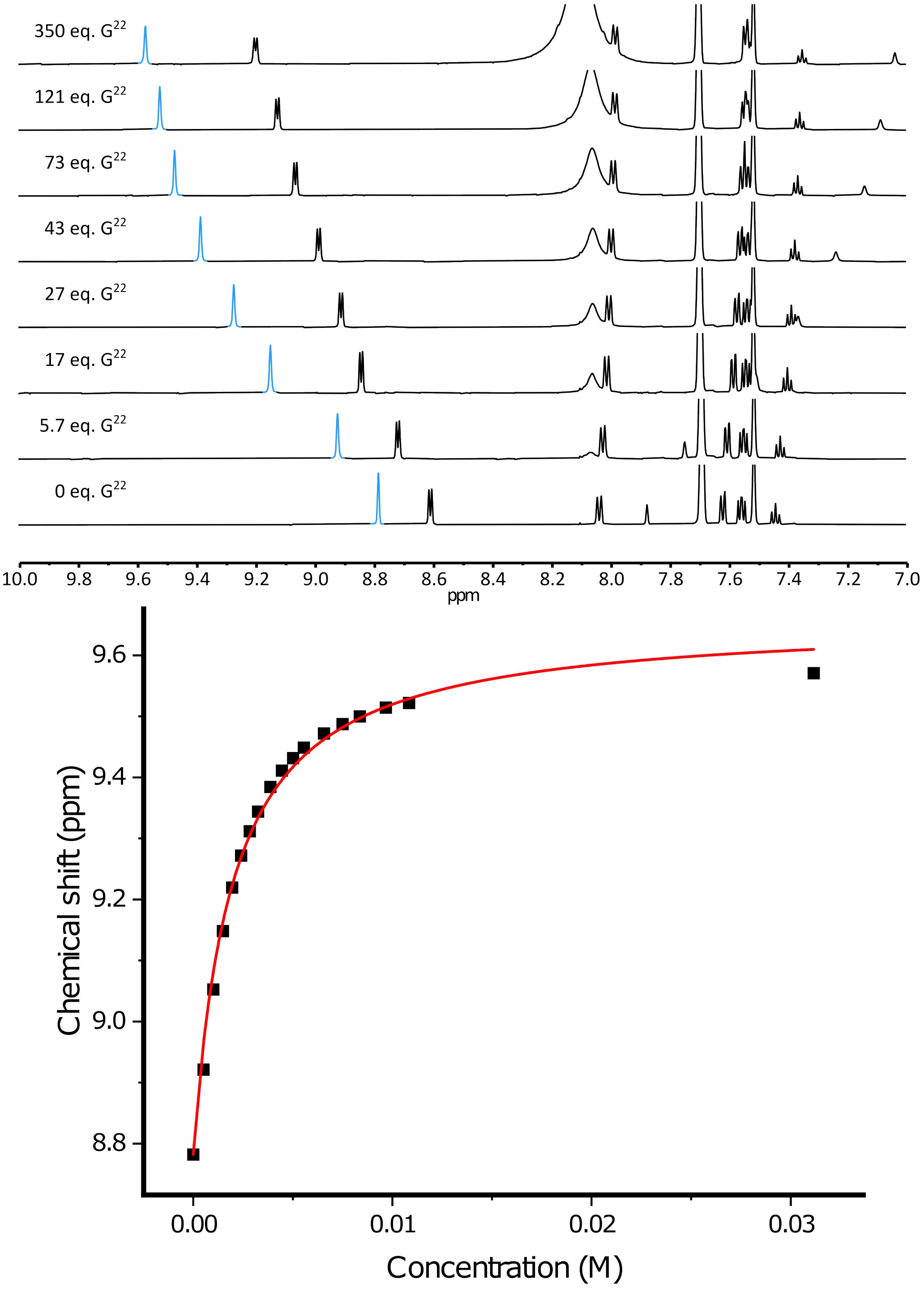

Supplementary Figure 226: Titration of $[Pd_2\mathbf{1}_4](BArF)_4$ with $\mathbf{G^{22}}$ inCD_2Cl_2 at 298 K. (upper) Titration data, partial ^{1}H NMR (600 MHz, CD_2Cl_2, 298 K). (lower) Fitted curve for proton H_a at $\delta = 8.78$ ppm (highlighted in blue on the spectra).

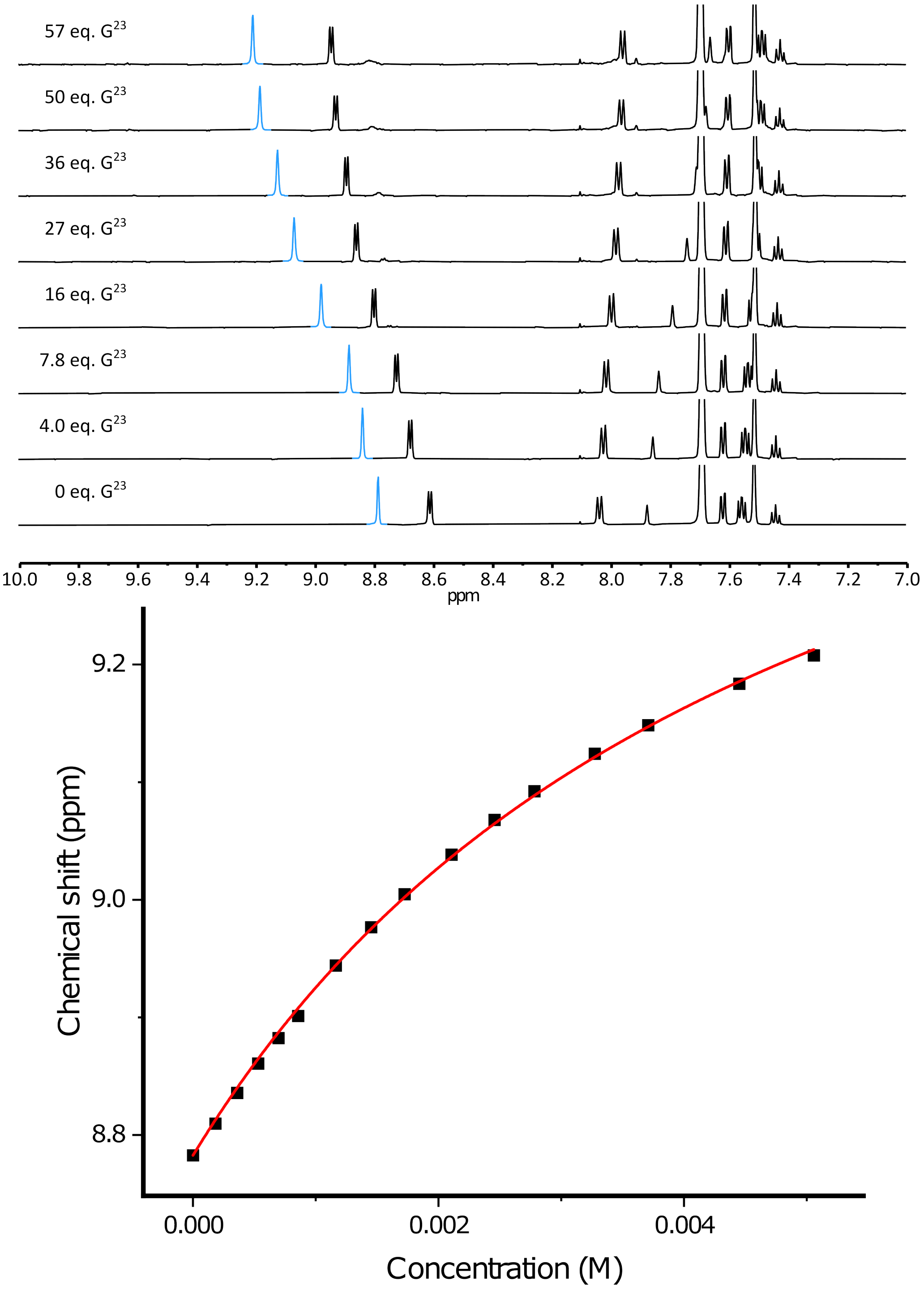

Supplementary Figure 227: Titration of $[Pd_2\mathbf{1}_4](BArF)_4$ with $\mathbf{G^{23}}$ inCD_2Cl_2 at 298 K. (upper) Titration data, partial ^{1}H NMR (600 MHz, CD_2Cl_2, 298 K). (lower) Fitted curve for proton H_a at $\delta = 8.78$ ppm (highlighted in blue on the spectra).

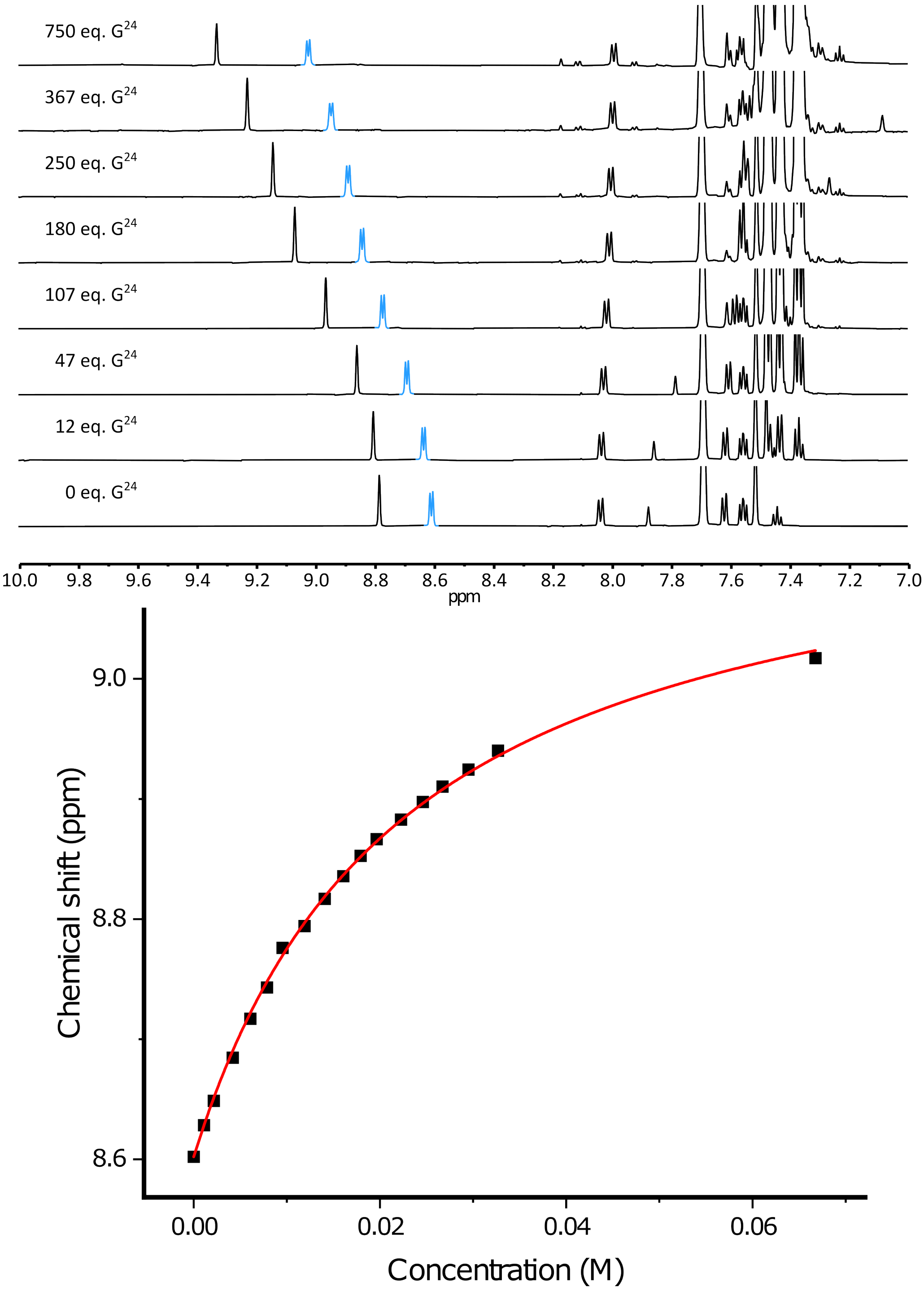

Supplementary Figure 228: Titration of $[Pd_2\mathbf{1}_4](BArF)_4$ with $\mathbf{G^{24}}$ inCD_2Cl_2 at 298 K. (upper) Titration data, partial ^{1}H NMR (600 MHz, CD_2Cl_2, 298 K). (lower) Fitted curve for proton H_a at $\delta = 8.60$ ppm (highlighted in blue on the spectra).

Supplementary Section 4 Benchmark

In order to benchmark the quality of the model-generated molecules, four different molecule sets have been compared. Each set comprises 40,000 random latent vectors generated by the means of uniform distribution with bounds ranging from 0.5 to 50. These latent vectors were input into the VAE decoder to reconstruct the 3D electron densities as well as electrostatic potentials thereof. The latter were, subsequently, input into the transformer mode in order to obtain the corresponding SMILES representations.

Two sets were generated by the means of greedy sampling, while the other two were generated by the means of probabilistic sampling (check Section 1.10.2 for more information):

- ED set only used electron densities. This set was generated using greedy sampling.
- $ED\ ESP$ set used electron densities decorated with electrostatic potential information. This set was generated using greedy sampling.
- $ED\_proba10$ set only used electron densities. This set was generated using probabilistic sampling.
- $ED\ ESP\_proba10$ set used electron densities decorated with electrostatic potential information. This set was generated using probabilistic sampling.

The degeneracy that is inherent in the design of the SMILES system was inevitably reflected in our results in the form of duplicate molecules. While most molecules occurred once or, sometimes, twice, some small fraction of molecules had as many as several thousand occurrences each, potentially reducing the size of the sets by a quarter after removal of the duplicates. The overall quality of the sets was very high, with almost all SMILES being valid and chemically plausible (i.e., passing structural filters used by popular generators like MolGen). Around 80% of the molecules were novel compared to the training set. Similarity measurements, assessing the similarity between molecules on a scale from zero (different) to one (identical) inside the new set of molecules gen erated (internal) or against the molecules in the training set (external), indicated that the molecules generated were internally diverse and divergent from the training molecules.

The following section in the Supplementary Information provides the data as well as the analysis of the aforementioned results.

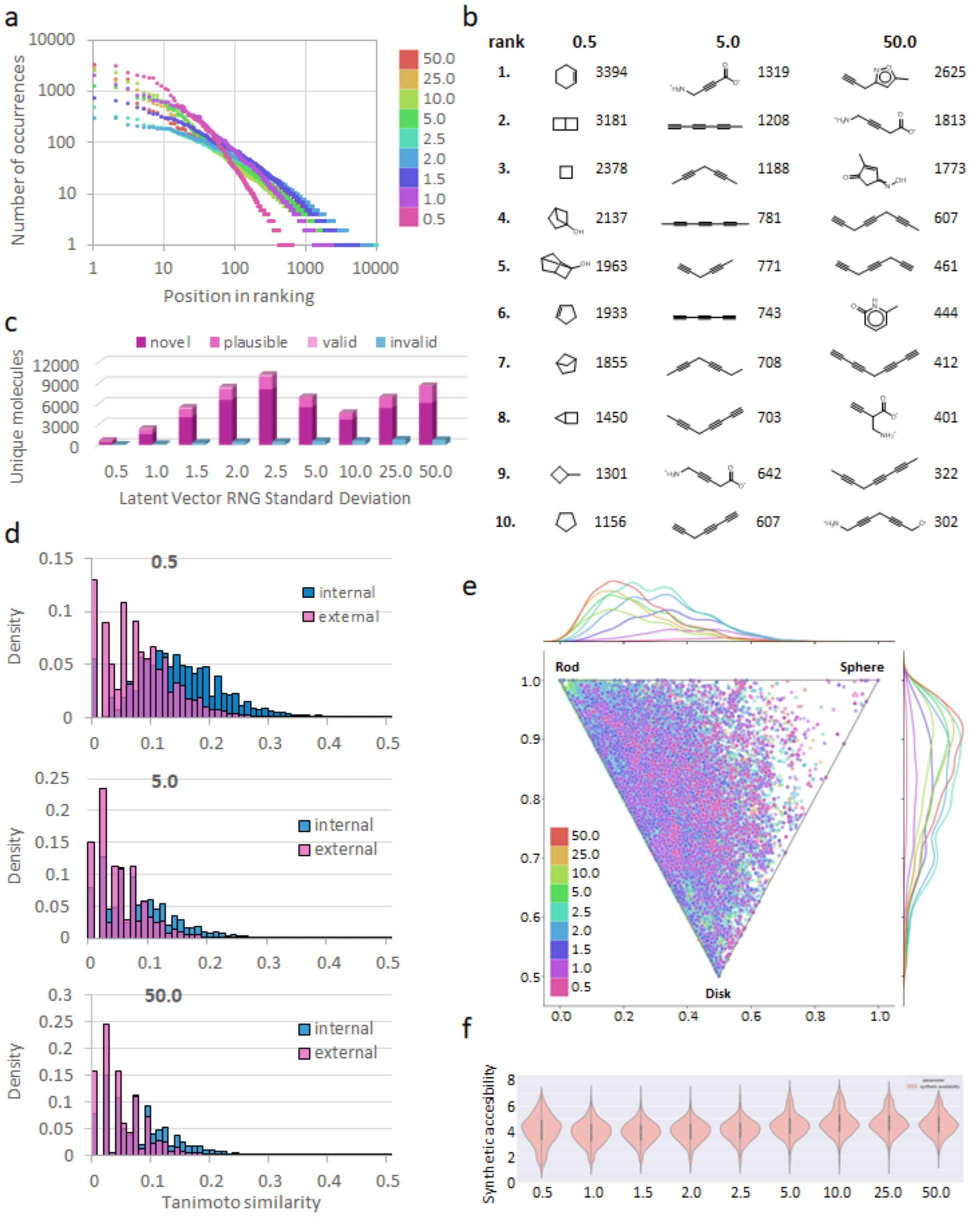

Supplementary Figure 229: Sample outline of analysis of the nine 40,000 SMILES sets generated by the means of *ED ESP* search. Each set was generated with a different random number standard deviation. **A)** Frequency of occurrence of specific molecules in the set. Molecules are ranked according to popularity. **B)** Depictions of 10 most popular structures from three chosen sets, along with the number of occurrences and their popularity rank. **C)** Total counts of valid and invalid unique molecules. Total height of pink column represents the unique valid SMILES count, where valid molecules may be chemically plausible and/or novel (as shown by different shades of pink). **D)** Diversity study of the molecules from three chosen sets. Internal similarity is where molecules are compared with each other, while external is where molecules are compared with the training set. **E)** Shapes of the molecules according to RDKit NPR calculations. **F)** Synthetic accessibility calculated by RDKit. The full analysis is shown later in the *ED ESP* chapter.

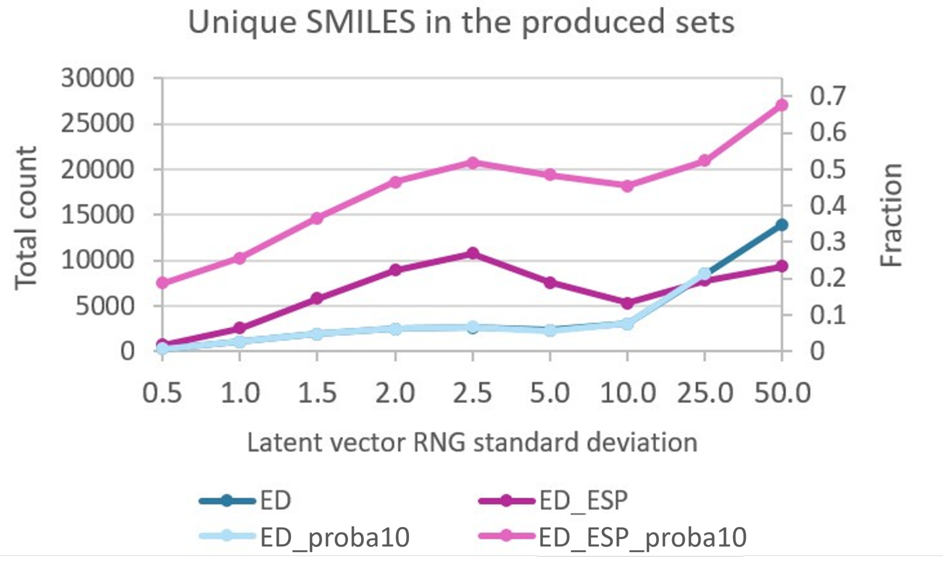

Supplementary Figure 230: The numbers of unique molecules remaining after removing duplicates from the produced data, expressed either as a total count (left axis) or a fraction of entire set (i.e., *uniqueness*, right axis). Each data point represents a set of 40,000 generated SMILES, where one SMILES may occur multiple times and multiple SMILES strings may represent the same molecule. All SMILES were transformed into their canonical form to detect such cases and properly count the number of unique encoded molecules.

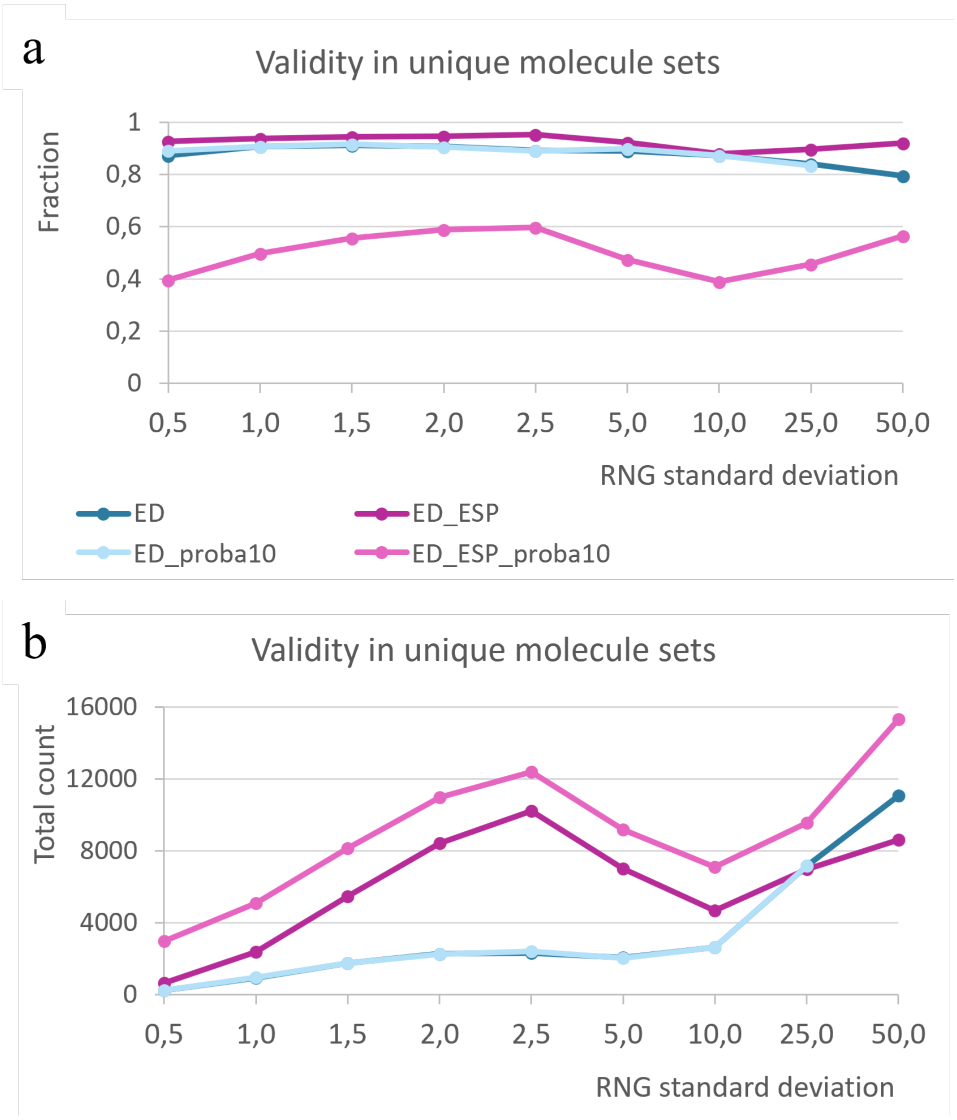

Supplementary Figure 231: The numbers of valid SMILES in unique molecule sets (i.e., after duplicates were removed). Each data point represents a different number of unique molecules, which contained within sets that originally counted 40,000 generated SMILES. Invalid SMILES, which were detected by the means of RDKit, either could not translate into any structure due to improper grammar or could not pass the sanitization test (e.g., because they contained pentavalent carbons). The numbers are expressed **A)** intensively (*validity*), i.e., of the fractions of valid molecules in the unique sets and **B)** extensively, i.e., the total counts. As seen in case of *ED_ESP_proba10*, high total count may not necessarily mean high fraction, because each set includes a different total number of unique molecules, as shown in **Figure S1.**

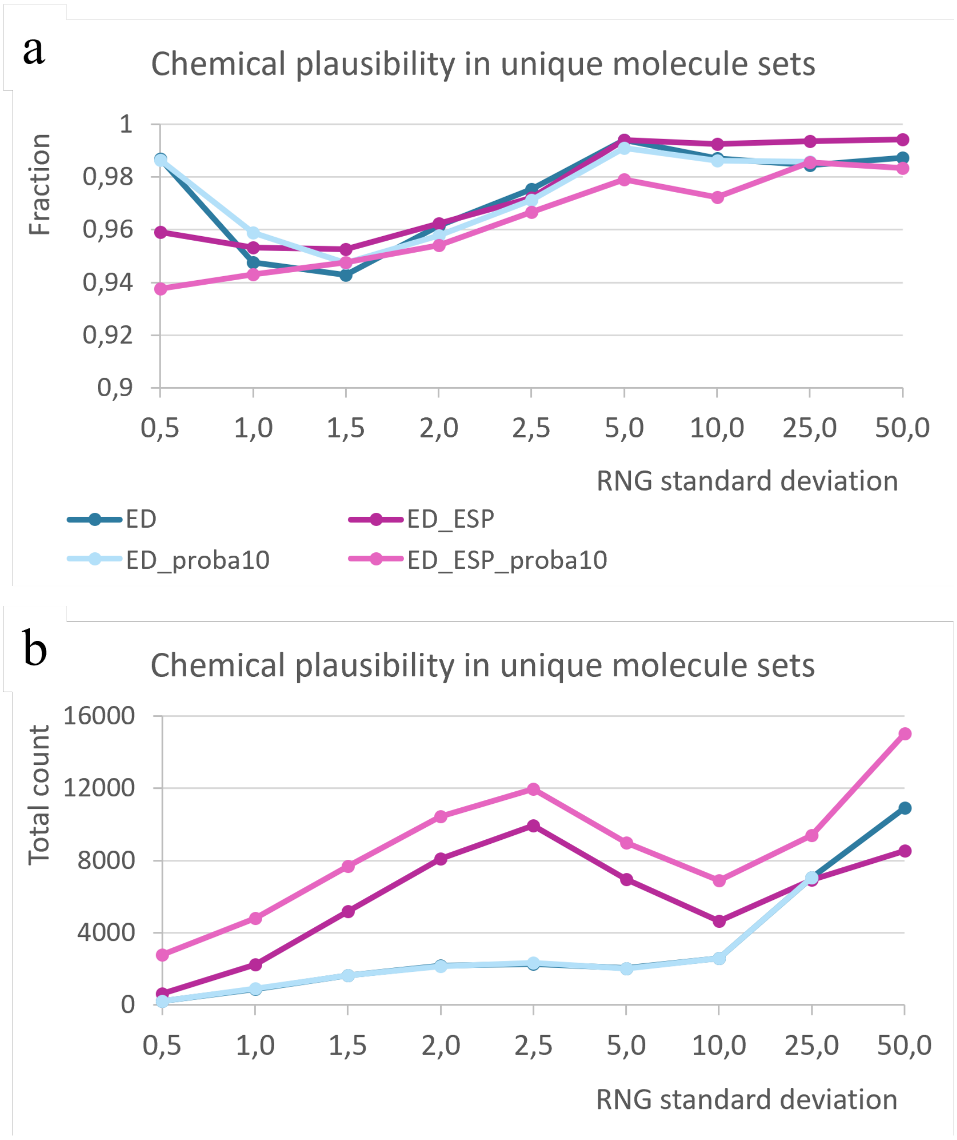

Supplementary Figure 232: The numbers of unique molecules that were chemically plausible enough to pass the structural filters used by MolGen. This represents the geometrical stability rather than synthetic accessibility. Each data point represents a different number of *unique valid* molecules that were contained within sets that originally counted 40,000 generated SMILES. The numbers are expressed **A)** intensively (*chemical plausibility*), i.e., of the fractions of positive passes in the unique sets of valid molecules only and **B)** extensively, i.e., the total counts thereof.

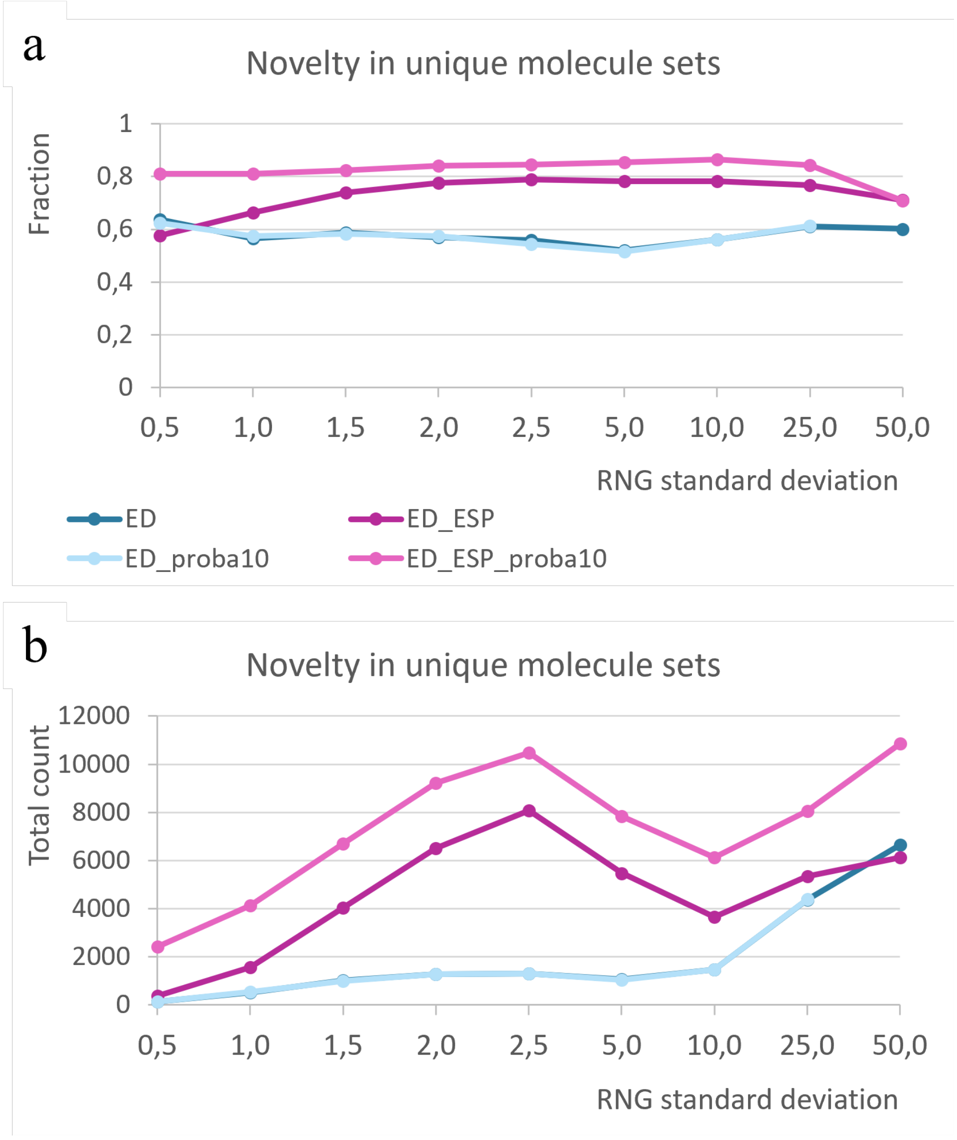

Supplementary Figure 233: The numbers of unique molecules that were novel, i.e., not present in the training set used in the machine learning processes. Each data point represents a different number of *unique valid* molecules that were contained within sets that originally counted 40,000 generated SMILES. The numbers are expressed **A)** intensively (*novelty*), i.e., of the fractions of novel molecules in the unique sets of valid molecules only and **B)** extensively, i.e., the total counts thereof.

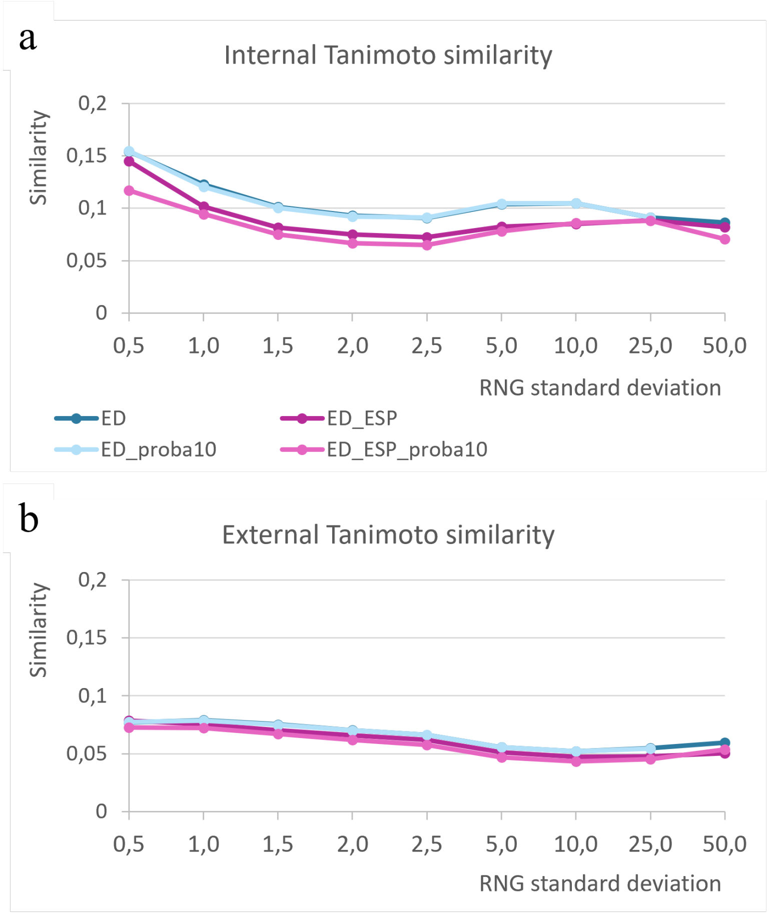

Supplementary Figure 234: The *diversity* of sets expressed by the means of **A)** internal Tanimoto similarities, i.e., where the molecules are compared with each other and **B)** external Tanimoto similarities, i.e., where the generated molecules are compared with the training set molecules. Each data point represents a different number of *unique valid* molecules that were contained within sets that originally counted 40,000 generated SMILES. The smaller the similarity, the greater the diversity.

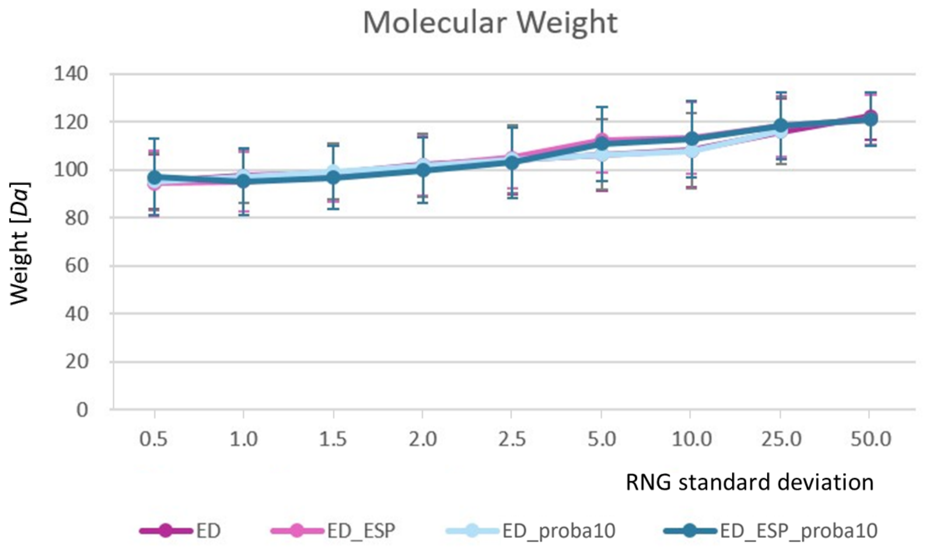

Supplementary Figure 235: Molecular weights of the generated molecules. Error bars express the standard deviation. Each data point represents a different number of *unique valid* molecules that were contained within sets that originally counted 40,000 generated SMILES.

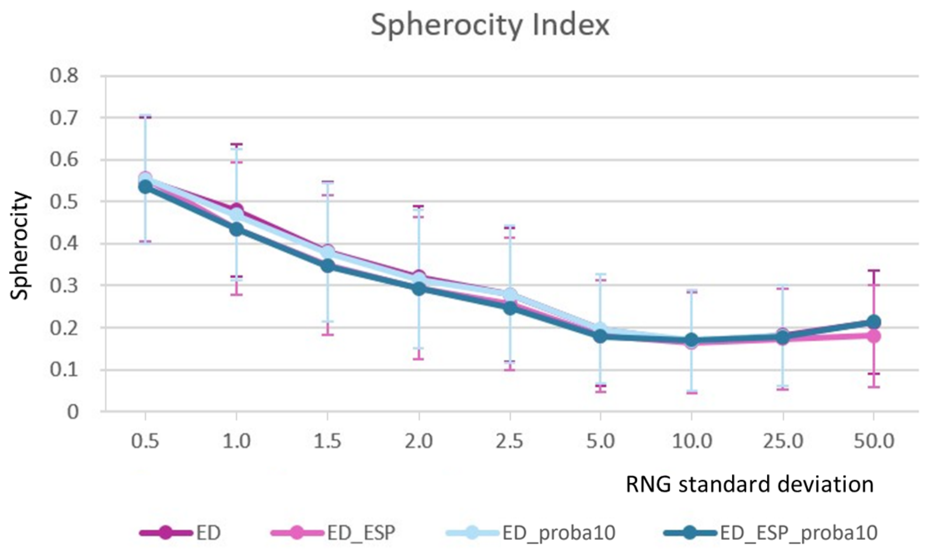

Supplementary Figure 236: Spherocity index (1 = perfectly spherical, 0 = perfectly non-spherical) of the generated molecules. Error bars express the standard deviation. Each data point represents a different number of *unique valid* molecules that were contained within sets that originally counted 40,000 generated SMILES.

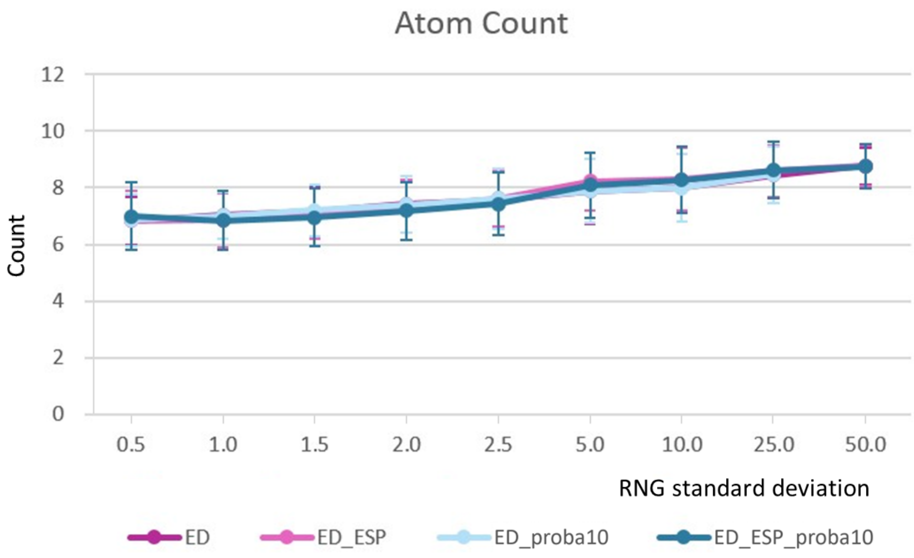

Supplementary Figure 237: Average atom counts of the generated molecules. Error bars express the standard deviation. Each data point represents a different number of *unique valid* molecules that were contained within sets that originally counted 40,000 generated SMILES, i.e., each *unique* molecule only counts once regardless of how many times it appeared in the set.

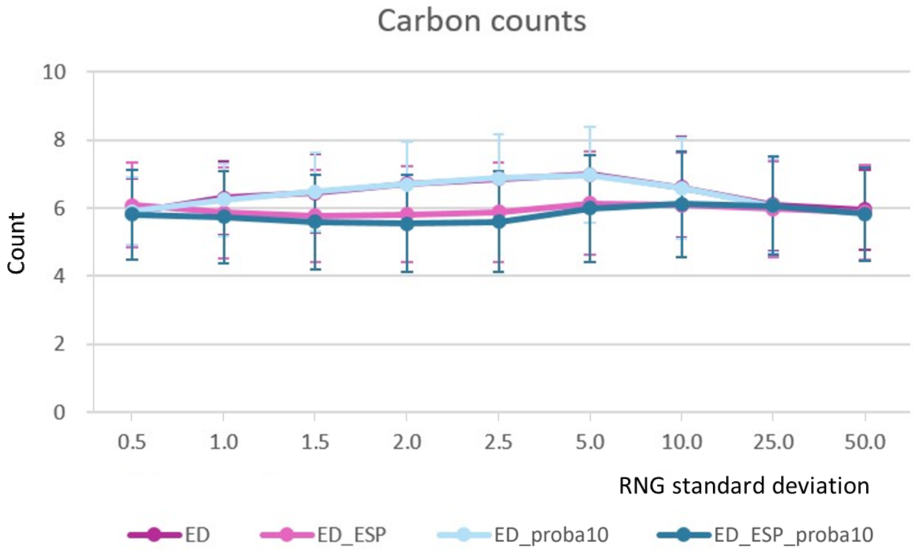

Supplementary Figure 238: Average carbon atom counts of the generated molecules. Error bars express the standard deviation. Each data point represents a different number of *unique valid* molecules that were contained within sets that originally counted 40,000 generated SMILES, i.e., each *unique* molecule only counts once regardless of how many times it appeared in the set.

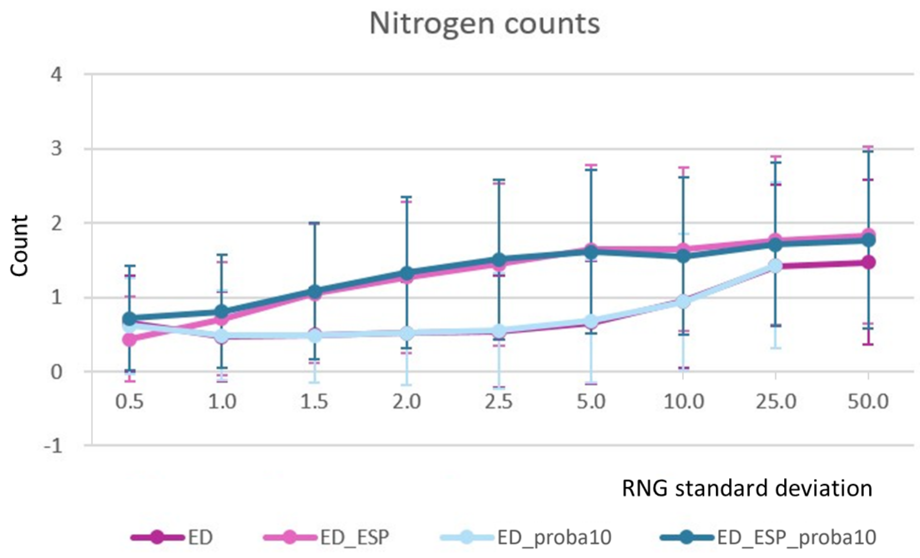

Supplementary Figure 239: Average nitrogen atom counts of the generated molecules. Error bars express the standard deviation. Each data point represents a different number of *unique valid* molecules that were contained within sets that originally counted 40,000 generated SMILES, i.e., each *unique* molecule only counts once regardless of how many times it appeared in the set.

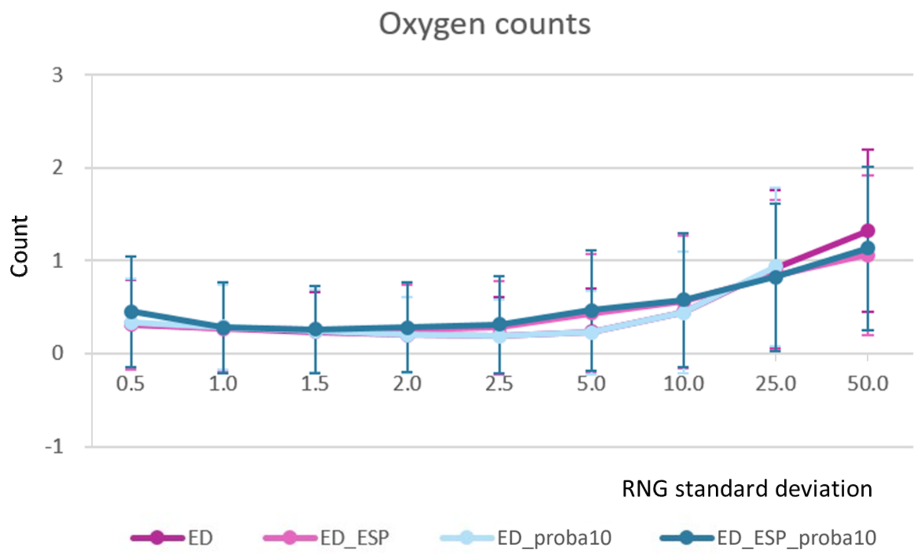

Supplementary Figure 240: Average oxygen atom counts of the generated molecules. Error bars express the standard deviation. Each data point represents a different number of *unique valid* molecules that were contained within sets that originally counted 40,000 generated SMILES, i.e., each *unique* molecule only counts once regardless of how many times it appeared in the set.

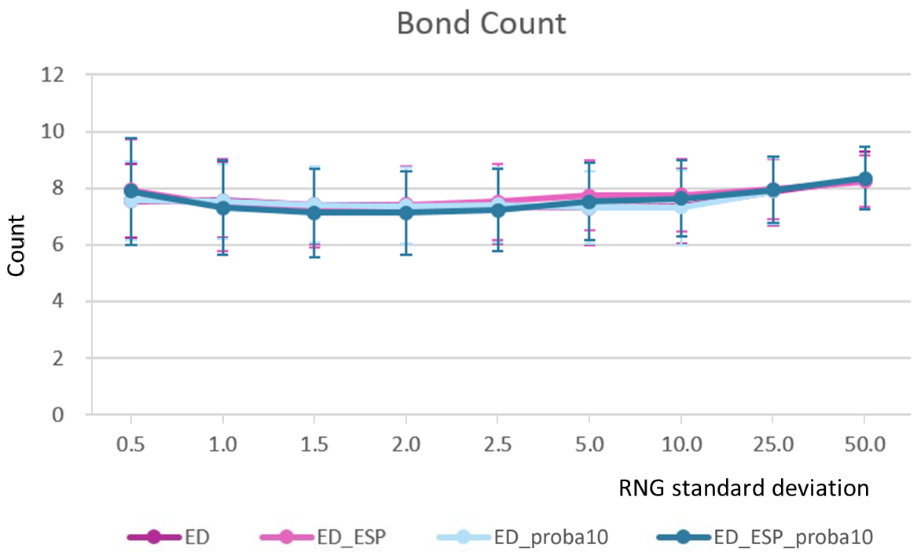

Supplementary Figure 241: Total bond counts of the generated molecules. Error bars express the standard deviation. Each data point represents a different number of *unique valid* molecules that were contained within sets that originally counted 40,000 generated SMILES, i.e., each *unique* molecule only counts once regardless of how many times it appeared in the set.

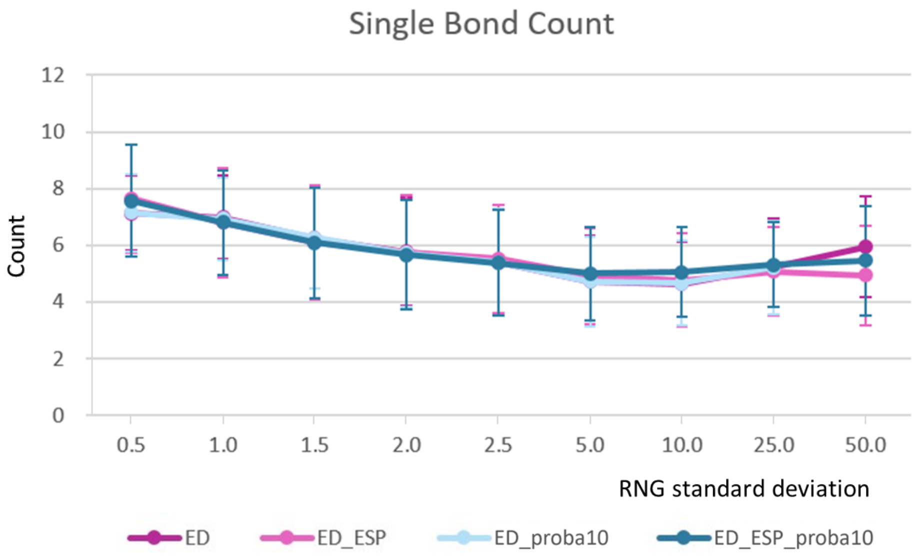

Supplementary Figure 242: Single bond counts of the generated molecules. Error bars express the standard deviation. Each data point represents a different number of *unique valid* molecules that were contained within sets that originally counted 40,000 generated SMILES, i.e., each *unique* molecule only counts once regardless of how many times it appeared in the set.

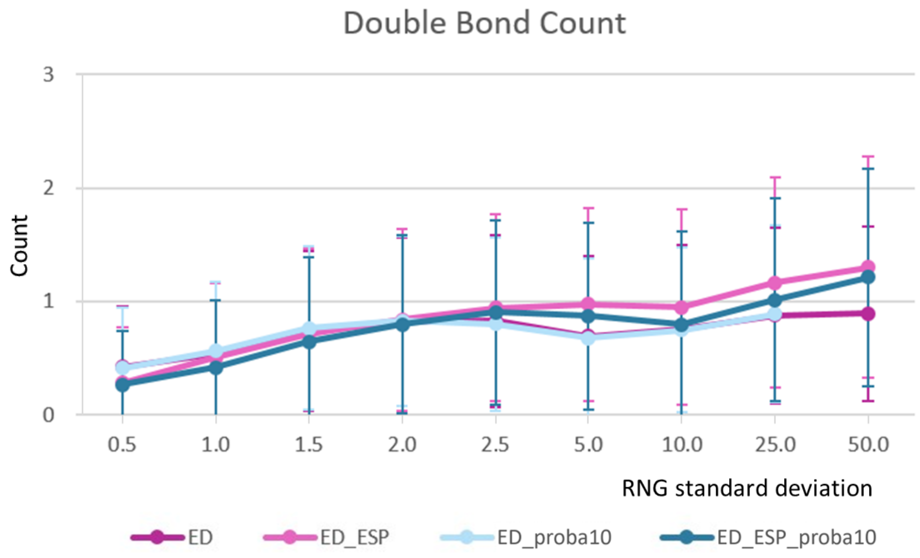

Supplementary Figure 243: Double bond counts of the generated molecules. Error bars express the standard deviation. Each data point represents a different number of *unique valid* molecules that were contained within sets that originally counted 40,000 generated SMILES, i.e., each *unique* molecule only counts once regardless of how many times it appeared in the set.

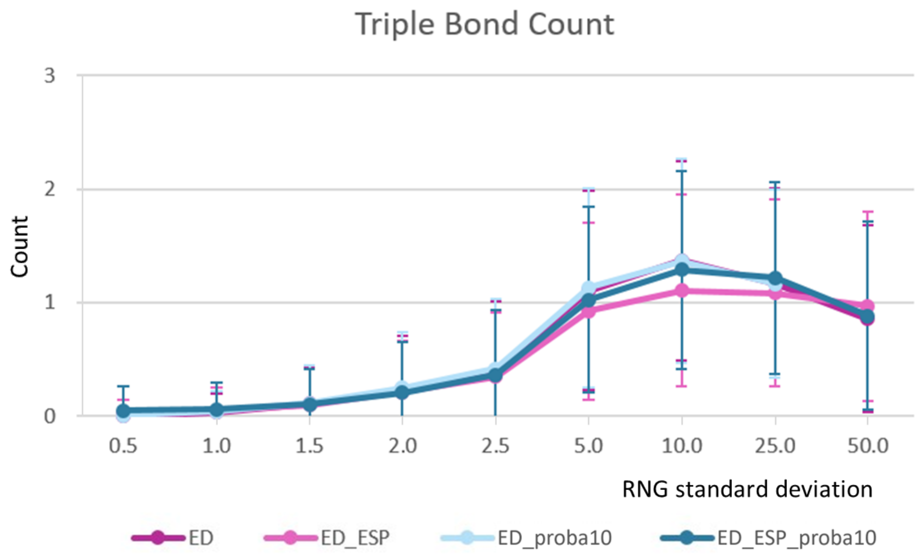

Supplementary Figure 244: Double bond counts of the generated molecules. Error bars express the standard deviation. Each data point represents a different number of *unique valid* molecules that were contained within sets that originally counted 40,000 generated SMILES, i.e., each *unique* molecule only counts once regardless of how many times it appeared in the set.

Supplementary Subsection 4.1 Electron Density (ED)

Here is the analysis of the first individual method, **ED**. Its results comprise nine datasets of 40,000 SMILES each, together representing an STD range from **0.5** to **50.0**.

**ED**	**unique**		**valid**	
**std**	**count**	**fraction**	**count**	**fraction**
**0.5**	262	<0.01	229	<0.01
**1.0**	1029	0.03	935	0.02
**1.5**	1897	0.05	1730	0.04
**2.0**	2492	0.06	2261	0.06
**2.5**	2598	0.06	2321	0.06
**5.0**	2293	0.06	2044	0.05
**10.0**	3006	0.08	2626	0.07
**25.0**	8502	0.21	7155	0.18
**50.0**	13910	0.35	11060	0.28

Supplementary Table 1: Number and fraction of unique and unique valid molecules in datasets generated by the means of ED method. Each dataset corresponds to a different RNG StD and includes a total of 40,000 non-unique SMILES.

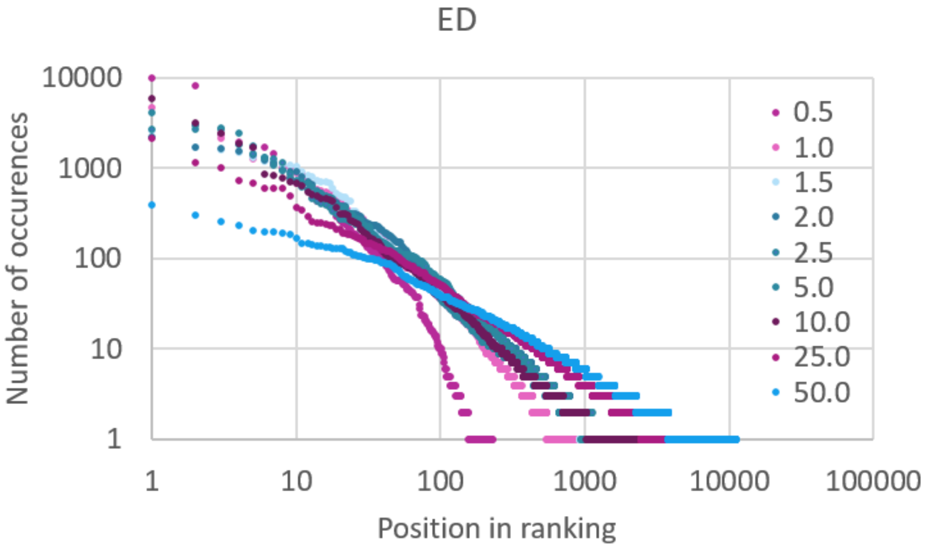

Supplementary Figure 245: Frequency of occurrence of specific valid SMILES in produced results. The plot ranks SMILES according to the number of times they appear in sets of 40,000 generated SMILES. Invalid SMILES are omitted.

ED	total	passes MolGen filters		novel vs training set		novel vs test set		commercially available	
std		count	fraction	count	fraction	count	fraction	count	fraction
**0.5**	229	226	0.99	146	0.64	216	0.94	68	0.30
**1.0**	935	886	0.95	528	0.56	876	0.94	240	0.26
**1.5**	1730	1631	0.94	1014	0.59	1633	0.94	418	0.24
**2.0**	2261	2174	0.96	1288	0.57	2158	0.95	496	0.22
**2.5**	2321	2264	0.98	1299	0.56	2208	0.95	493	0.21
**5.0**	2044	2032	0.99	1062	0.52	1957	0.96	273	0.13
**10.0**	2626	2592	0.99	1471	0.56	2510	0.96	222	0.08
**25.0**	7155	7044	0.98	4364	0.61	6857	0.96	235	0.03
**50.0**	11060	10920	0.99	6649	0.60	10574	0.96	262	0.02

Supplementary Table 2: Number and fraction of chemically possible and novel molecules in each set of unique valid SMILES corresponding to specific RNG StD used by the ED method.

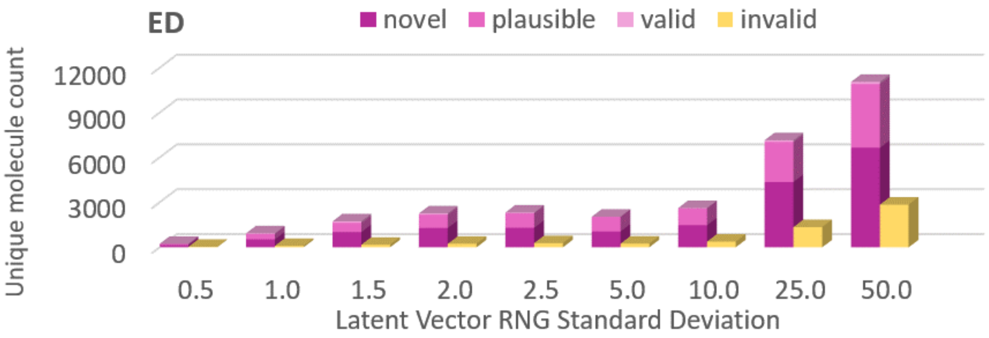

Supplementary Figure 246: Total counts of valid and invalid unique molecules. Total height of pink column represents the unique valid SMILES count, where valid molecules may be chemically plausible and/or novel (as shown by different shades of pink that overlap each other from the bottom-up).

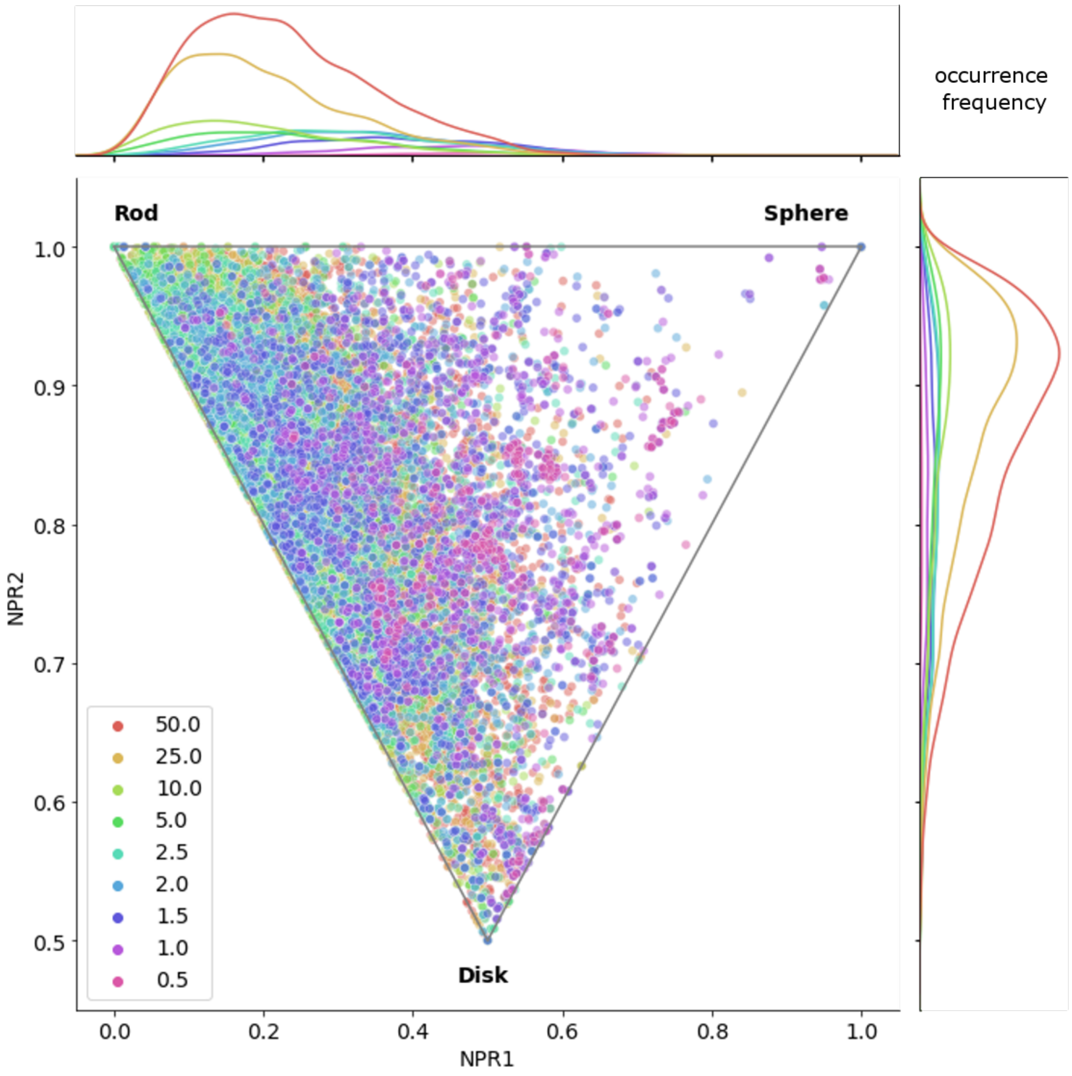

Supplementary Figure 247: Shapes of molecules from the ED sets according to RDKit NPR calculations. Each dot represents a different unique SMILES and the colors correspond to different RNG StD sets. For clarity purposes, plots have been added to the sides to show the spatial distributions of colored dots. Only those SMILES which geometry could be successfully determined by RDKit and/or OpenBabel tools were included in the plot.

4.1.1 ED 0.5

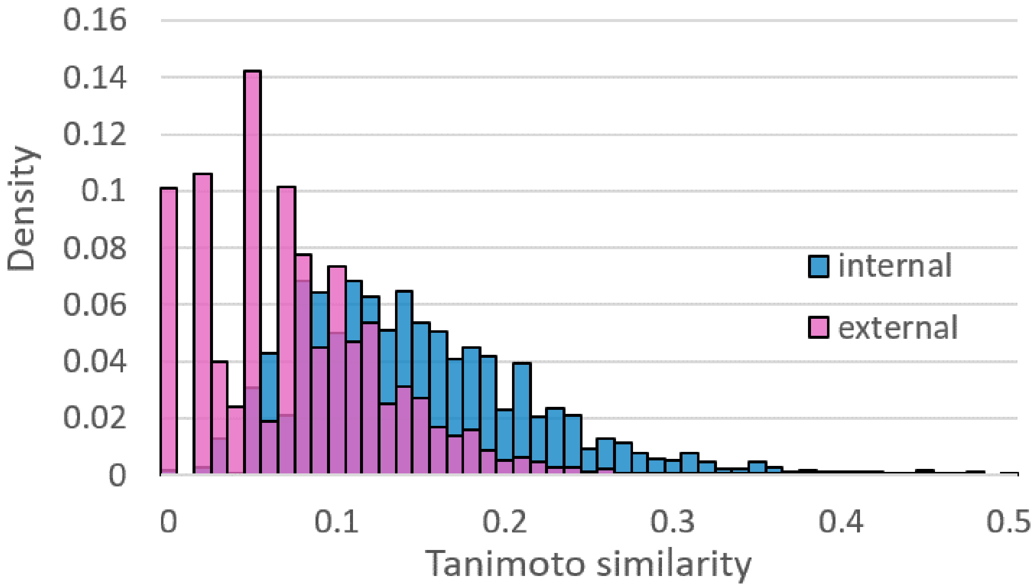

Supplementary Figure 248: Diversity study of the molecules from *ED 0.5*. Internal similarity is where molecules are compared with each other, while external is where molecules are compared with the training set.

rank	molecule	occurrences	novel	commercially available	synthetic availability
**1.**	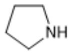	9858	yes	yes	2.19
**2.**	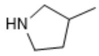	7994	yes	yes	3.04
**3.**	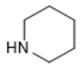	2375	yes	yes	2.06
**4.**	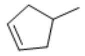	1924	yes	no	2.93
**5.**	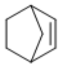	1680	no	yes	4.62
**6.**	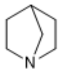	1671	yes	no	3.29
**7.**	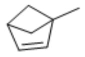	1422	no	no	4.39
**8.**	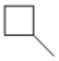	1013	yes	no	1.42
**9.**	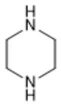	780	yes	yes	2.70
**10.**	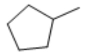	687	no	yes	1.57

Supplementary Table 3: Ten most frequently-occurring valid molecules in the ED 0.5 set, ranked by frequency. Synthetic availability was calculated by the means of RDKit.

4.1.2 ED 1.0

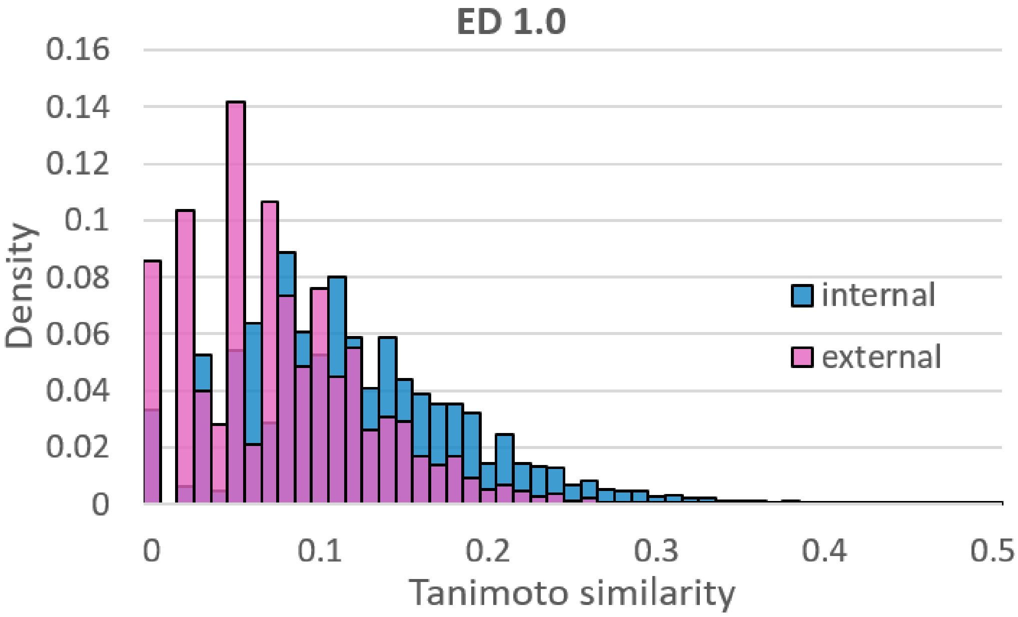

Supplementary Figure 249: Diversity study of the molecules from *ED 1.0*. Internal similarity is where molecules are compared with each other, while external is where molecules are compared with the training set.

rank	molecule	occurrences	novel	commercially available	synthetic availability
**1.**	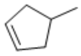	4672	**yes**	no	2.93
**2.**	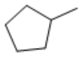	3023	no	**yes**	1.57
**3.**	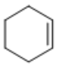	2136	no	**yes**	2.42
**4.**	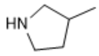	1886	**yes**	**yes**	3.04
**5.**	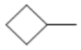	1282	**yes**	no	1.42
**6.**	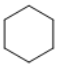	1277	**yes**	**yes**	1.00
**7.**	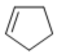	1110	no	**yes**	2.73
**8.**	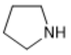	1033	**yes**	**yes**	2.19
**9.**	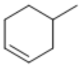	1018	no	**yes**	3.16
**10.**	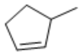	877	no	no	3.53

Supplementary Table 4: Ten most frequently-occurring valid molecules in the ED 1.0 set, ranked by frequency. Synthetic availability was calculated by the means of RDKit.

4.1.3 ED 1.5

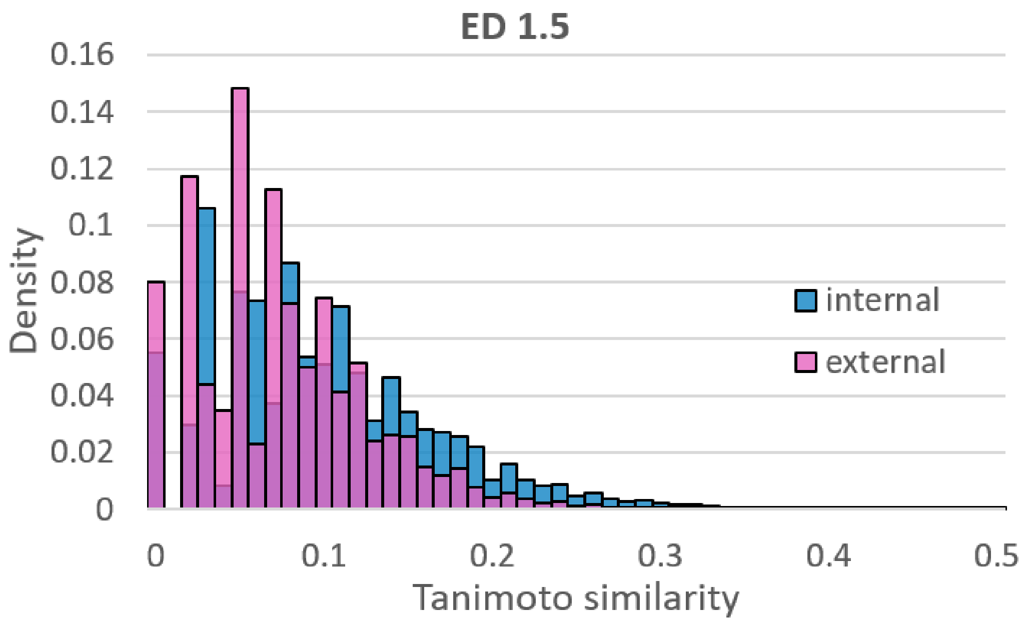

Supplementary Figure 250: Diversity study of the molecules from *ED 1.5*. Internal similarity is where molecules are compared with each other, while external is where molecules are compared with the training set.

**rank**	**molecule**	**occurrences**	**novel**	**commercially available**	**synthetic availability**
**1.**	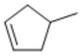	2626	**yes**	no	2.93
**2.**	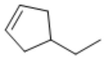	1669	no	no	2.72
**3.**	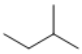	1586	no	**yes**	1.68
**4.**	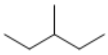	1507	no	**yes**	2.14
**5.**	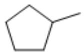	1304	no	**yes**	1.57
**6.**	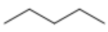	1194	no	**yes**	1.70
**7.**	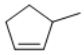	1171	no	no	3.53
**8.**	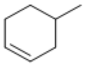	1079	no	**yes**	3.16
**9.**	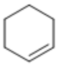	1058	no	**yes**	2.42
**10.**	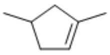	1053	no	no	3.22

Supplementary Table 5: Ten most frequently-occurring valid molecules in the ED 1.5 set, ranked by frequency. availability was calculated by the means of RDKit.

4.1.4 ED 2.0

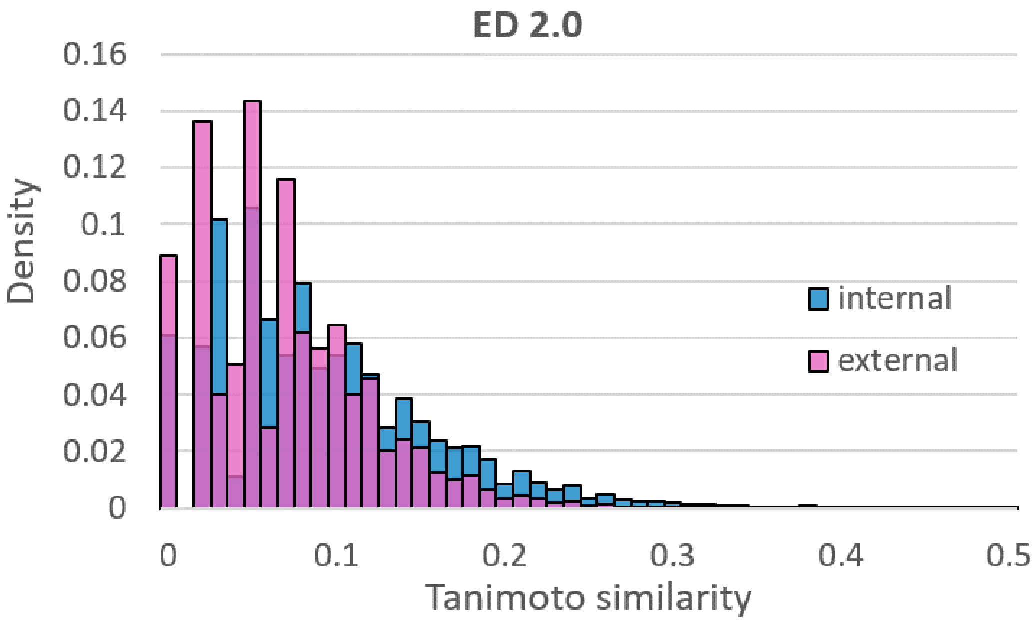

Supplementary Figure 251: Diversity study of the molecules from *ED 2.0*. Internal similarity is where molecules are compared with each other, while external is where molecules are compared with the training set.

rank	molecule	occurrences	novel	commercially available	synthetic availability
**1.**	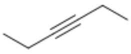	2221	no	**yes**	3.08
**2.**	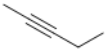	1704	no	**yes**	3.39
**3.**	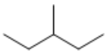	1653	no	**yes**	2.14
**4.**	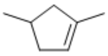	1523	**yes**	**yes**	3.22
**5.**	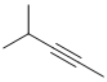	1382	no	**yes**	3.35
**6.**	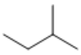	1230	no	**yes**	1.68
**7.**	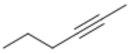	1160	no	**yes**	2.95
**8.**	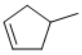	941	**yes**	no	2.93
**9.**	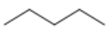	923	no	**yes**	1.70
**10.**	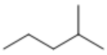	915	no	**yes**	1.89

Supplementary Table 6: Ten most frequently-occurring valid molecules in the ED 2.0 set, ranked by frequency. Synthetic availability was calculated by the means of RDKit.

4.1.5 ED 2.5

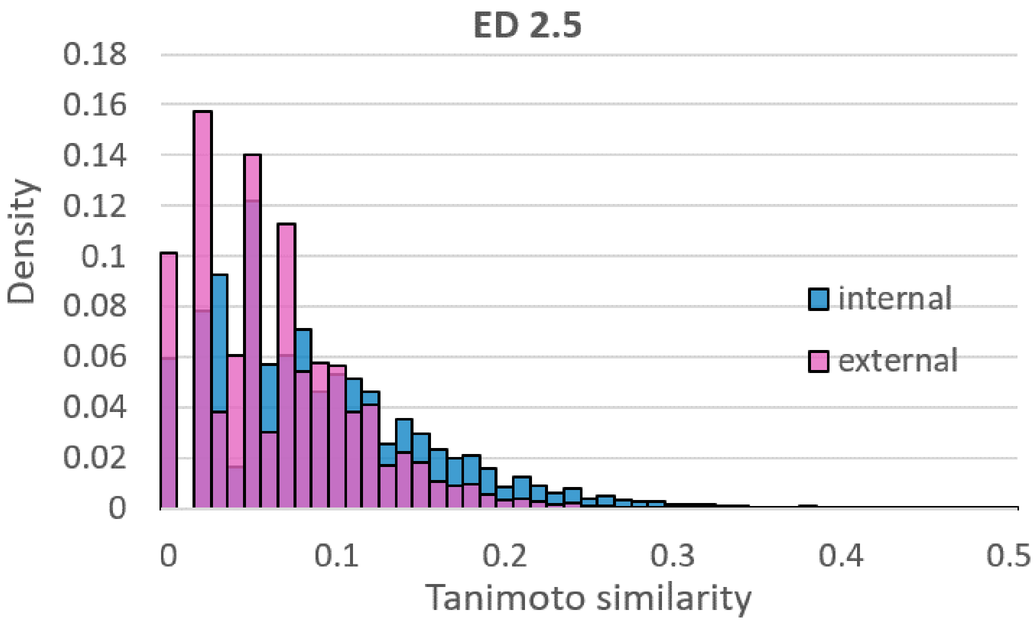

Supplementary Figure 252: Diversity study of the molecules from *ED 2.5*. Internal similarity is where molecules are compared with each other, while external is where molecules are compared with the training set.

**rank**	**molecule**	**occurrences**	**novel**	**commercially available**	**synthetic availability**
**1.**	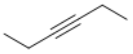	2672	no	**yes**	3.08
**2.**	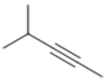	2654	no	**yes**	3.35
**3.**	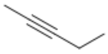	2650	no	**yes**	3.39
**4.**	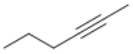	1787	no	**yes**	2.95
**5.**	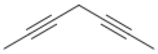	1423	no	no	3.92
**6.**	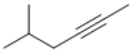	1305	no	no	2.89
**7.**	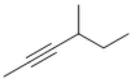	1262	no	no	3.72
**8.**	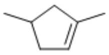	1135	no	no	3.22
**9.**	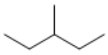	841	no	**yes**	2.14
**10.**	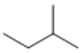	709	no	**yes**	1.68

Supplementary Table 7: Ten most frequently-occurring valid molecules in the ED 2.5 set, ranked by frequency. Synthetic availability was calculated by the means of RDKit.

4.1.6 ED 5.0

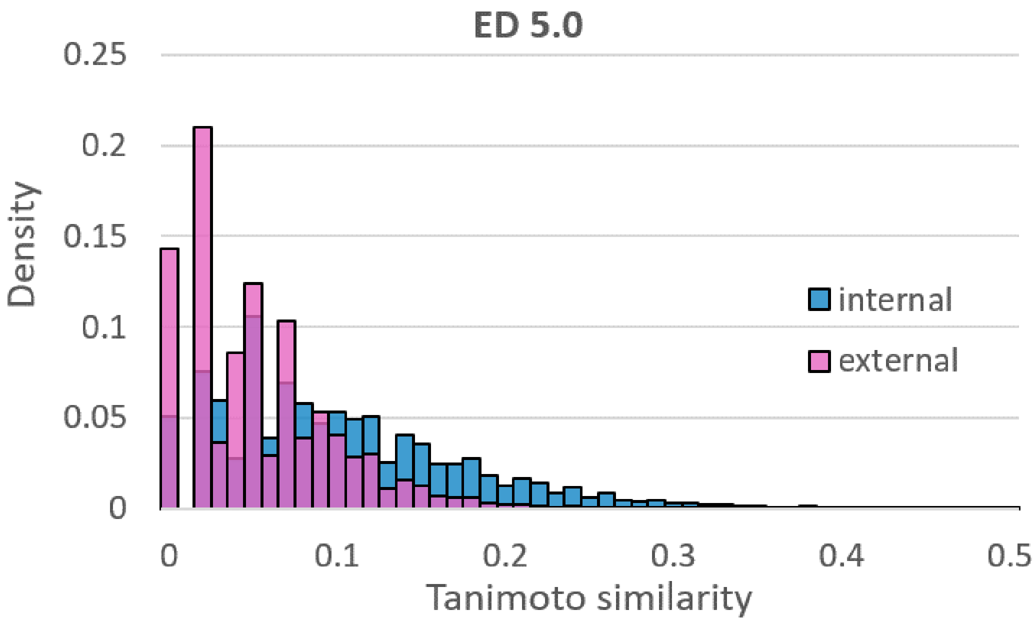

Supplementary Figure 253: Diversity study of the molecules from *ED 5.0*. Internal similarity is where molecules are compared with each other, while external is where molecules are compared with the training set.

rank	molecule	occurrences	novel	commercially available	synthetic availability
**1.**	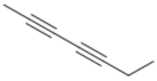	4089	no	no	3.71
**2.**		2981	no	**yes**	3.84
**3.**		2752	no	no	3.99
**4.**	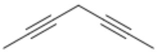	2438	no	no	3.92
**5.**	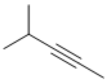	1765	no	**yes**	3.35
**6.**	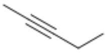	1317	no	**yes**	3.39
**7.**	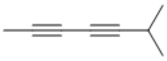	1081	**yes**	no	3.71
**8.**	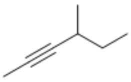	928	no	no	3.72
**9.**	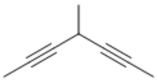	764	no	no	4.30
**10.**	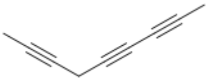	726	no	no	4.13

Supplementary Table 8: Ten most frequently-occurring valid molecules in the ED 5.0 set, ranked by frequency. Synthetic availability was calculated by the means of RDKit.

4.1.7 ED 10.0

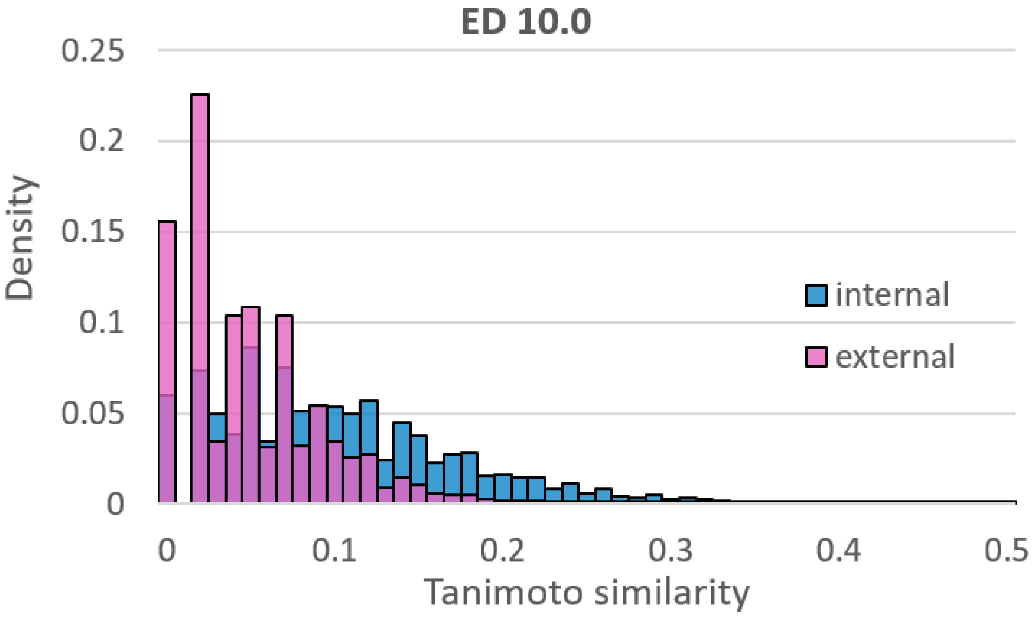

Supplementary Figure 254: Diversity study of the molecules from *ED 10.0*. Internal similarity is where molecules are compared with each other, while external is where molecules are compared with the training set.

**rank**	**molecule**	**occurrences**	**novel**	**commercially available**	**synthetic availability**
**1.**		5769	no	no	3.99
**2.**		3198	no	**yes**	3.84
**3.**	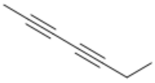	2464	no	no	3.71
**4.**		1842	no	no	4.07
**5.**	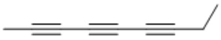	1670	no	no	3.88
**6.**	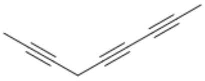	847	no	no	4.13
**7.**		834	no	no	4.16
**8.**	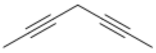	764	no	no	3.92
**9.**		703	no	no	4.25
**10.**		682	no	no	4.20

Supplementary Table 9: Ten most frequently-occurring valid molecules in the ED 10.0 set, ranked by frequency. Synthetic availability was calculated by the means of RDKit.

4.1.8 ED 25.0

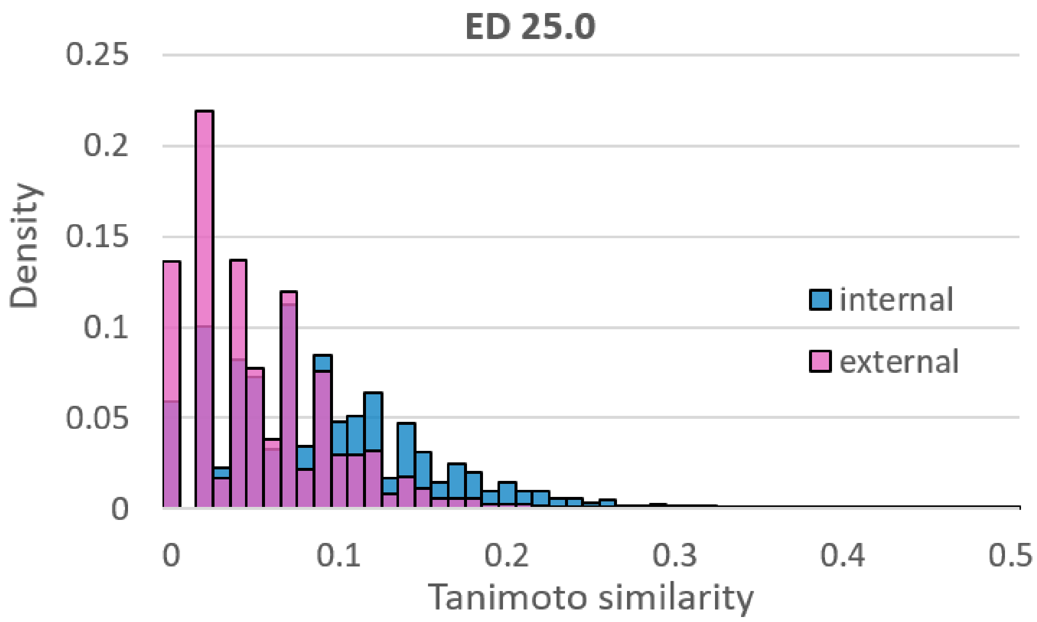

Supplementary Figure 255: Diversity study of the molecules from *ED 25.0*. Internal similarity is where molecules are compared with each other, while external is where molecules are compared with the training set.

rank	molecule	occurrences	novel	commercially available	synthetic availability
**1.**		2115	no	no	3.99
**2.**	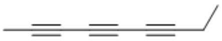	1143	no	no	3.88
**3.**		995	no	yes	3.84
**4.**		734	no	no	4.07
**5.**		670	no	no	4.16
**6.**	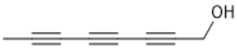	604	no	no	3.84
**7.**	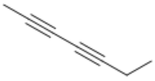	600	no	no	3.71
**8.**	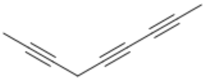	599	no	no	4.13
**9.**		490	no	no	4.25
**10.**	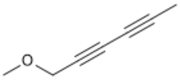	364	no	no	3.55

Supplementary Table 10: Ten most frequently-occurring valid molecules in the ED 25.0 set, ranked by frequency. Synthetic availability was calculated by the means of RDKit.

4.1.9 ED 50.0

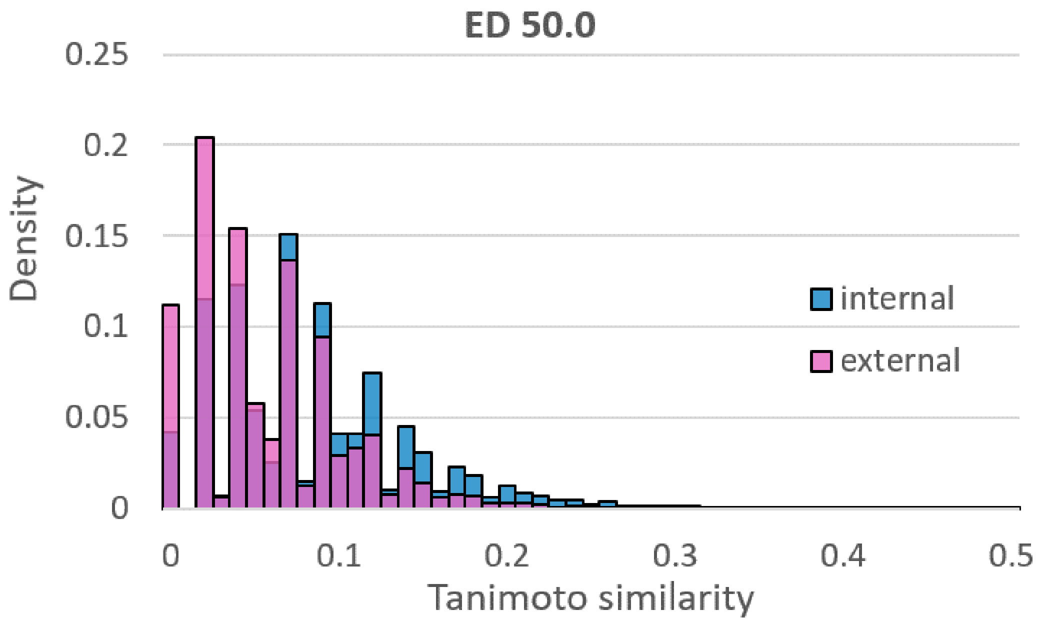

Supplementary Figure 256: Diversity study of the molecules from *ED 50.0*. Internal similarity is where molecules are compared with each other, while external is where molecules are compared with the training set.

rank	molecule	occurrences	novel	commercially available	synthetic availability
**1.**	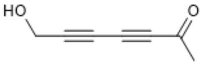	395	no	no	3.59
**2.**	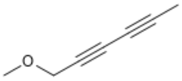	301	no	no	3.55
**3.**	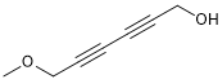	253	no	no	3.46
**4.**	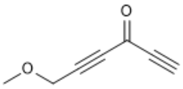	234	no	no	4.03
**5.**	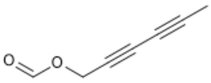	202	no	no	4.08
**6.**	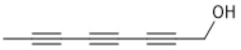	199	no	no	3.84
**7.**	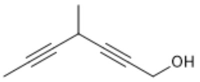	196	no	no	4.54
**8.**		188	no	no	3.99
**9.**	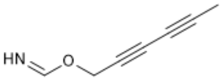	184	**yes**	no	4.48
**10.**	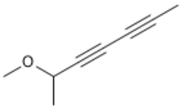	165	no	no	4.30

Supplementary Table 11: Ten most frequently-occurring valid molecules in the ED 50.0 set, ranked by frequency. Synthetic availability was calculated by the means of RDKit.

Supplementary Subsection 4.2 Electron densities decorated with Electrostatic Potentials (ED ESP)

Here is the analysis of thr augmented method, **ED ESP**. Its results comprise nine datasets of 40,000 SMILES each, together representing an STD range from **0.5** to 50.0.

ED ESP	unique		valid	
std	count	fraction	count	fraction
**0.5**	685	0.02	636	<0.01
**1.0**	2509	0.06	2357	0.02
**1.5**	5774	0.14	5459	0.04
**2.0**	8877	0.22	8412	0.06
**2.5**	10712	0.27	10220	0.06
**5.0**	7576	0.19	6996	0.05
**10.0**	5314	0.13	4679	0.07
**25.0**	7772	0.19	6977	0.18
**50.0**	9352	0.23	8607	0.28

Supplementary Table 12: Number and fraction of unique and unique valid molecules in datasets generated by the means of ED ESP method. Each dataset corresponds to a different RNG StD and includes a total of non-unique 40,000 SMILES.

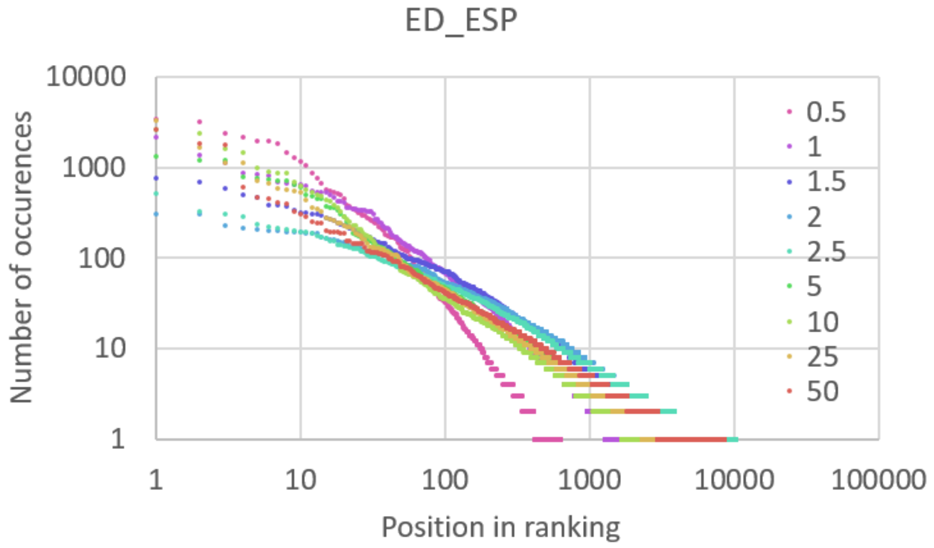

Supplementary Figure 257: Frequency of occurrence of specific valid SMILES in produced results. The plot ranks SMILES according to the number of times they appear in sets of 40,000 generated SMILES. Invalid SMILES are omitted.

ED ESP std	total	passes MolGen filters		novel vs training set		novel vs test set		commercially available	
		count	fraction	count	fraction	count	fraction	count	fraction
**0.5**	636	610	0.96	146	0.64	216	0.94	68	0.30
**1.0**	2357	2247	0.95	528	0.56	876	0.94	240	0.26
**1.5**	5459	5200	0.95	1014	0.59	1633	0.94	418	0.24
**2.0**	8412	8096	0.96	1288	0.57	2158	0.95	496	0.22
**2.5**	10220	9936	0.97	1299	0.56	2208	0.95	493	0.21
**5.0**	6996	6954	0.99	1062	0.52	1957	0.96	273	0.13
**10.0**	4679	4644	0.99	1471	0.56	2510	0.96	222	0.08
**25.0**	6977	6933	0.99	4364	0.61	6857	0.96	235	0.03
**50.0**	8607	8558	0.99	6649	0.60	10574	0.96	262	0.02

Supplementary Table 13: Number and fraction of chemically possible and novel molecules in each set of unique valid SMILES corresponding to specific RNG StD used by the ED ESP method.

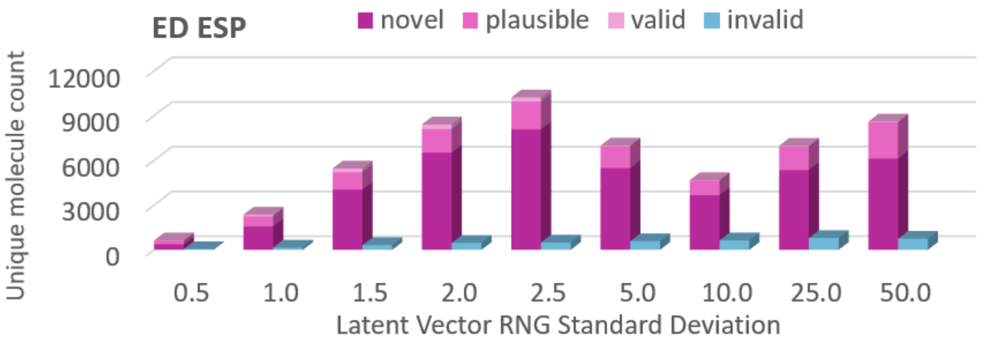

Supplementary Figure 258: Total counts of valid and invalid unique molecules. Total height of pink column represents the unique valid SMILES count, where valid molecules may be chemically plausible and/or novel (as shown by different shades of pink that overlap each other from the bottom-up).

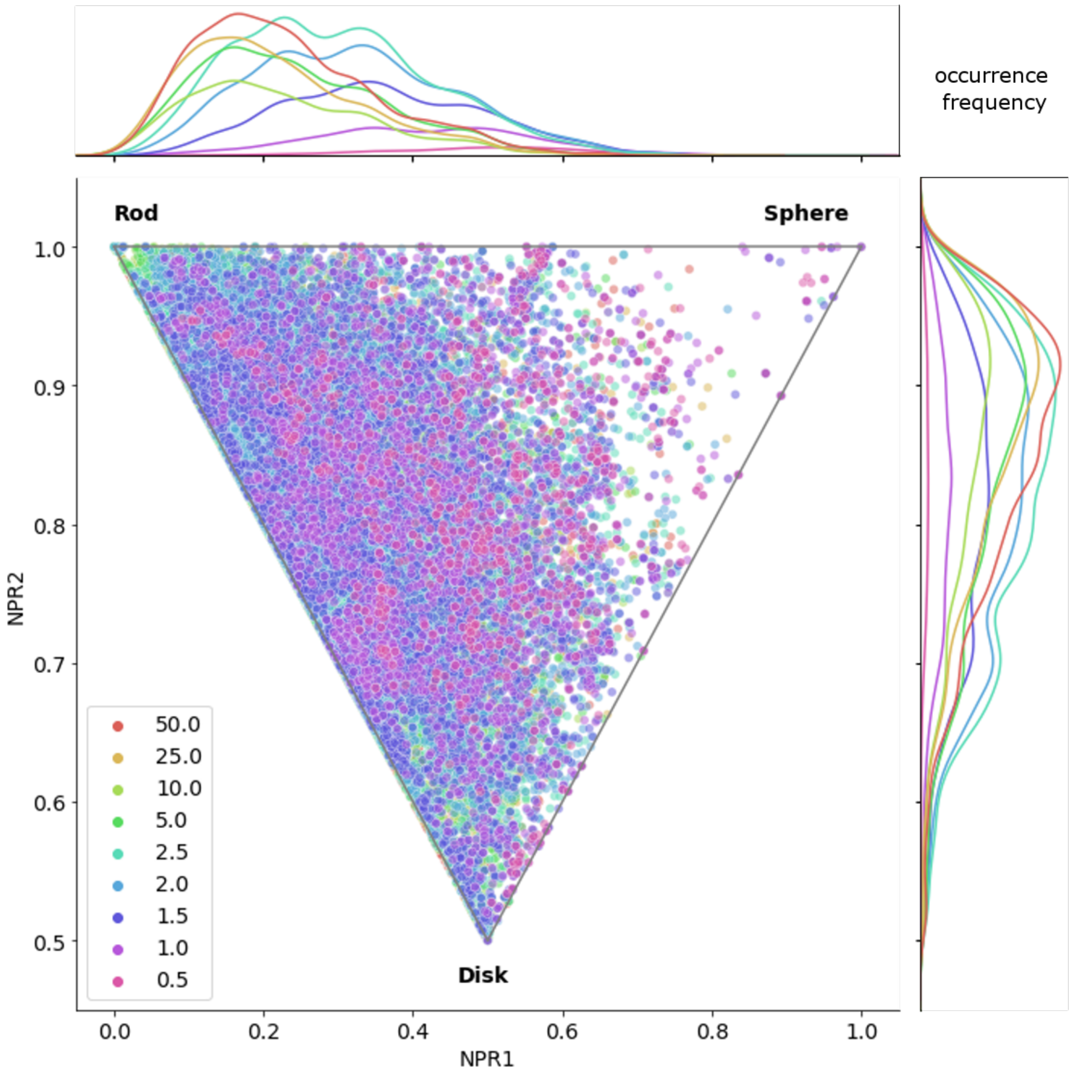

Supplementary Figure 259: Shapes of molecules from the ED ESP sets according to RDKit NPR calculations. Each dot represents a different unique SMILES and the colors correspond to different RNG StD sets. For clarity purposes, plots have been added to the sides to show the spatial distributions of colored dots. Only those SMILES which geometry could be successfully determined by RDKit and/or OpenBabel tools were included in the plot.

4.2.1 ED ESP 0.5

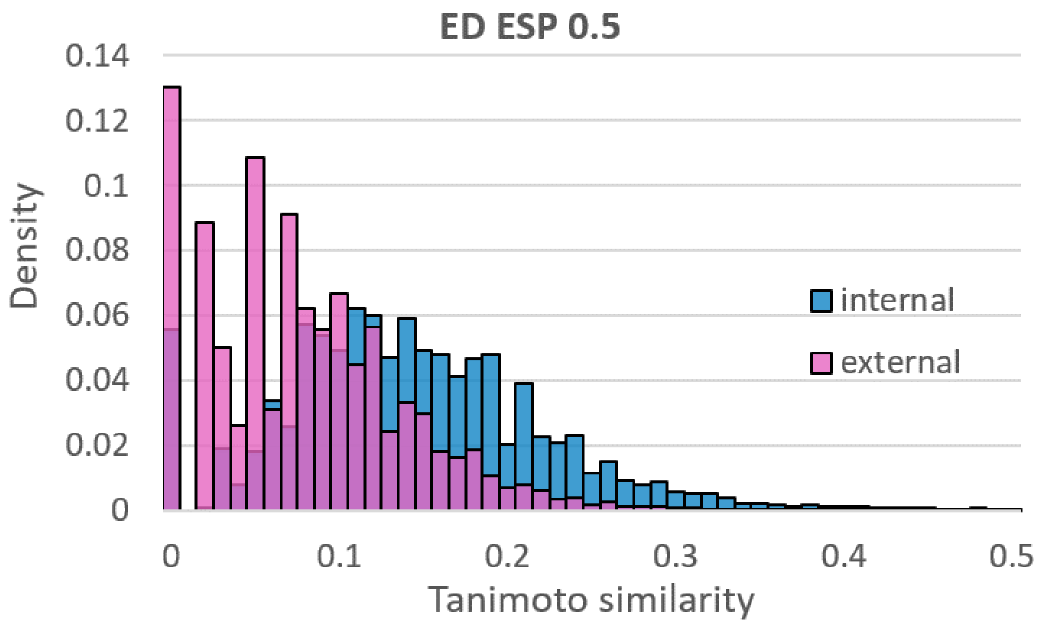

Supplementary Figure 260: Diversity study of the molecules from *ED ESP 0.5*. Internal similarity is where molecules are compared with each other, while external is where molecules are compared with the training set.

rank	molecule	occurrences	novel	commercially available	synthetic availability
**1.**	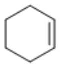	3394	no	**yes**	2.42
**2.**	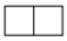	3181	no	no	1.90
**3.**	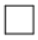	2378	no	no	1.00
**4.**	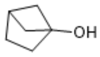	2137	no	no	3.92
**5.**	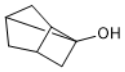	1963	no	no	5.69
**6.**	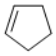	1933	no	**yes**	2.73
**7.**	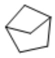	1855	no	no	2.80
**8.**	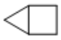	1450	no	no	2.78
**9.**	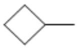	1301	**yes**	no	1.42
**10.**	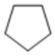	1156	no	**yes**	1.00

Supplementary Table 14: Ten most frequently-occurring valid molecules in the ED ESP 0.5 set, ranked by frequency. Synthetic availability was calculated by the means of RDKit.

4.2.2 ED ESP 1.0

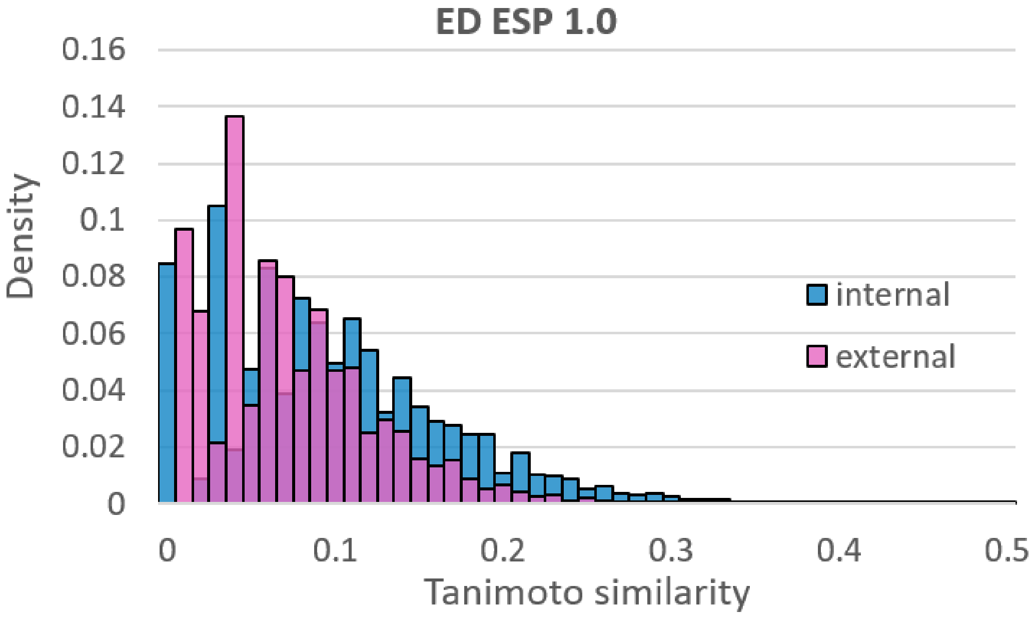

Supplementary Figure 261: Diversity study of the molecules from *ED ESP 1.0*. Internal similarity is where molecules are compared with each other, while external is where molecules are compared with the training set.

rank	molecule	occurrences	novel	commercially available	synthetic availability
**1.**	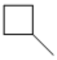	2166	**yes**	no	1.42
**2.**	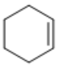	1369	no	**yes**	2.42
**3.**	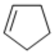	1134	no	**yes**	2.73
**4.**	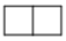	861	no	no	1.90
**5.**	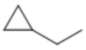	848	no	no	1.70
**6.**	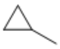	814	**yes**	no	1.37
**7.**	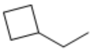	695	no	no	1.70
**8.**	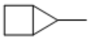	670	no	no	3.24
**9.**	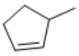	656	no	no	3.53
**10.**	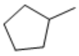	651	no	**yes**	1.57

Supplementary Table 15: Ten most frequently-occurring valid molecules in the ED ESP 1.0 set, ranked by frequency. Synthetic availability was calculated by the means of RDKit.

4.2.3 ED ESP 1.5

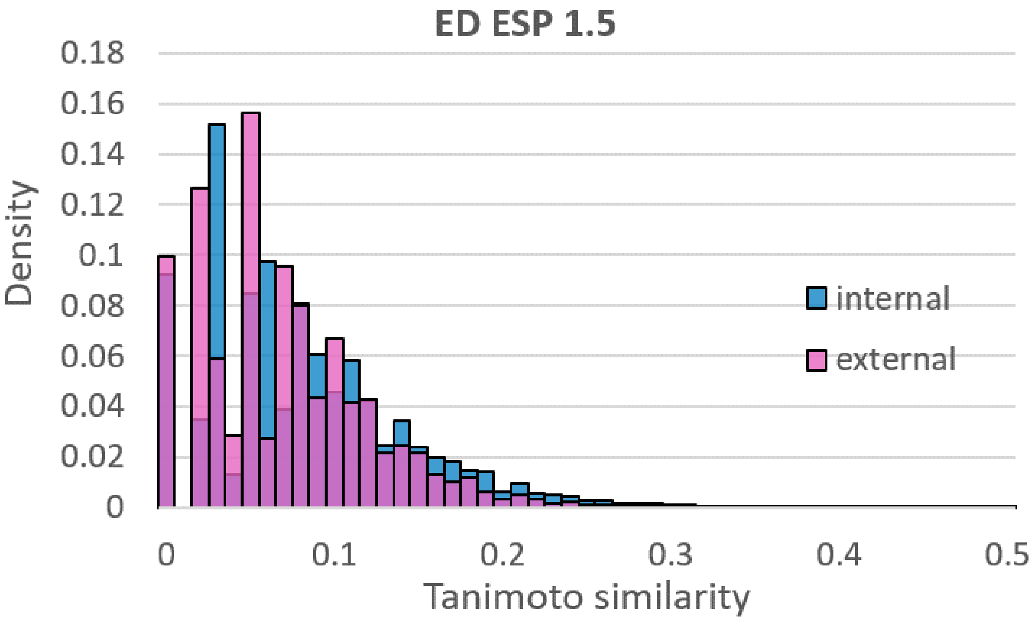

Supplementary Figure 262: Diversity study of the molecules from *ED ESP 1.5*. Internal similarity is where molecules are compared with each other, while external is where molecules are compared with the training set.

rank	molecule	occurrences	novel	commercially available	synthetic availability
**1.**	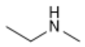	762	**yes**	**yes**	2.31
**2.**	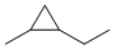	684	no	no	3.06
**3.**	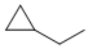	592	no	no	1.70
**4.**	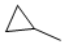	496	**yes**	no	1.37
**5.**	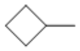	461	**yes**	no	1.42
**6.**	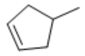	388	**yes**	no	2.93
**7.**	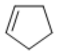	379	no	**yes**	2.73
**8.**	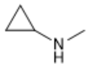	366	**yes**	**yes**	2.10
**9.**	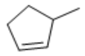	335	no	no	3.53
**10.**	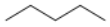	319	no	**yes**	1.70

Supplementary Table 16: Ten most frequently-occurring valid molecules in the ED ESP 1.5 set, ranked by frequency. Synthetic availability was calculated by the means of RDKit.

4.2.4 ED ESP 2.0

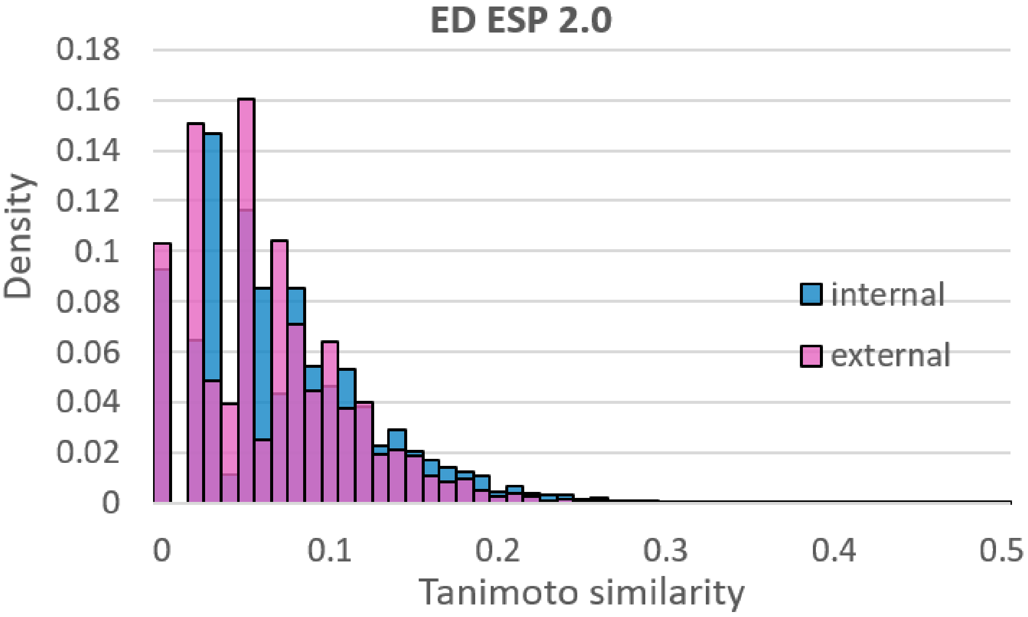

Supplementary Figure 263: Diversity study of the molecules from *ED ESP 2.0*. Internal similarity is where molecules are compared with each other, while external is where molecules are compared with the training set.

rank	molecule	occurrences	novel	commercially available	synthetic availability
**1.**	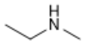	308	**yes**	**yes**	2.31
**2.**	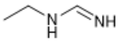	302	**yes**	no	4.32
**3.**	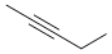	231	no	**yes**	3.39
**4.**	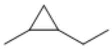	216	no	no	3.06
**5.**	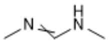	205	**yes**	no	4.34
**6.**	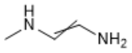	200	**yes**	no	4.42
**7.**	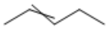	199	**yes**	**yes**	2.88
**8.**	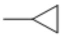	193	**yes**	no	1.37
**9.**	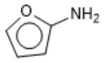	193	no	no	3.05
**10.**	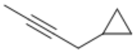	191	no	no	2.99

Supplementary Table 17: Ten most frequently-occurring valid molecules in the ED ESP 2.0 set, ranked by frequency. Synthetic availability was calculated by the means of RDKit.

4.2.5 ED ESP 2.5

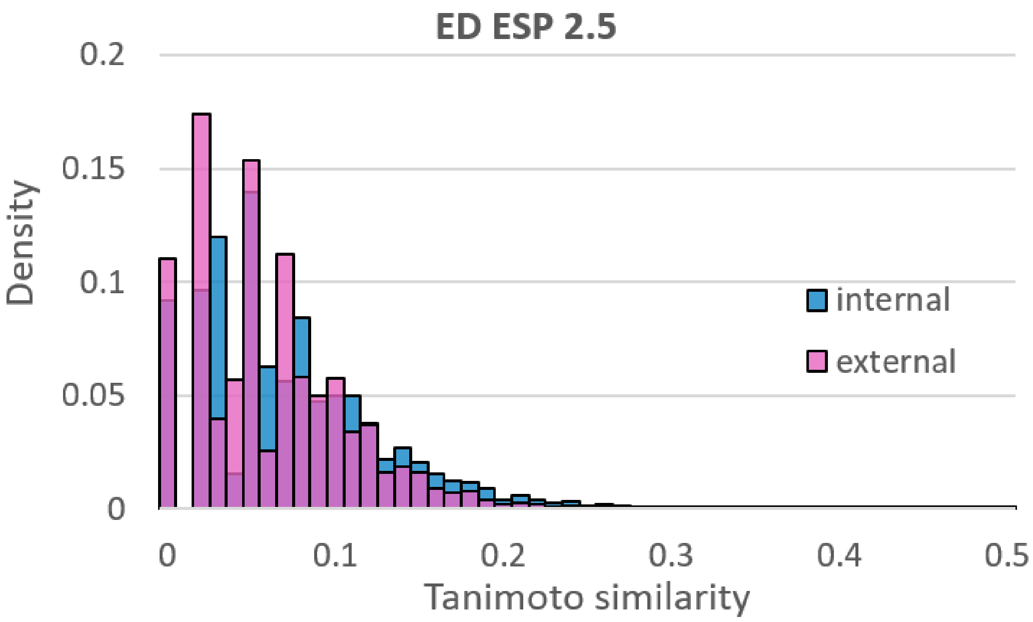

Supplementary Figure 264: Diversity study of the molecules from *ED ESP 2.5*. Internal similarity is where molecules are compared with each other, while external is where molecules are compared with the training set.

**rank**	**molecule**	**occurrences**	**novel**	**commercially available**	**synthetic availability**
**1.**	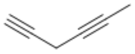	517	**yes**	no	4.10
**2.**	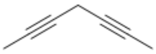	326	no	no	3.92
**3.**	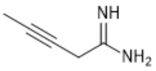	305	**yes**	no	3.88
**4.**	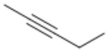	286	no	**yes**	3.39
**5.**	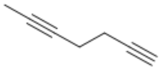	235	no	no	3.62
**6.**	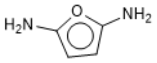	217	no	no	3.43
**7.**	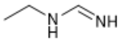	216	**yes**	no	4.32
**8.**	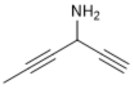	205	**yes**	no	4.99
**9.**	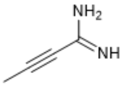	198	**yes**	no	4.28
**10.**	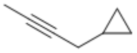	193	no	no	2.99

Supplementary Table 18: Ten most frequently-occurring valid molecules in the ED ESP 2.5 set, ranked by frequency. Synthetic availability was calculated by the means of RDKit.

4.2.6 ED ESP 5.0

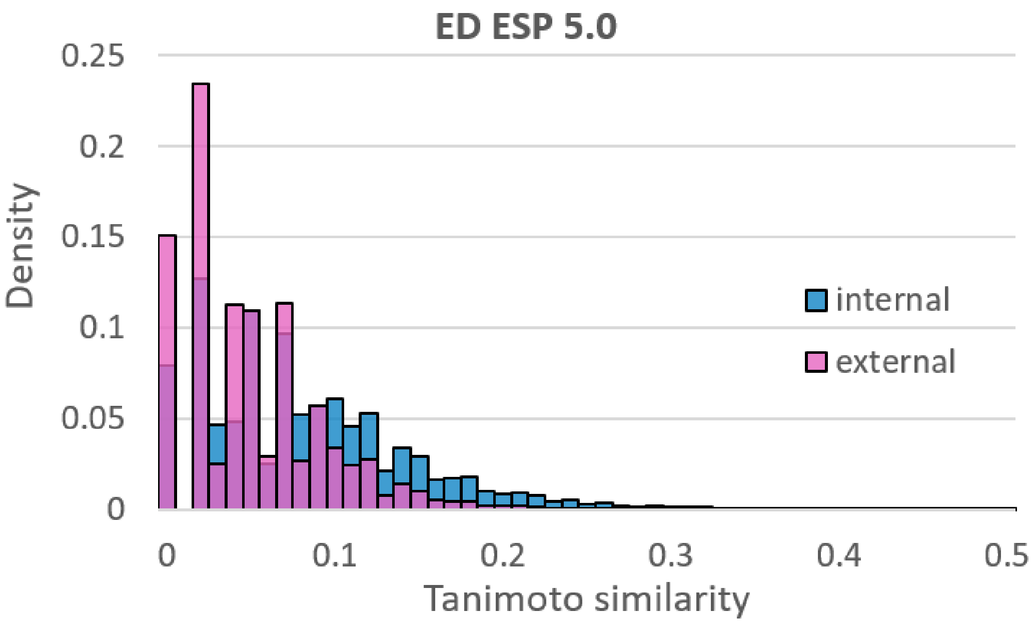

Supplementary Figure 265: Diversity study of the molecules from *ED ESP 5.0*. Internal similarity is where molecules are compared with each other, while external is where molecules are compared with the training set.

rank	molecule	occurrences	novel	commercially available	synthetic availability
**1.**	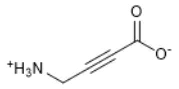	1319	no	no	5.73
**2.**		1208	no	no	4.07
**3.**	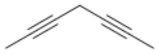	1188	no	no	3.92
**4.**		781	no	no	3.99
**5.**	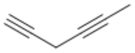	771	**yes**	no	4.10
**6.**		743	no	no	4.20
**7.**	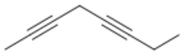	708	no	no	3.73
**8.**	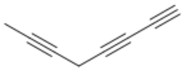	703	no	no	4.27
**9.**	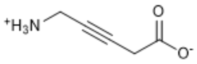	642	no	no	5.49
**10.**	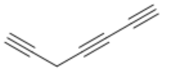	607	no	no	4.46

Supplementary Table 19: Ten most frequently-occurring valid molecules in the ED ESP 5.0 set, ranked by frequency. Synthetic availability was calculated by the means of RDKit.

4.2.7 ED ESP 10.0

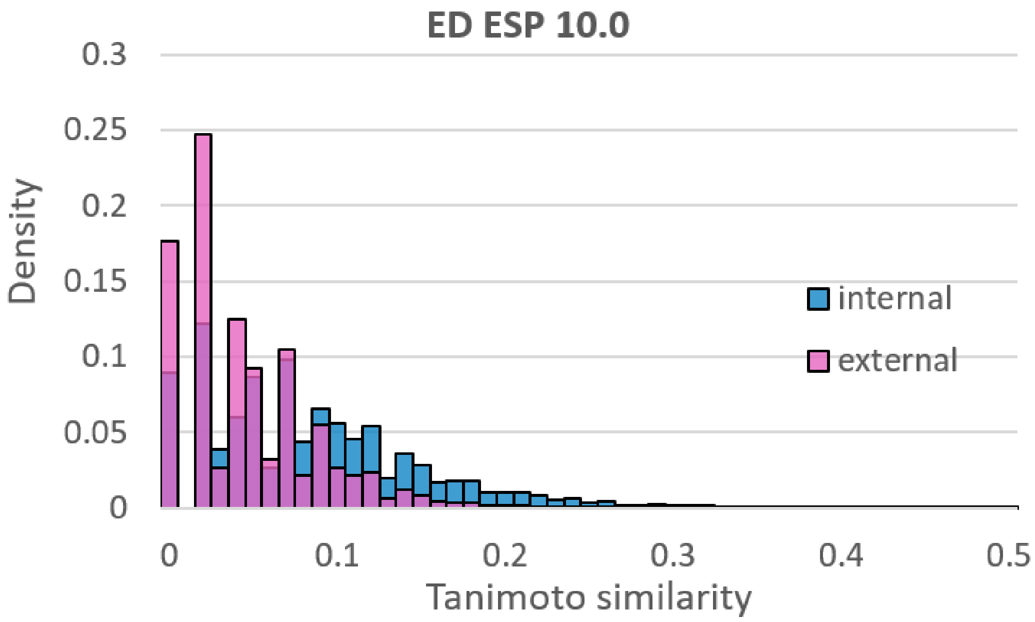

Supplementary Figure 266: Diversity study of the molecules from *ED ESP 10.0*. Internal similarity is where molecules are compared with each other, while external is where molecules are compared with the training set.

rank	molecule	occurrences	novel	commercially available	synthetic availability
**1.**		2645	no	no	3.99
**2.**	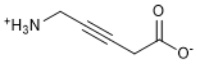	2412	no	no	5.49
**3.**	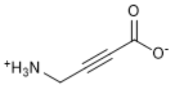	1612	no	no	5.73
**4.**		1441	no	no	4.07
**5.**	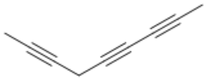	980	no	no	4.13
**6.**		889	no	no	4.20
**7.**	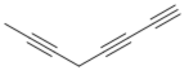	871	no	no	4.27
**8.**	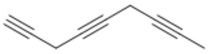	867	no	no	4.23
**9.**	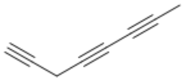	683	**yes**	no	4.27
**10.**	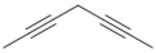	633	no	no	3.92

Supplementary Table 20: Ten most frequently-occurring valid molecules in the ED ESP 10.0 set, ranked by frequency. Synthetic availability was calculated by the means of RDKit.

4.2.8 ED ESP 25.0

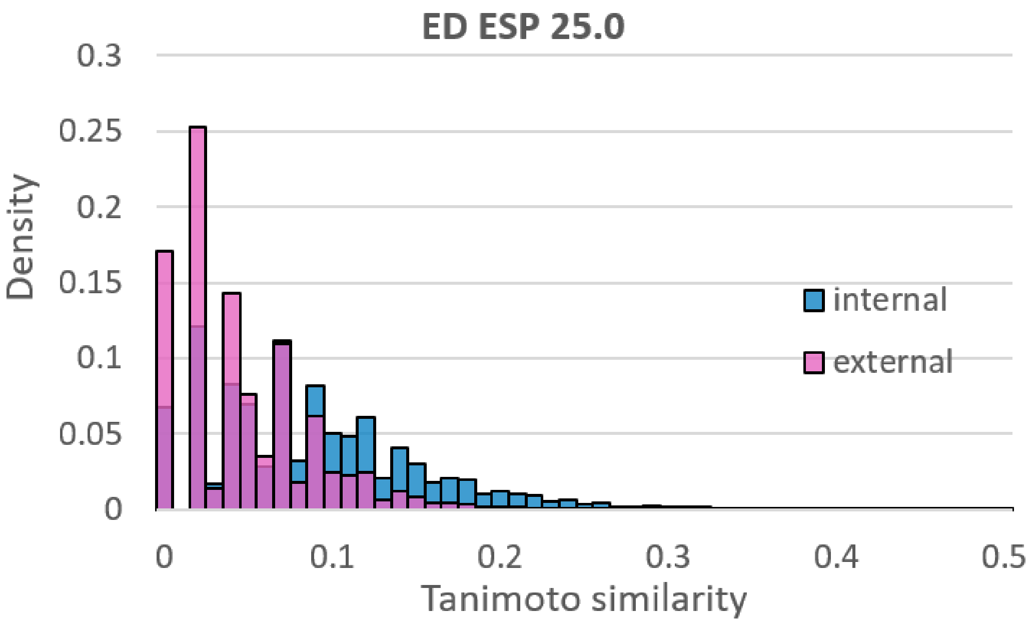

Supplementary Figure 267: Diversity study of the molecules from *ED ESP 25.0*. Internal similarity is where molecules are compared with each other, while external is where molecules are compared with the training set.

rank	molecule	occurrences	novel	commercially available	synthetic availability
**1.**	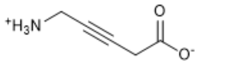	3331	no	no	5.49
**2.**		1649	no	no	3.99
**3.**	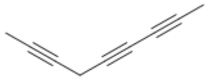	1129	no	no	4.13
**4.**	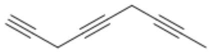	1111	no	no	4.23
**5.**	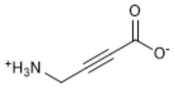	714	no	no	5.73
**6.**		672	no	no	4.07
**7.**	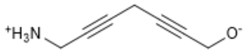	594	**yes**	no	5.64
**8.**	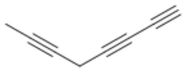	560	no	no	4.27
**9.**	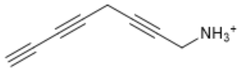	547	**yes**	no	5.24
**10.**	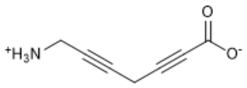	539	**yes**	no	5.42

Supplementary Table 21: Ten most frequently-occurring valid molecules in the ED ESP 25.0 set, ranked by frequency. Synthetic availability was calculated by the means of RDKit.

4.2.9 ED ESP 50.0

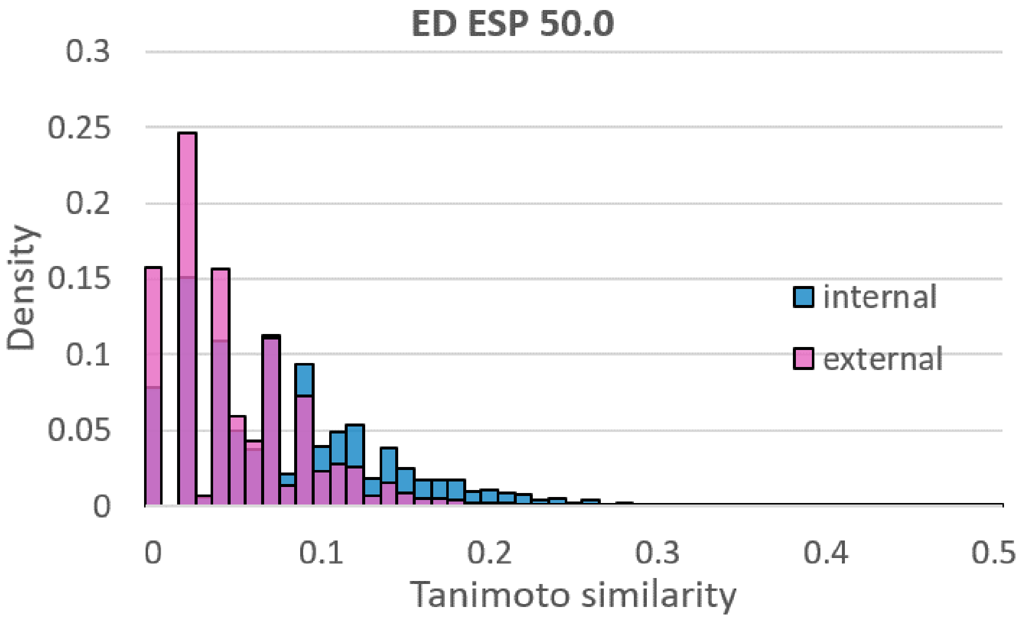

Supplementary Figure 268: Diversity study of the molecules from *ED ESP 50.0*. Internal similarity is where molecules are compared with each other, while external is where molecules are compared with the training set.

rank	molecule	occurrences	novel	commercially available	synthetic availability
**1.**	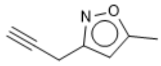	2625	no	no	3.02
**2.**	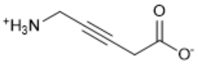	1813	no	no	5.49
**3.**	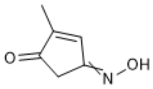	1773	no	no	3.67
**4.**	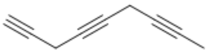	607	no	no	4.23
**5.**	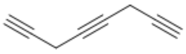	461	no	no	4.39
**6.**	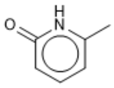	444	no	**yes**	2.28
**7.**	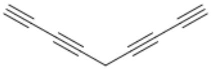	412	no	no	4.53
**8.**	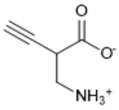	401	no	no	5.97
**9.**	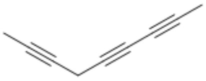	322	no	no	4.13
**10.**	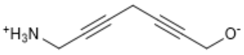	302	**yes**	no	5.64

Supplementary Table 22: Ten most frequently-occurring valid molecules in the ED ESP 50.0 set, ranked by frequency. Synthetic availability was calculated by the means of RDKit.

Supplementary Subsection 4.3 Electron densities (ED) Probabilistic Samplig (proba10)

Here is the analysis of **ED proba10**. Its results comprise eight datasets of 40,000 SMILES each, together representing an STD range from **0.5** to **25.0**.

ED proba10	unique		valid	
std	count	fraction	count	fraction
**0.5**	250	<0.01	223	<0.01
**1.0**	1043	0.03	948	0.02
**1.5**	1888	0.05	1731	0.04
**2.0**	2464	0.06	2233	0.06
**2.5**	2704	0.07	2412	0.06
**5.0**	2255	0.06	2028	0.05
**10.0**	2997	0.07	2620	0.07
**25.0**	8579	0.21	7160	0.18
**50.0**	-	-	-	-

Supplementary Table 23: Number and fraction of unique and unique valid molecules in datasets generated by the means of ED proba10 method. Each dataset corresponds to a different RNG StD and includes a total of 40,000 non-unique SMILES.

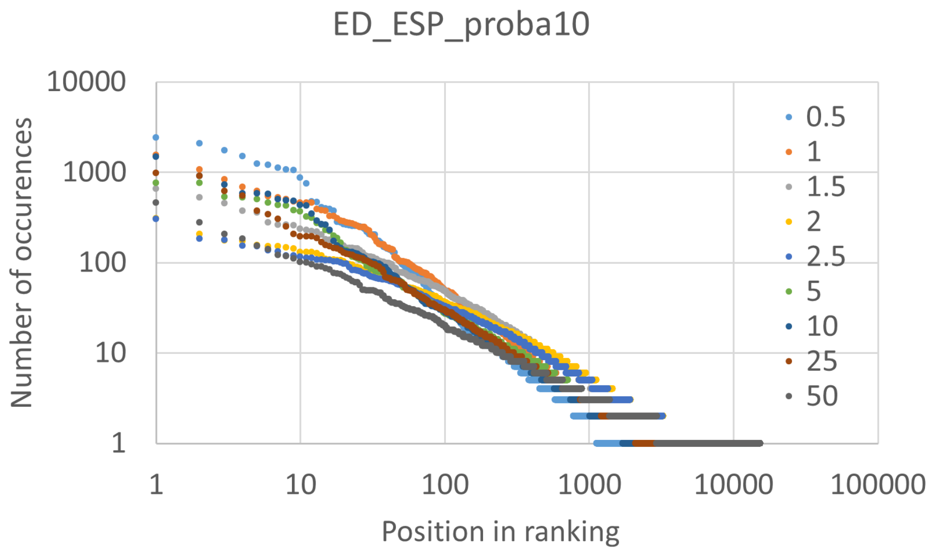

Supplementary Figure 269: Frequency of occurrence of specific valid SMILES in produced results. The plot ranks SMILES according to the number of times they appear in sets of 40,000 generated SMILES. Invalid SMILES are omitted.

ED proba10 std	total	passes MolGen filters count	passes MolGen filters fraction	novel vs training set count	novel vs training set fraction	novel vs test set count	novel vs test set fraction	commercially available count	commercially available fraction
**0.5**	223	220	0.99	139	0.62	210	0.94	69	0.31
**1.0**	948	909	0.96	545	0.57	892	0.94	241	0.25
**1.5**	1731	1640	0.95	1007	0.58	1627	0.94	408	0.24
**2.0**	2233	2139	0.96	1282	0.57	2131	0.95	477	0.21
**2.5**	2412	2343	0.97	1309	0.54	2300	0.95	503	0.21
**5.0**	2028	2010	0.99	1047	0.52	1953	0.96	286	0.14
**10.0**	2620	2584	0.99	1470	0.56	2509	0.96	209	0.08
**25.0**	7160	7059	0.99	4390	0.61	6861	0.96	238	0.03
**50.0**	-	-	-	-	-	-	-	-	-

Supplementary Table 24: Number and fraction of chemically possible and novel molecules in each set of unique valid SMILES corresponding to specific RNG StD used by the ED proba10 method.

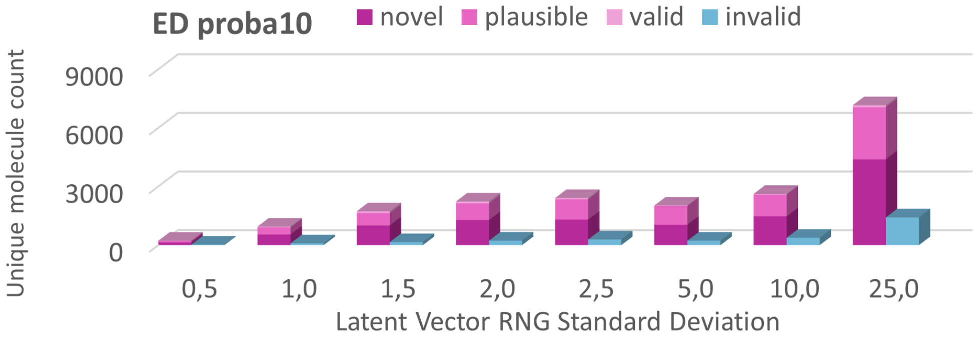

Supplementary Figure 270: Total counts of valid and invalid unique molecules. Total height of pink column represents the unique valid SMILES count, where valid molecules may be chemically plausible and/or novel (as shown by different shades of pink that overlap each other from the bottom-up).

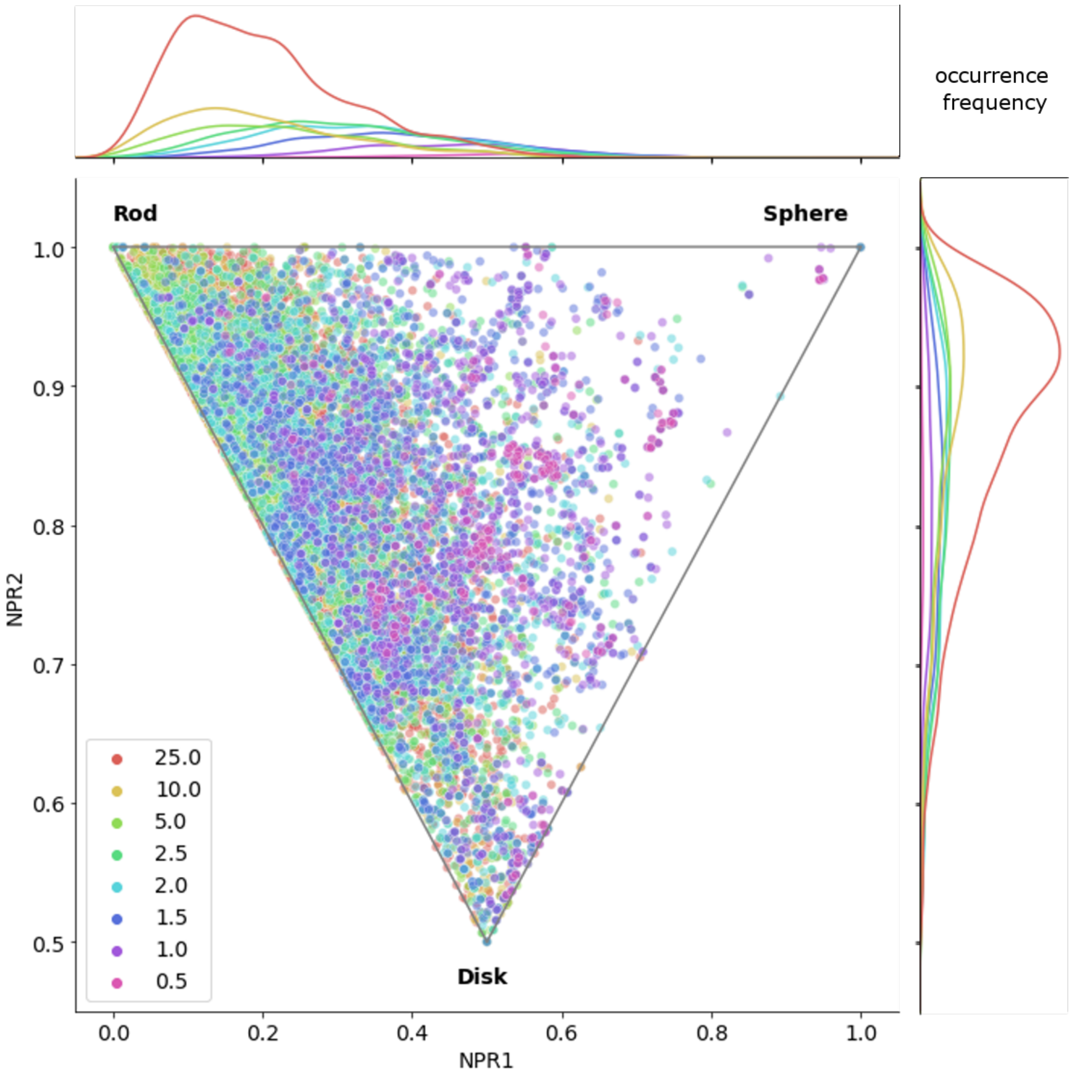

Supplementary Figure 271: Shapes of molecules from the ED proba10 sets according to RDKit NPR calculations. Each dot represents a different unique SMILES and the colors correspond to different RNG StD sets. For clarity purposes, plots have been added to the sides to show the spatial distributions of colored dots. Only those SMILES which geometry could be successfully determined by RDKit and/or OpenBabel tools were included in the plot.

4.3.1 ED proba10 0.5

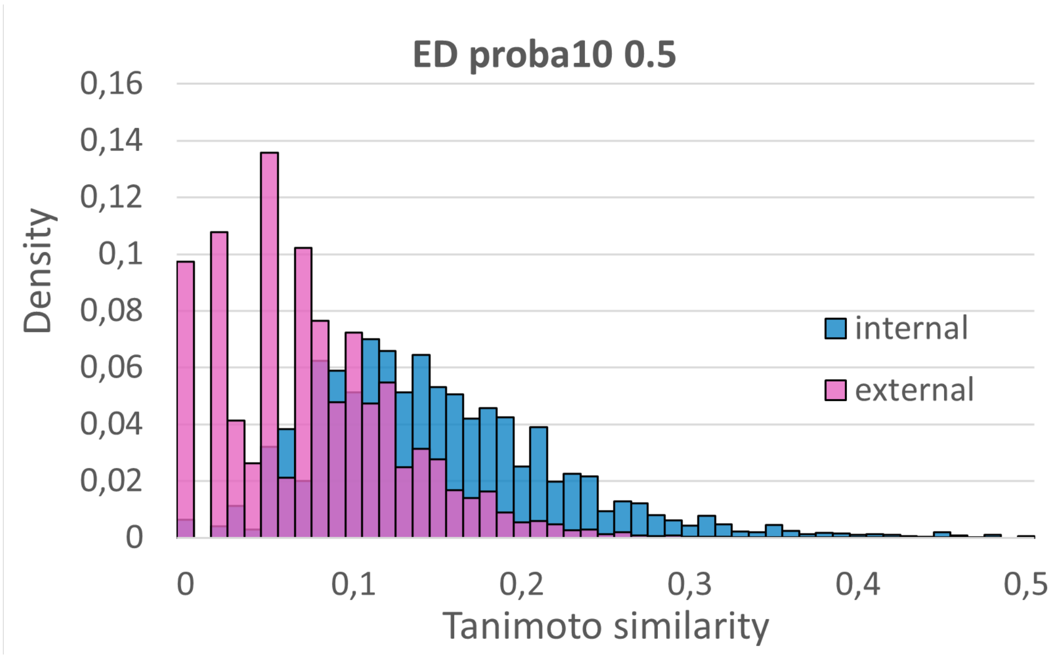

Supplementary Figure 272: Diversity study of the molecules from *ED proba10 0.5*. Internal similarity is where molecules are compared with each other, while external is where molecules are compared with the training set.

rank	molecule	occurrences	novel	commercially available	synthetic availability
**1.**	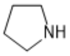	9901	**yes**	**yes**	2.19
**2.**	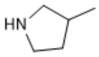	8154	**yes**	**yes**	3.04
**3.**	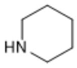	2337	**yes**	**yes**	2.06
**4.**	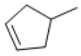	1950	**yes**	no	2.93
**5.**	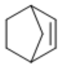	1661	no	**yes**	4.62
**6.**	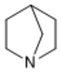	1634	**yes**	no	3.29
**7.**	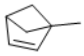	1470	no	no	4.39
**8.**	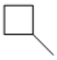	983	**yes**	no	1.42
**9.**	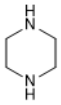	790	**yes**	**yes**	2.70
**10.**	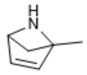	709	**yes**	no	5.84

Supplementary Table 25: Ten most frequently-occurring valid molecules in the ED proba10 0.5 set, ranked by frequency. Synthetic availability was calculated by the means of RDKit.

4.3.2 ED proba10 1.0

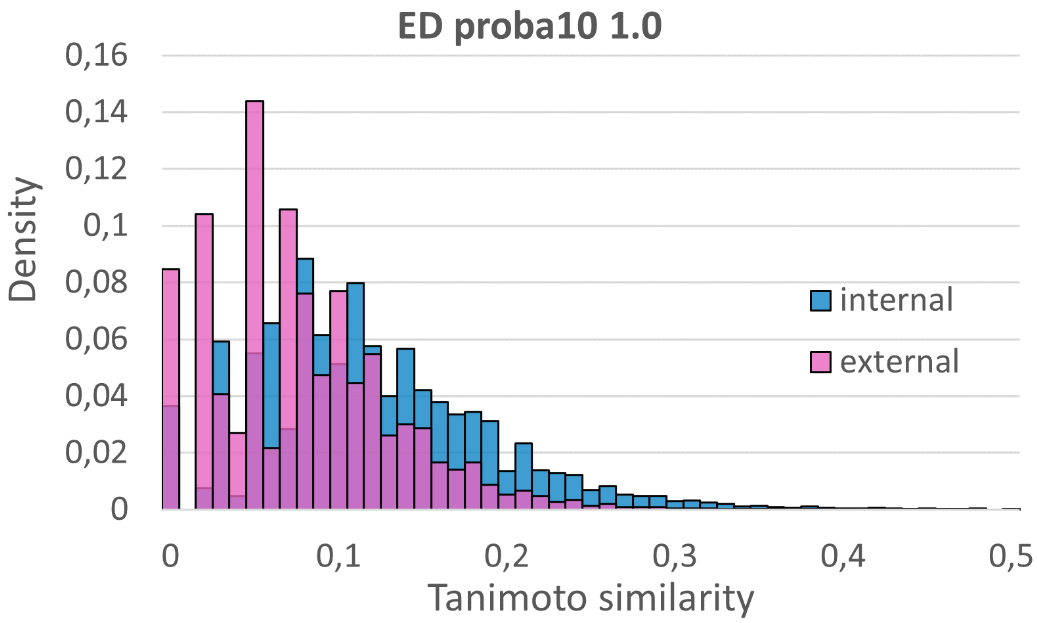

Supplementary Figure 273: Diversity study of the molecules from *ED proba10 1.0*. Internal similarity is where molecules are compared with each other, while external is where molecules are compared with the training set.

rank	molecule	occurrences	novel	commercially available	synthetic availability
**1.**	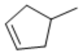	4667	**yes**	no	2.93
**2.**	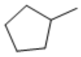	2975	no	**yes**	1.57
**3.**	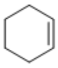	2020	no	**yes**	2.42
**4.**	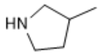	1945	**yes**	**yes**	3.04
**5.**	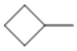	1381	**yes**	no	1.42
**6.**	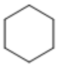	1292	**yes**	**yes**	1.00
**7.**	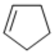	1058	no	**yes**	2.73
**8.**	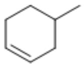	1026	no	**yes**	3.16
**9.**	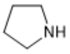	1015	**yes**	**yes**	2.19
**10.**	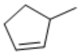	872	no	no	3.53

Supplementary Table 26: Ten most frequently-occurring valid molecules in the ED proba10 1.0 set, ranked by frequency. Synthetic availability was calculated by the means of RDKit.

4.3.3 ED proba10 1.5

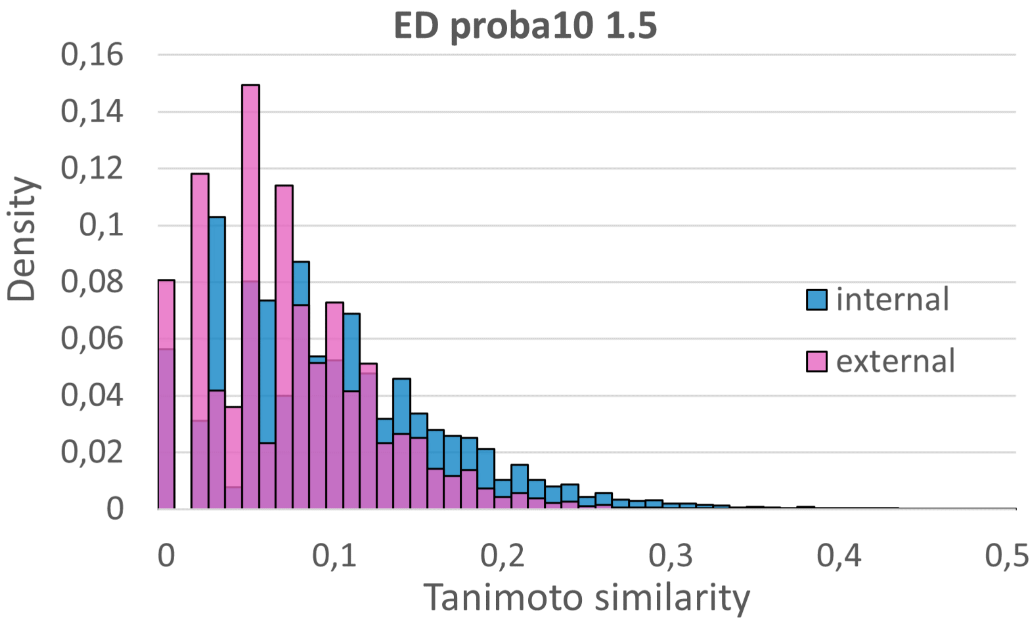

Supplementary Figure 274: Diversity study of the molecules from *ED proba10 1.5*. Internal similarity is where molecules are compared with each other, while external is where molecules are compared with the training set.

**rank**	**molecule**	**occurrences**	**novel**	**commercially available**	**synthetic availability**
**1.**	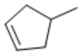	2583	**yes**	no	2.93
**2.**	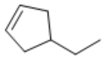	1677	no	no	2.72
**3.**	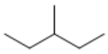	1573	no	**yes**	2.14
**4.**	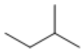	1477	no	**yes**	1.68
**5.**	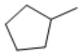	1321	no	**yes**	1.57
**6.**	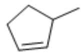	1218	no	no	3.53
**7.**	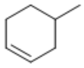	1150	no	**yes**	3.16
**8.**	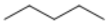	1136	no	**yes**	1.70
**9.**	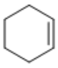	1122	no	**yes**	2.42
**10.**	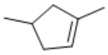	1011	no	no	3.22

Supplementary Table 27: Ten most frequently-occurring valid molecules in the ED proba10 1.5 set, ranked by frequency. Synthetic availability was calculated by the means of RDKit.

4.3.4 ED proba10 2.0

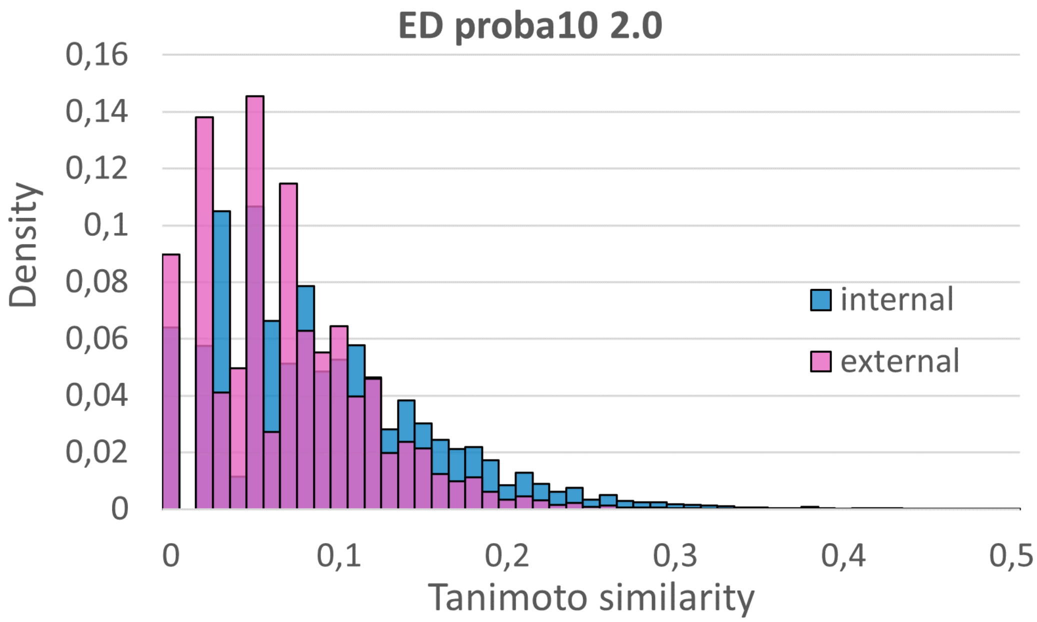

Supplementary Figure 275: Diversity study of the molecules from *ED proba10 2.0*. Internal similarity is where molecules are compared with each other, while external is where molecules are compared with the training set.

rank	molecule	occurrences	novel	commercially available	synthetic availability
**1.**	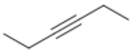	2188	no	**yes**	3.08
**2.**	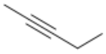	1772	no	**yes**	3.39
**3.**	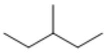	1652	no	**yes**	2.14
**4.**	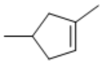	1626	no	no	3.22
**5.**	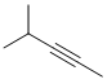	1403	no	**yes**	3.35
**6.**	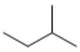	1239	no	**yes**	1.68
**7.**	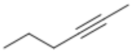	1210	no	**yes**	2.95
**8.**	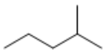	985	no	**yes**	1.89
**9.**	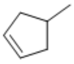	940	**yes**	no	2.93
**10.**	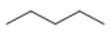	821	no	**yes**	1.70

Supplementary Table 28: Ten most frequently-occurring valid molecules in the ED proba10 2.0 set, ranked by frequency. Synthetic availability was calculated by the means of RDKit.

4.3.5 ED proba10 2.5

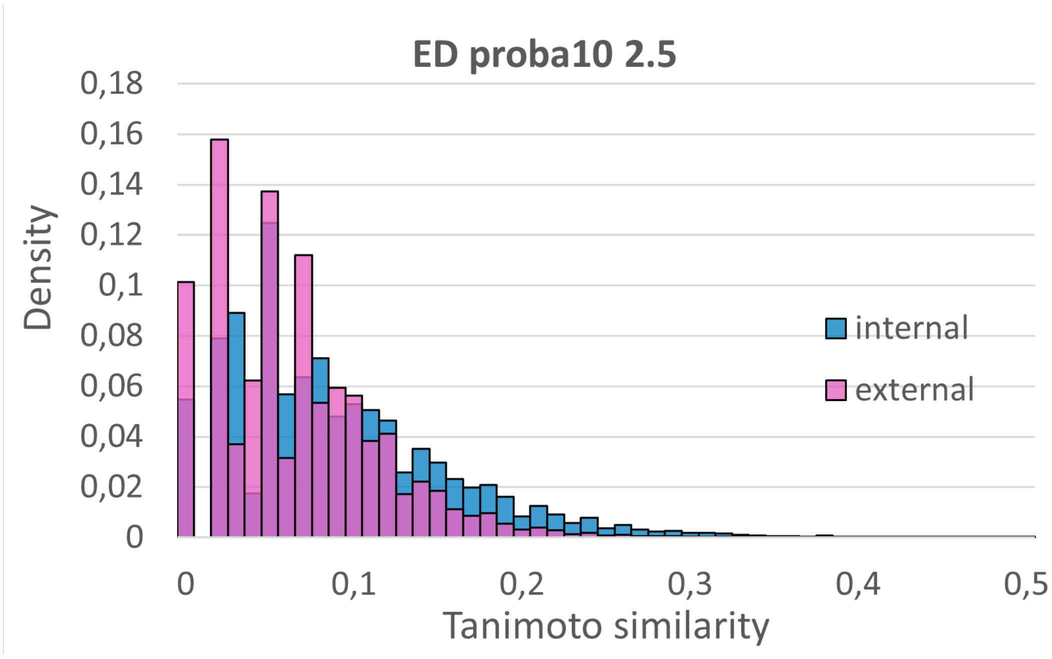

Supplementary Figure 276: Diversity study of the molecules from *ED proba10 2.5*. Internal similarity is where molecules are compared with each other, while external is where molecules are compared with the training set.

**rank**	**molecule**	**occurrences**	**novel**	**commercially available**	**synthetic availability**
**1.**	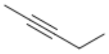	2764	no	**yes**	3.39
**2.**	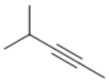	2642	no	**yes**	3.35
**3.**	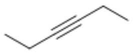	2631	no	**yes**	3.08
**4.**	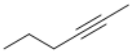	1822	no	**yes**	2.95
**5.**	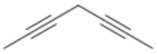	1334	no	no	3.92
**6.**	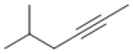	1326	no	no	2.89
**7.**	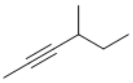	1259	no	no	3.72
**8.**	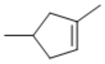	1114	no	no	3.22
**9.**	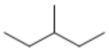	785	no	**yes**	2.14
**10.**	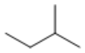	652	no	**yes**	1.68

Supplementary Table 29: Ten most frequently-occurring valid molecules in the ED proba10 2.5 set, ranked by frequency. Synthetic availability was calculated by the means of RDKit.

4.3.6 ED proba10 5.0

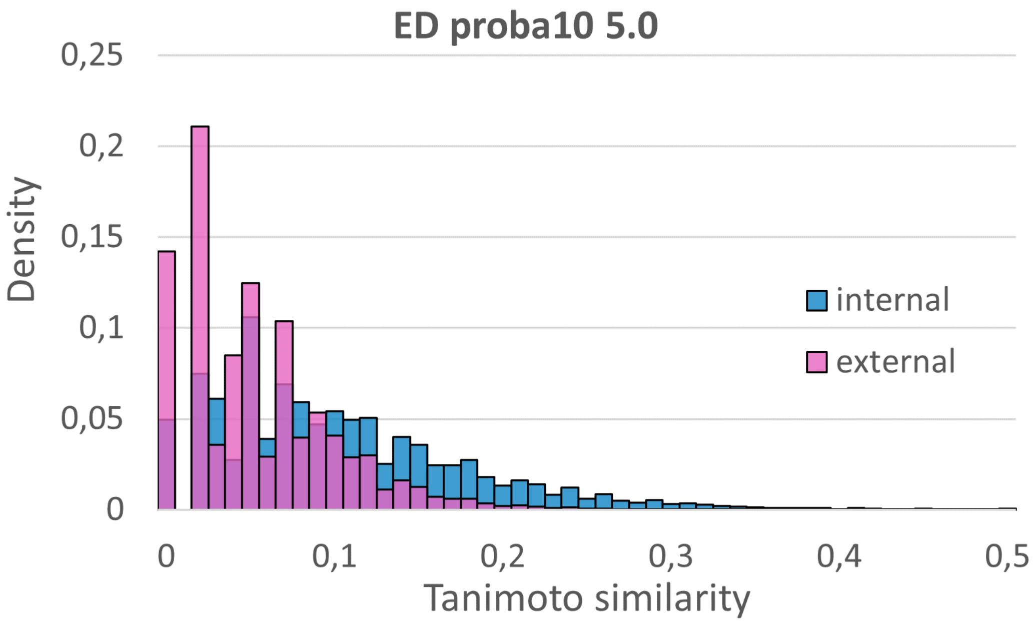

Supplementary Figure 277: Diversity study of the molecules from *ED proba10 5.0*. Internal similarity is where molecules are compared with each other, while external is where molecules are compared with the training set.

rank	molecule	occurrences	novel	commercially available	synthetic availability
**1.**	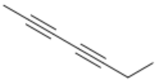	4118	no	no	3.71
**2.**		2899	no	**yes**	3.84
**3.**		2797	no	no	3.99
**4.**	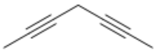	2294	no	no	3.92
**5.**	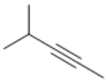	1763	no	**yes**	3.35
**6.**	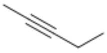	1298	no	**yes**	3.39
**7.**	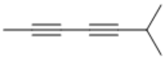	1183	**yes**	no	3.71
**8.**	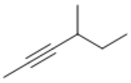	958	no	no	3.72
**9.**	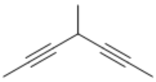	745	no	no	4.30
**10.**		696	no	no	4.07

Supplementary Table 30: Ten most frequently-occurring valid molecules in the ED proba10 5.0 set, ranked by frequency. Synthetic availability was calculated by the means of RDKit.

4.3.7 ED proba10 10.0

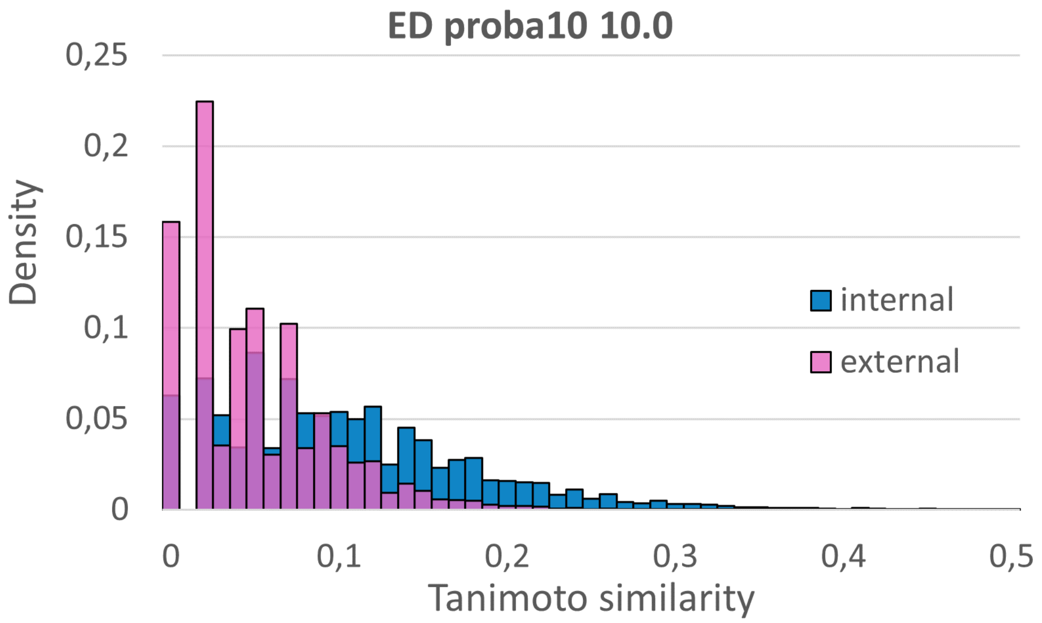

Supplementary Figure 278: Diversity study of the molecules from *ED proba10 10.0*. Internal similarity is where molecules are compared with each other, while external is where molecules are compared with the training set.

rank	molecule	occurrences	novel	commercially available	synthetic availability
**1.**		5686	no	no	3.99
**2.**		3216	no	yes	3.84
**3.**	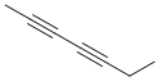	2484	no	no	3.71
**4.**		1818	no	no	4.07
**5.**	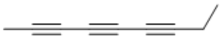	1713	no	no	3.88
**6.**	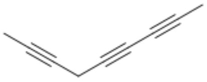	875	no	no	4.13
**7.**		848	no	no	4.16
**8.**	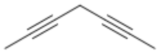	822	no	no	3.92
**9.**		743	no	no	4.25
**10.**		682	no	no	4.20

Supplementary Table 31: Ten most frequently-occurring valid molecules in the ED proba10 10.0 set, ranked by frequency. Synthetic availability was calculated by the means of RDKit.

4.3.8 ED proba10 25.0

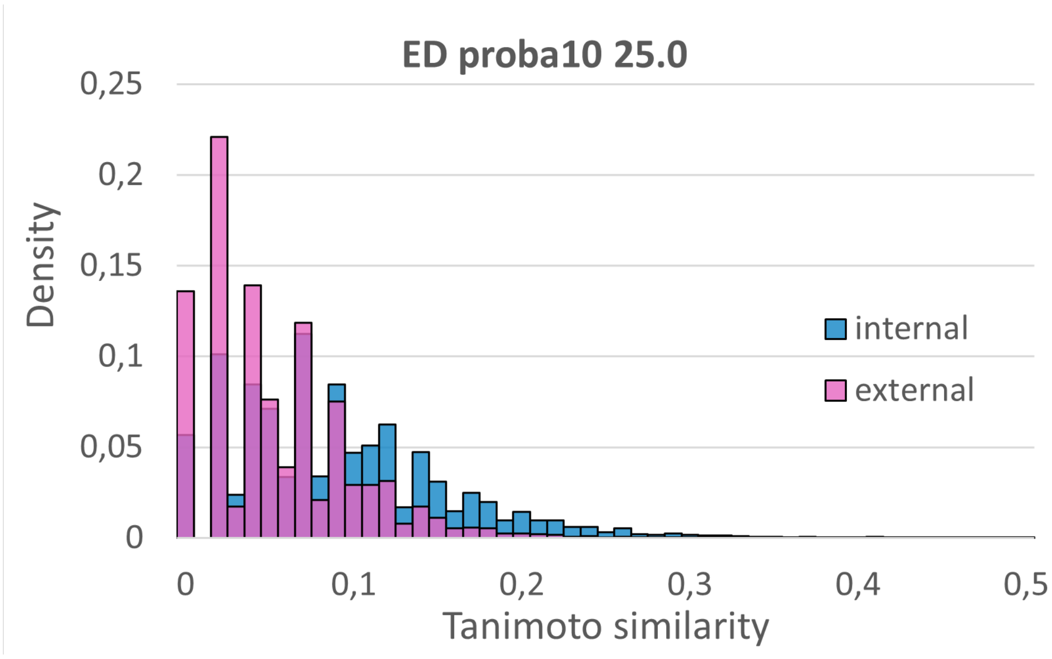

Supplementary Figure 279: Diversity study of the molecules from *ED proba10 25.0*. Internal similarity is where molecules are compared with each other, while external is where molecules are compared with the training set.

rank	molecule	occurrences	novel	commercially available	synthetic availability
**1.**		2081	no	no	3.99
**2.**	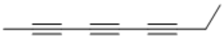	1165	no	no	3.88
**3.**		1020	no	**yes**	3.84
**4.**		720	no	no	4.16
**5.**		686	no	no	4.07
**6.**	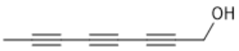	609	no	no	3.71
**7.**	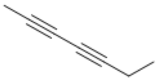	609	no	no	3.84
**8.**	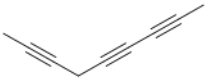	600	no	no	4.13
**9.**		496	no	no	4.25
**10.**	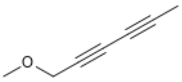	376	no	no	3.55

Supplementary Table 32: Ten most frequently-occurring valid molecules in the ED proba10 25.0 set, ranked by frequency. Synthetic availability was calculated by the means of RDKit.

Supplementary Subsection 4.4 ED decorated with Electrostatic Potentials (ED ESP) Probabilistic Sampling (proba10)

Here is the analysis of **ED ESP proba10**. Its results comprise nine datasets of 40,000 SMILES each, together representing an STD range from **0.5** to **50.0**.

**ED ESP proba10**	**unique**		**valid**	
**std**	**count**	**fraction**	**count**	**fraction**
**0.5**	7514	0.19	2978	0.07
**1.0**	10227	0.26	5091	0.13
**1.5**	14629	0.37	8126	0.20
**2.0**	18608	0.47	10958	0.27
**2.5**	20722	0.52	12380	0.31
**5.0**	19374	0.48	9176	0.23
**10.0**	18171	0.45	7082	0.18
**25.0**	20922	0.52	9548	0.24
**50.0**	27088	0.68	15305	0.38

Supplementary Table 33: Number and fraction of unique and unique valid molecules in datasets generated by the means of ED ESP proba10 method. Each dataset corresponds to a different RNG StD and includes a total of 40,000 non-unique SMILES.

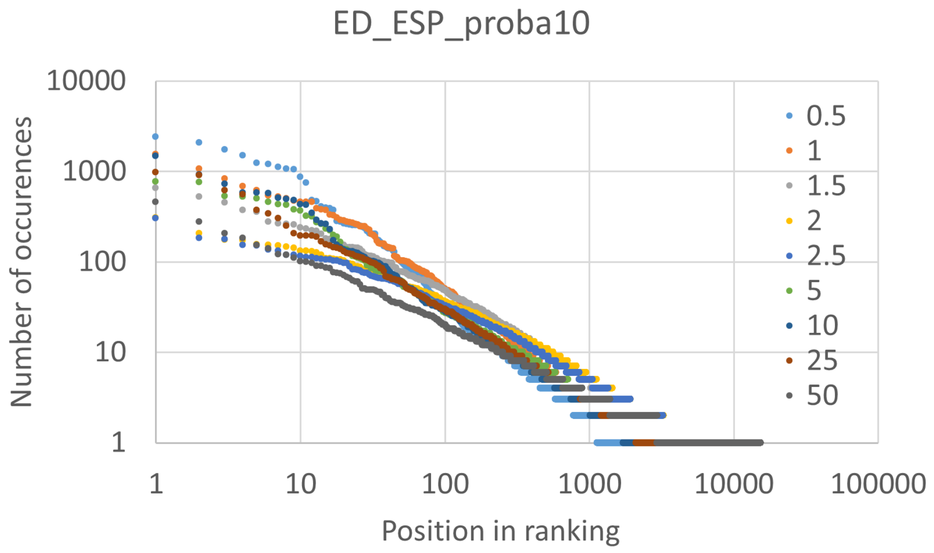

Supplementary Figure 280: Frequency of occurrence of specific valid SMILES in produced results. The plot ranks SMILES according to the number of times they appear in sets of 40,000 generated SMILES. Invalid SMILES are omitted.

ED ESP proba10	total	passes MolGen filters		novel vs training set		novel vs test set		commercially available	
std		count	fraction	count	fraction	count	fraction	count	fraction
**0.5**	2978	2792	0.94	2417	0.81	2912	0.98	242	0.08
**1.0**	5091	4801	0.94	4126	0.81	4978	0.98	534	0.10
**1.5**	8126	7700	0.95	6695	0.82	7962	0.98	801	0.10
**2.0**	10958	10455	0.95	9213	0.84	10775	0.98	876	0.08
**2.5**	12380	11968	0.97	10473	0.85	12205	0.99	771	0.06
**5.0**	9176	8984	0.98	7836	0.85	9068	0.99	244	0.03
**10.0**	7082	6887	0.97	6121	0.86	6981	0.99	137	0.02
**25.0**	9548	9411	0.99	8054	0.84	9393	0.98	130	0.01
**50.0**	15305	15053	0.98	10861	0.71	14892	0.97	341	0.02

Supplementary Table 34: Number and fraction of chemically possible and novel molecules in each set of unique valid SMILES corresponding to specific RNG StD used by the ED ESP proba10 method.

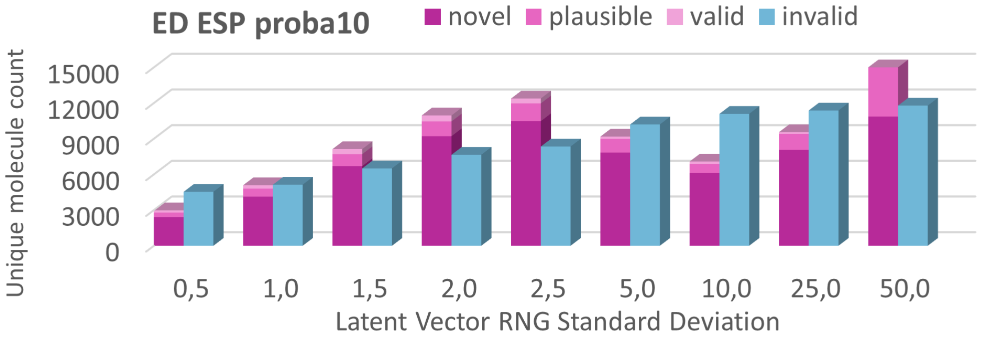

Supplementary Figure 281: Total counts of valid and invalid unique molecules. Total height of pink column represents the unique valid SMILES count, where valid molecules may be chemically plausible and/or novel (as shown by different shades of pink that overlap each other from the bottom-up).

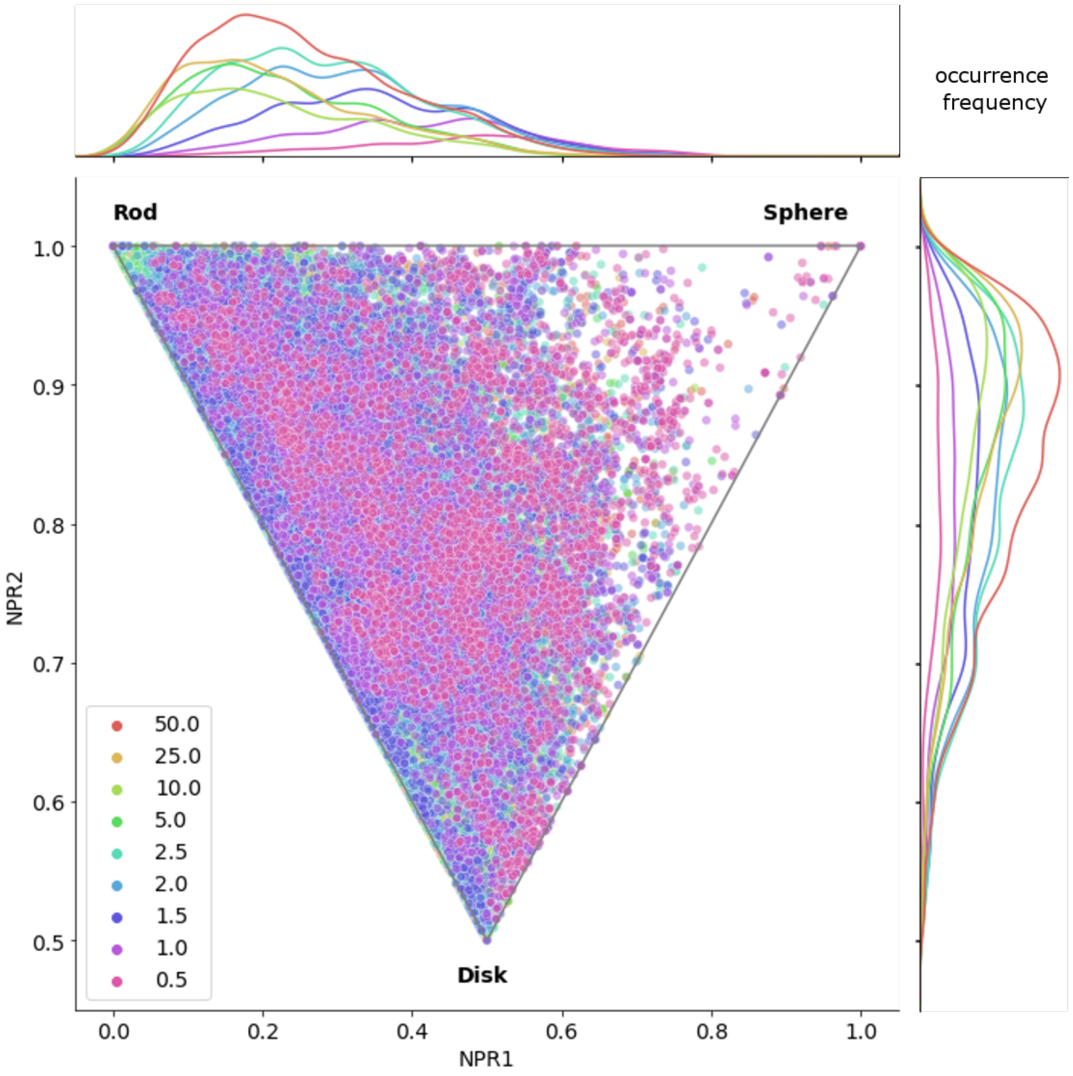

Supplementary Figure 282: Shapes of molecules from the ED ESP proba10 sets according to RDKit NPR calculations. Each dot represents a different unique SMILES and the colors correspond to different RNG StD sets. For clarity purposes, plots have been added to the sides to show the spatial distributions of colored dots. Only those SMILES which geometry could be successfully determined by RDKit and/or OpenBabel tools were included in the plot.

4.4.1 ED ESP proba10 0.5

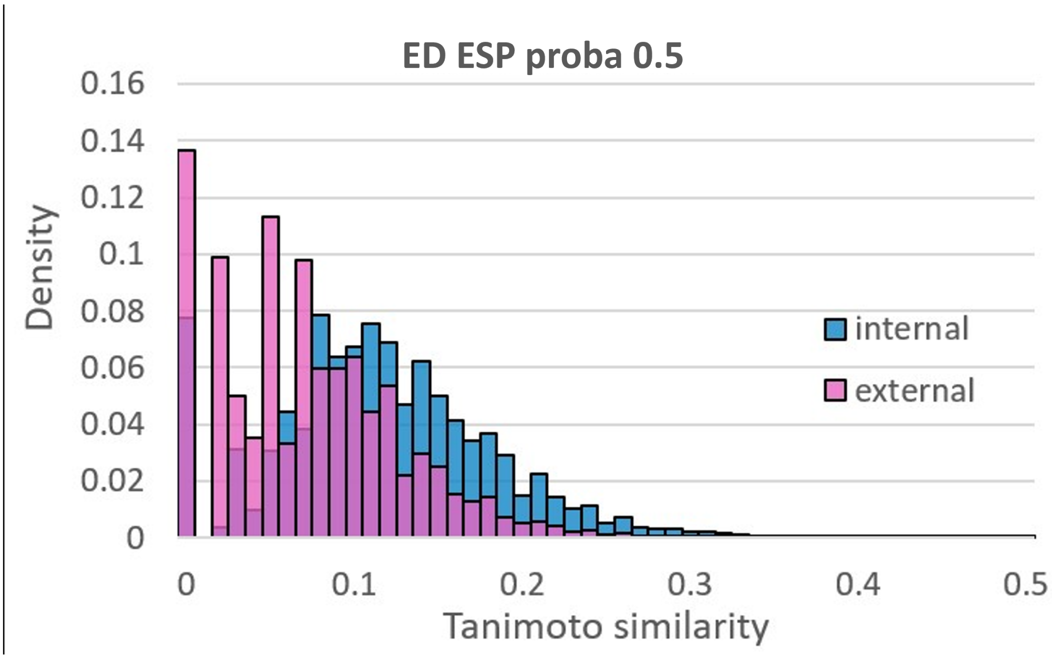

Supplementary Figure 283: Diversity study of the molecules from *ED ESP proba10 0.5*. Internal similarity is where molecules are compared with each other, while external is where molecules are compared with the training set.

rank	molecule	occurrences	novel	commercially available	synthetic availability
**1.**	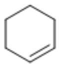	2418	no	**yes**	2.42
**2.**	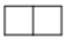	2063	no	no	1.90
**3.**	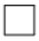	1739	no	no	1.00
**4.**	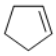	1499	no	**yes**	2.73
**5.**	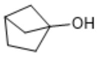	1236	no	no	3.92
**6.**	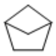	1203	no	no	2.80
**7.**	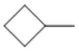	1120	**yes**	no	1.42
**8.**	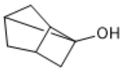	1060	no	no	5.69
**9.**	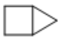	1056	no	no	2.78
**10.**	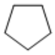	863	no	**yes**	1.00

Supplementary Table 35: Ten most frequently-occurring valid molecules in the ED ESP proba10 0.5 set, ranked by frequency. Synthetic availability was calculated by the means of RDKit.

4.4.2 ED ESP proba10 1.0

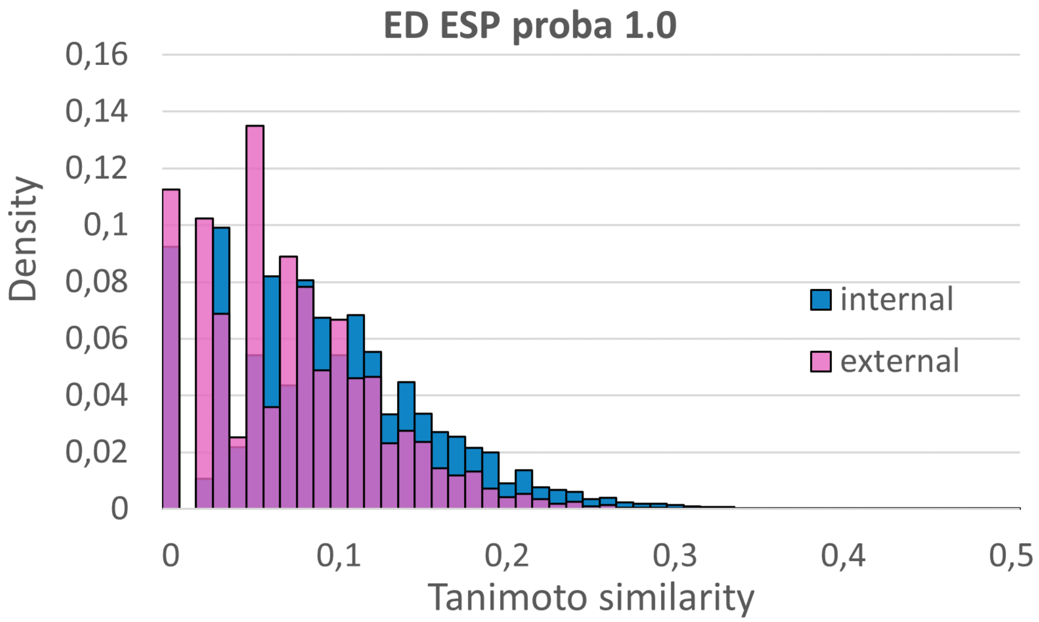

Supplementary Figure 284: Diversity study of the molecules from *ED ESP proba10 1.0*. Internal similarity is where molecules are compared with each other, while external is where molecules are compared with the training set.

rank	molecule	occurrences	novel	commercially available	synthetic availability
**1.**	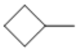	1549	**yes**	no	1.42
**2.**	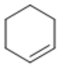	1069	no	**yes**	2.42
**3.**	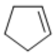	833	no	**yes**	2.73
**4.**	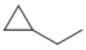	687	no	no	1.70
**5.**	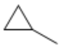	612	**yes**	no	1.37
**6.**	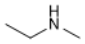	540	**yes**	**yes**	2.31
**7.**	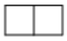	522	no	no	1.90
**8.**	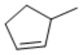	503	no	no	3.53
**9.**	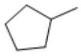	474	no	**yes**	1.57
**10.**	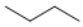	460	no	**yes**	1.61

Supplementary Table 36: Ten most frequently-occurring valid molecules in the ED ESP proba10 1.0 set, ranked by frequency. Synthetic availability was calculated by the means of RDKit.

4.4.3 ED ESP proba10 1.5

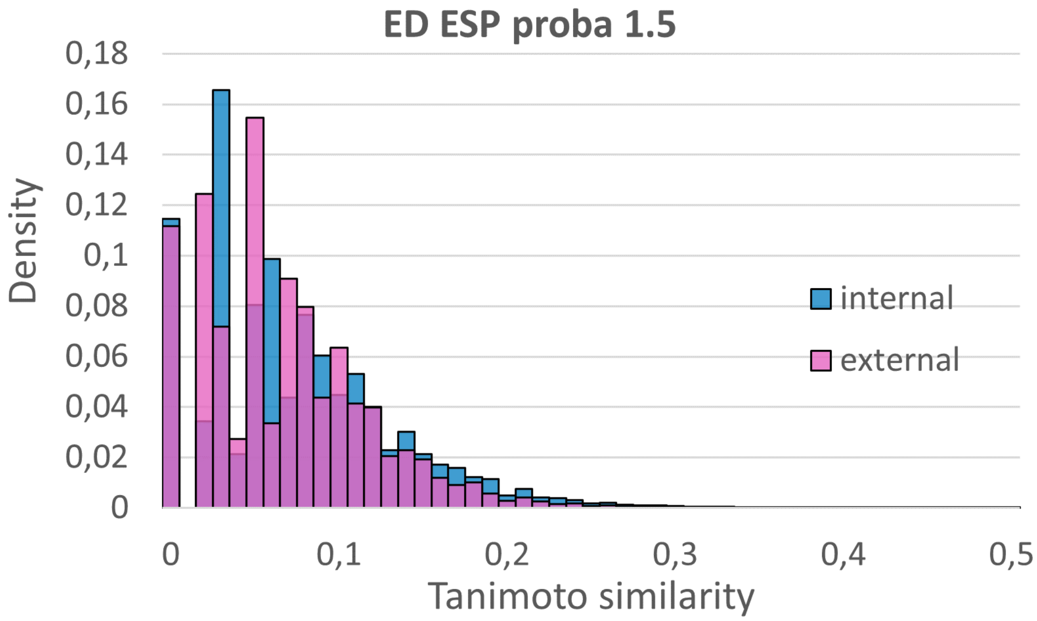

Supplementary Figure 285: Diversity study of the molecules from *ED ESP proba10 1.5*. Internal similarity is where molecules are compared with each other, while external is where molecules are compared with the training set.

rank	molecule	occurrences	novel	commercially available	synthetic availability
**1.**	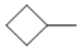	654	**yes**	**yes**	2.31
**2.**	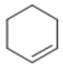	522	no	no	3.06
**3.**	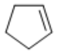	450	no	no	1.70
**4.**	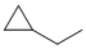	371	**yes**	no	1.37
**5.**	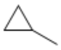	357	**yes**	no	1.42
**6.**	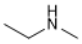	276	**yes**	**yes**	2.23
**7.**	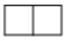	263	**yes**	no	2.93
**8.**	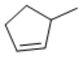	262	**yes**	**yes**	2.10
**9.**	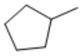	256	no	**yes**	2.73
**10.**	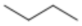	237	no	no	3.53

Supplementary Table 37: Ten most frequently-occurring valid molecules in the ED ESP proba10 1.5 set, ranked by frequency. Synthetic availability was calculated by the means of RDKit.

4.4.4 ED ESP proba10 2.0

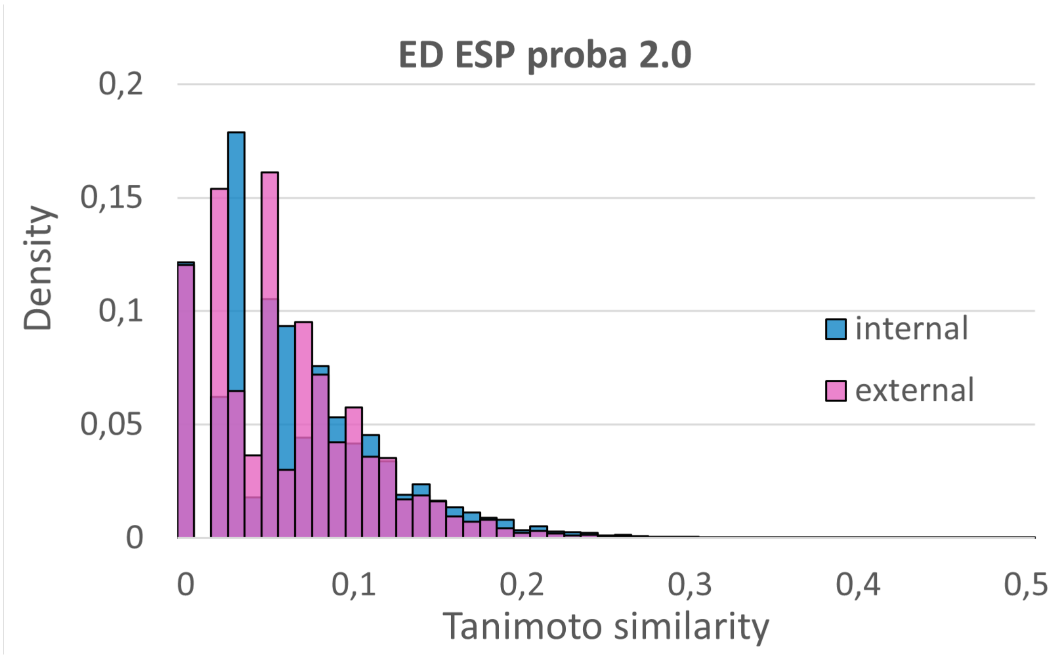

Supplementary Figure 286: Diversity study of the molecules from *ED ESP proba10 2.0*. Internal similarity is where molecules are compared with each other, while external is where molecules are compared with the training set.

rank	molecule	occurrences	novel	commercially available	synthetic availability
**1.**	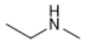	309	**yes**	**yes**	2.31
**2.**	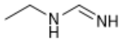	207	**yes**	no	4.32
**3.**	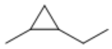	174	no	no	3.06
**4.**	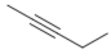	172	no	**yes**	3.39
**5.**	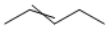	155	**yes**	**yes**	2.88
**6.**	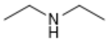	152	**yes**	**yes**	2.14
**7.**	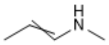	151	**yes**	no	3.79
**8.**	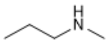	146	**yes**	**yes**	2.23
**9.**	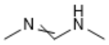	142	**yes**	no	4.34
**10.**	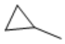	131	**yes**	no	3.58

Supplementary Table 38: Ten most frequently-occurring valid molecules in the ED ESP proba10 2.0 set, ranked by frequency. Synthetic availability was calculated by the means of RDKit.

4.4.5 ED ESP proba10 2.5

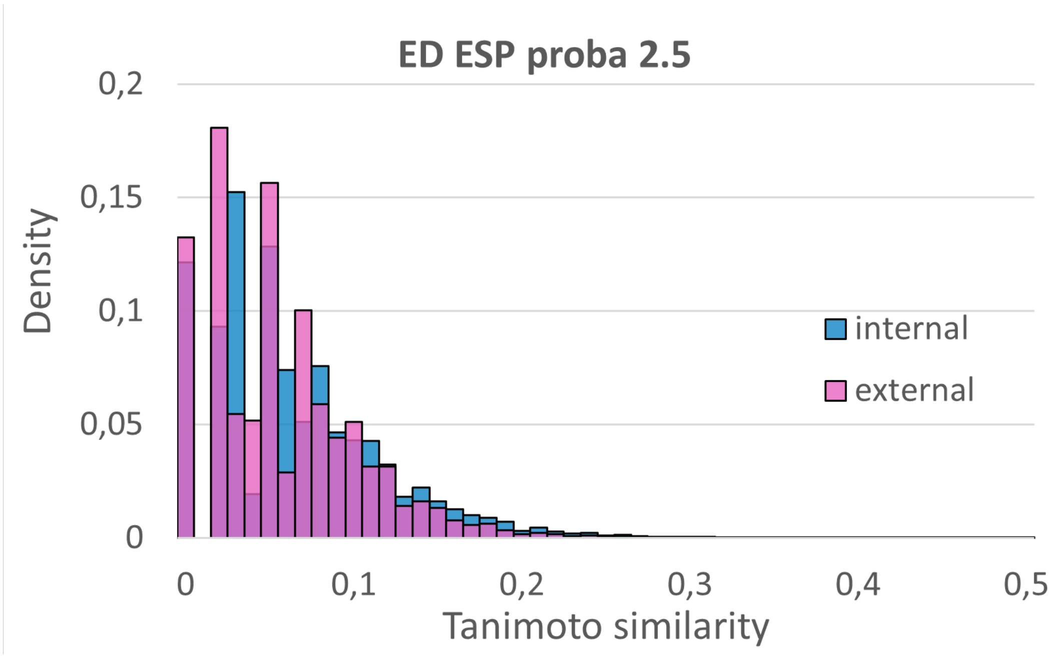

Supplementary Figure 287: Diversity study of the molecules from *ED ESP proba10 2.5*. Internal similarity is where molecules are compared with each other, while external is where molecules are compared with the training set.

**rank**	**molecule**	**occurrences**	**novel**	**commercially available**	**synthetic availability**
**1.**	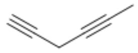	301	**yes**	no	4.10
**2.**	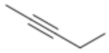	184	no	**yes**	3.39
**3.**	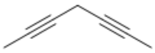	177	no	no	3.92
**4.**	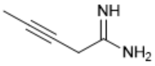	154	**yes**	no	3.88
**5.**	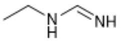	151	**yes**	no	4.32
**6.**	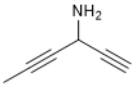	136	**yes**	no	4.99
**7.**	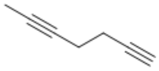	132	no	no	3.62
**8.**	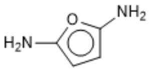	119	**yes**	no	4.34
**9.**	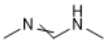	119	no	no	3.43
**10.**	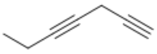	115	no	no	3.83

Supplementary Table 39: Ten most frequently-occurring valid molecules in the ED ESP proba10 2.5 set, ranked by frequency. Synthetic availability was calculated by the means of RDKit.

4.4.6 ED ESP proba10 5.0

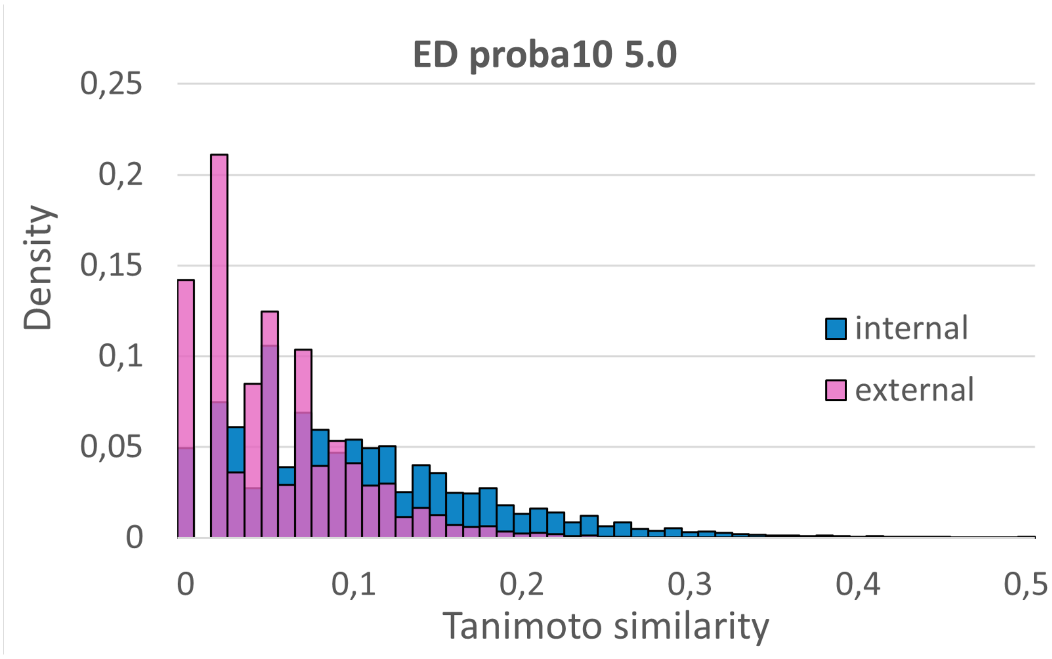

Supplementary Figure 288: Diversity study of the molecules from *ED ESP proba10 5.0*. Internal similarity is where molecules are compared with each other, while external is where molecules are compared with the training set.

rank	molecule	occurrences	novel	commercially available	synthetic availability
**1.**		764	no	no	4.07
**2.**	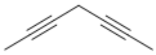	760	no	no	3.92
**3.**	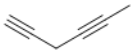	528	**yes**	no	4.10
**4.**		523	no	no	3.99
**5.**		499	no	no	4.20
**6.**	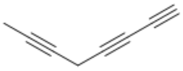	460	no	no	4.27
**7.**	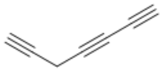	430	no	no	4.46
**8.**	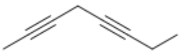	423	no	no	3.73
**9.**	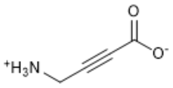	380	no	no	5.73
**10.**		369	no	no	4.20

Supplementary Table 40: Ten most frequently-occurring valid molecules in the ED ESP proba10 5.0 set, ranked by frequency. Synthetic availability was calculated by the means of RDKit.

4.4.7 ED ESP proba10 10.0

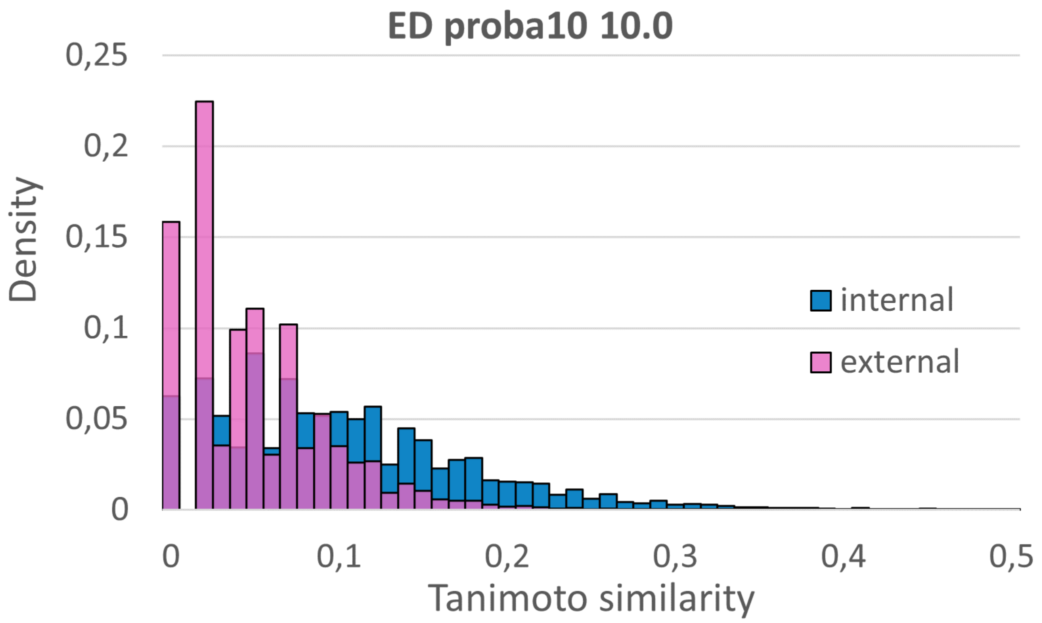

Supplementary Figure 289: Diversity study of the molecules from *ED ESP proba10 10.0*. Internal similarity is where molecules are compared with each other, while external is where molecules are compared with the training set.

rank	molecule	occurrences	novel	commercially available	synthetic availability
**1.**		1472	no	no	3.99
**2.**		912	no	no	4.07
**3.**	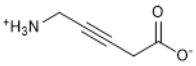	726	no	no	5.49
**4.**	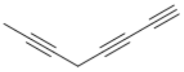	580	no	no	4.27
**5.**	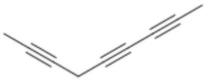	577	no	no	4.13
**6.**		569	no	no	4.20
**7.**	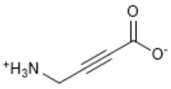	504	no	no	5.73
**8.**	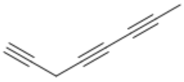	490	**yes**	no	4.27
**9.**	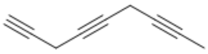	482	no	no	4.23
**10.**	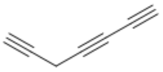	430	no	no	4.46

Supplementary Table 41: Ten most frequently-occurring valid molecules in the ED ESP proba10 10.0 set, ranked by frequency. Synthetic availability was calculated by the means of RDKit.

4.4.8 ED ESP proba10 25.0

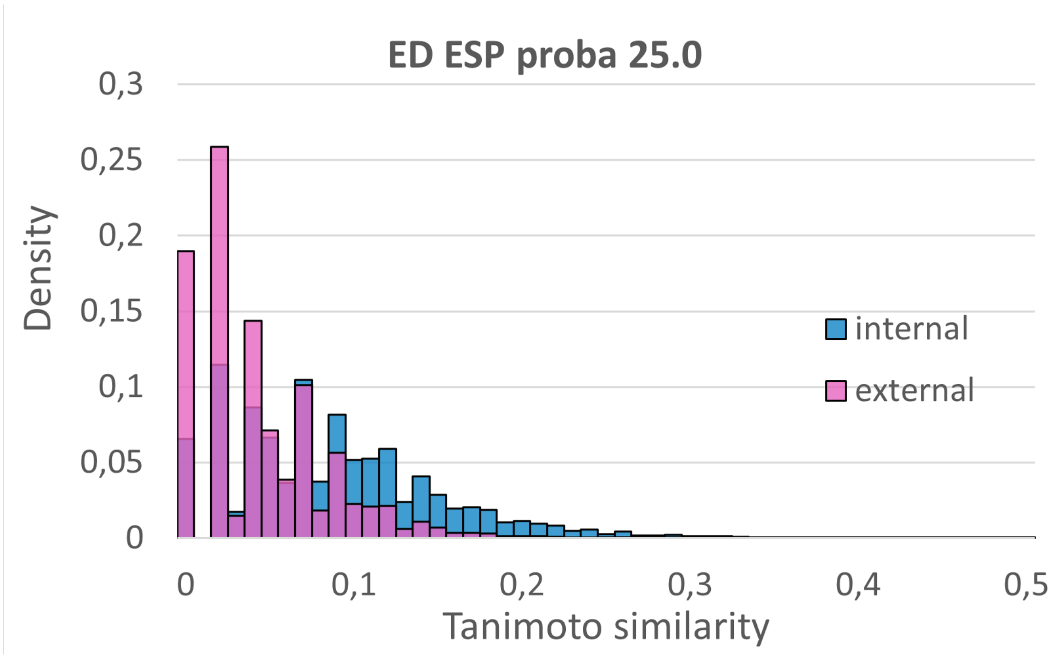

Supplementary Figure 290: Diversity study of the molecules from *ED ESP proba10 25.0*. Internal similarity is where molecules are compared with each other, while external is where molecules are compared with the training set.

rank	molecule	occurrences	novel	commercially available	synthetic availability
**1.**	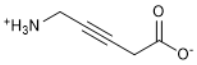	978	no	no	5.49
**2.**		905	no	no	3.99
**3.**	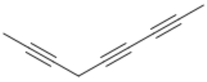	618	no	no	4.13
**4.**	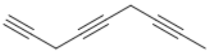	551	no	no	4.23
**5.**		373	no	no	4.07
**6.**	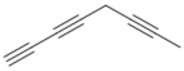	341	no	no	4.27
**7.**	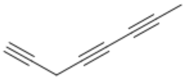	302	**yes**	no	4.27
**8.**	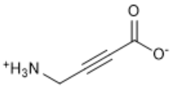	250	no	no	5.73
**9.**	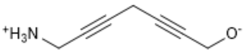	205	**yes**	no	5.64
**10.**	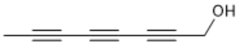	193	no	no	3.72

Supplementary Table 42: Ten most frequently-occurring valid molecules in the ED ESP proba10 25.0 set, ranked by frequency. Synthetic availability was calculated by the means of RDKit.

4.4.9 ED ESP proba10 50.0

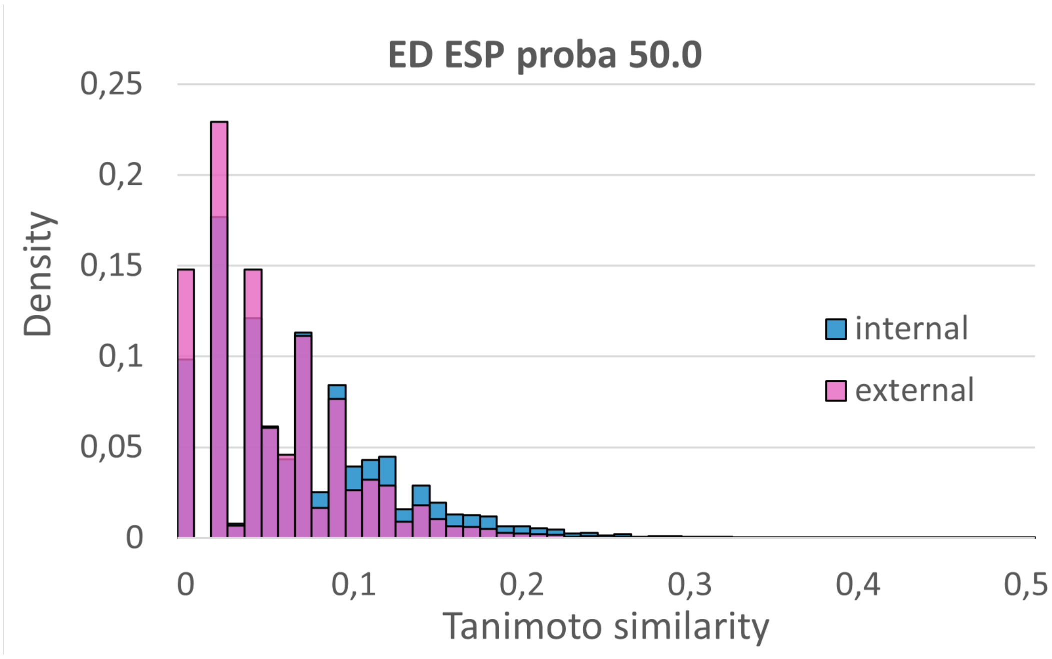

Supplementary Figure 291: Diversity study of the molecules from *ED ESP proba10 50.0*. Internal similarity is where molecules are compared with each other, while external is where molecules are compared with the training set.

rank	molecule	occurrences	novel	commercially available	synthetic availability
**1.**	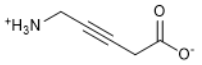	457	no	no	5.49
**2.**	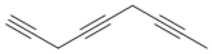	278	no	no	4.23
**3.**	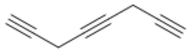	206	no	no	4.39
**4.**	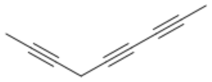	183	no	no	4.13
**5.**	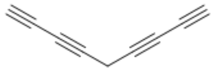	154	no	no	4.53
**6.**	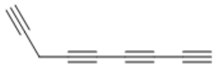	140	no	no	4.41
**7.**	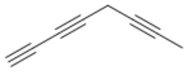	121	no	no	4.27
**8.**	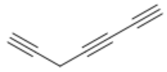	118	no	no	4.46
**9.**	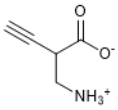	110	no	no	5.97
**10.**	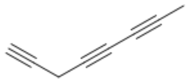	101	**yes**	no	4.27

Supplementary Table 43: Ten most frequently-occurring valid molecules in the ED ESP proba10 50.0 set, ranked by frequency. Synthetic availability was calculated by the means of RDKit.

Supplementary Subsection 4.5 Benchmarking the novelty of the generated guests against the QM9 dataset

4.5.1 Benchmarking literature known CB[6] guests against the QM9 dataset

The most complete dataset of guests for the CB[6] can be found in the research done by Mock, *et al.*[7]. In this paper, the authors describe 53 different guests. Out of these 53 guests, three of them are molecules present in the QM9 dataset. See Supplementary Tables 44 and 45.

**CB[6] guests from [7]**	**Present in the QM9 dataset? (1 yes, 0 no)**
NC1CCCCC1	0
NCC1=CC=CS1	0
NCCC1OCC1	0
NCCOCCN	0
OCCCCCCN	0
NCCOCC	0
COCCN	0
NCCSCCN	0
NCCCSCC	0
CSCCCN	0
NCCSCC	0
CSCCN	0
NCC1=CC=C(C)C=C1	0
NCC1=CC=CC=C1	0
NC1=CC=C(C)C=C1	1
NC1=CC=CC=C1	1
NCCCNCCCCNCCCN	0
NCCCCNCCCN	0
CCNCCCCCCNCC	0
CNCCCCCCNC	0
CCCCNCC	0
CCCCNC	0
NCC1CCCC1	0
NCC1CCC1	0
NCC1CC1	0
NC1CCCC1	0

Supplementary Table 44: Benchmarking the CB[6] guests described in [7] against the QM9 dataset.

4.5.2 Bencharmking literature known $[Pd_2\mathbf{1}_4](BArF)_4$ Cage guests against the QM9 dataset

The most complete dataset of guests for the Pd Cage can be found in the research done by Liao, *et al.*[4], and August *et al.*[5]. In these papers, the authors describe a total of 14

CB[6] guests from [7]	Present in the QM9 dataset? (1 yes, 0 no)
NC1CCC1	0
NC1CC1	0
CC(C)(C)CCN	0
NC(C)CCCCCC	0
NC(C)CCCCC	0
NC(C)CCCC	0
CCC(C)CCN	0
CCC(C)CN	0
CC(C)CCCCN	0
CC(C)CCCN	0
CC(C)CCN	0
NCCCCCCCCCCN	0
NCCCCCCCCCN	0
NCCCCCCCCN	0
NCCCCCCCN	0
NCCCCCCN	0
NCCCCCN	0
NCCCCN	0
NCCCN	0
CCCCCCCN	0
CCCCCCN	0
CCCCCN	0
CCCCN	0
CCCN	0
CCN	0
CN	0
N	1

Supplementary Table 45: Benchmarking the CB[6] guests described in [7] against the QM9 dataset.

guests. Out of these 14 guests, four of them are molecules present in the QM9 dataset. See Supplementary Table 46.

4.5.3 Bencharmking the molecules generated in this research through AI against the QM9 dataset

The main manuscript describes a total of 20 guests found using the AI: 16 for CB[6] and 4 for the Pd Cage. Out of the 16 CB[6] guests, one of them is present in the QM9 dataset, while 15 of them are not. See Supplementary Table 47. Out of the 4 PD Cage guests, all of them are present in the QM9. See Supplementary Table 48.

Pd Cage guests from [4],[5]	Present in the QM9 dataset?
N#CC1=CC=C(C#N)C=C1	0
N#CC1=CC=C(Cl)C=C1	0
N#CC1=CC=CC=C1	1
CC1=CC=C(C#N)C=C1	1
FC1=CC=C(F)C=C1	1
O=C(C=C1)C=CC1=O	0
[H]C(C(C=CC=C1)=C1[N+]([O-])=O)=O	0
[H]C(C(C=CC=C1)=C1C([H])=O)=O	0
O=C(CN1)NCC1=O	1
O=C(C(C=C(C=CC=C1)C1=C2)=C2C3=O)<br>C4=C3C=C(C=CC=C5)C5=C4	0
O=C(C(C=C(N)C=C1)=C1C2=O)C3=C2C=CC=C3	0
O=C(C(C=CC=C1)=C1C2=O)C3=C2C=CC=C3	0
O=C(C=CC1=O)C2=C1C=CC=C2	0
O=C(C=C1)C=CC1=O	0

Supplementary Table 46: Benchmarking the Pd Cage guests described in [4] and [5] against the QM9 dataset. Please note the cell with two rows contains a single molecule. This molecule was cut into two lines so that it fitted horizontally.

AI-discovered CB[6] guests	Present in the QM9 dataset?
Already known in literature	
CCCCC(C)N	0
CCC(C)CN	0
NCC1CC1	0
CCCC(C)N	0
CCCCN	0
CCN	0
CCCN	0
NC1=CC=CC=C1	1
NC1CC1	0
New (not in literature)	
CCCNC	0
CCNC	0
CCC(N)CC	0
CCC(C)NC	0
CCC(C)N	0
CC(C)N	0
CC(C)NCC#C	0

Supplementary Table 47: Benchmarking the CB[6] guests discovered by our AI models against the QM9 dataset.

AI-discovered Pd Cage guests	Present in the QM9 dataset?
CC1=CC(C#N)=C(C)N1	1
CC1=NC=CN=C1C#C	1
CC1C(C)=CC(C1)=O	1
CC1=CC=CC(C#N)=C1	1

Supplementary Table 48: Benchmarking the Pd Cage guests discovered by our AI models against the QM9 dataset.

References

[1] Han Zhang, Ian Goodfellow, Dimitris Metaxas, and Augustus Odena. Self-Attention Generative Adversarial Networks. In *Proc. 36th Int. Conf. Mach. Learn.*, volume 97, pages 7354–7363, 2019.

[2] Sandro Mecozzi and Julius Rebek. The 55% solution: A formula for molecular recognition in the liquid state. *Chem. - A Eur. J.*, 4(6):1016–1022, 1998.

[3] Alec Radford, Karthik Narasimhan, Tim Salimans, and Ilya Sutskever. Improving language understanding by generative pre-training. *OpenAI*, 2018.

[4] Puhong Liao, Brian W. Langloss, Amber M. Johnson, Eric R. Knudsen, Fook S. Tham, Ryan R. Julian, and Richard J. Hooley. Two-component control of guest binding in a self-assembled cage molecule. *Chem. Commun.*, 46(27):4932–4934, 2010.

[5] David P August, Gary S Nichol, and Paul J Lusby. Maximizing Coordination Capsule – Guest Polar Interactions in Apolar Solvents Reveals Significant Binding. *Angew. Chemie - Int. Ed.*, 55(15022):15026, 2016.

[6] Yana R. Hristova, Maarten M.J. Smulders, Jack K. Clegg, Boris Breiner, and Jonathan R. Nitschke. Selective anion binding by a "Chameleon" capsule with a dynamically reconfigurable exterior. *Chem. Sci.*, 2(4):638–641, 2011.

[7] William L. Mock and Neng Yang Shih. Structure and Selectivity in Host-Guest Complexes of Cucurbituril. *J. Org. Chem.*, 51(23):4440–4446, 1986.
